# Glycolysis-mediated H3K18la modifications drive aggressive bladder cancer through metabolic and epigenetic reprogramming

**DOI:** 10.3389/fimmu.2026.1777092

**Published:** 2026-06-30

**Authors:** Zhan Wang, Zhaokai Zhou, Shuai Yang, Zihao Zhao, Xiaozu Li, Xingchen Liu, Guangyang Cheng, Ran Xu, Qi Li, Dong Xing

**Affiliations:** 1 Department of Urology, The First Affiliated Hospital of Zhengzhou University, Zhengzhou, China; 2 Department of Urology, Sun Yat-sen University Cancer Center, State Key Laboratory of Oncology in South China, Collaborative Innovation Center for Cancer Medicine, Guangzhou, China; 3 Department of Urology, The Second Xiangya Hospital of Central South University, Changsha, Hunan, China; 4 National Clinical Research Center for Metabolic Diseases, The Second Xiangya Hospital of Central South University, Changsha, Hunan, China; 5 Department of Pediatric Urology, Guangzhou Medical University Affiliated Women and Children’s Medical Center, Guangzhou, Guangdong, China; 6 Department of Radiology, The First Affiliated Hospital of Zhengzhou University, Zhengzhou University, Zhengzhou, China; 7 The First Clinical College of Zhengzhou University, Zhengzhou University, Zhengzhou, China; 8 Department of Obstetrics and Gynecology, Xinyang Central Hospital, Xinyang, Henan, China; 9 Department of Pediatric Surgery, The First Affiliated Hospital of Zhengzhou University, Zhengzhou, China; 10 Department of Emergency Intensive Care Unit (ICU), The First Affiliated Hospital of Zhengzhou University, Zhengzhou, China

**Keywords:** bladder cancer, epigenetic reprogramming, glycolysis, H3K18la, histone lactylation

## Abstract

**Background:**

Metabolic reprogramming and epigenetic alterations contribute to the aggressiveness of human bladder cancer (BC). Lactate-dependent histone modification represents a novel class of histone marks that links the glycolytic metabolite to the epigenetic mechanism of lactylation. However, the role of histone lactylation in BC remains unclear.

**Materials and methods:**

The single-cell RNA sequencing dataset GSE135337 was analyzed to assess glycolysis and histone lactylation levels in BC samples. Subsequently, western blotting and immunofluorescence analyses were employed to detect the levels of histone lactylation in BC. The inhibition of histone lactylation, achieved via glycolysis inhibitors or lactate dehydrogenase A (LDHA) knockdown, was confirmed to impede BC growth and progression in both *in vitro* and *in vivo* studies. Potential target genes of H3K18 lactylation (H3K18la) were screened through CUT&Tag and RNA-seq analyses.

**Results:**

The study identified a notable increase in glycolytic activity and histone lactylation, especially H3K18la, which correlated with poor prognosis in BC patients. Inhibiting glycolytic activity through various inhibitors or LDHA knockdown led to anti-tumor effects in BC in both *in vitro* and *in vivo* studies. CUT&Tag of H3K18la combined with RNA-seq analysis identified four potential target genes (AHNAK2, PVR, SLC7A11, and SREBF1). These genes were found to be associated with the growth and invasion of BC potentially through complex metabolic regulatory mechanisms within the tumor microenvironment.

**Conclusion:**

Glycolysis closely linked to H3K18la enrichment at the AHNAK2, PVR, SLC7A11, and SREBF1 loci, established a correlative epigenetic network that accompanies aggressive BC progression. These findings reveal important connections between lactate metabolism reprogramming and epigenetic regulation, potentially leading to new therapeutic strategies targeting lactylation in BC treatment.

## Graphical Abstract

**Figure:**
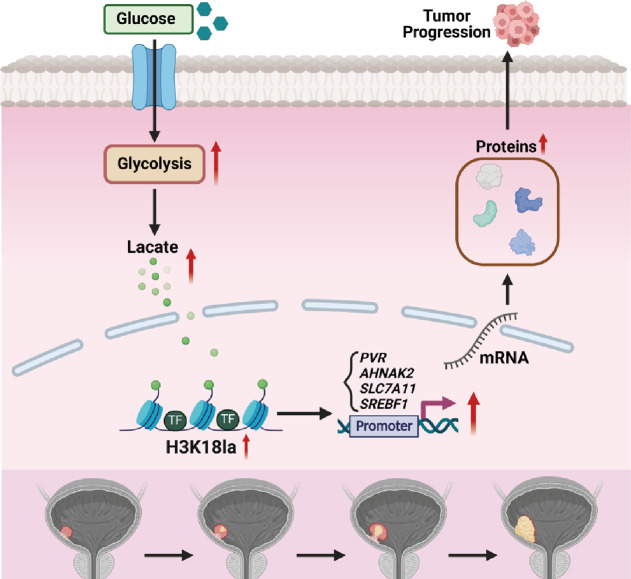

## Highlights

- Elevated histone lactylation (H3K18la) linked to glycolysis is associated with poor prognosis in bladder cancer (BC), highlighting its potential as a prognostic marker.
- Inhibition of glycolysis or LDHA knockdown suppresses BC growth, revealing lactate metabolism as a potential therapeutic target for anti-tumor strategies.
- H3K18la is positively correlated with the expression of key oncogenic genes (AHNAK2, PVR, SLC7A11, SREBF1), driving BC progression through metabolic and immunosuppressive mechanisms.

## 1 Introduction

Bladder cancer (BC), a prevalent urinary tract tumor, is noted for its high aggressiveness and unique histological, molecular, and clinical characteristics (1, 2). Due to the lack of effective therapeutic strategies, the overall 5-year survival rate for BC remains poor, and the clinical management of BC still faces many challenges (2). A comprehensive understanding of the mechanisms driving BC progression and metastasis is crucial for identifying novel therapeutic targets and potential prognostic biomarkers (2–4).

The molecular mechanisms underlying BC are similar to those of other malignancies and include DNA repair defects, alterations in cell cycle, dysregulation of protein glycosylation and clearance, as well as metabolic reprogramming events (5). Several studies have shown a direct link between BC’s aggressive tumor biology and metabolic reprogramming (6). Cancer cells are commonly characterized by abnormal oxidative phosphorylation and glucose metabolism (6). Cancer cells meet the increased bioenergetic and biosynthetic needs of tumor growth by enhancing glycolysis and generating substantial amounts of lactate, adopting the Warburg phenotype (5–7). Lactate, a key metabolite in cancer, is produced as the final output of glycolysis (7, 8). The enhancement of glycolysis and the accumulation of lactate are typical characteristics of many cancers (8). To date, lactylation has been widely identified in proteins within cancer cells, particularly histones (9, 10). Driven by lactate, this novel epigenetic modification regulates the biological behaviors of cells by modulating downstream gene transcription (10, 11). However, the mechanisms by which metabolic reprogramming remodels epigenetic events, particularly glycolysis and lactylation, remain unclear in BC. Emerging advancements in immunotherapy and targeted molecular therapy highlight the potential of metabolic reprogramming in BC for developing more effective, personalized treatments (12).

**Figure 1:**
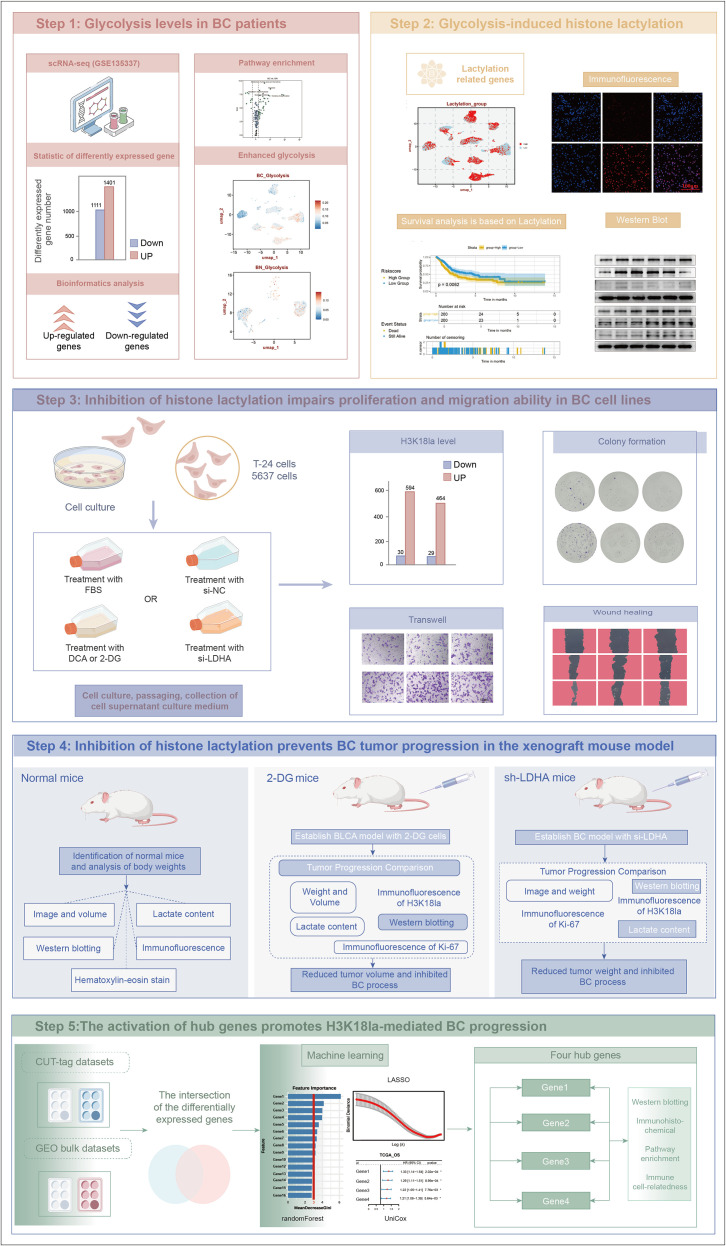
Flowchart illustrating the process by which histone lactylation promotes the malignant progression of BC.

Single-cell RNA sequencing (scRNA-seq) provides unique opportunities to identify cancer cell populations and their markers. It supports the investigation of metabolic changes in the tumor microenvironment and assists in identifying personalized therapeutic targets (13–15). This study analyzed scRNA-seq data of BC, emphasizing histone lactylation, particularly histone H3 lysine 18 lactylation (H3K18la), in the progression of BC. It was demonstrated that the level of glycolysis and lactylation in BC cells was significantly increased. Lactate accumulation drove histone lactylation, which was associated with tumorigenesis and a poor prognosis. Specifically, elevated H3K18la resulting from enhanced glycolysis was positively associated with the transcription of potentially malignant targets (AHNAK2, PVR, SLC7A11, and SREBF1). Our study elucidated the role of H3K18la and its downstream regulatory network in BC progression for the first time. Targeting the glycolysis-H3K18LA pathway and its related genes could offer a promising therapeutic approach for BC treatment. The research process of this study is shown in **Figure 1**.

## 2 Materials and methods

### 2.1 Data sources and analysis of scRNA-seq

The BC scRNA-seq dataset GSE135337, sourced from the Gene Expression Omnibus (GEO) database (https://www.ncbi.nlm.nih.gov/geo/), includes scRNA-seq data from tumor tissues of seven BC patients and one paracancerous tissue sample (**Supplementary Table 1**) (16). The original study has received the necessary ethical approval and fulfilled the informed consent procedures. Seurat (v5.1.0) was utilized to analyze the gene expression matrix (17). Genes found in fewer than three cells were not included in the analysis. Cells were selected if they exhibited between 500 and 6000 detected genes per cell and had mitochondrial gene expression below 10%. Following quality control, 36,407 tumor cells and 6,033 paracancerous cells were retained for analysis. To maintain the unique characteristics of each tumor, datasets from 7 patients’ tumor tissues were not combined. NormalizeData was employed to normalize gene expression across cells, while the top 3000 most variable genes were identified by FindVariableFeatures. Subsequently, principal component analysis (PCA) was performed on the data after it had been scaled using the ScaleData function. The analysis proceeded with the selection of the top 30 principal components (PCs). Using the FindClusters function, 31 distinct cell clusters were identified. The Uniform Manifold Approximation and Projection (UMAP) algorithm was employed to visualize these clusters. The FindAllMarkers function was utilized to identify differentially expressed genes (DEGs) for each cluster (18, 19). DEGs were identified in at least 25% of cells, exhibiting a log fold change of at least 0.5 and an adjusted P-value below 0.05, corrected using the Bonferroni method. Clusters were annotated using established markers: EPCAM, KRT8, and KRT18 for epithelial cells; CD14, CSF1R, and AIF1 for myeloid/macrophages; CD2 and CD3D for T cells; DCN, PDPN, and TAG for fibroblasts; and PECAM1, VWF, and CLDN5 for endothelial cells (16, 20–22). Additionally, Seurat’s CellCycleScoring function was used to score the cell cycle and the single-cell pathway enrichment analysis was conducted using the “SCPA” R package (version 1.6.2).

### 2.2 Analysis of cell-cell communication

The “CellChat” R package (https://github.com/sqjin/CellChat) was utilized to analyze ligand-receptor interactions among the five cell types in BC (23, 24). Cell expression data were employed as the input to construct a cell-cell communication model. This model integrates gene expression profiles with established interaction networks of signaling ligands, receptors, and cofactors. The reference data were sourced from the signal molecule interaction database called CellChatDB.

### 2.3 Lactylation activity scores

The lactylation activity scores for individual cells were computed using the “AUCell” R package (version 1.20.2), utilizing lactylation-related genes (LRGs) identified in prior studies (**Supplementary Table 2**) (25). Cells were categorized into high and low AUC groups based on the median AUC score. For further analysis, 9,288 differentially expressed genes (DEGs) were identified between the two groups, with the screening criteria set as |log_2_ fold change| > 1 and P value < 0.05 (**Supplementary Table 3**). To identify prognostic-relevant genes, we performed univariable Cox regression analysis on the TCGA-BLCA cohort from UCSC Xena database (https://xenabrowser.net/datapages/) for each DEGs. Genes with a P value < 0.05 were considered statistically significant prognostic genes. Finally, 1,542 lactylation-related survival genes (LRSGs) were identified, which were considered to have the most effects on survival and lactylation activity (**Supplementary Table 4**).

### 2.4 Bulk RNA-seq data for BC

The RNA-seq data and clinical information for the TCGA-BLCA cohort were obtained from the UCSC Xena database (https://xena.ucsc.edu/). This cohort comprised 400 primary bladder urothelial carcinoma samples. Sequencing was performed on the Illumina HiSeq 2000 platform, and raw counts were normalized using the RSEM method. Log_2_ transformation with a pseudo-count of 1 was applied, followed by normalization across samples. Two independent external cohorts were used for validation. The GSE13507 dataset was downloaded from the GEO database, which included 165 BC samples. The E-MTAB-4321 dataset was retrieved from the Array Express database, which contained 460 BC samples. The details of BC cohorts are provided in **Supplementary Table 1**.

### 2.5 The effect on survival of LRSGs

The z-score of LRSGs was calculated to evaluate the effect on survival. The z-score model was determined using the following equation.

The z-score for patient j at gene i was calculated using the formula:


$${\text{Z}}_{\text{ij }}=\frac{{\text{X}}_{\text{ij}}-{\text{μ}}_{\text{i}}}{{\text{σ}}_{\text{i}}}$$

Xij denotes the expression value of gene i in patient j, with μi as the gene’s mean expression across all patients and σi as the standard deviation among them.

Patient j’s z-scores on all genes were averaged to obtain the total z-score:


$${\text{Z}}_{\text{total},\text{j}}=\frac{1}{1542}\sum_{\text{i}=1}^{1542}{\text{Z}}_{\text{ij}}$$

Z_total,j_ represents the total z-score of patient j, Z_ij_ is the z-score of patient j on the i gene.

### 2.6 Cell culture and treatment

The study employed five BC cell lines (T24, 5637, J82, RT-112, and UM-UC-3) alongside the human normal bladder epithelial cell line SV-HUC. SV-HUC (CP-H166, Procell, Wuhan, China), T24 (SNL-038, Sunncell, Wuhan, China), 5637 (CL-0002, Procell, Wuhan, China), J82 (CL-0125, Procell, Wuhan, China), RT-112 (CL-0682, Procell, Wuhan, China), and UM-UC-3 (CC1003, CELLCOOK, Guangzhou, China) cells were cultured in Roswell Park Memorial Institute medium 1640 (RPMI-640) or Dulbecco ‘s Modified Eagle’s Medium (DMEM), supplemented with 10% fetal bovine serum (FBS) (Suzhou Shuangru Biotechnology Co., Ltd.) and 1% penicillin-streptomycin (HY-K1006, MCE, China). Cells were incubated at 37 °C in a 5% CO2 humidified atmosphere. The study examined lactate production, histone lactylation, and the expression levels of HK2, PFKM, PDK4, LDHA, PanKla, and H3K18la in SV-HUC and five BC cell lines.

5637 and T24 cells were used for subsequent experiments. Cells were exposed to glycolysis inhibitors, sodium dichloroacetate (DCA, 0–15 mmol/L) (HY-Y0445A, MCE, China) and 2-deoxy-D-glucose (2-DG, 0–10 mmol/L) (HY-13966, MCE, China) or the vehicle control (phosphate buffer saline).The cells were transfected with either LDHA siRNA or scramble siRNA as a negative control, then treated with 10 mmol/L sodium lactate (NaLa, HY-B2227B, MCE, China).

Protein levels were assessed using Western blot analysis. Colony formation assays were used to evaluate cell proliferation following 1–2 weeks of treatment. Cell migration was assessed using wound healing and transwell assays after a 48-hour intervention.

### 2.7 Protein extraction and Western blotting

Proteins were extracted from mouse or human bladder tissue using RIPA lysis buffer (HY-K1001, MCE, China) with 1% protease inhibitor cocktail (HY-K0010, MCE, China) and PMSF (HY-B0496, MCE, China). Protein concentration was measured using the GenStar Protein Assay Kit (E162-01, Beijing, China). Proteins were separated using an SDS-PAGE Gel kit (EC1022-B, Shandong Sparkjade Biotechnology Co., Ltd., China) with a Color Prestained Protein Marker (SW177, Seven, Beijing, China) and subsequently transferred to PVDF membranes (Millipore, Burlington, MA, USA). The membranes were incubated overnight at 4 °C with primary antibodies, each diluted 1:1000, targeting HK2 (R24552, Zenbio, China), PFKM (ET7106-97, HUABIO, China), PDK4 (860703, Zenbio, China), PanKla (PTM-1401RM, Jingjie PTM BioLab Co. Ltd), H3K18la (PTM-1427RM, Jingjie PTM BioLab Co. Ltd), H3K9la (1:1000; PTM-1419RM, Jingjie PTM BioLab Co. Ltd), H4K12la (1:1000; PTM-1411RM, Jingjie PTM BioLab Co. Ltd), AHNAK2 (HA721622, HUABIO, China), PVR (R27188, Zenbio, China), SLC7A11 (HA600098, HUABIO, China), and SREBF1 (HA722160, HUABIO, China). After washing, membranes were incubated with matching secondary antibodies diluted 1:20000 for 1 hour at room temperature. The protein bands were visualized using the Super ECL Plus Kit (S6009L, US EVERBRIGHT, Suzhou, China). For the proteins HK2, PFKM, PDK4, AHNAK2, PVR, SLC7A11, and SREBF1, the reference protein was GAPDH (CSB-MA000071M1m, CUSABIO, https://www.cusabio.com/). The reference protein for the PanKla and H3K18la proteins was H3 (1:2000; PTM-1001RM, Jingjie PTM BioLab Co. Ltd). Target protein expression levels were measured using Image Lab software (version 3.0, Bio-Rad, USA).

### 2.8 Immunofluorescence

IF was conducted on 4 μm-thick bladder sections from mice or human using an IF kit (abs996, Absin, China). Briefly, xylene and ethanol were used to deparaffinize the slides, and then citrate solution was utilized for retrieving the antigen. Following an hour of blocking, primary antibodies against PFKM (1:200; ET7106-97, HUABIO, China), LDHA (1:200; R24822, Zenbio, China), H3K18la (1:200; PTM-1427RM, Jingjie PTM BioLab Co. Ltd), and Ki-67 (1:200; HY-P81234, MCE, China) were incubated on the slides overnight at 4 °C. The 4’,6-diamidino-2-phenylindole served as a chromogen.

### 2.9 Immunohistochemistry

IHC was conducted on 4 μm-thick bladder sections from both healthy individuals and BC patients using an IHC kit (abs996, Absin, China). The slides underwent deparaffinization with xylene and ethanol, followed by antigen retrieval using citrate solution. Following an hour of blocking, primary antibodies against AHNAK2 (1:200; HA721622, HUABIO, China), PVR (1:200; R27188, Zenbio, China), SLC7A11 (1:200; HA600098, HUABIO, China), and SREBF1 (1:200; HA722160, HUABIO, China) were incubated on the slides overnight at 4 °C. 3,3’-Diaminobenzidine was used as a chromogen.

### 2.10 Lactate content measurement

After centrifuging the homogenized tumor tissue or cell culture at 12,000 g for 10 minutes, the supernatant was collected for further analysis. Lactate levels were measured using the lactic acid content assay kit (BC2235, Solarbio, China) following the manufacturer’s protocol. Absorbance values at 570 nm were measured using a spectrophotometer. Based on the cell number, the lactate content was standardized.

### 2.11 RNA interference and lentivirus transfection

50 mmol/L was the working concentration at which siRNA was dissolved. Using Lip3000 (L3000015, Invitrogen, USA), 5637 and T24 cells underwent RNA interference. Cells were harvested after transfection for 48 hours. Recombinant lentiviruses expressing si-LDHA were prepared by Beijing Tsingke Biotech Co., Ltd. to achieve stable LDHA knockdown expression in the cells. Subsequent investigations were conducted using the si-LDHA stable cell line. The sequence of si-LDHA was 5’- GCTACACATCCTGGGCTAT-3’.

### 2.12 CCK-8 assay

Cell viability was evaluated using the Cell Counting Kit-8 (K1018, APExBIO, Houston, USA). A total of 1 x 10^4 pretreated cells were seeded into 96-well plates, with each well receiving 100 µL of medium. CCK8 solution (10 µL) was introduced into each well the following day, and incubated cells for 2 h at 37 °C. The absorbance was recorded at a wavelength of 450 nm. Relative cell viability was calculated after subtracting the values of the blank control wells.

### 2.13 Colony formation and wound healing assay

5637 and T24 cells were plated at 1000 cells per well in 6-well plates, with regular medium changes. After 7–14 days of culture, formaldehyde fixation and crystal violet (HY-B0324A, MCE, China) staining were performed, and finally, photographs were taken to count colonies.

When cells were fully grown in 6-well plates, wounds were made by scratching with a 200 µL sterile pipette tip for the purpose of wound healing tests. After washing, the cells were cultured for 48 hours, and wound areas in images taken at 0, 24, and 48 hours were quantified using ImageJ software.

### 2.14 Transwell migration assay

Through a trans well chamber with inserts (Corning Life Science, NY, USA), the migration ability of 5637 and T24 cells was assessed. The lower chamber was filled with 600 µL of medium containing 10% FBS as an attractant, while 1 × 10^5 cells in 200 µL of medium were placed in the upper chamber. Cells that migrated to the lower chamber after 48 hours were fixed, stained, and quantitatively analyzed using ImageJ software.

### 2.15 Tumorigenesis model

Animal experiments received approval from the Ethics Committee of the First Affiliated Hospital of Zhengzhou University (2024-KY-1687) and adhered to ARRIVE 2.0 guidelines. A total of 20 four-week-old nude mice were used in this study, which randomly included both female and male. The mice were randomly allocated into 4 experimental groups (control groups vs. treatment groups), with n = 5 mice per group. The subjects were kept in a controlled, specific pathogen-free (SPF) environment with a 12-hour light/dark cycle, a temperature of 22 ± 2 °C, and 50% ± 10% relative humidity, having unrestricted access to autoclaved food and water.

To establish the xenograft model, T24 cells (5 × 106 in 8 μL of sterile phosphate-buffered saline) were subcutaneously injected into 4-week-old nude mice. Tumor growth was monitored weekly for four weeks. The termination criteria for tumor burden in the experimental mice were set as follows: when the tumor diameter reached 20mm, or when the mice experienced any signs of severe distress (>20% body weight loss, ulceration, or lethargy). After the 28-day experiment, all mice were humanely euthanized using CO2 inhalation. Tumors were excised and weighed for further analysis. Tumor volume was determined using the formula: Volume = (Length × Width²)/2.

### 2.16 CUT&Tag

The CUT&Tag assay was performed by Wuhan IGENEBOOK Biotechnology Co. Ltd. Briefly, the key steps involved harvesting and preparing 2 × 106 cells, binding them to concanavalin A-coated magnetic beads, incubating with H3K18la antibody, performing tagmentation using pG-Tn5, followed by DNA purification. The DNA library was amplified and sequenced via the Illumina NovaSeq platform. The experiment was conducted with three biological replicates and the Pearson correlation coefficient of each replicate sample was >0.9. DiffBind identified differential peaks (DP) using thresholds of a discovery rate (FDR) < 0.05 and |log2Fold Change| > 0.58. At the same time, the MACS2 software was combined for peak calling, with a q value <0.05 set as the significance threshold. ChIP seeker was employed to annotate these peaks to promoter regions defined as the upstream and downstream ±3 kb of the transcriptional start site and to identify the associated genes (**Supplementary Table 5**).

### 2.17 Differential analysis of BC cell lines

RNA-seq data for the BC cell line was sourced from the GEO database (GSE231383). Genes exhibiting altered expression between tumor and normal cells were identified through differential expression analysis. A total of 563 upregulated genes were identified in tumor cells (**Supplementary Table 6**). By intersecting these with the genes identified from CUT&Tag analysis, 39 genes were obtained and regarded as potentially malignant contributors to BC progression (**Supplementary Table 7**). The Kyoto Encyclopedia of Genes and Genomes (KEGG) analysis was performed to elucidate the functional roles of these genes.

### 2.18 Chip-qPCR assay

T24 cells were subjected to formaldehyde crosslinking, quenched with glycine, and thoroughly washed. After cell lysis and sonication to obtain 200–500 bp DNA fragments, the samples underwent immunoprecipitation with anti-H3K18la (PTM-1427RM, Jingjie PTM BioLab Co. Ltd.) and anti-IgG antibodies. Promoter-associated DNA fragments were subsequently quantified using qPCR.

### 2.19 Machine learning screening for core genes

To pinpoint crucial genes in BC progression, random forest (RF) and LASSO logistic regression analyses were applied to 39 candidate malignant genes. The “random Forest” R package (version 4.7-1.1) was used to build the RF model (26, 27). The number of decision trees was set to 500, and the number of randomly selected predictor variables at each split was the square root of the total number of variables to control the risk of overfitting. The top 4 genes with the highest average decrease in Gini coefficient were selected as candidate genes. To minimize potential bias, the model was internally validated using five-fold cross-validation to ensure the stability of variable selection. The LASSO regression analysis was performed using the “glmnet” R package (version 4.1-8). Ten-fold cross-validation was employed to determine λ.min and λ.1se for constructing a parsimonious model. 1000 random seeds were set to ensure the stability of the analysis. Genes identified through the intersection of the two algorithms were considered potential core genes. The Gene Set Enrichment Analysis (GSEA) for four core genes was performed using the “GSEA Base” R package (version 1.60.0). CIBERSORT and correlation analysis were utilized to examine the association between four core genes and 22 immune cells.

### 2.20 Construction and validation of risk signature

We developed a risk score model using coefficients from the LASSO analysis, as each of the four hub genes significantly influenced BC patient prognosis. The risk score model was calculated using the following formula:

Risk score = (0.07565607) × AHNAK2 + (0.11387579) × PVR + (0.01857407) × SLC7A11 + (0.18961588) × SREBF1.

A survival analysis was performed on TCGA-BLCA patients, categorizing them into high- and low-risk groups according to the median risk score. Group differences were visualized and assessed for statistical significance through log-rank testing. Additionally, the stability of the risk score was verified through GSE13507 and E-MTAB-4321 datasets.

### 2.21 Statistical analysis

R 4.2.2 software was used to perform data processing, cleaning, statistical analysis, and result plotting for this study. Statistical tests were selected according to data type: Wilcox/t-tests for group comparisons and Fisher’s exact/chi-square tests for categorical variables. At least three independent biological replicates were conducted for each experiment. All image acquisition and subsequent quantitative analysis were independently conducted by two researchers who were unaware of the experimental groupings. *p < 0.05, **p < 0.01, and ***p < 0.001.

## 3 Results

### 3.1 Glycolysis levels are elevated in the single-cell transcriptome landscape of BC patients

**Figure 2:**
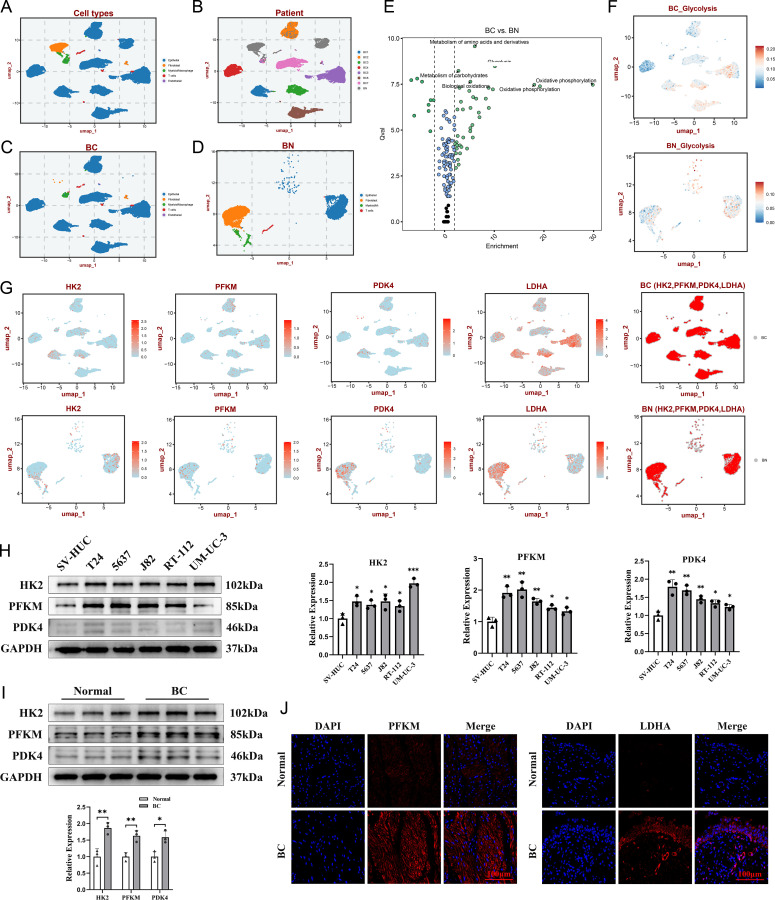
Glycolysis activation in bladder cancer. **(A)** The UMAP graph of 42–440 cells, colored according to the corresponding cell types. **(B)** The samples’ UMAP graphic, color-coded by the samples, displays the source of the samples. **(C)** The UMAP graph of 36–407 tumor cells from seven tumor samples. **(D)** The 6–033 tumor cells’ UMAP graphic from 1 normal sample, color-coded by the associated cell types. **(E)** Volcano plot of SCPA-derived pathway enrichment (mean pathway change from BC vs. BN sample comparison). Black points = non-significant pathways; blue points = significant pathways without enrichment; green points = significant and enriched pathways. **(F)** The UMAP graphic from Glycolysis pathway scores for BC and BN samples. Data are colored according to expression levels. **(G)** Canonical marker genes (e.g., HK2, PFKM, PDK4, and LDHA) of the Glycolysis pathway for BC and BN samples, respectively. Data are colored according to expression levels. **(H)** Western blotting for HK2, PFKM, and PDK4 in five distinct BC cell lines: T24, 5637, J82, RT-112, UM-UC-3. The glyceraldehyde phosphate dehydrogenase (GAPDH) was used as the reference protein. The bar graphs show the relative expression of HK2, PFKM, and PDK4 protein (n = 3). Each BC cell line was compared pairwise with SV-HUC. All data are presented as mean ± SD. Statistical analysis was performed using the paired sample two-tailed t-test (*p<0.05; **p<0.01; ***p<0.001; ns, not significant). The scattered points represent the distribution of the original data and reflect the variability of the data. **(I)** Western blotting for HK2, PFKM, and PDK4 proteins in normal bladder tissues and BC tissues. The glyceraldehyde phosphate dehydrogenase (GAPDH) was used as the reference protein. The bar graphs show the relative expression of HK2, PFKM, and PDK4 proteins (n = 3). Statistical analysis was performed using the paired sample two-tailed t-test (*p<0.05; **p<0.01; ***p<0.001; ns, not significant). The scattered points represent the distribution of the original data and reflect the variability of the data. **(J)** Immunofluorescence for PFKM and LDHA in normal bladder tissues and BC tissues (n = 3). The proteins’ expression is showed in red.

Primary diagnostic specimens were obtained from seven patients diagnosed with BC and one normal bladder sample (BN) for scRNA-seq. Initially, poor-quality cells were filtered out, finally yielding 42,440 cells. Using the UMAP algorithm, the cells were clustered into 31 distinct groups (**Supplementary Figure 1A**). Violin plots were created to depict the expression levels of key marker genes across various clusters (**Supplementary Figure 1D**). Analysis of signature gene expression in each cluster, alongside validated cell-type markers from prior research, identified five primary cell types: T cells, endothelial cells, myeloid/macrophages, fibroblasts, and epithelial cells (**Figure 2A**) (16, 28–30). As displayed in **Figure 2B**, significant inter-patient heterogeneity was observed among patients with BC. **Figure 2C, D** illustrate the distribution of the five cell species within BC and BN samples, respectively. As shown in Cell Cycle analysis, the predominance of epithelial cells in the S phase indicates their crucial involvement in promoting malignant processes during BC progression (**Supplementary Figure 1B**). Intercellular communication was predicted by constructing cell-cell communication networks using specific pathways and ligand-receptor interactions. Cellular interactions between fibroblasts, T cells, endothelial cells, and epithelial cells were commonly observed, as presented in the heatmap illustrating the number of ligand-receptor pairings (**Supplementary Figure 1C**). Notably, frequent and intense interactions were observed between fibroblast and epithelial cells, endothelial and epithelial cells, fibroblast and T cells, and fibroblast cells with myeloid/macrophages. The infrequent interactions between T-cells and other cell types may contribute to the inadequate immunotherapy response seen in BC patients.

To investigate the key mechanisms driving BC, we performed single-cell metabolic enrichment analysis (**Figure 2E**). BC samples showed notable enrichment in pathways involving amino acid and derivative metabolism, glycolysis, carbohydrate metabolism, and both glycosaminoglycan metabolism and related diseases, compared to BN samples. The results suggest that metabolic activation and disorders are pivotal in the progression of BC. It is well-established that enhanced glycolysis is a common feature in various cancers (31). The single-cell analysis revealed a marked elevation in glycolysis in BC samples relative to BN samples (**Figure 2F**). Additionally, glycolysis-associated signature genes (HK2, PFKM, PDK4, and LDHA) were significantly upregulated in BC samples (**Figure 2G**). Moreover, by comparing the level of glycolysis signature proteins between five BC cell lines and the normal bladder cell line, it was observed a higher expression level of HK2, PFKM, and PDK4 (**Figure 2H**) (32). Consistently, HK2, PFKM, and PDK4 levels were also elevated in the BC tissues compared to the surrounding noncancerous bladder tissues (**Figure 2I**). Immunofluorescence (IF) staining further revealed increased expression of PFKM and LDHA in tumor tissues (**Figure 2J**). In conclusion, scRNA-seq analysis revealed that metabolic activation and disruption, especially the upregulation of glycolysis, are pivotal in BC progression.

### 3.2 Glycolysis-induced histone lactylation affects the prognosis of BC patients

**Figure 3:**
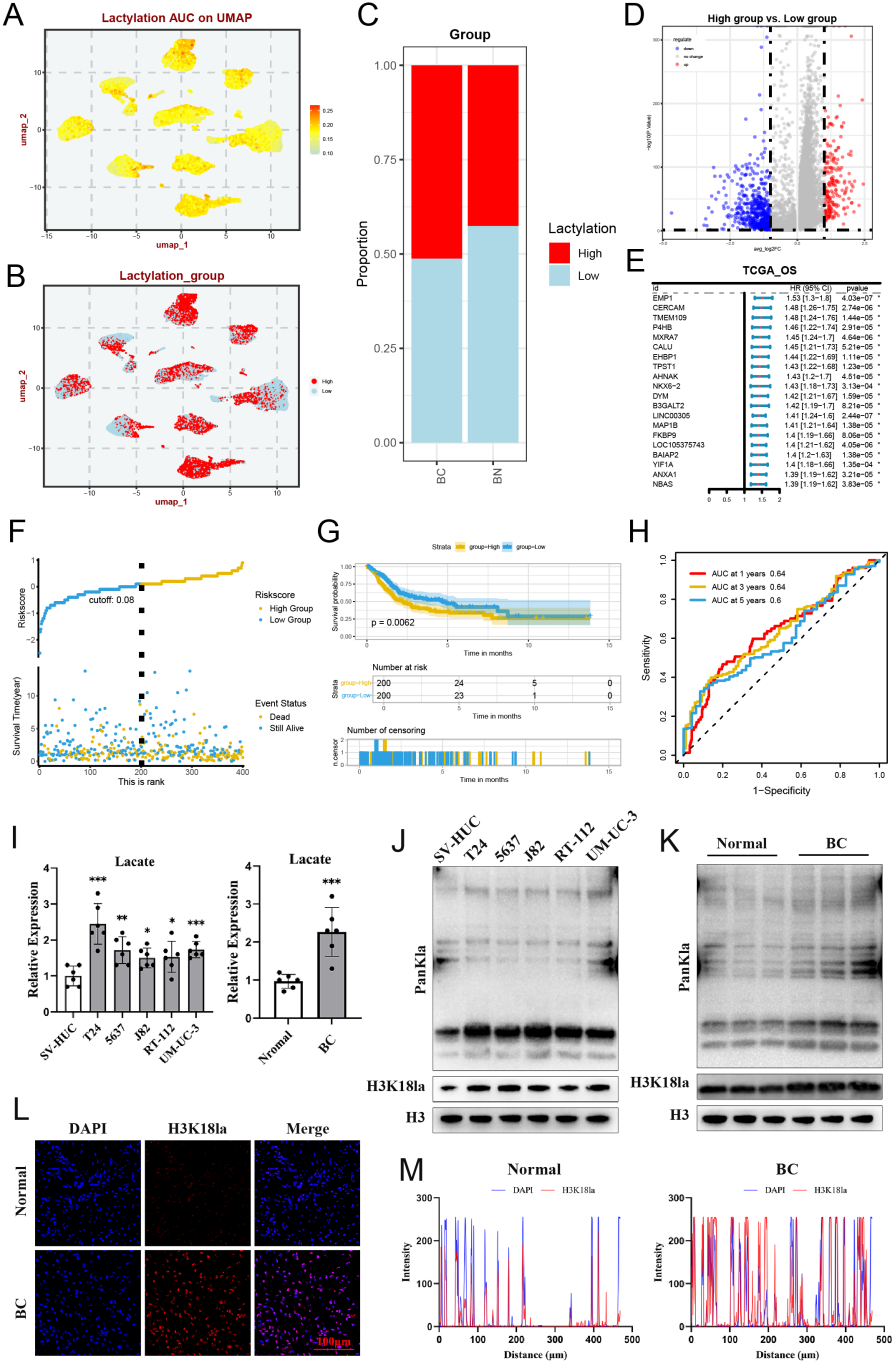
Histone lactylation in bladder cancer contributes to poor prognosis. **(A)** Visualization of AUCell scores, reflecting lactylation levels in single cells from scRNA-seq data of BC samples. **(B)** AUCell groups are projected, with high lactylation marked in red and low lactylation in blue. **(C)** The proportional plotting graph of the AUCell groups for BC and BN. **(D)** Volcano plot showing differential genes in the high lactylation group relative to the low lactylation group. A significance measure is depicted on the Y-axis (-log10(P.Value)) and effect size is depicted on the X-axis (logFC). Downregulated genes (logFC ≤ -1) are shown in blue, upregulated genes (logFC ≥ 1) in red. **(E)** Univariate Cox regression on differentially expressed genes within the TCGA-BLCA cohort. **(F)** Lactylation score of each patient in the TCGA-BLCA datasets, with patients ranked by score. **(G)** Kaplan-Meier analysis of high- and low-lactylation groups in TCGA-BLCA datasets. **(H)** Lactylation score-based time-dependent ROC analysis for prognosis prediction in TCGA-BLCA data. **(I)** Changes in lactate levels in five BC cell lines (left, n = 6 independent cultures) and paired BC tissues and adjacent normal tissues (right, n = 6 samples). Each BC cell line was compared pairwise with SV-HUC. All data are presented as mean ± SD. Statistical analysis was performed using the paired sample two-tailed t-test (*p<0.05; **p<0.01; ***p<0.001; ns, not significant). The scattered points represent the distribution of the original data and reflect the variability of the data. **(J)** Changes in PanKla (Histone pan lysine lactylation) and H3K18la levels in five BC cell lines. **(K)** Changes in PanKla **(Histone pan lysine lactylation)** and H3K18la levels in paired BC tissues compared to adjacent normal bladder tissues. **(L)** Immunofluorescence of H3K18la proteins **(red)** in normal bladder tissues and paired BC tissues. Nuclei were counterstained with DAPI (blue). **(M)** Normalized read densities for H3K18la at the normal bladder tissues and paired BC tissues.

We investigated lactylation’s role in BC by applying AUC scores to single-cell LRG profiles, categorizing cells into high- and low-lactylation groups based on median AUC scores to understand its impact on key metabolic pathways (**Figures 3A, B**). BC samples showed a greater proportion of cells in the high-lactylation group compared to BN (**Figure 3C**). A total of 9,288 differentially expressed genes (DEGs) were identified between the two groups (**Figure 3D**; **Supplementary Table 3**). Univariable Cox regression analysis identified 1542 prognostically significant genes from DEGs in the TCGA-BLCA cohort as LRSGs (**Supplementary Table 4**). **Figure 3E** displays the top 20 ranked LRSGs. A z-score (lactylation score) was calculated for each patient from the TCGA-BLCA datasets based on the LRSGs. Patients were categorized into high- and low-lactylation groups using the median z-score to assess the predictive capability of LRSGs (**Figure 3F**). A notable disparity in overall survival (OS) was observed between the two groups, indicating that increased lactylation correlates with reduced survival (**Figure 3G**). ROC analysis demonstrated the lactylation signature’s clear prognostic value for TCGA-BLCA cohort patients, with AUC values of 0.64 at 1 and 3 years, and 0.60 at 5 years (**Figure 3H**). These findings suggest that elevated lactylation levels are associated with poor survival outcomes in patients with BC.

To validate our findings, lactate levels were determined in BC cell lines, including T24, 5637, J82, RT-112, and UM-UC-3. The results demonstrated that compared to the normal bladder cell line, lactate production was higher in these BC cell lines (**Figure 3I**). Similarly, the lactate content was found to be higher in BC tissues (**Figure 3I**). Additionally, BC cell lines exhibited elevated levels of global/pan-lysine lactylation (PanKla) (**Figure 3J**). Given the recent discovery of H3K18la and a novel histone modification, our investigation focused on understanding its expression and function in BC progression (10). H3K18la levels were observed to be elevated in BC cell lines (**Figure 3J**). Moreover, compared to adjacent non-tumor tissues, both PanKla and H3K18la were significantly upregulated in BC tissues, but the expression of H3K9la and H4K12la did not change significantly (**Figure 3K**; **Supplementary Figure 1L**). IF staining of H3K18la also showed similar results (**Figures 3L, M**).

### 3.3 Inhibiting histone lactylation reduces the proliferation and migration capabilities of BC cells

**Figure 4:**
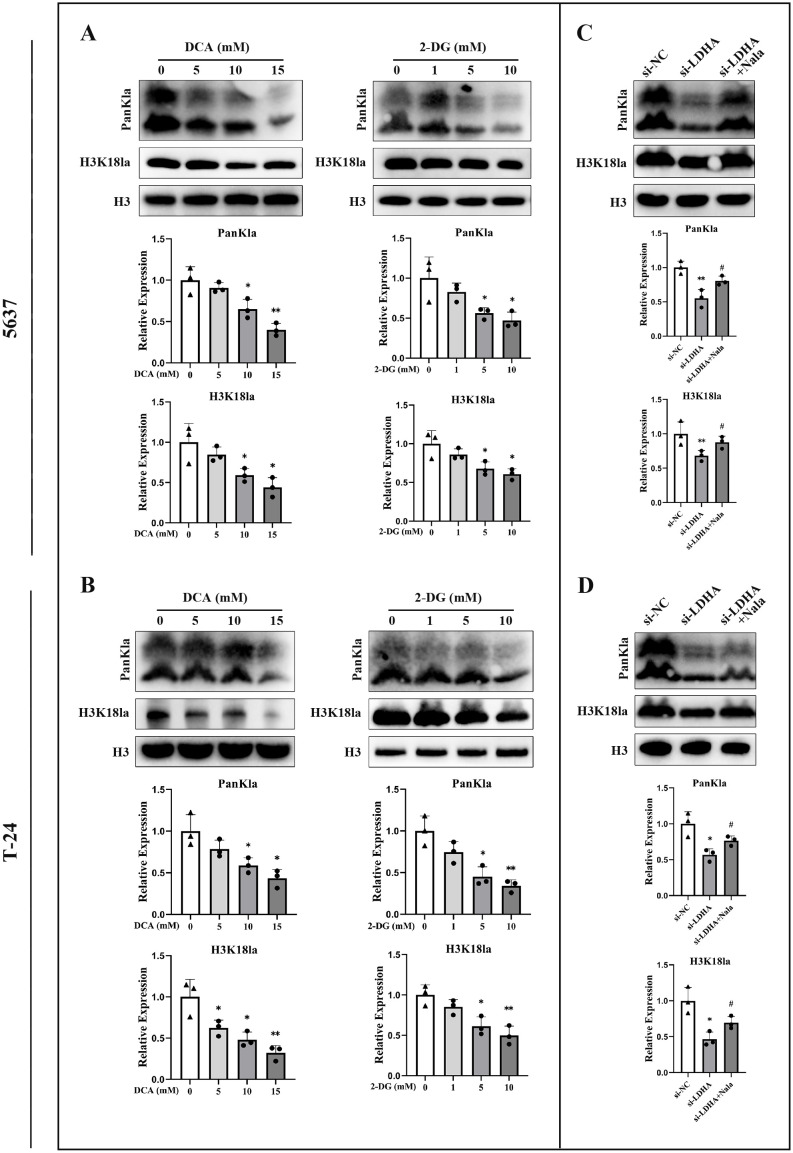
Glycolysis inhibition in BC cell lines reduces histone lactylation. Two BC cell lines 5637 and T-24 were treated with glycolysis inhibitors DCA (0–15 mmol/L) or 2-DG (0–10 mmol/L) for 24 h, or transfection with LDHA siRNA (si-LDHA) or negative control siRNA (si-NC) for 48 h with or without sodium lactate (NaLa, 10 mmol/L) treatment. **(A-D)** Pan-lysine lactylation (PanKla) and H3K18 lactylation (H3K18la) levels were assessed via western blot, with quantification using Image J. Triplicate independent biological replicates were done per group, with data shown as mean ± SD. Statistical analysis employed paired two-sided t-tests *p<0.05; **p<0.01; ns, not significant. Scattered points indicate original data distribution and reflect data variability.

To elucidate glycolysis’ effect on histone lactylation, we employed two glycolysis inhibitors (DCA, 2-DG) and LDHA knockdown, which were respectively tested in 5637 and T-24 cells. PanKla and H3K18la levels showed a consistent decrease as inhibitor dosages increased (**Figures 4A, B**). Moreover, si-LDHA treatment resulted in a significant reduction in histone lactylation levels, while Nala intervention effectively reversed this effect (**Figures 4C, D**).

**Figure 5:**
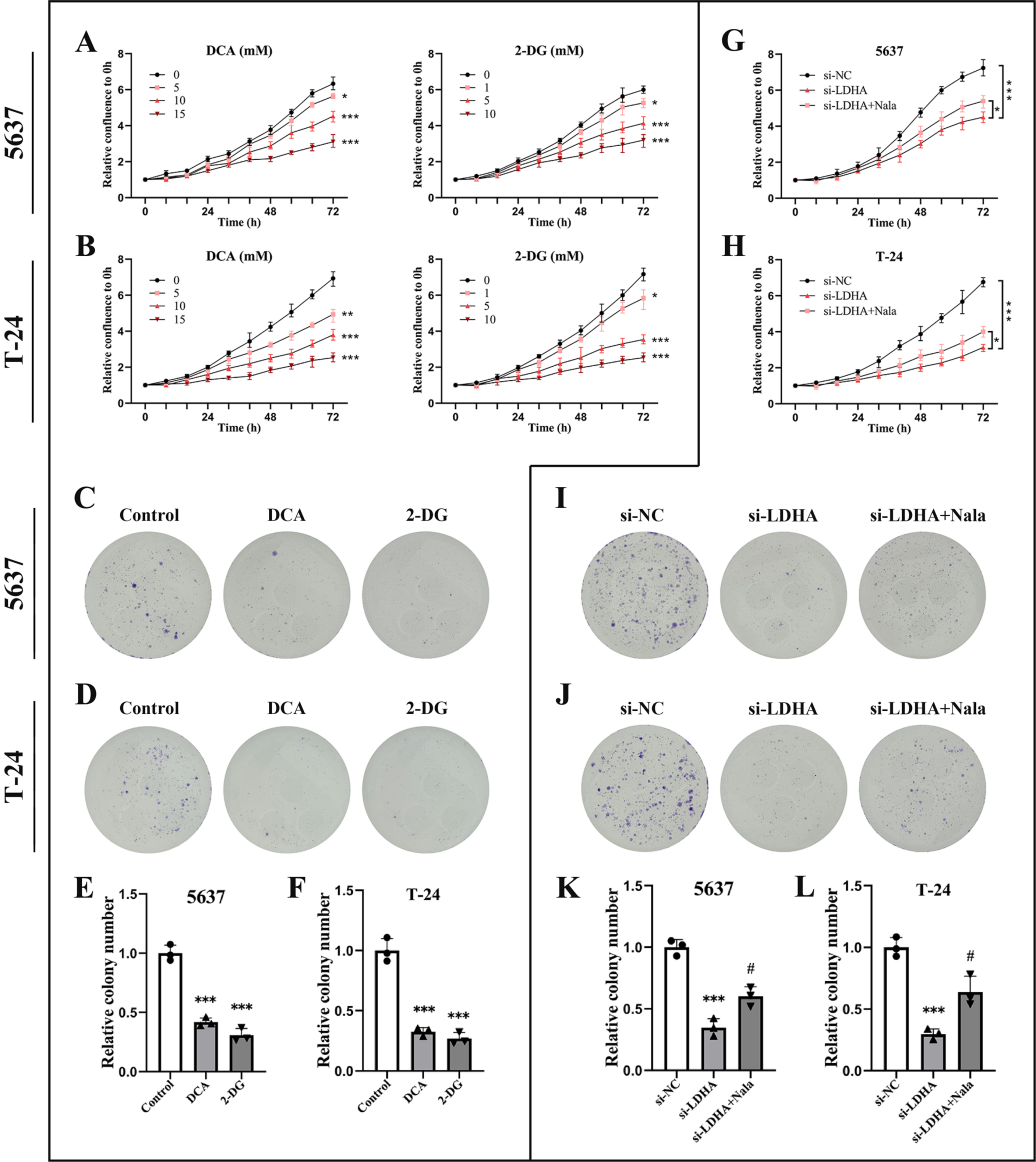
Glycolysis inhibition suppresses BC cell proliferation. Two BC cell lines (5637 and T-24) were treated with glycolysis inhibitors DCA (0–15 mmol/L) or 2-DG (0–10 mmol/L), or transfected with LDHA siRNA (si-LDHA) or negative control siRNA (si-NC), with or without 10 mmol/L sodium lactate (NaLa). Cell viability was visualized via IncuCyte S3 in 5637 **(A, G)** and T-24 **(B, H)** cells. Colony formation assays assessed cell proliferation in 5637 **(C, E, I, K)** and T-24 **(D, F, J, L)** cells. Triplicate independent biological replicates were done per group, with data shown as mean ± SD. Statistical analysis used paired two-sided t-tests (*p<0.05; **p<0.01; ***p<0.001; ns, not significant). Scattered points indicate original data distribution and variability.

Subsequently, we investigated the changes in cell proliferation. Treatment with DCA or 2-DG significantly decreased cell viability and colony formation ability (**Figures 5A–F**). LDHA silencing significantly suppressed cell proliferation and colony formation in 5637 and T-24 cells; however, treatment with Nala mitigated the anti-proliferative effects of LDHA silencing (**Figures 5G–L**).

**Figure 6:**
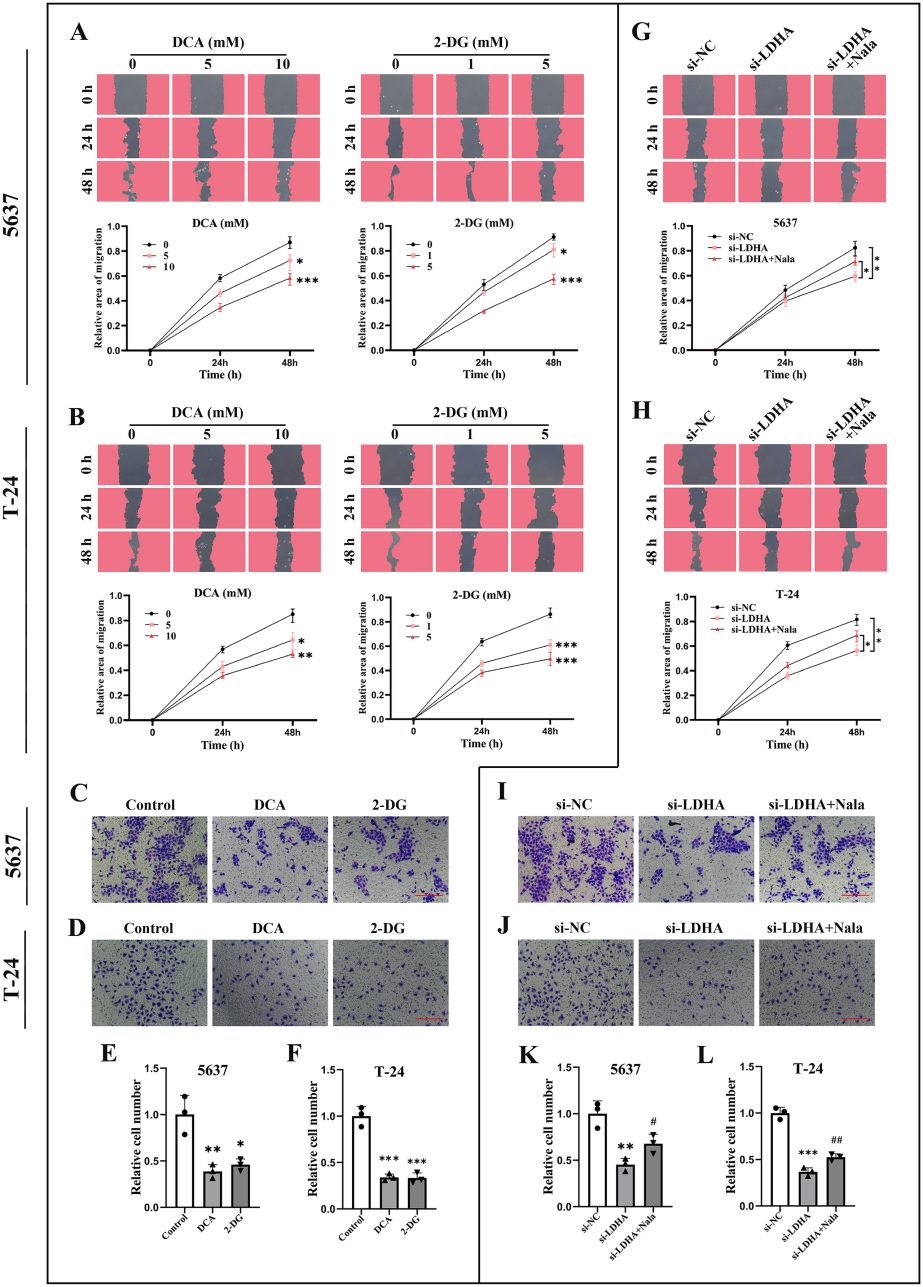
Glycolysis inhibition reduces histone lactylation and suppresses BC cell migration. **(A-F)** Two BC cell lines (5637 and T-24) were treated with glycolysis inhibitors DCA (0–10 mmol/L) or 2-DG (0–5 mmol/L) for 48 h; migration ability was assessed via wound healing and transwell assays. **(G-L)** 5637 and T-24 cells were transfected with LDHA siRNA (si-LDHA) or negative control siRNA (si-NC) for 48 h, with or without 10 mmol/L sodium lactate (NaLa) treatment; migration was evaluated using wound healing and transwell assays. Triplicate independent biological replicates were done per group, with data shown as mean ± SD. Statistical analysis utilized paired two-sided t-tests (*p<0.05; **p<0.01; ***p<0.001; ns, not significant). Scattered points indicate original data distribution and variability.

We performed wound healing and transwell assays to assess the effect of lactylation on BC cell migration. DCA and 2-DG treatments reduced migration of 5637 and T-24 cells, with higher concentrations demonstrating more pronounced inhibitory effects (**Figures 6A, B**). Consistently, LDHA knockdown diminished migration capabilities, whereas the inhibitory effect of si-LDHA was lessened by Nala intervention (**Figures 6G, H**). Transwell assays confirmed these findings, demonstrating that DCA and 2-DG treatments substantially decreased cell migration (**Figures 6C–F**). Notably, LDHA knockdown had a similar inhibitory effect on migration, which was countered by Nala in 5637 and T-24 cells (**Figures 6I–L**). These collective results indicated that histone lactylation played a critical role in the BC progression.

### 3.4 Inhibition of histone lactylation prevents BC tumor progression in the xenograft mouse model

**Figure 7:**
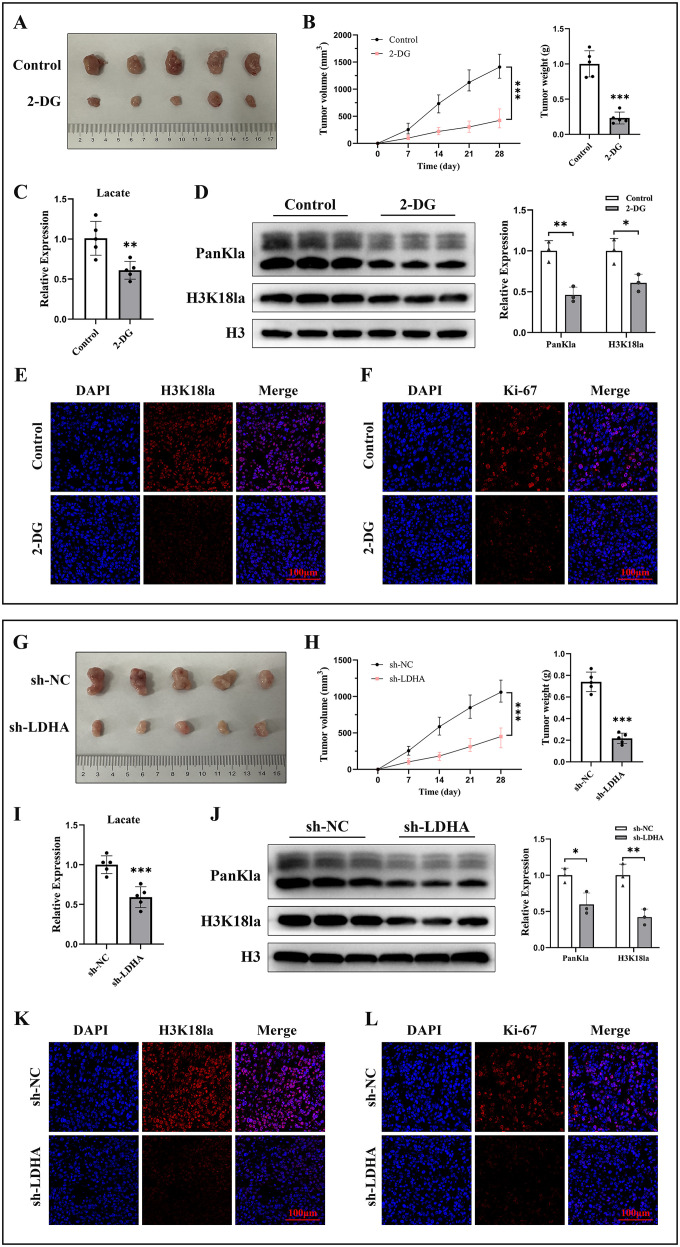
Glycolysis inhibition suppresses cancer progression. **(A-F)** T-24 cells were subcutaneously injected into nude mice, which were then intraperitoneally administered 2-DG (750 mg/kg daily) or vehicle for 30 days. **(G-L)** Stable LDHA knockdown (sh-LDHA) was achieved via lentivirus in T-24 cells; sh-LDHA and control (sh-NC) cells were subcutaneously injected into nude mice. Tumor gross images **(A, G)** (n=5), and tumor volume and weight after sacrifice **(B, H)** (n=5) are shown. Tumor lactate content **(C, I)** (n=5) was measured. PanKla and H3K18la levels were assessed by western blot or IF staining, with quantification via Image J **(D, J)** (n=3). IF staining (red) shows H3K18la expression **(E, K)** (n=3). Cell proliferation was evaluated by IF staining for Ki-67 **(F, L)** (n=3). Data are presented as mean ± SD, with statistical analysis using paired two-sided t-tests (*p<0.05; **p<0.01; ***p<0.001; ns, not significant). Scattered points indicate original data distribution and variability.

To assess the biological role of histone lactylation in BC progression, T24 cells were injected subcutaneously into nude mice to create a xenograft model. Mice were then treated with either 2-DG or a vehicle control. It was found that tumor volume measurements taken every seven days demonstrated a significant reduction, and tumor weight was also lower in the 2-DG group (**Figures 7A, B**). Furthermore, LDHA knockdown had a similar impact on tumor growth. Subcutaneous injection of stable sh-LDHA and control (sh-NC) T24 cells into nude mice resulted in a significant reduction in tumor size and weight in the sh-LDHA group (**Figures 7G, H**).

Next, the xenograft model was used to investigate the effect of glycolysis inhibition on lactate production and histone lactylation. Both 2-DG treatment and LDHA knockdown led to a decrease in lactate production (**Figures 7C, I**). Moreover, we observed reduced levels of PanKla and H3K18la in 2-DG-treated tumors (**Figures 7D, E**), with similar reductions noted in the LDHA inhibition groups (**Figures 7J, K**). Proliferation was assessed using Ki-67 staining, revealing diminished expression in 2-DG and sh-LDHA-treated groups (**Figures 7F, L**). Collectively, these findings demonstrate that inhibiting glycolysis, either through 2-DG treatment or LDHA knockdown, decreases tumor growth, restrains lactate production and histone lactylation, and diminishes proliferation. These results suggest a promising approach for impeding BC progression.

### 3.5 The activation of AHNAK2, PVR, SLC7A11, and SREBF1 transcription positively correlates with H3K18la-mediated BC progression

**Figure 8:**
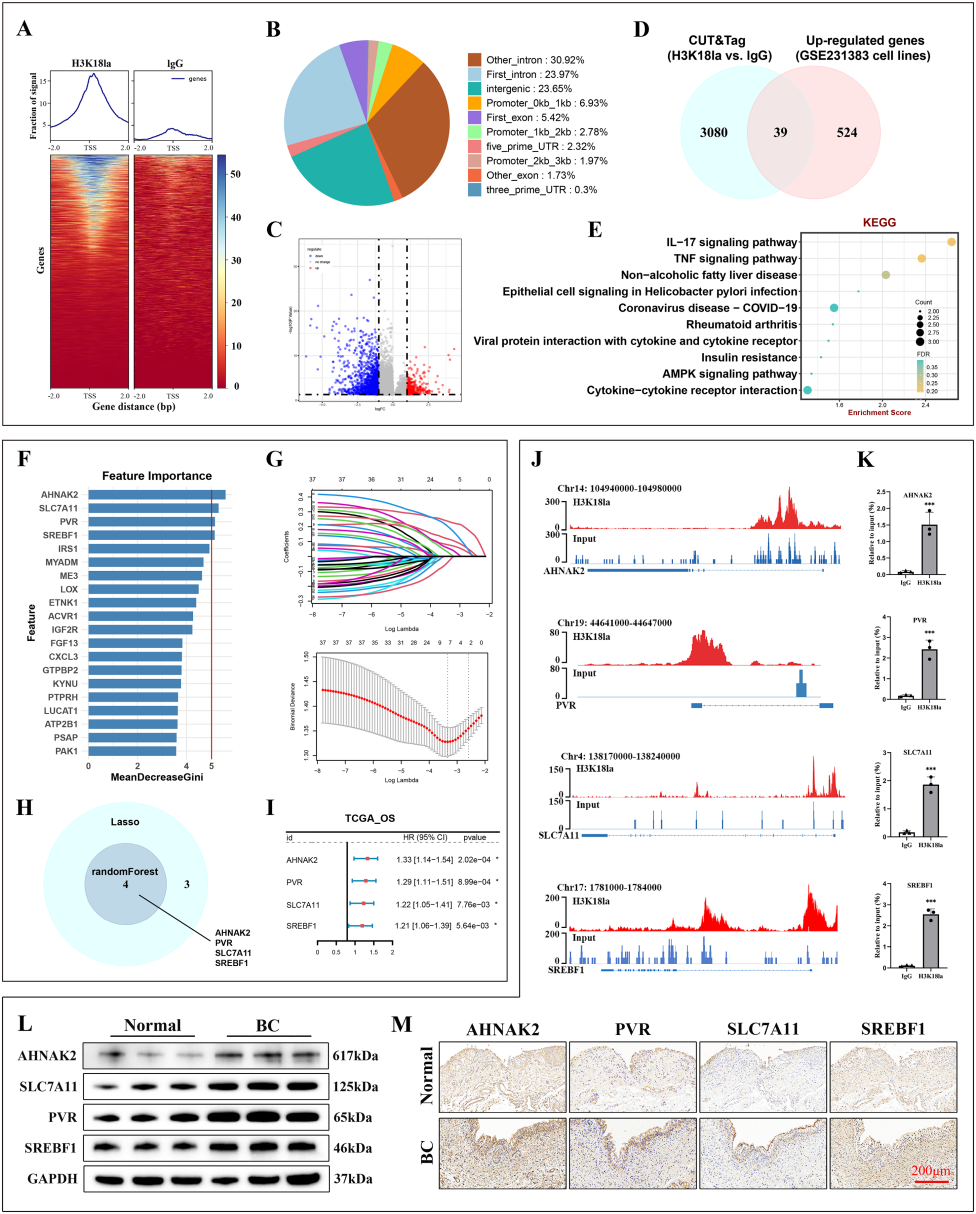
Identificating hubgenes for BC malignant development. **(A)** Heatmap displaying H3K18la peak distribution near the translation start site (TSS). **(B)** Genomic distribution of H3K18la. **(C)** Volcano plot showing differential genes in the bladder cancer cells relative to normal bladder epithelial cells. **(D)** Integration of CUT&Tag and BC cell lines for identifying potential downstream targets. **(E)** KEGG analysis of potential H3K18la targets. **(F)** Feature genes selected using the randomForest algorithm: x-axis = importance scores, y-axis = gene names; red line indicates the cutoff for selection. **(G)** A LASSO model identified significant genes. Partial likelihood deviance is plotted against log(λ); vertical lines mark the 1-SE rule for model selection. Genes with non-zero coefficients at the optimal λ were selected. **(H)** Combination of LASSO and RF to identify the hubgenes for BC malignant development. **(I)** Univariate Cox regression on hubgenes. **(J)** CUT&Tag-derived Integrative Genomics Viewer tracks showing H3K18la enrichment in the promoters of AHNAK2, PVR, SLC7A11, and SREBF1. **(K)** qPCR analysis of DNA fragments immunoprecipitated with the H3K18la antibody (n = 3). All data are presented as mean ± SD. Statistical analysis was performed by paired samples two-sided t-test (*p<0.05; **p<0.01; ***p<0.001; ns, not significant). The scattered points represent the distribution of the original data and reflect the variability of the data. **(L)** Western blot images and quantification of AHNAK2, SLC7A11, PVR, and SREBF1 protein levels in BC tissues compared to normal bladder tissues (n = 3). **(M)** Immunohistochemistry of AHNAK2, PVR, SLC7A11, and SREBF1 proteins in normal bladder tissues and BC tissues (n = 3).

We next aimed to investigate the impact of H3K18la on BC progression. As a recently identified epigenetic mark, histone lactylation has been demonstrated to influence gene transcription. A CUT&Tag assay utilizing the anti-H3K18la antibody identified 3118 genes (**Supplementary Table 5**). The H3K18la group showed a notable increase in the transcription start site (TSS) region (**Figure 8A**), accompanied by approximately 54% enrichment noted in intronic sequences (**Figure 8B**). These changes suggest that the increase in TSS may lead to more efficient transcription initiation, while the relative enrichment in intron regions has altered the splicing pattern of RNA and the stability of mRNA, which act together to significantly affect gene expression levels. In addition, the upregulated genes were screened in GSE231383 BC cell lines (**Supplementary Table 6**). Through the overlap of gene sets from CUT&Tag and BC cell lines, 39 marker genes were identified (**Figures 8C, D**; **Supplementary Table 7**). KEGG analysis of marker genes showed significant enrichment in immuno-inflammatory pathways, including IL-17 and TNF signaling, as well as cytokine-cytokine receptor interactions. Additionally, pathways involved in energy metabolism regulation, such as the AMPK pathway, insulin resistance, and non-alcoholic fatty liver disease, were significantly enriched (**Figure 8E**). To identify hub targets that promote tumor progression, RF and Lasso regression analyses were performed (**Figures 8F, G**). Finally, 4 hub genes (AHNAK2, PVR, SLC7A11, and SREBF1) were selected based on overlapping genes (**Figure 8H**).Univariate regression analysis validated the association of all four genes with prognosis in the TCGA-BLCA cohort (**Figure 8I**).

In the H3K18la group, a substantial increase in peak intensity was observed at the promoter regions of AHNAK2, PVR, SLC7A11, and SREBF1 (**Figure 8J**). ChIP-qPCR assays were used to validate this finding, confirming the enrichment of H3K18la at the gene promoters (**Figure 8K**). Moreover, our analysis revealed that the protein expression levels of AHNAK2, PVR, SLC7A11, and SREBF1 were significantly elevated in BC samples (**Figures 8L, M**). These observations collectively suggest that the activation of these four genes might play a positive role in the H3K18la-induced BC progression.

Furthermore, we explored the function of the four genes and their correlation with immune cells. Specifically, cell signaling transduction, inflammatory response, and immune activation are among the pathways where AHNAK2 is notably enriched. It was also observed to have a positive correlation with macrophages, activated T cells, neutrophils, mast cells, dendritic cells, and monocytes (**Supplementary Figure 1G**). PVR was markedly enriched in key biological processes involved in immune response, cell growth and proliferation, metabolism, and stress response. Furthermore, it showed a positive correlation with various innate immune cells, such as macrophages, dendritic cells, and monocytes (**Supplementary Figure 1H**). SLC7A11 was closely associated with various biological pathways, particularly those involved in cell metabolism and proliferation, redox equilibrium, and cell cycle regulation. It also demonstrated a strong correlation with activated CD4 T cells, dendritic cells, neutrophils, and eosinophils (**Supplementary Figure 1I**). SREBF1 influenced various cellular biological processes, particularly in lipid metabolism, cholesterol homeostasis, cell proliferation, and differentiation. However, it was not closely associated with immune cells (**Supplementary Figure 1J**).

### 3.6 Creation and validation of a BC prognosis risk score model using hub genes and its correlation with the immune microenvironment

**Figure 9:**
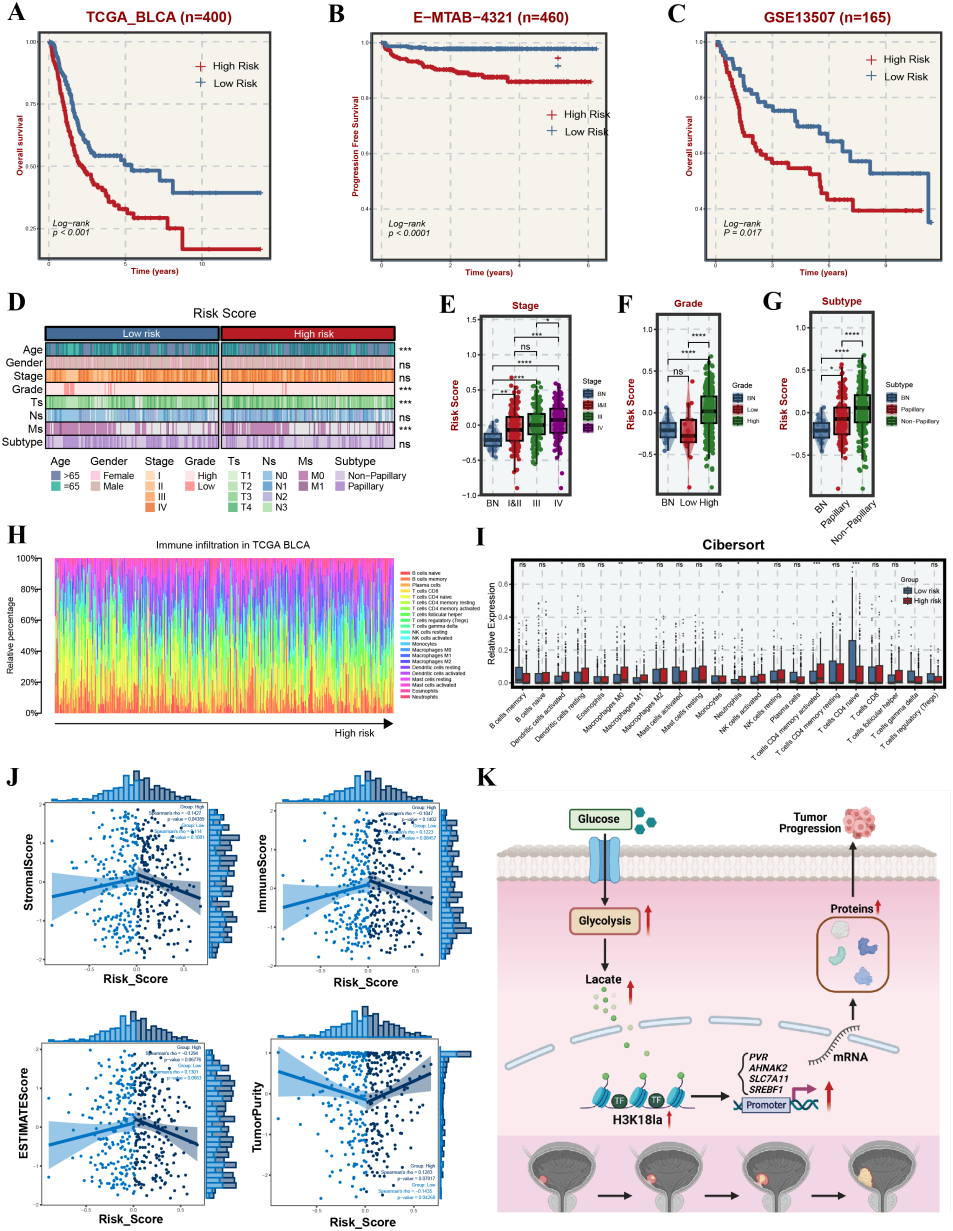
Construction of BC risk score and immune analysis. **(A)** Overall survival (OS) study of BC risk score in TCGA cohort. **(B)** Kaplan-Meier of progression-free survival (PFS) with log-rank test for BC risk score within E-MTAB-4321 cohort. **(C)** Kaplan-Meier of OS with log-rank test of BC risk score within GSE13507 cohort. **(D)** The distinct clinical characteristics between two BC risk groups. (*p<0.05; **p<0.01; ***p<0.001; ns, not significant). **(E-G)** The distribution plots for BC Stage, Grade, and Subtype between two BC risk groups. **(H)** CIBERSORT algorithm-based quantification of 22 immune infiltrates in the TCGA cohort. **(I)** The relative expression of 22 immune cells between two BC risk groups. (*p<0.05; **p<0.01; ***p<0.001; ns, not significant). **(J)** The BC risk score shows correlations with stromal, immune, ESTIMATE scores, as well as tumor purity. **(K)** How histone lactylation promotes the malignant progression of BC.

A risk model was developed to assess the influence of hub genes on the prognosis of BC patients. Dividing patients into high- and low-risk groups based on the median risk score revealed significant survival differences. Higher risk scores were associated with reduced overall survival and progression-free survival (PFS). To verify the stability and generalization of the model, this study adopted a multi-cohort independent validation strategy. In addition to the training set (TCGA-BLCA), the model also demonstrated good prognostic stratification ability in two large independent external validation sets, including E-MTAB-4321 and the GSE13507 cohort (**Figures 9A–C**). In the TCGA-BLCA cohort, age, grade, and stage significantly differed between the two groups (**Figure 9D**). The results of univariate and multivariate Cox regression analysis showed that the risk group was an independent prognostic factor for OS and disease-specific survival (DSS) in the TCGA-BLCA cohort (**Supplementary Figure 1K**). The risk score was notably elevated in patients with BC compared to normal individuals and demonstrated an increasing trend with higher stages and grades (**Figures 9E, F**). A greater prevalence of non-papillary growth patterns was associated with an increased risk score (**Figure 9G**).

Given that the four hub genes were variably implicated in the immune process, we examined the association between the risk score and immune profiles within the TCGA-BLCA cohort. CIBERSORT analysis revealed that an elevated risk score was associated with decreased B cell infiltration and increased infiltration of monocytes, macrophages, and dendritic cells (**Figure 9H**). The high-risk score group exhibited a significantly greater abundance of dendritic cells, macrophages, neutrophils, activated NK cells, and CD4 T cells (**Figure 9I**). The risk score showed a strong correlation with stromal, immune, and ESTIMATE scores, while displaying a significant inverse relationship with purity (**Figure 9J**). Analysis of the immune microenvironment indicated altered immune cell infiltration in high-risk patients. In the future, an in-depth multi-dimensional analysis combining genetic, immune microenvironment, and clinical data will improve the accuracy and clinical applicability of this model, offering more accurate guidance for the early diagnosis, prognosis evaluation, and immunotherapy of BC.

In conclusion, this study found that the mechanism by which enhanced glycolysis promotes BC progression was shown in **Figure 9K**. In BC, the dramatic enhancement of glycolysis leads to massive accumulation of lactate, which not only act as by-products of energy metabolism, but also serve as a substrate for epigenetic modification, directly promoting histone lactate modification, especially H3K18la. H3K18la is positively correlated with the transcription process of downstream oncogenes, including AHNAK2, PVR, SLC7A11 and SREBF1. The synergistic action of these genes together promotes the malignant progression of BC. This mechanism reveals how enhanced glycolysis drives tumor development through histone lactylation, providing new ideas for targeted intervention.

## 4 Discussion

In the global cancer context, among the many challenges facing the field of cancer medicine context is BC (33). Metabolic disorders are recognized as significant risk factors contributing to BC progression (33). Tumor cells undergo metabolic reprogramming to sustain their proliferation and support various cellular processes, a phenomenon now recognized as one of the key features of cancer (34). The Warburg effect, known as aerobic glycolysis, is a unique metabolic characteristic commonly observed in tumor cells (34, 35). Glycolysis is crucial in the metabolic reprogramming of BC cells and is closely associated with their malignant behavior. Tumor cells utilize the glycolytic pathway for energy production, leading to substantial lactate accumulation (35). Consequently, targeting the Warburg effect has emerged as a key direction for exploring novel therapeutic strategies for BC. In this study, we employed DCA and 2-DG as glycolysis inhibitors. DCA inhibits PDK to activate PDH to balance glycolysis and oxidative phosphorylation. It also can directly intervene in fatty acid metabolism, amino acid breakdown, cholesterol synthesis, and glutathione metabolism, forming multi-dimensional regulation of mitochondrial energy metabolism, lipid synthesis, and detoxification metabolism (36). 2-DG, as a glucose analogue, not only competitively inhibits hexokinase to block glycolysis but also interferes with the pentose phosphate pathway, protein N-glycosylation, lipid synthesis, and redox metabolism (37). Its effect is oxygen concentration-dependent. Under hypoxic conditions, it is mainly characterized by energy deficiency and oxidative stress, while it mainly exerts anti-tumor effects by inducing endoplasmic reticulum stress in normoxic conditions (37). However, our study replicated the results of drug treatment through the combined use of LDHA knockdown, strongly suggesting that the reduction of H3K18la and BC inhibition are mainly attributed to the decrease in lactate production rather than off-target effects. It is worth noting that DCA and 2-DG exert broad-spectrum interference on the cellular metabolic network, and the tumor metabolism itself exhibits significant heterogeneity and plasticity (38). When glycolysis is inhibited, tumor cells are highly likely to activate compensatory metabolic mechanisms, such as upregulation of oxidative phosphorylation, enhancement of glutamine breakdown or fatty acid oxidation, in order to bypass the metabolic blockage and maintain survival (39). In view of this, although glycolysis inhibitors have to some extent weakened the glycolysis flux, it has not yet systematically evaluated the activation status of these potential compensatory pathways. Future studies need to combine metabolic flux analysis and key enzymatic detection to comprehensively analyze the metabolic remodeling response triggered by glycolysis inhibition, and explore a combined treatment paradigm that targets the compensatory pathways such as glutaminase inhibitors to overcome the limitations of single metabolic targeting therapy.

In the past, lactate was often regarded as a by-product of glycolytic metabolism. Recent studies indicate that lactate can alter histone lysine residues, influencing epigenetic regulation (40). In this study, an increase in glycolysis levels was observed accompanied by a continuous accumulation of lactate. This finding strongly supports lactate’s role as a substrate in histone lactylation. We were the first to elucidate the role of histone lactylation, particularly H3K18la, in the development of BC in both *in vitro* and *in vivo* settings. Nevertheless, while our study delineates the critical role of H3K18la in BC, the lactoyltransferases that directly catalyze H3K18la modification and their cognate delactoylases remain to be identified in BC. Emerging evidence indicates that p300/CBP may function as histone lactoyltransferases responsible for H3K18la deposition, whereas classical deacetylases including HDACs and SIRTs have been reported to exhibit delactoylation activity (41–43). However, whether these enzymes directly modulate H3K18la in BC, as well as their tissue specificity and crosstalk with lactate metabolism, warrant further mechanistic investigation. Future studies can systematically identify the key writers and erasers that regulate H3K18la in BC through CRISPR screening, mass spectrometry analysis, and specific inhibitors. This will further reveal the dynamic equilibrium mechanism of lactylation modification in the epigenetic regulatory network and provide more precise molecular targets for intervention strategies targeting this modification.

Research consistently shows that metabolic reprogramming and epigenetic remodeling are interdependent processes that collectively drive tumor evolution across various diseases (44). In an animal model of sclera hypoxia-induced myopia, elevated sugar intake facilitates myopia progression via H3K18la and activates the glycolysis-lactate-histone modification pathway in the sclera, amplifying the response to myopia induction (45). In pancreatic ductal adenocarcinoma, enhanced H3K18la is enriched in promoter regions and promotes transcriptional activation of the mitotic checkpoint regulator TTK with BUB1B, which in turn promotes glycolysis and upregulates lactate and H3K18la levels (46). This process establishes a positive metabolic-epigenetic-metabolic feedback loop, which subsequently contributes to heightened metabolic disruption and dysfunction of pancreatic ductal epithelial cells. It is worth noting that studies on other lactylation sites, such as H3K18la, H4K12la, and H3K9la, have also revealed their crucial roles in different cancer types and drug resistance mechanisms (47). For example, in cisplatin-resistant BC, curcumol markedly reduces H3K9la levels by suppressing glycolysis and lactic acid production, thereby modulating the expression of its downstream target ORC6, inducing ferroptosis, and effectively reversing cisplatin resistance (48). These findings indicate that H3K9la may represent a promising therapeutic target for overcoming chemotherapy resistance. Additionally, the accumulation of lactate due to abnormal glycolysis activation can promote H4K12la modification in ovarian cancer, which activates the MYC transcription factor to activate RAD23A expression, enhancing DNA damage repair capabilities, and thereby mediating niraparib resistance (49). Similarly, in non-small cell lung cancer, H4K12la interacts with the transcription factor CEBPB to modulate the expression of AKR1C2, thereby activating the mTOR signaling cascade and fostering cisplatin resistance (50). Targeting this axis may effectively reverse chemoresistance. These findings emphasize the importance of the specificity of histone lactylation sites in tumor resistance, and further indicate that different lactylation sites have independent and crucial regulatory functions in various cancers, although they have not been systematically explored in BC. Moreover, the functional scope of lactylation is extending from histones to non-histones. Increasing studies have shown that non-histone lactylation also plays an important role in tumor progression, such as the lactylation modifications of AARS1, ACSS2, and MRE11, which participate in key processes such as metabolism and DNA damage repair, suggesting that exploring non-histone lactylation in BC is also of great value (47).

This study confirmed that elevated lactate levels promoted the proliferation of BC cells by enhancing histone lactylation, which in turn activated transcription factors. More importantly, four H3K18la target genes (AHNAK2, PVR, SLC7A11, and SREBF1) were identified, which were strongly associated with a high risk of BC and linked to abnormal tumor metabolism. Firstly, C14orf78, also known as AHNAK2, was originally identified in mouse heart tissue. Recent research indicates that AHNAK2 overexpression is linked to poor prognosis in several cancers, such as clear cell renal cell carcinoma, pancreatic ductal adenocarcinoma, thyroid cancer, and lung adenocarcinoma (51–54). Through promoting epithelial-mesenchymal transition, AHNAK2 plays a key regulatory role in tumor progression (55). Despite limited research on AHNAK2 in BC, high AHNAK2 expression has been linked to shorter overall survival in BC patients, and its knockdown significantly inhibits the invasive ability of BC cells (56, 57). In addition, the poor prognosis associated with AHNAK2 in BC may be linked to the activation of fibroblast growth factor-1. This factor activates all fibroblast growth factor receptors (FGFR), which subsequently drives BC progression (58). The Food and Drug Administration has approved FGFR inhibitors for use in BC patients (59). Exploring the combined anti-tumor effects of targeting AHNAK2 and FGFR in future studies may prove valuable. Furthermore, the poliovirus receptor, also known as PVR or CD155, demonstrates significant overexpression in BC and is linked to a poor clinical outcome (60). As one of the potential target genes identified by this study for H3K18la, PVR had a significantly elevated expression in muscle-invasive BC and was predominantly localized to the cell membrane of tumor cells (61). Additionally, PVR overexpression is closely related to poor overall survival rate, reduced tumor-infiltrating lymphocytes, higher histological stage and grade (61, 62). Moreover, the SLC7A11-GSH system plays a crucial role in protecting cells from ferroptosis (63). SLC7A11 overexpression inhibits ferroptosis and facilitates tumor progression (64). Multiple studies have revealed the regulatory network of SLC7A11 in BC. Phosphoglycerol dehydrogenase upregulates SLC7A11 by inhibiting the ubiquitination of PCBP2, promoting the malignant progression of BC (64). Deubiquitylase USP52 promotes BC development by stabilizing the SLC7A11/xCT complex (65). HSPA5 suppresses ferroptosis via the P53/SLC7A11/GPX4 pathway, while p53 induces ferroptosis by activating ALOX15B (66, 67). Additionally, IGF2BP2 activates SLC7A11 under hypoxic conditions, inhibiting cisplatin-induced apoptosis and ferroptosis, thereby contributing to chemotherapy resistance in BC (68). The negative regulation of SLC7A11 by miRNA-27a may become a potential biomarker for cisplatin chemotherapy (69). Taken together, SLC7A11 and its regulatory pathway serve as important targets and potential biomarkers for the treatment of BC through ferroptosis, and its localization downstream of H3K18la further reveals the epigenetic regulatory bridge between metabolic reprogramming and ferroptosis. It is worth noting that lipid metabolism plays a crucial role in tumor cell signal transduction and membrane synthesis, and is highly integrated with glucose and amino acid metabolism through the tricarboxylic acid cycle, jointly regulating tumor growth and therapeutic response (70). Fatty acid metabolism (FAM) reprogramming has been demonstrated a strong correlation with metastasis and therapeutic response in gastric cancer, and it affects the treatment outcome by regulating the tumor immune microenvironment (71). Fatty acid synthase (FASN) and stearoyl-CoA desaturase 1 (SCD1) are key enzymes in fatty acid metabolism, among which the expression of SCD1 is regulated by SREBF1 (72). In BC, FGFR3 activated SREBF1 through the PI3K-mTORC1 pathway, thereby regulating lipid metabolism to promote tumor growth (72). Moreover, metformin inhibits SREBF1 and its downstream target FASN, slowing BC progression (73). The SREBF1-regulated lipogenesis pathway in lipid metabolism is crucial for tumor growth, survival, and therapy response, making it a promising therapeutic target. These findings open up entirely new perspectives on the metabolic regulatory mechanisms in BC and highlight potential targets of action for future therapeutic strategies.

The combined application of glycolysis inhibitors with immunotherapy or targeted therapy is expected to become a highly promising treatment strategy for BC. Lactic acid, as a core metabolic product, can construct an immunosuppressive barrier by acidifying the tumor microenvironment, inhibiting the function of CD8^+^ T cells, promoting the polarization of M2-type macrophages, and facilitating the infiltration of Treg cells (74). By blocking the glycolysis pathway through DCA, 2-DG, or LDHA inhibitors, it can effectively reduce the generation of lactate, improve the acidification state of the microenvironment, and restore the immune surveillance function of the body, thereby significantly enhancing the anti-tumor effect of immune checkpoint inhibitors. The combination of glycolysis inhibition with targeted therapy, such as targeting the key genes identified in this study, can achieve a synergistic anti-tumor effect, which is helpful in overcoming the limitations and resistance problems of single treatment. In summary, the combined treatment strategy centered on glycolysis inhibition can regulate the interaction between tumor metabolism and the immune microenvironment, providing a more efficient, stable, and durable treatment plan for BC.

In recent years, several studies have successfully constructed different prognosis models for BC by integrating multi-omics data and machine learning algorithms. A study constructed a prognosis model consisting of 16 genes based on mitotic catastrophe heterogeneity (75). Yan et al. identified 8 key genes related to immunogenic cell death characteristics and constructed a prognosis model (76). Both of these models demonstrate robust prognostic efficacy, which can effectively reveal the tumor microenvironment characteristics or genomic variations, providing reliable basis for the precise prognosis assessment and individualized treatment of BC. Compared with the above models, the four-gene prognosis model constructed in our study has relatively limited predictive performance. However, through strict univariate and multivariate Cox regression analysis and validation in multiple external cohorts, this model exhibits advantages of structural simplicity and convenient detection, which has certain practical value in rapid clinical screening and resource-limited application scenarios. In the future, it is necessary to further verify through a prospective large-sample cohort and integrate multi-dimensional clinical variables and biomarkers to optimize the model, thereby improving its prognostic prediction accuracy and clinical application potential.

Although this study revealed that glycolysis and histone lactylation contribute to BC progression through the regulatory crosstalk of metabolic networks, there are undeniable limitations. Firstly, while our integrative CUT&Tag and RNA-seq analyses revealed a strong correlation between H3K18la enrichment at the promoter regions of AHNAK2, PVR, SLC7A11, and SREBF1 and their transcriptional upregulation, the mechanistic link between H3K18la and the transcriptional activation of these genes remains correlative. Direct evidences, such as promoter mutagenesis to disrupt putative lactylation sensitive elements or rescue experiments with lactylation-deficient histone mutants, is needed to establish a causal relationship. Secondly, our study do not investigate potential functional redundancy or synergy among these four target genes. Whether they act independently or cooperatively to promote BC progression remains unclear. Future studies employing combinatorial knockdown or knockout approaches, as well as functional interaction analyses, will be required to address this question. Moreover, this study was mainly based on two BC cell lines, 5637 and T24. It did not include patient-derived models or organoid systems for verification. The *in vivo* experiments used a subcutaneous xenograft model, which could not fully replicate the tumor microenvironment or metastasis behavior of BC. Therefore, the generalizability and clinical translational significance of the current conclusions still need to be further confirmed in more diverse and closer-to-real *in vivo* environment models. Lastly, the correlation analysis between immune cells and four risk genes is still at the level of association. The specific role of the tumor immune microenvironment in glycolysis and H3K18la regulation has not been fully elucidated and requires further in-depth exploration through subsequent studies.

## 5 Conclusion

This study provides new insights into the metabolic reprogramming and epigenetic regulation of BC. Our findings demonstrate that elevated glycolysis-associated histone lactylation (H3K18la) is linked to poor prognosis, highlighting its potential as a prognostic marker. Inhibition of glycolysis or LDHA knockdown suppresses BC growth, suggesting lactate metabolism as a potential therapeutic target. Furthermore, H3K18la is positively correlated with the expression of key oncogenic genes (AHNAK2, PVR, SLC7A11, SREBF1), accompanying BC progression in association with metabolic and immunosuppressive mechanisms. Collectively, these findings reveal a correlative epigenetic network linking glycolysis to H3K18la-associated gene expression, providing a foundation for future mechanistic studies and therapeutic strategies targeting lactylation in BC.

## Data Availability

The original contributions presented in the study are included in the article/**Supplementary Material**. Further inquiries can be directed to the corresponding authors.

## Supplementary material

Supplementary Figure 1 Single-cell landscape and functional enrichment of BC. (**A**) UMAP visualization of 42,440 cells, color-coded by clusters. (**B**) UMAP plot of expected outcomes for the cell cycle analysis. (**C**) Network of interaction quantity and intensity among five cell types. (**D**) Violin diagrams displaying DEG expression levels in individual cell clusters. (**E**) Single-cell pathway enrichment analysis of BC and BN samples. (**F**) Relative expression of glycolysis signature genes between BC and BN samples. (**G-J**) Single gene GSEA enrichment analysis and immune correlation analysis for AHNAK2, PVR, SLC7A11, and SREBF1, respectively. (**K**) Forest plots of univariate and multivariate Cox regression analyses for OS and DSS based on TCGA-BLCA cohort. (**L**) Western blot and quantitative statistical analysis of histone lactylation markers H3K9la and H4K12la in clinical normal tissue and BC tissue.

Supplementary Table 1 Summary of datasets in this study.

Supplementary Table 2 Lactylation related genes.

Supplementary Table 3 DEGs in high versus low lactylation-AUC groups.

Supplementary Table 4 Lactylation-related survival genes (LRSGs).

Supplementary Table 5 The result of CUT&Tag assay.

Supplementary Table 6 Up-regulated genes of BC cell lines.

Supplementary Table 7 Potentially malignant genes in BC progression.
