# Supplementary material for: Glycolysis-mediated H3K18la modifications drive aggressive bladder cancer through metabolic and epigenetic reprogramming
Source: Front Immunol. 2026 Jun 30;17:1777092. doi: 10.3389/fimmu.2026.1777092 (PMC13364861; doi:10.3389/fimmu.2026.1777092)
Supplement: Supplementary Table 1 — Summary of datasets in this study. [file DataSheet1.pdf]

Table S1. Summary of datasets in this study

Datasets	Platform	Number of samples	Technology	PMID	Survival	Survival samples	Gene numbers	Source
GSE135337	Illumina NovaSeq 6000	8	scRNA-seq	34480339	No		21489	https://www.ncbi.nlm.nih.gov/geo/
TCGA_BLCA	Illumina	418	RNA-seq		Yes	418	60484	https://xena.ucsc.edu/
E-MTAB-4321	Illumina HiSeq 2000	460	RNA-seq	27321955	Yes	460	38272	https://www.ebi.ac.uk/biostudies/arrayexpress
GSE13507	Illumina human-6 v2.0 expression beadchip	165	Microarray	20421545	Yes	165	24741	https://www.ncbi.nlm.nih.gov/geo/

Table S2. Lactylation related genes from previous research

Lactylation related genes from previous research
HDAC1
HDAC2
HDAC3
SIRT1
SIRT2
SIRT3

EP300
ACAT2
ACIN1
ADAR
ADNP
AHNAK
ALB
ALDH1A1
ALDOA
ALDOB
ALYREF
ARGLU1
ARID1A
ARID3A
ARID3B
ARPP19
BCLAF1
BOLA2
BRD4
BTF3
BZW1
BZW2
C19orf53
CACYBP
CALD1
CALM1

CALML5
CALR
CBR1
CBX3
CBX5
CCNA2
CCT5
CD2BP2
CDC5L
CDV3
CDV3
CDYL
CEBPZ
CFDP1
CHD4
CHERP
CNN2
CNN3
COPS4
CRABP2
CSRP1
CSRP2
CWC15
DDX17
DDX18
DDX21

DDX39A
DDX39B
DDX3X
DDX41
DDX42
DDX46
DDX5
DECR1
DFFA
DHRS7
DHX16
DHX9
EAF1
ECHDC1
EDF1
EEF1A1
EEF1G
EEF2
EHMT2
EIF3D
EIF3J
EIF4G1
EIF4G2
EIF4H
EMG1
ENO1

ENSA
FABP5
FAM50A
FKBP3
FLYWCH2
FUBP1
G6PD
GAPDH
GATAD2A
GATAD2B
GFAP
GIGYF2
GTF2F1
GTF2I
H1-2
H1-3
H1-5
H2AFV
H2AFZ
H2AJ
H2AX
H2AZ1
H2AZ2
H2BC13
H2BC14
H2BC18

H2BC5
H2BU1
H3-3A
H3C1
H3C15
H4C1
HCFC1
HDGF
HDGFL2
HEXIM1
HIST1H1C
HIST1H2BB
HIST1H2BD
HIST1H2BH
HIST1H2BK
HIST1H2BL
HIST1H2BN
HIST1H2BO
HIST1H3A
HIST1H4A
HIST2H2BE
HIST2H2BF
HISTIH2BH
HLTF
HMGA1
HMGB1

HMGN1
HMGN2
HMGN3
HMGN4
HNRNPA1
HNRNPC
HNRNPD
HNRNPF
HNRNPH1
HNRNPK
HNRNPL
HNRNPM
HNRNPU
HSDL2
HSPE1
IARS2
IFI16
IK
IKZF1
ILF2
ILF3
IRF2BP2
JMJD1C
JPT1
JPT2
KHDRBS1

KHSRP
KIF2C
KRT1
KRT10
LAP3
LCP1
LDHB
LEMD3
LGALS1
LRPPRC
LSP1
MAGOH
MAGOHB
MAP2K4
MAPRE1
MBD2
MBP
MDC1
MKI67
MNDA
MPHOSPH6
MSN
MTA1
MTA2
MYH13
NCDN

NCL
NEFL
NHLRC2
NOC3L
NOLC1
NONO
NOP2
NPM1
NSUN2
NUCKS1
NUDT21
NUDT5
NUP133
NUP50
PABPC1
PABPN1
PAK2
PARP1
PCBP1
PCBP2
PCMT1
PCNP
PDAP1
PDLIM1
PES1
PFKM

PFKP
PFN1
PGK1
PHC3
PHF6
PKM2
POLDIP3
PPIA
PPIL4
PPM1G
PPP1CB
PPP1CC
PPP1R2B
PRAM1
PRCC
PRDX1
PRKDC
PRPF6
PSMA7
PSMC1
PSME3IP1
PTBP1
PTMA
RACGAP1
RALYL
RAN

RANBP2
RB1
RBM10
RBM14
RBM17
RBM25
RBM39
RBMX
RCC2
RECQL
RFC1
RFC4
RIMS1
RPA1
RPL13
RPL14
RPL22
RPL24
RPL29
RPL5
RPS11
RPS23
RPS27A
RRP1B
RSL1D1
S100A11

S100A4
S100A6
SAFB
SARNP
SATB1
SET
SF3A1
SF3B1
SFPQ
SFPQ
SH3GL1
SMAP
SMARCA5
SMARCC1
SMARCC2
SMC3
SNRPA1
SOD1
SPR
SPR14
SRP14
SRRM1
SRRM2
SSB
STMN1
SUB1

SUMO2
TCOF1
TERF2
THOC2
THRAP3
THUMPD1
TKT
TMA7
TMPO
TMSB4X
TOP2B
TP53
TPM4
TPR
TRIM28
TRIR
TSSC4
U2AF2
U2SURP
UBE2E1
UBE2M
UPF1
VARS
VIM
WAS
WBP11

WDR33
WIZ
XPO5
XRCC4
YLPM1
ZC3H18
ZC3H4
ZMYM3
ZNF207
ZNF280C
ZNF706
ZNFX1
ZRANB2
ZYX

Table S3. Differentially expressed genes in high- and low-lactylation-AUC groups.

	X	ID	p_val	avg_log2FC	pct.1	pct.2	p_val_adj	regulate
1	1	RSL1D1	0	0.607820250607141	0.722	0.48	0	no change
2	2	RBMX	0	0.514582960148989	0.696	0.466	0	no change
3	3	EIF3D	0	0.691410606969653	0.707	0.478	0	no change
4	4	HNRNPM	0	0.514447391019666	0.676	0.45	0	no change

5	5	C1QBP	0	0.540056514175186	0.684	0.461	0	no change
6	6	ANP32B	0	0.552819768297978	0.596	0.375	0	no change
7	7	CCT5	0	0.803235183911056	0.508	0.29	0	no change
8	8	EIF4B	0	0.459989003573785	0.684	0.468	0	no change
9	9	SYT8	0	0.376330357811389	0.733	0.518	0	no change
10	10	NONO	0	0.574869481200099	0.566	0.355	0	no change
11	11	FBP1	0	0.643747700585338	0.67	0.46	0	no change
12	12	NSG1	0	0.838316684807062	0.498	0.293	0	no change
13	13	ODC1	0	1.0743917788649	0.399	0.195	0	up
14	14	NBEAL1	0	0.651261323231813	0.774	0.571	0	no change
15	15	NUPR1	0	-1.487585257	0.295	0.497	0	down
16	16	PGD	0	0.803376427570272	0.41	0.218	0	no change
17	17	CDKN2A	0	0.784972493787244	0.352	0.162	0	no change
18	18	EIF3L	0	0.652937596265301	0.831	0.644	0	no change

19	19	NAP1L1	0	0.425625759556397	0.835	0.65	0	no change
20	20	PIR	0	0.710395846839194	0.401	0.218	0	no change
21	21	TUBB	0	0.690922043847273	0.832	0.654	0	no change
22	22	RPL17	0	0.511806129633178	0.813	0.637	0	no change
23	23	GPNMB	0	-0.966099074	0.359	0.534	0	no change
24	24	LDHB	0	0.543111952127842	0.901	0.731	0	no change
25	25	CYP24A1	0	1.55432217548168	0.253	0.083	0	up
26	26	HNRNPC	0	0.391291007808648	0.883	0.714	0	no change
27	27	SCGB3A1	0	0.73470180105267	0.23	0.066	0	no change
28	28	PCBP2	0	0.420959182749942	0.888	0.727	0	no change
29	29	HNRNPK	0	0.397919114787131	0.887	0.727	0	no change
30	30	ATP5B	0	0.528349650606596	0.866	0.707	0	no change
31	31	SLC7A11	0	1.78356456722044	0.218	0.061	0	up
32	32	RAN	0	0.48737202282255	0.914	0.764	0	no change

33	33	PDLIM1	0	0.503096088324693	0.806	0.658	0	no change
34	34	HOXB6	0	1.35469812797216	0.198	0.064	0	up
35	35	CES1	0	1.06657628166484	0.19	0.063	0	up
36	36	NPM1	0	0.526889445516896	0.971	0.877	0	no change
37	37	IFI27	0	-1.124467133	0.701	0.791	0	down
38	38	EEF2	0	0.681087521901671	0.957	0.867	0	no change
39	39	RPL36A	0	0.593784259930997	0.96	0.878	0	no change
40	40	EEF1B2	0	0.572659493282191	0.956	0.876	0	no change
41	41	SLPI	0	-1.343896738	0.639	0.71	0	down
42	42	DNAJB1	0	-0.745228413	0.747	0.813	0	no change
43	43	ZFAS1	0	0.337412229226114	0.955	0.89	0	no change
44	44	HNRNPA1	0	0.582819605454046	0.984	0.92	0	no change
45	45	HINT1	0	0.48305260187217	0.976	0.914	0	no change
46	46	TXN	0	0.452283244186707	0.961	0.899	0	no change
47	47	LGALS3	0	-0.629057216	0.675	0.733	0	no change

48	48	PABPC1	0	0.277422446572447	0.981	0.923	0	no change
49	49	IER2	0	-0.759545277	0.749	0.804	0	no change
50	50	UQCRH	0	0.534575527912309	0.986	0.936	0	no change
51	51	RPS10	0	0.406672619196153	0.982	0.937	0	no change
52	52	YBX1	0	0.323604500383739	0.989	0.954	0	no change
53	53	BTF3	0	0.465933293185808	0.992	0.959	0	no change
54	54	GSTP1	0	0.335788424123871	0.992	0.959	0	no change
55	55	PHLDA2	0	-0.844251846	0.827	0.86	0	no change
56	56	NPC2	0	-0.548083304	0.897	0.869	0	no change
57	57	RPL4	0	0.459472142062769	0.993	0.972	0	no change
58	58	RPL7A	0	0.674229759694153	0.994	0.976	0	no change
59	59	RPSA	0	0.456396393283618	0.997	0.979	0	no change
60	60	NACA	0	0.369113437669652	0.994	0.977	0	no change

61	61	RPL5	0	0.538301099935562	0.997	0.98	0	no change
62	62	GNB2L1	0	0.612826816011582	0.998	0.988	0	no change
63	63	UBC	0	-0.505237334	0.962	0.952	0	no change
64	64	RPLP0	0	0.347820292771313	0.998	0.99	0	no change
65	65	RPL22	0	0.356195745363739	0.999	0.992	0	no change
66	66	RPL23	0	0.277307816944685	0.999	0.993	0	no change
67	67	RPL26	0	0.512979464150624	1	0.995	0	no change
68	68	RPL27	0	0.251857277712739	0.999	0.994	0	no change
69	69	RPL6	0	0.592360420449635	0.999	0.994	0	no change
70	70	RPS21	0	0.26544820220503	1	0.995	0	no change
71	71	CST3	0	-0.618130788	0.984	0.98	0	no change
72	72	RPL38	0	0.264536894717454	0.999	0.995	0	no change
73	73	RPS3A	0	0.619985563942994	0.999	0.995	0	no change

74	74	RPL10A	0	0.468549208083845	1	0.997	0	no change
75	75	RPL24	0	0.32059213619304	0.999	0.996	0	no change
76	76	RPL35	0	0.446615852450996	1	0.997	0	no change
77	77	RPL9	0	0.421725369512781	0.999	0.996	0	no change
78	78	RPS13	0	0.329344394276327	1	0.997	0	no change
79	79	RPS24	0	0.428658290589744	0.999	0.996	0	no change
80	80	RPS5	0	0.239875264780583	1	0.997	0	no change
81	81	RPS7	0	0.400277978042289	1	0.997	0	no change
82	82	SAT1	0	-0.718140192	0.91	0.913	0	no change
83	83	PTMA	0	0.265080846584557	1	0.998	0	no change
84	84	RPL12	0	0.57806228900137	1	0.998	0	no change
85	85	RPL14	0	0.323295504288937	1	0.998	0	no change
86	86	RPL18	0	0.24465520161193	1	0.998	0	no change

87	87	RPL7	0	0.592643700912407	1	0.998	0	no change
88	88	RPS11	0	0.226169550051319	0.999	0.997	0	no change
89	89	RPS16	0	0.235705320803716	1	0.998	0	no change
90	90	RPS17	0	0.419529283810081	1	0.998	0	no change
91	91	RPS20	0	0.268624022510765	1	0.998	0	no change
92	92	RPS3	0	0.488354986055429	1	0.998	0	no change
93	93	RPS4X	0	0.543518982913072	1	0.998	0	no change
94	94	EEF1A1	0	0.537833930618811	1	0.999	0	no change
95	95	RPL18A	0	0.324645207203806	1	0.999	0	no change
96	96	RPL3	0	0.489994937258625	1	0.999	0	no change
97	97	RPL31	0	0.433827382633767	1	0.999	0	no change
98	98	RPL35A	0	0.322898310787225	1	0.999	0	no change
99	99	RPL36	0	0.260682572671333	1	0.999	0	no change

100	100	RPL37	0	0.351825956126649	1	0.999	0	no change
101	101	RPL37A	0	0.246164853999384	1	0.999	0	no change
102	102	RPL8	0	0.323799720958224	1	0.999	0	no change
103	103	RPS15A	0	0.431810885063496	1	0.999	0	no change
104	104	RPS23	0	0.314839198196493	1	0.999	0	no change
105	105	RPS27A	0	0.37796878350635	1	0.999	0	no change
106	106	RPS6	0	0.393330915141778	1	0.999	0	no change
107	107	RPS8	0	0.387249797800719	1	0.999	0	no change
108	108	RPS9	0	0.284081108137772	1	0.999	0	no change
109	109	B2M	0	-0.461660651	1	1	0	no change
110	110	FTH1	0	-0.682912745	1	1	0	no change
111	111	FTL	0	-0.769065293	1	1	0	no change
112	112	RPL10	0	0.287411344006711	1	1	0	no change

113	113	RPL11	0	0.38172307871042	1	1	0	no change
114	114	RPL13	0	0.339117386640384	1	1	0	no change
115	115	RPL13A	0	0.294924953352496	1	1	0	no change
116	116	RPL15	0	0.444788608881153	1	1	0	no change
117	117	RPL19	0	0.180578062313804	1	1	0	no change
118	118	RPL27A	0	0.273322768397381	1	1	0	no change
119	119	RPL32	0	0.351916011860456	1	1	0	no change
120	120	RPL39	0	0.354647312590509	1	1	0	no change
121	121	RPL41	0	0.267523854962709	1	1	0	no change
122	122	RPS14	0	0.303932917432013	1	1	0	no change
123	123	RPS15	0	0.242758606941765	1	1	0	no change
124	124	RPS18	0	0.453499262727933	1	1	0	no change
125	125	RPS19	0	0.23667694065837	1	1	0	no change

126	126	RPS2	0	0.55310893251205	1	1	0	no change
127	127	S100A6	0	-0.643798641	0.998	0.998	0	no change
128	128	TMSB4X	0	-0.42570513	1	1	0	no change
129	129	JUN	1.34753023130703e-307	-0.671886218	0.77	0.823	2.94354503726707e-303	no change
130	130	CDKN1A	7.61705733752215e-307	-0.640978184	0.728	0.757	1.66387000480834e-302	no change
131	131	HMGN1	7.66238936915874e-307	0.359142899608207	0.941	0.812	1.67377233379904e-302	no change
132	132	HS6ST3	1.21831551897713e-306	2.00718585820827	0.119	0.025	2.66128841965364e-302	up
133	133	HDAC2	1.56440040901291e-306	0.526229251726873	0.566	0.35	3.41727625344779e-302	no change
134	134	NCL	1.65335339030112e-306	0.395641405891123	0.8	0.583	3.61158514577377e-302	no change
135	135	HMGB1	4.3266131913544e-305	0.347178525962781	0.982	0.926	9.45105385519454e-301	no change
136	136	CLU	4.56873695542892e-305	-1.134589873	0.641	0.733	9.97994900543894e-301	down
137	137	SLC25A6	7.20995913960162e-301	0.278693588606096	0.987	0.949	1.57494347445458e-296	no change
138	138	CBR1	2.83019097510574e-300	0.690159468100461	0.504	0.317	6.18226916602099e-296	no change

139	139	SERF2	2.6009560696063e-298	-0.277520123	0.999	0.998	5.681528438448e-294	no change
140	140	MALAT1	4.09353623650307e-295	-0.386383354	1	1	8.9419205550173e-291	no change
141	141	H3F3B	1.14177919386555e-294	-0.390159543	0.998	0.995	2.49410247107992e-290	no change
142	142	PHB2	1.15696120230913e-293	0.494277246606769	0.629	0.418	2.52726605032407e-289	no change
143	143	PRKDC	7.61216102412981e-292	0.642128446649688	0.374	0.196	1.66280045411092e-287	no change
144	144	BCAM	1.82949797809633e-291	0.7991342772655	0.455	0.289	3.99635538335362e-287	no change
145	145	C1orf186	4.62279566327926e-291	0.777698942854074	0.31	0.149	1.00980348468672e-286	no change
146	146	INSIG1	2.60203654583305e-289	-1.338709906	0.283	0.412	5.68388863071771e-285	down
147	147	FXYD3	7.6434121654078e-287	-0.424887192	0.982	0.934	1.66962695341168e-282	no change
148	148	JUNB	2.09723967202779e-283	-0.566708542	0.922	0.915	4.5812103395775e-279	no change
149	149	FABP4	1.91574627165629e-281	0.660459558694359	0.479	0.298	4.18475615580601e-277	no change
150	150	KRT5	2.35085255907795e-278	0.608662594651251	0.287	0.133	5.13520233004987e-274	no change
151	151	CAPNS2	4.60806858759821e-275	0.548973490241537	0.379	0.202	1.00658650227495e-270	no change

152	152	SLC25A5	3.92913124107977e-273	0.385542487135062	0.894	0.759	8.58279428301466e-269	no change
153	153	PSCA	6.31128059525268e-270	-0.77987657	0.944	0.907	1.378636133227e-265	no change
154	154	ATP5A1	7.31832095953566e-270	0.410903133090258	0.764	0.57	1.59861403040097e-265	no change
155	155	ILF2	1.69830123519364e-268	0.483511486336086	0.602	0.396	3.70976921815698e-264	no change
156	156	HDAC1	1.01093811514665e-267	0.577111739269782	0.429	0.246	2.20829321872633e-263	no change
157	157	PARK7	3.94393958448207e-266	0.371669336729225	0.881	0.768	8.61514162834263e-262	no change
158	158	ZFP36	1.52225862586563e-265	-0.629027775	0.764	0.817	3.32522174234088e-261	no change
159	159	ENO1	2.91341309204094e-262	0.485983663684829	0.871	0.733	6.36405955825423e-258	no change
160	160	S100P	4.36244473865845e-261	-0.342164704	0.994	0.973	9.52932428712552e-257	no change
161	161	HADHA	7.77716103973034e-261	0.452774328306284	0.557	0.351	1.6988430575187e-256	no change
162	162	EIF3M	5.04521609504931e-260	0.384410398559391	0.731	0.519	1.10207700380257e-255	no change
163	163	FBL	3.96125697013587e-259	0.407539806381516	0.678	0.468	8.65296972556479e-255	no change
164	164	FOS	2.75261875716265e-257	-0.502763926	0.851	0.903	6.0128204131461e-253	no change

165	165	IFI6	3.67924123273748e-257	-0.927006133	0.515	0.611	8.03693454879176e-253	no change
166	166	SLITRK6	1.35348283762082e-256	0.819499683208921	0.257	0.12	2.95654791049891e-252	no change
167	167	FBLIM1	3.55190586621913e-256	0.772667448835248	0.251	0.115	7.75878317416906e-252	no change
168	168	KHDRBS1	3.40407526497851e-255	0.423523581915746	0.633	0.422	7.43586200881905e-251	no change
169	169	HNRNPAB	2.4215122777814e-252	0.457373746582671	0.585	0.381	5.28955141958568e-248	no change
170	170	SFPQ	8.11562513278321e-252	0.380339723499745	0.723	0.513	1.77277715400516e-247	no change
171	171	PA2G4	9.66487280740893e-252	0.519896608768751	0.724	0.531	2.11119481605041e-247	no change
172	172	BTG2	1.02680296801784e-250	-0.584749368	0.834	0.832	2.24294840333817e-246	no change
173	173	TRAP1	2.9821094438328e-249	0.660182288190538	0.281	0.136	6.51411986910836e-245	no change
174	174	HSPB1	3.46035557335538e-247	-0.566429414	0.997	0.993	7.55880071443749e-243	no change
175	175	AHCY	6.7044962153024e-247	0.557437428888273	0.364	0.199	1.46453015327066e-242	no change
176	176	RXRA	1.32475017572601e-244	0.666673849831068	0.288	0.144	2.8937842838559e-240	no change
177	177	C10orf99	3.77418078449298e-244	0.396946808023829	0.619	0.418	8.24432050564647e-240	no change

178	178	ANXA10	1.08156322718671e-243	0.856760912507011	0.304	0.163	2.36256671346665e-239	no change
179	179	FAM120A	6.40753691938514e-243	0.475932766989156	0.421	0.245	1.39966236467049e-238	no change
180	180	EGFL6	1.98253007538985e-242	0.998444867012386	0.168	0.062	4.33063869668159e-238	no change
181	181	MACROD2	2.03957603217686e-241	0.685926988071187	0.231	0.104	4.45524988468714e-237	no change
182	182	EDF1	5.37474316094326e-241	0.260313476778535	0.943	0.851	1.17405889607645e-236	no change
183	183	HNRNPR	8.96343529237721e-241	0.457824086183157	0.579	0.38	1.95797280526688e-236	no change
184	184	PSMB7	1.00768809289946e-240	0.449117772765693	0.629	0.435	2.20119387012957e-236	no change
185	185	EIF3F	5.24393932630282e-240	0.386595923498392	0.838	0.697	1.14548610643759e-235	no change
186	186	USP31	2.17626325285753e-238	0.73246740595666	0.235	0.107	4.75382944954199e-234	no change
187	187	RPL30	3.37030114274273e-238	0.136984669197441	1	0.999	7.36208581620723e-234	no change
188	188	CCT7	5.696202399752e-238	0.469785883206377	0.507	0.318	1.24427845220183e-233	no change
189	189	SHH	2.0658014243165e-237	0.680313291854782	0.234	0.105	4.51253663127696e-233	no change
190	190	MCM3	3.86339608662736e-237	0.861200965511233	0.23	0.105	8.43920241162881e-233	no change

191	191	SHMT2	9.74971332618065e-237	0.709522155453407	0.271	0.134	2.1297273789709e-232	no change
192	192	DKC1	1.23721367443437e-236	0.601080940262074	0.32	0.169	2.70256955043444e-232	no change
193	193	EGR1	1.6031570445089e-236	-0.596659173	0.547	0.663	3.50193624802525e-232	no change
194	194	HNRNPD	7.15964744129724e-236	0.496524859092301	0.534	0.35	1.56395338707697e-231	no change
195	195	SMC4	9.87118145540663e-232	0.793664992205757	0.232	0.108	2.15626087711902e-227	no change
196	196	XRCC5	1.89874807572384e-230	0.403764628577366	0.63	0.428	4.14762529661116e-226	no change
197	197	GDF15	4.30635188973821e-230	-0.849895944	0.573	0.64	9.40679506794415e-226	no change
198	198	GPX1	7.9456277660928e-230	-0.411987781	0.91	0.873	1.73564292922531e-225	no change
199	199	RPS25	1.51053112431406e-229	0.214669616084308	1	0.998	3.29960418795162e-225	no change
200	200	ILF3	4.35627232493065e-229	0.496611700820171	0.434	0.26	9.51584126657851e-225	no change
201	201	UBA52	9.48046231828543e-229	0.158673371305881	1	0.996	2.07091218880627e-224	no change
202	202	PPP1R15A	2.8070873834731e-228	-0.622769757	0.735	0.773	6.13180168045865e-224	no change
203	203	S100A10	1.29813063950895e-227	-0.502276424	0.72	0.814	2.83563656894335e-223	no change

204	204	CCT3	6.9124717238945e-226	0.397242967657835	0.68	0.484	1.50996032336751e-221	no change
205	205	NDUFA8	3.74847408739176e-225	0.468862153059164	0.501	0.321	8.18816679649856e-221	no change
206	206	FAM60A	1.89529377304868e-224	0.501159877090602	0.392	0.227	4.14007971784754e-220	no change
207	207	CCNB1IP1	2.76625951343194e-224	0.545099133621994	0.282	0.142	6.04261728114074e-220	no change
208	208	BMP3	6.11630522662589e-224	0.796332908690367	0.189	0.079	1.33604571370416e-219	no change
209	209	MSI2	2.0159344108479e-223	0.483541032705851	0.516	0.336	4.40360712705614e-219	no change
210	210	PGLS	4.64562050656186e-223	0.39463062721244	0.656	0.467	1.01478934345337e-218	no change
211	211	CAT	3.18425596209259e-222	0.42411201313457	0.408	0.235	6.95568872359506e-218	no change
212	212	RPS28	1.99958261081928e-221	0.140053378045748	1	1	4.36788825507364e-217	no change
213	213	NPM3	5.41417625618991e-221	0.479142352028759	0.421	0.249	1.18267266140212e-216	no change
214	214	EIF2S3	6.07613104587922e-218	0.277179784564946	0.617	0.41	1.32727006566186e-213	no change
215	215	LY6D	3.78343850962634e-217	0.458364516670089	0.585	0.414	8.26454308042779e-213	no change
216	216	EIF3K	1.17010725363603e-216	0.26598667983371	0.926	0.821	2.55598228484254e-212	no change

217	217	THOC3	6.3008166348395e-216	0.592575554728567	0.281	0.144	1.37635038571434e-211	no change
218	218	TMEM261	8.92964920648929e-216	0.373013261782648	0.567	0.371	1.95059257266552e-211	no change
219	219	RPS27L	1.05465288914277e-214	-0.486602555	0.854	0.825	2.30378377104347e-210	no change
220	220	UTRN	1.93152039335332e-214	0.819352101034736	0.198	0.088	4.21921314724099e-210	no change
221	221	CXCL17	3.12134356259964e-214	-1.40396531	0.226	0.343	6.81826287814266e-210	down
222	222	ITGA6	3.16558471107781e-214	0.450420837277644	0.344	0.191	6.91490324287836e-210	no change
223	223	APEX1	6.24359459198218e-214	0.411200904041247	0.595	0.406	1.36385080267259e-209	no change
224	224	SRPK1	1.42602087867833e-213	0.46912614892013	0.425	0.258	3.11500000738494e-209	no change
225	225	WDR72	1.43896053483086e-213	0.611405277986524	0.305	0.168	3.14326539228453e-209	no change
226	226	SRD5A2	1.6238424085921e-212	1.5220609085208	0.107	0.03	3.54712135732858e-208	up
227	227	AC090498.1	2.15755502578472e-212	0.260295166359892	0.881	0.74	4.71296319832413e-208	no change
228	228	CCT8	3.15130894742914e-212	0.361563074027738	0.644	0.445	6.88371926476422e-208	no change
229	229	PARP1	5.3595994350744e-211	0.487230004871093	0.392	0.232	1.17075090059765e-206	no change

230	230	PPM1G	2.06274978341793e-210	0.494076277723575	0.458	0.289	4.50587062689814e-206	no change
231	231	FLRT3	3.72573184354261e-210	0.742505723492864	0.245	0.123	8.13848863903447e-206	no change
232	232	MARCKSL1	4.13379006534314e-210	0.294954077589584	0.693	0.504	9.02985101873555e-206	no change
233	233	TNFRSF12A	1.00848104070819e-208	-0.707104042	0.494	0.58	2.20292598532297e-204	no change
234	234	SLC26A2	1.41698480123979e-208	0.933994605871937	0.175	0.074	3.09526159982821e-204	no change
235	235	RBBP4	1.54599287001127e-207	0.430050197725334	0.562	0.379	3.37706682525262e-203	no change
236	236	SLC7A1	2.64783233461747e-207	0.7676697154736	0.163	0.064	5.78392495173841e-203	no change
237	237	RP1-27K12.2	2.48527518516089e-206	2.41469215795144	0.069	0.011	5.42883511446546e-202	up
238	238	DECR1	5.75847852473183e-206	0.416315628000382	0.564	0.381	1.25788204894242e-201	no change
239	239	S100A13	6.86122105652071e-206	-0.713763588	0.588	0.618	1.49876512758638e-201	no change
240	240	PGK1	1.22364934140198e-205	0.353328749142616	0.756	0.575	2.67293962135848e-201	no change
241	241	EXOSC5	1.2262940520232e-205	0.610242913153054	0.272	0.141	2.67871672723948e-201	no change
242	242	FAT1	2.57523545055034e-205	0.519290327471217	0.275	0.143	5.62534431818217e-201	no change

243	243	SET	8.12717656270031e-204	0.177416785804164	0.792	0.6	1.77530044835626e-199	no change
244	244	CYP4F11	1.62118153070575e-203	0.922969967801458	0.161	0.065	3.54130893567365e-199	no change
245	245	SORD	4.83577216690201e-202	0.63331005873136	0.232	0.113	1.05632607213807e-197	no change
246	246	STT3B	6.38707462038554e-202	0.415380919948472	0.366	0.212	1.39519258007702e-197	no change
247	247	PAICS	8.09568069622839e-202	0.535804238796955	0.264	0.136	1.76842049128413e-197	no change
248	248	IGBP1	1.5932177240406e-201	0.383405675171313	0.587	0.401	3.48022479639428e-197	no change
249	249	GCLM	6.31751327130973e-201	0.563780741043016	0.305	0.169	1.3799975989849e-196	no change
250	250	DDX17	1.12795301472631e-200	0.40095550006229	0.646	0.463	2.46390056536814e-196	no change
251	251	SERBP1	1.84065162854064e-199	0.306268923515791	0.822	0.648	4.02071941738418e-195	no change
252	252	STMN1	2.34517357319607e-199	0.696002701451489	0.588	0.43	5.1227971532895e-195	no change
253	253	HOXA7	3.18306623876462e-198	0.672734253718763	0.221	0.108	6.95308989195744e-194	no change
254	254	RHOV	7.73017627538859e-198	-0.969625738	0.368	0.459	1.68857970559588e-193	no change
255	255	BIRC5	1.11347786111704e-197	1.59531278538574	0.122	0.042	2.43228103982407e-193	up

256	256	RPL34	2.98301897806277e-197	0.22479613263337	1	1	6.51610665568032e-193	no change
257	257	TSHZ2	3.07916498992036e-197	0.52414014509285	0.212	0.098	6.72612800398203e-193	no change
258	258	SNRNP40	6.93026777259749e-197	0.455958252603334	0.358	0.207	1.5138476922462e-192	no change
259	259	DDX46	7.30904551160093e-197	0.42778761194644	0.563	0.383	1.59658790155411e-192	no change
260	260	LPP	6.67439713500809e-196	0.348805614201976	0.516	0.333	1.45795531017117e-191	no change
261	261	TNPO1	2.33141231894736e-195	0.525390984944195	0.268	0.14	5.09273706950861e-191	no change
262	262	CCT4	3.05192585076017e-195	0.307513055885336	0.574	0.383	6.66662682840053e-191	no change
263	263	AGR2	4.99065254241855e-195	0.262933781869979	0.906	0.782	1.09015814136591e-190	no change
264	264	CLNS1A	8.960981934348e-195	0.333437735012565	0.637	0.443	1.95743689373898e-190	no change
265	265	HNMT	5.11390264185295e-194	0.424321722283291	0.671	0.5	1.11708089308636e-189	no change
266	266	RANBP1	1.06264076815038e-193	0.49716628884549	0.793	0.637	2.32123249394768e-189	no change
267	267	TPR	1.39797721925139e-193	0.410773509625196	0.459	0.29	3.05374143773273e-189	no change
268	268	TP63	1.40488010345441e-193	0.494646801243285	0.266	0.138	3.06882009798581e-189	no change

269	269	RCN1	2.20806417599839e-193	0.636789274676158	0.572	0.411	4.82329538605088e-189	no change
270	270	TUFM	6.92372211729395e-193	0.378209242309348	0.745	0.574	1.51241785930169e-188	no change
271	271	HNRNPU	7.63802065953166e-193	0.313157922576644	0.686	0.49	1.6684492328681e-188	no change
272	272	PTBP3	2.98904723736103e-192	0.438475325595623	0.375	0.224	6.52927478529144e-188	no change
273	273	ELF3	3.55671784531452e-192	-0.39869751	0.94	0.88	7.76929446130504e-188	no change
274	274	CRABP2	4.83616435628688e-192	-0.782937387	0.528	0.599	1.05641174198731e-187	no change
275	275	RNF39	3.89392927779994e-191	-1.348011954	0.216	0.319	8.50589911442618e-187	down
276	276	NAP1L4	1.79353360830897e-190	0.375402507091821	0.446	0.277	3.91779481399011e-186	no change
277	277	ALDH3A1	1.88179415790782e-189	1.04584772675545	0.2	0.096	4.11059115853384e-185	up
278	278	UQCRC2	2.14698338547836e-189	0.320606314034362	0.657	0.465	4.68987050723893e-185	no change
279	279	SDHA	5.31222278995987e-189	0.48969147031221	0.343	0.2	1.16040194623883e-184	no change
280	280	MRPL50	6.48299125992972e-189	0.399840692020527	0.336	0.191	1.41614461081905e-184	no change
281	281	SSRP1	8.49242939935086e-189	0.423620518175419	0.402	0.246	1.8550862779942e-184	no change

282	282	RPS12	1.06480984439801e-188	0.177859443421481	1	0.999	2.32597062410301e-184	no change
283	283	HNRNPA0	2.49162671892529e-188	0.294261243402294	0.816	0.659	5.4427094048204e-184	no change
284	284	SSB	3.0515932889298e-188	0.349708659040816	0.627	0.438	6.66590038033825e-184	no change
285	285	CCDC34	1.24358916276343e-187	0.700419858264105	0.285	0.16	2.71649616714043e-183	no change
286	286	SGK1	1.79643485490647e-187	0.429120663249197	0.379	0.23	3.92413229705769e-183	no change
287	287	CCDC14	2.04466397794912e-187	0.544777644622831	0.276	0.149	4.46636399343205e-183	no change
288	288	TARDBP	2.19426931681371e-187	0.465224793315551	0.348	0.205	4.79316189564786e-183	no change
289	289	DSTN	3.69264543891597e-187	-0.283174823	0.967	0.943	8.06621469676804e-183	no change
290	290	DDOST	4.94470960078035e-187	0.455236490160862	0.573	0.4	1.08012236519446e-182	no change
291	291	NMD3	6.80746738694594e-187	0.421182802717152	0.425	0.266	1.48702317600447e-182	no change
292	292	SLC14A1	7.63077914682414e-187	0.352055551920985	0.469	0.3	1.66686739683227e-182	no change
293	293	MCM7	2.58747661536403e-186	0.737774831504363	0.192	0.089	5.65208391860118e-182	no change
294	294	DHRS3	8.1905845461018e-186	0.32965693150422	0.54	0.359	1.78915128825048e-181	no change

295	295	PRRC2B	1.78620246943523e-185	0.589035949029375	0.195	0.09	3.90178067423432e-181	no change
296	296	SAMM50	8.90556144068884e-185	0.470989048417801	0.273	0.146	1.94533084110407e-180	no change
297	297	ATP5G2	3.51663325899782e-184	0.188357207686039	0.987	0.944	7.68173369095484e-180	no change
298	298	ZNF326	5.22099179308949e-184	0.506382878069153	0.268	0.143	1.14047344728247e-179	no change
299	299	BZW2	2.78237745181303e-183	0.349996483048284	0.464	0.294	6.07782530574038e-179	no change
300	300	TMPO	2.13850549521767e-182	0.732422725150744	0.208	0.101	4.67135140375348e-178	no change
301	301	CCT6A	9.46209310838079e-182	0.336685075619144	0.542	0.363	2.0668996185947e-177	no change
302	302	SLC38A1	2.09935027845835e-181	0.357464396727405	0.509	0.335	4.58582074826443e-177	no change
303	303	IMPDH2	4.93958483000459e-181	0.334246404182739	0.629	0.442	1.0790029102662e-176	no change
304	304	LGALS1	8.64356395348233e-181	-0.768279171	0.501	0.596	1.88810010999868e-176	no change
305	305	DLX5	5.03628096036759e-180	1.47899984125363	0.13	0.05	1.1001252129827e-175	up
306	306	NASP	7.63175213149484e-180	0.498300413035573	0.376	0.231	1.66707993560373e-175	no change
307	307	MAD2L1	2.45411379862689e-179	1.14986859077677	0.126	0.047	5.36076618172058e-175	up

308	308	ITGB3BP	6.8755277319064e-179	0.502946283001178	0.292	0.164	1.50189027775763e-174	no change
309	309	SMARCC1	1.07140400841162e-178	0.505702464927089	0.311	0.179	2.34037491597434e-174	no change
310	310	TOP2B	2.11170959851156e-178	0.427138197229649	0.381	0.233	4.61281844698866e-174	no change
311	311	HOXB-AS3	2.68116829170064e-178	1.44497209402779	0.091	0.025	5.85674401639087e-174	up
312	312	LRPPRC	6.99165953841045e-178	0.553529621363313	0.231	0.118	1.52725810957038e-173	no change
313	313	HSPD1	1.10437378657631e-177	0.305116091335452	0.738	0.561	2.41239409939728e-173	no change
314	314	OLA1	1.22201203998372e-177	0.380413986347794	0.462	0.299	2.66936310014044e-173	no change
315	315	IER3	3.70844624280361e-177	-0.561398677	0.722	0.763	8.10072997278021e-173	no change
316	316	CENPV	5.40319413235319e-177	0.8927381131931	0.123	0.044	1.18027372627123e-172	no change
317	317	SORL1	1.18270877172625e-176	0.383985743663993	0.412	0.258	2.58350904095883e-172	no change
318	318	FAU	1.96729833913469e-176	0.129533859565302	1	1	4.29736649200583e-172	no change
319	319	MGP	2.58200513279342e-176	-1.917135247	0.179	0.283	5.64013201207395e-172	down
320	320	NQO1	6.82636802312811e-176	0.39099231913378	0.548	0.381	1.49115183097211e-171	no change

321	321	CELF1	7.18512828594569e-176	0.380006774816197	0.394	0.243	1.56951942278198e-171	no change
322	322	SERPINB5	1.250738409685e-175	0.353026714182753	0.504	0.342	2.73211298211591e-171	no change
323	323	HOXB3	4.63949142446052e-175	0.921417525371217	0.13	0.049	1.01345050675916e-170	no change
324	324	KPNB1	5.98971952156506e-175	0.360867083056226	0.472	0.307	1.30839433229067e-170	no change
325	325	DGKH	1.11162319831924e-174	0.332905274665314	0.323	0.184	2.42822971440855e-170	no change
326	326	PPIE	4.63711949244719e-174	0.471022026599427	0.301	0.172	1.01293238193016e-169	no change
327	327	RBPMS	7.2610981724946e-174	0.381131546791294	0.398	0.245	1.58611428479972e-169	no change
328	328	RBBP7	8.11676229987418e-174	0.355318508633318	0.408	0.252	1.77302555678452e-169	no change
329	329	CBX3	1.45090922669481e-173	0.376351497078293	0.738	0.573	3.16936611479215e-169	no change
330	330	MRPS2	2.24600735092924e-173	0.572024238436259	0.22	0.111	4.90617845736984e-169	no change
331	331	LTA4H	3.90345335669409e-173	0.354258706535187	0.345	0.203	8.52670351236256e-169	no change
332	332	YDJC	5.23922884583346e-173	0.48131608176529	0.317	0.186	1.14445714908386e-168	no change
333	333	C19orf48	1.47948497761535e-172	0.528058826253912	0.266	0.146	3.23178698510297e-168	no change

334	334	MPPED2	2.16083739343019e-172	0.783863580109008	0.126	0.047	4.7201332022089e-168	no change
335	335	CHMP1B	2.60639619871061e-172	0.362464865795292	0.631	0.453	5.69341185646347e-168	no change
336	336	RPAIN	5.80758144548147e-172	0.36546378105098	0.398	0.245	1.26860809095097e-167	no change
337	337	TMEM47	9.71833606231064e-172	0.476065000140503	0.283	0.161	2.12287332945114e-167	no change
338	338	EIF3E	1.19568990462516e-171	0.239033815244692	0.938	0.871	2.6118650276632e-167	no change
339	339	HNRNPA1L2	1.87815230410756e-171	0.64448242978626	0.146	0.06	4.10263589309255e-167	no change
340	340	CYP1B1	2.09378538985525e-171	0.649931006419178	0.184	0.087	4.5736648055998e-167	no change
341	341	SNRPA	2.13733851497317e-171	0.451621552820656	0.33	0.195	4.6688022521074e-167	no change
342	342	DCAF7	3.21024115829064e-171	0.515729408438925	0.311	0.183	7.01245078617008e-167	no change
343	343	GABPB1-AS1	4.51784194028301e-171	0.482782595010919	0.268	0.148	9.8687739343542e-167	no change
344	344	S100A11	4.52842663863583e-171	-0.185536725	0.999	0.998	9.8918951494361e-167	no change
345	345	CDCA7	1.7038617520251e-170	1.72132918159461	0.08	0.02	3.72191561112362e-166	up
346	346	HSPA9	1.71693533395867e-170	0.307651335693901	0.503	0.329	3.75047354349932e-166	no change

347	347	DCTPP1	3.14565059904386e-170	0.468480009657564	0.441	0.29	6.87135916855142e-166	no change
348	348	PSMA5	1.81984865182265e-169	0.340613443597901	0.575	0.399	3.9752773950414e-165	no change
349	349	GOT2	2.69093821276048e-169	0.396606371437605	0.413	0.263	5.87808543195399e-165	no change
350	350	CKS1B	4.17975058618323e-169	0.599520578299325	0.466	0.321	9.13024718045864e-165	no change
351	351	ANXA2	4.76794426790353e-169	-0.335630192	0.922	0.915	1.04150974588085e-164	no change
352	352	RPL39L	4.88779083275828e-169	1.20352622173597	0.093	0.028	1.06768902950772e-164	up
353	353	RAD50	4.91912281266851e-169	0.46540343141296	0.307	0.177	1.07453318719931e-164	no change
354	354	LARS	5.10135612279492e-169	0.383550670478094	0.272	0.148	1.11434023146332e-164	no change
355	355	KRCC1	5.35131380977784e-169	0.396062191741849	0.324	0.19	1.16894098860787e-164	no change
356	356	SPCS1	5.3854797029286e-169	-0.495443089	0.853	0.808	1.17640418630772e-164	no change
357	357	SMC1A	5.48327875512323e-169	0.61475836694795	0.211	0.107	1.19776741126912e-164	no change
358	358	CBX5	8.32424119042723e-169	0.540700232675406	0.252	0.137	1.81834724563692e-164	no change
359	359	EPB41L4A-AS1	9.04347281528523e-169	0.32607667268424	0.457	0.292	1.97545620177091e-164	no change

360	360	MAP3K5	9.19878507961083e-169	0.493074169708581	0.262	0.144	2.00938261279019e-164	no change
361	361	LMNB1	9.32220944808467e-169	1.23131070968861	0.086	0.024	2.03634343183961e-164	up
362	362	MEX3A	1.22885653807013e-168	1.26729031526949	0.096	0.03	2.68431422176039e-164	up
363	363	DBI	1.00271831561778e-167	-0.342823279	0.916	0.879	2.19033788863549e-163	no change
364	364	KLF6	1.21019104561876e-167	-0.350874685	0.87	0.867	2.64354132004963e-163	no change
365	365	WNT7B	2.11197864777423e-167	0.470053424962302	0.285	0.161	4.61340615819803e-163	no change
366	366	NIPSNAP3A	5.3508425481464e-167	0.543272781209508	0.228	0.119	1.1688380462171e-162	no change
367	367	NKAIN2	7.31368353116243e-167	1.31389880453933	0.089	0.026	1.59760103054712e-162	up
368	368	ABCC3	8.11278741956974e-167	0.427555752844688	0.314	0.183	1.77215728393081e-162	no change
369	369	EIF1	3.16406603999449e-166	-0.29861451	0.996	0.988	6.91158585776396e-162	no change
370	370	NOB1	5.40509567118157e-166	0.372165188736694	0.343	0.204	1.1806890984129e-161	no change
371	371	CAPN12	9.98457912666815e-166	0.666024446260015	0.153	0.066	2.18103146442939e-161	no change
372	372	SLC20A1	1.67462442665065e-165	-0.710423919	0.5	0.558	3.65804959757568e-161	no change

373	373	TOP2A	3.17678499991196e-165	1.64728275905305	0.105	0.037	6.93936915380768e-161	up
374	374	SNRPF	4.18007826145454e-165	0.318840226027945	0.856	0.717	9.13096295432129e-161	no change
375	375	SEPP1	6.9600409289182e-165	-0.872859567	0.48	0.533	1.52035134051289e-160	no change
376	376	HSDL2	1.34531007404834e-164	0.516155295426363	0.248	0.134	2.9386953257512e-160	no change
377	377	NOC2L	2.43903016045269e-164	0.438272277997696	0.386	0.244	5.32781748249285e-160	no change
378	378	CAPRIN1	3.74577640604979e-164	0.33175428012607	0.382	0.234	8.18227398137515e-160	no change
379	379	COLCA2	6.78542258975811e-164	1.13255386610018	0.103	0.035	1.48220771050676e-159	up
380	380	TK1	9.39522554419091e-164	1.6548448068383	0.125	0.051	2.05229306787306e-159	up
381	381	TMEM5	9.6527505791944e-164	0.504826949569372	0.227	0.119	2.10854683651922e-159	no change
382	382	FXR1	1.66086806755548e-163	0.320687545897305	0.368	0.222	3.62800020676819e-159	no change
383	383	FUBP1	1.93475214110124e-163	0.393209850411867	0.292	0.166	4.22627257702154e-159	no change
384	384	OSTF1	2.60031783649802e-163	0.324103186086218	0.489	0.324	5.68013428204627e-159	no change
385	385	RCC2	2.7656838540939e-163	0.693763058054325	0.223	0.12	6.04135981088272e-159	no change

386	386	EIF3H	3.65676065804229e-163	0.280374688646531	0.833	0.699	7.98782798142757e-159	no change
387	387	ZNF331	4.77942230499194e-163	0.697902733313935	0.248	0.139	1.04401700830244e-158	no change
388	388	ST13	7.06346516647582e-163	0.242005148948022	0.876	0.747	1.54294333096498e-158	no change
389	389	SCAF11	7.77609883384086e-163	0.321962502577418	0.586	0.409	1.6986110292642e-158	no change
390	390	RPLP2	1.45291345691673e-162	0.121583611702801	1	1	3.1737441552889e-158	no change
391	391	IPO5	1.46281182631706e-162	0.447410902730071	0.21	0.106	3.19536615340699e-158	no change
392	392	IFITM3	2.22125920944006e-162	-0.88506085	0.657	0.642	4.85211861710087e-158	no change
393	393	MRPL4	4.67145533945293e-162	0.393805116118775	0.498	0.339	1.0204327043501e-157	no change
394	394	SQSTM1	1.02302587203175e-161	-0.47484689	0.82	0.786	2.23469771486615e-157	no change
395	395	S100A2	1.13369789476181e-161	0.10637803627196	0.889	0.801	2.4764496813177e-157	no change
396	396	IMMT	3.37610727379468e-161	0.48355754419129	0.235	0.125	7.37476872887711e-157	no change
397	397	PAWR	5.01757926552625e-161	0.414899041799432	0.381	0.241	1.09604001476155e-156	no change
398	398	MCC	6.74912046793842e-161	0.479287502652687	0.207	0.105	1.47427787501647e-156	no change

399	399	LDOC1	5.4837249809369e-160	0.739900246802347	0.127	0.05	1.19786488483586e-155	no change
400	400	TRMT10C	7.16739272523578e-160	0.35788817810265	0.304	0.176	1.5656452669005e-155	no change
401	401	SMARCA5	1.66769425331515e-159	0.355793205199254	0.329	0.196	3.64291132694162e-155	no change
402	402	ATP5G3	1.73661539899589e-159	0.262541331480439	0.929	0.832	3.79346267756663e-155	no change
403	403	HOXB8	1.81574463731327e-159	0.721283562265278	0.191	0.095	3.9663125857471e-155	no change
404	404	PPHLN1	9.21461227664188e-159	0.364132828274444	0.423	0.271	2.01283990570965e-154	no change
405	405	GSTO1	1.70184224487194e-158	0.336426313813548	0.64	0.477	3.71750419969826e-154	no change
406	406	SLC25A3	1.71159943276086e-158	0.235846926298487	0.9	0.778	3.73881780092281e-154	no change
407	407	CORO2A	2.3853211761102e-158	0.621466086390624	0.154	0.068	5.21049557709512e-154	no change
408	408	ATRX	2.94022722076447e-158	0.300011255632882	0.489	0.324	6.4226323410379e-154	no change
409	409	RUVBL1	3.09489329741139e-158	0.534257542498035	0.214	0.111	6.76048491886544e-154	no change
410	410	NOP58	3.73010096631776e-158	0.290343993155317	0.336	0.198	8.14803255082451e-154	no change
411	411	VPS51	4.38774338711591e-158	0.362651754794765	0.308	0.179	9.58458665481598e-154	no change

412	412	NUDT21	6.7340631795175e-158	0.335391583444151	0.413	0.263	1.4709887609338e-153	no change
413	413	RPL7L1	7.59704610436084e-158	0.372689427544678	0.325	0.194	1.65949875103658e-153	no change
414	414	HOXA5	1.99937063752572e-157	0.656313636875065	0.178	0.085	4.36742522061118e-153	no change
415	415	DCTN2	2.12227601190069e-157	0.322492930576459	0.478	0.316	4.63589972039586e-153	no change
416	416	DHX9	3.03784343077183e-157	0.420864755550985	0.27	0.152	6.63586519017798e-153	no change
417	417	FNBP1L	3.52466458130324e-157	0.512400084314949	0.305	0.183	7.69927731139879e-153	no change
418	418	PSMD2	6.34190188017199e-157	0.345475723930351	0.409	0.261	1.38532504670477e-152	no change
419	419	UBE2E3	9.78835174145751e-157	0.282486626183977	0.674	0.493	2.13816755440398e-152	no change
420	420	CNN2	1.02922999289417e-156	0.329847272495014	0.468	0.311	2.24824999647803e-152	no change
421	421	SOX15	1.29389107729828e-156	0.421452583891347	0.215	0.11	2.82637566925037e-152	no change
422	422	DUSP1	3.39370445382778e-156	-0.619825237	0.735	0.794	7.4132080089414e-152	no change
423	423	FGFR3	5.05664186278849e-156	0.341608035652551	0.324	0.192	1.10457284850752e-151	no change
424	424	PSD3	8.09240524724248e-156	0.516371781855463	0.18	0.086	1.76770500220765e-151	no change

425	425	DCAF13	8.55152864191224e-156	0.349419634664028	0.348	0.211	1.86799591653931e-151	no change
426	426	ZFAND2A	2.35745028040522e-155	-1.221160693	0.321	0.412	5.14961439251715e-151	down
427	427	MAGOH	2.71611515985408e-155	0.32756370340905	0.557	0.386	5.93308195518526e-151	no change
428	428	TTC3	3.49512533890491e-155	0.296343529896576	0.495	0.331	7.63475179030388e-151	no change
429	429	GOLGB1	4.15135970929626e-155	0.357011884300416	0.369	0.229	9.06823014898676e-151	no change
430	430	RFC1	4.56120325453829e-155	0.482864391197466	0.321	0.195	9.96349238921343e-151	no change
431	431	RANBP2	4.65860458460012e-155	0.466584276596791	0.231	0.123	1.01762558546005e-150	no change
432	432	TANC2	5.92461541629264e-155	0.573631654193247	0.198	0.1	1.29417299153496e-150	no change
433	433	SLC44A3	1.03052763365076e-154	0.359949264200099	0.346	0.211	2.25108456294672e-150	no change
434	434	CCT2	1.12232358521158e-154	0.167590554662329	0.596	0.411	2.45160363953617e-150	no change
435	435	SERTAD1	2.55305240006876e-154	-0.810273582	0.463	0.529	5.5768876627102e-150	no change
436	436	XRCC6	2.85746933904577e-154	0.288203708030117	0.553	0.379	6.24185602421158e-150	no change
437	437	POLD2	4.81036444205695e-154	0.403605728738236	0.376	0.238	1.05077600872292e-149	no change

438	438	NSD1	5.06877371464244e-154	0.561949763226905	0.186	0.092	1.10722293022649e-149	no change
439	439	AR	7.28557288454141e-154	0.480644561956135	0.197	0.1	1.59146054089923e-149	no change
440	440	PTGES2	8.83173364125071e-154	0.412422627905671	0.286	0.165	1.92920389659481e-149	no change
441	441	BPTF	1.06050767952682e-153	0.37885842179259	0.387	0.247	2.31657297515839e-149	no change
442	442	KRTCAP3	1.97048623431745e-153	0.247101846077414	0.537	0.355	4.30433013024304e-149	no change
443	443	SBNO1	2.3576412818544e-153	0.413291527624642	0.261	0.146	5.15003161608274e-149	no change
444	444	FBXO21	2.5149229433373e-153	0.36705774397636	0.288	0.167	5.493597677426e-149	no change
445	445	HOXB4	2.6888005580603e-153	0.716437830840154	0.145	0.063	5.87341593902691e-149	no change
446	446	XPO1	4.12473213149207e-153	0.351846551481403	0.297	0.174	9.01006486803127e-149	no change
447	447	SNRNP200	9.5031347364483e-153	0.426117625393364	0.261	0.147	2.07586475182977e-148	no change
448	448	CBWD5	1.07844729422686e-152	0.387061819455719	0.229	0.121	2.35576026950914e-148	no change
449	449	BCLAF1	1.1203952355226e-152	0.288923949609838	0.422	0.27	2.44739135247556e-148	no change
450	450	ZFAND1	1.14788323439107e-152	0.270879639218536	0.389	0.24	2.50743613720385e-148	no change

451	451	TAF7	1.36728606293429e-152	0.278356675698127	0.515	0.349	2.98669967587366e-148	no change
452	452	HADHB	2.1021576985331e-152	0.315118474774252	0.553	0.386	4.59195327667571e-148	no change
453	453	TPTEP1	2.42282668241809e-152	0.748604668891101	0.126	0.051	5.29242260507407e-148	no change
454	454	GPX4	2.71238033720163e-152	-0.296133843	0.952	0.907	5.92492360858324e-148	no change
455	455	TCERG1	3.27307995106876e-152	0.39891030320914	0.245	0.134	7.1497158451146e-148	no change
456	456	ACTL6A	3.47240569873728e-152	0.39717356247837	0.305	0.181	7.5851230083217e-148	no change
457	457	BAZ1B	4.3740588255473e-152	0.520320850805145	0.236	0.129	9.55469409852553e-148	no change
458	458	KRT13	5.63664728192455e-152	0.355851023563417	0.732	0.617	1.2312692322636e-147	no change
459	459	TBL1XR1	7.46035810406734e-152	0.242900381192486	0.493	0.328	1.62964062425247e-147	no change
460	460	PRPF4B	8.71796420931511e-152	0.332121410459211	0.358	0.22	1.90435210188279e-147	no change
461	461	THRAP3	1.86256477334743e-151	0.309159580165589	0.429	0.278	4.06858649090012e-147	no change
462	462	RAD23B	2.39416523768488e-151	0.312326359585209	0.434	0.282	5.22981454519885e-147	no change
463	463	TNNT3	2.99891854559389e-151	0.318158840177596	0.336	0.203	6.55083767099529e-147	no change

464	464	EIF3I	4.25547636974791e-151	0.28942561108137	0.703	0.528	9.29566258207733e-147	no change
465	465	SH2D4A	5.15590778418839e-151	0.422721876154036	0.278	0.159	1.12625649637811e-146	no change
466	466	DHRS4	1.04436375294046e-150	0.520408772101907	0.212	0.112	2.28130818192315e-146	no change
467	467	LIMA1	1.11304143871168e-150	0.215337586641848	0.561	0.382	2.4313277187218e-146	no change
468	468	MDH1	1.15998449889989e-150	0.319149362170129	0.551	0.386	2.53387013939692e-146	no change
469	469	UQCRHL	1.43073491850249e-150	0.53079561968375	0.169	0.08	3.12529735597684e-146	no change
470	470	PPDPF	1.44147315866941e-150	-0.350991943	0.988	0.95	3.14875396779746e-146	no change
471	471	RPL21	2.86032613784907e-150	0.188815268786681	1	0.999	6.2480964155175e-146	no change
472	472	MTCL1	4.16275226780904e-150	0.761664562331867	0.141	0.062	9.09311605380206e-146	no change
473	473	MYO1B	9.10963176019642e-150	0.684995020556207	0.113	0.043	1.98990796169731e-145	no change
474	474	SMC2	1.80548170497952e-149	0.909142592659649	0.119	0.047	3.94389423635725e-145	no change
475	475	MAFF	4.03477637486388e-149	-0.640088004	0.622	0.621	8.81356551325265e-145	no change
476	476	PTPN13	4.44615914829249e-149	0.466441173716915	0.232	0.127	9.71219004353011e-145	no change

477	477	ITM2C	5.05184658226789e-149	0.24862650504389	0.509	0.342	1.1035253674306e-144	no change
478	478	PEG10	1.14560635338322e-148	1.61724550612213	0.07	0.018	2.5024625183303e-144	up
479	479	PRPF8	1.20897641129965e-148	0.391242675121128	0.233	0.126	2.64088807284295e-144	no change
480	480	TALDO1	2.06072183567047e-148	0.338311154000575	0.714	0.564	4.50144077783857e-144	no change
481	481	SMARCA4	2.48468262438086e-148	0.437374401612445	0.31	0.188	5.42754072469755e-144	no change
482	482	SH3BGRL3	3.35928375411744e-148	-0.269141288	0.955	0.935	7.33801943249414e-144	no change
483	483	LY96	3.77658951201366e-148	-1.583028777	0.07	0.145	8.24958213004263e-144	down
484	484	PRSS22	4.84567498513385e-148	-0.891534213	0.316	0.398	1.05848924375264e-143	no change
485	485	DEK	5.01074340135803e-148	0.418408656470634	0.588	0.437	1.09454678859265e-143	no change
486	486	NOP16	5.86977998178359e-148	0.325639985890721	0.314	0.185	1.28219473922081e-143	no change
487	487	HSD17B4	8.51786926602181e-148	0.348387975535848	0.313	0.186	1.8606433624698e-143	no change
488	488	VDAC3	8.55949857428009e-148	0.293407443071988	0.456	0.301	1.86973686856574e-143	no change
489	489	AC004556.1	8.78671362187586e-148	0.396297631669341	0.142	0.061	1.91936972356256e-143	no change

490	490	PPIH	1.56145315361571e-147	0.423453868674527	0.251	0.14	3.41083826875817e-143	no change
491	491	MRPL41	3.54020681824396e-147	0.322280464334702	0.68	0.516	7.73322777377212e-143	no change
492	492	BRD3	5.36423691440057e-147	0.515354150252953	0.181	0.09	1.17176391158166e-142	no change
493	493	MBP	5.9025458368029e-147	0.485366228576739	0.211	0.111	1.28935211259123e-142	no change
494	494	ADGRG6	6.99098684026533e-147	0.457571909101611	0.215	0.113	1.52711116538756e-142	no change
495	495	HNRNPUL1	1.9971419101766e-146	0.303873309378322	0.35	0.217	4.36255678858976e-142	no change
496	496	NHP2	1.03844165673642e-145	0.251868881983436	0.83	0.678	2.26837195497503e-141	no change
497	497	NUDT5	1.13412427218033e-145	0.298737115561395	0.412	0.267	2.47738106015072e-141	no change
498	498	ELAVL1	1.13912459723867e-145	0.304954675549691	0.486	0.33	2.48830377020815e-141	no change
499	499	GXYLT1	2.30398976072861e-145	0.718415564389053	0.161	0.077	5.03283523333557e-141	no change
500	500	TRUB2	3.32855912249382e-145	0.617445239049188	0.169	0.082	7.27090454717549e-141	no change
501	501	GTF2I	4.7144864850557e-145	0.280798123715666	0.65	0.471	1.02983242779557e-140	no change
502	502	NUDT2	2.02872840675741e-144	0.367118800316661	0.274	0.159	4.4315543317209e-140	no change

503	503	NSA2	3.66900659045269e-144	0.239085976903899	0.676	0.491	8.01457799618486e-140	no change
504	504	SSBP4	3.81077875925845e-144	0.37710671	0.29	0.171	8.32426512172415e-140	no change
505	505	SFN	3.99099962627306e-144	-0.426090573	0.748	0.766	8.71793958363088e-140	no change
506	506	PAK2	4.44930767929079e-144	0.352242054016746	0.379	0.244	9.71906769464281e-140	no change
507	507	GCLC	5.24559862476884e-144	0.362375705828705	0.453	0.309	1.1458485635945e-139	no change
508	508	CERS6	6.26211567455555e-144	0.417243583737955	0.273	0.16	1.36789654794992e-139	no change
509	509	WASF2	7.1702785644059e-144	0.268679138286699	0.531	0.363	1.56627564960882e-139	no change
510	510	P3H2	7.33752226497963e-144	1.03779768164897	0.095	0.033	1.60280836356215e-139	up
511	511	MECOM	8.43820834810985e-144	0.264945037200945	0.438	0.287	1.84324223156112e-139	no change
512	512	RAB14	8.61764621096416e-144	0.335759072817564	0.411	0.269	1.88243863832301e-139	no change
513	513	MPHOSPH10	1.15648221218505e-143	0.481584174448217	0.229	0.126	2.52621974429701e-139	no change
514	514	PTBP1	1.84831940900363e-143	0.323428415508928	0.415	0.271	4.03746891702753e-139	no change
515	515	NDUFS3	2.32483821238221e-143	0.299602938365642	0.526	0.362	5.07837659112769e-139	no change

516	516	HNRNPF	2.34877897740532e-143	0.281717327165848	0.757	0.589	5.13067279824417e-139	no change
517	517	CDC123	4.26431536995948e-143	0.288385477413498	0.374	0.235	9.31497049413949e-139	no change
518	518	CBX1	4.37364157375394e-143	0.391949757190807	0.303	0.182	9.55378265370811e-139	no change
519	519	EIF2A	7.66545059091507e-143	0.256500996127558	0.48	0.318	1.67444102707949e-138	no change
520	520	PPIL3	9.58241703411408e-143	0.263554969194458	0.377	0.236	2.09318317693188e-138	no change
521	521	IGFBP2	1.05654447288548e-142	0.202252801974666	0.654	0.496	2.30791574657104e-138	no change
522	522	CTC-524C5.2	1.71338653753095e-142	0.666675355072389	0.14	0.062	3.7427215525826e-138	no change
523	523	THOC2	2.47645419087489e-142	0.389884090595201	0.376	0.244	5.4095665345471e-138	no change
524	524	AMIGO2	3.52895924147803e-142	0.451419825303423	0.299	0.182	7.70865856708461e-138	no change
525	525	TYMS	5.08334346013152e-142	1.3532232188781	0.123	0.053	1.11040554543113e-137	up
526	526	CYP4F8	5.66563942931981e-142	-1.261188017	0.105	0.188	1.23760227694062e-137	down
527	527	CBWD3	8.88927205763e-142	0.585329297030095	0.147	0.067	1.9417725882687e-137	no change
528	528	PFN1	1.49158118891058e-141	0.180554145706518	0.991	0.957	3.25820994905628e-137	no change

529	529	VAMP8	1.58208688563487e-141	-0.364780041	0.882	0.808	3.45591059298081e-137	no change
530	530	WDR3	1.90725288638488e-141	0.744380074273689	0.127	0.054	4.16620320501913e-137	no change
531	531	LINC00958	3.48221366312766e-141	0.550475816730242	0.226	0.126	7.60654752573607e-137	no change
532	532	OXR1	3.78737359337857e-141	0.321647625390361	0.346	0.215	8.27313887737615e-137	no change
533	533	SUPT16H	9.11545204276483e-141	0.401286293580519	0.228	0.125	1.99117934422155e-136	no change
534	534	MT1F	9.79341294381051e-141	0.402827483654625	0.233	0.128	2.13927312344597e-136	no change
535	535	NIFK	1.13753735025636e-140	0.265919017639375	0.369	0.229	2.48483658789999e-136	no change
536	536	NMRK1	1.16361036567368e-140	0.386978389667978	0.287	0.17	2.54179048277759e-136	no change
537	537	LYPD6B	5.66114901284787e-140	0.315039617974237	0.429	0.285	1.23662139036649e-135	no change
538	538	BHLHE40	7.18589510463721e-140	-0.62517578	0.471	0.532	1.56968692665695e-135	no change
539	539	GJB2	7.60590851284774e-140	0.663205623303868	0.192	0.102	1.66143465554646e-135	no change
540	540	HES1	8.3671433865222e-140	-0.536496325	0.869	0.849	1.82771880135191e-135	no change
541	541	CNOT7	1.15804299600141e-139	0.260470871749412	0.481	0.324	2.52962912046547e-135	no change

542	542	SNHG7	1.2257926762429e-139	0.3280648080192	0.406	0.266	2.677621521985e-135	no change
543	543	SEL1L3	1.40327629203118e-139	0.730236464979563	0.163	0.08	3.06531673231292e-135	no change
544	544	BTG1	1.80247541359503e-139	-0.417365111	0.838	0.828	3.93732729345697e-135	no change
545	545	TUBA1B	4.8121184361578e-139	0.746384056043326	0.794	0.668	1.05115915119431e-134	no change
546	546	STAG2	1.35520741070846e-138	0.344455166970233	0.294	0.176	2.96031506795155e-134	no change
547	547	QARS	1.62139998341533e-138	0.274477065998298	0.417	0.271	3.54178612377244e-134	no change
548	548	WDR34	2.36390121080893e-138	0.625213747891352	0.22	0.123	5.16370580489102e-134	no change
549	549	PDCD6	2.78626950042205e-138	0.311343864923526	0.695	0.526	6.08632709672194e-134	no change
550	550	CD2AP	2.90857386722299e-138	0.343400983392958	0.411	0.271	6.35348875556189e-134	no change
551	551	SEPT10	5.96994105973093e-138	0.283627801086293	0.331	0.204	1.30407392508762e-133	no change
552	552	POLE3	7.10603066023288e-138	0.414647390193866	0.234	0.132	1.55224133742127e-133	no change
553	553	NFATC2IP	7.22416378244151e-138	0.371902809378791	0.272	0.16	1.57804633663652e-133	no change
554	554	PRPF38B	7.4646309650967e-138	0.245074623409631	0.519	0.354	1.63057398801572e-133	no change

555	555	DAZAP1	9.11380714004618e-138	0.35973302428927	0.339	0.214	1.99082003167169e-133	no change
556	556	MRPS27	9.77205816952788e-138	0.431771330313822	0.179	0.09	2.13460838655167e-133	no change
557	557	HOXB5	1.18344271087083e-137	1.11257362104458	0.084	0.027	2.58511225762624e-133	up
558	558	PCNA	5.97398696199795e-137	0.482735570240042	0.349	0.223	1.30495771197883e-132	no change
559	559	ATXN10	6.98369953543456e-137	0.268794490133281	0.47	0.317	1.52551932652033e-132	no change
560	560	SNCG	7.12550099940368e-137	-0.506465733	0.912	0.858	1.55649443830974e-132	no change
561	561	REXO4	9.23569833859716e-137	0.535415482911138	0.18	0.092	2.01744594508316e-132	no change
562	562	ZRANB2	1.10295622592772e-136	0.309621415921099	0.386	0.25	2.40929757991651e-132	no change
563	563	PKP4	1.20340164954692e-136	0.597563686475401	0.145	0.067	2.6287105632703e-132	no change
564	564	UBE2E1	2.7455945644529e-136	0.335461278733128	0.416	0.276	5.99747676659091e-132	no change
565	565	MRPL3	3.10602502048564e-136	0.270348906653349	0.423	0.277	6.78480105474882e-132	no change
566	566	KDM1A	3.32021395358001e-136	0.421816912817078	0.233	0.131	7.25267536020017e-132	no change
567	567	SATB1	3.49040370359468e-136	0.506656722208715	0.204	0.11	7.62443785013222e-132	no change

568	568	MAT2B	4.17029370891073e-136	0.300979708746942	0.368	0.234	9.10958957774461e-132	no change
569	569	EBPL	4.23580736869973e-136	0.277878943988104	0.595	0.43	9.25269761618768e-132	no change
570	570	CANX	6.08423396707738e-136	0.259854966124964	0.644	0.475	1.32904006776838e-131	no change
571	571	XRN2	7.36354401477226e-136	0.271890875149842	0.453	0.303	1.60849255458685e-131	no change
572	572	CYB5B	8.42376230885599e-136	0.270623894499383	0.51	0.352	1.8400866387465e-131	no change
573	573	CSTF3	1.33814621738244e-135	0.451538385934033	0.286	0.175	2.9230465972502e-131	no change
574	574	SMARCC2	1.58176733228695e-135	0.445198496637239	0.244	0.139	3.45521256064761e-131	no change
575	575	SEMA6A	1.7275328949383e-135	0.483282860733443	0.148	0.069	3.77362285570323e-131	no change
576	576	KIF21A	2.32086766153706e-135	0.695392120188461	0.143	0.066	5.06970331986154e-131	no change
577	577	ABHD2	2.39689889259811e-135	0.352905483658018	0.221	0.121	5.23578594099131e-131	no change
578	578	CNBP	3.69971722234788e-135	0.258099316909914	0.712	0.545	8.08166230049671e-131	no change
579	579	TOMM40	4.80948762310941e-135	0.34391612305668	0.365	0.235	1.05058447639202e-130	no change
580	580	GMDS	5.24724374845783e-135	0.502895258534153	0.221	0.123	1.14620792441313e-130	no change

581	581	PRPF6	5.45139080157386e-135	0.273701685395706	0.384	0.247	1.19080180669579e-130	no change
582	582	LARP1	6.76121832247135e-135	0.35310739438943	0.276	0.163	1.47692053036064e-130	no change
583	583	MCM5	1.61178835697415e-134	0.85927963897854	0.116	0.048	3.52079048697432e-130	no change
584	584	IMMP1L	2.33461432379224e-134	0.352147317898455	0.334	0.21	5.09973152889176e-130	no change
585	585	ZWINT	3.10783250139436e-134	1.14579483994556	0.106	0.042	6.78874931604584e-130	up
586	586	LCOR	3.25125230510205e-134	0.367964961717254	0.257	0.15	7.10203553526492e-130	no change
587	587	AZGP1	3.44692337686086e-134	0.849225071348553	0.099	0.038	7.52945942441486e-130	no change
588	588	FEN1	4.39403780749654e-134	1.23490028139027	0.089	0.031	9.59833618669543e-130	up
589	589	TP53	5.50237647311393e-134	0.421129943407786	0.224	0.126	1.20193911678701e-129	no change
590	590	PRPF19	8.7835327887313e-134	0.44169710378072	0.228	0.129	1.91867490237046e-129	no change
591	591	COQ4	9.17125073250463e-134	0.261414206480091	0.378	0.242	2.00336801000831e-129	no change
592	592	BYSL	1.00226663749533e-133	0.683904412880313	0.127	0.056	2.18935124294479e-129	no change
593	593	LETMD1	1.16265142840374e-133	0.329758761774688	0.309	0.189	2.53969578020514e-129	no change

594	594	TRIM56	1.57827131124184e-133	0.374679918646343	0.265	0.157	3.44757585227668e-129	no change
595	595	HP1BP3	2.90587672067535e-133	0.360100624813345	0.533	0.385	6.34759710864324e-129	no change
596	596	RSF1	3.06473407002388e-133	0.288760258635069	0.451	0.304	6.69460510256016e-129	no change
597	597	ATP8B1	4.38766248679228e-133	0.258513713370195	0.442	0.293	9.58440993614906e-129	no change
598	598	RPS29	4.71071900780893e-133	0.152482109322622	1	0.997	1.02900946006578e-128	no change
599	599	ABCE1	5.60127794361783e-133	0.296125511037202	0.261	0.151	1.22354315400388e-128	no change
600	600	PSIP1	1.10774608466557e-132	0.321207299726005	0.181	0.092	2.41976054734347e-128	no change
601	601	MRPS34	1.40992262119351e-132	0.301922890683144	0.676	0.514	3.07983497373511e-128	no change
602	602	CSE1L	1.56181691503043e-132	0.398774554425456	0.178	0.091	3.41163286919248e-128	no change
603	603	SDHB	2.54258371832125e-132	0.341441632229657	0.516	0.367	5.55401987430095e-128	no change
604	604	PHIP	3.98113595420774e-132	0.253929696240349	0.331	0.204	8.69639337837139e-128	no change
605	605	RBM25	4.93749737194441e-132	0.233336994097045	0.583	0.416	1.07854692592754e-127	no change
606	606	SEPT9	6.42641891083135e-132	0.266064217142722	0.442	0.294	1.403786946882e-127	no change

607	607	NDUFV1	6.99501737410643e-132	0.300295415183417	0.543	0.385	1.52799159519981e-127	no change
608	608	URM1	9.2679283120451e-132	0.340285626536714	0.283	0.169	2.02448626048313e-127	no change
609	609	SRSF1	9.97823809455643e-132	0.301221274	0.478	0.331	2.17964632937491e-127	no change
610	610	ARF4	9.98749860674338e-132	-0.444531242	0.748	0.73	2.18166919565702e-127	no change
611	611	ASF1B	1.18409674199297e-131	1.53486694963884	0.071	0.021	2.58654092320944e-127	up
612	612	CEP57	1.19083004618924e-131	0.42326733757412	0.231	0.131	2.60124915289577e-127	no change
613	613	FAIM	1.38430753033914e-131	0.440088130000518	0.287	0.177	3.02388136927282e-127	no change
614	614	KYNU	1.53298106797252e-131	0.416419282179803	0.326	0.209	3.34864384487917e-127	no change
615	615	VPS35	2.10943996061138e-131	0.252521753984249	0.628	0.459	4.6078606499595e-127	no change
616	616	S100A16	2.92289155776738e-131	-0.45681674	0.628	0.673	6.38476431878706e-127	no change
617	617	NIPSNAP1	3.4881855644637e-131	0.490982023886373	0.228	0.13	7.61959254701451e-127	no change
618	618	DNMT1	3.57043365937309e-131	0.598431085264154	0.199	0.109	7.79925528553458e-127	no change
619	619	MDK	3.57336710490738e-131	0.281967878658861	0.819	0.688	7.80566310395969e-127	no change

620	620	TUSC3	4.39460943429791e-131	0.261544872550312	0.498	0.344	9.59958484828036e-127	no change
621	621	CDC20	4.76636734055539e-131	1.51888836145117	0.079	0.026	1.04116528187092e-126	up
622	622	KARS	5.40769735187534e-131	0.267293627834196	0.377	0.242	1.18125740954365e-126	no change
623	623	IRS1	5.54432843955699e-131	0.373317829835075	0.232	0.132	1.21110310433683e-126	no change
624	624	LNPEP	5.59888059053908e-131	0.267733062074905	0.214	0.116	1.22301947619736e-126	no change
625	625	ZBTB44	6.90992146257981e-131	0.334137709870256	0.292	0.178	1.50940324428593e-126	no change
626	626	DHX15	9.72311881535824e-131	0.261138036665105	0.358	0.228	2.12391807402685e-126	no change
627	627	ADGRV1	1.16471943824956e-130	0.502578060245164	0.187	0.099	2.54421314091234e-126	no change
628	628	APOL2	2.25111179588542e-130	0.405888091308412	0.314	0.197	4.91732860693212e-126	no change
629	629	VARS	2.84087534484061e-130	0.570229889685975	0.152	0.073	6.20560810326984e-126	no change
630	630	EIF4G2	3.32242203242714e-130	0.239757930013012	0.857	0.73	7.25749868763385e-126	no change
631	631	SULT1E1	5.51898815286173e-130	-1.194642526	0.086	0.161	1.20556777211112e-125	down
632	632	C9orf78	5.67798455168125e-130	0.272975020618151	0.359	0.229	1.24029894546925e-125	no change

633	633	SRRM1	5.81448664003313e-130	0.25104657805696	0.75	0.583	1.27011646164884e-125	no change
634	634	DEFB1	6.43441773832537e-130	-0.886172662	0.248	0.337	1.40553421075979e-125	no change
635	635	HNRNPA3	7.21976088997324e-130	0.245873873046778	0.812	0.66	1.57708456880575e-125	no change
636	636	ASS1	7.55169939938818e-130	0.220446672299231	0.37	0.242	1.64959321680235e-125	no change
637	637	NDUFA10	9.78595895793854e-130	0.239481087	0.528	0.364	2.13764487477209e-125	no change
638	638	RP11-395G23.3	1.53835297394156e-129	0.517167562171129	0.187	0.098	3.36037823627795e-125	no change
639	639	MIS18A	1.59023428430694e-129	0.551903489	0.139	0.065	3.47370777064009e-125	no change
640	640	GART	2.04826306868005e-129	0.385700340952666	0.204	0.111	4.4742258472247e-125	no change
641	641	NOA1	2.55475467744535e-129	0.552056669958066	0.17	0.087	5.58060611741162e-125	no change
642	642	UQCRB	2.72409398409496e-129	0.202295487901123	0.964	0.913	5.95051089885703e-125	no change
643	643	MAST4	4.18360897911042e-129	0.270346109454902	0.588	0.43	9.1386754539688e-125	no change
644	644	CHD3	4.58221915192718e-129	0.271411364130226	0.288	0.174	1.00093995154697e-124	no change
645	645	PUS7L	6.4226514099805e-129	0.396531419481776	0.262	0.156	1.40296397399614e-124	no change

646	646	DDB1	6.72997591465229e-129	0.307458784765803	0.327	0.205	1.47009593879665e-124	no change
647	647	NOP56	1.16026202761189e-128	0.260651484530542	0.399	0.262	2.53447637311542e-124	no change
648	648	MZT2A	1.16341551069943e-128	0.250129743881959	0.82	0.666	2.54136484157183e-124	no change
649	649	RPA1	2.18536597761622e-128	0.569865728560007	0.149	0.073	4.77371344150486e-124	no change
650	650	G6PD	6.96138492213269e-128	0.363655626829722	0.283	0.172	1.52064492239067e-123	no change
651	651	MTAP	7.23994466547228e-128	0.586253314411684	0.141	0.066	1.58149351272576e-123	no change
652	652	TUBA1C	1.99188741963013e-127	0.316954908996712	0.626	0.463	4.35107887944005e-123	no change
653	653	SMARCD1	2.28462688518359e-127	0.421394327513148	0.212	0.117	4.99053896799503e-123	no change
654	654	NUDT3	3.0205909758093e-127	0.354148414273417	0.299	0.186	6.59817892755783e-123	no change
655	655	CTD-2008L17.2	3.12875286835484e-127	1.44822146814087	0.053	0.011	6.83444776563432e-123	up
656	656	PPIF	3.57375662327401e-127	0.327516484403423	0.252	0.147	7.80651396787976e-123	no change
657	657	BRIX1	3.9462488334484e-127	0.302455949607064	0.297	0.182	8.62018595178469e-123	no change
658	658	GAREM1	4.11917391756326e-127	0.448911831443294	0.205	0.114	8.99792350552518e-123	no change

659	659	NSMCE3	4.15431940882479e-127	0.492853553805673	0.168	0.086	9.07469531663687e-123	no change
660	660	VBP1	6.84835087717613e-127	0.250936943113071	0.441	0.296	1.49595376561035e-122	no change
661	661	SAFB	8.70468821929033e-127	0.379785632481833	0.248	0.144	1.90145209462178e-122	no change
662	662	CDK1	1.24683122789781e-126	1.41966600305375	0.089	0.033	2.72357813421998e-122	up
663	663	SF3B1	1.48156843561349e-126	0.227998698461072	0.51	0.35	3.23633809075411e-122	no change
664	664	ALG8	1.82022012160878e-126	0.390220016448694	0.219	0.123	3.97608883364222e-122	no change
665	665	RERE	2.45519131012673e-126	0.330177726460811	0.329	0.209	5.36311989784083e-122	no change
666	666	CS	2.48481301109197e-126	0.412045242545713	0.237	0.138	5.4278255414293e-122	no change
667	667	ANAPC5	2.76125930251167e-126	0.244446743120232	0.419	0.277	6.03169482040649e-122	no change
668	668	SNRPA1	4.50830910935922e-126	0.317778342190687	0.387	0.256	9.84795041848428e-122	no change
669	669	POLR2H	4.5944449683033e-126	0.307446085665462	0.424	0.288	1.00361055887617e-121	no change
670	670	DFFA	4.95051540570238e-126	0.422210879827294	0.283	0.175	1.08139058522163e-121	no change
671	671	GTF2F1	4.96284501650779e-126	0.346305278066525	0.256	0.15	1.08408386540596e-121	no change

672	672	ELP2	5.59254322564721e-126	0.326024895059785	0.238	0.137	1.22163514221038e-121	no change
673	673	HNRNPA2B1	7.17746896742762e-126	0.231618801563281	0.915	0.814	1.56784632124489e-121	no change
674	674	WDR12	7.83990242214449e-126	0.661502344005144	0.125	0.056	1.71254828509324e-121	no change
675	675	FKBP11	1.17626491536273e-125	0.127655803582444	0.286	0.169	2.56943308111835e-121	no change
676	676	MKI67	1.38063514732175e-125	1.99356721826567	0.053	0.012	3.01585941580962e-121	up
677	677	GLTSCR2	1.43022448362568e-125	0.247926893559159	0.864	0.779	3.12418236203193e-121	no change
678	678	MYCBP	1.7879200438547e-125	0.316430368906762	0.253	0.149	3.9055325437962e-121	no change
679	679	ABHD17B	1.92122005383879e-125	0.494987480419649	0.175	0.091	4.19671308560546e-121	no change
680	680	GANAB	2.28447140591104e-125	0.354176955297283	0.287	0.176	4.99019933907207e-121	no change
681	681	PRPF40A	2.33481792017034e-125	0.266134058148423	0.503	0.351	5.1001762648201e-121	no change
682	682	PMPCA	2.6519010895519e-125	0.449768758687482	0.171	0.088	5.79281274001716e-121	no change
683	683	RBM14	3.11839502819081e-125	0.414504526629575	0.194	0.104	6.81182209958001e-121	no change
684	684	IARS	3.34401252551356e-125	0.54504671120742	0.151	0.074	7.30466096073183e-121	no change

685	685	PDHA1	3.4927955806651e-125	0.336452978506821	0.29	0.177	7.62966266640484e-121	no change
686	686	ADH5	3.63900446839407e-125	0.256186542352985	0.433	0.29	7.94904136076001e-121	no change
687	687	PES1	4.6085477299881e-125	0.504921487876753	0.19	0.103	1.0066911661386e-120	no change
688	688	DDX54	6.79264960982204e-125	0.35507031467615	0.253	0.149	1.48378638076953e-120	no change
689	689	U2SURP	7.56620033020035e-125	0.25186078663788	0.445	0.302	1.65276080012896e-120	no change
690	690	SNRPD1	8.53968382834478e-125	0.282339246979652	0.831	0.683	1.86540853546363e-120	no change
691	691	CXADR	1.01190598875033e-124	0.20616994301323	0.773	0.599	2.21040744182621e-120	no change
692	692	PPARG	1.13153122162493e-124	0.255929237970893	0.535	0.377	2.47171680051749e-120	no change
693	693	ATP2A2	1.34197141421711e-124	0.260266169847584	0.371	0.24	2.93140235721585e-120	no change
694	694	MRPS21	1.48244074937915e-124	0.213371262822034	0.835	0.693	3.23824357294382e-120	no change
695	695	FNBP4	1.78863558552469e-124	0.268067369049927	0.302	0.185	3.90709557302013e-120	no change
696	696	PPP6C	1.98770268342696e-124	0.283641343068291	0.257	0.151	4.34193774167785e-120	no change
697	697	TRIM44	2.19904613614577e-124	0.298056893867848	0.267	0.16	4.80359637979681e-120	no change

698	698	EBNA1BP2	2.36709463636293e-124	0.299613467784026	0.417	0.28	5.17068152367118e-120	no change
699	699	NT5C	2.36782206010943e-124	0.256976156690065	0.536	0.382	5.17227050810304e-120	no change
700	700	IFITM2	2.81906217485595e-124	-1.229016979	0.163	0.242	6.15795941475533e-120	down
701	701	RPA2	4.94805179368112e-124	0.388747470932171	0.227	0.13	1.0808524338117e-119	no change
702	702	ZNF207	6.25737217110533e-124	0.247314329378431	0.579	0.416	1.36686037705625e-119	no change
703	703	TIMM10	6.96227146011783e-124	0.259983061247371	0.57	0.415	1.52083857774814e-119	no change
704	704	HUWE1	1.16440956942351e-123	0.378266255928135	0.237	0.138	2.54353626344872e-119	no change
705	705	SMC3	1.59098084394043e-123	0.414926090807587	0.231	0.134	3.47533855550347e-119	no change
706	706	PPIA	2.04404499796937e-123	0.19635861041368	0.99	0.957	4.4650118935643e-119	no change
707	707	PTDSS1	2.14292030763448e-123	0.408013303948028	0.191	0.103	4.68099511999675e-119	no change
708	708	PAX8	2.44376063416814e-123	1.03990771258174	0.082	0.029	5.33815072927688e-119	up
709	709	MAOA	2.69727267524933e-123	0.230865601371185	0.527	0.371	5.89192243181465e-119	no change
710	710	GPX2	2.93547060228614e-123	0.256910508553723	0.895	0.801	6.41224198363385e-119	no change

711	711	TTC17	4.3603024934349e-123	0.323488452075192	0.228	0.13	9.5246447666592e-119	no change
712	712	CHCHD10	5.36985406119936e-123	0.260554370201215	0.822	0.687	1.17299092112839e-118	no change
713	713	STAT6	6.90172824317236e-123	0.304820816881206	0.289	0.177	1.50761351743857e-118	no change
714	714	ZC3H8	7.71268769321071e-123	0.419426277252599	0.183	0.097	1.68475949970495e-118	no change
715	715	MAGED1	8.20236776939241e-123	0.409334411626412	0.224	0.129	1.79172521554608e-118	no change
716	716	GSPT1	8.89575708119402e-123	0.236444064070269	0.525	0.369	1.94318917681602e-118	no change
717	717	FGFR2	9.63738117213464e-123	0.677977115985326	0.104	0.042	2.10518954324109e-118	no change
718	718	S100A9	1.00865156653624e-122	-0.921713378	0.373	0.467	2.20329848194176e-118	no change
719	719	CPSF3L	1.2517117568494e-122	0.3379364845421	0.291	0.18	2.73423916166183e-118	no change
720	720	TRIM28	1.44840632006394e-122	0.350666712704573	0.258	0.153	3.16389876554767e-118	no change
721	721	CENPK	2.15980792627768e-122	1.22249642703969	0.077	0.026	4.71788443416097e-118	up
722	722	MID1	2.23863574213937e-122	0.396963543372017	0.254	0.152	4.89007591512923e-118	no change
723	723	RCC1	2.74603779228662e-122	0.477379600461818	0.198	0.109	5.99844495347088e-118	no change

724	724	LYAR	3.49755126519969e-122	0.443485043367758	0.187	0.101	7.64005098370221e-118	no change
725	725	SAFB2	4.51949795717342e-122	0.270521467908304	0.288	0.175	9.87239133764962e-118	no change
726	726	PABPN1	5.83695548839022e-122	0.273950851808617	0.453	0.311	1.27502455688396e-117	no change
727	727	DHFR	7.99729015980894e-122	0.872654518198832	0.12	0.055	1.74692806250866e-117	no change
728	728	SMAGP	1.53914771691206e-121	0.424123684554799	0.295	0.187	3.3621142728227e-117	no change
729	729	GMCL1	1.78599179830548e-121	0.282828554521699	0.288	0.177	3.90132048421849e-117	no change
730	730	BTN3A2	2.54532496859463e-121	0.426877376480401	0.205	0.115	5.56000786139811e-117	no change
731	731	B3GALNT1	3.13796698861524e-121	0.462985644893708	0.227	0.132	6.85457508993112e-117	no change
732	732	RAB6A	3.6944170158199e-121	0.269976810811513	0.516	0.363	8.070084529357e-117	no change
733	733	SMC5	3.79877081106689e-121	0.35767965078787	0.187	0.1	8.29803495969452e-117	no change
734	734	EPS8	4.07264177255041e-121	0.235864288375246	0.448	0.304	8.89627868795912e-117	no change
735	735	AC004791.2	4.26250577905741e-121	1.91027446556507	0.05	0.011	9.31101762377302e-117	up
736	736	IL1A	4.6858659834173e-121	-1.33711973	0.09	0.162	1.02358056541768e-116	down

737	737	ZNF581		5.09189222821112e-121	0.403994765409535	0.223	0.128	1.11227293833044e-116	no change
738	738	SRSF10		6.58657375099199e-121	0.287325020063294	0.548	0.397	1.43877117016669e-116	no change
739	739	USP34		9.84148665156607e-121	0.265727057885576	0.287	0.177	2.14977434416809e-116	no change
740	740		6-Mar	1.07404521982673e-120	0.260842372405376	0.391	0.259	2.3461443781895e-116	no change
741	741	RNF44		1.09294548195668e-120	0.353491275990379	0.183	0.098	2.38743011078617e-116	no change
742	742	PPP1CC		1.09792537978816e-120	0.23857992714523	0.612	0.447	2.39830819960925e-116	no change
743	743	UHRF1		2.74661937464843e-120	1.30376000239868	0.067	0.02	5.99971536198202e-116	up
744	744	CAPS		2.8706486804098e-120	-0.443743876	0.514	0.578	6.27064497748716e-116	no change
745	745	C5orf24		3.01389022634555e-120	0.269132554316522	0.275	0.166	6.58354181042922e-116	no change
746	746	SOD3		4.17682037175795e-120	-1.755667087	0.123	0.199	9.12384642006806e-116	down
747	747	CSNK2B		5.24311557878834e-120	0.253795817984005	0.681	0.517	1.14530616703052e-115	no change
748	748	CDC26		5.52716262885455e-120	0.234679081240792	0.48	0.332	1.20735340464699e-115	no change
749	749	PRRC2A		5.92205848303356e-120	0.328236509366695	0.219	0.124	1.29361445503385e-115	no change

750	750	NADSYN1	6.27378866037382e-120	0.36395140525924	0.298	0.187	1.37044639497206e-115	no change
751	751	MIB2	6.74783946981639e-120	0.598964964821117	0.208	0.12	1.47399805378669e-115	no change
752	752	LSM4	7.36252963617926e-120	0.340410052225115	0.697	0.545	1.608270973727e-115	no change
753	753	SRM	8.85078205701288e-120	0.283621040407387	0.419	0.285	1.93336483253389e-115	no change
754	754	RPL22L1	8.92251102885796e-120	0.233927786174102	0.585	0.427	1.94903330914373e-115	no change
755	755	MRTO4	1.0340710826877e-119	0.32378287913593	0.247	0.146	2.25882487302301e-115	no change
756	756	METAP1	1.13901541998764e-119	0.308688266669157	0.281	0.172	2.48806528342099e-115	no change
757	757	TPD52L1	1.19869490768127e-119	0.305733794185739	0.233	0.136	2.61842915633897e-115	no change
758	758	PDCD2	1.26008848766804e-119	0.270240687169292	0.411	0.276	2.75253729246207e-115	no change
759	759	HMGB3	1.28795652804659e-119	0.280704226823514	0.386	0.258	2.81341223986497e-115	no change
760	760	KCTD16	1.34246454372832e-119	1.99272095475072	0.044	0.008	2.93247954932014e-115	up
761	761	TOMM22	1.58395937210195e-119	0.284660391269612	0.671	0.508	3.46000085241951e-115	no change
762	762	SREK1	1.6230009241082e-119	0.250928409236051	0.39	0.258	3.54528321862194e-115	no change

763	763	MIS18BP1	2.44828266991359e-119	0.530729535899462	0.14	0.068	5.34802866415924e-115	no change
764	764	RNASEH2A	2.53361735097625e-119	0.511734742173225	0.179	0.097	5.53443374147252e-115	no change
765	765	AES	3.31021079779718e-119	0.228107072060831	0.747	0.585	7.23082446670815e-115	no change
766	766	CEBPZ	3.55362876153341e-119	0.220596180251505	0.313	0.196	7.76254666669357e-115	no change
767	767	DNAJC9	3.60732275157632e-119	0.608676850576216	0.176	0.095	7.87983581854331e-115	no change
768	768	C12orf10	3.79900296317624e-119	0.285453395402848	0.387	0.257	8.29854207276218e-115	no change
769	769	PRDX4	4.32283670529172e-119	0.267538944413234	0.505	0.36	9.44280449903924e-115	no change
770	770	EIF4A1	5.35887974571754e-119	0.171903295539786	0.752	0.583	1.17059369165454e-114	no change
771	771	TUG1	1.04041934626167e-118	0.283721758970606	0.289	0.179	2.27269201997399e-114	no change
772	772	WDR1	1.38023684177531e-118	0.24849641557772	0.493	0.345	3.01498935717398e-114	no change
773	773	HNRNPU-AS1	1.6385579090304e-118	0.338553332599979	0.191	0.104	3.57926589648601e-114	no change
774	774	ANP32A	1.71581427814555e-118	0.242944882508675	0.494	0.343	3.74802470918114e-114	no change
775	775	APOL1	1.94391901778991e-118	0.180404161596972	0.559	0.403	4.24629670246028e-114	no change

776	776	ADSS	1.98259208971424e-118	0.183862886435049	0.447	0.303	4.33077416077179e-114	no change
777	777	HPRT1	2.17654543342232e-118	0.235570384849932	0.335	0.215	4.75444584476771e-114	no change
778	778	DPM2	2.26920628450597e-118	0.372259588073217	0.286	0.178	4.95685420787483e-114	no change
779	779	CPNE3	2.39997541578849e-118	0.241589243890803	0.435	0.296	5.24250629824837e-114	no change
780	780	GMPR2	4.38724530371582e-118	0.387870014524635	0.267	0.164	9.58349864143684e-114	no change
781	781	BHMT	4.39106317409141e-118	0.818213667403623	0.197	0.115	9.59183839748527e-114	no change
782	782	LINC00665	5.04336034216337e-118	0.394199696023448	0.224	0.131	1.10167163314217e-113	no change
783	783	DDX18	8.6794529226559e-118	0.242599856793565	0.588	0.427	1.89593969642496e-113	no change
784	784	RMI2	8.98004379007446e-118	0.580390049968403	0.145	0.072	1.96160076550386e-113	no change
785	785	SLC38A4	1.20995556589587e-117	1.11619357363091	0.076	0.026	2.64302693814294e-113	up
786	786	LMO7	1.79215511879423e-117	0.279286337415005	0.371	0.249	3.91478364149412e-113	no change
787	787	MLEC	2.05115722070376e-117	0.265857245988681	0.431	0.295	4.48054783290529e-113	no change
788	788	MRPS25	3.19481602945016e-117	0.277618812160265	0.367	0.242	6.97875613473092e-113	no change

789	789	TMEM64	3.32664433643816e-117	0.663998314476157	0.151	0.078	7.26672188851551e-113	no change
790	790	ECSIT	4.11424442286415e-117	0.313639787578193	0.271	0.165	8.98715551730444e-113	no change
791	791	SCAMP1	5.48174808904211e-117	0.278517366922702	0.281	0.174	1.19743305257036e-112	no change
792	792	MRPL16	5.50221984054921e-117	0.319957618597912	0.336	0.217	1.20190490196957e-112	no change
793	793	ADAM10	6.36081629103626e-117	0.20490921268175	0.486	0.332	1.38945671061396e-112	no change
794	794	SRSF4	6.50792244294714e-117	0.245500190430152	0.476	0.33	1.42159057843737e-112	no change
795	795	RCN2	7.52619668406476e-117	0.229841108423281	0.674	0.506	1.64402240366711e-112	no change
796	796	SMYD3	1.20030524199114e-116	0.34438870026304	0.339	0.222	2.62194677060544e-112	no change
797	797	HMGN2	1.32372676761442e-116	0.38488642987275	0.85	0.711	2.89154875117693e-112	no change
798	798	SNRPD3	1.34487152466038e-116	0.273953299919369	0.654	0.491	2.93773735846813e-112	no change
799	799	MEMO1	1.36951293150667e-116	0.442434928610158	0.158	0.081	2.99156404758316e-112	no change
800	800	AURKB	1.42767384223941e-116	1.68156276187767	0.055	0.014	3.11861074098777e-112	up
801	801	HERPUD1	1.45540767490747e-116	-0.598983491	0.64	0.634	3.17919252506787e-112	no change

802	802	DHX29	1.81865411877435e-116	0.283005190366086	0.289	0.179	3.97266805705069e-112	no change
803	803	UBE2C	1.83732886176135e-116	1.20326276109642	0.153	0.082	4.01346116563149e-112	up
804	804	SNX5	2.07029044326248e-116	0.269764687091776	0.436	0.298	4.52234244426256e-112	no change
805	805	IK	3.25417452238523e-116	0.23860189151615	0.405	0.268	7.10841882669829e-112	no change
806	806	CENPF	3.69593104589513e-116	1.10648779028999	0.087	0.033	8.07339177665331e-112	up
807	807	BOD1	4.68294999704534e-116	0.287452516964505	0.327	0.21	1.02294359735458e-111	no change
808	808	PFDN5	5.3573268914377e-116	0.131033284751233	0.991	0.974	1.17025448616565e-111	no change
809	809	ABT1	5.73738360523778e-116	0.33875719767748	0.284	0.177	1.25327407472814e-111	no change
810	810	KANK1	6.1377045725596e-116	0.353062241673463	0.249	0.15	1.34072018682992e-111	no change
811	811	UQCRFS1	7.16328318687633e-116	0.23382166818286	0.746	0.584	1.56474757934127e-111	no change
812	812	CRIM1	8.64643756722787e-116	0.36668291535184	0.249	0.15	1.88872782218526e-111	no change
813	813	SF3B3	9.56592585478584e-116	0.380908060707315	0.174	0.093	2.08958084371942e-111	no change
814	814	CNPY2	1.45292362381027e-115	0.284179925276804	0.674	0.521	3.17376636385114e-111	no change

815	815	PM20D1	1.55380555823627e-115	-1.284451189	0.106	0.178	3.39413286141131e-111	down
816	816	C9orf142	1.5629373353658e-115	0.283186584348425	0.392	0.263	3.41408031537305e-111	no change
817	817	RNPS1	1.61417175401799e-115	0.240662561125441	0.598	0.438	3.5259967794769e-111	no change
818	818	PDCL	2.99817917520618e-115	0.548078413897615	0.111	0.049	6.54922259032038e-111	no change
819	819	TNFRSF21	3.05188855878195e-115	0.22258138955417	0.686	0.519	6.6665453678033e-111	no change
820	820	NDUFA7	3.09641050839895e-115	0.282484773304233	0.368	0.244	6.76379911454667e-111	no change
821	821	NET1	3.21711612686338e-115	0.189587827580169	0.514	0.359	7.02746846752037e-111	no change
822	822	CETN3	3.97742573867614e-115	0.210358362363465	0.311	0.194	8.68828878356416e-111	no change
823	823	SWI5	4.18034747492336e-115	0.250488820262492	0.358	0.234	9.13155102422258e-111	no change
824	824	ALYREF	4.22384097833007e-115	0.569908100797262	0.179	0.099	9.2265582330642e-111	no change
825	825	TPX2	4.86464710812934e-115	1.38024521286434	0.062	0.018	1.06263351429977e-110	up
826	826	HSPA1B	4.86582545024357e-115	-0.465595581	0.706	0.727	1.06289091135121e-110	no change
827	827	AIM1	4.88984520302363e-115	0.316898045076542	0.235	0.139	1.06813778614848e-110	no change

828	828	UNC5B	5.64781698813274e-115	0.407857371160273	0.208	0.119	1.23370914288772e-110	no change
829	829	SERPINB11	5.82973308242055e-115	1.34320496179366	0.055	0.014	1.27344689452395e-110	up
830	830	TTC19	5.94730441442984e-115	0.234685169907717	0.324	0.206	1.29912917628805e-110	no change
831	831	ZNF148	7.02852990352481e-115	0.345743882204127	0.23	0.135	1.53531207212596e-110	no change
832	832	ADGRF1	1.21424262352834e-114	0.277337078355047	0.326	0.211	2.6523915868353e-110	no change
833	833	VDAC2	1.28755957971435e-114	0.230793398056295	0.726	0.568	2.81254514592802e-110	no change
834	834	APIP	1.87328448842888e-114	0.290894083805474	0.272	0.167	4.09200263652404e-110	no change
835	835	RTCB	1.99226448189847e-114	0.278751751991373	0.326	0.209	4.35190253425901e-110	no change
836	836	PLCG2	2.2941560829452e-114	0.545001133248529	0.344	0.232	5.01135454758549e-110	no change
837	837	ZFR	2.296684947688e-114	0.308750995104353	0.23	0.135	5.01687859972967e-110	no change
838	838	PTTG1	2.30794980487373e-114	0.927039571750866	0.187	0.107	5.04148555376618e-110	no change
839	839	G3BP1	2.4716910344674e-114	0.218799148767309	0.396	0.265	5.39916189569059e-110	no change
840	840	FOSB	2.84929617915773e-114	-0.312919364	0.747	0.805	6.22400257375215e-110	no change

841	841	TMEM69	3.88186985666526e-114	0.324030717478269	0.289	0.182	8.4795565148996e-110	no change
842	842	PSMB4	4.5278067233923e-114	0.198189042424756	0.471	0.324	9.89054100657813e-110	no change
843	843	ZNF22	5.27097839512592e-114	0.311867540337317	0.219	0.127	1.15139252063131e-109	no change
844	844	TIMP2	5.4301915167736e-114	-1.476955539	0.061	0.123	1.18617103492403e-109	down
845	845	NDUFB5	5.75064502556365e-114	0.221408235880373	0.668	0.505	1.25617089938412e-109	no change
846	846	STARD7	5.7702621479339e-114	0.261411477205141	0.3	0.189	1.26045606359468e-109	no change
847	847	EWSR1	5.95423854017202e-114	0.229548831203102	0.469	0.324	1.30064386671518e-109	no change
848	848	CASC15	1.52982125004066e-113	0.86361064475371	0.079	0.028	3.34174153858883e-109	no change
849	849	PRPF4	1.60536309503239e-113	0.646184127252745	0.1	0.042	3.50675514478876e-109	no change
850	850	ERP44	2.23997368949204e-113	0.223911474369701	0.316	0.201	4.89299852732641e-109	no change
851	851	FLNA	2.59897602770747e-113	0.222460673555112	0.158	0.081	5.67720323492419e-109	no change
852	852	LDLR	2.77701336503716e-113	-0.884679123	0.203	0.285	6.06610799458718e-109	no change
853	853	FPGS	3.4488316371568e-113	0.481059430477145	0.155	0.08	7.5336278282053e-109	no change

854	854	ST3GAL5	4.92065070449262e-113	0.199279060872257	0.398	0.266	1.07486693988937e-108	no change
855	855	ARHGAP6	5.01621308341844e-113	0.517976111579086	0.122	0.057	1.09574158594192e-108	no change
856	856	ZNF552	5.48748007782284e-113	0.355679286309817	0.223	0.13	1.19868514819962e-108	no change
857	857	CCNB2	6.57244482013935e-113	1.49955711470125	0.061	0.018	1.43568484651124e-108	up
858	858	PCM1	7.83606284228882e-113	0.19414182523758	0.289	0.178	1.71170956726957e-108	no change
859	859	USP22	7.94054176749837e-113	0.269923350538625	0.32	0.204	1.73453194369234e-108	no change
860	860	GSTM3	8.68066436913975e-113	-0.433212873	0.615	0.667	1.89620432479489e-108	no change
861	861	SYF2	9.30019014445085e-113	0.216677775666615	0.713	0.547	2.03153353515384e-108	no change
862	862	DHX36	9.32993606375232e-113	0.204875830679292	0.396	0.265	2.03803123376606e-108	no change
863	863	VCP	9.47162649915892e-113	0.220685759341279	0.373	0.244	2.06898209247628e-108	no change
864	864	ISOC1	9.66339909271479e-113	0.20455645825735	0.372	0.244	2.11087289781262e-108	no change
865	865	LSM7	1.01322264829107e-112	0.229976383440037	0.852	0.719	2.21328355292702e-108	no change
866	866	HSD17B2	1.33342798557553e-112	0.36745534417606	0.44	0.329	2.91274009169118e-108	no change

867	867	DPP3	1.73918876978101e-112	0.499288329487416	0.186	0.104	3.79908394870964e-108	no change
868	868	NRXN3	1.79618343344021e-112	1.35300709217491	0.06	0.017	3.9235830920068e-108	up
869	869	MZT2B	1.92959013680035e-112	0.228522238164367	0.836	0.692	4.21499669482669e-108	no change
870	870	PPP1R12A	2.37037102558395e-112	0.239677136436774	0.339	0.219	5.17783846828558e-108	no change
871	871	MDH2	2.39538858244032e-112	0.272864718789787	0.699	0.541	5.23248681948265e-108	no change
872	872	SPIN1	2.66040441335785e-112	0.359728471383027	0.177	0.096	5.81138740053889e-108	no change
873	873	GGH	2.99633279300261e-112	0.543352004915846	0.167	0.09	6.54518935303489e-108	no change
874	874	GCC2	3.16851767018287e-112	0.234651817261025	0.357	0.233	6.92130999874746e-108	no change
875	875	LIMD2	3.64718640197157e-112	0.534515174013986	0.185	0.104	7.96691397646669e-108	no change
876	876	DPH5	3.6561147132304e-112	0.408899582378647	0.171	0.092	7.98641697958048e-108	no change
877	877	EIF3A	6.60507665942466e-112	0.209629898991288	0.555	0.398	1.44281294548472e-107	no change
878	878	SLC44A1	6.72662673515125e-112	0.164073752082111	0.36	0.235	1.46936434402644e-107	no change
879	879	HELLS	7.67311261758745e-112	0.736526826975518	0.119	0.056	1.6761147201858e-107	no change

880	880	MRPL24	9.03919043969176e-112	0.233379899125224	0.462	0.321	1.97452075964627e-107	no change
881	881	HCG18	9.73850512316618e-112	0.459577982404772	0.152	0.078	2.12727905910442e-107	no change
882	882	NFAT5	1.13275214988358e-111	0.228207152951486	0.362	0.238	2.4743837962057e-107	no change
883	883	BRD9	1.30109446533552e-111	0.372502899665588	0.24	0.145	2.84211075007892e-107	no change
884	884	SGOL1	1.31603339215683e-111	1.28338022008072	0.059	0.017	2.87474334182737e-107	up
885	885	COPG2	1.35883074489692e-111	0.63892483398643	0.117	0.054	2.96822987915282e-107	no change
886	886	FXN	1.53792195385527e-111	0.619836515991641	0.129	0.062	3.35943671600146e-107	no change
887	887	DST	1.95855696464282e-111	0.202781303514074	0.409	0.275	4.27827183356578e-107	no change
888	888	HSD17B10	2.53520310444118e-111	0.211304136280486	0.598	0.436	5.53789766134132e-107	no change
889	889	BRI3	2.75985792717974e-111	-0.663223144	0.605	0.584	6.02863365613143e-107	no change
890	890	EXOC7	4.10979114991356e-111	0.250062148827109	0.415	0.283	8.97742778787117e-107	no change
891	891	PLCB1	4.77464355581628e-111	0.428511512804268	0.166	0.089	1.04297313833251e-106	no change
892	892	C8orf4	5.93002157392101e-111	-0.817833519	0.206	0.292	1.29535391260731e-106	no change

893	893	MRPL15	6.33035749580265e-111	0.26758265169568	0.389	0.262	1.38280329138313e-106	no change
894	894	UNG	6.67468452951674e-111	0.615696901614673	0.166	0.09	1.45801808862764e-106	no change
895	895	HAT1	9.02157049088466e-111	0.254503598165417	0.28	0.173	1.97067185802885e-106	no change
896	896	MRPS10	9.72902450579985e-111	0.309082203974374	0.319	0.208	2.12520811304692e-106	no change
897	897	NME1	1.29034333550783e-110	0.282081088336141	0.685	0.536	2.8186259820833e-106	no change
898	898	PPP1R10	1.46604836698675e-110	0.170534362502002	0.346	0.224	3.20243605284586e-106	no change
899	899	SLTM	2.17152306842435e-110	0.231219395955999	0.54	0.387	4.74347499066616e-106	no change
900	900	TMEM245	2.18969407190176e-110	0.340123825662281	0.183	0.101	4.7831677306622e-106	no change
901	901	LYRM4	2.75936692278129e-110	0.218944748347858	0.381	0.254	6.02756110612345e-106	no change
902	902	MAGED2	2.90646956975961e-110	0.182174550633759	0.411	0.275	6.34889212818289e-106	no change
903	903	SURF6	4.12334737671083e-110	0.547867868038995	0.154	0.081	9.00704000968713e-106	no change
904	904	PBX1	4.95568718412681e-110	0.152172632240357	0.478	0.331	1.08252030850066e-105	no change
905	905	NOP14	6.2565079629895e-110	0.546307105064558	0.145	0.074	1.36667159943543e-105	no change

906	906	TMEM2	6.68848355063041e-110	0.34473826715459	0.194	0.109	1.46103234679971e-105	no change
907	907	MAZ	7.41980640547981e-110	0.357761265948694	0.207	0.12	1.62078251121301e-105	no change
908	908	CCAR1	7.61236621135799e-110	0.279997704521914	0.296	0.188	1.66284527520904e-105	no change
909	909	HLTF	7.67241858080592e-110	0.573988627325404	0.128	0.062	1.67596311479124e-105	no change
910	910	HOXA10	8.41524142722017e-110	0.445749433218457	0.183	0.102	1.83822533736197e-105	no change
911	911	IDI1	8.60982028593375e-110	-0.83533633	0.441	0.474	1.88072914325937e-105	no change
912	912	RC3H2	8.91238066585125e-110	0.652803819037492	0.109	0.049	1.94682043264855e-105	no change
913	913	DDI2	9.17850932220312e-110	0.413482931301085	0.199	0.115	2.00495357634205e-105	no change
914	914	GSTA4	1.01039501943308e-109	0.274051043588499	0.176	0.096	2.20710688044961e-105	no change
915	915	HDAC3	1.53113193499477e-109	0.371889670120646	0.24	0.145	3.34460459880257e-105	no change
916	916	RP11-499F3.2	1.71751552044105e-109	0.829387642642846	0.078	0.029	3.75174090285142e-105	no change
917	917	CCNB1	1.7298530751934e-109	1.13391222314229	0.087	0.035	3.77869105745246e-105	up
918	918	USP1	1.86175119278707e-109	0.398837304314877	0.189	0.107	4.06680930552408e-105	no change

919	919	APCDD1	2.00681428163617e-109	0.430181967321223	0.081	0.03	4.38368511680604e-105	no change
920	920	CAMK1D	2.04473984211722e-109	0.4155703754953	0.15	0.077	4.46652971112085e-105	no change
921	921	URI1	2.12579825387177e-109	0.272733874301281	0.326	0.213	4.64359370575749e-105	no change
922	922	POLR1C	2.74197881469763e-109	0.353971675252507	0.183	0.101	5.9895785228255e-105	no change
923	923	PRDX2	2.8033056726043e-109	0.221683311691459	0.865	0.75	6.12354091123684e-105	no change
924	924	POLR2G	3.10164297381247e-109	0.285884113141018	0.527	0.387	6.77522891199596e-105	no change
925	925	QSER1	3.17591122595008e-109	0.472475830369336	0.123	0.058	6.93746048196536e-105	no change
926	926	TROAP	6.07895602346228e-109	1.35737109609803	0.064	0.021	1.3278871537651e-104	up
927	927	PRMT1	8.3150821928466e-109	0.202219295951117	0.584	0.421	1.81634655420541e-104	no change
928	928	SUCLG1	9.14916611785111e-109	0.240873578731765	0.494	0.349	1.9985438467834e-104	no change
929	929	ATF6B	1.0058407558763e-108	0.254906972411473	0.356	0.236	2.19715854713619e-104	no change
930	930	ATF7IP	1.51451069573346e-108	0.370629782340647	0.178	0.098	3.30829716376017e-104	no change
931	931	HARS	1.73820669490479e-108	0.282773141352173	0.194	0.108	3.79693870435001e-104	no change

932	932	RAC1	1.89250484909311e-108	-0.196191585	0.983	0.961	4.13398759235898e-104	no change
933	933	TXNRD1	2.32797337707155e-108	0.419191117848185	0.164	0.088	5.0852250448751e-104	no change
934	934	PQBP1	3.14348283361574e-108	0.178402361930313	0.413	0.275	6.86662390175021e-104	no change
935	935	MTF2	3.36277797899223e-108	0.313739541017388	0.252	0.154	7.34565221731063e-104	no change
936	936	DNPH1	4.00416454333851e-108	0.256385780571973	0.532	0.389	8.74669702846864e-104	no change
937	937	NUP37	4.10079138864671e-108	0.318630915932131	0.238	0.143	8.95776870935987e-104	no change
938	938	HTATSF1	4.32372162986402e-108	0.279502992214946	0.296	0.189	9.44473752827496e-104	no change
939	939	C8orf82	4.66412168838168e-108	0.289186355024388	0.248	0.151	1.01883074161009e-103	no change
940	940	SMARCE1	6.20407948315659e-108	0.227881290097254	0.422	0.29	1.35521912230073e-103	no change
941	941	RIF1	1.04853282749494e-107	0.32969600040773	0.167	0.09	2.29041510837994e-103	no change
942	942	MRPS5	1.15926642762329e-107	0.263585501874267	0.403	0.276	2.53230158450031e-103	no change
943	943	EIF4G1	1.18798511378854e-107	0.272037586854395	0.341	0.225	2.59503468255968e-103	no change
944	944	LRRC41	1.27461386902068e-107	0.28508621284808	0.296	0.189	2.78426653548878e-103	no change

945	945	SKIV2L2	1.40790936891394e-107	0.308344290060903	0.161	0.085	3.07543722545562e-103	no change
946	946	SRSF11	1.40946952992762e-107	0.304600904028109	0.702	0.558	3.0788452411739e-103	no change
947	947	STRAP	1.4548648681321e-107	0.197633459684194	0.554	0.396	3.17800681794776e-103	no change
948	948	MCTP2	1.49559606312019e-107	0.572422875735577	0.105	0.046	3.26698004027975e-103	no change
949	949	PHF14	1.66286038343367e-107	0.286018575381024	0.471	0.339	3.6323522215725e-103	no change
950	950	GLO1	2.25804374441258e-107	0.467465436050419	0.546	0.418	4.93247075529483e-103	no change
951	951	TIMM9	2.52380574759997e-107	0.289153007817805	0.316	0.206	5.51300127505737e-103	no change
952	952	GPM6B	2.6878259358038e-107	0.56064276357861	0.12	0.057	5.87128697416982e-103	no change
953	953	GPBP1	3.2814640983908e-107	0.175228676164368	0.574	0.415	7.16803017652487e-103	no change
954	954	DCLRE1C	3.45376591494394e-107	0.220195009710199	0.22	0.129	7.54440626460354e-103	no change
955	955	SF3B2	3.9195401436464e-107	0.228641357995458	0.544	0.393	8.5618434897812e-103	no change
956	956	UGCG	3.9313147644878e-107	0.233835591181016	0.349	0.231	8.58756397154716e-103	no change
957	957	PRKCSH	5.2549666096136e-107	0.173064356200606	0.462	0.319	1.14789490620399e-102	no change

958	958	PRC1	5.34702949017989e-107	1.06939728033371	0.067	0.023	1.16800512183489e-102	up
959	959	PSMB8	5.54672192393575e-107	0.199038781017785	0.59	0.43	1.21162593706453e-102	no change
960	960	ORC6	5.86298318076457e-107	1.12196648191261	0.071	0.025	1.28071004600621e-102	up
961	961	CLSPN	1.02699012355833e-106	1.45444147056027	0.055	0.016	2.24335722590081e-102	up
962	962	GNB1	1.15850884183785e-106	0.298508985731339	0.393	0.271	2.53064671411061e-102	no change
963	963	DDX21	1.24148663343292e-106	0.176932768099567	0.583	0.427	2.71190340207087e-102	no change
964	964	ZKSCAN1	1.27660189195127e-106	0.296907255248761	0.279	0.177	2.78860917277835e-102	no change
965	965	MRPS35	1.38385584298552e-106	0.320298858131518	0.335	0.222	3.02289470341758e-102	no change
966	966	MRPL21	1.40555430005199e-106	0.225641392094683	0.521	0.374	3.07029281303358e-102	no change
967	967	C1S	2.06576869107391e-106	-1.785131063	0.051	0.107	4.51246512878184e-102	down
968	968	FIBP	2.2124633189342e-106	0.195566419782942	0.519	0.367	4.83290487387986e-102	no change
969	969	FARP1	2.27309232258237e-106	0.329134386961855	0.22	0.13	4.96534286944894e-102	no change
970	970	FARSA	2.53018132727259e-106	0.272191812401924	0.239	0.144	5.52692809129424e-102	no change

971	971	BAG6	2.74072647286976e-106	0.261974387078572	0.261	0.162	5.9868429073367e-102	no change
972	972	LEAP2	3.02551109231816e-106	-1.588339406	0.199	0.265	6.60892643005978e-102	down
973	973	THG1L	3.09136395445187e-106	0.335759611863712	0.185	0.103	6.75277542210466e-102	no change
974	974	TMEM161B-AS1	3.41320434828784e-106	0.482399372340136	0.123	0.059	7.45580357839997e-102	no change
975	975	LARP4	3.8504244663972e-106	0.403555944135086	0.198	0.115	8.41086720439805e-102	no change
976	976	KHSRP	3.86822787440793e-106	0.344425000422261	0.225	0.135	8.44975696885667e-102	no change
977	977	PXMP2	4.10104884380011e-106	0.365320326488744	0.263	0.166	8.95833109439697e-102	no change
978	978	MAGEF1	4.10202914986235e-106	0.342469914912297	0.295	0.191	8.96047247495932e-102	no change
979	979	WDR43	6.24377668183343e-106	0.127703125588473	0.317	0.202	1.36389057837969e-101	no change
980	980	PVRL1	6.37910828665037e-106	0.310650368741168	0.28	0.179	1.39345241413591e-101	no change
981	981	C9orf85	6.54977458903964e-106	0.440100361514709	0.155	0.082	1.43073276122982e-101	no change
982	982	PYGL	6.59262447736838e-106	0.131457541780526	0.26	0.159	1.44009289083635e-101	no change
983	983	CENPW	8.84613995601934e-106	0.762603406783042	0.142	0.075	1.93235081199286e-101	no change

984	984	EPRS	8.94250179110723e-106	0.340627188841566	0.203	0.118	1.95340009124946e-101	no change
985	985	CDC5L	9.01526249795449e-106	0.307117624064124	0.229	0.137	1.96929394005318e-101	no change
986	986	ZNF791	1.17519628169559e-105	0.335407541832846	0.183	0.102	2.56709875773584e-101	no change
987	987	ABI2	1.34474244120563e-105	0.235977861797226	0.285	0.18	2.93745538856958e-101	no change
988	988	INTS10	1.72348028308344e-105	0.294239231651646	0.215	0.126	3.76477033036747e-101	no change
989	989	CTNNA1	1.73469767145751e-105	0.19202807797229	0.44	0.304	3.78927359353178e-101	no change
990	990	TAF1D	2.388952662652e-105	0.174781817240386	0.515	0.364	5.21842819629704e-101	no change
991	991	SF3A3	2.40898992893832e-105	0.284738913664994	0.225	0.134	5.26219760077288e-101	no change
992	992	PDS5A	2.41653655457762e-105	0.264251030260477	0.262	0.163	5.27868244981935e-101	no change
993	993	PHF5A	2.51019645380247e-105	0.255379877661303	0.339	0.225	5.48327313368612e-101	no change
994	994	EPCAM	2.73236329225347e-105	0.206768965	0.478	0.338	5.96857437559849e-101	no change
995	995	NRDC	2.96085165933461e-105	0.275888109007409	0.267	0.168	6.46768436465051e-101	no change
996	996	TM4SF1	4.14021460099369e-105	-0.77612753	0.289	0.367	9.04388477441062e-101	no change

997	997	PODXL2	4.21623118116957e-105	0.576299684523701	0.17	0.095	9.20993539214681e-101	no change
998	998	LCN2	5.63726907091699e-105	-1.911151472	0.188	0.26	1.23140505585111e-100	down
999	999	NDUFAF4	9.72576262169586e-105	0.269760657298555	0.258	0.16	2.12449558708324e-100	no change
1000	1000	COPB1	1.02076401125236e-104	0.204747445038491	0.273	0.169	2.22975690617965e-100	no change
1001	1001	SNHG19	1.10167390514488e-104	0.248018995614325	0.267	0.167	2.40649647839847e-100	no change
1002	1002	ITGAE	1.31208869675028e-104	0.210366576503165	0.39	0.26	2.86612654918132e-100	no change
1003	1003	SNRNP25	1.71222644923468e-104	0.372758258368693	0.335	0.227	3.74018745570823e-100	no change
1004	1004	CLTB	1.82036138297555e-104	-0.473515239	0.623	0.62	3.97639740497178e-100	no change
1005	1005	ALDH3A2	1.85726295489737e-104	0.328191848986473	0.247	0.152	4.05700519867781e-100	no change
1006	1006	BAZ2B	3.04304452139399e-104	0.221485294583798	0.302	0.194	6.64722645253303e-100	no change
1007	1007	TXNDC12	4.03430840882707e-104	0.267072551300382	0.429	0.301	8.81254328824185e-100	no change
1008	1008	ARID1A	5.17104160307608e-104	0.311143881285076	0.261	0.164	1.12956232777594e-99	no change
1009	1009	RASD1	5.48996929902384e-104	0.498913984522746	0.146	0.077	1.19922889367877e-99	no change

1010	1010	LRRC47	5.84873792526482e-104	0.447332247682226	0.185	0.106	1.27759831239485e-99	no change
1011	1011	SMAD5	7.4845284242718e-104	0.234690789327817	0.266	0.166	1.63492038899793e-99	no change
1012	1012	FKBP4	8.38843571515405e-104	0.248977529707166	0.468	0.335	1.83236989761825e-99	no change
1013	1013	BIRC6	8.41984608614134e-104	0.218447136553809	0.23	0.137	1.83923117905671e-99	no change
1014	1014	PMPCB	8.42663449416876e-104	0.21450211212361	0.351	0.231	1.84071403890622e-99	no change
1015	1015	C2CD2	8.72788657059256e-104	0.491322601601397	0.14	0.072	1.90651954248024e-99	no change
1016	1016	DDX39A	9.42080162184404e-104	0.353296947654966	0.271	0.173	2.05787990627561e-99	no change
1017	1017	NBN	9.66146985381527e-104	0.325669578407632	0.203	0.119	2.11045147486741e-99	no change
1018	1018	DHRS4L2	9.92774548125015e-104	0.215359143734267	0.296	0.189	2.16861672292428e-99	no change
1019	1019	NOL11	1.28665675950314e-103	0.3871696115741	0.168	0.092	2.81057302545867e-99	no change
1020	1020	CPSF6	1.29946671702661e-103	0.182426904047117	0.315	0.204	2.83855509667294e-99	no change
1021	1021	SYNCRIP	1.84459206959852e-103	0.177121300415557	0.463	0.322	4.029326916831e-99	no change
1022	1022	S100A14	1.86567526211599e-103	-0.532737738	0.409	0.49	4.07538104256617e-99	no change

1023	1023	SERPING1	2.2722591267695e-103	-1.464888854	0.236	0.303	4.9635228365153e-99	down
1024	1024	HMGXB4	2.41424068905491e-103	0.333057524561659	0.267	0.169	5.27366736117155e-99	no change
1025	1025	YWHAQ	4.93179832182062e-103	0.185987308444224	0.761	0.604	1.0773020254185e-98	no change
1026	1026	LRRC1	5.16686029966671e-103	0.296467909278823	0.233	0.142	1.1286489638592e-98	no change
1027	1027	EEF1D	5.52921465776411e-103	0.118684770075941	0.99	0.968	1.20780164984199e-98	no change
1028	1028	PUM3	5.69129014058169e-103	0.304476744451952	0.171	0.094	1.24320541830867e-98	no change
1029	1029	QTRT1	5.98209820823122e-103	0.268771896340878	0.311	0.204	1.30672953260603e-98	no change
1030	1030	MTRNR2L1	7.23581323918089e-103	0.327745984006859	0.18	0.1	1.58059104396667e-98	no change
1031	1031	SRGN	8.12777395915751e-103	-0.304438345	0.248	0.156	1.77543094363837e-98	no change
1032	1032	TFDP2	8.42192159160905e-103	0.211578709356188	0.291	0.186	1.83968455247108e-98	no change
1033	1033	RPAP3	1.01532792622077e-102	0.433141082603955	0.172	0.096	2.21788232203665e-98	no change
1034	1034	GPX3	1.02321932160936e-102	-2.29478881	0.139	0.205	2.23512028612349e-98	down
1035	1035	C16orf13	1.03390520945335e-102	0.285466551650719	0.624	0.48	2.25846253952989e-98	no change

1036	1036	DDT	1.29624414337637e-102	0.418562796653555	0.863	0.747	2.83151570679134e-98	no change
1037	1037	SNAPC1	1.51740959835853e-102	0.292752608988634	0.252	0.156	3.31462952665438e-98	no change
1038	1038	YAF2	1.71556537732252e-102	0.288398760039925	0.326	0.217	3.7474810102233e-98	no change
1039	1039	EEF1E1	1.77344719637678e-102	0.20968813391162	0.41	0.281	3.87391805576544e-98	no change
1040	1040	C21orf59	2.10728065153754e-102	0.234405752410188	0.339	0.223	4.60314385521861e-98	no change
1041	1041	FOXA1	2.27089223659683e-102	0.19515624124615	0.374	0.252	4.96053700162213e-98	no change
1042	1042	KRT33B	2.54479928655359e-102	-2.040690435	0.038	0.087	5.55885956154767e-98	down
1043	1043	DUOXA1	2.91119388281146e-102	0.346400781851301	0.156	0.083	6.35921191761335e-98	no change
1044	1044	RRM1	2.9769969870475e-102	0.453477214277237	0.121	0.059	6.50295221850655e-98	no change
1045	1045	EIF4G3	3.04917724695103e-102	0.337206636922136	0.187	0.107	6.66062277823983e-98	no change
1046	1046	H2AFY	3.53210345612255e-102	0.215821206401398	0.716	0.559	7.7155267895541e-98	no change
1047	1047	GDI2	4.35327815434343e-102	0.102703528613014	0.707	0.535	9.50930080034779e-98	no change
1048	1048	IPO7	4.47343312299107e-102	0.161806251344203	0.271	0.169	9.77176731386169e-98	no change

1049	1049	NAALADL2	5.14269546096653e-102	0.360818737824867	0.142	0.073	1.12337039649353e-97	no change
1050	1050	SLC30A5	6.20631759351137e-102	0.257486280126511	0.21	0.124	1.35570801512662e-97	no change
1051	1051	IER5	6.46329582323044e-102	-0.775082361	0.308	0.377	1.41184233962646e-97	no change
1052	1052	PFKP	1.17687482931199e-101	0.311500299050429	0.226	0.135	2.57076537714911e-97	no change
1053	1053	TAOK1	1.60482207397511e-101	0.275736704627235	0.225	0.137	3.50557333839123e-97	no change
1054	1054	IDH3G	1.66632489535661e-101	0.199675655833909	0.38	0.256	3.63992010141697e-97	no change
1055	1055	CKAP5	2.37652156719799e-101	0.625233558644006	0.105	0.048	5.1912737113873e-97	no change
1056	1056	NUP85	2.43787979478459e-101	0.530137924682357	0.124	0.061	5.32530462372746e-97	no change
1057	1057	PPP2R2A	2.53848468464544e-101	0.163183579460442	0.435	0.299	5.54506594513949e-97	no change
1058	1058	CHD4	3.03006640339196e-101	0.24914982149342	0.339	0.226	6.61887705156939e-97	no change
1059	1059	ZNF770	3.19343629958436e-101	0.355037876739558	0.185	0.106	6.97574225281208e-97	no change
1060	1060	MTIF2	3.66733603326286e-101	0.469839304632533	0.151	0.08	8.0109288310594e-97	no change
1061	1061	ATIC	3.90885890519865e-101	0.335852179418806	0.224	0.136	8.53851139251593e-97	no change

1062	1062	H2AFZ	5.27236058328948e-101	0.388562034360468	0.888	0.78	1.15169444581375e-96	no change
1063	1063	DIS3	5.64157607832458e-101	0.287524653345612	0.189	0.109	1.23234587854922e-96	no change
1064	1064	ZDHHC13	7.09161770445752e-101	0.255355599883326	0.225	0.135	1.5490929713617e-96	no change
1065	1065	HK1	7.32820970784967e-101	0.25006696920242	0.248	0.153	1.60077412858268e-96	no change
1066	1066	FAH	8.35399549286461e-101	0.263055686560014	0.187	0.107	1.82484677546135e-96	no change
1067	1067	SLC39A6	9.02505583582555e-101	0.182123463556826	0.453	0.314	1.97143319677773e-96	no change
1068	1068	NTMT1	1.02611906271249e-100	0.277049330837755	0.257	0.161	2.24145448058917e-96	no change
1069	1069	HMG20B	1.10154412752877e-100	0.284491661213113	0.417	0.293	2.40621299217383e-96	no change
1070	1070	MFSD10	1.19155855141405e-100	0.232138794718306	0.471	0.334	2.60284049970885e-96	no change
1071	1071	SFTA2	1.32323081546964e-100	-1.864867527	0.048	0.101	2.89046539331189e-96	down
1072	1072	KIF5C	1.82437436498237e-100	0.450227135320432	0.134	0.069	3.98516336286749e-96	no change
1073	1073	GMPS	1.88886800203575e-100	0.320559850893679	0.181	0.103	4.12604326364689e-96	no change
1074	1074	RHOB	2.02295044504007e-100	-0.451304737	0.773	0.755	4.41893295214553e-96	no change

1075	1075	SNRPD2	2.6735924295235e-100	0.154087693368892	0.967	0.911	5.84019530305113e-96	no change
1076	1076	FAM25A	2.85361241041177e-100	-3.115158136	0.053	0.106	6.23343094930347e-96	down
1077	1077	RAD51AP1	3.58465584379747e-100	1.23712189847987	0.053	0.015	7.8303222251912e-96	up
1078	1078	TPRKB	3.63386793550113e-100	0.206784539872176	0.436	0.304	7.93782111830866e-96	no change
1079	1079	MRPL36	3.91602202176106e-100	0.346063409072391	0.532	0.4	8.55415850433487e-96	no change
1080	1080	EIF3G	3.94118497663797e-100	0.187857422732062	0.759	0.602	8.60912446296799e-96	no change
1081	1081	DYNC1H1	4.50187672235134e-100	0.185318430098453	0.3	0.193	9.83389951230427e-96	no change
1082	1082	BRCA1	5.20958242521041e-100	1.16019915729455	0.059	0.019	1.13798118496296e-95	up
1083	1083	CDCA8	5.314336562915e-100	1.64292408648041	0.044	0.01	1.16086367880315e-95	up
1084	1084	CMSS1	5.37480159831345e-100	0.205322291347892	0.287	0.184	1.17407166113559e-95	no change
1085	1085	DDX23	6.38917501290635e-100	0.44444021656975	0.18	0.103	1.39565138981926e-95	no change
1086	1086	FANCL	6.52092746956223e-100	0.500343416147675	0.15	0.08	1.42443139645117e-95	no change
1087	1087	CD24	6.90485532779997e-100	-0.892669769	0.753	0.674	1.50829659780463e-95	no change

1088	1088	CCNT1	6.9772206517098e-100	0.424238711533663	0.139	0.072	1.52410407915949e-95	no change
1089	1089	NDUFS2	6.99633847822199e-100	0.227851087052931	0.498	0.358	1.52828017718281e-95	no change
1090	1090	TRIP13	7.14355613778357e-100	1.05149048709637	0.072	0.027	1.56043840273744e-95	up
1091	1091	HMMR	7.36685333796133e-100	1.27775735059647	0.051	0.014	1.60921544314427e-95	up
1092	1092	MRPS18B	7.67929838861084e-100	0.2274367211815	0.404	0.279	1.67746594000815e-95	no change
1093	1093	NOL7	8.55262424991475e-100	0.181177928793193	0.515	0.365	1.86823524115138e-95	no change
1094	1094	METAP2	1.11330919148259e-99	0.196265447337433	0.612	0.453	2.43191259787456e-95	no change
1095	1095	ERMP1	1.26911286962412e-99	0.448579463700927	0.174	0.099	2.77225015240692e-95	no change
1096	1096	KLHL24	1.38433972761278e-99	0.322698976155495	0.212	0.127	3.02395170099736e-95	no change
1097	1097	YY1	1.43139963901004e-99	0.189404026598632	0.484	0.343	3.12674937145354e-95	no change
1098	1098	STEAP2	1.44355812190916e-99	0.367841633247766	0.268	0.171	3.15330836149837e-95	no change
1099	1099	MRPL37	1.66903169152588e-99	0.288815647521423	0.332	0.223	3.64583282696913e-95	no change
1100	1100	GTSE1	1.69214879096385e-99	1.52663470657414	0.045	0.011	3.69632981898143e-95	up

1101	1101	PHGDH	2.16230808264634e-99	0.688400771931075	0.225	0.144	4.72334577573267e-95	no change
1102	1102	UBA2	2.45331510207116e-99	0.216250799640967	0.307	0.2	5.35902150896424e-95	no change
1103	1103	CENPH	2.4590144194144e-99	0.473782681281568	0.123	0.061	5.37147109776882e-95	no change
1104	1104	EHMT2	3.33792431938923e-99	0.49323949771548	0.145	0.077	7.29136188327383e-95	no change
1105	1105	RBMS1	3.61728543117954e-99	0.116254495098077	0.532	0.376	7.90159829586858e-95	no change
1106	1106	COL6A2	3.64042028288693e-99	-1.722921266	0.047	0.099	7.95213406593821e-95	down
1107	1107	PLEC	4.73309579174477e-99	0.210919408436907	0.298	0.192	1.03389744474873e-94	no change
1108	1108	ZNRD1	4.91107874407906e-99	0.158025123960525	0.53	0.381	1.07277604085663e-94	no change
1109	1109	RPS27	5.46571181340465e-99	0.108399260633281	1	1	1.19393008852011e-94	no change
1110	1110	CNOT1	5.60765286219072e-99	0.231783328544354	0.266	0.168	1.22493569121694e-94	no change
1111	1111	ARID2	7.27404505249945e-99	0.393025195905177	0.167	0.093	1.58894240126798e-94	no change
1112	1112	MUC1	7.76526066988642e-99	-0.893109539	0.218	0.293	1.69624354072999e-94	no change
1113	1113	DUT	8.03807890923352e-99	0.224965803188758	0.654	0.498	1.75583795693297e-94	no change

1114	1114	ARF3	9.80600099649829e-99	0.244156974436462	0.408	0.284	2.14202285767509e-94	no change
1115	1115	HMCES	1.09200675830522e-98	0.326793238470951	0.208	0.123	2.38537956284192e-94	no change
1116	1116	LINC01355	1.26003966458536e-98	1.03081693478399	0.06	0.02	2.75243064332026e-94	up
1117	1117	MRPS9	1.33069919127492e-98	0.344027710838588	0.192	0.111	2.90677931342093e-94	no change
1118	1118	NARS	1.56696590935089e-98	0.18538373830595	0.475	0.335	3.42288033238608e-94	no change
1119	1119	UHMK1	1.57271336095098e-98	0.204857381873015	0.286	0.185	3.43543506566131e-94	no change
1120	1120	CYC1	1.81312774093068e-98	0.203423393347179	0.69	0.528	3.96059623728899e-94	no change
1121	1121	NR4A2	1.97550734817912e-98	0.144740950142455	0.367	0.253	4.31529825136247e-94	no change
1122	1122	EFEMP1	2.11691215112228e-98	-2.249483565	0.036	0.083	4.62418290291151e-94	down
1123	1123	NOLC1	2.47326162109428e-98	0.244985561753688	0.265	0.168	5.40259268511835e-94	no change
1124	1124	SFRP2	3.15660229085083e-98	-1.866427094	0.041	0.09	6.89528204413454e-94	down
1125	1125	PHKB	3.37290010196991e-98	0.256659797501048	0.179	0.102	7.36776298274306e-94	no change
1126	1126	CFAP36	3.43121153797359e-98	0.223554313861339	0.308	0.201	7.4951384835495e-94	no change

1127	1127	TIA1	3.48893528119871e-98	0.240687215520124	0.267	0.169	7.62123022825046e-94	no change
1128	1128	TSPAN7	4.26587066402428e-98	1.36343330233229	0.046	0.012	9.31836787849464e-94	up
1129	1129	NLE1	5.35825818333828e-98	0.711612499751892	0.085	0.035	1.17045791756841e-93	no change
1130	1130	MCM2	5.4736426756641e-98	1.27313043190768	0.052	0.015	1.19566250607207e-93	up
1131	1131	DHX40	6.17850869491047e-98	0.408651196146947	0.18	0.104	1.34963343931624e-93	no change
1132	1132	NABP2	1.16556537443243e-97	0.335066935284332	0.276	0.179	2.5460610039102e-93	no change
1133	1133	GUK1	1.24033854759691e-97	-0.318281503	0.866	0.814	2.7093955233707e-93	no change
1134	1134	ERBB2IP	1.56851868625214e-97	0.235270970071245	0.251	0.157	3.42627221824917e-93	no change
1135	1135	KCNS3	1.59829719514911e-97	0.535370623642461	0.126	0.064	3.49132039308372e-93	no change
1136	1136	LINC00969	1.97900263138059e-97	0.212163930245212	0.347	0.233	4.32293334798776e-93	no change
1137	1137	TOR1A	2.17683403079378e-97	0.364503220484671	0.151	0.081	4.75507625686592e-93	no change
1138	1138	AP3B1	2.18936512356114e-97	0.29524583830919	0.166	0.093	4.78244917590696e-93	no change
1139	1139	E2F3	2.75921233977979e-97	0.488640783080284	0.122	0.061	6.02722343501498e-93	no change

1140	1140	CDK11B	3.77982471464938e-97	0.359279438928687	0.19	0.111	8.2566491066801e-93	no change
1141	1141	SSH3	4.1813566424619e-97	0.209549212681281	0.443	0.311	9.13375544979378e-93	no change
1142	1142	CD63	4.44125482628592e-97	-0.208416557	0.98	0.961	9.70147704253896e-93	no change
1143	1143	PSMD12	5.23353686352433e-97	0.173475857072674	0.409	0.28	1.14321379246826e-92	no change
1144	1144	APITD1	5.32190476912767e-97	0.686679862045926	0.112	0.054	1.16251687776825e-92	no change
1145	1145	STK24	6.20056744683689e-97	0.201827200054149	0.403	0.278	1.35445195308705e-92	no change
1146	1146	PPA2	6.98541799905044e-97	0.215750592608838	0.441	0.312	1.52589470771258e-92	no change
1147	1147	SPEN	7.51408989222293e-97	0.264898454062364	0.213	0.128	1.64137779605718e-92	no change
1148	1148	VPS13A	9.27233833073876e-97	0.395147699410794	0.149	0.081	2.02544958496658e-92	no change
1149	1149	PITPNA-AS1	9.57701782709434e-97	0.334333498914877	0.216	0.132	2.09200377415049e-92	no change
1150	1150	PSMG4	9.9852227375603e-97	0.198176592204796	0.291	0.189	2.18117205479267e-92	no change
1151	1151	NIT2	1.02644809157506e-96	0.234110945278648	0.414	0.289	2.24217321123655e-92	no change
1152	1152	WDR46	1.09453481546664e-96	0.210973024035868	0.235	0.143	2.39090185090533e-92	no change

1153	1153	MRE11A	1.09603147719544e-96	0.47585705340663	0.125	0.063	2.39417115878571e-92	no change
1154	1154	WHSC1	1.34983257290136e-96	0.800638663996331	0.091	0.04	2.94857427224574e-92	no change
1155	1155	GNAQ	1.35993303353557e-96	0.215905358678869	0.264	0.168	2.9706377184551e-92	no change
1156	1156	ATP5D	1.48517459667341e-96	0.165831878584067	0.915	0.817	3.2442153889734e-92	no change
1157	1157	ZCCHC11	1.4865074095615e-96	0.27509126546428	0.19	0.11	3.24712678544614e-92	no change
1158	1158	CDCA3	1.6316792827108e-96	1.57943000975781	0.053	0.016	3.56424022515347e-92	up
1159	1159	PLK3	2.19348834196428e-96	-0.821836033	0.3	0.363	4.79145593418676e-92	no change
1160	1160	PFDN6	2.49514493721028e-96	0.186114725636663	0.462	0.326	5.45039460084214e-92	no change
1161	1161	ZNF146	2.53358572737283e-96	0.324154715776555	0.21	0.127	5.53436466287321e-92	no change
1162	1162	GATA2-AS1	2.59165445353216e-96	0.452789305145377	0.188	0.111	5.66120998829564e-92	no change
1163	1163	PRPS2	2.71881200392431e-96	0.180137048034891	0.249	0.155	5.93897294137226e-92	no change
1164	1164	TOX3	2.79405288159291e-96	0.318940767482377	0.254	0.161	6.10332911455156e-92	no change
1165	1165	DPT	2.83114396241578e-96	-2.17813332	0.033	0.078	6.18435087150103e-92	down

1166	1166	COPB2	2.86999682457284e-96	0.250209411656966	0.236	0.146	6.2692210635969e-92	no change
1167	1167	NELFCD	3.02704947377545e-96	0.245581191364629	0.349	0.236	6.61228687051509e-92	no change
1168	1168	MAPK10	3.07898365433908e-96	0.384302728744232	0.169	0.096	6.72573189453829e-92	no change
1169	1169	METTL7A	4.18920577642272e-96	0.178302950906773	0.45	0.315	9.1509010980178e-92	no change
1170	1170	FAM136A	4.27124268581152e-96	0.258023184506012	0.377	0.262	9.33010252288669e-92	no change
1171	1171	FH	4.63126215749123e-96	0.357348886001644	0.175	0.1	1.01165290568239e-91	no change
1172	1172	FBXO4	4.66091030121682e-96	0.420568812438375	0.14	0.074	1.0181292461978e-91	no change
1173	1173	CEP78	4.85590959620345e-96	0.89663255660304	0.062	0.021	1.06072489219468e-91	no change
1174	1174	UTP18	4.99904401391172e-96	0.244967772371207	0.229	0.14	1.09199117439888e-91	no change
1175	1175	MYBL2	5.12274025137261e-96	1.46408696339342	0.049	0.014	1.11901138050983e-91	up
1176	1176	RABEPK	5.26904476328802e-96	0.572943219136872	0.109	0.052	1.15097013809263e-91	no change
1177	1177	PSAP	6.26307454673582e-96	-0.52736095	0.774	0.721	1.36810600398897e-91	no change
1178	1178	MAML2	7.60161511493964e-96	0.377988038038819	0.136	0.071	1.66049680570742e-91	no change

1179	1179	ITGA2	7.82748032195108e-96	0.349472371409512	0.196	0.116	1.70983480152699e-91	no change
1180	1180	NUSAP1	8.1971691094494e-96	0.924516042257635	0.096	0.044	1.79058962026813e-91	no change
1181	1181	NT5E	8.27557764820256e-96	0.567617501186966	0.078	0.031	1.80771718147337e-91	no change
1182	1182	RNF5	9.33086363662379e-96	0.204333541748449	0.398	0.274	2.0382338527841e-91	no change
1183	1183	PHF6	1.0359409158586e-95	0.483245634555246	0.144	0.077	2.26290933660154e-91	no change
1184	1184	EVA1C	1.10514575860374e-95	0.567908751632818	0.083	0.034	2.414080395094e-91	no change
1185	1185	CKAP2	1.19471689814778e-95	0.561912223790659	0.137	0.073	2.60973959231401e-91	no change
1186	1186	RBM3	1.19849817832414e-95	0.213916286675574	0.752	0.603	2.61799942073124e-91	no change
1187	1187	OXA1L	1.24754125777906e-95	0.177424861995944	0.377	0.257	2.72512912349258e-91	no change
1188	1188	ALG6	1.26235297398343e-95	0.366421107201513	0.143	0.076	2.75748383636941e-91	no change
1189	1189	OGT	1.35238961848266e-95	0.270482168559235	0.215	0.13	2.95415988261353e-91	no change
1190	1190	HADH	1.36595908578949e-95	0.246533683587257	0.31	0.205	2.98380102699856e-91	no change
1191	1191	UBE2G2	1.41868132493739e-95	0.23785014682201	0.252	0.159	3.09896748619323e-91	no change

1192	1192	MTHFS	1.66420967220796e-95	0.367918487906441	0.179	0.103	3.63529960797106e-91	no change
1193	1193	ESRP1	1.87712716028899e-95	0.269538780572196	0.258	0.165	4.10039656893528e-91	no change
1194	1194	COPS4	2.12310576573505e-95	0.211030108129038	0.277	0.177	4.63771223467165e-91	no change
1195	1195	ARFGEF3	2.21349939017676e-95	0.344851192594141	0.185	0.108	4.83516806790212e-91	no change
1196	1196	SCD	2.33970281438266e-95	-1.114944445	0.191	0.262	5.11084682773749e-91	down
1197	1197	RBL2	2.3940414056655e-95	0.271271329140267	0.225	0.138	5.22954404653573e-91	no change
1198	1198	GPN3	2.4287360647484e-95	0.278770803651518	0.222	0.135	5.30533105983641e-91	no change
1199	1199	TBX3	2.66035286122093e-95	0.238793021816386	0.493	0.359	5.81127479005099e-91	no change
1200	1200	TMEM38B	2.99035340876595e-95	0.66318740649413	0.103	0.049	6.53212798610833e-91	no change
1201	1201	KNOP1	3.21347221183355e-95	0.26741196614393	0.301	0.2	7.0195086995292e-91	no change
1202	1202	ANAPC7	3.87459182774673e-95	0.410929270647113	0.146	0.079	8.46365838852995e-91	no change
1203	1203	MRPL22	4.19011942309516e-95	0.161735640403687	0.433	0.298	9.15289686780906e-91	no change
1204	1204	MRPL1	4.93563247646599e-95	0.227139293531514	0.268	0.172	1.07813955815923e-90	no change

1205	1205	DRAM2	4.9593635393344e-95	0.189943922990314	0.398	0.276	1.08332337153221e-90	no change
1206	1206	DNAJA2	6.30191973106351e-95	0.159640147963423	0.393	0.27	1.37659134605351e-90	no change
1207	1207	NUDT16L1	6.39073997408683e-95	0.274603080641776	0.274	0.177	1.39599323993953e-90	no change
1208	1208	PNN	6.90953325519285e-95	0.18749079180161	0.48	0.341	1.50931844426433e-90	no change
1209	1209	PRKAG1	6.91801779176509e-95	0.296307652058352	0.257	0.164	1.51117180643317e-90	no change
1210	1210	KIAA0101	7.58004294404959e-95	0.961578663953802	0.208	0.134	1.65578458069819e-90	no change
1211	1211	EFTUD2	8.22898528450118e-95	0.474042926699572	0.146	0.079	1.79753954554644e-90	no change
1212	1212	NT5DC1	8.97451794599773e-95	0.338919258577056	0.209	0.127	1.96039370012374e-90	no change
1213	1213	KPNA6	1.00577485635311e-94	0.271924176242262	0.264	0.17	2.19701459621774e-90	no change
1214	1214	RP3-395M20.12	1.12132837064851e-94	-1.661330125	0.038	0.085	2.4494296928446e-90	down
1215	1215	MRPL11	1.2569797268753e-94	0.224344517167161	0.527	0.388	2.74574651538641e-90	no change
1216	1216	CEBPB	1.49718787950656e-94	-0.624871496	0.721	0.702	3.27045720399413e-90	no change
1217	1217	FKBP3	1.59170511564212e-94	0.183591420458956	0.521	0.376	3.47692065460865e-90	no change

1218	1218	TJP2	1.66585468922403e-94	0.187721218879152	0.273	0.175	3.63889298314097e-90	no change
1219	1219	NUDT8	1.81752244237846e-94	0.302476726062428	0.215	0.133	3.9701960231315e-90	no change
1220	1220	S100A4	1.95898185547222e-94	-0.429633676	0.576	0.632	4.27919996509352e-90	no change
1221	1221	CITED4	2.03565117850558e-94	-0.837718956	0.202	0.275	4.4466764343276e-90	no change
1222	1222	BTBD2	2.63180105683567e-94	0.507129502330519	0.13	0.068	5.74890622855184e-90	no change
1223	1223	SMARCB1	2.64744591317473e-94	0.250301616802234	0.419	0.296	5.78308085273887e-90	no change
1224	1224	ADD1	2.72786303980821e-94	0.253305349615838	0.243	0.153	5.95874402415705e-90	no change
1225	1225	COX7C	2.74263452440376e-94	0.127200184882804	0.998	0.99	5.99101085510758e-90	no change
1226	1226	U2AF1	3.54956294776798e-94	0.27024229216655	0.186	0.108	7.75366530310438e-90	no change
1227	1227	PHF10	4.2357688438264e-94	0.278161804649008	0.168	0.095	9.2526134624544e-90	no change
1228	1228	HDDC2	4.45909200531406e-94	0.1811560694983	0.437	0.306	9.74044057640803e-90	no change
1229	1229	PDCD5	4.67062686278646e-94	0.246286884308042	0.685	0.536	1.02025173190707e-89	no change
1230	1230	KDM3B	4.79269820377695e-94	0.341505680635592	0.137	0.073	1.04691699563304e-89	no change

1231	1231	NDUFA9	5.7654615600609e-94	0.211135506187595	0.371	0.253	1.2594074231797e-89	no change
1232	1232	ANAPC4	5.95921407545883e-94	0.327484435863063	0.162	0.09	1.30173072264323e-89	no change
1233	1233	LRRCC1	5.9872406220072e-94	0.547906957136637	0.097	0.044	1.30785284147125e-89	no change
1234	1234	ATP2B1	6.11456744216871e-94	0.191409058775738	0.439	0.309	1.33566611206733e-89	no change
1235	1235	NETO2	6.40130155456509e-94	1.12183585019637	0.06	0.02	1.3983003115792e-89	up
1236	1236	EFNA5	7.24554704224063e-94	0.14049397622976	0.19	0.111	1.58271729590704e-89	no change
1237	1237	UBQLN1	7.8721865663513e-94	0.203839203771065	0.262	0.167	1.71960043355378e-89	no change
1238	1238	FERMT1	8.28227551378747e-94	0.223983899087051	0.332	0.223	1.80918026323173e-89	no change
1239	1239	TTC14	1.19354178029449e-93	0.297813014203137	0.258	0.167	2.60717266487527e-89	no change
1240	1240	JAGN1	1.23956940997514e-93	0.201501787580153	0.305	0.201	2.70771541914969e-89	no change
1241	1241	CHD7	1.29187306910822e-93	0.402030132046327	0.153	0.085	2.82196753215999e-89	no change
1242	1242	RACGAP1	1.33399396544607e-93	1.01478286007493	0.061	0.021	2.91397641812038e-89	up
1243	1243	HSPB8	1.35830730160891e-93	-1.141731701	0.132	0.199	2.9670864696345e-89	down

1244	1244	CHORDC1	1.36637526605857e-93	0.247632934033215	0.278	0.18	2.98471013117835e-89	no change
1245	1245	USP48	1.41120182525028e-93	0.278672537282209	0.245	0.155	3.08262926707672e-89	no change
1246	1246	TMPRSS4	1.53366164256894e-93	0.23695973157584	0.381	0.264	3.35013049202759e-89	no change
1247	1247	MTA1	1.80151806780267e-93	0.299414639655045	0.221	0.136	3.93523606730816e-89	no change
1248	1248	PRRC2C	1.82807779920619e-93	0.206424589980783	0.702	0.546	3.99325314458601e-89	no change
1249	1249	STOML2	1.977359214688e-93	0.183508035760866	0.542	0.394	4.31934346856447e-89	no change
1250	1250	SSR2	2.00515194062266e-93	0.175354800744886	0.856	0.729	4.38005389909614e-89	no change
1251	1251	B4GALT4	2.20624895411781e-93	0.224420051773908	0.357	0.245	4.81933021537495e-89	no change
1252	1252	RBFOX2	2.2206263557177e-93	0.297065459171953	0.247	0.157	4.85073621142975e-89	no change
1253	1253	COPA	2.40830035004384e-93	0.22705647362069	0.287	0.187	5.26069128463577e-89	no change
1254	1254	OAZ1	2.55586886227877e-93	-0.164211153	0.986	0.966	5.58303994276175e-89	no change
1255	1255	PI3	2.56217670421088e-93	-2.331317844	0.048	0.098	5.59681879267824e-89	down
1256	1256	CNDP2	2.62516450572601e-93	0.187649889522357	0.476	0.34	5.7344093463079e-89	no change

1257	1257	CTBP2	2.6792728170454e-93	0.321099371262116	0.191	0.113	5.85260354155398e-89	no change
1258	1258	PWAR6	2.864386444984e-93	0.85675396491399	0.059	0.02	6.25696575042304e-89	no change
1259	1259	SPC25	3.03982062760489e-93	1.27526125489651	0.048	0.014	6.64018417894013e-89	up
1260	1260	RARS	3.28374680587179e-93	0.204035094135423	0.25	0.157	7.17301652274635e-89	no change
1261	1261	PUM1	3.87948414597028e-93	0.235163905216194	0.26	0.166	8.47434516845748e-89	no change
1262	1262	C10orf10	3.95698844205407e-93	-1.318388698	0.125	0.191	8.64364555282291e-89	down
1263	1263	RP11-554I8.2	4.30000513885089e-93	0.619412745028762	0.091	0.04	9.39293122530589e-89	no change
1264	1264	RBM10	4.50167633691721e-93	0.42998216445373	0.14	0.075	9.83346179036195e-89	no change
1265	1265	RRM2	6.39539954968045e-93	1.05280449265394	0.079	0.033	1.3970110776322e-88	up
1266	1266	SURF1	6.55071931971616e-93	0.154315258171789	0.376	0.255	1.4309391281988e-88	no change
1267	1267	GJB6	7.05553400697778e-93	0.36731657012045	0.203	0.123	1.54121084848423e-88	no change
1268	1268	HN1L	7.12240079119839e-93	0.285058323026337	0.25	0.159	1.55581722882938e-88	no change
1269	1269	PSMD10	8.08551273862961e-93	0.18456885026592	0.306	0.203	1.76619940262625e-88	no change

1270	1270	ACIN1	8.14671527858379e-93	0.290497036869291	0.246	0.155	1.77956848545384e-88	no change
1271	1271	CSNK1G3	9.62077894441814e-93	0.263975687909696	0.199	0.118	2.1015629526187e-88	no change
1272	1272	RP11-44F21.5	9.69707387665216e-93	0.164664117165746	0.436	0.303	2.1182288176159e-88	no change
1273	1273	RPN1	9.7491087489371e-93	0.192773385506001	0.399	0.278	2.12959531511782e-88	no change
1274	1274	FRMD4A	9.9684556402106e-93	0.504387812552443	0.111	0.054	2.1775094500476e-88	no change
1275	1275	EIF3J	1.01462745478592e-92	0.167219828445938	0.622	0.467	2.21635221223437e-88	no change
1276	1276	ASH1L	1.15723980506811e-92	0.131794351041301	0.335	0.223	2.52787463019079e-88	no change
1277	1277	DICER1	1.43614025560253e-92	0.205688120124759	0.247	0.155	3.13710477433817e-88	no change
1278	1278	ARMCX3	1.46543061433151e-92	0.186420574269081	0.374	0.257	3.20108663394576e-88	no change
1279	1279	BTBD16	1.64582346553199e-92	0.290970589236502	0.54	0.427	3.59513677810807e-88	no change
1280	1280	TNRC6A	1.70410866320012e-92	0.304148167848159	0.195	0.117	3.72245496389434e-88	no change
1281	1281	LCMT1	1.92586578827557e-92	0.232864216427914	0.316	0.211	4.20686122790916e-88	no change
1282	1282	KDM5A	2.357401181114e-92	0.264407153871797	0.229	0.142	5.14950714002541e-88	no change

1283	1283	COPZ1	4.10192937987537e-92	0.201750804539404	0.524	0.383	8.96025453739976e-88	no change
1284	1284	ITM2B	4.4091162213473e-92	-0.199949305	0.983	0.968	9.63127347391104e-88	no change
1285	1285	ACADM	4.7640894171563e-92	0.160316216503773	0.377	0.256	1.04066769228362e-87	no change
1286	1286	NACA2	5.16727194722486e-92	0.625202473815502	0.082	0.035	1.1287388841518e-87	no change
1287	1287	ISG15	5.26190154963153e-92	-0.662080715	0.667	0.651	1.14940977450151e-87	no change
1288	1288	USO1	5.42352896046485e-92	0.174653546939706	0.306	0.202	1.18471566612394e-87	no change
1289	1289	PSMD14	8.52596149775722e-92	0.228561301000691	0.378	0.262	1.86241102957009e-87	no change
1290	1290	TIAM1	8.61938942298756e-92	0.389881793833341	0.198	0.12	1.8828194255574e-87	no change
1291	1291	ITPRIPL2	8.89578578862808e-92	0.191237911051819	0.233	0.145	1.94319544766792e-87	no change
1292	1292	MLLT6	9.38623660650676e-92	0.287381713410474	0.172	0.099	2.05032952432534e-87	no change
1293	1293	MAP3K6	9.97264149762813e-92	0.404084083329265	0.113	0.056	2.17842380874189e-87	no change
1294	1294	INHBB	1.08514808463584e-91	1.16779245843671	0.051	0.015	2.37039747607853e-87	up
1295	1295	CASP8	1.14507675252291e-91	0.399659297254872	0.158	0.089	2.50130565821105e-87	no change

1296	1296	KPNA4	1.35179906339151e-91	0.206269444028387	0.301	0.2	2.95286987407241e-87	no change
1297	1297	COL4A5	1.36450904862632e-91	0.310063784860878	0.147	0.08	2.98063356581934e-87	no change
1298	1298	TIMELESS	1.46560449560831e-91	0.903534147559449	0.071	0.028	3.20146646020679e-87	no change
1299	1299	SLC35E2B	1.48661609725925e-91	0.437575505815847	0.147	0.081	3.2473642028531e-87	no change
1300	1300	WBP11	1.6415681201738e-91	0.218547263647383	0.314	0.21	3.58584140170764e-87	no change
1301	1301	ARHGEF19	1.69849081082388e-91	0.573888630915439	0.095	0.043	3.71018332716368e-87	no change
1302	1302	KEAP1	1.76593733530835e-91	0.334520396497854	0.196	0.118	3.85751351524756e-87	no change
1303	1303	PFN2	1.81745448990184e-91	0.185674630159815	0.637	0.476	3.97004758774158e-87	no change
1304	1304	MBNL1	1.83054383134143e-91	0.107596061251912	0.274	0.176	3.99863994518223e-87	no change
1305	1305	CHID1	2.0123501514103e-91	0.125681766311547	0.388	0.265	4.39577767074067e-87	no change
1306	1306	ZNF638	2.05586946280501e-91	0.207522582984172	0.217	0.132	4.49084125455126e-87	no change
1307	1307	GNL1	2.0935701820051e-91	0.168141332964164	0.333	0.222	4.57319470557195e-87	no change
1308	1308	POP5	2.20659582342221e-91	0.161135449319879	0.334	0.223	4.82008791668348e-87	no change

1309	1309	KIAA0907	2.63444137797833e-91	0.253143936	0.267	0.173	5.75467374605586e-87	no change
1310	1310	GPBP1L1	3.68658557716692e-91	0.180520836776127	0.304	0.201	8.05297753476343e-87	no change
1311	1311	C8orf59	4.027599197607e-91	0.166628910352272	0.561	0.413	8.79788768725274e-87	no change
1312	1312	RARRES2	4.20101086096637e-91	-1.60119276	0.069	0.124	9.17668812469494e-87	down
1313	1313	NAA10	4.5691639722868e-91	0.162630094985172	0.554	0.406	9.98088178106328e-87	no change
1314	1314	MTHFD1	5.36402983223164e-91	0.537893367699887	0.116	0.059	1.17171867655268e-86	no change
1315	1315	BCCIP	5.66921304588844e-91	0.258981785035156	0.214	0.131	1.23838289774387e-86	no change
1316	1316	PKN2	5.71366958482415e-91	0.205693431963554	0.311	0.208	1.24809398410899e-86	no change
1317	1317	IMP4	5.96163357645636e-91	0.184369722378849	0.402	0.281	1.30225923844113e-86	no change
1318	1318	PTCD3	6.52106089817364e-91	0.345686448697809	0.161	0.091	1.42446054259705e-86	no change
1319	1319	EIF4H	7.33972020469199e-91	0.207095690944123	0.474	0.342	1.60328848151292e-86	no change
1320	1320	MRPL47	9.1381977532884e-91	0.202894775050498	0.466	0.333	1.99614791722832e-86	no change
1321	1321	EED	9.92357176884356e-91	0.406748541344515	0.141	0.077	2.16770501718619e-86	no change

1322	1322	RP11-456O19.2	1.03933924761128e-90	1.92716918148803	0.033	0.006	2.27033265248208e-86	up
1323	1323	RBMX2	1.04614096104457e-90	0.214075011118615	0.28	0.183	2.28519031530576e-86	no change
1324	1324	CD2BP2	1.1305865085977e-90	0.201996592163149	0.312	0.207	2.46965316938081e-86	no change
1325	1325	AC098973.2	1.23791518979415e-90	0.349643336553179	0.173	0.099	2.70410194058633e-86	no change
1326	1326	C1GALT1C1	1.26455369595751e-90	0.199353902504455	0.201	0.119	2.76229109344958e-86	no change
1327	1327	COL1A2	1.62953780590373e-90	-1.492411615	0.064	0.118	3.55956238321611e-86	down
1328	1328	GSTM1	1.64031501249109e-90	-0.997761534	0.11	0.174	3.58310411328554e-86	no change
1329	1329	AIFM1	1.68863409949938e-90	0.410191770131922	0.146	0.08	3.68865232694644e-86	no change
1330	1330	NDUFAB1	1.90757167927397e-90	0.207943536668043	0.736	0.582	4.16689957620605e-86	no change
1331	1331	SCO1	2.28829876834237e-90	0.47107332001923	0.146	0.081	4.99855982956707e-86	no change
1332	1332	NCK1	2.32383218074751e-90	0.156203253102363	0.233	0.145	5.07617901562486e-86	no change
1333	1333	HENMT1	2.43550957245718e-90	0.379736552329167	0.15	0.083	5.32012711007547e-86	no change
1334	1334	TTYH1	2.62591270671034e-90	-2.064092614	0.026	0.066	5.73604371653806e-86	down

1335	1335	ACTR6	3.2982391215122e-90	0.285514034365082	0.206	0.125	7.20467353703126e-86	no change
1336	1336	POLD3	4.55624274456485e-90	0.596087354117625	0.088	0.039	9.95265665122745e-86	no change
1337	1337	UTP11L	4.77817997800321e-90	0.183434407329387	0.27	0.174	1.04374563439502e-85	no change
1338	1338	MUM1	4.85047226294946e-90	0.202672972658851	0.307	0.205	1.05953716111868e-85	no change
1339	1339	ARGLU1	5.17585768062069e-90	0.194934753838107	0.687	0.537	1.13061435175478e-85	no change
1340	1340	ATM	5.86548303676538e-90	0.346306025565851	0.132	0.069	1.28125611455103e-85	no change
1341	1341	ATP11C	5.90509214986118e-90	0.514943600560583	0.113	0.056	1.28990832921568e-85	no change
1342	1342	RBM42	5.9140707136858e-90	0.229176327490332	0.355	0.244	1.29186960669753e-85	no change
1343	1343	CAPN1	5.96202438710733e-90	0.217283579445942	0.61	0.464	1.30234460711972e-85	no change
1344	1344	MT-ND1	6.77776789043324e-90	-0.279409439	0.995	0.993	1.48053561798624e-85	no change
1345	1345	ASCC2	7.80922156860419e-90	0.322598657218959	0.323	0.221	1.7058463594459e-85	no change
1346	1346	NSUN2	8.48248544869089e-90	0.458175881375375	0.146	0.081	1.85291412141204e-85	no change
1347	1347	XIAP	9.84185329335003e-90	0.188133612539934	0.27	0.175	2.14985443339938e-85	no change

1348	1348	JAG1	1.07436760937416e-89	0.105950406293448	0.223	0.136	2.34684860591691e-85	no change
1349	1349	MPST	1.20321045127948e-89	0.253271676354312	0.59	0.447	2.6282929097749e-85	no change
1350	1350	HSP90AB1	1.58013470146214e-89	0.158822122346428	0.986	0.957	3.4516462418739e-85	no change
1351	1351	ATPAF1	1.65160087109704e-89	0.300332098153763	0.207	0.127	3.60775694282437e-85	no change
1352	1352	SETD9	1.9195223229679e-89	0.383020474442282	0.112	0.056	4.19300456229109e-85	no change
1353	1353	GNAI1	2.07387679641872e-89	0.252675056673337	0.194	0.116	4.53017647409706e-85	no change
1354	1354	TSN	2.11214680676638e-89	0.21135348960931	0.303	0.202	4.61377348470048e-85	no change
1355	1355	DDX42	2.16689675351365e-89	0.337641599160973	0.207	0.128	4.73336926837522e-85	no change
1356	1356	IGF1R	2.28906740774884e-89	0.395184592239895	0.126	0.066	5.00023884548658e-85	no change
1357	1357	AGAP2-AS1	2.29457786372265e-89	0.80226465683854	0.058	0.02	5.01227588551576e-85	no change
1358	1358	MFAP4	2.54659213934384e-89	-1.907545406	0.05	0.098	5.56277586918268e-85	down
1359	1359	ZMPSTE24	2.79454944550192e-89	0.260881722408987	0.242	0.155	6.1044138087544e-85	no change
1360	1360	MRRF	2.99317972543908e-89	0.382506846280701	0.151	0.085	6.53830179224913e-85	no change

1361	1361	PRDX1	3.37877226319512e-89	0.146953934276959	0.958	0.903	7.38059013172342e-85	no change
1362	1362	PTGR1	3.40962228661973e-89	0.216081982019608	0.586	0.459	7.44797892289213e-85	no change
1363	1363	CSNK1A1	3.53196523204318e-89	0.142610618080277	0.709	0.548	7.71522485287513e-85	no change
1364	1364	LINC01213	3.69087515890425e-89	-1.751755449	0.037	0.081	8.06234769711045e-85	down
1365	1365	CBWD1	4.94844085373182e-89	0.227563711943594	0.239	0.151	1.08093742008918e-84	no change
1366	1366	TMEM9	5.04815841896797e-89	0.273385177834272	0.32	0.218	1.10271972503936e-84	no change
1367	1367	MYH9	5.93352690836465e-89	0.199227134519772	0.389	0.271	1.29611961786317e-84	no change
1368	1368	KPNA2	6.27469160188649e-89	0.57699536695559	0.181	0.109	1.37064363351608e-84	no change
1369	1369	MAP7	6.39390241993138e-89	0.255478619042979	0.252	0.162	1.39668404460981e-84	no change
1370	1370	ANAPC15	6.6847330890294e-89	0.197952893095334	0.346	0.236	1.46021309596758e-84	no change
1371	1371	VAV3	6.8940970347896e-89	0.2652306881228	0.222	0.138	1.50594655627944e-84	no change
1372	1372	ALKBH2	7.10702169354202e-89	0.304317260159458	0.19	0.113	1.55245781873732e-84	no change
1373	1373	CENPM	7.82772496235361e-89	1.0300176445496	0.089	0.041	1.70988824077652e-84	up

1374	1374	SNRPC		8.88395614652386e-89	0.190983299123641	0.564	0.419	1.94061138064667e-84	no change
1375	1375	ROCK1		9.2020807841937e-89	0.205414934377304	0.294	0.194	2.01010252649927e-84	no change
1376	1376	BEX4		9.37879926236865e-89	0.251367248106338	0.432	0.312	2.04870491087181e-84	no change
1377	1377		7-Mar	9.92984133268107e-89	0.193783362160344	0.249	0.159	2.16907454071085e-84	no change
1378	1378	SPRR3		1.10012047258079e-88	-2.606886197	0.094	0.152	2.40310316030549e-84	down
1379	1379	PTPN18		1.21680842713574e-88	0.270663160326975	0.162	0.091	2.65799632823532e-84	no change
1380	1380	FLOT2		1.31986253118069e-88	0.217249057482531	0.34	0.231	2.88310771311111e-84	no change
1381	1381	CLDN4		1.49735850893169e-88	-0.355217883	0.905	0.86	3.27082992691038e-84	no change
1382	1382	DLG1		1.60242871008219e-88	0.173075779038156	0.267	0.173	3.50034527430353e-84	no change
1383	1383	PLAC9		1.79086593009917e-88	-1.922536739	0.054	0.103	3.91196753770863e-84	down
1384	1384	ACO2		1.79461966046825e-88	0.187666638283864	0.335	0.227	3.92016718632684e-84	no change
1385	1385	DIAPH1		1.86034921931393e-88	0.239220438897513	0.169	0.097	4.06374683466936e-84	no change
1386	1386	CDC42BPA		1.94964040169955e-88	0.386383702219721	0.163	0.094	4.25879449347249e-84	no change

1387	1387	SMG1	2.00579005974453e-88	0.198635404884189	0.261	0.169	4.38144780650596e-84	no change
1388	1388	WDR18	2.22606026403916e-88	0.2228619390388	0.216	0.133	4.86260604076714e-84	no change
1389	1389	EIF3B	2.42498851938733e-88	0.19776893918407	0.263	0.17	5.29714492174969e-84	no change
1390	1390	YAP1	2.47679693043165e-88	0.196658578323341	0.287	0.189	5.4103152148349e-84	no change
1391	1391	IQCB1	2.94615666799143e-88	0.369973880865897	0.138	0.074	6.43558462556048e-84	no change
1392	1392	CTD-2034I4.2	3.01438921438917e-88	0.48290659292831	0.088	0.039	6.58463179991171e-84	no change
1393	1393	CNOT6	3.13774174211631e-88	0.452571004219591	0.122	0.063	6.85408306147886e-84	no change
1394	1394	NNMT	3.20559516249557e-88	-1.956259894	0.048	0.096	7.00230207295533e-84	down
1395	1395	PPP4R3A	3.45709149692823e-88	0.145882886815573	0.241	0.153	7.55167066589002e-84	no change
1396	1396	U2AF2	3.8522211533873e-88	0.248383639775662	0.226	0.141	8.41479188745921e-84	no change
1397	1397	POGZ	4.11649859871544e-88	0.19026008345071	0.234	0.148	8.99207953903402e-84	no change
1398	1398	DIMT1	4.19664289385698e-88	0.185921372929418	0.222	0.137	9.16714673734119e-84	no change
1399	1399	LUZP1	4.59700605421241e-88	0.210167682525991	0.335	0.228	1.00417000248216e-83	no change

1400	1400	RP11-262H14.4	4.96250486707413e-88	0.712879299899974	0.112	0.057	1.08400956316367e-83	no change
1401	1401	TRIM59	5.42489757187117e-88	0.983599636740557	0.059	0.02	1.18501462559954e-83	no change
1402	1402	MAP1LC3B	5.84388901950425e-88	-0.52194787	0.602	0.587	1.27653911742051e-83	no change
1403	1403	SPIDR	6.06727018023124e-88	0.427827323200442	0.137	0.075	1.32533449816971e-83	no change
1404	1404	TBL1X	6.43362518823423e-88	0.552940312929846	0.101	0.048	1.40536108611788e-83	no change
1405	1405	HIPK2	6.48739059520508e-88	0.299108339720332	0.122	0.063	1.4171056016166e-83	no change
1406	1406	ARMCX6	6.62916201851829e-88	0.226776305578599	0.288	0.191	1.44807415132513e-83	no change
1407	1407	PAIP1	6.95388319066221e-88	0.152915120933633	0.418	0.292	1.51900624416825e-83	no change
1408	1408	GSDMD	6.95878151222984e-88	0.153136690775016	0.345	0.232	1.52007623353149e-83	no change
1409	1409	PRKACB	7.12687362238402e-88	0.368223378762158	0.147	0.082	1.55679427407357e-83	no change
1410	1410	SNX31	9.56151912241902e-88	0.251059670446795	0.316	0.216	2.08861823710121e-83	no change
1411	1411	AP3D1	1.00841905363318e-87	0.270879275374142	0.259	0.169	2.20279058075632e-83	no change
1412	1412	ARG2	1.02391695735396e-87	0.237995309265186	0.277	0.183	2.236644201644e-83	no change

1413	1413	CDKN2AIPNL	1.13408406686423e-87	0.204711435029318	0.227	0.141	2.47729323565822e-83	no change
1414	1414	RAC3	1.29635479127009e-87	0.941875743538997	0.058	0.02	2.8317574060504e-83	no change
1415	1415	SART1	1.29987390823424e-87	0.316778449919371	0.15	0.084	2.83944456514687e-83	no change
1416	1416	UBE3A	1.31643036852802e-87	0.163100059054427	0.29	0.191	2.87561049701261e-83	no change
1417	1417	CEP70	1.33452465513354e-87	0.408963456886558	0.189	0.115	2.91513565667371e-83	no change
1418	1418	FRK	1.52575474819227e-87	0.547560448296754	0.094	0.044	3.3328586719512e-83	no change
1419	1419	RNPEP	2.08314430179049e-87	0.197215300421503	0.302	0.202	4.55042041283114e-83	no change
1420	1420	BRWD1	2.44277409189001e-87	0.235677146871776	0.294	0.196	5.33599572632453e-83	no change
1421	1421	MCL1	2.48514635002664e-87	-0.406949227	0.695	0.691	5.42855368699819e-83	no change
1422	1422	TCEB3	2.66464103195546e-87	0.314837293466027	0.208	0.129	5.82064187020351e-83	no change
1423	1423	HMGA1	2.88715582873462e-87	0.186000664995162	0.666	0.524	6.3067031922879e-83	no change
1424	1424	ZNF664	3.41255124869363e-87	0.321067060197251	0.209	0.13	7.45437694764638e-83	no change
1425	1425	CTNND1	3.70414093251437e-87	0.139736854246406	0.523	0.382	8.09132545298438e-83	no change

1426	1426	RBFA	3.76689555533447e-87	0.261500309206045	0.227	0.142	8.22840665107262e-83	no change
1427	1427	SST	3.99220513844487e-87	1.61841580658355	0.054	0.018	8.72057290441896e-83	up
1428	1428	RB1CC1	4.08846709202165e-87	0.199300241718136	0.25	0.16	8.93084751581209e-83	no change
1429	1429	ITGB4	4.27565280973581e-87	0.181140501508908	0.301	0.2	9.33973599758691e-83	no change
1430	1430	MMADHC	4.5539933722851e-87	0.178444733245242	0.641	0.484	9.94774312241957e-83	no change
1431	1431	ARL6IP6	5.34366405960295e-87	0.222410031742609	0.34	0.235	1.16726997717967e-82	no change
1432	1432	MCM4	5.55564337281858e-87	0.592662870387372	0.099	0.047	1.21357473835849e-82	no change
1433	1433	CUL5	6.00260781022497e-87	0.244221074682331	0.187	0.111	1.31120965006554e-82	no change
1434	1434	MBIP	6.01979006158039e-87	0.380232282879349	0.162	0.094	1.31496294105162e-82	no change
1435	1435	NCOR1	6.17530754550324e-87	0.202024306542235	0.42	0.298	1.34893418023973e-82	no change
1436	1436	STRBP	7.31160271996497e-87	0.62194880250929	0.094	0.044	1.59714649814915e-82	no change
1437	1437	KANSL1	7.40031842399008e-87	0.288749407873363	0.187	0.112	1.61652555653639e-82	no change
1438	1438	DRG1	7.90252495249368e-87	0.241443425774323	0.274	0.18	1.72622755062272e-82	no change

1439	1439	HLA-DPA1	8.1951577691982e-87	-1.028346564	0.276	0.339	1.79015026310366e-82	down
1440	1440	PRKX	8.92291666048474e-87	0.804854443841959	0.065	0.025	1.94912191531629e-82	no change
1441	1441	NOL8	1.05408716650438e-86	0.500250584866378	0.096	0.045	2.30254800651217e-82	no change
1442	1442	RUVBL2	1.06445283121819e-86	0.205864042812083	0.265	0.172	2.32519076451302e-82	no change
1443	1443	POGLUT1	1.32521244551257e-86	0.233933722630899	0.135	0.072	2.89479406597765e-82	no change
1444	1444	TEPP	1.37957454407312e-86	0.646518347973058	0.08	0.034	3.01354263407332e-82	no change
1445	1445	LYRM7	1.444545814055e-86	0.252717757729457	0.172	0.101	3.15546587622175e-82	no change
1446	1446	EFHD1	1.5884052192113e-86	-2.865775603	0.008	0.036	3.46971236084517e-82	down
1447	1447	PRR14L	1.70720369061482e-86	0.463571113177624	0.123	0.065	3.72921574177902e-82	no change
1448	1448	MFAP5	1.79486401695133e-86	-3.069903045	0.018	0.052	3.92070095862849e-82	down
1449	1449	SRSF8	1.8977452676097e-86	0.122828225353663	0.371	0.256	4.14543476256663e-82	no change
1450	1450	ARID4B	1.94897208547975e-86	0.15617548030043	0.425	0.299	4.25733462352197e-82	no change
1451	1451	YLPM1	2.11570511289662e-86	0.40117948433151	0.146	0.081	4.62154624861137e-82	no change

1452	1452	FOXC1	2.1289676943193e-86	0.337988018087238	0.173	0.102	4.65051703147109e-82	no change
1453	1453	AP2M1	2.2058019426275e-86	0.196480664743181	0.759	0.611	4.81835376347551e-82	no change
1454	1454	MRPS26	2.65462296604612e-86	0.228101861147591	0.569	0.435	5.79875840703114e-82	no change
1455	1455	IARS2	2.84407406571299e-86	0.240423804496591	0.252	0.164	6.21259538914347e-82	no change
1456	1456	SNRPB	2.91524460059806e-86	0.277579841921558	0.769	0.636	6.3680603055464e-82	no change
1457	1457	SLC25A24	3.10183482352524e-86	0.223647540603043	0.244	0.157	6.77564798850852e-82	no change
1458	1458	LYPLA2	3.26377057120754e-86	0.277801236118571	0.38	0.272	7.12938043574576e-82	no change
1459	1459	TMEM246	3.70581044895046e-86	0.819783026992313	0.068	0.027	8.09497234468738e-82	no change
1460	1460	ZNF322	3.78867591808796e-86	0.416120979945231	0.16	0.093	8.27598367547133e-82	no change
1461	1461	OTUB1	3.94993317576422e-86	0.191589808323388	0.453	0.326	8.62823402913936e-82	no change
1462	1462	PAK1	4.24520054906944e-86	0.257415188807568	0.214	0.133	9.27321607938729e-82	no change
1463	1463	ATP5F1	4.27068299144109e-86	0.190073227013637	0.704	0.553	9.32887992650392e-82	no change
1464	1464	MRPL35	5.12732656436261e-86	0.286935830580422	0.214	0.134	1.12001321471937e-81	no change

1465	1465	SNRNP70	5.49701989589412e-86	0.16243593137304	0.491	0.354	1.20076902605911e-81	no change
1466	1466	CYSTM1	5.71189133265288e-86	-0.32294768	0.653	0.669	1.2477055427047e-81	no change
1467	1467	KRT16	6.56144099868798e-86	-1.047168521	0.17	0.234	1.4332811717534e-81	down
1468	1468	TRPM4	6.68324526689653e-86	0.60593443012784	0.197	0.126	1.45988809610088e-81	no change
1469	1469	POLR2L	6.93496028800274e-86	-0.234658114	0.966	0.938	1.51487272531132e-81	no change
1470	1470	UBAP2L	7.09397575575647e-86	0.296005100528558	0.174	0.102	1.54960806408744e-81	no change
1471	1471	PRIM1	7.58040763579814e-86	0.655402472613508	0.073	0.03	1.65586424396375e-81	no change
1472	1472	RABGAP1	7.64761640567514e-86	0.51256043080642	0.113	0.058	1.67054532765568e-81	no change
1473	1473	PTPRF	7.74270156630141e-86	0.171598930185606	0.436	0.311	1.69131573014288e-81	no change
1474	1474	LAPTM4B	8.03124663756364e-86	0.163727576736172	0.364	0.249	1.7543455155094e-81	no change
1475	1475	DCTN6	8.66052672442463e-86	0.107537437160718	0.373	0.255	1.89180545768332e-81	no change
1476	1476	ALG3	8.69027828623088e-86	0.19790260053552	0.297	0.2	1.89830438884427e-81	no change
1477	1477	ATOH8	1.06459145270772e-85	0.327259852214166	0.196	0.119	2.32549356929474e-81	no change

1478	1478	TMA16	1.19846567941985e-85	0.121913627855574	0.321	0.216	2.61792843012471e-81	no change
1479	1479	ATAD3A	1.30899024627156e-85	0.43924267842998	0.135	0.074	2.85935829395559e-81	no change
1480	1480	PBK	1.52047500251875e-85	1.33033060041005	0.045	0.013	3.32132559550195e-81	up
1481	1481	PCNP	1.58569260355822e-85	0.176393444207305	0.687	0.529	3.46378692321258e-81	no change
1482	1482	RASSF10	1.6224768693365e-85	0.652545362985841	0.084	0.037	3.54413847337866e-81	no change
1483	1483	DLD	1.67757127614267e-85	0.207446547637479	0.316	0.214	3.66448669560605e-81	no change
1484	1484	THAP4	1.69862407087911e-85	0.194899240471105	0.296	0.197	3.71047442042833e-81	no change
1485	1485	UMPS	1.93770444366842e-85	0.403559754378096	0.119	0.062	4.23272158674929e-81	no change
1486	1486	UGGT2	1.98765011969815e-85	0.431232243931982	0.108	0.054	4.34182292146865e-81	no change
1487	1487	LIN7C	2.42250910948532e-85	0.312765082337334	0.165	0.096	5.29172889875974e-81	no change
1488	1488	TSPAN14	2.43608806875265e-85	0.181447521688096	0.383	0.267	5.3213907773833e-81	no change
1489	1489	RRS1	2.46718517076185e-85	0.334570548155852	0.179	0.107	5.38931928701218e-81	no change
1490	1490	TFB2M	2.73340503080466e-85	0.361926823733405	0.134	0.073	5.97084994928969e-81	no change

1491	1491	PPIL4	2.959179641423e-85	0.207766401519415	0.214	0.132	6.4640320087244e-81	no change
1492	1492	NCKAP1	3.16813370260918e-85	0.117354701781322	0.395	0.274	6.92047125997949e-81	no change
1493	1493	NFKBIA	3.31542790268693e-85	-0.539475154	0.823	0.8	7.24222071062933e-81	no change
1494	1494	RRAGA	3.45057892433651e-85	0.176319209408427	0.343	0.234	7.53744460232068e-81	no change
1495	1495	USP5	3.50770412318827e-85	0.373486242413557	0.14	0.077	7.66222888669246e-81	no change
1496	1496	CLIC4	4.68194112157784e-85	-1.179138592	0.079	0.136	1.02272321859746e-80	down
1497	1497	SP1	5.06463757881844e-85	0.304460718276802	0.206	0.128	1.1063194327171e-80	no change
1498	1498	SEPT2	5.17654819866837e-85	0.127482088263858	0.528	0.384	1.13076518851712e-80	no change
1499	1499	ENOSF1	5.78615203430953e-85	0.328393827138541	0.201	0.125	1.26392705037457e-80	no change
1500	1500	ZFP14	6.31538030844983e-85	0.549507529726425	0.118	0.061	1.37953167457778e-80	no change
1501	1501	PSMD1	6.83581043485599e-85	0.215127185637552	0.225	0.143	1.49321443138994e-80	no change
1502	1502	PCOLCE2	7.42217095890807e-85	-2.552655636	0.028	0.067	1.62129902426388e-80	down
1503	1503	TKT	8.53152425820096e-85	0.180056157773323	0.622	0.47	1.86362615896142e-80	no change

1504	1504	SERPINE1	9.126732276896e-85	-0.910647994	0.176	0.244	1.99364339856516e-80	no change
1505	1505	RALGDS	9.49378667220647e-85	0.295625381518922	0.178	0.106	2.07382276067678e-80	no change
1506	1506	CLTC	9.66239669808015e-85	0.149979106739082	0.423	0.3	2.11065393472863e-80	no change
1507	1507	NANS	1.08744536537429e-84	0.131044408489484	0.333	0.225	2.3754156561236e-80	no change
1508	1508	CMTM3	1.16950308228426e-84	0.150081560998077	0.146	0.08	2.55466253294173e-80	no change
1509	1509	TERF1	1.34135611395865e-84	0.209902222482952	0.242	0.155	2.93005829533128e-80	no change
1510	1510	SOX4	1.38186029161424e-84	0.157683887798791	0.79	0.66	3.01853562100214e-80	no change
1511	1511	GPI	1.46481960933158e-84	0.195877215979152	0.354	0.244	3.1997519546239e-80	no change
1512	1512	DTYMK	1.48419552506727e-84	0.429031085058935	0.263	0.177	3.24207670495694e-80	no change
1513	1513	ZNF32	1.60990890182444e-84	0.266651510207102	0.268	0.177	3.5166850051453e-80	no change
1514	1514	AP1S1	1.6241523381099e-84	0.184811571452556	0.576	0.432	3.54779836736726e-80	no change
1515	1515	SMARCA1	1.67301540908787e-84	0.149985805991614	0.231	0.145	3.65453485961153e-80	no change
1516	1516	ALDH6A1	1.89237866720383e-84	0.269443274455338	0.148	0.083	4.13371196064005e-80	no change

1517	1517	SPATA20	2.00261217350082e-84	0.239518547320087	0.24	0.154	4.37450603179519e-80	no change
1518	1518	CBY1	2.18605618255081e-84	0.307588619338261	0.232	0.149	4.77522112516398e-80	no change
1519	1519	RPRD2	2.18890350256291e-84	0.295006717236359	0.143	0.079	4.78144081099842e-80	no change
1520	1520	PDXDC1	2.2179448224027e-84	0.261481783289112	0.235	0.151	4.84487867005646e-80	no change
1521	1521	LUC7L3	2.31491553246001e-84	0.205636489490385	0.562	0.426	5.05670148910564e-80	no change
1522	1522	CARNMT1	2.67273389766907e-84	0.50694228559424	0.096	0.046	5.83831992606831e-80	no change
1523	1523	ALG1L	2.73143343504562e-84	0.178234410552798	0.267	0.175	5.96654319551366e-80	no change
1524	1524	SMIM15	2.75983197134576e-84	0.147979587710634	0.467	0.333	6.02857695820767e-80	no change
1525	1525	CLIP1	2.83287216495627e-84	0.17364129903525	0.347	0.24	6.18812595713047e-80	no change
1526	1526	DDIT3	2.98111664417189e-84	-0.89338431	0.305	0.363	6.51195119752907e-80	no change
1527	1527	RFC2	3.01348997159359e-84	0.45284000511779	0.128	0.069	6.58266749394905e-80	no change
1528	1528	CCNDBP1	3.01863308102285e-84	0.106014941934269	0.403	0.279	6.59390210218631e-80	no change
1529	1529	UBFD1	3.04946955851208e-84	0.483173045177996	0.104	0.052	6.66126130361379e-80	no change

1530	1530	BEX2	3.12107148269125e-84	0.214403988221206	0.35	0.243	6.81766854679076e-80	no change
1531	1531	PCMT1	3.65892651095e-84	0.154589331897539	0.388	0.271	7.99255907051919e-80	no change
1532	1532	NAE1	4.54341816697781e-84	0.161628303803543	0.295	0.196	9.92464264394633e-80	no change
1533	1533	NCBP2-AS2	4.68529632537706e-84	0.247425660911502	0.348	0.245	1.02345612931536e-79	no change
1534	1534	CD96	4.73007331964769e-84	0.641859363839143	0.096	0.046	1.03323721594384e-79	no change
1535	1535	QDPR	5.13805664272716e-84	0.223293237373509	0.229	0.145	1.12235709303732e-79	no change
1536	1536	FN3K	5.3533638737572e-84	0.457960236514519	0.121	0.063	1.16938880458352e-79	no change
1537	1537	HNRNPH2	5.53702259098253e-84	0.126104576840208	0.412	0.288	1.20950721477422e-79	no change
1538	1538	FTSJ3	5.57090195645507e-84	0.329256043904432	0.156	0.089	1.21690782336804e-79	no change
1539	1539	LINC01106	5.60776495776574e-84	0.831016010194046	0.067	0.026	1.22496017737435e-79	no change
1540	1540	USP15	6.10897755638975e-84	0.16495391014311	0.232	0.147	1.33444505741778e-79	no change
1541	1541	TROVE2	6.58817411381856e-84	0.17410626967463	0.282	0.188	1.43912075342253e-79	no change
1542	1542	CLCN3	7.21788110996498e-84	0.147618878440177	0.34	0.232	1.57667394966075e-79	no change

1543	1543	RNF128	7.34005165768052e-84	0.242549140238475	0.307	0.209	1.60336088410373e-79	no change
1544	1544	NAA15	7.57856553449388e-84	0.178194365854426	0.21	0.13	1.65546185535484e-79	no change
1545	1545	CECR5	8.43930757055468e-84	0.333408363311435	0.204	0.127	1.84348234571197e-79	no change
1546	1546	SH3PXD2A	1.09673978769188e-83	0.237662301098864	0.158	0.09	2.39571839223414e-79	no change
1547	1547	YIPF5	1.12834606196694e-83	0.118169425973429	0.255	0.164	2.46475913776059e-79	no change
1548	1548	WDR36	1.17218056492792e-83	0.32927053914386	0.113	0.058	2.56051122602854e-79	no change
1549	1549	PDCL3	1.25634871699841e-83	0.186020820639153	0.333	0.227	2.74436813741132e-79	no change
1550	1550	ATP5O	1.47869638595806e-83	0.146020273617898	0.906	0.805	3.23006438548678e-79	no change
1551	1551	RSL24D1	1.5585108120873e-83	0.104051424219017	0.792	0.635	3.4044110179235e-79	no change
1552	1552	SULT2A1	1.59565742819843e-83	-1.066417157	0.064	0.117	3.48555408615665e-79	down
1553	1553	PEF1	1.74192140722441e-83	0.226322481698825	0.294	0.197	3.80505312194099e-79	no change
1554	1554	TCEAL1	1.91059599404916e-83	0.12821062999042	0.359	0.247	4.17350588940098e-79	no change
1555	1555	PLCD3	1.92268434386114e-83	0.262628955666349	0.247	0.161	4.19991168073028e-79	no change

1556	1556	DHPS	2.18979688438533e-83	0.155569438758724	0.344	0.236	4.78339231425132e-79	no change
1557	1557	ZDHHC12	2.34189361625498e-83	0.203822457456566	0.384	0.271	5.11563241534737e-79	no change
1558	1558	RNF126	2.42516592556291e-83	0.205241753765657	0.338	0.233	5.29753244779962e-79	no change
1559	1559	MRPL19	2.50570782416132e-83	0.242199465631488	0.279	0.186	5.47346817109799e-79	no change
1560	1560	NNT-AS1	2.5414423470752e-83	0.436543062930888	0.178	0.108	5.55152666295106e-79	no change
1561	1561	SOD2	2.64005326393324e-83	-0.976471341	0.515	0.523	5.76693234973578e-79	no change
1562	1562	ASPH	2.64801931620117e-83	0.132111502648085	0.584	0.437	5.78433339430983e-79	no change
1563	1563	BICD2	3.06480275478667e-83	0.460694220185016	0.095	0.046	6.69475513755601e-79	no change
1564	1564	SDC4	3.36940304138623e-83	-0.44321387	0.527	0.566	7.36012400360408e-79	no change
1565	1565	FCHO1	3.97888100358573e-83	0.604103013387965	0.124	0.067	8.69146766423267e-79	no change
1566	1566	DNTTIP2	4.09273582149897e-83	0.155697767514982	0.247	0.158	8.94017212848234e-79	no change
1567	1567	DPP10-AS1	4.17672997167613e-83	1.26520226990491	0.046	0.014	9.12364895012934e-79	up
1568	1568	HNRNPH3	4.36919351811813e-83	0.155124304369235	0.534	0.391	9.54406632097724e-79	no change

1569	1569	NIPBL	4.83559495332132e-83	0.215146478943602	0.241	0.155	1.05628736160351e-78	no change
1570	1570	MAVS	5.0441475430843e-83	0.262551658906229	0.165	0.097	1.10184358931133e-78	no change
1571	1571	RNASEH2C	6.33743842982995e-83	0.13071222025425	0.496	0.359	1.38435005061205e-78	no change
1572	1572	TRIM14	6.62506864786487e-83	0.574699765936901	0.077	0.033	1.4471799954396e-78	no change
1573	1573	SPAG7	7.6306799990156e-83	0.114108654301171	0.45	0.319	1.66684573898497e-78	no change
1574	1574	PBDC1	7.7098864579664e-83	0.163534343818025	0.307	0.207	1.68414759787818e-78	no change
1575	1575	KIAA0368	7.85540469510293e-83	0.419997544075549	0.1	0.049	1.71593460159828e-78	no change
1576	1576	PFKM	9.894668634671e-83	0.448333713651101	0.122	0.065	2.16139141655753e-78	no change
1577	1577	CDKN3	1.21916083387989e-82	1.27802624687736	0.066	0.026	2.66313492552724e-78	up
1578	1578	RPAP2	1.24083724052194e-82	0.433177466342822	0.111	0.057	2.71048486819612e-78	no change
1579	1579	CLDND1	1.29974812294823e-82	0.126471512503629	0.294	0.196	2.83916979976812e-78	no change
1580	1580	ABCF1	1.31278812672864e-82	0.204487674013028	0.397	0.284	2.86765438402604e-78	no change
1581	1581	TM9SF2	1.365684060123e-82	0.118551034551661	0.468	0.335	2.98320026093267e-78	no change

1582	1582	USP9X	1.37986316584693e-82	0.187555829865825	0.226	0.143	3.01417309947603e-78	no change
1583	1583	PRDM2	1.47962377383828e-82	0.173022115075918	0.272	0.18	3.23209017157235e-78	no change
1584	1584	ELAC2	1.65465952474859e-82	0.528381603533687	0.119	0.063	3.61443826586083e-78	no change
1585	1585	CAPG	1.71269604661884e-82	-0.422850951	0.579	0.604	3.74121324423419e-78	no change
1586	1586	ADAR	1.93296285410609e-82	0.248567807602275	0.354	0.249	4.22236405850934e-78	no change
1587	1587	OGN	1.94635251056742e-82	-2.110305002	0.028	0.067	4.25161242408347e-78	down
1588	1588	ATP5C1	2.53822282258222e-82	0.142058694754357	0.822	0.691	5.5444939336486e-78	no change
1589	1589	MRFAP1L1	2.72189374092243e-82	0.209029854502542	0.238	0.153	5.94570468767097e-78	no change
1590	1590	PRDX3	2.99875790588351e-82	0.190467645465322	0.568	0.423	6.55048676961195e-78	no change
1591	1591	C1orf131	3.18082761930012e-82	0.218030088144056	0.216	0.136	6.94819985159919e-78	no change
1592	1592	CCBL2	3.22057943054667e-82	0.297010518934562	0.167	0.099	7.03503370808615e-78	no change
1593	1593	FXYD1	3.30437201085562e-82	-1.645518703	0.044	0.089	7.21807022051301e-78	down
1594	1594	TMEM256	3.60587102830702e-82	0.158967718953141	0.649	0.496	7.87666467423386e-78	no change

1595	1595	PURB	3.64790231197172e-82	0.216541132488417	0.2	0.124	7.96847781027103e-78	no change
1596	1596	PABPC4	3.82742889455559e-82	0.146584333481411	0.455	0.325	8.36063567726723e-78	no change
1597	1597	CPSF3	4.00853934315192e-82	0.387451720433723	0.126	0.068	8.75625334118106e-78	no change
1598	1598	DNAJA3	4.33132541927177e-82	0.419804874932706	0.151	0.088	9.46134724585726e-78	no change
1599	1599	FAM215B	4.95975122334792e-82	0.615432750524751	0.069	0.028	1.08340805722812e-77	no change
1600	1600	ARHGEF10L	5.01527359935563e-82	0.322291637632998	0.145	0.081	1.09553636504324e-77	no change
1601	1601	QTRTD1	5.62919982953165e-82	0.491293587288638	0.087	0.04	1.22964241076289e-77	no change
1602	1602	C11orf1	6.29234659839277e-82	0.158324507222603	0.391	0.275	1.37450019095292e-77	no change
1603	1603	CPNE1	7.18943939564602e-82	0.148324321478097	0.356	0.246	1.57046114158492e-77	no change
1604	1604	CMIP	7.30156910926902e-82	0.102769084147037	0.294	0.196	1.59495475622873e-77	no change
1605	1605	LAP3	7.33784096712577e-82	0.185484135615577	0.259	0.168	1.60287798085895e-77	no change
1606	1606	ACSL5	7.3748980743196e-82	0.171856307083827	0.35	0.243	1.61097273535437e-77	no change
1607	1607	CRK	8.34348048686844e-82	0.170725216041412	0.265	0.174	1.82254987755154e-77	no change

1608	1608	PWP1	8.41114832554362e-82	0.159788363303846	0.298	0.2	1.83733124023175e-77	no change
1609	1609	DSG2	8.72069542676168e-82	0.179926335018336	0.376	0.267	1.90494870902182e-77	no change
1610	1610	IVD	9.70459157450571e-82	0.277243763258245	0.171	0.101	2.11987098353503e-77	no change
1611	1611	TTF1	1.18085837147511e-81	0.339131820305892	0.142	0.08	2.57946702665022e-77	no change
1612	1612	RASEF	1.18955792824375e-81	0.332869942435865	0.159	0.093	2.59847033845564e-77	no change
1613	1613	DENR	1.28606114100362e-81	0.184674195553523	0.418	0.301	2.80927195640831e-77	no change
1614	1614	ANXA3	1.31508288222094e-81	-1.334765086	0.053	0.102	2.87266704792343e-77	down
1615	1615	PCSK1N	1.33149306632095e-81	0.807185865646721	0.061	0.023	2.90851345407149e-77	no change
1616	1616	SPARC	1.44523241333383e-81	-1.350401545	0.063	0.113	3.15696568368641e-77	down
1617	1617	CHCHD4	1.44535833270034e-81	0.435076042430343	0.133	0.074	3.15724074195062e-77	no change
1618	1618	ATXN2	1.45294962996453e-81	0.261371514423185	0.213	0.134	3.17382317169453e-77	no change
1619	1619	CIAPIN1	1.54797976194359e-81	0.344679356250231	0.186	0.114	3.38140699198958e-77	no change
1620	1620	KIF20B	1.55705726679485e-81	0.790629323772643	0.069	0.028	3.40123589358667e-77	no change

1621	1621	FAT2	1.55849694007164e-81	0.711362532449186	0.057	0.02	3.4043807158925e-77	no change
1622	1622	RPS6KA5	1.56299787161927e-81	0.320377473915566	0.139	0.077	3.41421255076513e-77	no change
1623	1623	NF1	1.57074851796264e-81	0.18882201290308	0.265	0.174	3.43114306263759e-77	no change
1624	1624	EDARADD	1.68869258004711e-81	0.320334707583117	0.191	0.118	3.68878007185491e-77	no change
1625	1625	TGS1	1.73357275780044e-81	0.21837900711414	0.208	0.13	3.78681633213927e-77	no change
1626	1626	ZDHHC21	1.78592438202995e-81	0.335508331883104	0.144	0.081	3.90117322010623e-77	no change
1627	1627	ANKRD50	1.97992588749583e-81	0.254334223542347	0.164	0.096	4.32495010864589e-77	no change
1628	1628	TMBIM6	2.01061200648551e-81	0.22461848477848	0.912	0.811	4.39198086696694e-77	no change
1629	1629	VMA21	2.12811140518966e-81	0.184570338076503	0.281	0.188	4.6486465534963e-77	no change
1630	1630	LYPLA1	2.23641690089084e-81	0.188561726974977	0.793	0.638	4.88522907830594e-77	no change
1631	1631	DNAJC16	2.25729797787484e-81	0.325005823620165	0.15	0.086	4.9308417028698e-77	no change
1632	1632	RRP1B	2.33199348920679e-81	0.481724790614951	0.12	0.064	5.09400657782331e-77	no change
1633	1633	MRPS31	2.47670372230124e-81	0.170600334984194	0.251	0.162	5.41011161099482e-77	no change

1634	1634	PREP	2.61798724284141e-81	0.381997687494436	0.124	0.067	5.71873133326277e-77	no change
1635	1635	EHMT1	2.81834314765281e-81	0.323573261115099	0.138	0.077	6.1563887717328e-77	no change
1636	1636	H2AFV	3.03111403876133e-81	0.34159807482933	0.581	0.449	6.62116550627025e-77	no change
1637	1637	TAF1	3.06174004482851e-81	0.450473580671932	0.095	0.046	6.68806495392341e-77	no change
1638	1638	ZNF605	3.1786222263054e-81	0.701569044374798	0.083	0.038	6.94338239114152e-77	no change
1639	1639	ANKRD17	3.23245987974753e-81	0.190066422608586	0.223	0.141	7.06098536132051e-77	no change
1640	1640	MINA	3.33895136260548e-81	0.322269884391039	0.135	0.075	7.29360535647541e-77	no change
1641	1641	USP10	3.41127384342152e-81	0.229665142458909	0.172	0.102	7.45158658356996e-77	no change
1642	1642	FZD6	4.04528951038024e-81	0.213634797737743	0.235	0.152	8.83653040647459e-77	no change
1643	1643	FRYL	4.32948899710516e-81	0.292495922607785	0.173	0.103	9.4573357652765e-77	no change
1644	1644	USP7	4.40915681914418e-81	0.239869798772483	0.182	0.11	9.63136215573854e-77	no change
1645	1645	MRPL9	4.71796329007176e-81	0.165936415866426	0.365	0.254	1.03059190108328e-76	no change
1646	1646	AGO3	5.09852691263891e-81	0.177281049239068	0.165	0.097	1.11372221879684e-76	no change

1647	1647	TKFC	5.13787035757604e-81	0.330299244390062	0.149	0.085	1.12231640090891e-76	no change
1648	1648	KLHDC3	5.51504642628966e-81	0.274389173105871	0.241	0.157	1.20470674135871e-76	no change
1649	1649	AHSA1	5.55637943195206e-81	0.123197294689301	0.424	0.3	1.21373552311561e-76	no change
1650	1650	TIPIN	6.29777341013284e-81	0.572249348421326	0.083	0.038	1.37568562370942e-76	no change
1651	1651	DCUN1D5	6.31958092471463e-81	0.194484667728025	0.349	0.243	1.38044925719466e-76	no change
1652	1652	HERC2	1.17582842771924e-80	0.306696600553208	0.158	0.093	2.56847961750992e-76	no change
1653	1653	ACTR3	1.22105182334111e-80	0.149617371303199	0.625	0.469	2.66726560290632e-76	no change
1654	1654	VPS72	1.24196583416104e-80	0.244390514895129	0.25	0.164	2.71295016814138e-76	no change
1655	1655	TACC3	1.60720228103126e-80	1.32891236175315	0.042	0.012	3.5107726626847e-76	up
1656	1656	HMGCS1	1.61377734444069e-80	-0.835155467	0.32	0.373	3.52513523119625e-76	no change
1657	1657	PTRHD1	1.61583374782439e-80	0.154887216420319	0.711	0.555	3.5296272387476e-76	no change
1658	1658	MRFAP1	1.77037509762983e-80	0.150061709519076	0.611	0.458	3.86720736326261e-76	no change
1659	1659	FZD3	1.78986951767656e-80	0.405177815885462	0.133	0.074	3.90979097441268e-76	no change

1660	1660	ERP27	2.28737893610374e-80	-0.94274912	0.246	0.302	4.99655054802502e-76	no change
1661	1661	XAB2	2.63947249315981e-80	0.320963142788881	0.22	0.143	5.76566371405828e-76	no change
1662	1662	CYP3A5	2.71883346533714e-80	-0.881315969	0.26	0.318	5.93901982168246e-76	no change
1663	1663	NDUFA4L2	2.96299863645884e-80	0.747018754410042	0.129	0.073	6.47237422148068e-76	no change
1664	1664	NAA50	3.14690039993957e-80	0.173405398503265	0.484	0.355	6.87408923362799e-76	no change
1665	1665	SMARCA2	3.22922958455118e-80	0.20645356751631	0.167	0.098	7.05392910449361e-76	no change
1666	1666	GORASP2	3.36840399761195e-80	0.165942396462495	0.281	0.188	7.35794169238353e-76	no change
1667	1667	CFL1	3.66645177010671e-80	0.187480610848641	0.987	0.956	8.00899724662109e-76	no change
1668	1668	PRRC1	3.67437982210929e-80	0.261660114573381	0.164	0.097	8.02631528341554e-76	no change
1669	1669	WRAP73	3.95395718266454e-80	0.452056259751888	0.142	0.081	8.63702406981242e-76	no change
1670	1670	RP3-325F22.5	4.04191644874923e-80	0.355029407643226	0.118	0.063	8.82916229064781e-76	no change
1671	1671	GCAT	4.1645020359302e-80	0.274538870732639	0.181	0.11	9.09693824728593e-76	no change
1672	1672	LAMTOR4	4.17706222971757e-80	-0.225170291	0.902	0.852	9.12437473459506e-76	no change

1673	1673	ARPC5L	4.21680177279811e-80	0.159317656644071	0.447	0.322	9.2111817925002e-76	no change
1674	1674	ZNF131	4.39232718698704e-80	0.141517999228022	0.25	0.163	9.5945995072545e-76	no change
1675	1675	ARHGDIB	4.41424399982918e-80	0.137022427970383	0.735	0.584	9.64247459322685e-76	no change
1676	1676	NAT10	4.41485907887537e-80	0.552275900561252	0.083	0.038	9.64381817189536e-76	no change
1677	1677	ANKS3	4.58809502838656e-80	0.432829589308118	0.126	0.069	1.00222347800076e-75	no change
1678	1678	ECI2	4.93516421528411e-80	0.190456151470004	0.239	0.154	1.07803727118666e-75	no change
1679	1679	MYCNUT	5.20123671377872e-80	1.30418466629342	0.045	0.014	1.13615814775782e-75	up
1680	1680	OTUD6B-AS1	5.49846357414163e-80	0.112916356115434	0.375	0.262	1.2010843831355e-75	no change
1681	1681	TCOF1	5.8380994280501e-80	0.271083430953038	0.145	0.082	1.27527443906326e-75	no change
1682	1682	AAAS	5.87080149373435e-80	0.365127409724023	0.131	0.072	1.28241787829133e-75	no change
1683	1683	TIMM17B	6.0890095585547e-80	0.166189172848091	0.497	0.365	1.33008324797069e-75	no change
1684	1684	TPGS2	6.44348860871872e-80	0.17007095824066	0.452	0.325	1.40751565168852e-75	no change
1685	1685	WDR61	6.62311045205371e-80	0.139496583303948	0.387	0.27	1.44675224714661e-75	no change

1686	1686	ARL3	7.59330955912985e-80	0.16650639018929	0.343	0.238	1.65868254009632e-75	no change
1687	1687	GPD2	7.86706135653367e-80	0.29173595421527	0.19	0.118	1.71848088272121e-75	no change
1688	1688	LUC7L2	8.19067259139298e-80	0.121509620256127	0.338	0.232	1.78917052086388e-75	no change
1689	1689	THEM6	8.67550047314287e-80	0.226324360764719	0.402	0.29	1.89507632335333e-75	no change
1690	1690	UBE2T	8.83399812213641e-80	0.544260842971663	0.158	0.095	1.92969854979948e-75	no change
1691	1691	DAP3	9.44150602979924e-80	0.145933979470464	0.407	0.288	2.06240257714935e-75	no change
1692	1692	PGM2	9.44542027937852e-80	0.276834164808988	0.235	0.153	2.06325760582744e-75	no change
1693	1693	HOXA-AS2	9.73662791275565e-80	0.450194449901978	0.107	0.055	2.12686900126235e-75	no change
1694	1694	TSNAX	1.1239591890094e-79	0.153971544318308	0.253	0.166	2.45517645247213e-75	no change
1695	1695	ARHGEF12	1.21219468280175e-79	0.203694550538899	0.266	0.177	2.64791806511214e-75	no change
1696	1696	API5	1.25253852704331e-79	0.199463209829973	0.19	0.116	2.73604515847341e-75	no change
1697	1697	MAGOHB	1.44955814287423e-79	0.24383015890291	0.232	0.15	3.16641480729446e-75	no change
1698	1698	TFAP4	1.4609265556525e-79	0.564599606127455	0.081	0.036	3.19124796816731e-75	no change

1699	1699	NRM	1.46200667673499e-79	0.722133120161133	0.067	0.028	3.1936073846599e-75	no change
1700	1700	PRKCI	1.48325182868529e-79	0.179782652266359	0.196	0.121	3.24001529458016e-75	no change
1701	1701	SPOCK3	1.55637646302524e-79	1.46255064160704	0.038	0.01	3.39974874583233e-75	up
1702	1702	FUS	1.80668092886875e-79	0.14351880831065	0.698	0.547	3.94651382102091e-75	no change
1703	1703	CDCA5	1.86932389586885e-79	1.3572540903906	0.037	0.009	4.08335111813592e-75	up
1704	1704	SH3BP1	2.04500668730017e-79	0.235123293557103	0.247	0.162	4.46711260773848e-75	no change
1705	1705	ADSL	2.18427710421564e-79	0.25582294016154	0.176	0.105	4.77133490644864e-75	no change
1706	1706	CSTB	2.21586942327391e-79	-0.768804848	0.979	0.942	4.84034516819952e-75	no change
1707	1707	TTC37	2.33867267969396e-79	0.244640153488873	0.151	0.087	5.10859660152349e-75	no change
1708	1708	MTCH2	2.37094065464328e-79	0.171750118742194	0.489	0.358	5.17908276600278e-75	no change
1709	1709	MRS2	2.5275281851357e-79	0.343006027870503	0.206	0.131	5.52113256761043e-75	no change
1710	1710	UBE2K	2.64414017062536e-79	0.148280464374855	0.478	0.344	5.77585978871404e-75	no change
1711	1711	EIF1AX	2.69534624758648e-79	0.115225325556949	0.705	0.548	5.8877143432279e-75	no change

1712	1712	FAM192A	2.72473946391847e-79	0.171235245184083	0.329	0.227	5.95192088498351e-75	no change
1713	1713	H2AFY2	2.80214478268482e-79	0.306271913468086	0.228	0.148	6.12100506329673e-75	no change
1714	1714	PHF3	3.05760170602444e-79	0.23750748724143	0.18	0.11	6.67902516663978e-75	no change
1715	1715	PI16	3.21682315595285e-79	-2.425398851	0.045	0.088	7.02682850186341e-75	down
1716	1716	ZNF90	3.2998082723529e-79	0.222305513716313	0.161	0.094	7.20810119012768e-75	no change
1717	1717	CNOT2	4.55955055436885e-79	0.155492841305451	0.333	0.229	9.95988223096332e-75	no change
1718	1718	MARVELD2	4.9812859876375e-79	0.359175678784119	0.134	0.075	1.08811211113954e-74	no change
1719	1719	BTF3L4	5.39368004226326e-79	0.1871113220438	0.683	0.535	1.17819546843199e-74	no change
1720	1720	PTAR1	5.40707050512277e-79	0.346630117923586	0.118	0.063	1.18112048113902e-74	no change
1721	1721	HIGD1A	5.64295827520491e-79	-0.596818939	0.534	0.534	1.23264780563576e-74	no change
1722	1722	RP11-111M22.3	6.26352808217957e-79	0.528591283800032	0.097	0.049	1.36820507427131e-74	no change
1723	1723	GNL2	6.30785812237197e-79	0.251713415513538	0.182	0.11	1.37788852825093e-74	no change
1724	1724	ISY1	7.46382515436034e-79	0.285005433044937	0.178	0.108	1.63039796671847e-74	no change

1725	1725	PRELID2	7.56884673546336e-79	0.366409519035088	0.13	0.072	1.65333888089462e-74	no change
1726	1726	NIPAL4	7.77711477623346e-79	0.370264455550161	0.134	0.075	1.69883295172044e-74	no change
1727	1727	CNTNAP3B	7.9469869150767e-79	1.46646299861373	0.031	0.006	1.73593982172936e-74	up
1728	1728	POMP	8.50750906371402e-79	-0.21402163	0.907	0.854	1.85838027987769e-74	no change
1729	1729	CYYR1	8.96934482962915e-79	1.7592593214536	0.03	0.006	1.95926368458419e-74	up
1730	1730	WAPL	9.71842897001066e-79	0.253614057765071	0.177	0.108	2.12289362420913e-74	no change
1731	1731	PSMG2	1.00921614146107e-78	0.164422387551712	0.623	0.472	2.20453173940756e-74	no change
1732	1732	ITLN1	1.06230196418482e-78	-2.714836782	0.015	0.046	2.32049241056532e-74	down
1733	1733	GPATCH8	1.06932357845728e-78	0.133835298581316	0.314	0.213	2.33583042478207e-74	no change
1734	1734	SUDS3	1.10343130109572e-78	0.199124578684436	0.372	0.264	2.41033533411349e-74	no change
1735	1735	SAMD5	1.11151282793151e-78	2.13066229107982	0.026	0.004	2.4279886213336e-74	up
1736	1736	TACC2	1.13197780358661e-78	0.295171671281361	0.201	0.127	2.4726923141546e-74	no change
1737	1737	CNOT8	1.20987300180377e-78	0.175794970806336	0.198	0.123	2.64284658514016e-74	no change

1738	1738	BDP1	1.21246886067538e-78	0.209531554714993	0.217	0.138	2.6485169792593e-74	no change
1739	1739	SPSB3	1.21701163205352e-78	0.205367384180366	0.356	0.252	2.65844020905771e-74	no change
1740	1740	NAA35	1.2928628402722e-78	0.465088385062157	0.103	0.052	2.8241295882906e-74	no change
1741	1741	AQR	1.31434718981811e-78	0.293972758366821	0.128	0.07	2.87106000143868e-74	no change
1742	1742	CCNA2	1.41931432321384e-78	0.990536263685972	0.05	0.017	3.10035020762831e-74	no change
1743	1743	TGOLN2	1.41946559338303e-78	0.107072107484325	0.387	0.271	3.10068064218589e-74	no change
1744	1744	WBP5	1.4436754762881e-78	-0.264582278	0.715	0.734	3.15356471040373e-74	no change
1745	1745	ZNF302	1.54953250108876e-78	0.438568042716881	0.109	0.056	3.38479879537829e-74	no change
1746	1746	STT3A	1.55722823991211e-78	0.233204152929815	0.151	0.087	3.40160936726402e-74	no change
1747	1747	NME4	1.68276202199508e-78	0.173445833084005	0.545	0.405	3.67582536084606e-74	no change
1748	1748	APPBP2	1.71361853223124e-78	0.244510573843533	0.167	0.1	3.74322832180593e-74	no change
1749	1749	OFD1	1.80888469505248e-78	0.129056346484455	0.318	0.217	3.95132772787264e-74	no change
1750	1750	MRPS30	2.24799774183372e-78	0.247870557757938	0.214	0.136	4.91052626726158e-74	no change

1751	1751	TXN2	2.3687745612556e-78	0.222252031726084	0.64	0.5	5.17435115160674e-74	no change
1752	1752	KIAA2026	2.71147611212503e-78	0.229388423723095	0.168	0.101	5.92294841932592e-74	no change
1753	1753	MRPL28	2.7614218673088e-78	0.135417481527334	0.379	0.266	6.03204992694935e-74	no change
1754	1754	RBM26	3.19011212623598e-78	0.158729074664427	0.225	0.143	6.96848092854988e-74	no change
1755	1755	STON2	3.35557713750758e-78	0.338011144516306	0.146	0.085	7.32992269917157e-74	no change
1756	1756	COL4A3BP	4.32729244986769e-78	0.260958992387875	0.164	0.097	9.45253762749098e-74	no change
1757	1757	TFAM	4.3402239872837e-78	0.258572680752961	0.247	0.163	9.48078527782251e-74	no change
1758	1758	PPP2R1A	5.31475568200243e-78	0.157186684848171	0.455	0.33	1.16095523117661e-73	no change
1759	1759	PUM2	6.19501340058739e-78	0.179586042397374	0.239	0.156	1.35323872722431e-73	no change
1760	1760	DNAJC11	6.21642714894857e-78	0.509027332843468	0.11	0.058	1.35791634641633e-73	no change
1761	1761	KLHL23	6.36907973217505e-78	0.548311330429804	0.09	0.044	1.39126177669632e-73	no change
1762	1762	MED13	6.79783830443859e-78	0.203012686614958	0.234	0.152	1.48491979922157e-73	no change
1763	1763	GBP2	7.88853322881808e-78	0.139911384179417	0.386	0.272	1.72317119850302e-73	no change

1764	1764	CNTN3	8.18595238986678e-78	0.890673441813234	0.05	0.017	1.7881394400425e-73	no change
1765	1765	ATP9A	8.28548251054269e-78	0.259268920537505	0.136	0.077	1.80988079960295e-73	no change
1766	1766	ZBTB10	9.16980574892574e-78	0.270966816367552	0.129	0.071	2.00305236779534e-73	no change
1767	1767	HNRNPUL2	9.64953035752666e-78	0.219930734680737	0.206	0.131	2.10784341129812e-73	no change
1768	1768	ZBTB43	9.88859137404142e-78	0.247271805509507	0.178	0.108	2.16006389974561e-73	no change
1769	1769	HMOX2	1.08094902272134e-77	0.203879101399254	0.32	0.222	2.36122504523249e-73	no change
1770	1770	CDK9	1.15171162036607e-77	0.357441955834491	0.119	0.064	2.51579886352764e-73	no change
1771	1771	MDFIC	1.27178742771411e-77	0.267670440028225	0.145	0.083	2.77809245709871e-73	no change
1772	1772	EXOSC10	1.3683250177428e-77	0.471588979276141	0.113	0.06	2.98896916875737e-73	no change
1773	1773	HS6ST1	1.36958474496463e-77	0.398903831972006	0.131	0.073	2.99172091690074e-73	no change
1774	1774	TIMM8A	1.49130286796209e-77	0.347047651802385	0.151	0.088	3.25760198477638e-73	no change
1775	1775	UBXN1	1.55140218835413e-77	0.155917253264653	0.704	0.555	3.38888294024077e-73	no change
1776	1776	PIN4	2.01086244113329e-77	0.161629144451842	0.573	0.434	4.39252791641155e-73	no change

1777	1777	ANKRD11	2.02378506466859e-77	0.128666761622267	0.389	0.276	4.42075609526207e-73	no change
1778	1778	DPP10	2.09131331543865e-77	1.94073091215446	0.029	0.006	4.56826480624419e-73	up
1779	1779	UBE2G1	2.1810145519477e-77	0.209848797052449	0.273	0.184	4.76420818727456e-73	no change
1780	1780	HCFC1	2.51189678158758e-77	0.366386308555727	0.134	0.076	5.48698732969992e-73	no change
1781	1781	BRD8	2.59415751965592e-77	0.378275294448398	0.128	0.071	5.6666776859364e-73	no change
1782	1782	CEP55	2.73631876703929e-77	1.54116565881684	0.037	0.009	5.97721471472062e-73	up
1783	1783	NEMF	2.91183975828536e-77	0.121790356502878	0.309	0.21	6.36062276799855e-73	no change
1784	1784	DEPDC1	3.12106792974528e-77	1.46938834995431	0.035	0.008	6.81766078573559e-73	up
1785	1785	NUF2	3.36185425653951e-77	1.20489935050339	0.043	0.013	7.3436344379849e-73	up
1786	1786	GINS2	3.6628119715923e-77	0.701748773403163	0.086	0.041	8.00104647074621e-73	no change
1787	1787	PRPF38A	3.77430387629027e-77	0.159586477109405	0.276	0.184	8.24458938736846e-73	no change
1788	1788	UBXN4	3.85488284839854e-77	0.227201754475688	0.71	0.572	8.42060609404177e-73	no change
1789	1789	DNAJC10	3.94957693685973e-77	0.222632401021908	0.196	0.123	8.62745586087639e-73	no change

1790	1790	MGRN1	4.05270010213131e-77	0.306253373177627	0.153	0.09	8.85271810309562e-73	no change
1791	1791	TBRG4	4.95065563842461e-77	0.273636567536589	0.207	0.132	1.08142121765747e-72	no change
1792	1792	CH17-189H20.1	5.15736725082656e-77	0.142577224740167	0.213	0.136	1.12657530227055e-72	no change
1793	1793	PDCD11	6.42880699630053e-77	0.577637919270135	0.09	0.044	1.40430860027189e-72	no change
1794	1794	GPATCH4	6.77883471668715e-77	0.207434798571234	0.233	0.153	1.48076865551314e-72	no change
1795	1795	CMTM4	7.62244933265049e-77	0.623162299789825	0.073	0.032	1.66504783222417e-72	no change
1796	1796	RP11-4L24.3	7.73631461601542e-77	1.26388795204644	0.038	0.01	1.68992056472241e-72	up
1797	1797	AKR7A2	7.88195120820308e-77	0.20103239187827	0.467	0.346	1.72173342191988e-72	no change
1798	1798	ITPA	8.49277074378056e-77	0.145932547436134	0.498	0.366	1.85516084127142e-72	no change
1799	1799	MORC3	9.04211844639859e-77	0.257181093384298	0.17	0.102	1.97516035343131e-72	no change
1800	1800	MFSD14B	9.47462199892044e-77	0.213328910065591	0.189	0.118	2.06963642944418e-72	no change
1801	1801	C3orf58	9.73392499207046e-77	0.191997190757726	0.203	0.128	2.12627857526787e-72	no change
1802	1802	C5orf42	1.04668964364581e-76	0.599911722731319	0.08	0.037	2.28638885757991e-72	no change

1803	1803	SRRT	1.06100500385325e-76	0.268372225831117	0.174	0.106	2.31765933041703e-72	no change
1804	1804	RP11-480C16.1	1.13800059222971e-76	0.782684210991216	0.06	0.024	2.48584849366659e-72	no change
1805	1805	PRAME	1.25161518162893e-76	1.31002815176403	0.034	0.008	2.73402820275024e-72	up
1806	1806	CTPS1	1.26947662808269e-76	0.717595341088062	0.07	0.03	2.77304474638383e-72	no change
1807	1807	POLR2B	1.30784066141311e-76	0.122765516680937	0.233	0.151	2.8568471407908e-72	no change
1808	1808	AGGF1	1.31841072559604e-76	0.225537419330583	0.169	0.102	2.87993638899198e-72	no change
1809	1809	HIBCH	1.38374316059479e-76	0.109461559488852	0.323	0.218	3.02264856000326e-72	no change
1810	1810	SUPT3H	1.39199940634143e-76	0.509795940538444	0.084	0.04	3.04068350321222e-72	no change
1811	1811	ODF2L	1.51336184752165e-76	0.164596917141976	0.438	0.319	3.3057876197263e-72	no change
1812	1812	TARS	1.53787337204821e-76	0.264276930248205	0.198	0.125	3.35933059390211e-72	no change
1813	1813	PLP2	1.5774346647149e-76	0.161205478712394	0.627	0.485	3.44574828160323e-72	no change
1814	1814	SND1	1.79420883105016e-76	0.26132804570586	0.186	0.115	3.91926977054597e-72	no change
1815	1815	DDX6	1.82179238082537e-76	0.12039796930789	0.335	0.231	3.97952327667494e-72	no change

1816	1816	LRP11	1.95478579617441e-76	0.185274927637904	0.193	0.121	4.27003409316338e-72	no change
1817	1817	RIOK1	2.04732716751449e-76	0.255208523175234	0.145	0.084	4.47218146471864e-72	no change
1818	1818	ATP2C1	2.10596599244165e-76	0.192946027859284	0.255	0.169	4.60027211388955e-72	no change
1819	1819	GTPBP3	2.23090380660769e-76	0.399737689674919	0.123	0.068	4.87318627515384e-72	no change
1820	1820	TBC1D1	2.47114859827484e-76	0.287946310270182	0.255	0.172	5.39797699807157e-72	no change
1821	1821	MRPL42	2.49753325721244e-76	0.183582579604032	0.57	0.429	5.45561164705486e-72	no change
1822	1822	ALDH1L1	3.02605260433548e-76	0.237163906043424	0.278	0.19	6.61010930891042e-72	no change
1823	1823	TYSND1	3.17453832035101e-76	0.314403054742193	0.173	0.106	6.93446150697475e-72	no change
1824	1824	TPD52	3.37492809192722e-76	0.135612365140771	0.397	0.281	7.37219292400583e-72	no change
1825	1825	STX6	3.55633663528984e-76	0.19545750430655	0.212	0.136	7.76846174612713e-72	no change
1826	1826	C6orf48	3.5609785354161e-76	0.131395547847924	0.693	0.561	7.77860151276294e-72	no change
1827	1827	PAFAH1B1	3.58596052631446e-76	0.14319183737341	0.282	0.19	7.83317217368131e-72	no change
1828	1828	SHPRH	3.81267915710581e-76	0.388249946648627	0.115	0.062	8.32841635078193e-72	no change

1829	1829	RYK	4.02152953624998e-76	0.193929249664295	0.225	0.146	8.78462911898447e-72	no change
1830	1830	DCP2	4.11977354884722e-76	0.307582895091626	0.155	0.091	8.99923334010186e-72	no change
1831	1831	MAD2L2	4.33160291285595e-76	0.312503904776593	0.223	0.147	9.46195340284253e-72	no change
1832	1832	BRD4	4.40838531547063e-76	0.183496085313868	0.401	0.289	9.62967688311405e-72	no change
1833	1833	ST20	4.83317526660755e-76	0.691046521123173	0.06	0.024	1.05575880523775e-71	no change
1834	1834	RABL6	5.36323874795115e-76	0.325416207853606	0.132	0.075	1.17154587210245e-71	no change
1835	1835	GLE1	5.62769713947973e-76	0.691402216174559	0.066	0.027	1.22931416314795e-71	no change
1836	1836	ALG2	5.77655899824015e-76	0.279536518770901	0.145	0.084	1.26183154757558e-71	no change
1837	1837	UBA1	5.87505968565129e-76	0.124822058239755	0.285	0.191	1.28334803773367e-71	no change
1838	1838	FUBP3	5.90201289851596e-76	0.259259663786835	0.141	0.081	1.28923569755183e-71	no change
1839	1839	SEC61B	7.18308486324763e-76	0.162803701196615	0.796	0.665	1.56907305752781e-71	no change
1840	1840	ZNF891	7.5303695658565e-76	0.948185271316893	0.054	0.02	1.64493392796569e-71	no change
1841	1841	SORBS2	8.246321948852e-76	0.381088807471908	0.143	0.083	1.80132656650723e-71	no change

1842	1842	BRD7	8.73949622476951e-76	0.174848615709928	0.311	0.216	1.90905555533865e-71	no change
1843	1843	NCBP2	9.42394988768498e-76	0.154122411298395	0.397	0.284	2.05856761346591e-71	no change
1844	1844	TOP1	9.76918236387222e-76	0.118721162271354	0.483	0.353	2.13398019556425e-71	no change
1845	1845	KIF2C	9.88543995646834e-76	1.75019218610505	0.03	0.006	2.15937550409094e-71	up
1846	1846	CCND1	1.00302588285882e-75	-0.555119517	0.675	0.654	2.1910097385168e-71	no change
1847	1847	DSC3	1.03105877673887e-75	0.942023268766627	0.041	0.012	2.25224479190838e-71	no change
1848	1848	SYT17	1.04722322363257e-75	0.378059183562169	0.123	0.068	2.28755440970298e-71	no change
1849	1849	TRIM27	1.05226094131391e-75	0.191489558130797	0.273	0.184	2.29855880020611e-71	no change
1850	1850	IDH3B	1.09551935504493e-75	0.149654999661763	0.41	0.294	2.39305247916015e-71	no change
1851	1851	RALGAPA1	1.17661483390209e-75	0.25910185076793	0.157	0.093	2.57019744317573e-71	no change
1852	1852	CALD1	1.26654180153626e-75	-1.170133831	0.132	0.192	2.7666339112758e-71	down
1853	1853	SEMA5A	1.27087964958739e-75	0.274554693527953	0.235	0.155	2.7761095065587e-71	no change
1854	1854	EEF1G	1.27778081129095e-75	0.459907192305496	0.107	0.056	2.79118440418396e-71	no change

1855	1855	GOLPH3L	1.31540526037021e-75	0.245092333422966	0.176	0.108	2.87337125075269e-71	no change
1856	1856	RASIP1	1.31894392670375e-75	0.863044086352557	0.072	0.032	2.88110111349166e-71	no change
1857	1857	CYSRT1	1.35258208476842e-75	-1.346046147	0.078	0.129	2.95458030596814e-71	down
1858	1858	DPY19L4	1.354726779314e-75	0.329118317979596	0.125	0.069	2.95926517673349e-71	no change
1859	1859	PAK1IP1	1.37366847151866e-75	0.208333798675032	0.174	0.106	3.00064140918536e-71	no change
1860	1860	UCHL5	1.37383903639037e-75	0.288208165516657	0.155	0.092	3.00101399109113e-71	no change
1861	1861	RBM17	1.7048254559047e-75	0.180313880061473	0.556	0.421	3.72402072587822e-71	no change
1862	1862	CARD11	1.78163882967422e-75	0.420545889875944	0.093	0.046	3.89181185954036e-71	no change
1863	1863	VGLL4	2.07737781109901e-75	0.173068633921456	0.387	0.276	4.53782409056468e-71	no change
1864	1864	NCBP1	2.18093850671487e-75	0.52511871976292	0.078	0.036	4.76404207406795e-71	no change
1865	1865	TTC9C	2.26740081534755e-75	0.345164406296273	0.179	0.112	4.9529103410452e-71	no change
1866	1866	SFXN1	2.57888336831745e-75	0.309831351637504	0.2	0.128	5.63331282975263e-71	no change
1867	1867	NUP93	2.58659407262965e-75	0.273334853133206	0.131	0.074	5.6501560922522e-71	no change

1868	1868	IKZF2	2.61723390070188e-75	0.364920133931195	0.149	0.088	5.71708573269319e-71	no change
1869	1869	TMTC4	2.73279662582034e-75	0.428794721988071	0.089	0.043	5.96952094944195e-71	no change
1870	1870	GTF3C5	3.1164535597423e-75	0.299927837708781	0.132	0.074	6.80758115590109e-71	no change
1871	1871	SNRPN	3.15330637555884e-75	0.140937543004997	0.614	0.466	6.88808244677074e-71	no change
1872	1872	CST6	3.34339202186824e-75	-1.265479609	0.112	0.169	7.30330553256899e-71	down
1873	1873	PSMB2	3.58725138696456e-75	0.198842927634085	0.654	0.511	7.83599192968539e-71	no change
1874	1874	GABRG3	3.78436908041912e-75	1.33879236269423	0.035	0.009	8.26657581926752e-71	up
1875	1875	ACAP2	3.96850435073345e-75	0.21226416161019	0.23	0.152	8.66880090374215e-71	no change
1876	1876	FOPNL	4.04333953393732e-75	0.21454974705725	0.213	0.137	8.83227087793269e-71	no change
1877	1877	ATL2	4.2485630900783e-75	0.411340035097949	0.109	0.058	9.28056121396704e-71	no change
1878	1878	NMI	4.88231131063683e-75	0.254678980135814	0.204	0.129	1.06649208269551e-70	no change
1879	1879	MRPL2	4.90860142338582e-75	0.17652106073181	0.288	0.196	1.0722348949244e-70	no change
1880	1880	CTBP1	4.94055569921043e-75	0.171941075026116	0.312	0.217	1.07921498693553e-70	no change

1881	1881	HMGB2	5.32171076172163e-75	0.65040382136419	0.403	0.307	1.16247449879047e-70	no change
1882	1882	MSH3	5.81875438517907e-75	0.279031726723314	0.126	0.07	1.27104870789852e-70	no change
1883	1883	NR2C2AP	6.33584340670868e-75	0.307329696518485	0.165	0.1	1.38400163376144e-70	no change
1884	1884	RAP1GAP	6.57532473662177e-75	0.723777991728433	0.081	0.038	1.43631393546766e-70	no change
1885	1885	FGD5-AS1	6.65639441105587e-75	0.144686574215896	0.26	0.173	1.45402279515104e-70	no change
1886	1886	INADL	8.08841910336256e-75	0.206095165797799	0.214	0.138	1.76683426893852e-70	no change
1887	1887	ZFP36L2	8.20435496686671e-75	-0.390049167	0.744	0.723	1.79215929896236e-70	no change
1888	1888	YTHDC2	8.88387891735581e-75	0.338061517243098	0.12	0.066	1.9405945107072e-70	no change
1889	1889	TC2N	8.89511439834728e-75	0.104440093219892	0.246	0.162	1.94304878917498e-70	no change
1890	1890	SBSPON	9.1647878653188e-75	1.1565045544654	0.048	0.016	2.00195626130024e-70	up
1891	1891	PPIP5K2	1.08519451435773e-74	0.293902646579316	0.131	0.074	2.37049889716303e-70	no change
1892	1892	LMAN1	1.19179673832915e-74	0.110265932618712	0.405	0.289	2.6033607952062e-70	no change
1893	1893	RRN3	1.29089904264042e-74	0.331763085312085	0.129	0.072	2.81983986874373e-70	no change

1894	1894	CCDC50	1.34114513080804e-74	0.186032548539115	0.24	0.158	2.92959742373708e-70	no change
1895	1895	NNT	1.35654871002901e-74	0.396521053863868	0.124	0.069	2.96324500218738e-70	no change
1896	1896	FLAD1	1.37734156927866e-74	0.226892143386415	0.212	0.137	3.00866492393231e-70	no change
1897	1897	IKZF3	1.37999231197751e-74	0.669310778887619	0.063	0.026	3.01445520628368e-70	no change
1898	1898	NUBP2	1.40711691069988e-74	0.185433491399086	0.342	0.241	3.07370617973281e-70	no change
1899	1899	RNF20	1.51316549882156e-74	0.426066813453123	0.088	0.042	3.30535871562581e-70	no change
1900	1900	EXOSC6	1.61693094887094e-74	0.143859466458514	0.205	0.13	3.53202396471368e-70	no change
1901	1901	TOP1MT	1.76539838548868e-74	0.19823431686521	0.2	0.125	3.85633623326147e-70	no change
1902	1902	LETM1	1.8193679595787e-74	0.135511446546885	0.211	0.135	3.97422737090371e-70	no change
1903	1903	NFXL1	1.94857351603089e-74	0.511707358806326	0.09	0.044	4.25646398841788e-70	no change
1904	1904	DYNC2LI1	2.09943430510097e-74	0.182880932379136	0.19	0.118	4.58600429606256e-70	no change
1905	1905	LLPH	2.53316886210528e-74	0.17085165108538	0.389	0.279	5.53345406238277e-70	no change
1906	1906	ANXA9	2.69518597855431e-74	-1.320309576	0.059	0.106	5.88736425155404e-70	down

1907	1907	TOMM20	3.24579520971609e-74	0.160391033869084	0.754	0.603	7.09011505610382e-70	no change
1908	1908	C2	3.2877888335152e-74	-1.207019275	0.062	0.111	7.18184592793061e-70	down
1909	1909	C1orf106	4.03947785028404e-74	0.144101251460902	0.345	0.243	8.82383541616045e-70	no change
1910	1910	GAL	4.0814215941245e-74	2.45233660801319	0.024	0.003	8.91545733020555e-70	up
1911	1911	PGGT1B	4.53524334713132e-74	0.184623118742048	0.154	0.091	9.90678556747366e-70	no change
1912	1912	NT5DC2	4.6666538275148e-74	0.245062067409874	0.145	0.085	1.01938386208233e-69	no change
1913	1913	CTGF	4.86550832212473e-74	-2.232147532	0.053	0.097	1.06282163788493e-69	down
1914	1914	SH3RF1	4.97198070815033e-74	0.244909757868216	0.166	0.1	1.08607946588836e-69	no change
1915	1915	FAM83H	5.8984921152579e-74	0.283385639800453	0.155	0.093	1.28846661765694e-69	no change
1916	1916	ARFGEF1	6.05339788209945e-74	0.217568036501502	0.211	0.136	1.3223042333658e-69	no change
1917	1917	CSNK2A1	6.20194897687174e-74	0.152202575839017	0.306	0.211	1.35475373450786e-69	no change
1918	1918	DDX41	6.66437785184742e-74	0.231137847690781	0.189	0.118	1.45576669795755e-69	no change
1919	1919	PLEKHJ1	6.73372534496098e-74	0.246780793660065	0.416	0.311	1.47091496435328e-69	no change

1920	1920	RP11-758M4.4	7.2270438960648e-74	0.801121326160462	0.048	0.016	1.57867546865639e-69	no change
1921	1921	DAPL1	7.71623009769905e-74	0.136707189742735	0.194	0.122	1.68553330254138e-69	no change
1922	1922	C21orf58	7.91368949352227e-74	1.0003358258516	0.048	0.016	1.72866633296501e-69	up
1923	1923	PITPNB	8.4352488170257e-74	0.107280498695218	0.282	0.191	1.84259575159109e-69	no change
1924	1924	CCDC115	9.91864144682566e-74	0.1033337621038	0.403	0.287	2.1666280376446e-69	no change
1925	1925	NAB1	1.1155699573777e-73	0.225592787065643	0.149	0.088	2.43685101489584e-69	no change
1926	1926	TMX2	1.12981819615628e-73	0.165169693453161	0.334	0.235	2.46797486768378e-69	no change
1927	1927	FSIP2-AS1	1.18787006661823e-73	0.880953976101088	0.048	0.016	2.59478337352087e-69	no change
1928	1928	KRR1	1.26473318856473e-73	0.157251578301692	0.319	0.222	2.76268317710079e-69	no change
1929	1929	EXOSC2	1.36224087541487e-73	0.591035694383214	0.066	0.028	2.97567896825624e-69	no change
1930	1930	RILPL2	1.40427044078508e-73	0.107027150178076	0.25	0.164	3.06748835085093e-69	no change
1931	1931	FAM168B	1.43074906142136e-73	0.401486161738001	0.121	0.067	3.12532824976882e-69	no change
1932	1932	VEZF1	1.44487976087154e-73	0.172900074104583	0.201	0.127	3.1561953496478e-69	no change

1933	1933	LSM5	1.54088834485383e-73	0.181024303678587	0.776	0.637	3.3659165004987e-69	no change
1934	1934	ZNF440	1.76552385014398e-73	0.244131477441044	0.192	0.121	3.85661029825451e-69	no change
1935	1935	C14orf166	1.76635034154319e-73	0.170414812470517	0.755	0.611	3.85841568606694e-69	no change
1936	1936	SDHD	1.83458401623611e-73	0.142118385276967	0.465	0.338	4.00746532506616e-69	no change
1937	1937	GTPBP4	1.91831178187536e-73	0.130691522266632	0.302	0.206	4.19036025632854e-69	no change
1938	1938	PYCR2	1.94854164764894e-73	0.191042552196472	0.296	0.204	4.25639437512434e-69	no change
1939	1939	WSB1	1.97448402322655e-73	-0.517989052	0.581	0.575	4.31306290033607e-69	no change
1940	1940	DDX56	2.01439535296039e-73	0.179555403902942	0.207	0.132	4.40024520900669e-69	no change
1941	1941	ADGRG1	2.18728843941481e-73	0.177028308974293	0.253	0.169	4.77791286705771e-69	no change
1942	1942	USP11	2.28711349845238e-73	0.140690786156538	0.206	0.13	4.99597072601938e-69	no change
1943	1943	UPF3B	2.43128293208938e-73	0.291719095904742	0.191	0.121	5.31089443685603e-69	no change
1944	1944	TRMT61B	2.47830483107651e-73	0.311260005444244	0.119	0.065	5.41360907300353e-69	no change
1945	1945	SELK	2.53658504262906e-73	-0.309933635	0.771	0.737	5.54091636711892e-69	no change

1946	1946	HELZ	2.63672733151324e-73	0.215784458567238	0.189	0.118	5.75966718295752e-69	no change
1947	1947	GTF2H3	2.8801120880677e-73	0.304476405065769	0.127	0.071	6.29131684517508e-69	no change
1948	1948	ATP6V1H	2.94765873978232e-73	0.18043808017541	0.311	0.216	6.43886575118051e-69	no change
1949	1949	ABCA4	2.95664412515051e-73	2.02627230517823	0.029	0.006	6.45849342697876e-69	up
1950	1950	DDAH1	2.96702496323711e-73	0.34426022321382	0.162	0.098	6.48116932969515e-69	no change
1951	1951	SNX7	3.19356614891854e-73	0.1351608880372	0.278	0.188	6.97602589569766e-69	no change
1952	1952	ZSWIM7	3.22049650145091e-73	0.165228004867168	0.299	0.206	7.03485255776936e-69	no change
1953	1953	ZNF24	3.47661178123981e-73	0.168728228387187	0.218	0.141	7.59431077494023e-69	no change
1954	1954	TSR1	3.64294898162367e-73	0.282717555923387	0.13	0.073	7.95765775545874e-69	no change
1955	1955	IGF2BP3	3.86095086653876e-73	1.28887170343599	0.034	0.009	8.43386107286728e-69	up
1956	1956	COPS3	3.86227471501102e-73	0.228065873734305	0.197	0.126	8.43675288747007e-69	no change
1957	1957	PNO1	4.19602604811676e-73	0.189340663398283	0.18	0.111	9.16579929950625e-69	no change
1958	1958	KMT2A	4.78010549811234e-73	0.181464726672775	0.224	0.146	1.04416624500766e-68	no change

1959	1959	NTPCR	5.03321442533154e-73	0.14086042542651	0.369	0.262	1.09945535906942e-68	no change
1960	1960	SCN11A	5.30759347885572e-73	0.242264260765318	0.288	0.2	1.15939071952124e-68	no change
1961	1961	SYNE2	5.4191540886647e-73	0.294873740771644	0.169	0.103	1.18376001912792e-68	no change
1962	1962	HMGN5	6.01406339894973e-73	0.174299359371601	0.182	0.113	1.31371200886658e-68	no change
1963	1963	SDHAF3	6.63537850696204e-73	0.163969047210134	0.328	0.231	1.44943208106079e-68	no change
1964	1964	FAM207A	7.2682949079338e-73	0.107810713411109	0.251	0.167	1.58768633968906e-68	no change
1965	1965	UBQLN2	7.88621148508074e-73	0.205300509334175	0.209	0.135	1.72266403680104e-68	no change
1966	1966	NSMCE4A	7.92916839034623e-73	0.201158576670041	0.228	0.149	1.73204754318723e-68	no change
1967	1967	FOXM1	7.94795239319511e-73	1.35965348427722	0.039	0.011	1.73615072076954e-68	up
1968	1968	CENPA	8.67290639801143e-73	1.17924597168621	0.046	0.016	1.89450967358162e-68	up
1969	1969	PKP1	9.44218517111878e-73	0.249301573941844	0.077	0.035	2.06255092877919e-68	no change
1970	1970	MAP4K4	9.60443490839355e-73	0.180336399792052	0.166	0.101	2.09799276138949e-68	no change
1971	1971	MIEF1	1.15607211995909e-72	0.437788437955864	0.103	0.054	2.52532393883864e-68	no change

1972	1972	SNAI2	1.25613924558209e-72	0.180165391391603	0.339	0.242	2.74391056804951e-68	no change
1973	1973	ERI3	1.27267213840082e-72	0.153117247236265	0.33	0.232	2.78002501912276e-68	no change
1974	1974	TMEM216	1.4513777890151e-72	0.254007522267774	0.203	0.13	3.17038964232458e-68	no change
1975	1975	TAMM41	1.45795802611255e-72	0.581183541529965	0.077	0.035	3.18476351224026e-68	no change
1976	1976	CDT1	1.55296808989046e-72	0.831579716292016	0.054	0.02	3.39230349555673e-68	no change
1977	1977	TLE2	1.7152600666573e-72	0.214773866349063	0.174	0.107	3.74681408960622e-68	no change
1978	1978	FKBP7	2.02722678960344e-72	0.102311250223265	0.119	0.064	4.42827419920975e-68	no change
1979	1979	RAB3IP	2.16159268809432e-72	0.276790210647161	0.244	0.166	4.72178306787324e-68	no change
1980	1980	POLR3D	2.16569920387671e-72	0.272958809875846	0.125	0.07	4.73075334094828e-68	no change
1981	1981	PURA	2.1697633812129e-72	0.136152008204829	0.261	0.175	4.73963112992145e-68	no change
1982	1982	CFH	2.18597405886965e-72	-0.837647798	0.33	0.217	4.77504173419486e-68	no change
1983	1983	DYRK2	2.40803390305397e-72	0.29147053512286	0.144	0.085	5.2601092578311e-68	no change
1984	1984	SURF4	2.43984984297409e-72	0.1285031551802	0.272	0.183	5.3296079969926e-68	no change

1985	1985	SLU7	2.49725144261979e-72	0.186532808968134	0.232	0.152	5.45499605125867e-68	no change
1986	1986	DAXX	2.66153545320497e-72	0.226574283528107	0.197	0.125	5.81385804398094e-68	no change
1987	1987	CSRP1	2.71866643018219e-72	0.189483521936107	0.255	0.171	5.93865495008997e-68	no change
1988	1988	RFC5	2.85760219527288e-72	0.641947601188372	0.071	0.032	6.24214623535408e-68	no change
1989	1989	POU5F1	2.87217448970166e-72	0.417485289064325	0.107	0.057	6.27397795530431e-68	no change
1990	1990	CST1	2.88700188888942e-72	-1.762817054	0.08	0.131	6.30636692609005e-68	down
1991	1991	TCEB2	2.98705260137719e-72	-0.160864666	0.981	0.952	6.52491770244834e-68	no change
1992	1992	ITM2A	4.11970681395011e-72	-2.054875658	0.057	0.101	8.99908756439262e-68	down
1993	1993	CAND1	4.16498208380163e-72	0.178488485696961	0.333	0.235	9.09798686385628e-68	no change
1994	1994	ARV1	4.30932241241156e-72	0.216353050177652	0.179	0.111	9.41328387767182e-68	no change
1995	1995	AEBP1	4.43664069150622e-72	-1.66403991	0.038	0.077	9.69139792652618e-68	down
1996	1996	LAS1L	5.03902967120075e-72	0.343337007954812	0.124	0.07	1.10072564137709e-67	no change
1997	1997	COPG1	5.17266910733325e-72	0.15188406688784	0.199	0.126	1.12991783980587e-67	no change

1998	1998	CMBL	5.69116773309622e-72	0.324752892324051	0.145	0.085	1.24317867961754e-67	no change
1999	1999	PLEKHB2	5.75207757114135e-72	0.158625196047111	0.367	0.261	1.25648382464012e-67	no change
2000	2000	PHB	6.32210333537008e-72	0.206443391214453	0.671	0.532	1.38100025257824e-67	no change
2001	2001	THUMPD1	6.7565882132221e-72	0.181027931356165	0.232	0.153	1.47590912929624e-67	no change
2002	2002	PKMYT1	6.96235690157638e-72	1.20031387445228	0.037	0.011	1.52085724158035e-67	up
2003	2003	PRMT6	7.05126309418918e-72	0.355793315922169	0.098	0.051	1.54027791029468e-67	no change
2004	2004	ASPM	7.1077172701987e-72	1.41070879254536	0.036	0.01	1.5526097605022e-67	up
2005	2005	EMC10	7.22365526863526e-72	0.102848575209881	0.399	0.284	1.57793525688069e-67	no change
2006	2006	DTNBP1	7.42907269703844e-72	0.307157279713916	0.159	0.096	1.62280663994108e-67	no change
2007	2007	UGT2B7	8.61985066017535e-72	0.479724592186467	0.114	0.062	1.8829201782087e-67	no change
2008	2008	FAF2	8.71820897763616e-72	0.258399384483123	0.15	0.089	1.90440556907484e-67	no change
2009	2009	THY1	9.89608917109426e-72	-1.653523845	0.025	0.06	2.16170171853383e-67	down
2010	2010	HSP90B1	1.04803954930652e-71	0.162821952996132	0.762	0.617	2.28933759150517e-67	no change
2011	2011	SYNC	1.07654201822547e-7	0.16000556319845	0.187	0.116	2.35159838461172e-6	no

			1				7	change
2012	2012	ZNF512	1.53269753689524e-71	0.528077532006885	0.076	0.035	3.34802449959397e-67	no change
2013	2013	OCIAD2	1.65516389379059e-71	0.159978800908034	0.824	0.68	3.61554000959616e-67	no change
2014	2014	GRK6	1.70820792264137e-71	0.31509019480274	0.12	0.066	3.73140938621782e-67	no change
2015	2015	TNKS2	1.8551086944418e-71	0.177733727056647	0.229	0.151	4.05229943213867e-67	no change
2016	2016	TBC1D5	1.9073219189069e-71	0.325014739537147	0.123	0.069	4.16635399966023e-67	no change
2017	2017	PLEKHF1	2.19937564411009e-71	0.246501075487894	0.256	0.175	4.80431615699409e-67	no change
2018	2018	CENPN	2.29974204117956e-71	0.474632230488484	0.111	0.061	5.02355651475263e-67	no change
2019	2019	DYNC1I2	2.50325272486945e-71	0.13126763118292	0.589	0.445	5.46810525220482e-67	no change
2020	2020	MSL3	2.60396274150668e-71	0.29976512544405	0.15	0.09	5.68809621254719e-67	no change
2021	2021	UBN1	2.97130580264482e-71	0.289985994334093	0.138	0.08	6.49052039529733e-67	no change
2022	2022	RPP40	3.13317352272378e-71	0.347143023078551	0.113	0.061	6.84410424303782e-67	no change
2023	2023	EPHB6	3.47161061037852e-71	0.161324092981499	0.268	0.181	7.58338621731084e-67	no change
2024	2024	R3HDM2	3.55338371261241e-7	0.14211599797974	0.268	0.181	7.76201138183054e-6	no

			1	1			7	change
2025	2025	MED17	3.76829551360886e-71	0.225386924950703	0.166	0.101	8.2314647199272e-67	no change
2026	2026	ARMC1	3.90284388990051e-71	0.262062369378087	0.192	0.123	8.52537219309867e-67	no change
2027	2027	G3BP2	4.25485731402002e-71	0.117111285231676	0.401	0.288	9.29431031674533e-67	no change
2028	2028	OPA1	4.5410320197976e-71	0.286036785351319	0.148	0.088	9.91943034404588e-67	no change
2029	2029	CAP1	4.92609557510275e-71	0.147203162112455	0.656	0.506	1.07605631742545e-66	no change
2030	2030	BRAT1	5.14159007936072e-71	0.320079885931121	0.122	0.068	1.12312893693556e-66	no change
2031	2031	AGRN	5.34580405378177e-71	0.191134771563851	0.279	0.192	1.16773743750809e-66	no change
2032	2032	GFM1	5.41215640051428e-71	0.220184107160075	0.175	0.109	1.18223144412834e-66	no change
2033	2033	CTSL	5.80922311522286e-71	0.2316973215744	0.284	0.2	1.26896669728928e-66	no change
2034	2034	BBX	6.50540538650755e-71	0.126894934494603	0.448	0.328	1.42104075262871e-66	no change
2035	2035	DDX3X	6.5180440843458e-71	0.149108187041715	0.639	0.496	1.4238015497845e-66	no change
2036	2036	LSM14A	6.6933803469021e-71	0.11104677421525	0.41	0.294	1.46210200297729e-66	no change
2037	2037	CTB-92J24.3	7.29771422411447e-7	-2.093343587	0.017	0.047	1.59411269511556e-6	down

			1				6	
2038	2038	C4orf3	7.3130129672375e-71	-0.4722926	0.821	0.749	1.59745455256336e-66	no change
2039	2039	ALKBH7	7.61737972343789e-71	0.142013695219044	0.715	0.569	1.66394042678777e-66	no change
2040	2040	NCBP3	7.79128595165316e-71	0.270599560719827	0.158	0.096	1.70192850327912e-66	no change
2041	2041	EPM2AIP1	7.98637604058224e-71	0.258711123468986	0.162	0.099	1.74454398230478e-66	no change
2042	2042	COQ9	8.68602712823174e-71	0.281068723738411	0.23	0.154	1.89737576589094e-66	no change
2043	2043	UBE2J2	1.17341457700919e-70	0.199411002004129	0.321	0.226	2.56320680201888e-66	no change
2044	2044	GOLGA4	1.25729334865728e-70	0.143857489829459	0.404	0.293	2.74643159080697e-66	no change
2045	2045	DPH7	1.33416371182461e-70	0.390919381362826	0.107	0.057	2.91434721210968e-66	no change
2046	2046	IRAK1	1.42669468004479e-70	0.326001674058706	0.146	0.087	3.11647185908983e-66	no change
2047	2047	FBXW11	1.47609878440043e-70	0.188511506328231	0.174	0.108	3.22439018464429e-66	no change
2048	2048	CTR9	1.56702133359439e-70	0.186277155504718	0.164	0.1	3.42300140110359e-66	no change
2049	2049	TMSB10	1.65005100959846e-70	0.252184925903079	0.999	0.997	3.60437142536688e-66	no change
2050	2050	NDUFB11	1.80248289347887e-7	0.15793798633472	0.893	0.782	3.93734363251525e-6	no

			0	4			6	change
2051	2051	AK2	1.84397499367304e-70	0.146281914534546	0.528	0.396	4.02797897617939e-66	no change
2052	2052	NDUFS1	1.87844683261973e-70	0.187713561190489	0.166	0.102	4.10327926117454e-66	no change
2053	2053	TIMM44	1.924216624212e-70	0.172075370088311	0.263	0.179	4.20325879392869e-66	no change
2054	2054	PSMD5-AS1	1.99134649707568e-70	0.359261907518962	0.108	0.058	4.34989728821211e-66	no change
2055	2055	C5orf38	2.14543856431119e-70	0.735674907766778	0.049	0.018	4.68649599988136e-66	no change
2056	2056	TAF9B	2.16561014310497e-70	0.311103703921215	0.132	0.076	4.7305587965985e-66	no change
2057	2057	FBXO3	2.22353917894615e-70	0.156604290438438	0.192	0.121	4.85709898248997e-66	no change
2058	2058	TLE1	2.26855965548302e-70	0.178353208827313	0.195	0.123	4.95544171143712e-66	no change
2059	2059	CEP290	2.3307039990499e-70	0.292306283714155	0.141	0.083	5.09118981552461e-66	no change
2060	2060	WRB	2.43856991490922e-70	0.16563185444008	0.244	0.163	5.32681212212769e-66	no change
2061	2061	ACTR2	2.45923290804812e-70	0.122069138208129	0.549	0.409	5.37194836434032e-66	no change
2062	2062	MSH2	2.54138360155895e-70	0.466239008567596	0.085	0.041	5.55139833924536e-66	no change
2063	2063	PCID2	2.56243954650477e-7	0.19651021175710	0.167	0.102	5.59739294538501e-6	no

			0	6			6	change
2064	2064	DARS	2.76244628361981e-70	0.119128376737635	0.481	0.354	6.03428766193911e-66	no change
2065	2065	FBXO7	3.02624222834353e-70	0.134463262144195	0.355	0.252	6.61052352359362e-66	no change
2066	2066	SNX14	4.23118355983925e-70	0.171185147829052	0.242	0.161	9.24259736811285e-66	no change
2067	2067	SEPN1	5.94556259547419e-70	0.409727394947071	0.123	0.07	1.29874869335538e-65	no change
2068	2068	TXLNG	6.02493020181026e-70	0.171367892865649	0.132	0.076	1.31608575328343e-65	no change
2069	2069	MRPS23	6.31268308416893e-70	0.16276091081477	0.382	0.274	1.37894249290586e-65	no change
2070	2070	CCDC80	7.02584039097458e-70	-2.017404455	0.065	0.111	1.53472457500449e-65	down
2071	2071	INIP	7.36693706102751e-70	0.243862917975707	0.137	0.08	1.60923373161085e-65	no change
2072	2072	TOPORS	7.80094592811656e-70	0.212964289857219	0.139	0.081	1.70403862853778e-65	no change
2073	2073	ADM	8.06695183430323e-70	-0.817280336	0.383	0.421	1.7621449586852e-65	no change
2074	2074	PRMT5	9.14227837983449e-70	0.352839335075486	0.116	0.065	1.99703928929105e-65	no change
2075	2075	TSEN2	9.22630390533998e-70	0.490999867499299	0.092	0.047	2.01539382508246e-65	no change
2076	2076	STIP1	9.44453482795124e-7	0.10165298151269	0.339	0.238	2.06306418781767e-6	no

			0	7			5	change
2077	2077	DDX27	9.46193820280114e-70	0.101596927110168	0.325	0.226	2.06686578101988e-65	no change
2078	2078	HIST1H3G	9.59231837148292e-70	2.06810124180075	0.03	0.007	2.09534602506673e-65	up
2079	2079	METAP1D	9.91590156980612e-70	0.500134083656916	0.071	0.032	2.16602953890845e-65	no change
2080	2080	KIF2A	1.00217899030167e-69	0.28416165911265	0.132	0.076	2.18915978641498e-65	no change
2081	2081	PRSS27	1.12732413126251e-69	-1.719740825	0.041	0.08	2.46252683232983e-65	down
2082	2082	PTK2	1.2446342932542e-69	0.128161132799379	0.296	0.203	2.71877915018448e-65	no change
2083	2083	RAD21	1.35592665828115e-69	0.152492740436975	0.304	0.212	2.96188619234934e-65	no change
2084	2084	RANBP17	1.36123895718295e-69	0.775910868533543	0.056	0.023	2.97349037807044e-65	no change
2085	2085	MAPKAP1	1.41644007203828e-69	0.27080987638384	0.151	0.091	3.09407169336042e-65	no change
2086	2086	PSMA1	1.42657695986677e-69	0.124855594071995	0.731	0.576	3.11621471113297e-65	no change
2087	2087	RPP21	1.57035237414514e-69	0.206688571162097	0.179	0.112	3.43027772608264e-65	no change
2088	2088	ZNF451	1.58115330530604e-69	0.180833905742908	0.165	0.102	3.45387128011052e-65	no change
2089	2089	ARPP19	1.63312492168646e-6	0.15906503806984	0.52	0.391	3.5673980789319e-65	no

			9	7				change
2090	2090	RP11-382A20.3	1.6812212266932e-69	0.28386338407803	0.186	0.119	3.67245964758863e-65	no change
2091	2091	ZNF827	1.74095106479682e-69	0.296924840469672	0.125	0.071	3.80293350594217e-65	no change
2092	2092	ARMCX2	2.07412948826683e-69	0.348944257416638	0.113	0.063	4.53072845417007e-65	no change
2093	2093	PSMB5	2.07763091878892e-69	0.149620518424201	0.714	0.57	4.53837697900252e-65	no change
2094	2094	EP300	2.07980761629725e-69	0.177182357078578	0.173	0.107	4.54313175703971e-65	no change
2095	2095	RBM27	2.14536290021311e-69	0.336608799024005	0.097	0.051	4.68633071922551e-65	no change
2096	2096	USP14	2.22873296272223e-69	0.205058112344781	0.255	0.174	4.86844428377045e-65	no change
2097	2097	EIF2AK2	2.27782431059828e-69	0.181592471265198	0.301	0.211	4.97567942407089e-65	no change
2098	2098	SFT2D2	2.42836101195136e-69	0.172596539245486	0.156	0.094	5.30451179450656e-65	no change
2099	2099	TOMM70A	2.47494925714642e-69	0.265013161809878	0.167	0.104	5.40627915731065e-65	no change
2100	2100	TRIM33	2.49039073665753e-69	0.165295504140613	0.24	0.161	5.4400095251547e-65	no change
2101	2101	PI4K2B	2.51793602862588e-69	0.380927400505908	0.112	0.061	5.50017946093037e-65	no change
2102	2102	CXorf57	2.54348121069959e-6	0.65394014254348	0.062	0.026	5.55598035665219e-6	no

			9	9			5	change
2103	2103	TRMT2A	2.58167804248123e-69	0.329763809165692	0.136	0.08	5.63941751599599e-65	no change
2104	2104	SEH1L	2.60376658333624e-69	0.304979553750504	0.163	0.102	5.68766772463968e-65	no change
2105	2105	TMEM223	3.06213284798498e-69	0.176274998190918	0.258	0.175	6.6889229931384e-65	no change
2106	2106	TCEA1	3.17446131440898e-69	0.153411156869862	0.648	0.504	6.93429329519498e-65	no change
2107	2107	LL22NC03-2H8.5	3.45975313046084e-69	0.889089741745871	0.047	0.017	7.55748473817866e-65	no change
2108	2108	TAPBPL	3.95417573996477e-69	0.114144616789805	0.145	0.085	8.63750148637904e-65	no change
2109	2109	MAEA	3.96398760573289e-69	0.300570478896152	0.178	0.113	8.65893452596292e-65	no change
2110	2110	NUTF2	4.43984407171563e-69	0.132790049012089	0.579	0.438	9.69839539025563e-65	no change
2111	2111	PRR4	4.46234549800699e-69	0.495173517838774	0.082	0.04	9.74754750584647e-65	no change
2112	2112	KLHL13	4.72884806979855e-69	0.967089841467445	0.043	0.015	1.0329695723668e-64	no change
2113	2113	AURKA	4.8094013447091e-69	1.05584520257351	0.053	0.02	1.05056562973826e-64	up
2114	2114	TMED5	5.31693588803418e-69	0.122762519389798	0.332	0.234	1.16143147538219e-64	no change
2115	2115	CLINT1	6.06275165819716e-6	0.10823447862019	0.358	0.254	1.32434747221659e-6	no

			9	4			4	change
2116	2116	PEX26	6.56519577348002e-69	0.340027798934287	0.116	0.064	1.43410136475898e-64	no change
2117	2117	DNAAF2	7.22059820679297e-69	0.266508403008124	0.167	0.104	1.57726747229186e-64	no change
2118	2118	CDYL	7.4866849981461e-69	0.195811248609097	0.226	0.151	1.63539147099503e-64	no change
2119	2119	YIPF4	7.80448568893468e-69	0.126756787622843	0.37	0.266	1.70481185389089e-64	no change
2120	2120	ZBTB38	9.25678963530987e-69	0.150984311560026	0.212	0.138	2.02205312793709e-64	no change
2121	2121	GPSM2	1.03703517094959e-68	0.482862306692402	0.087	0.044	2.26529962742229e-64	no change
2122	2122	PRMT9	1.17624353688317e-68	0.179545572075944	0.165	0.102	2.5693863819676e-64	no change
2123	2123	RBM28	1.17988968979592e-68	0.362907125722253	0.118	0.066	2.57735103839021e-64	no change
2124	2124	VRK1	1.24004868230843e-68	0.396790738678167	0.098	0.051	2.70876234163454e-64	no change
2125	2125	CC2D1A	1.55221457969574e-68	0.241239347320788	0.186	0.118	3.39065752788737e-64	no change
2126	2126	DCTN5	1.55887134533872e-68	0.240844551684207	0.158	0.097	3.4051985667579e-64	no change
2127	2127	ATP5EP2	1.60536549748795e-68	0.13363694904784	0.303	0.211	3.50676039271268e-64	no change
2128	2128	NDC1	1.63974766135764e-6	0.51395670786581	0.083	0.041	3.58186479146963e-6	no

			8	4			4	change
2129	2129	SP3	1.81730110807074e-68	0.102612649747173	0.178	0.111	3.96971254046972e-64	no change
2130	2130	MARVELD1	1.8494705970171e-68	0.58955500836434	0.073	0.034	4.03998357212414e-64	no change
2131	2131	AGL	1.87949904954914e-68	0.234403671664048	0.153	0.093	4.10557772383515e-64	no change
2132	2132	GALNT3	1.90709192542974e-68	0.202179342480275	0.298	0.21	4.16585160190873e-64	no change
2133	2133	LIAS	1.96102494656344e-68	0.350119466705018	0.118	0.066	4.28366289327317e-64	no change
2134	2134	GPR157	2.01937911995496e-68	0.241312155933751	0.137	0.081	4.41113174962961e-64	no change
2135	2135	SF3A1	2.0415588112879e-68	0.240275325330382	0.181	0.114	4.45958106737729e-64	no change
2136	2136	MCM6	2.09990961954842e-68	0.655214891437819	0.059	0.024	4.58704257294156e-64	no change
2137	2137	THOP1	2.16158610173803e-68	0.477881154451462	0.091	0.047	4.72176868063656e-64	no change
2138	2138	ARHGAP5	2.28100843887898e-68	0.116125416868656	0.315	0.22	4.98263483388724e-64	no change
2139	2139	ELK4	2.33209379726404e-68	0.119272858505425	0.229	0.152	5.09422569074356e-64	no change
2140	2140	ANKRD54	2.38921424619141e-68	0.331770392372951	0.128	0.074	5.21899959938051e-64	no change
2141	2141	TRIAP1	2.39246583333245e-6	0.16651699513885	0.271	0.187	5.2261023663314e-64	no

			8	4				change
2142	2142	TRAPPC2	2.43486783256863e-68	0.287137437744633	0.125	0.071	5.3187252934629e-64	no change
2143	2143	PRKCA	2.53597971431628e-68	0.160433675518298	0.276	0.19	5.53959408795249e-64	no change
2144	2144	RBM47	2.61214862237871e-68	0.214273224917393	0.387	0.285	5.70597745072405e-64	no change
2145	2145	MYCBP2	2.62280791112399e-68	0.120711427860052	0.258	0.175	5.72926160105923e-64	no change
2146	2146	RECQL	2.64982083902132e-68	0.163843295812672	0.14	0.083	5.78826864075816e-64	no change
2147	2147	DBT	2.70475668593772e-68	0.273296784152587	0.121	0.068	5.90827050476235e-64	no change
2148	2148	FIP1L1	2.79042485566999e-68	0.142583085627137	0.217	0.142	6.09540405472552e-64	no change
2149	2149	ARHGAP23	2.97478646873862e-68	0.246287354959211	0.189	0.121	6.49812356231264e-64	no change
2150	2150	ERP29	3.03156638087602e-68	0.138679712363988	0.807	0.667	6.62215360238558e-64	no change
2151	2151	CRH	3.20113734334706e-68	0.777431356450724	0.16	0.105	6.99256441280733e-64	no change
2152	2152	WDR48	3.28187670288121e-68	0.375682019627731	0.122	0.07	7.16893146977372e-64	no change
2153	2153	PRPSAP2	3.30047226577815e-68	0.421746041618633	0.118	0.067	7.20955161736578e-64	no change
2154	2154	DDX50	3.4094969337247e-68	0.18136733455238	0.179	0.113	7.44770510202823e-6	no

				3			4	change
2155	2155	TTF2	3.63583569792037e-68	0.511685818853757	0.082	0.04	7.94211949853726e-64	no change
2156	2156	EPB41	3.74041764935299e-68	0.393146207572325	0.139	0.083	8.17056831324667e-64	no change
2157	2157	GOLM1	4.13908337775724e-68	0.265615056732683	0.122	0.069	9.04141373037293e-64	no change
2158	2158	SLC12A5	5.21676481595502e-68	0.863691959500296	0.046	0.016	1.13955010639721e-63	no change
2159	2159	EVL	5.40794849451359e-68	0.272373157617988	0.085	0.042	1.18131226914155e-63	no change
2160	2160	C11orf54	5.51149430675392e-68	0.311658367802074	0.146	0.088	1.20393081636733e-63	no change
2161	2161	WARS2	5.80036569556936e-68	0.2539486865833	0.129	0.074	1.26703188254017e-63	no change
2162	2162	KIF4A	5.8714983878397e-68	1.83084119053459	0.025	0.005	1.2825701078397e-63	up
2163	2163	IGKC	5.92907841075072e-68	-2.735001029	0.29	0.344	1.29514788804439e-63	down
2164	2164	IKBIP	6.32023844930178e-68	0.277148189444347	0.106	0.057	1.38059288686548e-63	no change
2165	2165	PLGRKT	6.85757787136904e-68	0.207157904585019	0.182	0.115	1.49796931022185e-63	no change
2166	2166	NR2F2	7.1595879595753e-68	0.151623272875036	0.257	0.175	1.56394039388963e-63	no change
2167	2167	ILVBL	7.23257275672188e-68	0.166552925688185	0.255	0.173	1.57988319297833e-63	no change

2168	2168	PDZK1IP1	7.41196212229651e-68	-1.42929208	0.096	0.146	1.61906900599445e-63	down
2169	2169	TFCP2	8.27324059200425e-68	0.366877293631735	0.108	0.059	1.80720667491741e-63	no change
2170	2170	PPFIBP2	8.49603526395224e-68	0.186075934461883	0.207	0.135	1.85587394305773e-63	no change
2171	2171	CSNK1E	9.45166767523526e-68	0.116671463103339	0.358	0.255	2.06462228697839e-63	no change
2172	2172	CHTF8	9.59197088933641e-68	0.200703545846333	0.207	0.135	2.09527012106665e-63	no change
2173	2173	ATXN7L3B	9.5972211865429e-68	0.232765552502299	0.199	0.129	2.09641699598843e-63	no change
2174	2174	DOCK7	1.00731745518981e-67	0.431455328073302	0.101	0.054	2.20038424911661e-63	no change
2175	2175	CDK6	1.12581534283167e-67	0.570458258529841	0.061	0.026	2.45923103488151e-63	no change
2176	2176	S100A8	1.14595276375919e-67	-0.962873884	0.165	0.226	2.50321921715558e-63	no change
2177	2177	SNRNP27	1.15933325198338e-67	0.146834039483694	0.35	0.252	2.53244755563249e-63	no change
2178	2178	RMDN1	1.1910343316107e-67	0.191825759327553	0.156	0.095	2.6016953939704e-63	no change
2179	2179	BMPR2	1.19512662488505e-67	0.120136083005197	0.222	0.147	2.61063459939891e-63	no change
2180	2180	TIMMDC1	1.21250360789445e-67	0.143697521737688	0.399	0.289	2.64859288108464e-63	no change

2181	2181	FAM173B	1.38547612794967e-67	0.508760400801483	0.098	0.052	3.02643405389325e-63	no change
2182	2182	SCNN1B	1.39905297092891e-67	0.683427378142496	0.172	0.114	3.0560913096971e-63	no change
2183	2183	COL14A1	1.58843706310537e-67	-1.436736502	0.032	0.068	3.46978192064737e-63	down
2184	2184	SLC35D1	1.61422758697402e-67	0.358639648291596	0.107	0.059	3.52611874098606e-63	no change
2185	2185	C9orf114	1.62418851268004e-67	0.437387953727931	0.089	0.045	3.54787738709829e-63	no change
2186	2186	SAAL1	1.64899977582417e-67	0.423331186810727	0.086	0.043	3.60207511031032e-63	no change
2187	2187	ABO	1.72119598861101e-67	0.450690237755633	0.098	0.052	3.75978051752188e-63	no change
2188	2188	RNASEH2B	1.81479186187113e-67	0.137362659820515	0.291	0.202	3.9642313430713e-63	no change
2189	2189	FAM84B	1.82194332353502e-67	0.313009511441271	0.165	0.103	3.9798529959299e-63	no change
2190	2190	BCOR	1.87591828995125e-67	0.396704263836736	0.094	0.049	4.09775591256951e-63	no change
2191	2191	PPIL1	1.99268952635193e-67	0.309072083678633	0.131	0.077	4.35283100136316e-63	no change
2192	2192	TRIP12	2.02655128107198e-67	0.142723624817948	0.215	0.141	4.42679861837363e-63	no change
2193	2193	DHX33	2.08524050770715e-67	0.481288313617605	0.077	0.037	4.55499936503549e-63	no change

2194	2194	DYNC1LI1	2.15901948609378e-67	0.127032130204634	0.159	0.097	4.71616216542326e-63	no change
2195	2195	SLC25A36	2.29832836424739e-67	0.149052292609869	0.221	0.146	5.02046847886199e-63	no change
2196	2196	MCCC1	2.36459229018796e-67	0.223045911015029	0.25	0.171	5.16521539868657e-63	no change
2197	2197	GRHPR	2.42453640589648e-67	0.126639468878229	0.298	0.206	5.29615732504027e-63	no change
2198	2198	HDGF	2.46692854912779e-67	0.211538019828793	0.422	0.315	5.38875872271474e-63	no change
2199	2199	CTSH	2.68077889501515e-67	0.153626148693371	0.847	0.736	5.85589341827109e-63	no change
2200	2200	KREMEN2	2.86607426540469e-67	0.966231831924883	0.043	0.015	6.26065262535e-63	no change
2201	2201	MEG3	2.86688466856627e-67	-1.246901943	0.039	0.078	6.26242287001616e-63	down
2202	2202	EMX2	3.3629154317992e-67	0.500119381440502	0.112	0.063	7.34595246922216e-63	no change
2203	2203	PDE4D	3.47877829778329e-67	0.172411835764382	0.134	0.078	7.59904331367782e-63	no change
2204	2204	AP1M2	3.55599515600253e-67	0.156429064649405	0.394	0.286	7.76771581877193e-63	no change
2205	2205	BLVRB	3.72536648910481e-67	-0.476244797	0.539	0.538	8.13769055880056e-63	no change
2206	2206	EIF4EBP2	3.89984725410069e-67	0.115500793896258	0.371	0.265	8.51882634185754e-63	no change

2207	2207	SLCO3A1	4.02228914100476e-67	0.155611398043642	0.123	0.07	8.7862883996108e-63	no change
2208	2208	MOGS	4.06082951477701e-67	0.348241911398466	0.13	0.076	8.87047599207891e-63	no change
2209	2209	TELO2	4.72270749192589e-67	0.399461458563138	0.092	0.048	1.03162822453629e-62	no change
2210	2210	UBAC1	4.75593000888926e-67	0.209419001473463	0.168	0.105	1.03888535114177e-62	no change
2211	2211	PDCD6IP	5.18782772461532e-67	0.122381062791785	0.294	0.205	1.13322908816497e-62	no change
2212	2212	TSPYL5	5.52668498736079e-67	0.515806577671565	0.075	0.036	1.20724906863909e-62	no change
2213	2213	THOC6	5.98144425241105e-67	0.145698849490991	0.262	0.179	1.30658668249667e-62	no change
2214	2214	CLSTN1	6.48243213139428e-67	0.180602278287923	0.188	0.12	1.41602247478177e-62	no change
2215	2215	PSMC3IP	7.09468165481185e-67	0.921048570053201	0.048	0.018	1.5497622606771e-62	no change
2216	2216	REST	7.55900791796631e-67	0.151490458469615	0.282	0.195	1.65118968960056e-62	no change
2217	2217	MRPL48	7.61491546990092e-67	0.167873529436336	0.279	0.194	1.66340213524516e-62	no change
2218	2218	MPDU1	7.74969516256916e-67	0.200812652924057	0.215	0.142	1.69284341131161e-62	no change
2219	2219	XPR1	7.94069368211178e-67	0.348209130823181	0.145	0.088	1.7345651279205e-62	no change

2220	2220	POLR3K	8.04848028210942e-67	0.201612757538018	0.294	0.207	1.75811003282398e-62	no change
2221	2221	ZNF358	8.37229595278743e-67	0.298066843731675	0.128	0.074	1.82884432792689e-62	no change
2222	2222	CASP1	9.05977971436887e-67	0.170395026529537	0.174	0.109	1.97901828080674e-62	no change
2223	2223	EYA3	9.71686137461754e-67	0.418981623074162	0.097	0.052	2.12255119867146e-62	no change
2224	2224	MRPS18C	1.02545844687865e-66	0.112839847067086	0.501	0.373	2.24001143136172e-62	no change
2225	2225	EXOSC8	1.14552250640231e-66	0.162521191601734	0.212	0.139	2.5022793629852e-62	no change
2226	2226	USP8	1.1736579156342e-66	0.114433701848205	0.311	0.218	2.56373835091135e-62	no change
2227	2227	NBR1	1.18604917646643e-66	0.164067828392562	0.234	0.158	2.59080582107327e-62	no change
2228	2228	FAM98A	1.19721093230545e-66	0.315821050430352	0.136	0.081	2.61518756052802e-62	no change
2229	2229	TAF3	1.22087604444847e-66	0.18719727266773	0.171	0.107	2.66688163149324e-62	no change
2230	2230	AGPS	1.25717485096929e-66	0.243288507944862	0.116	0.065	2.74617274445731e-62	no change
2231	2231	GCDH	1.30325096184486e-66	0.298236963508114	0.13	0.076	2.84682140105391e-62	no change
2232	2232	PREPL	1.3255755868474e-66	0.225347630311621	0.158	0.098	2.89558731190947e-62	no change

2233	2233	LPGAT1	1.37303491405954e-66	0.205074749785869	0.15	0.091	2.99925746627165e-62	no change
2234	2234	NQO2	1.42966374401451e-66	0.131941839721101	0.184	0.117	3.12295748242529e-62	no change
2235	2235	ZMYM2	1.52387292235542e-66	0.120624474767189	0.297	0.206	3.32874801159318e-62	no change
2236	2236	PLAU	1.59447002060855e-66	-0.99620794	0.191	0.247	3.48296031301731e-62	no change
2237	2237	BRI3BP	1.59690811968962e-66	0.34994176622803	0.114	0.064	3.48828609665001e-62	no change
2238	2238	FAM199X	1.91990433533993e-66	0.234234294349303	0.162	0.101	4.19383903011654e-62	no change
2239	2239	CDK12	2.01777101452919e-66	0.143668182191938	0.269	0.185	4.40761900413755e-62	no change
2240	2240	KIAA0141	2.09453023376204e-66	0.249426590452024	0.121	0.068	4.57529184262979e-62	no change
2241	2241	MRPS36	2.14574464729057e-66	0.107583312566823	0.603	0.456	4.68716460754153e-62	no change
2242	2242	RP11-706O15.1	2.35599401578917e-66	0.669421298304798	0.067	0.03	5.14643332808986e-62	no change
2243	2243	WRNIP1	2.43653358362887e-66	0.340079817691931	0.121	0.069	5.3223639600789e-62	no change
2244	2244	RBM4B	2.50510913106824e-66	0.310727303984564	0.129	0.075	5.47216038590547e-62	no change
2245	2245	ID1	2.5207758947076e-66	-0.519699137	0.944	0.91	5.50638286439927e-62	no change

2246	2246	PIK3CD-AS2	2.65724396753553e-66	0.975741090750351	0.038	0.012	5.8044837226846e-62	no change
2247	2247	LRRC58	3.0723345745692e-66	0.247749692079792	0.184	0.119	6.71120764468896e-62	no change
2248	2248	POLR3E	3.17318353433783e-66	0.255391233723267	0.129	0.075	6.93150211240755e-62	no change
2249	2249	HCCS	3.17612032259277e-66	0.155703874054854	0.257	0.175	6.93791723267165e-62	no change
2250	2250	MCMBP	3.28459067013774e-66	0.227963270686496	0.146	0.088	7.17485985984888e-62	no change
2251	2251	CDC37	3.57493488919728e-66	0.112355515370947	0.584	0.442	7.80908777196254e-62	no change
2252	2252	TSSC4	3.58478091464686e-66	0.128991624751246	0.275	0.19	7.83059542995461e-62	no change
2253	2253	RP11-322E11.2	3.61325048678958e-66	0.494436412879017	0.062	0.027	7.89278436334317e-62	no change
2254	2254	THUMPD2	3.79019233669504e-66	0.343544302955993	0.119	0.068	8.27929614027665e-62	no change
2255	2255	TPRXL	3.80884778069761e-66	-0.929655799	0.088	0.139	8.32004709215586e-62	no change
2256	2256	CTC-444N24.11	3.89129129005536e-66	0.147775942777799	0.129	0.075	8.50013669399692e-62	no change
2257	2257	HM13	4.10590936343405e-66	0.107518031141016	0.509	0.382	8.96894841348533e-62	no change
2258	2258	PSMC1	4.17008943985649e-66	0.138576467496264	0.433	0.317	9.10914337242251e-62	no change

2259	2259	NCLN	4.57527147408962e-66	0.31717387630305	0.142	0.086	9.99422300800136e-62	no change
2260	2260	PELP1	4.95300759080249e-66	0.406950636408339	0.088	0.045	1.0819349781349e-61	no change
2261	2261	SNHG18	5.42673817621266e-66	0.259970577992984	0.465	0.358	1.18541668721189e-61	no change
2262	2262	MCM3AP	5.70369289284813e-66	0.368409761806468	0.091	0.047	1.24591467551374e-61	no change
2263	2263	ADH1B	6.00000042314512e-66	-1.885061528	0.031	0.066	1.31064009243182e-61	down
2264	2264	EBP	6.14128207394732e-66	0.115401090785575	0.377	0.272	1.34150165623305e-61	no change
2265	2265	PLOD1	6.18531029608815e-66	0.340774992503579	0.123	0.071	1.3511191810775e-61	no change
2266	2266	SYAP1	6.21815386185615e-66	0.126957014743397	0.441	0.326	1.35829352958386e-61	no change
2267	2267	HACD2	6.38497127180811e-66	0.261856316503525	0.156	0.097	1.39473312461376e-61	no change
2268	2268	RCAN3	7.19112675981926e-66	0.203933197795815	0.205	0.135	1.57082972941492e-61	no change
2269	2269	ANK3	7.3244788063035e-66	0.506456724514179	0.092	0.048	1.59995915044894e-61	no change
2270	2270	SETX	7.56144086230591e-66	0.216748370118978	0.123	0.07	1.6517211419621e-61	no change
2271	2271	CNPY3	7.88978875216962e-66	0.17059733882619	0.344	0.248	1.72344545502393e-61	no change

2272	2272	TAF9	7.93304330834994e-66	0.101411560430975	0.445	0.324	1.73289398027596e-61	no change
2273	2273	CLIC3	7.96295727321296e-66	-1.216843949	0.152	0.206	1.73942838676064e-61	down
2274	2274	AURKAIP1	8.02187026583672e-66	0.224394689809056	0.834	0.707	1.75229734086937e-61	no change
2275	2275	KCTD1	8.30869556703385e-66	0.278518877998112	0.117	0.066	1.81495145966288e-61	no change
2276	2276	CNOT6L	8.35800055001599e-66	0.1536583374059	0.181	0.115	1.82572164014549e-61	no change
2277	2277	PSMA3	9.81961688373089e-66	0.103175795301842	0.593	0.45	2.14499711208218e-61	no change
2278	2278	RBM22	1.03344366615914e-65	0.120376425975725	0.192	0.122	2.25745434435803e-61	no change
2279	2279	TRAF2	1.22831070237095e-65	0.506413440552098	0.075	0.036	2.68312189825909e-61	no change
2280	2280	CTA-243E7.1	1.44118761242073e-65	0.470291619426674	0.073	0.034	3.14813022057185e-61	no change
2281	2281	ANKS1A	1.49037345505342e-65	0.35004828983653	0.142	0.086	3.25557177521868e-61	no change
2282	2282	RAF1	1.52886574669941e-65	0.160140953765628	0.222	0.148	3.33965433709019e-61	no change
2283	2283	PCBP1	1.58293229476242e-65	0.140319648986069	0.853	0.725	3.45775730467904e-61	no change
2284	2284	ZNF33B	1.61072797126715e-65	0.30793752469106	0.131	0.077	3.51847418043597e-61	no change

2285	2285	PEMT	1.66560225971059e-65	0.17638525380077	0.191	0.123	3.63834157611182e-61	no change
2286	2286	BCAS1	1.77558808729219e-65	0.156126622066235	0.418	0.307	3.87859461788106e-61	no change
2287	2287	IMPA2	1.78941862640251e-65	0.440780072126564	0.105	0.058	3.90880604751364e-61	no change
2288	2288	EML4	1.90950350849093e-65	0.118595318103649	0.288	0.2	4.17111946394758e-61	no change
2289	2289	UTP14A	2.0005961611215e-65	0.449165803247619	0.079	0.039	4.3701022543538e-61	no change
2290	2290	PSMD4	2.00621540151209e-65	0.126431466391878	0.594	0.452	4.38237692306302e-61	no change
2291	2291	CSTF2T	2.106680595412e-65	0.460942276093578	0.078	0.038	4.60183309261797e-61	no change
2292	2292	PCSK7	2.17321456482899e-65	0.164068506230501	0.242	0.165	4.74716989541244e-61	no change
2293	2293	CSDE1	2.18215575367474e-65	0.126535499938659	0.718	0.569	4.76670102832711e-61	no change
2294	2294	ZBED5	2.24109023469438e-65	0.202032831518646	0.131	0.077	4.89543750866641e-61	no change
2295	2295	GEMIN8	2.27685632538456e-65	0.372474950665514	0.127	0.074	4.97356495717004e-61	no change
2296	2296	SPATS2	2.28636073948328e-65	0.203488238466599	0.231	0.155	4.99432639932728e-61	no change
2297	2297	TOB1	2.30172594016531e-65	-0.376739074	0.76	0.708	5.0278901436971e-61	no change

2298	2298	NELFA	2.5067215538342e-65	0.249159982818002	0.166	0.104	5.47568256219542e-61	no change
2299	2299	CNOT10	2.73857420488816e-65	0.424633306193425	0.095	0.051	5.9821414931577e-61	no change
2300	2300	COPS5	2.79435226069067e-65	0.165577458215388	0.329	0.235	6.1039830782527e-61	no change
2301	2301	DYNLL2	2.84688837508549e-65	0.18790088067581	0.245	0.168	6.21874296653675e-61	no change
2302	2302	ANO10	2.95955107640908e-65	0.361156333785957	0.149	0.092	6.46484337130798e-61	no change
2303	2303	MSH6	2.96001843576731e-65	0.550601296997972	0.074	0.036	6.46586427109011e-61	no change
2304	2304	SUPT20H	3.05781802923675e-65	0.219315952671456	0.17	0.107	6.67949770306476e-61	no change
2305	2305	METTL3	3.06835672555265e-65	0.359456108773498	0.104	0.057	6.70251843129721e-61	no change
2306	2306	SPPL3	3.41213194781655e-65	0.194708280002473	0.226	0.153	7.45346102681047e-61	no change
2307	2307	TOMM5	3.41583011479019e-65	0.116883257970004	0.263	0.18	7.46153930274769e-61	no change
2308	2308	ARHGAP42	3.43454705852615e-65	0.669748706429657	0.058	0.025	7.50242459464451e-61	no change
2309	2309	BCAP31	3.56298409097805e-65	0.169309654289082	0.815	0.676	7.78298244833245e-61	no change
2310	2310	PPP4R3B	3.71008763921007e-65	0.170222214149677	0.221	0.148	8.10431543909047e-61	no change

2311	2311	TP53AIP1	3.71107958113544e-65	1.43354715531035	0.029	0.007	8.10648223703225e-61	up
2312	2312	CCP110	3.8882011508364e-65	0.392635601262335	0.079	0.039	8.49338659388703e-61	no change
2313	2313	DLX6-AS1	4.13059492134056e-65	0.846589540718171	0.056	0.023	9.02287154617632e-61	no change
2314	2314	CCDC137	4.40141082017706e-65	0.278273322212028	0.19	0.124	9.61444179559476e-61	no change
2315	2315	KAT2A	4.45638951879631e-65	0.243840641227816	0.158	0.098	9.73453726485866e-61	no change
2316	2316	UNC93B1	4.61950847524692e-65	0.216949220557586	0.24	0.165	1.00908543133294e-60	no change
2317	2317	POU2F1	4.76933412194707e-65	0.404776749892248	0.1	0.054	1.04181334559812e-60	no change
2318	2318	PTER	4.97117214405278e-65	0.172038407091249	0.165	0.103	1.08590284314689e-60	no change
2319	2319	ODF2	8.02007456725074e-65	0.450735065104293	0.078	0.038	1.75190508847025e-60	no change
2320	2320	AEBP2	8.1266113934949e-65	0.167292861545272	0.175	0.111	1.77517699279503e-60	no change
2321	2321	BRCC3	8.58059308707529e-65	0.415057011924915	0.087	0.044	1.87434475394073e-60	no change
2322	2322	EFNB2	9.02104740389979e-65	0.235039662277957	0.126	0.073	1.97055759490787e-60	no change
2323	2323	ESRRA	9.03839431950803e-65	0.186855295988261	0.209	0.138	1.97434685515333e-60	no change

2324	2324	RP3-510D11.2	9.3914818279936e-65	0.593560975799514	0.09	0.047	2.05147529050692e-60	no change
2325	2325	CASP2	1.0839343584693e-64	0.299965204705624	0.134	0.08	2.36774621264033e-60	no change
2326	2326	TWF1	1.12390028986847e-64	0.133282664568101	0.434	0.318	2.45504779318869e-60	no change
2327	2327	C7orf55	1.15692502159612e-64	0.130126433544537	0.425	0.313	2.52718701717456e-60	no change
2328	2328	ZMIZ1	1.25279968856339e-64	0.243342653955868	0.152	0.094	2.73661563969786e-60	no change
2329	2329	LINC00668	1.26846196995087e-64	0.87374572096117	0.046	0.017	2.77082832716068e-60	no change
2330	2330	TOPBP1	1.27617145548078e-64	0.391105869031366	0.079	0.039	2.78766892735222e-60	no change
2331	2331	CERS2	1.46498306010853e-64	0.156301286600365	0.249	0.17	3.20010899650107e-60	no change
2332	2332	C16orf74	1.54582836897822e-64	0.109641912465427	0.515	0.381	3.37670748919602e-60	no change
2333	2333	C1orf174	1.58539107343799e-64	0.227131609891423	0.175	0.112	3.46312826081794e-60	no change
2334	2334	HOXA3	1.67192223616221e-64	0.33807368671912	0.092	0.048	3.65214693267274e-60	no change
2335	2335	RNF4	1.82215424825948e-64	0.250384417743791	0.158	0.099	3.98031373989801e-60	no change
2336	2336	SLC30A9	1.9538588633386e-64	0.182399349783504	0.214	0.143	4.26800930107684e-60	no change

2337	2337	NT5C3B	2.15961272666707e-64	0.175392101714156	0.253	0.174	4.71745804013155e-60	no change
2338	2338	CDC45	2.22184117189883e-64	1.66905962132913	0.026	0.006	4.85338985589581e-60	up
2339	2339	AP2B1	2.26778964118452e-64	0.105764809139758	0.345	0.247	4.95375969220346e-60	no change
2340	2340	SHCBP1	2.34367841984117e-64	1.43617053194765	0.027	0.006	5.11953114030106e-60	up
2341	2341	STX16	2.63026705091065e-64	0.134204316079889	0.213	0.141	5.74555534600922e-60	no change
2342	2342	ACYP1	2.81608106174486e-64	0.272597089103495	0.218	0.147	6.15144747127548e-60	no change
2343	2343	UBXN7	2.86113043945279e-64	0.331836399152934	0.119	0.069	6.24985333194068e-60	no change
2344	2344	SEC61A1	3.07963662561708e-64	0.134272556121359	0.3	0.211	6.72715824499794e-60	no change
2345	2345	WDR45	3.10990212194326e-64	0.100583269667084	0.367	0.264	6.79327019517285e-60	no change
2346	2346	AFF1	3.11656973852949e-64	0.224233735480954	0.139	0.083	6.80783493684383e-60	no change
2347	2347	PSAT1	3.12957608369525e-64	0.426125591254241	0.1	0.054	6.83624599722389e-60	no change
2348	2348	ACOT11	3.24020521808936e-64	0.240318982500551	0.155	0.096	7.0779042783944e-60	no change
2349	2349	MIB1	3.43261511483397e-64	0.266803703219376	0.127	0.075	7.49820445684332e-60	no change

2350	2350	LGR4	3.47105501112527e-64	0.250194454933908	0.101	0.055	7.58217256630204e-60	no change
2351	2351	RNF34	3.52888904825256e-64	0.237894499546818	0.178	0.114	7.70850523700288e-60	no change
2352	2352	CIR1	3.88171341135455e-64	0.117530114870887	0.339	0.243	8.47921477576288e-60	no change
2353	2353	TMC4	4.37012996987343e-64	0.131941016076875	0.326	0.231	9.54611190619151e-60	no change
2354	2354	HSPBP1	4.49107734818914e-64	0.122309712852229	0.21	0.138	9.81030935938436e-60	no change
2355	2355	C12orf73	4.69725310254491e-64	0.391155128925909	0.125	0.073	1.02606796771991e-59	no change
2356	2356	PTPMT1	4.94522427455015e-64	0.161379533831776	0.271	0.188	1.08023479053274e-59	no change
2357	2357	MARS	5.28670708166867e-64	0.33149608164109	0.124	0.073	1.1548282949197e-59	no change
2358	2358	PYCR1	5.9646559532756e-64	0.516160672169569	0.162	0.105	1.30291944643352e-59	no change
2359	2359	GPRC5A	6.01798103796315e-64	-0.396623097	0.5	0.537	1.31456777793267e-59	no change
2360	2360	F2RL1	6.08397064240756e-64	0.304590350186698	0.159	0.1	1.32898254712751e-59	no change
2361	2361	BSDC1	6.22903606805539e-64	0.181636743786149	0.246	0.168	1.36067063870602e-59	no change
2362	2362	RFC4	6.50752289696742e-64	0.613539792995283	0.072	0.034	1.42150330161356e-59	no change

2363	2363	NKX2-8	6.56821183551714e-64	1.80532131973224	0.025	0.005	1.43476019335036e-59	up
2364	2364	ALDH18A1	6.64182627894569e-64	0.430617672590322	0.107	0.06	1.4508405323729e-59	no change
2365	2365	IRS2	6.84427800041777e-64	0.127180835178462	0.142	0.085	1.49506408641126e-59	no change
2366	2366	MRPS22	7.5327073826047e-64	0.162346998056668	0.208	0.137	1.64544460065617e-59	no change
2367	2367	DAB2IP	7.98673028755615e-64	0.55762396283573	0.064	0.029	1.74462136401377e-59	no change
2368	2368	TRIM29	8.14700948947626e-64	0.11053287651325	0.422	0.31	1.7796327528812e-59	no change
2369	2369	CCDC3	8.89984157038855e-64	1.1020155847103	0.03	0.008	1.94408139263567e-59	up
2370	2370	COL3A1	9.06016808590511e-64	-1.537122153	0.044	0.083	1.97910311668511e-59	down
2371	2371	CD320	9.48382855826213e-64	0.183025032168945	0.153	0.095	2.07164751026678e-59	no change
2372	2372	CDC23	1.00260825812524e-63	0.323038196855863	0.095	0.051	2.19009747904877e-59	no change
2373	2373	EP400	1.00774341873427e-63	0.425761357544449	0.103	0.057	2.20131472388313e-59	no change
2374	2374	SPON2	1.03162644765033e-63	-1.625146542	0.035	0.071	2.25348481224737e-59	down
2375	2375	NFU1	1.0354349027835e-63	0.10396389690044	0.298	0.208	2.26180400164027e-59	no change

2376	2376	RBM4	1.04056394431669e-63	0.157516593932144	0.22	0.147	2.27300787996538e-59	no change
2377	2377	NAT9	1.04265783605787e-63	0.200006265919306	0.228	0.154	2.27758177708481e-59	no change
2378	2378	EIF2AK1	1.05970172862371e-63	0.146859627930505	0.316	0.224	2.31481245600563e-59	no change
2379	2379	MTMR4	1.08398804176469e-63	0.327554335017851	0.138	0.083	2.3678634784308e-59	no change
2380	2380	ASF1A	1.19773179714465e-63	0.195758508458522	0.212	0.142	2.61632533768276e-59	no change
2381	2381	POC1B	1.24403319658812e-63	0.326590043894845	0.098	0.053	2.71746611462708e-59	no change
2382	2382	KLHL8	1.28720420414104e-63	0.292277456050356	0.109	0.061	2.8117688635257e-59	no change
2383	2383	MTOR	1.43345824394335e-63	0.588332127689711	0.069	0.032	3.13124618806986e-59	no change
2384	2384	CMTM7	1.45795868130807e-63	0.138664805137588	0.376	0.272	3.18476494344934e-59	no change
2385	2385	ATAD2	1.61670813047435e-63	0.493195079078487	0.087	0.045	3.53153724020817e-59	no change
2386	2386	BAG5	1.65051078365387e-63	0.150990472930595	0.182	0.117	3.60537575581351e-59	no change
2387	2387	TMEM87B	1.65629283787153e-63	0.204097207133905	0.172	0.11	3.61800607504657e-59	no change
2388	2388	STAG1	1.6671468784501e-63	0.196159143321205	0.147	0.09	3.64171564128641e-59	no change

2389	2389	FANCI	1.72875395870062e-63	0.785097621963384	0.047	0.018	3.77629014738564e-59	no change
2390	2390	TLK2	1.82733151720465e-63	0.329426626639399	0.123	0.072	3.99162296618183e-59	no change
2391	2391	EIF3J-AS1	1.8605418134706e-63	0.251573706126829	0.142	0.086	4.06416753734518e-59	no change
2392	2392	COA4	1.89506230221535e-63	0.134339133457196	0.537	0.404	4.13957409295922e-59	no change
2393	2393	NRIP1	2.00593809044413e-63	0.292012225200903	0.183	0.12	4.38177116476616e-59	no change
2394	2394	FAM83D	2.06087241462453e-63	0.817327422540505	0.046	0.017	4.50176970250582e-59	no change
2395	2395	PSMA6	2.06493793557444e-63	0.239736551499324	0.13	0.077	4.51065042646881e-59	no change
2396	2396	SEPT7	2.10201444825226e-63	0.106872841042484	0.637	0.497	4.59164036076225e-59	no change
2397	2397	PARP14	2.10491294501098e-63	0.187228875329288	0.196	0.128	4.59797183708198e-59	no change
2398	2398	PRDX5	2.13629294149425e-63	-0.174708545	0.974	0.938	4.66651830140004e-59	no change
2399	2399	KLHL42	2.15143291863785e-63	0.321985133781398	0.104	0.058	4.69959006747251e-59	no change
2400	2400	GOT1	2.17609316793996e-63	0.300181212411603	0.149	0.092	4.75345791604805e-59	no change
2401	2401	PPAN	2.35415579217806e-63	0.160329312684016	0.166	0.104	5.14241791243376e-59	no change

2402	2402	C6orf203	2.35595947257743e-63	0.299028992768878	0.134	0.081	5.14635787189814e-59	no change
2403	2403	PNPT1	2.3580133088463e-63	0.223920403139494	0.133	0.079	5.15084427184387e-59	no change
2404	2404	STXBP3	2.3635836658454e-63	0.128674422883224	0.21	0.14	5.1630121596727e-59	no change
2405	2405	NCAPG	2.41837135858191e-63	2.0087212517382	0.021	0.003	5.28269039568633e-59	up
2406	2406	SMCO4	2.52012187720395e-63	0.375038340801825	0.142	0.087	5.50495422856431e-59	no change
2407	2407	PLEKHA5	2.85899538843492e-63	0.129007128093617	0.26	0.179	6.24518952649724e-59	no change
2408	2408	SLC35B2	3.06103501958804e-63	0.214058237017673	0.258	0.18	6.6865248967881e-59	no change
2409	2409	NLGN4X	3.0681870580725e-63	0.729941109376859	0.05	0.02	6.70214780965357e-59	no change
2410	2410	TRIP11	3.08767295301906e-63	0.207647751929637	0.159	0.1	6.74471279857483e-59	no change
2411	2411	GRPEL1	3.10461521752157e-63	0.108973688335552	0.348	0.25	6.78172148115411e-59	no change
2412	2412	MRPL44	3.15372980797973e-63	0.240764707684638	0.161	0.101	6.88900739255092e-59	no change
2413	2413	NDUFAF6	3.52119806737303e-63	0.305966279794867	0.133	0.08	7.69170505836965e-59	no change
2414	2414	LSM2	3.7014222169458e-63	0.166497935985407	0.6	0.468	8.08538669069642e-59	no change

2415	2415	HSCB	4.11988528324867e-63	0.155841117520584	0.196	0.128	8.99947741272839e-59	no change
2416	2416	MED28	4.35178082537375e-63	0.113444830127306	0.501	0.373	9.50603003494641e-59	no change
2417	2417	TPM3	4.52715042910013e-63	0.167728994611723	0.692	0.558	9.88910739732632e-59	no change
2418	2418	C12orf56	4.6424792218839e-63	1.27523782592618	0.03	0.007	1.01410316122832e-58	up
2419	2419	PSPC1	5.16210621048323e-63	0.12559698840146	0.193	0.125	1.12761048061796e-58	no change
2420	2420	RP11-128M1.1	5.17459945452773e-63	1.05351566448708	0.033	0.009	1.13033950484704e-58	up
2421	2421	LUM	5.46673174617287e-63	-2.053457481	0.091	0.138	1.194152882634e-58	down
2422	2422	MED20	5.50452138399764e-63	0.488273105520027	0.081	0.041	1.20240765112044e-58	no change
2423	2423	C20orf194	5.6609107250705e-63	0.247604961549905	0.112	0.063	1.2365693387844e-58	no change
2424	2424	GEMIN6	5.7964022097394e-63	0.201411366566105	0.171	0.109	1.26616609869547e-58	no change
2425	2425	FAM172A	5.81937726055064e-63	0.138283094951834	0.138	0.083	1.27118476879468e-58	no change
2426	2426	BLMH	5.85672654295239e-63	0.368154219867309	0.099	0.054	1.27934334604252e-58	no change
2427	2427	PMF1	5.96444764823981e-63	0.102173484693175	0.451	0.332	1.3028739442815e-58	no change

2428	2428	NUP205	6.34314915593385e-63	0.505335306763603	0.068	0.032	1.38559750162219e-58	no change
2429	2429	LUC7L	6.89979822842842e-63	0.136401956170649	0.202	0.133	1.5071919250179e-58	no change
2430	2430	HEATR1	7.28642101513366e-63	0.449478841671489	0.079	0.039	1.5916458065458e-58	no change
2431	2431	RAE1	7.39420364867354e-63	0.270113227098516	0.144	0.088	1.61518984501625e-58	no change
2432	2432	CDC73	7.94543691824033e-63	0.184141240857791	0.215	0.144	1.73560124042042e-58	no change
2433	2433	CTSE	8.22720663287465e-63	0.279163730618963	0.416	0.334	1.79715101688514e-58	no change
2434	2434	TIMM50	8.57812679359021e-63	0.115384149246456	0.243	0.165	1.87380601679184e-58	no change
2435	2435	NUPL2	9.15175562132106e-63	0.155726910119589	0.184	0.118	1.99910949792137e-58	no change
2436	2436	CIZ1	9.53254719155703e-63	0.171295118383805	0.12	0.069	2.08228960852372e-58	no change
2437	2437	STIM2	9.74850028256976e-63	0.346311627526098	0.134	0.081	2.12946240172454e-58	no change
2438	2438	SDR42E1	9.80033811914577e-63	0.518396803538259	0.075	0.037	2.1407858587462e-58	no change
2439	2439	ERCC3	9.8942024722534e-63	0.39802667128313	0.099	0.054	2.16128958803903e-58	no change
2440	2440	C12orf45	1.01895791537685e-62	0.131058697467126	0.197	0.129	2.22581167034918e-58	no change

2441	2441	MVB12B	1.18442776877921e-62	0.736906199388181	0.05	0.02	2.5872640181213e-58	no change
2442	2442	KRT18	1.21528099820279e-62	-0.295866155	0.929	0.848	2.65465981247418e-58	no change
2443	2443	HSD17B14	1.23937590676987e-62	-0.79850363	0.159	0.213	2.7072927307481e-58	no change
2444	2444	ALDH4A1	1.35803977206922e-62	0.225521182895655	0.203	0.134	2.966502078108e-58	no change
2445	2445	STK38	1.46606000546979e-62	0.201744532297163	0.179	0.116	3.20246147594821e-58	no change
2446	2446	INPP4A	1.53994739502237e-62	0.406930479107859	0.136	0.083	3.36386108968685e-58	no change
2447	2447	MKLN1	1.61694185825865e-62	0.101621911505858	0.193	0.126	3.53204779518019e-58	no change
2448	2448	SLC35A4	1.72210769055799e-62	0.262045575870029	0.144	0.088	3.76177203925487e-58	no change
2449	2449	GNPTAB	1.84742750107658e-62	0.208421725227417	0.175	0.112	4.03552063335169e-58	no change
2450	2450	FAM127B	1.89761679117762e-62	0.142723260146417	0.454	0.339	4.14515411864839e-58	no change
2451	2451	TCEAL8	2.1326224158789e-62	0.113101777415754	0.665	0.514	4.65850040524586e-58	no change
2452	2452	PFKL	2.1641174294666e-62	0.17362098656751	0.425	0.318	4.72729811292685e-58	no change
2453	2453	EIF2S1	2.18481167899575e-62	0.101652107647216	0.292	0.205	4.77250263159832e-58	no change

2454	2454	SDAD1	2.37531553666773e-62	0.146960305690184	0.178	0.115	5.188639258297e-58	no change
2455	2455	CCNL2	2.40716093680656e-62	0.149430922086455	0.326	0.235	5.25820235036025e-58	no change
2456	2456	NXN	2.88297552042425e-62	0.514365978286866	0.076	0.037	6.29757172681473e-58	no change
2457	2457	MAPKAPK5	3.05955142280432e-62	0.194428412437403	0.175	0.113	6.68328412797375e-58	no change
2458	2458	CDK2	3.82966350594272e-62	0.353419194954291	0.116	0.067	8.36551696238129e-58	no change
2459	2459	SCAF4	3.88959155925805e-62	0.159886603741569	0.163	0.102	8.49642380204329e-58	no change
2460	2460	RHOA	4.42527948404517e-62	-0.294362643	0.774	0.727	9.66658050494826e-58	no change
2461	2461	CHAF1A	4.58439039415906e-62	0.560661612050132	0.099	0.055	1.00141423770011e-57	no change
2462	2462	SULT1A1	4.6651424302137e-62	0.116225402838928	0.149	0.091	1.01905371245588e-57	no change
2463	2463	MCTS1	4.98958912187366e-62	0.115217062525772	0.504	0.379	1.08992584778208e-57	no change
2464	2464	ELFN1-AS1	5.25958490054979e-62	1.13047193570799	0.048	0.019	1.1489037256761e-57	up
2465	2465	FRG1	5.32387884667944e-62	0.102136423457301	0.274	0.19	1.16294809526866e-57	no change
2466	2466	POLR2C	5.42828409001427e-62	0.122341858950914	0.35	0.253	1.18575437662272e-57	no change

2467	2467	SGMS1	5.43772507566866e-62	0.3131676855638	0.108	0.061	1.18781666552906e-57	no change
2468	2468	RBM12	5.47513499393052e-62	0.196877061188613	0.147	0.091	1.19598848807418e-57	no change
2469	2469	TMEM154	5.47661427839797e-62	0.124028539400186	0.298	0.211	1.19631162297325e-57	no change
2470	2470	GRIPAP1	6.00165842374867e-62	0.20748784529634	0.16	0.1	1.31100226608366e-57	no change
2471	2471	PATZ1	6.02698580115256e-62	0.35668669048879	0.119	0.069	1.31653477840377e-57	no change
2472	2472	ACSF3	6.22066236304651e-62	0.409054500157511	0.079	0.04	1.35884148658388e-57	no change
2473	2473	NUP88	6.29875115654853e-62	0.35661597702735	0.085	0.044	1.37589920263646e-57	no change
2474	2474	HIST1H2BH	6.71365349763162e-62	1.15942335796531	0.059	0.026	1.46653047002265e-57	up
2475	2475	FLII	6.8476017016853e-62	0.184568618214191	0.181	0.118	1.49579011571614e-57	no change
2476	2476	SURF2	7.17417932833131e-62	0.126698953647237	0.227	0.153	1.56712773248069e-57	no change
2477	2477	ECT2	7.21307453421403e-62	0.863193812817815	0.051	0.021	1.57562400125371e-57	no change
2478	2478	NPRL3	7.59446466900999e-62	0.252935716016323	0.159	0.101	1.65893486229854e-57	no change
2479	2479	TP53BP1	7.74521499550822e-62	0.306869881938352	0.108	0.061	1.69186476361882e-57	no change

2480	2480	DTD1	8.9649949595538e-62	0.154606869511756	0.303	0.215	1.95831349896493e-57	no change
2481	2481	BFAR	9.24154155942044e-62	0.105708615286344	0.243	0.166	2.0187223382398e-57	no change
2482	2482	SEC23B	9.42011857091509e-62	0.25451305544235	0.153	0.096	2.05773070063069e-57	no change
2483	2483	CTPS2	9.81141963090321e-62	0.529630551964972	0.07	0.034	2.1432065041745e-57	no change
2484	2484	SNX4	9.86601527794143e-62	0.166939160513092	0.189	0.124	2.15513237731353e-57	no change
2485	2485	GAMT	1.02873988911524e-61	0.181742954749357	0.167	0.107	2.24717941378334e-57	no change
2486	2486	CNOT11	1.03052706809622e-61	0.191820432525702	0.194	0.128	2.25108332754938e-57	no change
2487	2487	MPRIP	1.05529489377402e-61	0.210934165523752	0.176	0.114	2.30518616595998e-57	no change
2488	2488	ZNF397	1.06934547597353e-61	0.204590909001578	0.129	0.076	2.33587825771658e-57	no change
2489	2489	RASA2	1.07780909305596e-61	0.217719466612766	0.121	0.071	2.35436618287145e-57	no change
2490	2490	HARS2	1.1029970753136e-61	0.498535384447468	0.076	0.038	2.40938681131504e-57	no change
2491	2491	FAR2	1.11197514035804e-61	1.07093952615124	0.031	0.008	2.4289984965981e-57	up
2492	2492	ENOPH1	1.18841902103287e-61	0.187159221427383	0.249	0.173	2.5959825095442e-57	no change

2493	2493	UGT2B15	1.21030046652638e-61	0.253081055919623	0.14	0.086	2.64378033908022e-57	no change
2494	2494	PRKAR1B	1.22688112522224e-61	0.462312868161829	0.092	0.049	2.67999912993546e-57	no change
2495	2495	MECR	1.24332806583673e-61	0.28725083632934	0.142	0.087	2.71592582701376e-57	no change
2496	2496	SNX17	1.26321489930726e-61	0.104252585019537	0.346	0.248	2.75936662604678e-57	no change
2497	2497	CBWD7	1.27854083031742e-61	0.685079673425248	0.054	0.023	2.79284458974536e-57	no change
2498	2498	ABCD4	1.29701033894135e-61	0.210337105922567	0.15	0.093	2.83318938438348e-57	no change
2499	2499	PLEKHA2	1.66509270354817e-61	0.152435181018357	0.174	0.112	3.63722850163062e-57	no change
2500	2500	IWS1	1.86148057130225e-61	0.203257997189121	0.19	0.125	4.06621815995263e-57	no change
2501	2501	RAD23A	1.9025502650752e-61	0.156508764167057	0.63	0.492	4.15593079903026e-57	no change
2502	2502	UBE2D2	1.97000265780816e-61	0.137942310135665	0.701	0.553	4.30327380571614e-57	no change
2503	2503	TRIT1	2.00151396582109e-61	0.311835905505308	0.135	0.082	4.37210710693959e-57	no change
2504	2504	LRP5	2.11669598670282e-61	0.333569086802337	0.11	0.063	4.62371071335364e-57	no change
2505	2505	RBM41	2.13756429056682e-61	0.470975394202162	0.081	0.041	4.66929543631415e-57	no change

2506	2506	MYO6	2.37543791214161e-61	0.108524610932434	0.399	0.292	5.18890657528214e-57	no change
2507	2507	VIM	2.41286860715278e-61	-1.144137333	0.107	0.157	5.27067018546454e-57	down
2508	2508	MTERF3	2.60157293984861e-61	0.369606598404691	0.089	0.047	5.68287592980529e-57	no change
2509	2509	FOXK1	2.62725566043929e-61	0.271349273380346	0.096	0.052	5.73897726466358e-57	no change
2510	2510	TRMT13	2.8524797487387e-61	0.237488558150735	0.128	0.076	6.23095676314483e-57	no change
2511	2511	SLC25A38	2.93669574943968e-61	0.277894749820366	0.162	0.103	6.41491819507604e-57	no change
2512	2512	C9orf3	2.95767392073415e-61	0.117215557847006	0.204	0.135	6.46074291245167e-57	no change
2513	2513	GPALPP1	3.22521937302124e-61	0.347552911541863	0.116	0.067	7.0451691984276e-57	no change
2514	2514	ECE1	3.70194572750282e-61	0.243668739855579	0.108	0.061	8.08653024715716e-57	no change
2515	2515	ASCC3	3.70644279446059e-61	0.20612394479533	0.157	0.099	8.09635364021972e-57	no change
2516	2516	OMD	4.39063672001188e-61	-2.9325981	0.008	0.029	9.59090685119394e-57	down
2517	2517	STK4	4.7873480595407e-61	0.104231384178291	0.276	0.193	1.04574831012607e-56	no change
2518	2518	RP11-65I12.1	5.11021732100099e-61	0.618951168476698	0.053	0.022	1.11627587159946e-56	no change

2519	2519	KCNG1	5.21117989592804e-61	0.677605114520249	0.052	0.021	1.13833013646652e-56	no change
2520	2520	CCNI	5.33080558703336e-61	0.115364276175862	0.904	0.811	1.16446117243157e-56	no change
2521	2521	MXRA5	5.34657687224294e-61	0.11962922387175	0.118	0.068	1.16790625197275e-56	no change
2522	2522	UBTF	5.45086984974054e-61	0.166054314081316	0.176	0.113	1.19068800997732e-56	no change
2523	2523	FAM64A	5.59357124360462e-61	1.78033751792614	0.022	0.004	1.22185970245299e-56	up
2524	2524	UCK2	5.60370967482369e-61	0.360812181703632	0.102	0.057	1.22407434136849e-56	no change
2525	2525	PCCB	6.26515763849528e-61	0.333554769197308	0.135	0.082	1.36856103455291e-56	no change
2526	2526	BTBD10	7.1267528153418e-61	0.12468399729583	0.192	0.125	1.55676788498326e-56	no change
2527	2527	SYTL2	7.39934031856476e-61	0.209354449216878	0.169	0.109	1.61631189918729e-56	no change
2528	2528	WNK1	7.41818420087529e-61	0.126058070289084	0.258	0.179	1.6204281568392e-56	no change
2529	2529	AFG3L2	8.22887682207449e-61	0.181820244529536	0.247	0.171	1.79751585301395e-56	no change
2530	2530	HMBOX1	8.41826483498045e-61	0.278362100659122	0.116	0.067	1.83888577055313e-56	no change
2531	2531	DMAP1	8.47284796770521e-61	0.172426302692149	0.159	0.1	1.85080891006553e-56	no change

2532	2532	NUDC	8.91762545971801e-61	0.137507753521009	0.626	0.491	1.9479661054208e-56	no change
2533	2533	RBM12B	9.49479978459533e-61	0.438045924849261	0.069	0.033	2.074044064947e-56	no change
2534	2534	ATR	9.96154352440441e-61	0.57003563667845	0.06	0.027	2.1759995674709e-56	no change
2535	2535	CHEK1	1.06105267319958e-60	0.84509526001484	0.054	0.023	2.31776345933717e-56	no change
2536	2536	LINC01133	1.08261786345533e-60	-1.424868886	0.055	0.096	2.36487046093183e-56	down
2537	2537	CCHCR1	1.18404637794815e-60	0.399311034046149	0.103	0.058	2.58643090798994e-56	no change
2538	2538	ACLY	1.19312271681154e-60	0.165048206832799	0.22	0.15	2.60625726260313e-56	no change
2539	2539	TCEB3-AS1	1.19665908041501e-60	0.440629905653597	0.095	0.052	2.61398209525854e-56	no change
2540	2540	RPF1	1.58605955254839e-60	0.101801085778949	0.274	0.191	3.4645884865867e-56	no change
2541	2541	SMCR5	1.59879134580548e-60	0.136276560879348	0.196	0.129	3.49239981577748e-56	no change
2542	2542	CUX1	1.65660476316152e-60	0.103520573574372	0.239	0.163	3.61868744465002e-56	no change
2543	2543	NUP160	1.74215021591889e-60	0.2207739432733	0.136	0.082	3.80555293165322e-56	no change
2544	2544	ECHS1	1.79533515144431e-60	0.149093057767389	0.641	0.503	3.92173010481496e-56	no change

2545	2545	AGMAT	1.81904347237706e-60	0.883877961322256	0.042	0.015	3.97351856106046e-56	no change
2546	2546	CLPP	1.91108640250651e-60	0.112461176368867	0.418	0.308	4.17457713763522e-56	no change
2547	2547	RP11-277P12.20	2.06197506840321e-60	0.142518237000616	0.257	0.178	4.50417833941998e-56	no change
2548	2548	PLCE1	2.11392760526109e-60	0.328706855655184	0.112	0.065	4.61766346093232e-56	no change
2549	2549	ELOVL6	2.20652453493588e-60	0.230493770546674	0.128	0.076	4.81993219411394e-56	no change
2550	2550	CBX6	2.44061271313172e-60	0.123328184499305	0.143	0.087	5.33127441056492e-56	no change
2551	2551	MORC2	2.53327038973018e-60	0.499138809163281	0.075	0.038	5.5336758393266e-56	no change
2552	2552	HIST1H4C	2.59466786317435e-60	0.508038681321386	0.547	0.435	5.66779248031806e-56	no change
2553	2553	GRSF1	2.60268664256898e-60	0.119723752498128	0.384	0.281	5.68530870202769e-56	no change
2554	2554	DDX52	2.77500993194149e-60	0.113739152114928	0.286	0.201	6.061731695333e-56	no change
2555	2555	IFT27	2.85949274009221e-60	0.200815654528305	0.321	0.235	6.24627594145742e-56	no change
2556	2556	HMHA1	2.94260014670314e-60	0.516855254541323	0.106	0.061	6.42781576045834e-56	no change
2557	2557	PTPN11	2.98248290814261e-60	0.209253277438915	0.168	0.108	6.51493566454671e-56	no change

2558	2558	ALDH9A1	3.3131982344578e-60	0.129700544783305	0.237	0.163	7.23735022334962e-56	no change
2559	2559	PCBD2	3.45749294038693e-60	0.302971827084113	0.106	0.06	7.55254757898121e-56	no change
2560	2560	NIF3L1	3.61285191675202e-60	0.368832470814568	0.1	0.056	7.89191372695311e-56	no change
2561	2561	NUP35	3.66460990878671e-60	0.391839511691126	0.079	0.04	8.00497388475369e-56	no change
2562	2562	TMEM139	3.75401691864622e-60	-1.055478441	0.067	0.111	8.20027455709081e-56	down
2563	2563	SLC39A10	3.96677160086146e-60	0.218922731187871	0.106	0.06	8.66501588492176e-56	no change
2564	2564	THOC1	3.97931074435512e-60	0.429951873626369	0.093	0.051	8.69240638996932e-56	no change
2565	2565	NCK2	3.98685039578834e-60	0.332873628428668	0.12	0.071	8.70887600456004e-56	no change
2566	2566	OIP5	4.34264740940176e-60	1.32730121149062	0.029	0.008	9.48607900109721e-56	up
2567	2567	EMC1	4.56322468308317e-60	0.3360786727082	0.094	0.051	9.96790799772688e-56	no change
2568	2568	CNTRL	4.71165941453427e-60	0.425345405686099	0.065	0.03	1.02921488251086e-55	no change
2569	2569	WDR24	4.85122407849006e-60	0.460111603455089	0.102	0.057	1.05970138770537e-55	no change
2570	2570	FTX	5.06276771217539e-60	0.386369889035294	0.079	0.04	1.10591097904759e-55	no change

2571	2571	CDK7	5.18250627535576e-60	0.2342561578996	0.13	0.078	1.13206667078871e-55	no change
2572	2572	SEC11A	5.66903319077146e-60	0.138298572387566	0.809	0.681	1.23834361019212e-55	no change
2573	2573	TBC1D2	5.96697050355427e-60	0.429953726122034	0.093	0.051	1.3034250367964e-55	no change
2574	2574	CCDC82	6.84918846493693e-60	0.132289168973233	0.186	0.122	1.49613672828082e-55	no change
2575	2575	PCNXL4	6.86232882547784e-60	0.213963703248843	0.114	0.066	1.49900710863738e-55	no change
2576	2576	CTCF	7.34394182191153e-60	0.179943234972413	0.149	0.093	1.60421065157835e-55	no change
2577	2577	MYO10	8.13313160450976e-60	0.390619833258751	0.115	0.068	1.77660126768911e-55	no change
2578	2578	ERAP1	8.20877543430158e-60	0.141669543127191	0.134	0.08	1.79312490586884e-55	no change
2579	2579	CDCA7L	1.12681773526172e-59	0.724228203429278	0.047	0.018	2.4614206609057e-55	no change
2580	2580	SHROOM2	1.12845889261761e-59	0.421148759256914	0.086	0.046	2.46500560503391e-55	no change
2581	2581	EIF2B1	1.2397358614085e-59	0.201955151446019	0.189	0.125	2.70807901566074e-55	no change
2582	2582	CAST	1.24994424917988e-59	0.127877558094755	0.784	0.646	2.73037821790853e-55	no change
2583	2583	VEZT	1.25833753849807e-59	0.126404150518986	0.239	0.164	2.74871251909519e-55	no change

2584	2584	FAM208B	1.30801122287592e-59	0.17015441293209	0.142	0.088	2.85721971525016e-55	no change
2585	2585	AGPAT3	1.42535163740502e-59	0.35633408545135	0.108	0.062	3.11353811674753e-55	no change
2586	2586	ZNF793	1.45747354639283e-59	0.692314566874648	0.055	0.024	3.1837052147405e-55	no change
2587	2587	TFDP1	1.65341014539695e-59	0.149953823789899	0.318	0.23	3.61170912160509e-55	no change
2588	2588	BAZ2A	1.68158515870108e-59	0.135074822220345	0.272	0.191	3.67325462066665e-55	no change
2589	2589	SDC2	1.69425667129255e-59	-0.308303297	0.203	0.133	3.70093427277144e-55	no change
2590	2590	SPAG5	1.89720349152921e-59	1.44179886759165	0.026	0.006	4.14425130689641e-55	up
2591	2591	PTPN4	1.90282129293557e-59	0.234267688161127	0.117	0.068	4.15652283228846e-55	no change
2592	2592	EMD	1.93973241023849e-59	0.101120370747277	0.332	0.239	4.23715147692495e-55	no change
2593	2593	SNX1	2.00080366141162e-59	0.160959272160829	0.224	0.153	4.37055551798755e-55	no change
2594	2594	FAM117B	2.05720678948902e-59	0.399462586909816	0.087	0.046	4.49376251095981e-55	no change
2595	2595	CRIP2	2.10562956318582e-59	0.113311567701829	0.454	0.347	4.5995372178231e-55	no change
2596	2596	SLC16A7	2.12961376473257e-59	0.143670493753585	0.13	0.078	4.65192830768182e-55	no change

2597	2597	AKAP7	2.14978204908061e-59	0.478230529522092	0.097	0.054	4.69598390801167e-55	no change
2598	2598	SMNDC1	2.2015628113408e-59	0.111071057865346	0.204	0.136	4.80909380509283e-55	no change
2599	2599	KIAA1522	2.24260735678852e-59	0.121576563760992	0.251	0.174	4.89875151016885e-55	no change
2600	2600	RPIA	2.29527804895555e-59	0.167044458796229	0.283	0.202	5.0138053701385e-55	no change
2601	2601	OTUD6B	2.34202008047781e-59	0.387184470520372	0.081	0.041	5.11590866379573e-55	no change
2602	2602	INO80E	2.64606256882592e-59	0.170852403115163	0.174	0.113	5.78005907534334e-55	no change
2603	2603	ATP5SL	2.6567745115881e-59	0.217253021297061	0.197	0.131	5.80345824311304e-55	no change
2604	2604	WDR54	2.72657935233329e-59	0.195672930028194	0.219	0.149	5.95593993723684e-55	no change
2605	2605	USP36	2.74525563306694e-59	0.205681517701343	0.141	0.087	5.99673640487143e-55	no change
2606	2606	PHF8	2.82055842838196e-59	0.34063945289843	0.095	0.052	6.16122783095756e-55	no change
2607	2607	KIAA1429	3.17307015011178e-59	0.418455194576351	0.082	0.042	6.93125443590417e-55	no change
2608	2608	NUP62	3.3275635271931e-59	0.198818191870044	0.152	0.096	7.26872976880061e-55	no change
2609	2609	FBXO22	3.36975275643032e-59	0.168960582902879	0.217	0.147	7.36088792114638e-55	no change

2610	2610	GLIDR	3.56507632151212e-59	1.01929223843108	0.048	0.019	7.78755271671108e-55	up
2611	2611	HAUS6	3.7793111160819e-59	0.468328890951765	0.067	0.032	8.2555272019693e-55	no change
2612	2612	QPRT	3.89908322103718e-59	-0.718318114	0.228	0.28	8.51715738803361e-55	no change
2613	2613	SRP68	3.96199944283169e-59	0.190845925953103	0.224	0.153	8.65459158292154e-55	no change
2614	2614	TSSC1	3.99633848438621e-59	0.218482258640534	0.185	0.122	8.72960178529324e-55	no change
2615	2615	ICE1	4.11555568401879e-59	0.364908619122542	0.103	0.058	8.99001983617065e-55	no change
2616	2616	SGPL1	4.18558952101377e-59	0.139163440555648	0.277	0.195	9.14300174970249e-55	no change
2617	2617	EIF2D	4.26544577170222e-59	0.201548836198298	0.174	0.113	9.31743974370632e-55	no change
2618	2618	LAMB1	4.26906140419488e-59	0.202554854091644	0.143	0.088	9.32533773132329e-55	no change
2619	2619	LARP4B	4.35192692539024e-59	0.270926656730939	0.141	0.088	9.50634917582244e-55	no change
2620	2620	WDR75	4.460408069098e-59	0.285682335902421	0.095	0.052	9.74331538613768e-55	no change
2621	2621	LAMTOR5	4.85466174593575e-59	-0.228188501	0.861	0.789	1.06045231178221e-54	no change
2622	2622	ETV6	4.89543430748731e-59	0.245802767055288	0.142	0.088	1.06935867012753e-54	no change

2623	2623	COQ2	4.93893245345521e-59	0.388628701406251	0.097	0.054	1.07886040513276e-54	no change
2624	2624	PMS1	5.02903119909056e-59	0.252224367589639	0.097	0.053	1.09854157512934e-54	no change
2625	2625	PDIA4	5.11761176419642e-59	0.107892122600802	0.53	0.409	1.11789111377107e-54	no change
2626	2626	SMAD3	5.11839529793115e-59	0.125745438460476	0.275	0.195	1.11806226888008e-54	no change
2627	2627	CEP83	5.29398461420964e-59	0.552840724376942	0.073	0.037	1.15641799912795e-54	no change
2628	2628	SGOL2	5.99841951023072e-59	0.816406399277406	0.045	0.018	1.3102947578148e-54	no change
2629	2629	SAC3D1	6.14730071953193e-59	0.415470497226431	0.128	0.078	1.34281636917455e-54	no change
2630	2630	HAUS1	6.75652866212863e-59	0.174347067003411	0.174	0.113	1.47589612095538e-54	no change
2631	2631	SNAPC3	6.87470283312928e-59	0.305060573650944	0.112	0.065	1.50171008686876e-54	no change
2632	2632	WISP2	7.08994368295838e-59	-2.15621469	0.015	0.041	1.54872729810543e-54	down
2633	2633	CDC42BPB	7.16175926943579e-59	0.234162454238378	0.119	0.07	1.56441469481555e-54	no change
2634	2634	C1orf109	7.55830309530796e-59	0.434088801992343	0.075	0.038	1.65103572813907e-54	no change
2635	2635	LANCL3	7.65762669774852e-59	0.96849942365556	0.036	0.012	1.67273197585619e-54	no change

2636	2636	FSD1	7.70835381719902e-59	1.75867094376164	0.021	0.003	1.68381280782895e-54	up
2637	2637	CCDC86	7.84001787807429e-59	0.388378499009849	0.127	0.077	1.71257350528655e-54	no change
2638	2638	TTC32	7.87112529012928e-59	0.343335729965392	0.114	0.067	1.71936860837584e-54	no change
2639	2639	CDC27	8.40597684182671e-59	0.17780542387696	0.148	0.092	1.83620158132863e-54	no change
2640	2640	PLS1	8.74756805105807e-59	0.272688455238249	0.17	0.112	1.91081876507313e-54	no change
2641	2641	PHLDB2	9.23856837421197e-59	0.152957230434185	0.127	0.077	2.01807287566286e-54	no change
2642	2642	RP11-192H23.6	9.32752417645161e-59	0.370543049950056	0.086	0.046	2.03750438110409e-54	no change
2643	2643	PAXBP1	9.51815587018079e-59	0.243934831570663	0.104	0.059	2.07914596828229e-54	no change
2644	2644	ZC3H14	9.69960968392695e-59	0.212867753560693	0.139	0.086	2.118782739357e-54	no change
2645	2645	SEPHS1	1.04220658557216e-58	0.196713554633456	0.145	0.091	2.27659606552383e-54	no change
2646	2646	IRF2	1.13349581051118e-58	0.101493174299765	0.287	0.203	2.47600824848062e-54	no change
2647	2647	PLRG1	1.30156670893763e-58	0.16315610974037	0.139	0.085	2.84314231900337e-54	no change
2648	2648	PSMB6	1.3062052645116e-58	0.138365568361307	0.707	0.563	2.85327477979913e-54	no change

2649	2649	MED10	1.31231536265348e-58	0.207143848054338	0.345	0.258	2.86662167818025e-54	no change
2650	2650	NOL4L	1.32839264962666e-58	0.273965628980116	0.128	0.077	2.90174090384447e-54	no change
2651	2651	ASUN	1.35270253916886e-58	0.491490132595908	0.084	0.045	2.95484342656047e-54	no change
2652	2652	TDP2	1.3594811332199e-58	0.118989854240122	0.273	0.192	2.96965058740555e-54	no change
2653	2653	MMS22L	1.45311847074553e-58	0.617978077875807	0.05	0.021	3.17419198749653e-54	no change
2654	2654	C20orf27	1.46363408894013e-58	0.164073863543238	0.316	0.23	3.19716230388083e-54	no change
2655	2655	SMIM22	1.51021147472736e-58	-0.250824677	0.91	0.836	3.29890594539444e-54	no change
2656	2656	KIAA1191	1.54476511023081e-58	0.135675831651322	0.207	0.139	3.37438490678817e-54	no change
2657	2657	LASP1	1.67530558992968e-58	0.117124322081288	0.285	0.201	3.6595375306424e-54	no change
2658	2658	UBAP2	1.69829717041798e-58	0.175703291968946	0.138	0.085	3.70976033906104e-54	no change
2659	2659	AMN	1.80653641333535e-58	0.23127783001192	0.091	0.049	3.94619814128973e-54	no change
2660	2660	ABR	1.8185253176189e-58	0.319022111967603	0.086	0.045	3.97238670380673e-54	no change
2661	2661	DIP2B	1.89822582676151e-58	0.370017021200549	0.084	0.044	4.14648449597784e-54	no change

2662	2662	GPR27	1.90692567797063e-58	0.974642122066879	0.039	0.013	4.16548845095904e-54	no change
2663	2663	PARP4	2.0330442311172e-58	0.218311199920714	0.134	0.081	4.44098181845242e-54	no change
2664	2664	ZMYM4	2.22187650466307e-58	0.339905391963852	0.106	0.061	4.853467036786e-54	no change
2665	2665	PARD3	2.23406266209329e-58	0.141117345585749	0.153	0.097	4.88008647907658e-54	no change
2666	2666	PTPRU	2.30570007255667e-58	0.316468871317317	0.113	0.066	5.0365712384928e-54	no change
2667	2667	ARFGAP2	2.30804090421096e-58	0.10541657343089	0.191	0.126	5.04168455115842e-54	no change
2668	2668	NMRAL1	2.50287501928009e-58	0.137875989041296	0.349	0.256	5.46728019211543e-54	no change
2669	2669	CADM1	2.54727773526262e-58	0.551423660353277	0.052	0.022	5.56427348490766e-54	no change
2670	2670	PPP6R3	2.55579782905561e-58	0.157083344620021	0.177	0.116	5.58288477778908e-54	no change
2671	2671	ADPRHL2	2.57261030703495e-58	0.181821421404948	0.209	0.142	5.61960995468715e-54	no change
2672	2672	MLLT3	2.91590222333358e-58	0.267079332510126	0.096	0.053	6.36949681664987e-54	no change
2673	2673	WBSCR16	3.14792213340191e-58	0.229302786477357	0.147	0.092	6.87632110820312e-54	no change
2674	2674	ITPR3	3.27017024596384e-58	0.412642610295088	0.074	0.037	7.14335988528342e-54	no change

2675	2675	ZC2HC1A	3.35910231296321e-58	0.126351196145163	0.194	0.128	7.33762309243683e-54	no change
2676	2676	PIGX	3.40441584286851e-58	0.17899491032589	0.181	0.12	7.43660596716197e-54	no change
2677	2677	C16orf62	3.50436486024925e-58	0.325338984717721	0.095	0.053	7.65493460072846e-54	no change
2678	2678	CLPTM1L	3.64664921476674e-58	0.251247216802585	0.198	0.134	7.96574054473646e-54	no change
2679	2679	DPF2	3.64788967896112e-58	0.204396831053042	0.184	0.122	7.96845021472267e-54	no change
2680	2680	CBWD2	3.75483674956854e-58	0.352931551293758	0.097	0.054	8.20206539575752e-54	no change
2681	2681	EPSTI1	4.03923274087074e-58	-0.983659839	0.112	0.161	8.82329999915805e-54	no change
2682	2682	FARSB	4.44253075761522e-58	0.21817875102969	0.149	0.094	9.70426418693469e-54	no change
2683	2683	CCND3	4.84780304259141e-58	0.170378441469474	0.214	0.146	1.05895409662367e-53	no change
2684	2684	C5orf28	4.88816925799447e-58	0.247925302895069	0.162	0.105	1.06777169271631e-53	no change
2685	2685	NIPAL3	5.68220251425749e-58	0.274938994401395	0.126	0.076	1.24122031721441e-53	no change
2686	2686	EIF2B4	5.89602688117736e-58	0.158543268550099	0.153	0.097	1.28792811192438e-53	no change
2687	2687	ADCK3	6.01628886115942e-58	0.440684097419048	0.076	0.039	1.31419813883166e-53	no change

2688	2688	CAD	6.25284276716178e-58	0.665138115547595	0.049	0.02	1.36587097405882e-53	no change
2689	2689	CLUH	6.31997881417811e-58	0.355369245033586	0.124	0.075	1.38053617216907e-53	no change
2690	2690	TRIM38	6.85506420931652e-58	0.105878928219138	0.249	0.173	1.4974202258831e-53	no change
2691	2691	ALOX5	6.9563816695632e-58	0.121842161317229	0.297	0.213	1.51955201189939e-53	no change
2692	2692	HLA-E	7.15939988294581e-58	-0.325927946	0.806	0.736	1.56389931043068e-53	no change
2693	2693	GTF3C4	7.19279657756015e-58	0.603102496519641	0.051	0.021	1.57119448440224e-53	no change
2694	2694	E2F8	7.30578341735309e-58	0.67841423373106	0.05	0.021	1.59587532968661e-53	no change
2695	2695	UBXN2A	8.00805652562839e-58	0.166412044783369	0.165	0.106	1.74927986745826e-53	no change
2696	2696	ZNF436	8.89363597103241e-58	0.353942157263118	0.08	0.041	1.94272584151232e-53	no change
2697	2697	THUMPD3	1.06155384930369e-57	0.220166612837592	0.158	0.101	2.31885822841898e-53	no change
2698	2698	SLC7A2	1.09560777426731e-57	0.760113883637177	0.044	0.017	2.39324562210951e-53	no change
2699	2699	ZWILCH	1.16156893582539e-57	0.405547306258568	0.076	0.039	2.53733118341699e-53	no change
2700	2700	COL6A3	1.2428405535159e-57	-1.641268647	0.031	0.063	2.71486090510012e-53	down

2701	2701	PRKD3	1.42319429007138e-57	0.15321286818283	0.128	0.077	3.10882560723192e-53	no change
2702	2702	FAM83F	1.44584154985497e-57	0.387842578979311	0.11	0.064	3.15829628150319e-53	no change
2703	2703	SEC16A	1.44759852598406e-57	0.233406209297787	0.113	0.066	3.16213422015957e-53	no change
2704	2704	MYCL	1.4786547536648e-57	0.167457222193201	0.266	0.189	3.2299734439054e-53	no change
2705	2705	MTHFD2L	1.70094957312594e-57	0.26812045927897	0.215	0.149	3.71555424753631e-53	no change
2706	2706	PDE7A	1.78902005944488e-57	0.285870305657252	0.135	0.084	3.9079354178514e-53	no change
2707	2707	HSD17B11	1.952167540389e-57	0.175356813853064	0.533	0.418	4.26431477522574e-53	no change
2708	2708	WDR70	1.98502447101413e-57	0.177778606225365	0.162	0.104	4.33608745448327e-53	no change
2709	2709	RALGPS2	2.01403213108902e-57	0.202599065578353	0.145	0.091	4.39945178715085e-53	no change
2710	2710	KLC1	2.07098741485795e-57	0.11499188323824	0.185	0.122	4.52386490901571e-53	no change
2711	2711	GIGYF2	2.11595836265986e-57	0.244456756679282	0.156	0.1	4.62209944739421e-53	no change
2712	2712	STK39	2.19849875082839e-57	0.257563364978005	0.102	0.058	4.80240067130954e-53	no change
2713	2713	RSRC2	2.32761423388608e-57	0.113887610591141	0.642	0.503	5.08444053250075e-53	no change

2714	2714	MECP2	2.36159087883148e-57	0.100259371527128	0.218	0.148	5.15865911571948e-53	no change
2715	2715	F10	2.50320127896884e-57	-2.024147801	0.018	0.045	5.46799287377953e-53	down
2716	2716	PUS1	2.63099235796286e-57	0.300407554120012	0.127	0.077	5.74713970673406e-53	no change
2717	2717	ALKBH5	2.72277913230339e-57	0.129959654218629	0.242	0.168	5.94763873660353e-53	no change
2718	2718	FBXO9	2.73721999043295e-57	0.175618589793135	0.183	0.121	5.97918334710173e-53	no change
2719	2719	TRIM4	3.06931718125104e-57	0.152965703382888	0.126	0.076	6.70461645072477e-53	no change
2720	2720	BMS1	3.12972214689789e-57	0.244904773186152	0.143	0.09	6.83656505768374e-53	no change
2721	2721	KIFC1	3.3770175031429e-57	1.11687206970374	0.038	0.013	7.37675703386536e-53	up
2722	2722	RNPC3	3.45049685875083e-57	0.294925917815487	0.1	0.056	7.53726533825532e-53	no change
2723	2723	KRT17	3.87874689238236e-57	0.171390548327807	0.897	0.857	8.47273471172002e-53	no change
2724	2724	BASP1	3.9988787935892e-57	0.202765056090934	0.334	0.246	8.73515083671626e-53	no change
2725	2725	EFEMP2	4.08268222051651e-57	-1.500502465	0.045	0.081	8.91821104249626e-53	down
2726	2726	POLR2D	4.11817075253192e-57	0.275087266735105	0.129	0.079	8.99573219183072e-53	no change

2727	2727	SCAI	4.29675521438934e-57	0.567573182050258	0.055	0.024	9.38583209031207e-53	no change
2728	2728	PCAT7	4.419017379636e-57	0.803129175130456	0.051	0.022	9.65290156407687e-53	no change
2729	2729	MBTPS1	4.4413558743976e-57	0.192399079280372	0.174	0.114	9.70169777203412e-53	no change
2730	2730	PRPSAP1	4.80358119831842e-57	0.154294486216511	0.236	0.163	1.04929427696068e-52	no change
2731	2731	TIMM21	5.16270021335211e-57	0.385727988203113	0.103	0.059	1.12774023460463e-52	no change
2732	2732	TRIO	5.24066050254511e-57	0.338091846435463	0.087	0.047	1.14476988017595e-52	no change
2733	2733	PHLDA3	5.75823574945862e-57	-0.617248897	0.478	0.478	1.25782901711174e-52	no change
2734	2734	CERS5	6.01004779988265e-57	0.226158668939867	0.128	0.078	1.31283484140637e-52	no change
2735	2735	ZNF566	6.61414370767583e-57	0.629969867259203	0.056	0.025	1.44479355150471e-52	no change
2736	2736	TMEM57	7.60455944997761e-57	0.233593463595001	0.134	0.083	1.66113996625311e-52	no change
2737	2737	BPHL	7.94214452709596e-57	0.268748963924981	0.149	0.094	1.73488205049884e-52	no change
2738	2738	TUBGCP4	8.0763560687434e-57	0.279455816190343	0.098	0.055	1.76419921965631e-52	no change
2739	2739	THADA	8.27911583554186e-57	0.277987650871518	0.1	0.057	1.80849006311576e-52	no change

2740	2740	ZNF275	8.36115888485542e-57	0.442676925791305	0.07	0.035	1.82641154680782e-52	no change
2741	2741	UNC45A	8.40609942735393e-57	0.16606977664104	0.179	0.118	1.83622835891119e-52	no change
2742	2742	YRDC	9.32712800674524e-57	0.218079523539208	0.146	0.092	2.03741784179343e-52	no change
2743	2743	RNF113A	9.34251073163707e-57	0.22197434676155	0.143	0.09	2.0407780442188e-52	no change
2744	2744	SMC6	1.01265932122676e-56	0.318015464788322	0.105	0.061	2.21205302128773e-52	no change
2745	2745	GLYR1	1.01305460868097e-56	0.178863184048702	0.172	0.113	2.21291648720271e-52	no change
2746	2746	DTWD1	1.05212431690593e-56	0.103295792941314	0.162	0.104	2.29826035784932e-52	no change
2747	2747	BPGM	1.05616655502255e-56	-0.989771072	0.28	0.319	2.30709022279126e-52	no change
2748	2748	HAGLR	1.0743183909907e-56	0.461117170326415	0.076	0.039	2.34674109328009e-52	no change
2749	2749	PRRG4	1.07744402175748e-56	0.101875862095634	0.378	0.28	2.35356872112703e-52	no change
2750	2750	CREBBP	1.08408448673903e-56	0.17688651127582	0.158	0.102	2.36807415283273e-52	no change
2751	2751	NUP54	1.12251075144825e-56	0.143154584281234	0.172	0.113	2.45201248546357e-52	no change
2752	2752	CASC9	1.1545803418654e-56	0.340140397717266	0.072	0.036	2.52206529877079e-52	no change

2753	2753	GHITM	1.17876935777275e-56	0.120138575492065	0.648	0.506	2.5749037851188e-52	no change
2754	2754	CDCA4	1.30155753546975e-56	0.34792752609569	0.098	0.055	2.84312228048013e-52	no change
2755	2755	NAA38	1.36352335756906e-56	0.120108566719763	0.768	0.618	2.97848042227384e-52	no change
2756	2756	CXorf38	1.37243525039176e-56	0.31962350308592	0.096	0.054	2.99794756095576e-52	no change
2757	2757	JPH1	1.38053441814968e-56	0.625807901268893	0.051	0.022	3.01563938300617e-52	no change
2758	2758	RDH14	1.45205291000267e-56	0.133241336924985	0.2	0.134	3.17186437660984e-52	no change
2759	2759	FRY	1.49264258327015e-56	0.272758772795249	0.133	0.082	3.26052845889533e-52	no change
2760	2760	PARPBP	1.55028491975247e-56	0.894774345132331	0.046	0.019	3.38644237870729e-52	no change
2761	2761	EIF2B3	1.55782409915093e-56	0.231488007954684	0.119	0.071	3.40291096218528e-52	no change
2762	2762	UBP1	1.59657833289109e-56	0.217841982173653	0.155	0.1	3.4875657103673e-52	no change
2763	2763	NUP210	1.59784177403361e-56	0.643694329503681	0.06	0.028	3.49032557119903e-52	no change
2764	2764	ATP7A	1.68842742610609e-56	0.183455360402853	0.1	0.056	3.68820086958615e-52	no change
2765	2765	KDM2A	1.78483835414478e-56	0.129950254555236	0.198	0.133	3.89880090079385e-52	no change

2766	2766	PSME3	1.80500490207218e-56	0.160740051190984	0.294	0.213	3.94285270808646e-52	no change
2767	2767	ANLN	1.87678932051255e-56	0.871679398817159	0.04	0.015	4.09965859172761e-52	no change
2768	2768	IFIT1	1.95551443641163e-56	-1.648907525	0.023	0.052	4.27162573489757e-52	down
2769	2769	DCN	1.99247702472708e-56	-1.940797445	0.091	0.134	4.35236681281384e-52	down
2770	2770	MET	2.00350694175094e-56	0.284213763765309	0.115	0.068	4.37646056356075e-52	no change
2771	2771	CRCT1	2.04638982033967e-56	-2.652884029	0.072	0.113	4.47013392354997e-52	down
2772	2772	NUMA1	2.09428686376736e-56	0.161319329809677	0.211	0.144	4.57476022521341e-52	no change
2773	2773	CCNT2	2.15885264681708e-56	0.177190443868416	0.154	0.098	4.71579772170723e-52	no change
2774	2774	MELK	2.25018561155726e-56	1.48437614302188	0.026	0.007	4.91530544988568e-52	up
2775	2775	USP33	2.27123391505536e-56	0.19994238075452	0.162	0.104	4.96128336404692e-52	no change
2776	2776	DHX8	2.40557861466007e-56	0.303420401364734	0.107	0.062	5.25474592586345e-52	no change
2777	2777	PUSL1	2.42091718036439e-56	0.252623295790736	0.192	0.131	5.28825148878796e-52	no change
2778	2778	ZNF704	2.73269906850068e-56	0.206194802667824	0.119	0.071	5.96930784523289e-52	no change

2779	2779	SZRD1	2.80614464061478e-56	0.126053371486606	0.322	0.235	6.12974235295893e-52	no change
2780	2780	PHACTR4	2.85026391245747e-56	0.137900368440923	0.185	0.123	6.2261164903721e-52	no change
2781	2781	ING4	2.92259315517839e-56	0.241445854039168	0.159	0.102	6.38411248817167e-52	no change
2782	2782	ATP10B	3.01645482054093e-56	0.353960676351618	0.062	0.029	6.58914390998961e-52	no change
2783	2783	WNT10A	3.01985780349563e-56	0.231542990740896	0.103	0.059	6.59657738595586e-52	no change
2784	2784	TFCP2L1	3.07181447182018e-56	0.530648357009658	0.061	0.028	6.71007153224399e-52	no change
2785	2785	FAM13B	3.21410570131443e-56	0.224790955533419	0.1	0.057	7.02089249395124e-52	no change
2786	2786	CTD-2008P7.9	3.24877468131053e-56	-1.144504062	0.053	0.091	7.09662341385473e-52	down
2787	2787	KCNQ1	3.34055653186274e-56	0.365971875434699	0.084	0.045	7.29711168820096e-52	no change
2788	2788	MSMO1	3.6383110516994e-56	-0.750972142	0.361	0.389	7.94752666133216e-52	no change
2789	2789	PAMR1	3.65976239464304e-56	-1.9647532	0.02	0.047	7.99438497485826e-52	down
2790	2790	ZDHHC2	3.66179211898328e-56	0.228042769662657	0.133	0.082	7.99881870470707e-52	no change
2791	2791	ZNF771	3.66387207174023e-56	0.311296677679751	0.09	0.05	8.00336215350936e-52	no change

2792	2792	CA4	4.01777785583076e-56	1.12765766218364	0.028	0.008	8.77643394827671e-52	up
2793	2793	NELFB	4.24118961401476e-56	0.366119106886799	0.089	0.049	9.26445459285384e-52	no change
2794	2794	R3HDM1	4.40477226294532e-56	0.303497192934793	0.091	0.05	9.62178453117777e-52	no change
2795	2795	ZNF37A	4.41725083600614e-56	0.236000458441179	0.089	0.049	9.64904272617181e-52	no change
2796	2796	MFN2	5.20906119895001e-56	0.31671794186473	0.122	0.074	1.13786732829864e-51	no change
2797	2797	FBXW2	5.41161252842517e-56	0.193012486713348	0.148	0.094	1.18211264070919e-51	no change
2798	2798	MAX	5.49734434221025e-56	0.104574638620965	0.331	0.24	1.20083989811241e-51	no change
2799	2799	STYXL1	5.68789367295417e-56	0.16010394637343	0.195	0.131	1.24246349392011e-51	no change
2800	2800	FOXF2	5.93161524642841e-56	0.872954769783596	0.05	0.022	1.29570203442982e-51	no change
2801	2801	TULP4	6.05195479332458e-56	0.14832497745313	0.17	0.111	1.32198900505382e-51	no change
2802	2802	CEP89	6.07767533219768e-56	0.285725457874765	0.113	0.066	1.32760739956526e-51	no change
2803	2803	IMPDH1	6.5199184157164e-56	0.363790112786424	0.09	0.049	1.42421097872909e-51	no change
2804	2804	POLK	6.6692722914855e-56	0.167009957780295	0.116	0.069	1.45683583935209e-51	no change

2805	2805	NIPAL1	6.81759100364946e-56	0.252042538893698	0.177	0.119	1.48923457883719e-51	no change
2806	2806	MBD4	6.91474636037604e-56	0.134418809186478	0.192	0.128	1.51045719496054e-51	no change
2807	2807	BOD1L1	6.94238403958397e-56	0.16933831873526	0.175	0.116	1.51649436960672e-51	no change
2808	2808	HOXB2	6.97102570547222e-56	0.131999244532897	0.236	0.163	1.52275085510335e-51	no change
2809	2809	UBA6	7.01080017872188e-56	0.107007559795841	0.268	0.19	1.53143919104001e-51	no change
2810	2810	ZNF644	7.10175147451388e-56	0.113461899960695	0.238	0.165	1.55130659209281e-51	no change
2811	2811	ZNF562	7.23338758492448e-56	0.109580805269034	0.265	0.188	1.5800611840509e-51	no change
2812	2812	BMP2	8.00542729064113e-56	-0.962562793	0.294	0.329	1.74870553736765e-51	no change
2813	2813	RGMB	8.33902499684461e-56	0.265624039311635	0.093	0.051	1.82157662031074e-51	no change
2814	2814	PTGES3	8.84147154989686e-56	0.191878312351695	0.894	0.801	1.93133104535947e-51	no change
2815	2815	PTPN3	9.37844690527909e-56	0.347513197039063	0.091	0.05	2.04862794198916e-51	no change
2816	2816	BANF1	9.51506157538479e-56	0.150910959647876	0.775	0.646	2.07847005052705e-51	no change
2817	2817	OLFML3	9.62655622466452e-56	-1.674703329	0.031	0.062	2.10282494171572e-51	down

2818	2818	TMTC3	1.0552005378193e-55	0.18400886378963	0.149	0.094	2.30498005481248e-51	no change
2819	2819	DAB1	1.08606953135528e-55	1.09391620172879	0.03	0.009	2.37241028429247e-51	up
2820	2820	TMEM181	1.0978023699167e-55	0.126314945352737	0.218	0.15	2.39803949684604e-51	no change
2821	2821	PPIG	1.10282938213421e-55	0.102792843124535	0.605	0.47	2.40902050233396e-51	no change
2822	2822	SLC18B1	1.1175997528343e-55	0.230053572794819	0.121	0.073	2.44128490009125e-51	no change
2823	2823	PPWD1	1.13906022241844e-55	0.215116174850259	0.096	0.054	2.48816314985084e-51	no change
2824	2824	MAPK1	1.15189611737719e-55	0.115345654337159	0.215	0.148	2.51620187879874e-51	no change
2825	2825	SF3B4	1.15519459620879e-55	0.101992623519798	0.278	0.196	2.52340707595848e-51	no change
2826	2826	GAPVD1	1.15674108047373e-55	0.251205267878031	0.095	0.053	2.52678521618681e-51	no change
2827	2827	APOL6	1.19574231478347e-55	0.205630075753425	0.187	0.126	2.61197951241301e-51	no change
2828	2828	LIG1	1.26162213175328e-55	0.52876125884277	0.064	0.031	2.75588738460186e-51	no change
2829	2829	PSMG1	1.46203635222486e-55	0.116813300604804	0.25	0.174	3.19367220779998e-51	no change
2830	2830	UBR2	1.52769762692336e-55	0.297276841166765	0.106	0.062	3.33710269625138e-51	no change

2831	2831	TRIB1	1.5365066847956e-55	-0.557062842	0.457	0.473	3.35634520226751e-51	no change
2832	2832	PRR11	1.87173236320708e-55	1.04731902264149	0.031	0.009	4.08861217418955e-51	up
2833	2833	RPUSD1	1.98569136163794e-55	0.303595170883482	0.121	0.074	4.33754421036192e-51	no change
2834	2834	ZZZ3	2.02333691587915e-55	0.300765589249147	0.117	0.07	4.41977715904642e-51	no change
2835	2835	JMY	2.03574346248073e-55	0.365519106755996	0.073	0.037	4.4468780194429e-51	no change
2836	2836	RMI1	2.04561218537772e-55	0.706348227607812	0.046	0.019	4.4684352577391e-51	no change
2837	2837	FAM98B	2.2985199279816e-55	0.271577854111241	0.111	0.066	5.020886930683e-51	no change
2838	2838	KIAA1109	2.34374847661138e-55	0.150296231130435	0.142	0.089	5.11968417230989e-51	no change
2839	2839	RPN2	2.55899917149279e-55	0.138402425512846	0.563	0.442	5.58987779020884e-51	no change
2840	2840	TMEM39B	2.58172532652076e-55	0.245085058897443	0.106	0.062	5.63952080325194e-51	no change
2841	2841	HAS3	2.68122285378373e-55	0.141516651987928	0.222	0.153	5.85686320180518e-51	no change
2842	2842	SNRNP48	2.73760263889093e-55	0.273683799149818	0.105	0.061	5.98001920439334e-51	no change
2843	2843	THAP3	2.76707637464331e-55	0.414018537016819	0.103	0.059	6.04440163277085e-51	no change

2844	2844	RBM18	2.79896541172768e-55	0.244595445690646	0.141	0.089	6.11406004537794e-51	no change
2845	2845	GABRP	2.81557702280746e-55	-3.188127766	0.006	0.024	6.15034644862061e-51	down
2846	2846	ROCK2	2.83726578601578e-55	0.119256810029149	0.175	0.115	6.19772338297287e-51	no change
2847	2847	SERPINF1	2.92774290645132e-55	-1.889003752	0.089	0.131	6.39536160485226e-51	down
2848	2848	TMEM121	2.96020235610288e-55	0.995321528466899	0.032	0.01	6.46626602667114e-51	no change
2849	2849	PPP2R4	3.01802947046073e-55	0.183611413149689	0.153	0.098	6.59258357527442e-51	no change
2850	2850	CNTLN	3.04749238385968e-55	0.414983786321618	0.054	0.024	6.65694236330308e-51	no change
2851	2851	TARBP1	3.57666457550078e-55	0.363742123761131	0.077	0.04	7.8128660987239e-51	no change
2852	2852	GPS1	3.61320855191267e-55	0.155329018785333	0.288	0.209	7.89269276079803e-51	no change
2853	2853	CIAO1	3.6708424177387e-55	0.147214878140129	0.25	0.177	8.01858817730842e-51	no change
2854	2854	BOP1	3.71506739414464e-55	0.309613567720993	0.098	0.055	8.11519321576956e-51	no change
2855	2855	KLF13	4.08177229094883e-55	0.14286010793059	0.098	0.055	8.91622339234862e-51	no change
2856	2856	ICAM1	4.13495028078086e-55	-1.404778623	0.062	0.101	9.03238539333771e-51	down

2857	2857	PDCD7	4.27082731313261e-55	0.118793968339475	0.235	0.163	9.32919518280687e-51	no change
2858	2858	OCIAD1	4.64719648881355e-55	0.111386022253547	0.566	0.432	1.01513360101643e-50	no change
2859	2859	PHLPP1	4.70565627896977e-55	0.443141924581379	0.069	0.035	1.02790355757816e-50	no change
2860	2860	TNXB	4.71503957379998e-55	-1.990635327	0.025	0.053	1.02995324450087e-50	down
2861	2861	NOM1	5.02208992116476e-55	0.226726393031722	0.115	0.069	1.09702532237923e-50	no change
2862	2862	PRAF2	5.07921754740674e-55	0.148958764785736	0.208	0.141	1.10950428105553e-50	no change
2863	2863	IL32	5.3258119614328e-55	-0.846956502	0.146	0.196	1.16337036485538e-50	no change
2864	2864	YES1	5.83132167484046e-55	0.181075742434668	0.204	0.14	1.27379390665215e-50	no change
2865	2865	UBE4B	5.99593864593357e-55	0.270275635903664	0.113	0.067	1.30975283781773e-50	no change
2866	2866	ZBED5-AS1	6.09822914503397e-55	0.140213023342203	0.16	0.103	1.33209717444122e-50	no change
2867	2867	FAM107B	6.15852518846097e-55	0.200648515796851	0.363	0.274	1.34526824216741e-50	no change
2868	2868	CSTF1	6.31043302651606e-55	0.223110290893167	0.12	0.073	1.37845099031217e-50	no change
2869	2869	ZNF812P	6.38998069706889e-55	0.116153154747561	0.139	0.086	1.39582738346773e-50	no change

2870	2870	CEP68	7.23984654558604e-55	0.370648291499367	0.078	0.041	1.58147207941781e-50	no change
2871	2871	TRIM16L	7.72447443989486e-55	0.608705988152024	0.065	0.032	1.68733419665063e-50	no change
2872	2872	CAMSAP1	7.75263650033614e-55	0.350165308731067	0.095	0.054	1.69348591713343e-50	no change
2873	2873	BAALC	8.16421077923578e-55	0.607786342133781	0.073	0.038	1.78339020261626e-50	no change
2874	2874	ACP1	8.22958740772101e-55	0.110950915054129	0.574	0.441	1.79767107334258e-50	no change
2875	2875	BUB1B	8.29416832134089e-55	1.57361274745545	0.02	0.004	1.8117781281137e-50	up
2876	2876	TMEM237	8.55959787354141e-55	0.144734004086664	0.192	0.13	1.86975855949639e-50	no change
2877	2877	UPF1	9.27228901274071e-55	0.401914394762592	0.107	0.064	2.02543881194308e-50	no change
2878	2878	ZCCHC6	9.86177225989814e-55	0.194496583267286	0.123	0.075	2.15420553245215e-50	no change
2879	2879	KIAA0100	1.02132889746058e-54	0.290268869652445	0.115	0.069	2.2309908436129e-50	no change
2880	2880	SKA2	1.04569716022487e-54	0.212904443795001	0.25	0.178	2.28422087679521e-50	no change
2881	2881	DBF4	1.185244286692e-54	0.37514160042288	0.082	0.044	2.58904761985e-50	no change
2882	2882	PM20D2	1.22362299629088e-54	0.2566661319071	0.103	0.06	2.67288207309781e-50	no change

2883	2883	KRT23	1.308386562126e-54	-1.444179431	0.146	0.192	2.85803960630804e-50	down
2884	2884	FTSJ2	1.42822109242501e-54	0.244950344363648	0.139	0.088	3.1198061542932e-50	no change
2885	2885	TNFAIP6	1.66982042546864e-54	-1.418318755	0.034	0.066	3.64755573739369e-50	down
2886	2886	PLK1	1.73265411951214e-54	1.33773468739952	0.033	0.011	3.78480965866231e-50	up
2887	2887	RNF217	1.88978042585543e-54	0.377763433057988	0.058	0.027	4.12803636223859e-50	no change
2888	2888	CLUAP1	1.91883132939133e-54	0.370972919089834	0.088	0.048	4.19149515592243e-50	no change
2889	2889	NUDT1	2.17063675459693e-54	0.185002797265948	0.223	0.156	4.74153892674153e-50	no change
2890	2890	ZNHIT6	2.43966642489157e-54	0.328794312343996	0.102	0.059	5.32920733853314e-50	no change
2891	2891	VWA8	2.76098838197945e-54	0.305477798802337	0.085	0.046	6.03110302159591e-50	no change
2892	2892	CASP9	2.7787750338336e-54	0.387233392754248	0.089	0.049	6.06995618390612e-50	no change
2893	2893	C16orf91	2.91676639430665e-54	0.180504345949125	0.192	0.13	6.37138451172344e-50	no change
2894	2894	HDAC10	3.05261496881116e-54	0.170489757350886	0.123	0.075	6.66813213787111e-50	no change
2895	2895	KRAS	3.06684822543005e-54	0.111195155078567	0.367	0.274	6.69922326362941e-50	no change

2896	2896	RCL1	3.15642127779757e-54	0.320868478934536	0.078	0.041	6.89488663922101e-50	no change
2897	2897	PDE10A	3.29574476045115e-54	0.477754125029686	0.088	0.049	7.1992248547295e-50	no change
2898	2898	PPME1	3.40884597998387e-54	0.225895920211635	0.184	0.124	7.44628315867677e-50	no change
2899	2899	YARS2	3.40957271754085e-54	0.237697191240855	0.12	0.073	7.44787064419623e-50	no change
2900	2900	SRPRA	3.43873478881935e-54	0.120472898662051	0.2	0.136	7.51157227269699e-50	no change
2901	2901	PPIB	3.89754315790641e-54	0.117913255264353	0.872	0.773	8.51379327413076e-50	no change
2902	2902	CCDC22	4.27117432488568e-54	0.388486138613779	0.096	0.054	9.32995319528029e-50	no change
2903	2903	NUTM2B-AS1	4.48042610838317e-54	0.128295953430277	0.224	0.155	9.78704279115219e-50	no change
2904	2904	NUP62CL	4.6164384017795e-54	0.63400492076635	0.045	0.018	1.00841480448471e-49	no change
2905	2905	COX7B	4.8127380810644e-54	0.11845444326291	0.956	0.888	1.05129450642771e-49	no change
2906	2906	FBXO18	4.93931612571873e-54	0.116731189388168	0.155	0.1	1.078944214502e-49	no change
2907	2907	BUB3	5.42441468061283e-54	0.112475059848129	0.375	0.278	1.18490914283307e-49	no change
2908	2908	MCM10	5.5752362893075e-54	1.14698638688969	0.026	0.007	1.21785461503633e-49	up

2909	2909	TNIP2	6.07875860642502e-54	0.175125758823167	0.27	0.196	1.32784402998748e-49	no change
2910	2910	DNAAF5	6.18367315894694e-54	0.430721025445346	0.066	0.033	1.35076156484037e-49	no change
2911	2911	SRFBP1	6.6229291274195e-54	0.188920156333141	0.117	0.07	1.44671263859352e-49	no change
2912	2912	CEP41	6.67695485925989e-54	0.322550737336516	0.106	0.062	1.45851401945673e-49	no change
2913	2913	EMP3	7.09100276028953e-54	-1.380923714	0.099	0.143	1.54895864295765e-49	down
2914	2914	GLOD4	7.65669814025697e-54	0.114638992965565	0.245	0.173	1.67252914175773e-49	no change
2915	2915	UBE2R2	7.80457409517249e-54	0.109444061457559	0.268	0.191	1.70483116534948e-49	no change
2916	2916	KCTD3	7.91703614615953e-54	0.1852243107774	0.111	0.066	1.72939737576709e-49	no change
2917	2917	PRKAR1A	8.11018811667914e-54	0.103395426500388	0.554	0.43	1.77158949220739e-49	no change
2918	2918	RICTOR	8.28039260590549e-54	0.116426507622903	0.209	0.144	1.808768960834e-49	no change
2919	2919	SFSWAP	9.10974051940945e-54	0.15810785603112	0.178	0.118	1.9899317190598e-49	no change
2920	2920	GSTO2	9.16893467167035e-54	0.129619645907768	0.31	0.227	2.00286208967967e-49	no change
2921	2921	PDRG1	9.25153146628169e-54	0.243438576930477	0.147	0.094	2.02090453349457e-49	no change

2922	2922	KIF14	9.57357145373633e-54	1.15352486594987	0.028	0.008	2.09125094835416e-49	up
2923	2923	ORC4	1.00483063042634e-53	0.103425796235487	0.204	0.139	2.1949520291033e-49	no change
2924	2924	JADE2	1.03840713386438e-53	0.3441815635971	0.069	0.034	2.26829654321335e-49	no change
2925	2925	CSAD	1.08013932081923e-53	0.230018880797747	0.155	0.101	2.35945633239753e-49	no change
2926	2926	LMNB2	1.11221875843305e-53	0.406127514422348	0.097	0.056	2.42953065592116e-49	no change
2927	2927	ZNF622	1.11433854375669e-53	0.121148302103392	0.183	0.122	2.43416111498212e-49	no change
2928	2928	C14orf142	1.17951311041074e-53	0.256158704548709	0.128	0.08	2.57652843838122e-49	no change
2929	2929	TAF11	1.19352557307862e-53	0.113372769337013	0.279	0.201	2.60713726183294e-49	no change
2930	2930	RCE1	1.19549258094561e-53	0.194467613097413	0.156	0.101	2.61143399381759e-49	no change
2931	2931	PAAF1	1.24996631762806e-53	0.25694776587067	0.117	0.071	2.73042642422674e-49	no change
2932	2932	SLFN13	1.31955537476029e-53	0.986891959113534	0.035	0.012	2.88243676062638e-49	no change
2933	2933	TFB1M	1.39170044979874e-53	0.357552038726559	0.096	0.055	3.04003046254037e-49	no change
2934	2934	NDC80	1.43271538438605e-53	1.11187247423305	0.032	0.011	3.12962348565288e-49	up

2935	2935	GPATCH2L	1.4838386944878e-53	0.109383482992608	0.175	0.115	3.24129724423916e-49	no change
2936	2936	TMEM80	1.49094333078771e-53	0.174537622127609	0.115	0.068	3.25681661177268e-49	no change
2937	2937	KRT19	1.58418436990927e-53	-0.20839684	0.993	0.965	3.46049233762982e-49	no change
2938	2938	UBN2	1.6886869716197e-53	0.173277325915032	0.124	0.076	3.68876782080607e-49	no change
2939	2939	UBE3C	1.99716420933637e-53	0.217957717787517	0.142	0.091	4.36260549887437e-49	no change
2940	2940	NDUFA12	2.19615798937403e-53	0.102119030989769	0.681	0.538	4.79728751198864e-49	no change
2941	2941	KCNJ15	2.42109512223598e-53	0.120714639903767	0.295	0.213	5.28864018501227e-49	no change
2942	2942	SNX18	2.4621264004634e-53	0.199306140811549	0.1	0.058	5.37826890917226e-49	no change
2943	2943	REEP4	2.69230876434817e-53	0.143380138463952	0.258	0.184	5.88107926484214e-49	no change
2944	2944	SPOPL	2.70815532042252e-53	0.32383577229304	0.136	0.087	5.91569448193096e-49	no change
2945	2945	CEP350	2.7426499933494e-53	0.13042608862153	0.251	0.178	5.99104464547244e-49	no change
2946	2946	CDK11A	2.776561259038e-53	0.229311758177166	0.133	0.083	6.06512041424261e-49	no change
2947	2947	SLC38A10	2.86901507236331e-53	0.122383921024844	0.191	0.129	6.26707652407041e-49	no change

2948	2948	PPA1	3.18793687889703e-53	0.116140300089806	0.735	0.595	6.96372931826267e-49	no change
2949	2949	CEP104	3.20902625577676e-53	0.29114439689856	0.127	0.079	7.00979695311875e-49	no change
2950	2950	STAMBP	3.40482374129359e-53	0.134203970846653	0.267	0.192	7.43749698048172e-49	no change
2951	2951	PGAP1	3.43336514009287e-53	0.195625333999624	0.151	0.098	7.49984281201888e-49	no change
2952	2952	SMYD2	3.43645921599785e-53	0.145130304364419	0.194	0.131	7.50660151142571e-49	no change
2953	2953	GTF2H2	3.48793533287463e-53	0.25676635692861	0.112	0.067	7.61904594113133e-49	no change
2954	2954	CDK13	3.57406067472539e-53	0.193271821645751	0.125	0.077	7.80717813787015e-49	no change
2955	2955	LTN1	3.72403659330613e-53	0.144107986402519	0.13	0.081	8.13478553441792e-49	no change
2956	2956	FAF1	3.76484453444032e-53	0.148245345390813	0.18	0.121	8.22392640103144e-49	no change
2957	2957	MRPL12	3.88225158427532e-53	0.191676148885412	0.557	0.443	8.48039036069101e-49	no change
2958	2958	CRTAC1	3.9470305082559e-53	0.168795848944334	0.467	0.385	8.62189344223419e-49	no change
2959	2959	RP9	4.35505760640875e-53	0.290850363451138	0.127	0.079	9.51318783543926e-49	no change
2960	2960	MGA	4.40980724367003e-53	0.259731368341113	0.097	0.056	9.63278294307281e-49	no change

2961	2961	GEMIN4	4.75482389255061e-53	0.780770157097157	0.041	0.016	1.03864373108876e-48	no change
2962	2962	YARS	5.07865532751344e-53	0.258790293095787	0.141	0.09	1.10938146974204e-48	no change
2963	2963	CENPE	5.13522133278603e-53	1.22814147335176	0.028	0.008	1.12173774793378e-48	up
2964	2964	RP11-352M15.2	6.15707011770265e-53	0.302639462548453	0.136	0.086	1.34495039651097e-48	no change
2965	2965	PYM1	6.16221375842032e-53	0.153558792463015	0.331	0.246	1.34607397338933e-48	no change
2966	2966	SYMPK	6.17673359333467e-53	0.127893642879068	0.145	0.092	1.34924568612803e-48	no change
2967	2967	POF1B	7.53319406097043e-53	0.330604294045618	0.142	0.091	1.64555091067838e-48	no change
2968	2968	NEDD1	7.54377316592147e-53	0.247881439785678	0.098	0.056	1.64786181036389e-48	no change
2969	2969	FBLN5	7.95825244764393e-53	-1.62316459	0.034	0.065	1.73840066466334e-48	down
2970	2970	CASP7	8.67034079798814e-53	0.22174675090031	0.132	0.083	1.89394924391253e-48	no change
2971	2971	L3MBTL3	8.79256673189367e-53	0.517552446261551	0.062	0.03	1.92064827691485e-48	no change
2972	2972	MCRS1	9.07766916305637e-53	0.145980057259487	0.24	0.17	1.98292605197803e-48	no change
2973	2973	RIIAD1	9.39345671837185e-53	-1.352868586	0.024	0.052	2.05190668556115e-48	down

2974	2974	XPA	9.48854560384588e-53	0.128924393968452	0.157	0.102	2.07267790170409e-48	no change
2975	2975	ACVR1C	9.6030991970478e-53	0.815250847507216	0.036	0.013	2.09770098860312e-48	no change
2976	2976	APEX2	1.04954998760461e-52	0.211800010502477	0.133	0.084	2.29263699292352e-48	no change
2977	2977	TUBGCP2	1.29703965826418e-52	0.135162591286979	0.176	0.117	2.83325342951227e-48	no change
2978	2978	PIGN	1.3122146157567e-52	0.285305686738035	0.094	0.053	2.86640160665893e-48	no change
2979	2979	RP11-660L16.2	1.37343650368493e-52	0.483187705047702	0.103	0.061	3.00013469864936e-48	no change
2980	2980	POLR2E	1.38492231259567e-52	0.119282769085596	0.537	0.415	3.02522429963398e-48	no change
2981	2981	SART3	1.38827600558204e-52	0.360845547537218	0.085	0.047	3.0325501065934e-48	no change
2982	2982	PJA1	1.53161620798879e-52	0.196984402247659	0.117	0.071	3.34566244473072e-48	no change
2983	2983	MED14	1.69132119355573e-52	0.18472579619713	0.11	0.066	3.69452201520313e-48	no change
2984	2984	TAPBP	1.7110206454922e-52	0.101115195650767	0.504	0.386	3.73755349801315e-48	no change
2985	2985	UTP4	1.72424510283856e-52	0.187855391101389	0.125	0.077	3.76644100264056e-48	no change
2986	2986	DDX39B	1.89616581334718e-52	0.36582934574782	0.089	0.05	4.14198460267559e-48	no change

2987	2987	MRPL39	1.95204586489237e-52	0.189752139697444	0.137	0.086	4.2640489872709e-48	no change
2988	2988	ZNF680	1.96748130187863e-52	0.233915007812922	0.143	0.092	4.29776615582368e-48	no change
2989	2989	SPG11	2.29728830329752e-52	0.191213757569029	0.133	0.084	5.01819656972311e-48	no change
2990	2990	SGTA	2.32435488667465e-52	0.157529673799055	0.178	0.119	5.07732081445211e-48	no change
2991	2991	PRSS16	2.34539726475933e-52	0.293849739620331	0.123	0.076	5.12328578514029e-48	no change
2992	2992	LSG1	2.37549248104866e-52	0.315233634338642	0.1	0.058	5.1890257756027e-48	no change
2993	2993	RPS6KB1	2.62126076865062e-52	0.211387206613901	0.126	0.078	5.72588202304041e-48	no change
2994	2994	GPR107	2.96890309387175e-52	0.435660817879046	0.066	0.033	6.48527191825344e-48	no change
2995	2995	INTS8	3.15342340974943e-52	0.293916331295663	0.071	0.037	6.88833809625666e-48	no change
2996	2996	CAMSAP2	3.2529882867922e-52	0.133410437742789	0.133	0.083	7.10582761366888e-48	no change
2997	2997	PPARGC1B	3.29114939260865e-52	0.534301954770935	0.051	0.023	7.18918673321434e-48	no change
2998	2998	CTDSPL	3.49400472705614e-52	0.186497954706094	0.136	0.086	7.63230392578143e-48	no change
2999	2999	BDH1	3.63459403681335e-52	0.281231069800485	0.115	0.07	7.93940721401509e-48	no change

3000	3000	KIF3B	4.05144102135648e-52	0.345871165547823	0.097	0.056	8.84996776705109e-48	no change
3001	3001	RPP38	4.30966039739874e-52	0.122111133658128	0.217	0.152	9.41402217207781e-48	no change
3002	3002	WDR77	4.57472841861763e-52	0.132517622495474	0.24	0.17	9.99303675762835e-48	no change
3003	3003	USP39	4.71083802792665e-52	0.296665460076224	0.12	0.074	1.0290354588203e-47	no change
3004	3004	AGO1	4.7726605003723e-52	0.249853339779066	0.075	0.039	1.04253995970132e-47	no change
3005	3005	DERA	4.78960104760965e-52	0.119224464211421	0.18	0.12	1.04624045283985e-47	no change
3006	3006	MEN1	4.8300666561781e-52	0.343985855891511	0.1	0.058	1.05507976037554e-47	no change
3007	3007	PRKRIR	5.49237889004709e-52	0.160277187520685	0.124	0.077	1.19975524474189e-47	no change
3008	3008	TUBG1	5.57618891991076e-52	0.22481725316928	0.241	0.172	1.21806270766531e-47	no change
3009	3009	KDM4A	6.00040286474374e-52	0.334169086916363	0.104	0.061	1.31072800177462e-47	no change
3010	3010	CEP95	6.08383460004014e-52	0.235044186785579	0.126	0.079	1.32895283003277e-47	no change
3011	3011	NKTR	6.41367622189237e-52	0.102732897361363	0.363	0.271	1.40100343391017e-47	no change
3012	3012	AP3M2	6.79662127440521e-52	0.225392919112869	0.12	0.074	1.48465395118107e-47	no change

3013	3013	SAE1	7.08057512597804e-52	0.192066966120182	0.211	0.147	1.54668083051864e-47	no change
3014	3014	PHC3	7.24266057508271e-52	0.279275374082804	0.12	0.074	1.58208677602107e-47	no change
3015	3015	IGSF8	7.47073064978528e-52	0.126846634450131	0.189	0.128	1.6319064031391e-47	no change
3016	3016	GFM2	7.5577346361373e-52	0.353049608355586	0.081	0.044	1.65091155391783e-47	no change
3017	3017	SEC61G	7.70784171853151e-52	-0.171776293	0.949	0.915	1.68370094499602e-47	no change
3018	3018	TRAPPC12	7.8791047415248e-52	0.226365722322339	0.112	0.068	1.72111163973868e-47	no change
3019	3019	MCCC2	9.65944643232914e-52	0.177231878516515	0.263	0.19	2.11000947867798e-47	no change
3020	3020	DUS1L	9.8730596510387e-52	0.177249160211713	0.249	0.178	2.15667115017289e-47	no change
3021	3021	PHYKPL	1.02143710331066e-51	0.152586451620847	0.153	0.099	2.23122720847181e-47	no change
3022	3022	GRB2	1.06757197264454e-51	0.112875171107798	0.285	0.206	2.33200421704473e-47	no change
3023	3023	SIGLEC14	1.07290474524189e-51	-2.172648553	0.005	0.023	2.34365312550639e-47	down
3024	3024	ZFP36L1	1.12257643200936e-51	-0.299712471	0.848	0.828	2.45215595808126e-47	no change
3025	3025	NUDT15	1.15222242005304e-51	0.175059721124796	0.199	0.137	2.51691465436387e-47	no change

3026	3026	DRG2	1.20125807286229e-51	0.245966574563457	0.133	0.084	2.62402813436038e-47	no change
3027	3027	NUP214	1.21350760813829e-51	0.280760698618069	0.077	0.041	2.65078601921728e-47	no change
3028	3028	CREBZF	1.299851248426e-51	0.221763823275964	0.145	0.093	2.83939506706175e-47	no change
3029	3029	ANKIB1	1.33057635024084e-51	0.153857961260129	0.152	0.099	2.90651097946609e-47	no change
3030	3030	NCAPH	1.45061380113958e-51	1.91837728424298	0.019	0.003	3.16872078720929e-47	up
3031	3031	TSGA10IP	1.46022112558036e-51	-1.482876648	0.02	0.047	3.18970702671773e-47	down
3032	3032	CHD8	1.50279383930453e-51	0.272378673320016	0.116	0.071	3.28270286257681e-47	no change
3033	3033	BCAS3	1.57947324616202e-51	0.343752899487801	0.097	0.056	3.45020135891631e-47	no change
3034	3034	WNK2	1.58590722872932e-51	0.805428640702327	0.041	0.017	3.46425575043633e-47	no change
3035	3035	RAD51	1.70099936800523e-51	1.17796043462614	0.032	0.011	3.71566301947063e-47	up
3036	3036	MZT1	1.77121181367109e-51	0.138549085229567	0.306	0.227	3.86903508578313e-47	no change
3037	3037	TRAK1	1.869038342986e-51	0.150108319780718	0.336	0.251	4.08272735641862e-47	no change
3038	3038	ATAD5	1.87263192106771e-51	0.819323757911466	0.035	0.013	4.0905771683803e-47	no change

3039	3039	CBR4	1.92559242164057e-51	0.109698864413134	0.235	0.165	4.20626408583166e-47	no change
3040	3040	ICK	1.98484983683057e-51	0.305983213726877	0.093	0.054	4.33570598357269e-47	no change
3041	3041	MGME1	2.03365194950091e-51	0.183938904093743	0.143	0.091	4.44230931848979e-47	no change
3042	3042	TSEN54	2.04569419419178e-51	0.237168202594384	0.147	0.095	4.46861439779252e-47	no change
3043	3043	PRKAA2	2.10928166188168e-51	0.796440422235312	0.036	0.013	4.60751486221434e-47	no change
3044	3044	SUZ12	2.11582179122518e-51	0.14393762744556	0.213	0.149	4.62180112075228e-47	no change
3045	3045	ERH	2.22352458605294e-51	0.12852942644366	0.86	0.737	4.85706710577405e-47	no change
3046	3046	RUFY1	2.3116667419763e-51	0.248102949951841	0.095	0.055	5.04960483117303e-47	no change
3047	3047	GADD45A	2.42951091621279e-51	-0.528713934	0.62	0.592	5.30702364537523e-47	no change
3048	3048	RBMXL1	2.66991653994988e-51	0.115393462542674	0.187	0.127	5.83216568986651e-47	no change
3049	3049	GOLGA3	2.71314003938866e-51	0.219146580993579	0.13	0.082	5.92658310204059e-47	no change
3050	3050	ZNF280D	2.79227246880639e-51	0.138667494584884	0.146	0.094	6.09943998086068e-47	no change
3051	3051	RENBP	3.21134184355839e-51	-1.330202647	0.033	0.064	7.01485512306894e-47	down

3052	3052	TBCD	3.26030235196799e-51	0.293529616145282	0.123	0.076	7.12180445763888e-47	no change
3053	3053	AUH	3.3665850391967e-51	0.369631315063359	0.085	0.047	7.35396835962128e-47	no change
3054	3054	RBBP8	3.48715172000673e-51	0.261570490048558	0.111	0.067	7.61733421718269e-47	no change
3055	3055	MR1	3.60158365979587e-51	0.12037220868518	0.156	0.102	7.86729934645809e-47	no change
3056	3056	WDR11	3.69264329454471e-51	0.268331286051974	0.121	0.075	8.06621001260346e-47	no change
3057	3057	PSMD3	3.71269724418943e-51	0.139931774537659	0.345	0.257	8.1100158602074e-47	no change
3058	3058	SRC	3.71739029262555e-51	0.12930238512542	0.191	0.13	8.12026735521126e-47	no change
3059	3059	ZC3H18	3.74141627531164e-51	0.254403407862044	0.105	0.062	8.17274971179075e-47	no change
3060	3060	FAM73A	3.83851610704229e-51	0.182772767011781	0.132	0.083	8.38485458422317e-47	no change
3061	3061	SNAP25	4.11485890098902e-51	1.2041882103587	0.025	0.007	8.98849778332043e-47	up
3062	3062	XPOT	4.2486864614029e-51	0.25170055637069	0.136	0.087	9.2808307062885e-47	no change
3063	3063	PRAP1	4.27099905107427e-51	-1.356612373	0.055	0.092	9.32957032716664e-47	down
3064	3064	NSL1	4.2836076903871e-51	0.107985813687965	0.196	0.134	9.35711263888158e-47	no change

3065	3065	TOR2A	4.40378645521051e-51	0.398499333629224	0.101	0.06	9.61963113276185e-47	no change
3066	3066	HAUS7	4.67243907190343e-51	0.623514642618054	0.043	0.018	1.02064759086658e-46	no change
3067	3067	ADGRA3	4.77741833544279e-51	0.549058891594897	0.053	0.025	1.04357926119412e-46	no change
3068	3068	ATG10	4.7852629182949e-51	0.258815864882463	0.099	0.058	1.04529283187234e-46	no change
3069	3069	DLAT	5.10547273566264e-51	0.290347321787901	0.097	0.057	1.11523946437815e-46	no change
3070	3070	RAD1	5.23666434785466e-51	0.161983692498332	0.193	0.133	1.14389696014537e-46	no change
3071	3071	XG	5.5686920142051e-51	-1.22759823	0.044	0.079	1.21642508358296e-46	down
3072	3072	UTP6	5.62748056006111e-51	0.186885973255541	0.123	0.076	1.22926685353975e-46	no change
3073	3073	KRI1	5.70968411168257e-51	0.316463921496416	0.083	0.046	1.24722339735594e-46	no change
3074	3074	CDK5RAP2	5.72170943435261e-51	0.390056521731371	0.058	0.027	1.24985020883999e-46	no change
3075	3075	KIF13A	5.73922891739226e-51	0.155937263408022	0.133	0.084	1.25367716471516e-46	no change
3076	3076	DNMT3A	5.88416774039229e-51	0.181715391998408	0.099	0.058	1.28533760121129e-46	no change
3077	3077	DSN1	5.96297973201404e-51	0.408738013558853	0.074	0.039	1.30255329266115e-46	no change

3078	3078	B9D2	6.08626684551312e-51	0.198555542585943	0.124	0.077	1.32948412973389e-46	no change
3079	3079	ABCA10	6.20204374462823e-51	0.588400249148475	0.078	0.042	1.35477443557659e-46	no change
3080	3080	NARS2	6.40471338946538e-51	0.293264190177634	0.103	0.061	1.39904559279482e-46	no change
3081	3081	AKAP8	7.19458211104087e-51	0.200522411157431	0.119	0.073	1.57158451633577e-46	no change
3082	3082	ZFP91	7.22162755580414e-51	0.103361774017152	0.214	0.149	1.57749232328986e-46	no change
3083	3083	NUP133	7.44478483582051e-51	0.322785454377856	0.091	0.052	1.62623879953663e-46	no change
3084	3084	DENND4C	7.99228302914307e-51	0.226942427221771	0.12	0.074	1.74583430488601e-46	no change
3085	3085	TAP2	8.56549450488593e-51	0.23057809762251	0.11	0.066	1.87104661964728e-46	no change
3086	3086	EXOC3	9.21681022428336e-51	0.247372095712881	0.109	0.066	2.01332002539246e-46	no change
3087	3087	KIAA0430	9.42962899396205e-51	0.260760310696706	0.106	0.064	2.05980815744107e-46	no change
3088	3088	LBR	9.62783352603732e-51	0.105063325928765	0.166	0.111	2.10310395542759e-46	no change
3089	3089	ERCC4	9.94934129154046e-51	0.318629714356188	0.081	0.045	2.1733341117241e-46	no change
3090	3090	WDFY3	9.99357523318818e-51	0.233794320167483	0.099	0.058	2.18299657393763e-46	no change

3091	3091	FBXL17	1.05486771643564e-50	0.126012340343495	0.127	0.079	2.30425303978201e-46	no change
3092	3092	CARM1	1.07952148455262e-50	0.286370879186563	0.097	0.057	2.35810673085674e-46	no change
3093	3093	CLN6	1.30979459856314e-50	0.248159265647291	0.127	0.08	2.86111532110131e-46	no change
3094	3094	DDX10	1.38832766634221e-50	0.242310189202175	0.099	0.058	3.03266295435792e-46	no change
3095	3095	NF2	1.46774384277485e-50	0.32464209866761	0.085	0.048	3.20613965015739e-46	no change
3096	3096	PARN	1.49863785429405e-50	0.223488585026901	0.109	0.066	3.27362452891992e-46	no change
3097	3097	TMEM203	1.58841276233743e-50	0.201027743924113	0.143	0.093	3.46972883804989e-46	no change
3098	3098	CYP11A1	1.64238685458592e-50	-3.030517798	0.006	0.023	3.58762984515748e-46	down
3099	3099	SLAMF7	1.68832271820833e-50	-1.603168257	0.013	0.036	3.68797214565427e-46	down
3100	3100	ZBED1	1.73947938254665e-50	0.439610516536764	0.092	0.053	3.79971876323491e-46	no change
3101	3101	CTSK	1.79150541684167e-50	-1.529083828	0.046	0.079	3.91336443254894e-46	down
3102	3102	WDR73	1.82085672101112e-50	0.211200067146408	0.12	0.074	3.97747942137669e-46	no change
3103	3103	C5orf63	2.10757725498571e-50	0.218269084018138	0.116	0.071	4.60379175579078e-46	no change

3104	3104	TIPARP-AS1	2.12349607738174e-50	0.438228908413471	0.069	0.036	4.63856483143267e-46	no change
3105	3105	DTX3L	2.14842178216857e-50	0.24478228304382	0.115	0.07	4.69301254096902e-46	no change
3106	3106	DEAF1	2.17821440586093e-50	0.140372882082335	0.11	0.066	4.75809154816262e-46	no change
3107	3107	KMT5B	2.19401481329393e-50	0.112186531386849	0.193	0.132	4.79260595815926e-46	no change
3108	3108	GNL3L	2.2459816256395e-50	0.508718398561451	0.057	0.028	4.90612226304693e-46	no change
3109	3109	IQCA1	2.31333066924687e-50	1.07467074506121	0.027	0.008	5.05323951390286e-46	up
3110	3110	IKBKAP	2.41782211459047e-50	0.619241228646083	0.046	0.02	5.28149062711142e-46	no change
3111	3111	ZNF652	2.47399881126964e-50	0.12485853650418	0.153	0.1	5.40420300333741e-46	no change
3112	3112	MEA1	2.50629649054978e-50	0.103018247466209	0.584	0.45	5.47475405395695e-46	no change
3113	3113	MAN2A1	2.55389821337166e-50	0.10576595327372	0.148	0.096	5.57873525728905e-46	no change
3114	3114	MUT	2.62624630134208e-50	0.181208640560128	0.111	0.067	5.73677242065163e-46	no change
3115	3115	ICE2	2.65073131102966e-50	0.173785765700477	0.134	0.085	5.79025747581319e-46	no change
3116	3116	CFAP20	2.79532768092218e-50	0.100243843293406	0.236	0.167	6.10611378620641e-46	no change

3117	3117	VAPB	2.85440079733843e-50	0.15079563605793	0.17	0.114	6.23515310170607e-46	no change
3118	3118	XPO6	2.85954751020196e-50	0.342783914243949	0.095	0.056	6.24639558128515e-46	no change
3119	3119	RP11-800A18.4	2.99972929016941e-50	0.877498409690677	0.038	0.015	6.55260866144607e-46	no change
3120	3120	C16orf89	3.01747051096923e-50	-1.856151358	0.019	0.044	6.5913625841612e-46	down
3121	3121	DTX4	3.2466790073184e-50	0.327850417105683	0.096	0.056	7.09204562358631e-46	no change
3122	3122	VPS45	3.25046722384072e-50	0.265955487599153	0.102	0.06	7.10032060375767e-46	no change
3123	3123	ST6GALNAC4	3.43442233828324e-50	0.320833149741564	0.066	0.033	7.50215215574591e-46	no change
3124	3124	TMC7	3.67996739922926e-50	0.169993495385884	0.11	0.066	8.03852078687639e-46	no change
3125	3125	C3	4.01186169977695e-50	-1.220922056	0.124	0.167	8.76351069699277e-46	down
3126	3126	F12	4.18309823053639e-50	0.148204161131173	0.099	0.058	9.13755977478369e-46	no change
3127	3127	JUND	4.47739342695293e-50	-0.318282248	0.642	0.633	9.78041820183598e-46	no change
3128	3128	ARL14EP	4.79855060512864e-50	0.123954312753777	0.135	0.086	1.0481953941843e-45	no change
3129	3129	C1orf35	5.02238130418832e-50	0.106147238432365	0.345	0.258	1.0970889720869e-45	no change

3130	3130	EXOSC3	5.26238812462316e-50	0.206134735032001	0.142	0.091	1.14951606194268e-45	no change
3131	3131	C11orf57	5.60493566459389e-50	0.225960457750703	0.119	0.074	1.22434214657389e-45	no change
3132	3132	OMA1	5.74660436921416e-50	0.157198244667482	0.125	0.078	1.25528825841114e-45	no change
3133	3133	ASB8	5.81013129914125e-50	0.236978729008445	0.137	0.088	1.26916508098441e-45	no change
3134	3134	RASSF6	5.83721200998683e-50	0.115357949924177	0.18	0.122	1.27508059146152e-45	no change
3135	3135	SFXN4	5.86622306979577e-50	0.168893619819698	0.166	0.111	1.28141776736619e-45	no change
3136	3136	GDAP2	5.94463785324865e-50	0.316309001395927	0.113	0.069	1.29854669266364e-45	no change
3137	3137	ZNF33A	6.54177102974564e-50	0.120608362426121	0.202	0.139	1.42898446373764e-45	no change
3138	3138	ZNF621	6.55084725271232e-50	0.499595725035065	0.06	0.03	1.43096707388248e-45	no change
3139	3139	UBE2Z	6.5608283116026e-50	0.115599190974038	0.206	0.143	1.43314733638647e-45	no change
3140	3140	HMBS	7.27603686768831e-50	0.274660920476068	0.136	0.087	1.58937749337784e-45	no change
3141	3141	PAFAH1B3	7.78588857574697e-50	0.162635540267776	0.503	0.399	1.70074950048617e-45	no change
3142	3142	FAM169A	7.93564130890555e-50	0.662321743968996	0.042	0.017	1.73346148751733e-45	no change

3143	3143	PEX14	8.90712843936749e-50	0.279827148806643	0.123	0.077	1.94567313629543e-45	no change
3144	3144	PLA2G2F	9.33885884068035e-50	-0.584203936	0.251	0.3	2.03998032515821e-45	no change
3145	3145	LINC01426	9.4595157422944e-50	0.964576068350574	0.036	0.014	2.06633661874679e-45	no change
3146	3146	CYP4F35P	9.59637430211098e-50	0.588579158671958	0.045	0.019	2.09623200255312e-45	no change
3147	3147	KIAA2013	1.09987982646901e-49	0.178398088891939	0.176	0.119	2.4025774929389e-45	no change
3148	3148	ZNF800	1.13594064112114e-49	0.141277273179132	0.162	0.107	2.48134873646502e-45	no change
3149	3149	TRIM32	1.15296467919965e-49	0.479407585812504	0.057	0.027	2.51853604524372e-45	no change
3150	3150	MDFI	1.16281115757636e-49	0.229641032781351	0.2	0.14	2.5400446926098e-45	no change
3151	3151	NOP2	1.16763284747373e-49	0.269816993057449	0.093	0.054	2.55057719202163e-45	no change
3152	3152	CCDC66	1.17693874249586e-49	0.105627375490603	0.155	0.102	2.57090498910796e-45	no change
3153	3153	APOE	1.21772060804505e-49	-1.328515984	0.186	0.233	2.65998889621362e-45	down
3154	3154	KIF20A	1.31545308943629e-49	1.71164101773644	0.017	0.003	2.87347572856462e-45	up
3155	3155	BRCA2	1.32234414428807e-49	0.892674236734373	0.028	0.009	2.88852854878287e-45	no change

3156	3156	WIZ	1.32834695521602e-49	0.314450106729868	0.101	0.06	2.90164108897387e-45	no change
3157	3157	CHCHD6	1.45901212942467e-49	0.189741144515448	0.159	0.105	3.18706609551525e-45	no change
3158	3158	UBL7	1.48293476513186e-49	0.11912747993049	0.224	0.158	3.23932270095403e-45	no change
3159	3159	TXNRD2	1.48966967475907e-49	0.328163315792987	0.098	0.057	3.25403443754371e-45	no change
3160	3160	MED13L	1.50063610150837e-49	0.11301580172285	0.149	0.097	3.27798950013489e-45	no change
3161	3161	METTL8	1.62450108434976e-49	0.13823530532584	0.112	0.068	3.54856016865362e-45	no change
3162	3162	TBC1D9B	1.67983895650787e-49	0.180573656783438	0.118	0.073	3.6694402165958e-45	no change
3163	3163	POLR3H	1.68531206206869e-49	0.157453249998191	0.164	0.109	3.68139566838284e-45	no change
3164	3164	DNAJC19	1.96390086381174e-49	0.110480862677177	0.569	0.44	4.28994504691036e-45	no change
3165	3165	DROSHA	2.09514233375818e-49	0.467409212698555	0.072	0.038	4.57662891386136e-45	no change
3166	3166	PCBD1	2.11976952629241e-49	0.119351186753561	0.776	0.636	4.63042455323315e-45	no change
3167	3167	SLC33A1	2.59681193842764e-49	0.223067695504154	0.116	0.072	5.67247599830133e-45	no change
3168	3168	RP11-14N7.2	2.6842002418801e-49	0.114259615384002	0.245	0.177	5.8633670083629e-45	no change

3169	3169	VHL	2.72360517068334e-49	0.175989514772443	0.131	0.083	5.94944313484069e-45	no change
3170	3170	TMA7	2.77285100927958e-49	-0.122624615	0.99	0.969	6.05701574467032e-45	no change
3171	3171	OTUD5	2.82694030808257e-49	0.177146006194124	0.137	0.088	6.17516840897556e-45	no change
3172	3172	SCARA5	2.86598370343698e-49	-2.087757708	0.017	0.041	6.26045480178773e-45	down
3173	3173	RAC2	3.54547412247874e-49	0.422271204261633	0.114	0.071	7.74473367314255e-45	no change
3174	3174	NUDCD1	3.55948917163946e-49	0.312430093667025	0.084	0.047	7.77534814652923e-45	no change
3175	3175	MRPL51	3.74781175675794e-49	0.141039884675782	0.81	0.681	8.18672000146204e-45	no change
3176	3176	TIMM23	3.99946910319685e-49	0.131483879405144	0.192	0.132	8.73644030902319e-45	no change
3177	3177	TCEANC2	4.13340748831701e-49	0.652212335577982	0.048	0.022	9.02901531747968e-45	no change
3178	3178	CHAF1B	4.23477346618067e-49	1.0273239460711	0.03	0.01	9.25043915952505e-45	up
3179	3179	DYRK4	4.33914334489822e-49	0.218615928832718	0.155	0.103	9.47842472259566e-45	no change
3180	3180	CHERP	4.38730952226065e-49	0.207543284613574	0.141	0.092	9.58363892042617e-45	no change
3181	3181	SETD4	4.3875572001461e-49	0.270403820601328	0.096	0.056	9.58417994799915e-45	no change

3182	3182	ZNF883	4.44832568160707e-49	0.486425400039454	0.085	0.049	9.71692261890248e-45	no change
3183	3183	PIK3CB	4.46318517643639e-49	0.377398570111529	0.078	0.043	9.74938169940766e-45	no change
3184	3184	NPAT	4.70583428371449e-49	0.34878775230173	0.074	0.04	1.02794244093459e-44	no change
3185	3185	SMARCAD1	4.87522550308301e-49	0.264294356914884	0.086	0.049	1.06494425889345e-44	no change
3186	3186	CREB3L4	4.95704964175025e-49	0.336436387816918	0.109	0.067	1.08281792374393e-44	no change
3187	3187	TBK1	5.04241072358115e-49	0.117855116547708	0.154	0.101	1.10146419845907e-44	no change
3188	3188	CSNK2A2	5.23860723751838e-49	0.198456608476052	0.123	0.077	1.14432136496351e-44	no change
3189	3189	GPT2	5.8012193805407e-49	0.555588347455464	0.076	0.042	1.26721836148531e-44	no change
3190	3190	RPGR	5.90240445976921e-49	0.530317884458878	0.056	0.027	1.28932123019199e-44	no change
3191	3191	DUSP4	5.97387352105175e-49	0.111483419832463	0.359	0.273	1.30493293193854e-44	no change
3192	3192	FAM234A	6.65352364509091e-49	0.134958844541178	0.186	0.128	1.45339570503366e-44	no change
3193	3193	HOOK1	6.68872780442097e-49	0.318212078494339	0.094	0.055	1.46108570159772e-44	no change
3194	3194	ENTPD4	6.71625172034115e-49	0.158078703925891	0.096	0.056	1.46709802579132e-44	no change

3195	3195	BRWD3	7.6175465752664e-49	0.315584474361571	0.078	0.043	1.66397687390119e-44	no change
3196	3196	MRPS7	7.64163828422192e-49	0.109725779241676	0.529	0.411	1.66923946680544e-44	no change
3197	3197	MRPL30	7.68695272635969e-49	0.204600403329321	0.133	0.085	1.67913795354601e-44	no change
3198	3198	GNA11	7.72415301583355e-49	0.246677962678319	0.094	0.055	1.68726398477868e-44	no change
3199	3199	MAML1	8.20247874626485e-49	0.283067326363002	0.093	0.054	1.79174945733409e-44	no change
3200	3200	CLEC2B	8.34777658574911e-49	-0.56616704	0.275	0.322	1.82348831739103e-44	no change
3201	3201	PSMD5	8.67788279073472e-49	0.19369072046409	0.083	0.047	1.89559671680809e-44	no change
3202	3202	EPHX2	9.27081216061951e-49	0.253428186803029	0.086	0.048	2.02511620836573e-44	no change
3203	3203	PDPK1	9.66405792022677e-49	0.129329457379357	0.14	0.09	2.11101681209434e-44	no change
3204	3204	SASS6	9.75502640617756e-49	0.517398171718686	0.051	0.024	2.13088796816543e-44	no change
3205	3205	ELMOD3	1.20033370756308e-48	0.322347709144863	0.105	0.063	2.62200895080079e-44	no change
3206	3206	RIC8A	1.2109522297758e-48	0.129385445333287	0.174	0.117	2.64520405072226e-44	no change
3207	3207	R3HCC1	1.22707997161604e-48	0.143764006754236	0.151	0.099	2.68043348999809e-44	no change

3208	3208	TRMT1	1.23504203538643e-48	0.116353209196104	0.128	0.081	2.69782582209811e-44	no change
3209	3209	SNHG25	1.33640087117885e-48	0.415414058697284	0.452	0.371	2.91923406300309e-44	no change
3210	3210	NMNAT1	1.39079433336844e-48	0.359758269346505	0.058	0.029	3.03805114181001e-44	no change
3211	3211	ATP13A2	1.39838466621699e-48	0.305702226258567	0.096	0.056	3.05463146488439e-44	no change
3212	3212	UCKL1	1.42425968799556e-48	0.143845814186379	0.143	0.092	3.11115286245751e-44	no change
3213	3213	FAM120C	1.48490112483777e-48	0.477478626628192	0.053	0.025	3.24361801709564e-44	no change
3214	3214	RAB28	1.62499727498917e-48	0.136542711512714	0.176	0.119	3.54964404748633e-44	no change
3215	3215	MAP9	1.62583292283278e-48	0.336897886237724	0.114	0.071	3.55146943663592e-44	no change
3216	3216	KMT2D	1.63065705645167e-48	0.34195991507858	0.071	0.038	3.56200727411303e-44	no change
3217	3217	CCL2	1.65332152000015e-48	-1.089467953	0.071	0.11	3.61151552828833e-44	down
3218	3218	CWC27	1.6534390667809e-48	0.12464222820721	0.183	0.124	3.6117722974762e-44	no change
3219	3219	ATN1	1.80642015711961e-48	0.141730402689909	0.165	0.111	3.94594419121208e-44	no change
3220	3220	DEPDC1B	1.86090312686876e-48	1.22770965764596	0.029	0.009	4.06495679033213e-44	up

3221	3221	LINC00869	1.99612484983874e-48	0.191782753208472	0.121	0.076	4.36033512198774e-44	no change
3222	3222	BCL7A	2.18253165373355e-48	0.21100506172367	0.13	0.083	4.76752214441557e-44	no change
3223	3223	ZNF260	2.32629286047041e-48	0.393009086629878	0.088	0.05	5.08155412441156e-44	no change
3224	3224	NIT1	2.3616120649787e-48	0.167143208773472	0.167	0.112	5.15870539473948e-44	no change
3225	3225	H2AFJ	2.38332308102057e-48	-0.324147618	0.851	0.776	5.20613093818133e-44	no change
3226	3226	SAMD1	2.45830335386523e-48	0.214508898938878	0.099	0.059	5.36991784618322e-44	no change
3227	3227	MRPS17	2.58972704253904e-48	0.147867710233288	0.144	0.093	5.65699975172227e-44	no change
3228	3228	SHMT1	2.60480113937036e-48	0.448344365507615	0.076	0.042	5.68992760884061e-44	no change
3229	3229	CXCL12	2.71127617087825e-48	-1.573436359	0.029	0.057	5.92251166766644e-44	down
3230	3230	SYPL1	2.7655909379285e-48	0.106210910610249	0.832	0.707	6.04115684481101e-44	no change
3231	3231	CHCHD3	2.80663173793672e-48	0.135170716469209	0.268	0.196	6.13080636834897e-44	no change
3232	3232	EMB	2.88958707326222e-48	0.381268501478822	0.081	0.045	6.31201400283399e-44	no change
3233	3233	NUP50	2.99391716007337e-48	0.10956026248747	0.196	0.136	6.53991264446426e-44	no change

3234	3234	RND1	3.17133774348576e-48	-1.442765919	0.053	0.088	6.92747016687029e-44	down
3235	3235	EZH2	3.18766259917051e-48	0.42820704726965	0.075	0.041	6.96313018162806e-44	no change
3236	3236	TCTN3	3.43865344748319e-48	0.113349917258752	0.139	0.09	7.51139459068228e-44	no change
3237	3237	TBC1D30	3.51920631565369e-48	0.547383428195907	0.061	0.031	7.68735427591392e-44	no change
3238	3238	C16orf87	3.57171043995394e-48	0.120043187552126	0.233	0.167	7.80204428503538e-44	no change
3239	3239	KIF13B	3.68839661702152e-48	0.221497387901053	0.088	0.05	8.05693357022182e-44	no change
3240	3240	ZNF141	3.73754046947204e-48	0.752784584998274	0.039	0.016	8.16428340151472e-44	no change
3241	3241	TBC1D7	3.92344231553867e-48	0.177999194724343	0.151	0.1	8.57036739406268e-44	no change
3242	3242	SGF29	4.04521059798546e-48	0.131240153505889	0.123	0.077	8.83635803023945e-44	no change
3243	3243	XRCC1	4.09239547176822e-48	0.196804695078327	0.092	0.053	8.9394286685305e-44	no change
3244	3244	HACL1	4.10032587303412e-48	0.359964910099043	0.072	0.039	8.95675183705573e-44	no change
3245	3245	DGKZ	4.3011810275315e-48	0.119925161639897	0.159	0.105	9.39549983653981e-44	no change
3246	3246	UBA5	4.60243369676329e-48	0.174665819017834	0.149	0.098	1.00535561672097e-43	no change

3247	3247	SLC48A1	4.61376728580377e-48	0.255476408842484	0.13	0.084	1.00783132591098e-43	no change
3248	3248	ABCF2	4.7695477796528e-48	0.190222660454914	0.134	0.086	1.04186001698736e-43	no change
3249	3249	RPRD1B	4.89361576583359e-48	0.199440025035503	0.139	0.091	1.06896142788869e-43	no change
3250	3250	USP46	4.93436151815619e-48	0.24183133205438	0.162	0.11	1.07786193002604e-43	no change
3251	3251	TRAF7	5.67273600578901e-48	0.109112552798851	0.201	0.14	1.23915245310455e-43	no change
3252	3252	SMIM8	6.12516450373455e-48	0.182097626443495	0.142	0.093	1.33798093419578e-43	no change
3253	3253	ZNF519	6.33156387187678e-48	0.999078200239383	0.028	0.009	1.38306681217276e-43	no change
3254	3254	TANC1	6.39938057059041e-48	0.237457579531096	0.102	0.061	1.39788069183977e-43	no change
3255	3255	NCAPD3	6.65338899340355e-48	0.541178950711193	0.055	0.027	1.45336629171907e-43	no change
3256	3256	ATAD2B	7.75587872312192e-48	0.309765548320413	0.085	0.049	1.69419414827875e-43	no change
3257	3257	IGF1	7.87461730248363e-48	-1.490392369	0.025	0.052	1.72013140355452e-43	down
3258	3258	FBXO34	8.13789975813542e-48	0.138977060952415	0.127	0.081	1.7776428231671e-43	no change
3259	3259	SUPV3L1	8.47472445139017e-48	0.351917759056417	0.071	0.038	1.85121880916167e-43	no change

3260	3260	PAPD7	8.76226555911543e-48	0.493187842730629	0.062	0.031	1.91402928873317e-43	no change
3261	3261	OTULIN	8.90741568115702e-48	0.279179911327189	0.111	0.069	1.94573588139194e-43	no change
3262	3262	ASPG	9.40687852259904e-48	-1.186060741	0.041	0.073	2.05483854447654e-43	down
3263	3263	AUTS2	9.88431264215319e-48	0.172163759773599	0.08	0.045	2.15912925355194e-43	no change
3264	3264	CEP131	9.95976958807306e-48	0.233122373565613	0.099	0.059	2.17561206881868e-43	no change
3265	3265	NUDT19	1.03616551559448e-47	0.128074071665374	0.128	0.082	2.26339995226457e-43	no change
3266	3266	VPS53	1.07424028943186e-47	0.240522565338488	0.105	0.064	2.34657048823494e-43	no change
3267	3267	CHMP4C	1.07543074697639e-47	0.1126582445186	0.228	0.162	2.34917092369523e-43	no change
3268	3268	ZBTB8A	1.11855987307988e-47	0.354383196804598	0.064	0.033	2.44338218675568e-43	no change
3269	3269	MON2	1.22540421938974e-47	0.18084118641688	0.117	0.073	2.67677297683494e-43	no change
3270	3270	CAPN7	1.22875048410973e-47	0.143577679289403	0.115	0.071	2.68408255748929e-43	no change
3271	3271	CCDC117	1.36255176717148e-47	0.21525324113962	0.111	0.069	2.97635808020938e-43	no change
3272	3272	IRF1	1.59262395457298e-47	-0.795793378	0.29	0.324	3.47892776636923e-43	no change

3273	3273	FDXR	1.59714829628065e-47	0.159794587712943	0.166	0.111	3.48881073839545e-43	no change
3274	3274	NSUN5	1.61947033377578e-47	0.124893354532241	0.158	0.105	3.53757099709981e-43	no change
3275	3275	CENPC	1.96062927905576e-47	0.190143558905818	0.103	0.062	4.2827985971694e-43	no change
3276	3276	FAM92A1	1.97213143824256e-47	0.235387320482867	0.111	0.069	4.30792391369704e-43	no change
3277	3277	RP4-605O3.4	1.97961511435779e-47	0.299821901832969	0.096	0.057	4.32427125580316e-43	no change
3278	3278	WDR83	2.14672561775461e-47	0.153350455571103	0.126	0.08	4.68930743942317e-43	no change
3279	3279	XPO7	2.27524098436995e-47	0.281619589165998	0.083	0.047	4.97003640625771e-43	no change
3280	3280	EXT2	2.31171059380646e-47	0.130653496478801	0.097	0.057	5.04970062111083e-43	no change
3281	3281	POLRMT	2.35229916399112e-47	0.289573867157503	0.093	0.054	5.1383622938222e-43	no change
3282	3282	PIGA	2.35921718213324e-47	0.104633276284447	0.186	0.128	5.15347401265184e-43	no change
3283	3283	NPTXR	2.42065881040432e-47	0.741874863930808	0.038	0.015	5.28768710544721e-43	no change
3284	3284	CECR1	2.49861621220269e-47	0.393178269331495	0.097	0.058	5.45797725393555e-43	no change
3285	3285	KIF23	2.77533618655609e-47	0.859888017381143	0.032	0.011	6.06244436591312e-43	no change

3286	3286	LINC01550	2.77986297913152e-47	0.678268419983254	0.031	0.011	6.07233269161488e-43	no change
3287	3287	FAHD2A	2.78270352543245e-47	0.211033856533697	0.119	0.075	6.07853758095463e-43	no change
3288	3288	SPRYD4	2.87192096356275e-47	0.183061810890937	0.166	0.112	6.27342415280647e-43	no change
3289	3289	SH3YL1	3.05133207528246e-47	0.114183999540328	0.633	0.515	6.66532978524701e-43	no change
3290	3290	ENDOG	3.16716235649813e-47	0.211449536090393	0.11	0.068	6.91834945153452e-43	no change
3291	3291	FAM111A	3.2457321052072e-47	0.171808457556429	0.156	0.105	7.0899772106146e-43	no change
3292	3292	ASRGL1	3.25686093923513e-47	0.690884481852274	0.042	0.018	7.11428703566522e-43	no change
3293	3293	LDHA	3.40642177943013e-47	-0.294719351	0.734	0.688	7.44098773498717e-43	no change
3294	3294	RP11-258C19.7	3.69433328560563e-47	0.604225401911039	0.045	0.02	8.06990162907695e-43	no change
3295	3295	NACC1	3.82983047723918e-47	0.270877225184041	0.105	0.064	8.36588169448127e-43	no change
3296	3296	SPDL1	4.08032095991432e-47	0.601456657478172	0.041	0.017	8.91305310483684e-43	no change
3297	3297	PSMD9	4.28046117723377e-47	0.13106556832131	0.198	0.138	9.35023939554944e-43	no change
3298	3298	SDHAF1	4.29728714445413e-47	0.233664631638146	0.155	0.105	9.3869940383456e-43	no change

3299	3299	TPRG1L	4.49661642343457e-47	0.306040943197066	0.112	0.07	9.82240891535048e-43	no change
3300	3300	ZNF518B	4.53657931430032e-47	0.413816568483974	0.054	0.026	9.90970385415762e-43	no change
3301	3301	CYGB	4.68330526636522e-47	-1.542352405	0.043	0.075	1.02302120238482e-42	down
3302	3302	CLASP2	4.71875116726997e-47	0.229216571847069	0.092	0.054	1.03076400497845e-42	no change
3303	3303	KIAA0355	4.80138748612117e-47	0.178844761606213	0.117	0.073	1.04881508246831e-42	no change
3304	3304	ABI3BP	4.87272692366821e-47	-1.599345483	0.027	0.054	1.06439846920608e-42	down
3305	3305	PCYT1A	5.04481000104916e-47	0.181555414668485	0.133	0.086	1.10198829662918e-42	no change
3306	3306	ORC2	5.43333330121742e-47	0.228636316644959	0.097	0.058	1.18685732631793e-42	no change
3307	3307	DCAF12	5.45365286342467e-47	0.210281331750577	0.129	0.083	1.19129593148649e-42	no change
3308	3308	C17orf97	5.45688666810573e-47	0.522076723151518	0.049	0.022	1.19200232378102e-42	no change
3309	3309	NAA25	5.59989655346353e-47	0.195508908969908	0.148	0.098	1.22324140313857e-42	no change
3310	3310	NFS1	5.69410256577074e-47	0.196903836601729	0.119	0.075	1.24381976446696e-42	no change
3311	3311	C2orf49	6.00063057780281e-47	0.169705185454131	0.15	0.1	1.31077774341525e-42	no change

3312	3312	SPICE1	6.32020797529952e-47	0.307476252270332	0.088	0.051	1.38058623012443e-42	no change
3313	3313	AGAP3	6.32895013431303e-47	0.156960104908056	0.152	0.101	1.38249586733934e-42	no change
3314	3314	STRA13	6.40400920437403e-47	0.406745995216834	0.598	0.509	1.39889177060346e-42	no change
3315	3315	ABCC1	6.44820822189706e-47	0.465929204211622	0.064	0.033	1.40854660399119e-42	no change
3316	3316	CCDC88A	6.59868247436515e-47	0.278580413804744	0.077	0.043	1.44141619970032e-42	no change
3317	3317	RP11-670E13.6	7.90842415523314e-47	-0.827361074	0.192	0.234	1.72751617246913e-42	no change
3318	3318	LONRF2	8.0286776193692e-47	0.963867927652688	0.027	0.008	1.75378433917501e-42	no change
3319	3319	SPAG4	8.32491628436686e-47	-0.771555539	0.189	0.232	1.8184947131571e-42	no change
3320	3320	MT-ND5	8.406679118053e-47	0.136272965645643	0.976	0.955	1.8363549865475e-42	no change
3321	3321	RCCD1	8.55589522991089e-47	0.262019444688715	0.089	0.052	1.86894975402174e-42	no change
3322	3322	BUB1	8.59405999950605e-47	1.17299282393065	0.024	0.007	1.8772864662921e-42	up
3323	3323	HSP90AA1	9.18058393017815e-47	-0.278207026	0.988	0.97	2.00540675370811e-42	no change
3324	3324	RFC3	9.58950548477243e-47	0.52650132185699	0.051	0.024	2.09473157809369e-42	no change

3325	3325	PTPN14	9.89994463754229e-47	0.100733973881731	0.131	0.084	2.16254390662474e-42	no change
3326	3326	PIM1	1.07374664318542e-46	-0.646254791	0.466	0.466	2.34549216737422e-42	no change
3327	3327	RNF40	1.07412675604907e-46	0.177876739655555	0.136	0.088	2.3463224859136e-42	no change
3328	3328	H1FX	1.11454875115768e-46	0.152307953333982	0.352	0.271	2.43462029202885e-42	no change
3329	3329	LINC01006	1.11880586938078e-46	0.521968425584739	0.049	0.022	2.44391954107537e-42	no change
3330	3330	AKAP1	1.14153426138107e-46	0.150233568655004	0.167	0.113	2.4935674405608e-42	no change
3331	3331	TEX10	1.1611426212202e-46	0.394582919761358	0.051	0.024	2.53639994179341e-42	no change
3332	3332	GABPB2	1.18625987363264e-46	0.293847920993966	0.087	0.05	2.59126606796314e-42	no change
3333	3333	MSL2	1.26097588033714e-46	0.184354084804811	0.083	0.047	2.75447571300845e-42	no change
3334	3334	RNF41	1.27102320098136e-46	0.136572120052679	0.157	0.105	2.77642308022367e-42	no change
3335	3335	KIAA1147	1.34273543054509e-46	0.402088174796957	0.065	0.034	2.93307127448268e-42	no change
3336	3336	ZADH2	1.35678850126484e-46	0.205486198759796	0.107	0.065	2.96376880216291e-42	no change
3337	3337	MIA3	1.39444548757513e-46	0.119276695324448	0.133	0.086	3.04602672305912e-42	no change

3338	3338	DCUN1D4	1.47074200408236e-46	0.221870369461748	0.148	0.099	3.21268883371751e-42	no change
3339	3339	STX17	1.48767161917417e-46	0.236643641766241	0.114	0.072	3.24966988492406e-42	no change
3340	3340	NCAPD2	1.62319902784306e-46	0.732646824054114	0.042	0.018	3.54571595642039e-42	no change
3341	3341	SYK	1.65507855229349e-46	0.4020383426409	0.063	0.033	3.6153535896299e-42	no change
3342	3342	MYLPF	1.80951249854494e-46	0.261252942599652	0.093	0.055	3.95269910182158e-42	no change
3343	3343	DGKE	1.88382802339328e-46	0.394697963026851	0.071	0.039	4.11503393430028e-42	no change
3344	3344	TMEM177	1.89058614339394e-46	0.560994724459973	0.054	0.026	4.12979637162972e-42	no change
3345	3345	CTD-3157E16.2	1.90758743425703e-46	0.397804223693131	0.069	0.037	4.16693399139105e-42	no change
3346	3346	XBP1	1.97054715566094e-46	-0.431171992	0.624	0.59	4.30446320682576e-42	no change
3347	3347	RFWD3	2.28834345834586e-46	0.341033244799558	0.066	0.035	4.99865745041069e-42	no change
3348	3348	MTR	2.29510704978622e-46	0.226710890344692	0.086	0.049	5.01343183955303e-42	no change
3349	3349	COX7A1	2.33492095926369e-46	-1.681406964	0.027	0.054	5.10040134341559e-42	down
3350	3350	TMEM92	2.4631224060161e-46	0.172397541185434	0.163	0.11	5.38044458370156e-42	no change

3351	3351	TET3	2.48265422517252e-46	0.312071387913706	0.071	0.039	5.42310988946685e-42	no change
3352	3352	KLHDC10	2.70476200964311e-46	0.246436784608753	0.088	0.051	5.90828213386441e-42	no change
3353	3353	G2E3	2.80496792430979e-46	0.183692081153661	0.095	0.056	6.12717193386231e-42	no change
3354	3354	ZKSCAN8	2.9350234052311e-46	0.557873580998892	0.053	0.025	6.41126512638682e-42	no change
3355	3355	PDAP1	2.98469244496008e-46	0.131005315530014	0.58	0.459	6.51976217677079e-42	no change
3356	3356	POC5	3.05339477813705e-46	0.55763396288947	0.045	0.02	6.66983555336257e-42	no change
3357	3357	BCL11B	3.18741694826661e-46	0.134221809596962	0.063	0.033	6.96259358179358e-42	no change
3358	3358	CTD-2020K17.4	3.18913291632685e-46	0.133705490126165	0.218	0.155	6.96634194242438e-42	no change
3359	3359	POM121C	3.24406794446483e-46	0.207087950785572	0.114	0.071	7.08634201788898e-42	no change
3360	3360	MBTD1	3.31158740282024e-46	0.272286140300802	0.081	0.046	7.23383152272052e-42	no change
3361	3361	MUTYH	3.42085187922755e-46	0.249752972793149	0.081	0.046	7.47250884498467e-42	no change
3362	3362	MND1	3.47613032656414e-46	0.952862268168507	0.03	0.01	7.5932590853467e-42	no change
3363	3363	GNB5	3.64051705974724e-46	0.133088576024442	0.163	0.11	7.95234546531187e-42	no change

3364	3364	PCNXL3	3.65032923354514e-46	0.330846219366669	0.069	0.037	7.97377917775601e-42	no change
3365	3365	SLF1	3.82626762441398e-46	0.395064595102681	0.052	0.025	8.3580989987699e-42	no change
3366	3366	OGFOD1	4.04675853616003e-46	0.187581782574473	0.093	0.055	8.83973934638798e-42	no change
3367	3367	AHCTF1	4.10073878935075e-46	0.161866358503399	0.095	0.056	8.95765381145779e-42	no change
3368	3368	RAP1GAP2	4.76457134403439e-46	0.6976193560718	0.036	0.014	1.04077296439087e-41	no change
3369	3369	RAB15	4.9812611319042e-46	0.159793965021595	0.13	0.084	1.08810668165315e-41	no change
3370	3370	GATC	4.99542155471894e-46	0.248151865584884	0.103	0.063	1.09119988441281e-41	no change
3371	3371	FAM227A	5.01574451229901e-46	0.761913657993913	0.035	0.014	1.09563923126659e-41	no change
3372	3372	FBXW9	5.13056626713211e-46	0.393873259710541	0.069	0.037	1.12072089539234e-41	no change
3373	3373	FAAP24	5.25281385809007e-46	0.492880804995525	0.053	0.026	1.1474246591612e-41	no change
3374	3374	MED11	5.55707037926826e-46	0.102571216716232	0.178	0.123	1.21388645364736e-41	no change
3375	3375	BGN	5.68333644191892e-46	-1.142728956	0.029	0.057	1.24146801237277e-41	down
3376	3376	ZNF248	5.71175300503968e-46	0.351975418100249	0.068	0.036	1.24767532642087e-41	no change

3377	3377	ATRN	5.73840458804012e-46	0.26558228712976	0.08	0.045	1.25349709821148e-41	no change
3378	3378	KIAA0232	6.20893341912767e-46	0.231133003035203	0.113	0.071	1.35627941607425e-41	no change
3379	3379	METTL13	6.41853844133172e-46	0.283593376593797	0.083	0.047	1.4020655371245e-41	no change
3380	3380	ELAVL2	6.5289533949873e-46	1.11187099290589	0.024	0.007	1.42618457960103e-41	up
3381	3381	GABRB3	6.53052289620227e-46	0.525747025871413	0.035	0.014	1.42652742144642e-41	no change
3382	3382	HAUS5	6.53434308568549e-46	0.500796189395552	0.056	0.027	1.42736190363714e-41	no change
3383	3383	NOL9	7.01420660727384e-46	0.324374787416474	0.063	0.033	1.5321832912929e-41	no change
3384	3384	LSM3	7.12754692406388e-46	0.139507080455802	0.791	0.659	1.55694135009251e-41	no change
3385	3385	LIG3	7.5953564842169e-46	0.418838614847086	0.077	0.043	1.65912967041234e-41	no change
3386	3386	CPSF7	8.08044786947696e-46	0.143283310993208	0.134	0.087	1.76509303260855e-41	no change
3387	3387	UBQLN4	8.53325913128943e-46	0.206657044081589	0.12	0.077	1.86400512463886e-41	no change
3388	3388	NCOR2	8.60785979223017e-46	0.153678932438476	0.111	0.069	1.88030089301476e-41	no change
3389	3389	MXD3	8.7795047653498e-46	0.954565662314999	0.037	0.015	1.91779502094301e-41	no change

3390	3390	PMEL	9.24905426610522e-46	-1.378764034	0.029	0.056	2.02036341388802e-41	down
3391	3391	KREMEN1	9.78560220783013e-46	0.418352277520127	0.085	0.05	2.13756694627841e-41	no change
3392	3392	RNF8	1.00713361480807e-45	0.211965199919154	0.136	0.089	2.19998266818675e-41	no change
3393	3393	HAUS8	1.09653244585e-45	0.495595256571353	0.054	0.026	2.39526547471473e-41	no change
3394	3394	GCNT4	1.11754267430987e-45	0.251767634438069	0.118	0.075	2.44116021776248e-41	no change
3395	3395	CDKL5	1.11990148732702e-45	0.317334595292785	0.084	0.048	2.44631280891715e-41	no change
3396	3396	APC	1.19275968016191e-45	0.304213498445357	0.069	0.037	2.60546424534568e-41	no change
3397	3397	ELP4	1.21963314524435e-45	0.30920887312616	0.066	0.035	2.66416664247177e-41	no change
3398	3398	PRSS8	1.35303482441515e-45	-0.876677084	0.099	0.141	2.95556927045245e-41	no change
3399	3399	RP11-706O15.5	1.3703249438259e-45	1.30379148338468	0.023	0.006	2.99333780729329e-41	up
3400	3400	SLC12A8	1.38901462090452e-45	0.88571524190124	0.032	0.012	3.03416353790383e-41	no change
3401	3401	CCAR2	1.43010749367811e-45	0.246643602905361	0.091	0.053	3.12392680919046e-41	no change
3402	3402	MMGT1	1.43569340278196e-45	0.145877726456205	0.124	0.079	3.1361286690369e-41	no change

3403	3403	MFHAS1	1.50105523819591e-45	0.455704091456212	0.056	0.028	3.27890506231514e-41	no change
3404	3404	UGGT1	1.51525114532944e-45	0.279655489298535	0.085	0.049	3.30991460185762e-41	no change
3405	3405	NFIA	1.51600037345044e-45	-0.284408397	0.381	0.274	3.31155121576514e-41	no change
3406	3406	PITRM1	1.52079863127143e-45	0.297015262238137	0.1	0.061	3.32203253014931e-41	no change
3407	3407	ADIRF	1.54682320595933e-45	-0.407732586	0.977	0.955	3.37888061109756e-41	no change
3408	3408	SIPA1L2	1.5484033276011e-45	0.218988226581909	0.087	0.051	3.38233222881184e-41	no change
3409	3409	SRRM2	1.60076710862105e-45	0.113414478358983	0.626	0.503	3.49671567207181e-41	no change
3410	3410	CUL1	1.6020005838893e-45	0.16912316891374	0.138	0.091	3.49941007544778e-41	no change
3411	3411	TTC5	1.69797893838807e-45	0.213774996129908	0.084	0.048	3.70906519301491e-41	no change
3412	3412	SMAD6	1.70330934811241e-45	0.333622657935818	0.099	0.06	3.72070894001675e-41	no change
3413	3413	DUOX1	1.73760973993584e-45	0.140131753896746	0.117	0.074	3.79563471591586e-41	no change
3414	3414	ARHGEF1	1.83924992596235e-45	0.18774731270224	0.101	0.061	4.01765753827217e-41	no change
3415	3415	NOTCH3	1.92276287007941e-45	0.100090531302274	0.165	0.113	4.20008321340146e-41	no change

3416	3416	EXOC4	2.04555195415906e-45	0.11137891524363	0.157	0.105	4.46830368866505e-41	no change
3417	3417	ACADS	2.05354901312246e-45	0.167464497383596	0.134	0.087	4.48577246426469e-41	no change
3418	3418	DLL1	2.15011861613556e-45	0.48653311529747	0.042	0.018	4.69671910508652e-41	no change
3419	3419	SUGP2	2.16693437585588e-45	0.171286128010314	0.138	0.091	4.73345145061959e-41	no change
3420	3420	CYBRD1	2.18399772777697e-45	-1.472547499	0.087	0.125	4.77072463655601e-41	down
3421	3421	TRMT11	2.21635135925595e-45	0.145993440002935	0.108	0.067	4.84139790915869e-41	no change
3422	3422	UBE2E2	2.23134494275638e-45	0.165732974382575	0.083	0.047	4.87414989295704e-41	no change
3423	3423	RP11-1055B8.9	2.30824399404988e-45	0.559943440618204	0.067	0.036	5.04212818060256e-41	no change
3424	3424	LINC00667	2.46731210591268e-45	0.15483414370443	0.128	0.083	5.38959656415566e-41	no change
3425	3425	CKAP2L	2.46735963956468e-45	1.3143407740925	0.021	0.005	5.38970039666509e-41	up
3426	3426	UQCC1	2.48263061223175e-45	0.225783879466573	0.101	0.061	5.42305830935903e-41	no change
3427	3427	IPO8	2.49780416077681e-45	0.26104484328312	0.085	0.049	5.45620340880086e-41	no change
3428	3428	TTC33	2.50154543879581e-45	0.361581705540187	0.058	0.029	5.46437585650556e-41	no change

3429	3429	PRPF3	2.54608967558052e-45	0.205514050388353	0.097	0.058	5.5616782873381e-41	no change
3430	3430	GDA	2.68701377488384e-45	0.449510574327226	0.09	0.054	5.86951288985625e-41	no change
3431	3431	TSC22D1	2.83294593133229e-45	-0.487833151	0.643	0.628	6.18828709240226e-41	no change
3432	3432	KAT6A	2.90498206440399e-45	0.153255009158489	0.13	0.084	6.34564282148408e-41	no change
3433	3433	RHNO1	2.96476156939746e-45	0.221076844877659	0.16	0.109	6.47622517219182e-41	no change
3434	3434	NR1D2	3.04200596548935e-45	0.165655377189622	0.122	0.078	6.64495783101494e-41	no change
3435	3435	PKP2	3.16814091759843e-45	0.328793986570306	0.077	0.043	6.92048702040201e-41	no change
3436	3436	MAN1B1	3.27589297910607e-45	0.149169263806112	0.133	0.086	7.1558606235593e-41	no change
3437	3437	RASA1	3.57625681247088e-45	0.188540565791557	0.103	0.063	7.8119753811614e-41	no change
3438	3438	PIGG	3.58504837244138e-45	0.292578248021018	0.087	0.051	7.83117966476095e-41	no change
3439	3439	CDC14B	3.69200891676235e-45	0.251969546648803	0.081	0.046	8.06482427777568e-41	no change
3440	3440	AKR1C1	3.77495392816644e-45	0.1241206923392	0.542	0.45	8.24600936068677e-41	no change
3441	3441	CAMTA1	3.79941795619858e-45	0.113031753782189	0.692	0.561	8.29944858352018e-41	no change

3442	3442	GPKOW	3.81173618516206e-45	0.160664936307781	0.112	0.069	8.32635652286801e-41	no change
3443	3443	C5orf51	3.87641374297237e-45	0.192429921977682	0.139	0.092	8.46763818014885e-41	no change
3444	3444	SOWAHC	3.89160807654721e-45	0.12830298975863	0.427	0.334	8.50082868240973e-41	no change
3445	3445	NARF	4.09376211250494e-45	0.113476192193334	0.376	0.287	8.94241395855578e-41	no change
3446	3446	DDX55	4.28637151628423e-45	0.31867759102352	0.082	0.047	9.36314994017127e-41	no change
3447	3447	ZNF75A	4.66822615243805e-45	0.341885134919926	0.09	0.053	1.01972732073857e-40	no change
3448	3448	MTRR	4.8055580692327e-45	0.299052198531988	0.078	0.044	1.04972610464319e-40	no change
3449	3449	KIAA1524	5.05066642644389e-45	0.728967378164353	0.03	0.011	1.1032675741924e-40	no change
3450	3450	UHRF2	5.12146314878326e-45	0.394027258184427	0.063	0.033	1.11873241022022e-40	no change
3451	3451	LINC01410	5.24891463244545e-45	0.546310154780562	0.054	0.027	1.14657291231138e-40	no change
3452	3452	KANSL2	5.33501934464751e-45	0.277304861177956	0.092	0.055	1.1653816256448e-40	no change
3453	3453	EIF4ENIF1	5.49463077411504e-45	0.469595371586397	0.061	0.032	1.20024714629769e-40	no change
3454	3454	INPP5K	5.64324926990367e-45	0.144257365786841	0.114	0.072	1.23271137051776e-40	no change

3455	3455	KLF10	6.05716123774673e-45	-0.662903125	0.308	0.343	1.32312630077339e-40	no change
3456	3456	LRFN4	6.06546218904569e-45	0.851351782148116	0.03	0.011	1.32493956057514e-40	no change
3457	3457	KLHDC7B	6.11448204552942e-45	-1.310899327	0.027	0.053	1.33564745802545e-40	down
3458	3458	PVALB	6.35965858207725e-45	0.55010563531475	0.069	0.038	1.38920382066895e-40	no change
3459	3459	C12orf65	6.62309702948395e-45	0.167789432413826	0.126	0.081	1.44674931512047e-40	no change
3460	3460	GTF3C3	6.7924104383593e-45	0.189337893761661	0.095	0.057	1.48373413615521e-40	no change
3461	3461	ZBTB41	6.88506252564778e-45	0.327157164868671	0.078	0.044	1.5039730581025e-40	no change
3462	3462	SLC25A10	6.89804176909918e-45	0.296075845493683	0.123	0.08	1.50680824404203e-40	no change
3463	3463	SFRP1	7.00840511635355e-45	-1.189336139	0.032	0.06	1.53091601361627e-40	down
3464	3464	HIST1H4J	7.03365629302878e-45	0.618103079846613	0.061	0.032	1.53643188064921e-40	no change
3465	3465	PROC	7.28734937287376e-45	0.236148655232696	0.085	0.049	1.59184859701054e-40	no change
3466	3466	MEST	7.32913190046246e-45	0.145206676420365	0.187	0.13	1.60097557233702e-40	no change
3467	3467	WRN	7.35233465830793e-45	0.424801881640652	0.051	0.024	1.60604398276078e-40	no change

3468	3468	DLG3	7.43051417838355e-45	0.425925550110071	0.073	0.04	1.6231215171261e-40	no change
3469	3469	BPIFB1	7.59110881272247e-45	0.456000525792651	0.043	0.019	1.6582018090511e-40	no change
3470	3470	ALOX15B	7.64686731430602e-45	-1.375057323	0.027	0.054	1.67038169613701e-40	down
3471	3471	CLDN10	7.83923451461417e-45	2.02913858203994	0.016	0.003	1.71240238737232e-40	up
3472	3472	TNPO2	8.8881890498504e-45	0.168950751977555	0.1	0.061	1.94153601604932e-40	no change
3473	3473	DPH2	9.40596313919046e-45	0.38816710600831	0.078	0.044	2.05463858812476e-40	no change
3474	3474	ATAT1	9.87091014688261e-45	0.171333324382397	0.118	0.074	2.15620161248504e-40	no change
3475	3475	TOM1L1	1.07080993353059e-44	0.216332672521523	0.156	0.106	2.33907721880423e-40	no change
3476	3476	NIPSNAP3B	1.10480266575151e-44	1.1868597678456	0.02	0.005	2.4133309430676e-40	up
3477	3477	LEO1	1.13259065696123e-44	0.138896217138101	0.112	0.07	2.47403103106612e-40	no change
3478	3478	RAD51C	1.15374481775063e-44	0.175069128214364	0.199	0.141	2.52024017989448e-40	no change
3479	3479	MXRA8	1.33684693085121e-44	-1.626479855	0.025	0.051	2.92020843575137e-40	down
3480	3480	NCS1	1.40729590374596e-44	0.391663966892819	0.055	0.027	3.07409717214268e-40	no change

3481	3481	MGST3	1.50377601372155e-44	-0.318227751	0.725	0.672	3.28484832437334e-40	no change
3482	3482	VDAC1	1.54089764471114e-44	0.128402134996403	0.784	0.662	3.36593681510701e-40	no change
3483	3483	SPARCL1	1.55306906211031e-44	-1.519384278	0.052	0.084	3.39252405927377e-40	down
3484	3484	LINC00967	1.70577735554298e-44	-2.656324192	0.018	0.04	3.72610005544809e-40	down
3485	3485	NOC3L	1.80603716203393e-44	0.257451802060938	0.086	0.05	3.94510757674692e-40	no change
3486	3486	C1orf159	1.85186140129791e-44	0.308679928898314	0.089	0.053	4.04520604499515e-40	no change
3487	3487	PPAT	1.93307912078524e-44	0.325377869168519	0.059	0.03	4.22261803144327e-40	no change
3488	3488	MAD2L1BP	1.94944408326887e-44	0.1641309029182	0.126	0.081	4.25836565549252e-40	no change
3489	3489	SETD6	2.07794244568131e-44	0.511208920240228	0.057	0.029	4.53905747834624e-40	no change
3490	3490	GABBR1	2.19666120922704e-44	0.411737915274299	0.057	0.029	4.79838674543555e-40	no change
3491	3491	BCL11A	2.30955106666798e-44	0.155412372198743	0.079	0.044	5.04498335002953e-40	no change
3492	3492	GADD45B	2.41628374412652e-44	-0.693701343	0.566	0.566	5.27813021066998e-40	no change
3493	3493	RUSC1	2.48086817801399e-44	0.117076510371485	0.272	0.202	5.41920844805375e-40	no change

3494	3494	COLGALT1	2.55148634414352e-44	0.200404782078971	0.09	0.053	5.5734667701471e-40	no change
3495	3495	TMPRSS3	2.70881732255501e-44	-1.361707563	0.032	0.06	5.91714055938916e-40	down
3496	3496	IGSF3	2.7100872503078e-44	0.236101568642505	0.094	0.056	5.91991458957236e-40	no change
3497	3497	APOD	2.80926652898859e-44	-2.141657889	0.08	0.116	6.13656180592267e-40	down
3498	3498	ENSA	2.82660737183258e-44	0.117696562885636	0.767	0.629	6.1744411430311e-40	no change
3499	3499	PEX10	2.84024850855338e-44	0.170296993304972	0.184	0.129	6.20423884208401e-40	no change
3500	3500	TEAD1	2.89937441018542e-44	0.171391159290905	0.094	0.057	6.33339346160904e-40	no change
3501	3501	RAD54L	2.98505332035828e-44	2.01382314163247	0.017	0.003	6.52055047299063e-40	up
3502	3502	CFD	3.03207156761679e-44	-1.98406545	0.37	0.399	6.62325713230212e-40	down
3503	3503	PTRH2	3.05323972049269e-44	0.124789080653089	0.173	0.119	6.66949684544423e-40	no change
3504	3504	HLCS	3.28177408892973e-44	0.407255258613529	0.068	0.037	7.1687073198581e-40	no change
3505	3505	PACS1	3.4123704556308e-44	0.206875966417031	0.117	0.075	7.45398202327992e-40	no change
3506	3506	GAS6	3.50243642073097e-44	-1.294350307	0.065	0.1	7.65072211744473e-40	down

3507	3507	FARS2	3.56888116958325e-44	0.254134790944609	0.091	0.054	7.79586402683765e-40	no change
3508	3508	MPHOSPH9	3.61443258890586e-44	0.632078655263603	0.04	0.017	7.89536654720597e-40	no change
3509	3509	DDX11	3.8153200068056e-44	0.487344490034592	0.054	0.027	8.33418502286616e-40	no change
3510	3510	BCKDHB	3.8842004143237e-44	0.112681570208065	0.166	0.113	8.48464738504869e-40	no change
3511	3511	LY6G6C	3.89777736090724e-44	-1.76373393	0.017	0.04	8.51430486716577e-40	down
3512	3512	MAPKAPK5-AS1	4.04400645486441e-44	0.110327530174517	0.201	0.142	8.83372770000581e-40	no change
3513	3513	ICT1	4.08443547671679e-44	0.129014732	0.25	0.182	8.92204085534015e-40	no change
3514	3514	TAF1B	4.19968810917677e-44	0.217196541580154	0.086	0.05	9.17379870568574e-40	no change
3515	3515	IPO9	4.55723276339231e-44	0.254524524398162	0.102	0.063	9.95481924835417e-40	no change
3516	3516	PGAP2	4.55798923514567e-44	0.233075561305212	0.119	0.076	9.9564716852522e-40	no change
3517	3517	JARID2	4.64903785061874e-44	0.114835068734215	0.131	0.086	1.01553582808916e-39	no change
3518	3518	DPAGT1	4.78942093074597e-44	0.150566938595266	0.148	0.099	1.04620110811215e-39	no change
3519	3519	PALM	4.85032296407298e-44	0.249606712592756	0.039	0.016	1.0595045482721e-39	no change

3520	3520	TMEM161B	5.00322627065052e-44	0.120467847090242	0.081	0.046	1.0929047465609e-39	no change
3521	3521	HNRNPH1	5.0340113820381e-44	-0.192244177	0.683	0.548	1.0996294462924e-39	no change
3522	3522	CD34	5.27272471901029e-44	-2.290229031	0.012	0.032	1.15177398762061e-39	down
3523	3523	XCL1	5.51947576000015e-44	0.925844422178154	0.023	0.007	1.20567428501443e-39	no change
3524	3524	SLC25A46	5.75862901919973e-44	0.113846955892131	0.129	0.084	1.25791492295399e-39	no change
3525	3525	SPESP1	6.09939398660954e-44	1.01756646587594	0.026	0.008	1.33235162243499e-39	up
3526	3526	DLGAP5	6.11044241088595e-44	0.917743877637148	0.024	0.007	1.33476504023393e-39	no change
3527	3527	VSIG2	6.28061860546122e-44	-0.383444157	0.796	0.692	1.37193832817695e-39	no change
3528	3528	LINC01082	6.31782552416324e-44	-1.541708627	0.025	0.051	1.38006580749822e-39	down
3529	3529	METTL2B	6.32441620879777e-44	0.245739909750001	0.091	0.055	1.38150547664978e-39	no change
3530	3530	C19orf52	6.63331863536866e-44	0.154587108	0.13	0.085	1.44898212270993e-39	no change
3531	3531	ORAOV1	7.22945982054473e-44	-0.96116145	0.144	0.186	1.57920320319979e-39	no change
3532	3532	C1R	7.24729035317766e-44	-1.743961012	0.105	0.142	1.58309810474813e-39	down

3533	3533	RAI14	7.52115799754962e-44	0.211529539090759	0.196	0.14	1.64292175298474e-39	no change
3534	3534	SOS1	7.69473440439976e-44	0.126291689774476	0.146	0.098	1.68083778329708e-39	no change
3535	3535	PIGM	7.73525625389881e-44	0.184985181690533	0.105	0.065	1.68968937610166e-39	no change
3536	3536	FAM58A	7.88108080216542e-44	0.196911789877069	0.18	0.126	1.72154329042501e-39	no change
3537	3537	TCTEX1D2	8.63272685615214e-44	0.150139403700143	0.141	0.094	1.88573285445787e-39	no change
3538	3538	DIEXF	9.08977100469449e-44	0.293960320408924	0.072	0.04	1.98556957826546e-39	no change
3539	3539	CASC5	9.2951515302824e-44	0.938591836471254	0.026	0.009	2.03043290027489e-39	no change
3540	3540	MMP28	9.8900238083494e-44	-0.660807454	0.201	0.245	2.16037680069584e-39	no change
3541	3541	COX14	1.06228704154283e-43	0.209143913261976	0.788	0.665	2.32045981354616e-39	no change
3542	3542	POLA2	1.07216012200061e-43	0.708952442629752	0.037	0.016	2.34202657049814e-39	no change
3543	3543	ZBTB11	1.10996176546365e-43	0.211600745017582	0.096	0.058	2.4246004804788e-39	no change
3544	3544	PODN	1.11149520181453e-43	-1.862521163	0.02	0.043	2.42795011884367e-39	down
3545	3545	MPV17L2	1.1202614722979e-43	0.279205759368427	0.083	0.049	2.44709916008754e-39	no change

3546	3546	ZNF320	1.12490081800182e-43	0.313382860024458	0.111	0.07	2.45723334684318e-39	no change
3547	3547	TBL3	1.14265168238079e-43	0.115728695298714	0.105	0.065	2.4960083349926e-39	no change
3548	3548	N6AMT2	1.16867953902973e-43	0.10430886868855	0.132	0.086	2.55286358505655e-39	no change
3549	3549	IGFBP3	1.17399242121792e-43	-0.478644558	0.658	0.525	2.56446904490842e-39	no change
3550	3550	FBXO38	1.26999515855185e-43	0.233671999621098	0.069	0.037	2.77417742434067e-39	no change
3551	3551	MAPK9	1.27573245221462e-43	0.291628939925344	0.098	0.06	2.78670996861762e-39	no change
3552	3552	ZMYM1	1.3204602066514e-43	0.446041449732384	0.051	0.025	2.88441327540932e-39	no change
3553	3553	XRCC4	1.32745244959975e-43	0.245169516619332	0.082	0.047	2.8996871309057e-39	no change
3554	3554	RAB3GAP1	1.34207735542934e-43	0.104571898038742	0.19	0.134	2.93163377519986e-39	no change
3555	3555	SLC2A8	1.40284251905656e-43	0.190484272611205	0.103	0.064	3.06436919862715e-39	no change
3556	3556	SLC22A5	1.50712112147415e-43	0.139217133754935	0.091	0.054	3.29215537774813e-39	no change
3557	3557	ZNF268	1.64302244664267e-43	0.453937187857578	0.054	0.027	3.58901823244626e-39	no change
3558	3558	ZNF10	1.88278769467778e-43	0.469305301528935	0.054	0.027	4.11276144025414e-39	no change

3559	3559	LPCAT1	1.95761705729276e-43	0.33527009422235	0.088	0.053	4.27621869995031e-39	no change
3560	3560	PHF23	1.97699881668577e-43	0.110653022070314	0.157	0.107	4.3185562151684e-39	no change
3561	3561	COX6A1	2.07040772773678e-43	0.13218998331083	0.978	0.938	4.52259864046821e-39	no change
3562	3562	PRKCZ	2.20875070792897e-43	0.184537116888649	0.155	0.106	4.82479504640004e-39	no change
3563	3563	FAM122B	2.27931217390252e-43	0.105332951324619	0.15	0.101	4.97892951267267e-39	no change
3564	3564	TAF8	2.37202886040575e-43	0.201384708322125	0.112	0.071	5.18145984267032e-39	no change
3565	3565	MIRLET7BHG	2.39685279807951e-43	0.196031550089611	0.114	0.073	5.23568525212489e-39	no change
3566	3566	THOC5	2.76405342307409e-43	0.332059758555345	0.079	0.045	6.03779829736304e-39	no change
3567	3567	UTP20	2.8978679877314e-43	0.309934607079029	0.055	0.028	6.33010283240047e-39	no change
3568	3568	ATE1	2.94630209907762e-43	0.215014120575626	0.126	0.082	6.43590230522515e-39	no change
3569	3569	ERCC6L2	3.07265872942965e-43	0.463313714110627	0.049	0.023	6.71191572856613e-39	no change
3570	3570	TARS2	3.08109631073202e-43	0.30169518654069	0.08	0.046	6.73034678116303e-39	no change
3571	3571	CTDSPL2	3.08421136992764e-43	0.124675061703511	0.113	0.072	6.73715131646994e-39	no change

3572	3572	NDUFV3	3.16725336067627e-43	0.12093216416693	0.416	0.325	6.91854824106123e-39	no change
3573	3573	RP11-736K20.5	3.18783491554105e-43	-1.277453143	0.03	0.057	6.96350658950787e-39	down
3574	3574	SLC41A3	3.2977644654953e-43	0.170322197307844	0.15	0.101	7.20363669842794e-39	no change
3575	3575	SNRPE	3.32429437076095e-43	0.100544140740194	0.908	0.806	7.26158862349021e-39	no change
3576	3576	PSRC1	3.47559111998636e-43	0.919499880882733	0.028	0.01	7.59208124249821e-39	no change
3577	3577	UBALD1	3.55163848308218e-43	0.100068259273782	0.175	0.121	7.75819910244471e-39	no change
3578	3578	ZNF609	3.6280868027221e-43	0.198233404614764	0.097	0.059	7.92519281186616e-39	no change
3579	3579	DIO1	3.9497973881682e-43	-1.909092842	0.008	0.026	8.62793741471462e-39	down
3580	3580	SCMH1	4.40280608397723e-43	0.21518019272003	0.128	0.084	9.61748960983986e-39	no change
3581	3581	RFT1	4.42958815513462e-43	0.242662765640683	0.094	0.057	9.67599236607606e-39	no change
3582	3582	SPATA7	4.52661057466555e-43	0.460022886129657	0.068	0.037	9.88792813929942e-39	no change
3583	3583	FBXO45	4.71330020737664e-43	0.504075532629875	0.066	0.036	1.02957329729935e-38	no change
3584	3584	MIR4458HG	4.87585791723185e-43	0.181758059448726	0.374	0.293	1.06508240344013e-38	no change

3585	3585	ERI2	4.88438976124869e-43	0.290786024286534	0.077	0.044	1.06694609944716e-38	no change
3586	3586	ZMAT5	5.00500632782448e-43	0.182488870587951	0.176	0.123	1.09329358224998e-38	no change
3587	3587	WDR27	5.07194917461687e-43	0.47373178802853	0.051	0.025	1.10791657770331e-38	no change
3588	3588	FBRSL1	5.07471886674456e-43	0.350240216436056	0.075	0.043	1.10852158925168e-38	no change
3589	3589	TOR3A	5.26816698580826e-43	0.143218234326029	0.196	0.139	1.15077839637996e-38	no change
3590	3590	UBE2I	5.65434979468487e-43	0.11875584438289	0.716	0.581	1.23513616915096e-38	no change
3591	3591	LINC01088	5.87702841153852e-43	-1.85950379	0.03	0.057	1.28377808621647e-38	down
3592	3592	TARBP2	6.00848244896954e-43	0.173570204572824	0.159	0.109	1.31249290615291e-38	no change
3593	3593	GULP1	6.48029145594346e-43	-1.439433695	0.038	0.067	1.41555486563629e-38	down
3594	3594	TFF1	6.61490176121837e-43	0.543689499458517	0.196	0.145	1.44495914072054e-38	no change
3595	3595	HERC1	6.78420132335371e-43	0.226410129820906	0.09	0.054	1.48194093707338e-38	no change
3596	3596	NEK2	6.80381867563128e-43	0.844195722815001	0.028	0.01	1.4862261515049e-38	no change
3597	3597	CXXC4	6.87600161435402e-43	0.745609045925877	0.025	0.008	1.50199379263949e-38	no change

3598	3598	MRPL20	6.96564100851124e-43	0.127555443252444	0.785	0.652	1.52157462189919e-38	no change
3599	3599	UBL4A	7.03135878205277e-43	0.209367363595232	0.119	0.077	1.53593001235161e-38	no change
3600	3600	DHRS4-AS1	7.20469274258525e-43	0.16604243430724	0.153	0.105	1.57379308269032e-38	no change
3601	3601	GIT1	7.40857844383965e-43	0.332148330380694	0.077	0.044	1.61832987527233e-38	no change
3602	3602	LRRC40	8.12602282987315e-43	0.202753708391934	0.073	0.041	1.77504842695749e-38	no change
3603	3603	TSGA10	8.22089463799116e-43	0.301071593998028	0.09	0.054	1.79577222472279e-38	no change
3604	3604	LA16c-431H6.6	8.56155523328393e-43	0.411087967941668	0.052	0.026	1.87018612515854e-38	no change
3605	3605	ERRFI1	8.86019116837642e-43	-0.447218397	0.602	0.588	1.93542015882014e-38	no change
3606	3606	ITGBL1	1.02493450866988e-42	-1.951290292	0.016	0.037	2.23886694073848e-38	down
3607	3607	VAV2	1.02966812781425e-42	0.520339204856054	0.043	0.019	2.24920705839745e-38	no change
3608	3608	ENTPD1	1.03742706178379e-42	-1.18437178	0.027	0.053	2.26615567376051e-38	down
3609	3609	VPS8	1.06477754147519e-42	0.219757767455271	0.109	0.069	2.3259000615984e-38	no change
3610	3610	ECI1	1.07647789912606e-42	0.134228438261053	0.231	0.169	2.35145832285097e-38	no change

3611	3611	NOC4L	1.17243461547486e-42	0.119963574775952	0.135	0.089	2.56106617404329e-38	no change
3612	3612	DPY19L1	1.24776576130266e-42	0.425218424688967	0.055	0.028	2.72561952898953e-38	no change
3613	3613	PTBP2	1.32691425071033e-42	0.245712036077898	0.061	0.032	2.89851148925164e-38	no change
3614	3614	NBPF1	1.35983563360621e-42	0.456128432428822	0.074	0.042	2.97042495804941e-38	no change
3615	3615	UIMC1	1.36777217437302e-42	0.225818667137567	0.073	0.041	2.98776153770042e-38	no change
3616	3616	HIST1H3H	1.4584835662014e-42	0.70472688089756	0.055	0.028	3.18591150201035e-38	no change
3617	3617	GLMN	1.48799780981502e-42	0.290540153793622	0.082	0.048	3.25038241575994e-38	no change
3618	3618	SIRT6	1.50331861737109e-42	0.197620281126099	0.127	0.083	3.2838491877854e-38	no change
3619	3619	SHQ1	1.61164491586736e-42	0.342637118946413	0.065	0.035	3.52047715422066e-38	no change
3620	3620	ZNF559	1.65484773537591e-42	0.239008530600132	0.088	0.052	3.61484939315514e-38	no change
3621	3621	INTS2	1.66282724001366e-42	0.437404755346224	0.055	0.028	3.63227982308583e-38	no change
3622	3622	C3orf17	1.73864065124953e-42	0.11649968118771	0.111	0.07	3.79788663858947e-38	no change
3623	3623	CHTF18	1.77221945117281e-42	0.546356566191176	0.04	0.017	3.87123616914189e-38	no change

3624	3624	PARD6G	1.7924611278913e-42	0.242884444627351	0.111	0.071	3.91545208776576e-38	no change
3625	3625	VWA9	1.80956303824754e-42	0.151312479479021	0.136	0.09	3.95280950074792e-38	no change
3626	3626	POC1A	1.85404455745668e-42	0.770355396255812	0.037	0.016	4.04997493130837e-38	no change
3627	3627	ADRBK1	1.88577006278534e-42	0.205611910673024	0.112	0.071	4.11927612514829e-38	no change
3628	3628	GATAD2A	2.11440382896339e-42	0.120558240289744	0.142	0.095	4.61870372398762e-38	no change
3629	3629	ETAA1	2.20704785540083e-42	0.327193626412324	0.06	0.031	4.82107533533758e-38	no change
3630	3630	MYO9A	2.23417907865819e-42	0.16744656827118	0.093	0.056	4.88034077942096e-38	no change
3631	3631	CHM	2.30627923250326e-42	0.100801343831051	0.113	0.071	5.03783635548012e-38	no change
3632	3632	GRAMD3	2.31342169816092e-42	0.106652185704412	0.172	0.119	5.05343835746272e-38	no change
3633	3633	SRPX	2.39747509093879e-42	-1.429463428	0.033	0.06	5.23704458864669e-38	down
3634	3634	KIF3A	2.43215771655065e-42	0.227691789650647	0.087	0.052	5.31280531603325e-38	no change
3635	3635	DVL3	2.49060168249292e-42	0.123555098606152	0.165	0.115	5.44047031523753e-38	no change
3636	3636	APOO	2.55564548246991e-42	0.114339650075454	0.133	0.087	5.58255199190727e-38	no change

3637	3637	JRKL	2.58230596438008e-42	0.27604564364783	0.077	0.044	5.64078914859185e-38	no change
3638	3638	SLC25A1	2.71844038369174e-42	0.151222957999407	0.34	0.262	5.93816117413624e-38	no change
3639	3639	CPSF2	3.0142446891636e-42	0.157007714411294	0.127	0.083	6.58431609900896e-38	no change
3640	3640	XRCC2	3.16886298775772e-42	1.44915259743057	0.018	0.004	6.92206431045795e-38	up
3641	3641	HTRA2	3.31748386946902e-42	0.102736002939391	0.145	0.098	7.24671176446812e-38	no change
3642	3642	RP11-262H14.3	3.7714822685949e-42	0.20305753990988	0.089	0.053	8.23842586751871e-38	no change
3643	3643	RCAN1	3.83171129372339e-42	-1.083370066	0.119	0.158	8.36999015000936e-38	down
3644	3644	TP53I11	3.88663490179181e-42	0.263454313851465	0.075	0.043	8.48996527947403e-38	no change
3645	3645	ESCO1	3.8931854390018e-42	0.248326044708502	0.108	0.069	8.50427427295553e-38	no change
3646	3646	PCOLCE	3.97385886397427e-42	-1.596174232	0.08	0.115	8.6804973024654e-38	down
3647	3647	ARL14	4.17538193211512e-42	-1.077049764	0.239	0.275	9.12070429251226e-38	down
3648	3648	SRGAP2C	4.45142037797464e-42	0.158800346141043	0.102	0.064	9.72368267364781e-38	no change
3649	3649	EGLN3	4.45798734930687e-42	-0.803052709	0.228	0.266	9.73802756582593e-38	no change

3650	3650	CIT	4.51914124442932e-42	1.33019017049889	0.018	0.004	9.8716121343314e-38	up
3651	3651	GTF3C2	4.53063470233216e-42	0.232453947266095	0.104	0.066	9.89671844377438e-38	no change
3652	3652	CXorf40B	4.59221446414249e-42	0.205376731216163	0.105	0.067	1.00312332754729e-37	no change
3653	3653	PTPDC1	4.66372517926056e-42	0.558496370515048	0.039	0.017	1.01874412815768e-37	no change
3654	3654	LANCL1	4.7069717554519e-42	0.22529080554985	0.1	0.062	1.02819091026091e-37	no change
3655	3655	SUMO2	4.72582762092067e-42	0.108981479971675	0.981	0.934	1.03230978551391e-37	no change
3656	3656	MARK2	4.87455355986102e-42	0.154148548186116	0.129	0.085	1.06479747961604e-37	no change
3657	3657	TCF12	4.90902909430402e-42	0.100354046935563	0.126	0.083	1.07232831535977e-37	no change
3658	3658	ALDH5A1	5.11199964573357e-42	0.177043707800945	0.14	0.095	1.11666520261404e-37	no change
3659	3659	TBC1D4	5.30118349156043e-42	0.116668719866678	0.102	0.063	1.15799052189646e-37	no change
3660	3660	MKL2	5.33599778474973e-42	0.153345903469905	0.097	0.06	1.16559535610073e-37	no change
3661	3661	PHF2	5.34865631154317e-42	0.373823882307632	0.061	0.033	1.16836048469349e-37	no change
3662	3662	SMAD4	5.42044728128229e-42	0.127882240079456	0.12	0.077	1.1840425041233e-37	no change

3663	3663	NOTCH1	5.66044701897852e-42	0.324496279376813	0.069	0.039	1.23646804682567e-37	no change
3664	3664	DCAF10	6.06827305810073e-42	0.119103416910251	0.129	0.085	1.32555356681152e-37	no change
3665	3665	INTS3	6.26030515160182e-42	0.156744903692735	0.128	0.084	1.3675010573159e-37	no change
3666	3666	S100PBP	6.65670085760578e-42	0.161084477330993	0.099	0.061	1.45408973533541e-37	no change
3667	3667	HIST1H2BD	6.99483868411362e-42	0.313737535099916	0.13	0.087	1.52795256215778e-37	no change
3668	3668	DMKN	7.15242195553904e-42	-0.620659587	0.546	0.536	1.56237505196795e-37	no change
3669	3669	GSTM4	7.3557052320274e-42	-0.556560328	0.373	0.396	1.60678025088407e-37	no change
3670	3670	PNPLA4	7.37642302252806e-42	0.119716385407942	0.164	0.113	1.61130584504103e-37	no change
3671	3671	ADAM28	7.4177408754287e-42	0.219783488966567	0.057	0.029	1.62033131682865e-37	no change
3672	3672	HIRIP3	7.96155025087666e-42	0.213081696920708	0.086	0.051	1.7391210368015e-37	no change
3673	3673	ESYT1	8.17667279927276e-42	0.10077962269256	0.159	0.109	1.78611240627314e-37	no change
3674	3674	SECISBP2L	8.23808957888981e-42	0.106423979536209	0.12	0.077	1.79952828761269e-37	no change
3675	3675	PSMA4	8.30322741163744e-42	0.106720337685592	0.821	0.708	1.81375699579808e-37	no change

3676	3676	FANCA	8.40287875529327e-42	0.482309692770888	0.044	0.02	1.83552483530626e-37	no change
3677	3677	PPP5C	8.57664606261609e-42	0.215871194688668	0.107	0.068	1.87348256591786e-37	no change
3678	3678	TAX1BP1	8.98330915906242e-42	0.1377056014091	0.75	0.621	1.9623140527056e-37	no change
3679	3679	CTB-50L17.10	9.46809198187755e-42	0.212101304025885	0.112	0.072	2.06821001252133e-37	no change
3680	3680	TSPAN4	9.66864817658461e-42	-1.470533009	0.05	0.081	2.11201950769314e-37	down
3681	3681	TIMM13	1.12209440532776e-41	0.163233243316062	0.809	0.695	2.45110301899795e-37	no change
3682	3682	SPPL2B	1.18398519011783e-41	0.270977758441095	0.086	0.052	2.58629724929338e-37	no change
3683	3683	XPO5	1.21113477476715e-41	0.562637809814333	0.058	0.03	2.64560280200136e-37	no change
3684	3684	GALNT14	1.27754467478934e-41	0.502655462863634	0.096	0.06	2.79066858760984e-37	no change
3685	3685	CHMP7	1.29233560825067e-41	0.186270662149048	0.099	0.061	2.82297790266277e-37	no change
3686	3686	ARHGAP11A	1.33465280778241e-41	0.943733450162132	0.027	0.01	2.91541559331989e-37	no change
3687	3687	MYSM1	1.43563566242253e-41	0.201959289500335	0.107	0.067	3.13600254099577e-37	no change
3688	3688	PPARA	1.49424373997803e-41	0.294659329605619	0.069	0.039	3.26402602560802e-37	no change

3689	3689	HLA-DRB5	1.50051634584585e-41	-0.698083162	0.284	0.327	3.27772790586569e-37	no change
3690	3690	FBXL20	1.51224172709034e-41	0.187972932844148	0.092	0.056	3.30334082865614e-37	no change
3691	3691	THBD	1.53182582540782e-41	-0.780778949	0.173	0.215	3.34612033302085e-37	no change
3692	3692	KIF18A	1.5954663840067e-41	1.39064518136117	0.018	0.004	3.48513676922424e-37	up
3693	3693	C6orf223	1.62839968430311e-41	-1.416292247	0.015	0.036	3.5570762703917e-37	down
3694	3694	CSNK1G1	1.73578463811602e-41	0.167299066759185	0.092	0.056	3.79164796350063e-37	no change
3695	3695	GHDC	1.78781860782132e-41	0.213293555672105	0.094	0.058	3.90531096692489e-37	no change
3696	3696	NFIC	1.85222642041349e-41	-0.141887612	0.205	0.144	4.04600339275122e-37	no change
3697	3697	PUS7	1.92333888049433e-41	0.513960941020179	0.049	0.024	4.20134145055181e-37	no change
3698	3698	KIAA1217	2.08611815090674e-41	-0.103964075	0.216	0.153	4.55691648884067e-37	no change
3699	3699	ZNF738	2.18317129624876e-41	0.417751302803532	0.066	0.036	4.76891937952579e-37	no change
3700	3700	SKA3	2.21361654770653e-41	1.01792966511608	0.023	0.007	4.83542398681014e-37	up
3701	3701	ZC3H7A	2.26126013074499e-41	0.143061625391852	0.095	0.058	4.93949662959935e-37	no change

3702	3702	GGA2	2.315795299268e-41	0.132844840761207	0.135	0.09	5.05862325172102e-37	no change
3703	3703	CELSR1	2.35990911213388e-41	0.464604605643618	0.047	0.022	5.15498546454526e-37	no change
3704	3704	MKS1	2.36929162245178e-41	0.336725443378936	0.067	0.037	5.17548062008366e-37	no change
3705	3705	RP11-465N4.4	2.40200022696309e-41	-1.232874008	0.062	0.096	5.24692929577818e-37	down
3706	3706	RUNDC1	2.45090754592471e-41	0.12868795337176	0.126	0.083	5.35376244331793e-37	no change
3707	3707	IL1RN	2.47893715977658e-41	-0.634035527	0.338	0.367	5.41499033181596e-37	no change
3708	3708	SKA1	2.48574516857971e-41	1.35034001199725	0.02	0.005	5.42986174624551e-37	up
3709	3709	CCDC6	2.5456960904054e-41	0.11431183583341	0.16	0.111	5.56081853988155e-37	no change
3710	3710	CNKSR3	2.55437400384519e-41	0.240701851224329	0.065	0.036	5.57977457399943e-37	no change
3711	3711	ZNF814	2.56761141612315e-41	0.509963699967836	0.045	0.021	5.60869037737941e-37	no change
3712	3712	LMO3	2.67556430108603e-41	0.332227646030144	0.045	0.021	5.84450265929232e-37	no change
3713	3713	RBMS3	2.68147006555588e-41	-0.155278227	0.056	0.029	5.85740321120027e-37	no change
3714	3714	TARSL2	2.81685103450059e-41	0.118665155491798	0.09	0.054	6.15312939976309e-37	no change

3715	3715	RAD17	2.85821329064276e-41	0.268733065727661	0.087	0.052	6.24348111208005e-37	no change
3716	3716	TMEM176A	3.00425404677155e-41	-1.419759177	0.042	0.071	6.56249253976776e-37	down
3717	3717	ABHD10	3.05214661468146e-41	0.233883192366884	0.081	0.048	6.66710906511017e-37	no change
3718	3718	CARF	3.06242980964294e-41	0.467121124	0.051	0.025	6.68957167618403e-37	no change
3719	3719	LONP1	3.10807730522081e-41	0.227827043320775	0.114	0.074	6.78928406552434e-37	no change
3720	3720	KRTAP5-8	3.29983414776717e-41	0.601394741355053	0.047	0.023	7.2081577123826e-37	no change
3721	3721	RAD18	3.52716729984932e-41	0.661316049515661	0.038	0.016	7.70474424979086e-37	no change
3722	3722	RP11-490M8.1	3.66847520486155e-41	0.353256838592522	0.1	0.063	8.01341723749956e-37	no change
3723	3723	GTF2H2C	3.78695022503762e-41	0.365956277234016	0.051	0.025	8.27221407157217e-37	no change
3724	3724	CD3EAP	3.81116099807762e-41	0.228786170070164	0.078	0.045	8.32510008420075e-37	no change
3725	3725	EXOC6B	3.9283691444765e-41	0.252420749176649	0.095	0.059	8.58112955919447e-37	no change
3726	3726	COIL	4.10548456724558e-41	0.104075294451563	0.191	0.136	8.96802048869125e-37	no change
3727	3727	C2orf69	4.32260226521545e-41	0.136085511521927	0.132	0.088	9.44229238813663e-37	no change

3728	3728	ZCCHC8	4.48841980356887e-41	0.130673434404789	0.112	0.072	9.80450421891584e-37	no change
3729	3729	LNX1	4.86557991343767e-41	0.217402765123476	0.081	0.048	1.06283727629133e-36	no change
3730	3730	BMP7	5.32383654861944e-41	0.186492045289205	0.142	0.096	1.16293885568043e-36	no change
3731	3731	MAL	5.39799876571018e-41	-1.603396793	0.06	0.093	1.17913885038173e-36	down
3732	3732	TMEM205	5.52799661530719e-41	-0.37941132	0.644	0.595	1.2075355806477e-36	no change
3733	3733	PCNT	5.55933454140355e-41	0.359048819210453	0.054	0.027	1.21438103722419e-36	no change
3734	3734	CEP152	5.77561833517725e-41	0.707889508447053	0.036	0.015	1.26162606913612e-36	no change
3735	3735	CEP295	5.82512636726798e-41	0.444942141245573	0.055	0.028	1.27244060366602e-36	no change
3736	3736	SIRT7	5.96632043892461e-41	0.116256505352788	0.312	0.239	1.30328303667869e-36	no change
3737	3737	CLPX	6.0188603868037e-41	0.196667990600485	0.126	0.083	1.3147598628934e-36	no change
3738	3738	SARNP	6.04630245425087e-41	0.134356079650043	0.122	0.08	1.32075430810656e-36	no change
3739	3739	ZBTB33	6.05364863760517e-41	0.337893359385321	0.07	0.04	1.32235900839847e-36	no change
3740	3740	TTLL12	6.06832014499856e-41	0.275774971831308	0.101	0.063	1.32556385247348e-36	no change

3741	3741	RP5-821D11.7	6.58328315846228e-41	-1.35872039	0.021	0.044	1.4380523731345e-36	down
3742	3742	FANCF	6.79814959413068e-41	0.291238007244097	0.077	0.045	1.48498779734191e-36	no change
3743	3743	DHX57	6.92490113373933e-41	0.433572226617507	0.058	0.03	1.51267540365402e-36	no change
3744	3744	IQCK	7.17235272885427e-41	0.215256003231635	0.083	0.049	1.56672873009093e-36	no change
3745	3745	PPP1CA	7.23505749592506e-41	0.106343321939821	0.734	0.6	1.58042595940987e-36	no change
3746	3746	RP11-544A12.8	7.25581095672652e-41	0.315310775425335	0.056	0.029	1.58495934538734e-36	no change
3747	3747	COQ5	7.28030594772583e-41	0.175779541821342	0.123	0.081	1.59031003122123e-36	no change
3748	3748	WDR5	7.28152975938833e-41	0.372256969386447	0.072	0.041	1.59057736064079e-36	no change
3749	3749	NLN	7.48559414271829e-41	0.312965777289011	0.068	0.038	1.63515318453538e-36	no change
3750	3750	GUCD1	7.52032419095884e-41	0.205065799655594	0.125	0.083	1.64273961627305e-36	no change
3751	3751	TERF2	7.57481247215465e-41	0.332800117199036	0.074	0.042	1.65464203641746e-36	no change
3752	3752	CCDC127	7.59943795105779e-41	0.185818861950635	0.129	0.086	1.66002122602906e-36	no change
3753	3753	WDR92	7.78982670118079e-41	0.467997163950584	0.048	0.024	1.70160974460593e-36	no change

3754	3754	IFI27L2	8.02294281634462e-41	-0.367073149	0.586	0.561	1.75253162880232e-36	no change
3755	3755	FANCG	8.18949749091478e-41	0.685786873011289	0.036	0.015	1.78891383191542e-36	no change
3756	3756	TAF2	8.39456435131398e-41	0.142674179966931	0.112	0.072	1.83370863690103e-36	no change
3757	3757	AP3M1	8.47288297599015e-41	0.169901383680315	0.13	0.087	1.85081655727529e-36	no change
3758	3758	FADD	9.14911770223629e-41	0.279849312295837	0.084	0.05	1.9985332708765e-36	no change
3759	3759	FBLN2	9.31160833289456e-41	-1.688003141	0.073	0.107	2.03402772423749e-36	down
3760	3760	SLCO1A2	9.36878060550141e-41	0.904878067688094	0.02	0.005	2.04651643546573e-36	no change
3761	3761	ZC3HAV1	9.75176193360974e-41	-0.135175095	0.233	0.167	2.13017487677771e-36	no change
3762	3762	TMEM45A	9.76774703960989e-41	-0.803629065	0.163	0.204	2.13366666333238e-36	no change
3763	3763	NOL10	1.00157277868817e-40	0.150930173042133	0.102	0.064	2.18783557776644e-36	no change
3764	3764	DHX16	1.03400847217684e-40	0.26519967345408	0.098	0.061	2.25868810662309e-36	no change
3765	3765	TMEM222	1.10414028485791e-40	0.111535830523846	0.154	0.106	2.41188403824362e-36	no change
3766	3766	TNNC1	1.15221376755295e-40	-1.339256069	0.088	0.124	2.51689575384267e-36	down

3767	3767	SNX3	1.17431113133502e-40	-0.176462086	0.892	0.832	2.56516523528823e-36	no change
3768	3768	NDUFAF1	1.20248627150635e-40	0.111957177749821	0.134	0.089	2.62671101147847e-36	no change
3769	3769	NKRF	1.25613592648671e-40	0.325721177137403	0.051	0.025	2.74390331781756e-36	no change
3770	3770	ADCY5	1.26993485080785e-40	1.14581053164277	0.019	0.005	2.77404568810467e-36	up
3771	3771	ZMYND19	1.28520379982282e-40	0.431799938863986	0.054	0.028	2.80739918033297e-36	no change
3772	3772	ELN	1.36727919372237e-40	-1.478578813	0.025	0.05	2.98668467076714e-36	down
3773	3773	RP11-624L4.1	1.40103418367296e-40	0.536749421189777	0.042	0.02	3.06041907081522e-36	no change
3774	3774	LINC01060	1.40360753745413e-40	2.29444893520168	0.014	0.002	3.06604030481479e-36	up
3775	3775	UBE2L3	1.41159255104743e-40	0.100854220845929	0.707	0.568	3.08348276850801e-36	no change
3776	3776	CXCL2	1.41595314995219e-40	-0.151141885	0.193	0.139	3.09300806075556e-36	no change
3777	3777	GALNT12	1.43667237196518e-40	0.185158814650316	0.079	0.046	3.13826712932074e-36	no change
3778	3778	COL4A6	1.45017520970705e-40	0.257518768755707	0.074	0.042	3.16776272808408e-36	no change
3779	3779	RIOK2	1.5288909676373e-40	0.159843537860207	0.074	0.043	3.33970942970693e-36	no change

3780	3780	NREP	1.61481056743741e-40	0.333358013801214	0.042	0.019	3.52739220351028e-36	no change
3781	3781	NGLY1	1.6383194761675e-40	0.109773582608961	0.198	0.142	3.57874506374029e-36	no change
3782	3782	CXCL8	1.70140211390873e-40	-0.714336642	0.389	0.429	3.71654277762223e-36	no change
3783	3783	FAM161A	1.7203417716765e-40	0.529218397352235	0.044	0.021	3.75791456605014e-36	no change
3784	3784	KIF15	1.84641279070765e-40	1.5229011924582	0.016	0.003	4.03330410002179e-36	up
3785	3785	LRRC14	1.8919536717639e-40	0.329577849967378	0.063	0.035	4.13278360060106e-36	no change
3786	3786	LRCH3	1.93742135228626e-40	0.168404915744208	0.083	0.049	4.2321032019341e-36	no change
3787	3787	IFT80	1.96049919262476e-40	0.315271910029398	0.064	0.035	4.28251443636953e-36	no change
3788	3788	HTRA3	2.16804909763738e-40	-2.160597019	0.016	0.036	4.73588644887908e-36	down
3789	3789	ZBTB26	2.21322600458677e-40	1.19143904856189	0.024	0.008	4.83457088441935e-36	up
3790	3790	PMP22	2.22102977308786e-40	-1.691864985	0.055	0.085	4.85161743633312e-36	down
3791	3791	ALG14	2.22633385156113e-40	0.147204714681302	0.148	0.101	4.86320366535014e-36	no change
3792	3792	EVPL	2.24147552455647e-40	0.166804920859825	0.183	0.131	4.89627913584115e-36	no change

3793	3793	NOL12	2.41699413065043e-40	0.225440287627972	0.099	0.062	5.2796819789928e-36	no change
3794	3794	MFSD6	2.4663521941876e-40	0.164086696355018	0.105	0.067	5.3874997329834e-36	no change
3795	3795	GIPC2	2.54211028046441e-40	0.363497627738703	0.059	0.032	5.55298569664646e-36	no change
3796	3796	MLH1	2.6628624828147e-40	0.222580481847105	0.078	0.046	5.81675680746043e-36	no change
3797	3797	AP000251.3	2.81410728407156e-40	0.717393913266211	0.03	0.012	6.14713595132591e-36	no change
3798	3798	PGP	3.03382677699584e-40	0.110251483249534	0.285	0.215	6.62709121166971e-36	no change
3799	3799	DOCK8	3.03856537827819e-40	0.146998307064105	0.09	0.054	6.63744221231088e-36	no change
3800	3800	NEIL2	3.1281832311649e-40	0.181034789986021	0.093	0.057	6.8332034501566e-36	no change
3801	3801	PIBF1	3.1288118640618e-40	0.26972693261994	0.08	0.047	6.8345766358566e-36	no change
3802	3802	RAI1	3.14457349704159e-40	0.33411989418992	0.096	0.06	6.86900634693765e-36	no change
3803	3803	C11orf70	3.14801597513817e-40	0.403526385836543	0.062	0.034	6.87652609609183e-36	no change
3804	3804	B3GALNT2	3.2420077571314e-40	0.110161204490538	0.109	0.07	7.08184174467784e-36	no change
3805	3805	RP11-332H14.2	3.26821458781563e-40	0.308255998419234	0.073	0.042	7.13908794562445e-36	no change

3806	3806	ZCCHC7	3.27493514889423e-40	0.104969422146696	0.114	0.073	7.15376833924457e-36	no change
3807	3807	PDCD2L	3.32874301351119e-40	0.413374403975728	0.065	0.036	7.27130623871385e-36	no change
3808	3808	TMEM176B	3.39235429699267e-40	-1.445837526	0.049	0.079	7.41025872635079e-36	down
3809	3809	PWWP2A	3.50229165238797e-40	0.125228641143317	0.094	0.058	7.65040588547628e-36	no change
3810	3810	LINC00662	3.54457952606605e-40	0.265561908992694	0.099	0.062	7.74277951673869e-36	no change
3811	3811	AASS	3.6569084441182e-40	0.210605009089684	0.067	0.037	7.98815080533181e-36	no change
3812	3812	RP11-255M2.3	3.79819943589458e-40	0.382414488791778	0.05	0.025	8.29678684776811e-36	no change
3813	3813	COQ3	4.09271433449304e-40	0.356379910523075	0.064	0.035	8.94012519226659e-36	no change
3814	3814	COG2	4.09532174850101e-40	0.235092315165891	0.093	0.057	8.9458208274256e-36	no change
3815	3815	RP11-35G9.3	4.24200974168249e-40	0.326980589814119	0.071	0.041	9.26624607973123e-36	no change
3816	3816	LRRC8D	4.32125855379593e-40	0.518269926485194	0.049	0.024	9.43935718491184e-36	no change
3817	3817	CCNF	4.36339264970704e-40	0.470716663915068	0.037	0.016	9.53139490402006e-36	no change
3818	3818	TMEM161A	4.60720385586008e-40	0.168676279892058	0.107	0.068	1.00639761027408e-35	no change

3819	3819	TRIM31	4.66417254670899e-40	-0.719206876	0.304	0.34	1.01884185110311e-35	no change
3820	3820	FANCD2	4.80818434681434e-40	0.26950426912882	0.053	0.027	1.05029978871812e-35	no change
3821	3821	AC019117.1	4.82012186927815e-40	0.438664524657986	0.051	0.026	1.05290742112512e-35	no change
3822	3822	XPNPEP3	5.14500931561994e-40	0.214437937425306	0.083	0.05	1.12387583490402e-35	no change
3823	3823	RRAGD	5.40206327963848e-40	-1.194145472	0.03	0.056	1.18002670280423e-35	down
3824	3824	CCNE1	5.56052306594168e-40	0.408645535534898	0.077	0.046	1.2146406585243e-35	no change
3825	3825	CENPU	5.65516281141141e-40	0.520675331767223	0.051	0.026	1.23531376452471e-35	no change
3826	3826	TMEM97	5.69623607832868e-40	-0.670109173	0.519	0.503	1.24428580895012e-35	no change
3827	3827	SPAST	6.08146168251509e-40	0.304745916433344	0.071	0.041	1.3284344899286e-35	no change
3828	3828	DEF6	6.33338909102173e-40	0.225306523649765	0.084	0.05	1.38346551304279e-35	no change
3829	3829	PIGU	6.35580936724564e-40	0.127455922015944	0.152	0.104	1.38836299818114e-35	no change
3830	3830	ZNF587	6.40265552109357e-40	0.197104555780396	0.095	0.059	1.39859607202768e-35	no change
3831	3831	CLASP1	6.43994489443709e-40	0.233836545889239	0.073	0.042	1.40674156274084e-35	no change

3832	3832	C15orf48	6.46032170797173e-40	-1.06976301	0.135	0.176	1.41119267388934e-35	down
3833	3833	RP1-34B20.21	6.79438550160544e-40	0.535998895878109	0.053	0.028	1.48416556897069e-35	no change
3834	3834	TTC27	7.07238026718208e-40	0.382992361348599	0.051	0.025	1.54489074556325e-35	no change
3835	3835	ANGPTL4	7.5906788161635e-40	-0.800784243	0.225	0.264	1.65810788060276e-35	no change
3836	3836	MAP3K8	7.70439434128209e-40	-0.549781152	0.357	0.378	1.68294789990966e-35	no change
3837	3837	PEBP1	7.86075592639963e-40	0.114316359662236	0.866	0.759	1.71710352456273e-35	no change
3838	3838	FGFR1	7.86595129317042e-40	-1.234623651	0.034	0.061	1.71823840048015e-35	down
3839	3839	NDUFS5	7.87262787518731e-40	0.137925078013033	0.953	0.902	1.71969683305592e-35	no change
3840	3840	CILP	8.22002278101185e-40	-2.744421375	0.01	0.027	1.79558177628423e-35	down
3841	3841	ZNF618	8.34707652508187e-40	0.402391185066033	0.039	0.018	1.82333539613888e-35	no change
3842	3842	ZBTB48	8.62902181128289e-40	0.189311842506049	0.086	0.052	1.88492352445663e-35	no change
3843	3843	FAM189B	9.50605299183359e-40	0.189308939311834	0.121	0.08	2.07650221553613e-35	no change
3844	3844	TMEM131	9.78350560685192e-40	0.119372879031825	0.129	0.086	2.13710896476073e-35	no change

3845	3845	CASP8AP2	9.84821105578727e-40	0.2802752826663	0.074	0.043	2.15124322302617e-35	no change
3846	3846	MAP2K4	1.01120735672708e-39	0.267623956585641	0.101	0.065	2.20888135003462e-35	no change
3847	3847	SETD2	1.08187216724007e-39	0.161199431142663	0.107	0.069	2.36324156211922e-35	no change
3848	3848	ZNF785	1.09382541014777e-39	0.442497818923931	0.051	0.026	2.38935222592678e-35	no change
3849	3849	CATSPER2	1.25715084013923e-39	0.291639587369801	0.062	0.034	2.74612029520013e-35	no change
3850	3850	IRAK4	1.40038322826529e-39	0.210551197552205	0.105	0.067	3.0589971238227e-35	no change
3851	3851	NR2C1	1.46648098631694e-39	0.255300332744403	0.082	0.049	3.20338106651072e-35	no change
3852	3852	RNF219	1.4821830311902e-39	0.439923889056547	0.059	0.032	3.23768061333187e-35	no change
3853	3853	PCCA	1.5152233123952e-39	0.161982962331406	0.101	0.063	3.30985380359608e-35	no change
3854	3854	FAM46B	1.57565401638558e-39	-1.215201361	0.031	0.057	3.44185863339265e-35	down
3855	3855	TMCO4	1.57661641180436e-39	0.155356726281396	0.109	0.07	3.44396088994544e-35	no change
3856	3856	ATP11B	1.59793647979546e-39	0.108800695456878	0.15	0.103	3.49053244646521e-35	no change
3857	3857	UBR7	1.69941192938451e-39	0.345555674661184	0.06	0.033	3.71219541854753e-35	no change

3858	3858	HMGCL	1.72357783229772e-39	0.123320390600433	0.229	0.169	3.76498341687114e-35	no change
3859	3859	HS6ST2	1.84891931683455e-39	0.60231017879195	0.053	0.028	4.03877935569339e-35	no change
3860	3860	SIPA1L3	2.01326218705884e-39	0.167838818894511	0.09	0.055	4.39776992141132e-35	no change
3861	3861	GCN1	2.06866379433602e-39	0.227047328568405	0.098	0.062	4.51878919234759e-35	no change
3862	3862	ATP6V1F	2.11009894640191e-39	-0.199728916	0.85	0.768	4.60930013852034e-35	no change
3863	3863	POM121	2.12519994170171e-39	0.102040074909714	0.129	0.086	4.64228675265322e-35	no change
3864	3864	WDR4	2.20203575271102e-39	0.321443181981241	0.063	0.034	4.81012689822195e-35	no change
3865	3865	REPS2	2.21512380411625e-39	0.43384319788462	0.058	0.031	4.83871643771155e-35	no change
3866	3866	NAMPT	2.23060077236271e-39	-0.496233317	0.575	0.549	4.87252432714911e-35	no change
3867	3867	ORC3	2.24981827830216e-39	0.176999358538351	0.102	0.065	4.91450304712325e-35	no change
3868	3868	BCS1L	2.34539704004163e-39	0.113651689647745	0.099	0.062	5.12328529426694e-35	no change
3869	3869	C16orf59	2.65413037817756e-39	0.989101846915026	0.028	0.01	5.79768239809105e-35	no change
3870	3870	GEMIN5	2.68244023981971e-39	0.387434667144432	0.036	0.016	5.85952245986218e-35	no change

3871	3871	IER5L	2.83870326306324e-39	0.171271489820108	0.107	0.069	6.20086340783534e-35	no change
3872	3872	B9D1	2.91184445738288e-39	0.207380876920526	0.101	0.064	6.36063303270715e-35	no change
3873	3873	SLC36A4	2.9134786543959e-39	0.133320123417297	0.096	0.06	6.36420277266241e-35	no change
3874	3874	RNF6	2.96715360319105e-39	0.112636105480989	0.143	0.098	6.48145033081053e-35	no change
3875	3875	FAM76B	3.20786505711271e-39	0.249124406290599	0.078	0.046	7.00726043075701e-35	no change
3876	3876	HOXA9	3.4133198739567e-39	0.3570673781158	0.114	0.075	7.45605593267102e-35	no change
3877	3877	LRRC45	3.67718646317107e-39	0.265722777519258	0.094	0.059	8.03244611015089e-35	no change
3878	3878	CEP63	3.86197787126796e-39	0.122694523874139	0.129	0.086	8.43610446199773e-35	no change
3879	3879	ZNF677	3.96606683224028e-39	0.197074168853738	0.073	0.043	8.66347638834567e-35	no change
3880	3880	DNAJC24	4.12027024153589e-39	0.258969720358341	0.077	0.045	9.00031831561101e-35	no change
3881	3881	AC009478.1	4.12863743224346e-39	0.994254257292281	0.024	0.008	9.0185956069926e-35	no change
3882	3882	HSPBAP1	4.88673266077386e-39	0.446889637540207	0.054	0.028	1.06745788241944e-34	no change
3883	3883	SCYL2	5.73259118713838e-39	0.117781176271196	0.159	0.111	1.25222721891851e-34	no change

3884	3884	VPS13D	5.89292774453594e-39	0.194040662435448	0.103	0.066	1.28725113651643e-34	no change
3885	3885	GINS1	5.97565825071657e-39	0.47205268001199	0.038	0.017	1.30532278828653e-34	no change
3886	3886	AC016549.1	5.9980638947726e-39	0.278980931808314	0.051	0.026	1.31021707717413e-34	no change
3887	3887	ARHGEF16	6.49354875980108e-39	0.208990243411532	0.094	0.058	1.41845079109095e-34	no change
3888	3888	XDH	6.6389304639869e-39	0.597797460861492	0.042	0.02	1.4502079705533e-34	no change
3889	3889	DCAF16	6.74446425642778e-39	0.184264983707601	0.099	0.063	1.47326077217408e-34	no change
3890	3890	HIP1	6.85725162703051e-39	0.209464166377526	0.06	0.032	1.49789804540854e-34	no change
3891	3891	C2CD5	6.94897693363712e-39	0.355969304983562	0.053	0.028	1.51793452138369e-34	no change
3892	3892	KNTC1	7.25514931215039e-39	0.586284426518537	0.03	0.012	1.58481481574613e-34	no change
3893	3893	KIF16B	7.46179886238494e-39	0.175109325577758	0.102	0.065	1.62995534349937e-34	no change
3894	3894	ARHGAP35	7.87329627851089e-39	0.212210976882443	0.093	0.058	1.71984283907792e-34	no change
3895	3895	FBXO44	8.2357777336507e-39	0.209085836309356	0.085	0.052	1.79902328813866e-34	no change
3896	3896	FAM20B	8.75683519980598e-39	0.14988042710561	0.111	0.073	1.91284308104562e-34	no change

3897	3897	KCNK15	9.14340499993985e-39	-1.349089641	0.034	0.06	1.99728538818686e-34	down
3898	3898	EIF5A2	9.31990664436223e-39	0.376331612650757	0.036	0.016	2.03584040739448e-34	no change
3899	3899	DIAPH3	1.02255556329346e-38	1.21184924371947	0.017	0.004	2.23367037245824e-34	up
3900	3900	EPPK1	1.05553606138398e-38	-0.140576836	0.21	0.15	2.30571297248718e-34	no change
3901	3901	SPSB2	1.17491188519945e-38	0.221645294036365	0.097	0.061	2.56647752202968e-34	no change
3902	3902	ATG13	1.19583815033444e-38	0.135170584207797	0.094	0.059	2.61218885559055e-34	no change
3903	3903	NCAPG2	1.34789719721424e-38	0.696016424863517	0.032	0.013	2.94434663759478e-34	no change
3904	3904	ZMAT1	1.35812521438974e-38	0.480068703632011	0.032	0.013	2.96668871831296e-34	no change
3905	3905	C6orf136	1.37010053637288e-38	0.140363142746949	0.135	0.091	2.99284761165292e-34	no change
3906	3906	CDC25C	1.38340869954094e-38	1.45974517465575	0.016	0.004	3.02191796327723e-34	up
3907	3907	ZNF43	1.40376283915192e-38	0.445728101490399	0.055	0.029	3.06637954584346e-34	no change
3908	3908	HPCAL1	1.41021996417565e-38	0.217963904724159	0.106	0.068	3.08048448974528e-34	no change
3909	3909	HMGXB3	1.44635892352177e-38	0.11831534473065	0.116	0.076	3.15942643254095e-34	no change

3910	3910	ANAPC1	1.46866909400425e-38	0.186917292656694	0.09	0.055	3.20816076894289e-34	no change
3911	3911	AGBL5	1.63400198819384e-38	0.234490507293993	0.096	0.06	3.56931394301061e-34	no change
3912	3912	URB1	1.70985410205333e-38	0.259136079027328	0.049	0.024	3.7350053005253e-34	no change
3913	3913	DIS3L	1.72033486138676e-38	0.25108020685435	0.075	0.044	3.75789947121323e-34	no change
3914	3914	RP11-242D8.1	1.73698033094217e-38	0.258438868509062	0.082	0.049	3.79425983491008e-34	no change
3915	3915	PITPNA	1.74053534436754e-38	0.159132429107062	0.132	0.089	3.80202540623646e-34	no change
3916	3916	MDN1	1.77362622641368e-38	0.378143523323692	0.054	0.028	3.87430912897805e-34	no change
3917	3917	NIN	1.79453885889317e-38	0.119502877457131	0.08	0.048	3.91999068336624e-34	no change
3918	3918	DIRC2	1.86283611366229e-38	0.235345060000768	0.096	0.061	4.0691792066839e-34	no change
3919	3919	WWC3	1.97370926943269e-38	0.271332592295579	0.058	0.031	4.31137052814876e-34	no change
3920	3920	COPS7B	2.03845098318664e-38	0.238036343732887	0.099	0.063	4.45279232767289e-34	no change
3921	3921	NSUN4	2.06456668979236e-38	0.230203649940818	0.095	0.059	4.50983947718244e-34	no change
3922	3922	ZNF703	2.11668141363877e-38	-0.770274922	0.146	0.185	4.62367887995252e-34	no change

3923	3923	RP11-379B8.1	2.15312439436907e-38	0.528054373376672	0.038	0.017	4.70328492705979e-34	no change
3924	3924	FASTKD2	2.22318213435878e-38	0.162438886406639	0.09	0.056	4.85631905429333e-34	no change
3925	3925	PAX9	2.35652246821006e-38	1.17954086055695	0.019	0.005	5.14758767955806e-34	up
3926	3926	MFAP3L	2.4060341800242e-38	0.136441498487457	0.121	0.08	5.25574106284487e-34	no change
3927	3927	CABIN1	2.47814501276353e-38	0.159792162667629	0.088	0.054	5.41325996588066e-34	no change
3928	3928	CTC-459F4.3	2.5129448735014e-38	0.533677132753851	0.044	0.022	5.48927678167646e-34	no change
3929	3929	C12orf76	2.54537045461759e-38	0.109419795736716	0.167	0.118	5.56010722106667e-34	no change
3930	3930	PRELID1	2.55267452471171e-38	0.105011348464234	0.809	0.677	5.57606223178027e-34	no change
3931	3931	RPS6KA3	2.74824052431666e-38	0.103006215408993	0.168	0.119	6.00325660131731e-34	no change
3932	3932	CMTM8	2.99427716319892e-38	0.143234969742439	0.15	0.103	6.54069903529171e-34	no change
3933	3933	RMRP	3.04402114061662e-38	0.742589618266055	0.035	0.015	6.64935977956294e-34	no change
3934	3934	XPO4	3.40537759681035e-38	0.31194301769604	0.056	0.03	7.43870682247252e-34	no change
3935	3935	EXPH5	3.40861779185864e-38	0.372044821687675	0.055	0.029	7.44578470453601e-34	no change

3936	3936	ME2	3.81548583460852e-38	0.106324072836714	0.121	0.08	8.33454725711884e-34	no change
3937	3937	ANAPC2	3.91572113901113e-38	0.208256671127635	0.073	0.043	8.55350125605592e-34	no change
3938	3938	CUL4B	3.96079694147386e-38	0.191669194041532	0.101	0.065	8.6519648389555e-34	no change
3939	3939	NUTM2A-AS1	3.9953959539348e-38	0.1157025613867	0.171	0.122	8.72754292177517e-34	no change
3940	3940	CEP192	4.065601760023e-38	0.375942663004824	0.057	0.031	8.88090048459423e-34	no change
3941	3941	PICK1	4.25559472659987e-38	0.163250936551745	0.14	0.096	9.29592112078475e-34	no change
3942	3942	CBX7	4.34841674685378e-38	0.275042252303942	0.064	0.036	9.4986815418274e-34	no change
3943	3943	MPDZ	4.70582072610992e-38	0.444867009716631	0.044	0.021	1.02793947941145e-33	no change
3944	3944	AGPAT5	4.86626204074342e-38	0.214963950647661	0.08	0.048	1.06298628017999e-33	no change
3945	3945	LMCD1	4.8989725493242e-38	-0.890405055	0.055	0.086	1.07013156367438e-33	no change
3946	3946	C8orf44	5.20898005257827e-38	0.47734330005492	0.054	0.029	1.1378496026852e-33	no change
3947	3947	RRAGB	5.26020068219735e-38	0.147265063654663	0.102	0.065	1.14903823701919e-33	no change
3948	3948	ZNF219	5.51795456640703e-38	0.16997727358953	0.093	0.058	1.20534199548595e-33	no change

3949	3949	OCRL	6.20110101744435e-38	0.376391673793822	0.065	0.037	1.35456850625054e-33	no change
3950	3950	BAG3	6.24374275971715e-38	-1.017958281	0.276	0.304	1.36388316843261e-33	down
3951	3951	FO538757.2	6.30430018914167e-38	-0.525561185	0.308	0.339	1.37711133331611e-33	no change
3952	3952	PRELP	6.48719207302831e-38	-1.700823377	0.022	0.043	1.4170622364323e-33	down
3953	3953	CPT2	6.79817713430831e-38	0.266429099939751	0.084	0.052	1.48499381321831e-33	no change
3954	3954	PALM3	6.90454649424045e-38	-0.890127489	0.056	0.087	1.50822913620188e-33	no change
3955	3955	BEST1	7.0272131068065e-38	-0.693012119	0.162	0.202	1.53502443105081e-33	no change
3956	3956	CDC42EP1	7.17833838936503e-38	-0.693134688	0.176	0.216	1.5680362377729e-33	no change
3957	3957	FSTL4	7.51109525417459e-38	0.346489256675899	0.045	0.022	1.6407236473219e-33	no change
3958	3958	RP11-390P2.4	7.60868460366908e-38	0.478883308157585	0.059	0.032	1.66204106482547e-33	no change
3959	3959	FAM53B	8.01240962882306e-38	0.492929539211844	0.042	0.02	1.75023075932011e-33	no change
3960	3960	CLEC11A	8.21713668872553e-38	-1.30396632	0.067	0.1	1.7949513382852e-33	down
3961	3961	SFI1	8.31691475163341e-38	0.24994624689958	0.081	0.049	1.8167468583468e-33	no change

3962	3962	C1orf112	8.69684334084676e-38	0.686956765654667	0.029	0.011	1.89973845937457e-33	no change
3963	3963	ME1	9.49546648249162e-38	0.192342693963674	0.053	0.028	2.07418969843547e-33	no change
3964	3964	UBR3	1.00422950719939e-37	0.135772251321156	0.117	0.078	2.19363893552634e-33	no change
3965	3965	SPP1	1.00439622620356e-37	-0.965905375	0.051	0.082	2.19400311651905e-33	no change
3966	3966	UBE2O	1.02150147165063e-37	0.434228421149375	0.062	0.035	2.23136781467364e-33	no change
3967	3967	CRKL	1.0233652959166e-37	0.18314675387215	0.113	0.074	2.23543915240021e-33	no change
3968	3968	GALNT7	1.03285168149688e-37	0.101067262984378	0.112	0.073	2.25616121306178e-33	no change
3969	3969	FBXO6	1.03526359183758e-37	0.23300736182397	0.139	0.096	2.26142979001002e-33	no change
3970	3970	ATP9B	1.03592709835633e-37	0.292783058122293	0.072	0.042	2.26287915364957e-33	no change
3971	3971	PRIMPOL	1.05643171667694e-37	0.202599421000646	0.067	0.038	2.30766944190911e-33	no change
3972	3972	CCSER1	1.07949258139514e-37	0.336296113848072	0.08	0.049	2.35804359479955e-33	no change
3973	3973	TTBK2	1.11128306136116e-37	0.102685704155122	0.063	0.035	2.42748671923732e-33	no change
3974	3974	RP11-421L21.3	1.11267196703121e-37	0.383211871799107	0.064	0.036	2.43052064478298e-33	no change

3975	3975	YTHDF1	1.11977839732648e-37	0.121897542715679	0.133	0.091	2.44604393111997e-33	no change
3976	3976	ATP5I	1.15322461009008e-37	0.124814871593581	0.973	0.932	2.51910383828077e-33	no change
3977	3977	TMSB15A	1.21116420668384e-37	1.86381016052997	0.016	0.004	2.64566709308017e-33	up
3978	3978	DKK3	1.21159524323554e-37	-1.455607869	0.031	0.055	2.64660864932371e-33	down
3979	3979	GSPT2	1.24708295909731e-37	0.575847324848919	0.028	0.011	2.72412801585216e-33	no change
3980	3980	DERL3	1.26949393961554e-37	-0.160527258	0.036	0.016	2.77308256169618e-33	no change
3981	3981	AP1B1	1.3669247613045e-37	0.106976971311699	0.218	0.161	2.98591044859355e-33	no change
3982	3982	TMEM209	1.4466882857202e-37	0.188303290524169	0.081	0.049	3.1601458913272e-33	no change
3983	3983	FIBIN	1.55196331923497e-37	-2.216733275	0.011	0.028	3.39010867453687e-33	down
3984	3984	DNAL4	1.5549145903914e-37	0.165533425199467	0.163	0.116	3.39655543125096e-33	no change
3985	3985	SOS2	1.5641402012001e-37	0.118784879777347	0.093	0.058	3.4167078555015e-33	no change
3986	3986	ESCO2	1.66460684855707e-37	0.944221840133137	0.021	0.006	3.63616719998807e-33	no change
3987	3987	DYM	1.77643134244259e-37	0.121349300295719	0.113	0.074	3.88043662443159e-33	no change

3988	3988	SFRP4	1.78468282111708e-37	-2.579447751	0.008	0.023	3.89846115444815e-33	down
3989	3989	C12orf43	1.82607574374144e-37	0.180290988909801	0.096	0.061	3.9888798546288e-33	no change
3990	3990	UBL7-AS1	1.89714343910538e-37	0.282948966302711	0.07	0.041	4.1441201283818e-33	no change
3991	3991	ZNF277	1.94050032856913e-37	0.101089013856697	0.199	0.144	4.23882891772642e-33	no change
3992	3992	TMEM186	2.08426106748532e-37	0.276030565078689	0.071	0.041	4.55285987581493e-33	no change
3993	3993	SLC31A1	2.16407118042382e-37	0.157476865865899	0.104	0.067	4.7271970865178e-33	no change
3994	3994	FBN1	2.82651484259334e-37	-2.116772581	0.018	0.039	6.1742390221609e-33	down
3995	3995	TRIM52	3.12148225357417e-37	0.374144203400931	0.044	0.021	6.81856583470741e-33	no change
3996	3996	NPAS2	3.2025599091699e-37	0.157101561779232	0.154	0.108	6.99567186559072e-33	no change
3997	3997	FBXL4	3.424897790057e-37	0.225008301953035	0.081	0.049	7.4813467326005e-33	no change
3998	3998	IGFL1	3.6549114489341e-37	-2.069166961	0.04	0.067	7.98378856905166e-33	down
3999	3999	ZC3H7B	3.67648305264601e-37	0.298653908925283	0.069	0.04	8.03090958019995e-33	no change
4000	4000	TFEB	3.72950351647365e-37	0.203167688748617	0.063	0.035	8.14672748138504e-33	no change

4001	4001	INTU	3.79077327174088e-37	0.172137073248908	0.066	0.038	8.28056513479078e-33	no change
4002	4002	HPS4	3.85205348212846e-37	0.109491149723219	0.176	0.126	8.41442562636141e-33	no change
4003	4003	VASN	4.12439861297685e-37	-1.451818567	0.037	0.063	9.00933633018663e-33	down
4004	4004	RALGAPB	4.60318660842661e-37	0.112657983524668	0.137	0.094	1.00552008274471e-32	no change
4005	4005	FAM155A	4.79019367867946e-37	0.364384205820668	0.031	0.013	1.04636990717074e-32	no change
4006	4006	DONSON	4.82483119982557e-37	0.319473522301861	0.052	0.028	1.0539361272899e-32	no change
4007	4007	ADO	4.85796912088877e-37	0.279963716555956	0.064	0.036	1.06117477476694e-32	no change
4008	4008	GLUL	5.09520777388815e-37	-0.301745384	0.76	0.713	1.11299718612813e-32	no change
4009	4009	PMM1	5.25540363660411e-37	0.173154391055433	0.171	0.123	1.1479903703798e-32	no change
4010	4010	NUP43	5.42299058799067e-37	0.281107332300989	0.058	0.031	1.18459806404068e-32	no change
4011	4011	WWC1	5.47185590260869e-37	0.218723898107755	0.096	0.061	1.19527220336584e-32	no change
4012	4012	SLC16A3	5.54344735462968e-37	-0.601273639	0.329	0.355	1.21091064014531e-32	no change
4013	4013	MLH3	6.00043859509763e-37	0.174928514346548	0.076	0.045	1.31073580671313e-32	no change

4014	4014	RAB3GAP2	6.50466298367205e-37	0.110614629637233	0.127	0.086	1.42087858215332e-32	no change
4015	4015	CD244	7.02764368373292e-37	-3.300978932	0.002	0.011	1.53511848627462e-32	down
4016	4016	FBLN1	7.41793135014145e-37	-0.958710503	0.652	0.629	1.6203729241249e-32	no change
4017	4017	CALCOCO1	7.44501866168217e-37	0.1261930389426	0.141	0.097	1.62628987645785e-32	no change
4018	4018	PCDHB16	7.66551766298423e-37	0.323151055208952	0.028	0.011	1.67445567830227e-32	no change
4019	4019	MTL5	7.78962676094527e-37	0.459317719061429	0.037	0.017	1.70156606966089e-32	no change
4020	4020	APAF1	8.12394211176221e-37	0.217502512680436	0.067	0.038	1.77459391489334e-32	no change
4021	4021	TBX2	8.25748874308952e-37	0.396946192482603	0.129	0.089	1.80376584104047e-32	no change
4022	4022	UBIAD1	8.6007446438586e-37	0.1617276583142	0.103	0.067	1.87874666000447e-32	no change
4023	4023	ZNF281	9.1669155709997e-37	0.148461815286007	0.118	0.079	2.00242103732917e-32	no change
4024	4024	HTT	9.32644271157586e-37	0.210641943808445	0.091	0.057	2.03726814591663e-32	no change
4025	4025	VCPIP1	9.70439362027821e-37	0.146425014065109	0.083	0.051	2.11982774241357e-32	no change
4026	4026	RAB31	9.76181200018728e-37	-0.140032699	0.174	0.122	2.13237021332091e-32	no change

4027	4027	MLLT1	9.92694489300294e-37	0.128725875479811	0.114	0.075	2.16844184242756e-32	no change
4028	4028	SLC2A4RG	1.0029206099068e-36	0.10531645591176	0.186	0.135	2.19077978028041e-32	no change
4029	4029	HTR7	1.01032535947835e-36	1.73954578765299	0.016	0.004	2.20695471524451e-32	up
4030	4030	TAF4	1.01912086897935e-36	0.187952377522928	0.062	0.035	2.22616762619848e-32	no change
4031	4031	ADD3	1.02318123891663e-36	-0.189906537	0.259	0.191	2.23503709828948e-32	no change
4032	4032	SPRR1B	1.05206196019748e-36	-1.029489603	0.055	0.086	2.29812414585537e-32	down
4033	4033	FIGNL1	1.06260754280149e-36	0.699508135451421	0.036	0.016	2.32115991649558e-32	no change
4034	4034	CENPL	1.07789848850707e-36	0.411804772063715	0.041	0.02	2.35456145829485e-32	no change
4035	4035	RPS4Y2	1.09011085204165e-36	-1.854456215	0.01	0.026	2.38123814519977e-32	down
4036	4036	SDCCAG3	1.16909977297261e-36	0.355610678847523	0.059	0.033	2.55378154408137e-32	no change
4037	4037	TMED8	1.19782283269166e-36	0.207198444393471	0.075	0.045	2.61652419573166e-32	no change
4038	4038	HR	1.25683094181833e-36	0.314513348363121	0.052	0.027	2.74542150930796e-32	no change
4039	4039	SENP2	1.28231373494425e-36	0.130643405826909	0.111	0.073	2.80108612261223e-32	no change

4040	4040	IFT81	1.29988380452276e-36	0.233590530245182	0.071	0.042	2.83946618259952e-32	no change
4041	4041	PANK4	1.30794887549104e-36	0.428680677179511	0.041	0.02	2.85708352362263e-32	no change
4042	4042	NDUFAF7	1.41604635764035e-36	0.308985012485447	0.059	0.033	3.09321166362958e-32	no change
4043	4043	AC010287.1	1.45841002115683e-36	0.714630325202112	0.032	0.014	3.18575085021497e-32	no change
4044	4044	MIIP	1.62837130874705e-36	0.146852247798507	0.153	0.107	3.55701428682706e-32	no change
4045	4045	POLR1A	1.66108121456281e-36	0.375997581206993	0.046	0.024	3.62846580509099e-32	no change
4046	4046	TOM1L2	1.66234903545425e-36	0.108586794211913	0.154	0.109	3.63123523304625e-32	no change
4047	4047	GIT2	1.66852640157063e-36	0.166184130027431	0.09	0.057	3.64472907159088e-32	no change
4048	4048	PKM	1.6817630801265e-36	0.114123434454502	0.907	0.813	3.67364327222832e-32	no change
4049	4049	RP11-398K22.12	1.77389767534126e-36	0.758164301799942	0.029	0.012	3.87490208201545e-32	no change
4050	4050	AC002310.12	1.77413692964508e-36	0.410479011245314	0.059	0.033	3.87542470911672e-32	no change
4051	4051	ARL6IP4	1.7992046954528e-36	0.101403203398182	0.865	0.752	3.9301827367471e-32	no change
4052	4052	GSN	1.98734293017935e-36	-1.663141539	0.368	0.382	4.34115189668376e-32	down

4053	4053	ACP5	2.07680929988917e-36	-0.890162794	0.097	0.132	4.53658223467789e-32	no change
4054	4054	TMEM214	2.127534449842e-36	0.101312239531945	0.132	0.09	4.64738625223486e-32	no change
4055	4055	RHBDD3	2.13134527590741e-36	0.177551124628493	0.124	0.084	4.65571062069215e-32	no change
4056	4056	MTFR1	2.20322044615859e-36	0.110289252566464	0.132	0.091	4.81271474258881e-32	no change
4057	4057	NEU1	2.48993777782629e-36	-0.617046253	0.322	0.343	5.43902008188374e-32	no change
4058	4058	RP11-22N19.2	2.54004232817953e-36	-1.083405504	0.038	0.065	5.54846846167537e-32	down
4059	4059	ZNF768	2.57235218733974e-36	0.257984768661166	0.087	0.055	5.61904611802493e-32	no change
4060	4060	FEM1A	2.58665596707883e-36	0.136096685019751	0.105	0.069	5.650291294487e-32	no change
4061	4061	SLC35G2	2.64720769807885e-36	0.219297423780699	0.05	0.026	5.78256049568345e-32	no change
4062	4062	MADD	2.93539761166991e-36	0.220705221056091	0.08	0.048	6.41208254293175e-32	no change
4063	4063	DOHH	2.94584993599281e-36	0.117947425580001	0.137	0.094	6.4349146001827e-32	no change
4064	4064	MMP23B	3.39542650219914e-36	-1.434354602	0.04	0.067	7.41696965140379e-32	down
4065	4065	CSTF2	3.46517679641034e-36	0.374944406730401	0.057	0.032	7.56933219407875e-32	no change

4066	4066	WDR90	3.59491181508605e-36	0.53328957626636	0.037	0.017	7.85272536887397e-32	no change
4067	4067	TRMT10B	3.60381048269635e-36	0.270327476567074	0.072	0.042	7.87216361840192e-32	no change
4068	4068	ADAMTS1	3.61450567486211e-36	-1.315914833	0.077	0.109	7.89552619616879e-32	down
4069	4069	TAC3	3.66544964398307e-36	-2.2105066	0.016	0.036	8.00680820231663e-32	down
4070	4070	LINC01473	3.86063049284175e-36	0.185302889533324	0.064	0.036	8.43316124856352e-32	no change
4071	4071	MBD3	4.00992031720439e-36	0.127517662828392	0.089	0.056	8.75926994090126e-32	no change
4072	4072	FAAH	4.01376495070808e-36	0.13950801294399	0.13	0.089	8.76766815832672e-32	no change
4073	4073	RP11-849H4.2	4.06504544115141e-36	0.491062746753375	0.037	0.017	8.87968526165114e-32	no change
4074	4074	KIAA1841	4.18026817551982e-36	0.371880530904336	0.047	0.024	9.13137780260549e-32	no change
4075	4075	MORC2-AS1	4.33939420323624e-36	0.217591671713897	0.048	0.024	9.47897269754925e-32	no change
4076	4076	PPM1H	4.37102692454195e-36	0.381398906011676	0.08	0.049	9.54807121396943e-32	no change
4077	4077	CTD-2507G9.1	4.38947139444042e-36	1.25713085426999	0.017	0.005	9.58836131401565e-32	up
4078	4078	EREG	4.4260090482814e-36	-1.378356868	0.027	0.05	9.66817416506589e-32	down

4079	4079	C12orf66	4.44013300334374e-36	0.235186301167456	0.064	0.037	9.69902653250407e-32	no change
4080	4080	SCRIB	4.54301453741393e-36	0.158350101738485	0.079	0.048	9.923760955527e-32	no change
4081	4081	PEX6	4.75368803932388e-36	0.434764935207217	0.054	0.029	1.03839561530991e-31	no change
4082	4082	NINL	4.99330156551043e-36	0.479776913762564	0.041	0.02	1.0907367939701e-31	no change
4083	4083	LAMA3	5.42853098410656e-36	0.192948915875295	0.078	0.047	1.18580830816824e-31	no change
4084	4084	PDPR	5.43498015127719e-36	0.393659100146063	0.041	0.02	1.18721706424499e-31	no change
4085	4085	SPINK7	5.54750266903274e-36	0.599664190334295	0.033	0.014	1.21179648302351e-31	no change
4086	4086	KAT7	5.56532134808917e-36	0.101560984744524	0.112	0.074	1.2156887952766e-31	no change
4087	4087	VTCN1	5.72692176543929e-36	-1.383083228	0.029	0.052	1.25098879044256e-31	down
4088	4088	NCDN	6.34560152841563e-36	0.356660659986351	0.05	0.027	1.38613319786711e-31	no change
4089	4089	ICMT	6.34858132175784e-36	0.191496360794756	0.167	0.121	1.38678410392478e-31	no change
4090	4090	NUDT7	6.5620011313351e-36	0.42831108790354	0.035	0.015	1.43340352712884e-31	no change
4091	4091	MGAT5	6.80184316273017e-36	0.302103106677722	0.068	0.04	1.48579462046678e-31	no change

4092	4092	RSAD1	6.80850116479401e-36	0.241508647615268	0.087	0.055	1.4872489944376e-31	no change
4093	4093	LEMD3	7.16048697166489e-36	0.195739013559732	0.086	0.054	1.56413677409048e-31	no change
4094	4094	UBE3D	7.1618693494539e-36	0.354771628378915	0.052	0.028	1.56443874069471e-31	no change
4095	4095	ACVR2B	7.27714867930702e-36	0.470878344619879	0.05	0.026	1.58962035750782e-31	no change
4096	4096	RABL2B	7.33445752511366e-36	0.142349916167151	0.082	0.05	1.60213890178583e-31	no change
4097	4097	COG7	7.56091361865228e-36	0.215885136972492	0.079	0.048	1.6516059708584e-31	no change
4098	4098	POLR2F	7.8272310968315e-36	0.105343049256845	0.658	0.531	1.70978036079187e-31	no change
4099	4099	SS18L1	8.30888207833096e-36	0.169798010559828	0.071	0.042	1.81499220119061e-31	no change
4100	4100	HYOU1	8.55208681479609e-36	0.125724850364644	0.11	0.073	1.86811784382406e-31	no change
4101	4101	LTV1	8.62977353133212e-36	0.136624819649746	0.106	0.069	1.88508773018419e-31	no change
4102	4102	TYW5	8.77550811523681e-36	0.299290244021456	0.068	0.04	1.91692199269233e-31	no change
4103	4103	GAS2L3	9.09240604676038e-36	0.602942601990597	0.035	0.016	1.98614517685434e-31	no change
4104	4104	HSPB6	9.35178323704342e-36	-1.542259679	0.022	0.043	2.04280353029976e-31	down

4105	4105	ZNF507	9.39049588605511e-36	0.342128252032691	0.061	0.034	2.05125992134988e-31	no change
4106	4106	RP11-488L18.10	9.41522306003704e-36	0.134261919232198	0.066	0.038	2.05666132523449e-31	no change
4107	4107	METRN	1.01305568165605e-35	-0.93103082	0.061	0.092	2.21291883100948e-31	no change
4108	4108	MIER3	1.05848243805369e-35	0.277557057012575	0.05	0.026	2.31214903768449e-31	no change
4109	4109	NUP188	1.13062780428942e-35	0.327855992819002	0.044	0.022	2.4697433756898e-31	no change
4110	4110	ATP6V0E2	1.14577284709704e-35	-0.632485785	0.134	0.173	2.50282620719878e-31	no change
4111	4111	PLEKHG1	1.1559689968428e-35	0.461549942269622	0.053	0.028	2.5250986767034e-31	no change
4112	4112	OTUD3	1.16415369183348e-35	0.423871844400055	0.048	0.025	2.54297732444105e-31	no change
4113	4113	AC006262.5	1.26810049651934e-35	-2.727057466	0.01	0.026	2.77003872459684e-31	down
4114	4114	MSANTD3	1.29231605055661e-35	0.21084433781506	0.071	0.042	2.82293518083585e-31	no change
4115	4115	ZNF274	1.2948344187679e-35	0.199160837013094	0.074	0.044	2.82843630435659e-31	no change
4116	4116	UBR1	1.49512311256246e-35	0.137777748010162	0.075	0.045	3.26594692708143e-31	no change
4117	4117	PDPN	1.54209336656949e-35	-1.482223103	0.029	0.052	3.36854874993438e-31	down

4118	4118	ASB6	1.56340946749867e-35	0.449516428157122	0.047	0.024	3.41511164080409e-31	no change
4119	4119	ZNF395	1.61856475368941e-35	0.268348690058412	0.085	0.054	3.53559284795914e-31	no change
4120	4120	CEP120	1.64350585800507e-35	0.210101832718705	0.052	0.028	3.59007419622628e-31	no change
4121	4121	SLC12A9	1.73153308718597e-35	0.130884260322107	0.104	0.068	3.78236087564904e-31	no change
4122	4122	CXCL1	1.73606277072989e-35	-1.275047246	0.135	0.172	3.79225551638237e-31	down
4123	4123	SOCS4	1.7464071674269e-35	0.214782026200747	0.089	0.056	3.81485181652732e-31	no change
4124	4124	PGPEP1	1.82938684286621e-35	0.140837004304623	0.122	0.082	3.99611261955695e-31	no change
4125	4125	AARSD1	1.92376777643367e-35	0.129180856158929	0.071	0.042	4.20227833084171e-31	no change
4126	4126	TCF19	1.97229852195702e-35	0.644245376889485	0.037	0.017	4.30828889136292e-31	no change
4127	4127	APTX	2.21132163796762e-35	0.165180649640971	0.116	0.078	4.83041098597646e-31	no change
4128	4128	FKBP15	2.22190904632816e-35	0.260556163686315	0.054	0.029	4.85353812079924e-31	no change
4129	4129	NCOA5	2.22723779039714e-35	0.270661498506642	0.055	0.03	4.86517822934352e-31	no change
4130	4130	YBEY	2.22724783838876e-35	-0.122659177	0.382	0.288	4.86520017817641e-31	no change

4131	4131	POLDIP3	2.2464497066629e-35	0.118819074986842	0.126	0.086	4.90714473923445e-31	no change
4132	4132	MT1A	2.30705829477536e-35	-1.589949121	0.034	0.059	5.03953813910729e-31	down
4133	4133	AC007246.3	2.39012414184312e-35	0.131960879399269	0.164	0.117	5.2209871754421e-31	no change
4134	4134	NIFK-AS1	2.5122446562861e-35	0.356496348893731	0.053	0.029	5.48774722719135e-31	no change
4135	4135	EPB41L4A	2.53415474438841e-35	0.176504983374903	0.07	0.041	5.53560762364204e-31	no change
4136	4136	RLF	2.53823311423648e-35	0.129124645762423	0.104	0.068	5.54451641473817e-31	no change
4137	4137	NSMAF	2.65751570829811e-35	0.155667348084499	0.082	0.05	5.80507731320639e-31	no change
4138	4138	ZDHHC5	2.70288633711439e-35	0.106983470074794	0.137	0.095	5.90418491479267e-31	no change
4139	4139	ALOX12	2.7191998551106e-35	1.16569599867483	0.017	0.005	5.9398201635036e-31	up
4140	4140	ANKRD52	2.74483508023699e-35	0.470170700124988	0.052	0.028	5.99581774926968e-31	no change
4141	4141	BEND7	2.83242389606321e-35	0.388926416372764	0.045	0.023	6.18714675856048e-31	no change
4142	4142	LRP6	2.95297057478803e-35	0.126033272251556	0.073	0.043	6.45046892356698e-31	no change
4143	4143	POP1	3.17517410393461e-35	0.679767134579583	0.032	0.014	6.93585031263475e-31	no change
4144	4144	ERLIN1	3.28597486720274e-3	0.18083412742243	0.102	0.067	7.17788349991766e-3	no

			5	7			1	change
4145	4145	NDUFB4	3.6180870597787e-35	0.10911527851394<br>7	0.893	0.795	7.90334937338059e-3<br>1	no<br>change
4146	4146	CH17-373J23.1	3.6538078736396e-35	-1.085586797	0.079	0.112	7.98137791917834e-3<br>1	down
4147	4147	HS3ST5	3.66026151290931e-3<br>5	0.45543651515830<br>9	0.044	0.022	7.99547524879909e-3<br>1	no<br>change
4148	4148	FAM184B	3.85463955615481e-3<br>5	0.83788028251982<br>9	0.019	0.006	8.42007464646457e-3<br>1	no<br>change
4149	4149	GSTA1	3.89309095591056e-3<br>5	0.16356728649274<br>2	0.207	0.156	8.50406788409104e-3<br>1	no<br>change
4150	4150	BNIP3	3.9626829554789e-35	-0.466863393	0.268	0.305	8.65608464794811e-3<br>1	no<br>change
4151	4151	ANKFY1	4.01944402770757e-3<br>5	0.20331239156453<br>1	0.085	0.053	8.78007353412441e-3<br>1	no<br>change
4152	4152	GUF1	4.02497909830797e-3<br>5	0.29551981087426<br>3	0.074	0.045	8.79216434234393e-3<br>1	no<br>change
4153	4153	NEMP1	4.53387134775393e-3<br>5	0.47371273962723<br>8	0.037	0.017	9.90378857203368e-3<br>1	no<br>change
4154	4154	RCN3	4.94521400658629e-3<br>5	-1.712511495	0.023	0.044	1.08023254759871e-3<br>0	down
4155	4155	FAHD2B	5.01893854887389e-3<br>5	0.15934961644658<br>1	0.101	0.066	1.09633693661601e-3<br>0	no<br>change
4156	4156	DDIAS	5.04352136339159e-3<br>5	0.89833555515100<br>6	0.022	0.008	1.10170680661926e-3<br>0	no<br>change
4157	4157	EXOG	5.26710059196862e-3	0.21263785294441	0.111	0.074	1.15054545330963e-3	no

			5	8			0	change
4158	4158	ORAI2	5.29564325666613e-35	0.112961363831973	0.089	0.056	1.15678031298615e-30	no change
4159	4159	ARFGEF2	5.43043760909202e-35	0.10978036076354	0.113	0.075	1.18622479133006e-30	no change
4160	4160	NLK	5.64725650233716e-35	0.309773455850548	0.07	0.042	1.23358671037053e-30	no change
4161	4161	ZNF189	5.76929737629739e-35	0.495571245261489	0.034	0.015	1.2602453188784e-30	no change
4162	4162	RIC8B	5.93913768413645e-35	0.275904013266338	0.053	0.028	1.29734523572277e-30	no change
4163	4163	PPID	5.9428437759104e-35	0.109901837517554	0.143	0.1	1.29815479440987e-30	no change
4164	4164	NPEPL1	6.02691473875025e-35	0.103876550314193	0.117	0.078	1.31651925553261e-30	no change
4165	4165	STK11	6.47525639455791e-35	0.13035324711867	0.107	0.071	1.41445500682723e-30	no change
4166	4166	NCOA6	6.48263516958942e-35	0.232757025091814	0.089	0.056	1.41606682644511e-30	no change
4167	4167	IGSF9	6.68825221794121e-35	0.415291709898726	0.06	0.034	1.46098181448708e-30	no change
4168	4168	ZNF736	6.69302455036299e-35	0.400767310319969	0.051	0.028	1.46202428278129e-30	no change
4169	4169	SLITRK4	6.88722065679401e-35	1.72974284261217	0.013	0.003	1.50444448027008e-30	up
4170	4170	KIF11	7.12249587057965e-3	1.25788783954703	0.018	0.005	1.55583799796942e-3	up

			5				0	
4171	4171	PLAA	7.15827694742933e-35	0.228938232610404	0.062	0.035	1.56365401639646e-30	no change
4172	4172	PHTF1	7.39510340668514e-35	0.282112283741444	0.076	0.046	1.6153863881563e-30	no change
4173	4173	TRMT1L	7.57818831261564e-35	0.180646713384466	0.1	0.066	1.65537945500776e-30	no change
4174	4174	CDC7	7.8243365619189e-35	0.682488175061635	0.034	0.015	1.70914807858556e-30	no change
4175	4175	RP11-977G19.12	7.93714665781204e-35	0.567300148618542	0.03	0.013	1.73379031593246e-30	no change
4176	4176	GSK3A	8.76799088887662e-35	0.127390815414575	0.132	0.091	1.91527992976621e-30	no change
4177	4177	ADCY3	9.07205625542886e-35	0.257895333315362	0.043	0.022	1.98169996843588e-30	no change
4178	4178	XCR1	9.24139353708064e-35	-1.485203796	0.013	0.031	2.01869000423989e-30	down
4179	4179	RNF169	9.38650827192368e-35	0.204874192932698	0.093	0.06	2.05038886691901e-30	no change
4180	4180	RP11-1114A5.4	9.41649359056646e-35	0.378815732194099	0.04	0.02	2.05693885992334e-30	no change
4181	4181	XXbac-BPGBPG55C20.2	9.88260669805225e-35	0.481346242918577	0.037	0.017	2.15875660712253e-30	no change
4182	4182	ZNF350-AS1	1.02098932180218e-34	0.140028847855481	0.081	0.05	2.23024907454469e-30	no change
4183	4183	CEP97	1.03481799527759e-3	0.47383408146114	0.041	0.02	2.26045642888436e-3	no

			4	2			0	change
4184	4184	ARID5B	1.05195840890112e-34	-0.57034995	0.419	0.317	2.29789794840361e-30	no change
4185	4185	EMSY	1.0622681796853e-34	0.224524088624515	0.071	0.042	2.32041861170456e-30	no change
4186	4186	LINC00992	1.08963910207859e-34	0.444696274611066	0.062	0.036	2.38020765458048e-30	no change
4187	4187	FGL2	1.18792690618887e-34	-1.720715614	0.034	0.058	2.59490753387898e-30	down
4188	4188	PTH2R	1.28775274729757e-34	1.16002713183765	0.018	0.005	2.81296710119681e-30	up
4189	4189	DHTKD1	1.29723749494636e-34	0.116927376128455	0.096	0.062	2.83368558396083e-30	no change
4190	4190	CEACAM6	1.32651202076372e-34	-2.267319099	0.014	0.032	2.89763285815626e-30	down
4191	4191	VGLL3	1.3625154167312e-34	0.111095150893058	0.157	0.113	2.97627867630763e-30	no change
4192	4192	ATPAF2	1.36319636818426e-34	0.139458385115802	0.116	0.078	2.9777661466617e-30	no change
4193	4193	TUBD1	1.39201360059848e-34	0.152344242444079	0.066	0.039	3.04071450914732e-30	no change
4194	4194	THNSL2	1.45902296334621e-34	0.241655701492818	0.068	0.04	3.18708976113347e-30	no change
4195	4195	HDAC8	1.47296760958027e-34	0.193284466200717	0.097	0.063	3.21755044636715e-30	no change
4196	4196	PISD	1.49657770414167e-3	0.16734939567321	0.12	0.082	3.26912433692706e-3	no

			4	8			0	change
4197	4197	PGS1	1.58760316907938e-34	0.238924338082944	0.089	0.057	3.46796036253699e-30	no change
4198	4198	H1F0	1.6008472899018e-34	-0.647314389	0.248	0.28	3.49689082006149e-30	no change
4199	4199	STK26	1.61226876453091e-34	0.345391522691617	0.093	0.06	3.52183988924131e-30	no change
4200	4200	NELL2	1.6328565304339e-34	1.21028995802679	0.015	0.004	3.56681180507981e-30	up
4201	4201	PPP1R9A	1.70371560551762e-34	0.431510271124354	0.079	0.049	3.72159636869268e-30	no change
4202	4202	TPPP	1.70765639406133e-34	0.453228411552152	0.035	0.016	3.73020462718757e-30	no change
4203	4203	RXRB	1.73614603759884e-34	0.114623005911469	0.106	0.07	3.79243740453092e-30	no change
4204	4204	HACD1	2.02843559052744e-34	0.408891628985267	0.054	0.03	4.43091470394815e-30	no change
4205	4205	ARHGEF9	2.04604441169691e-34	0.148751692442703	0.078	0.048	4.46937941291073e-30	no change
4206	4206	GTPBP8	2.13199329816936e-34	0.165910002961213	0.101	0.066	4.65712616052115e-30	no change
4207	4207	TCF3	2.2474182411178e-34	0.171497158617811	0.102	0.067	4.90926040589772e-30	no change
4208	4208	COL16A1	2.27829388351318e-34	-1.355729605	0.03	0.054	4.9767051591462e-30	down
4209	4209	NHLRC2	2.30339015504342e-3	0.19344837324797	0.084	0.053	5.03152545467685e-3	no

			4	8			0	change
4210	4210	CACNB3	2.498476501693e-34	0.233933688797174	0.101	0.067	5.45767207029819e-30	no change
4211	4211	PLAC8	2.70622899771111e-34	-1.2707294	0.028	0.051	5.91148662260015e-30	down
4212	4212	PID1	2.72454822696509e-34	-1.587889879	0.021	0.041	5.95150314698255e-30	down
4213	4213	PPP1R14D	2.7853516253078e-34	-2.110885026	0.006	0.019	6.08432209032236e-30	down
4214	4214	CSK	2.91676962045875e-34	0.172220410625755	0.106	0.07	6.3713915589301e-30	no change
4215	4215	SNX10	3.02451072183407e-34	0.295509573900842	0.094	0.062	6.60674122077434e-30	no change
4216	4216	CPSF4	3.23710730450639e-34	0.169774857016823	0.125	0.086	7.07113719596375e-30	no change
4217	4217	C4orf36	3.89079203623295e-34	0.216509740351535	0.071	0.043	8.49904612394725e-30	no change
4218	4218	TNFAIP2	3.96013823040181e-34	-0.473934553	0.532	0.507	8.65052595048972e-30	no change
4219	4219	TRUB1	4.09494516987429e-34	0.294262937036121	0.081	0.051	8.94499822907339e-30	no change
4220	4220	ZCWPW1	4.20807020158015e-34	0.102829214669776	0.078	0.047	9.19210854833169e-30	no change
4221	4221	GRTP1	4.35425015200414e-34	0.166399348448941	0.096	0.062	9.51142403203783e-30	no change
4222	4222	POLR2J	4.5841581416989e-34	-0.247879934	0.741	0.669	1.00136350447271e-2	no

							9	change
4223	4223	LRP8	4.8430306934927e-34	0.916143712413809	0.017	0.005	1.05791162468655e-29	no change
4224	4224	KRTAP5-10	4.95288084163999e-34	0.350292013271599	0.059	0.033	1.08190729104784e-29	no change
4225	4225	PLEKHN1	5.25201792651541e-34	0.100194013879794	0.137	0.095	1.14725079586803e-29	no change
4226	4226	TDRP	5.25615455914877e-34	0.136982143684887	0.07	0.041	1.14815440190046e-29	no change
4227	4227	ZNF516	5.28182799605509e-34	0.335639288616239	0.048	0.026	1.15376250745827e-29	no change
4228	4228	RMND1	6.153418085192e-34	0.252687681187953	0.073	0.044	1.34415264652934e-29	no change
4229	4229	PI4KA	6.1621654204795e-34	0.182225713325507	0.08	0.049	1.34606341444954e-29	no change
4230	4230	CA2	6.41767524113923e-34	-1.119095685	0.082	0.114	1.40187697967445e-29	down
4231	4231	TAGLN	6.85777357880946e-34	-0.780113795	0.06	0.09	1.49801206055514e-29	no change
4232	4232	RANBP6	6.94489947575303e-34	0.495960344527344	0.042	0.021	1.51704384148349e-29	no change
4233	4233	VANGL1	7.31535299887821e-34	0.219203420045512	0.083	0.052	1.59796570907496e-29	no change
4234	4234	FASTKD1	7.46113100696716e-34	0.126154162653444	0.076	0.046	1.62980945716191e-29	no change
4235	4235	ANKRD27	8.13808640248429e-3	0.24174607086427	0.074	0.046	1.77768359375867e-2	no

			4	1			9	change
4236	4236	WWC2	8.19445107717742e-34	0.196782154092576	0.043	0.022	1.78999589329864e-29	no change
4237	4237	GPR37L1	8.29216513584961e-34	-2.428101348	0.003	0.013	1.81134055227499e-29	down
4238	4238	SPIN2B	8.29864436207663e-34	0.21733639538706	0.087	0.055	1.81275587445202e-29	no change
4239	4239	RNF111	8.54898087889991e-34	0.151805996108243	0.1	0.066	1.8674393831869e-29	no change
4240	4240	SIGMAR1	8.66194825327858e-34	0.110269370162083	0.148	0.105	1.89211597644617e-29	no change
4241	4241	BTN2A1	8.73639398792734e-34	0.2840740673114	0.063	0.036	1.90837790272285e-29	no change
4242	4242	TMEM67	8.81695617017256e-34	0.192641636812611	0.053	0.029	1.92597590581249e-29	no change
4243	4243	ARNTL	9.0388750214522e-34	0.487006139365302	0.038	0.019	1.97445185968602e-29	no change
4244	4244	SKP2	1.00683378463646e-33	0.309983891798821	0.085	0.055	2.19932771915988e-29	no change
4245	4245	SETMAR	1.01351599045348e-33	0.350685956766638	0.06	0.034	2.21392432954659e-29	no change
4246	4246	PRR3	1.01636028105744e-33	0.259951826211129	0.071	0.043	2.22013739794188e-29	no change
4247	4247	ACOT7	1.07235891170603e-33	0.271813197503167	0.117	0.08	2.34246080673064e-29	no change
4248	4248	ZNF639	1.07756254871741e-3	0.14588762768021	0.092	0.059	2.3538276314183e-29	no

			3	6				change
4249	4249	TEX30	1.11847831718201e-33	0.147810920866033	0.119	0.081	2.44320403605238e-29	no change
4250	4250	CFAP44	1.22516894573638e-33	0.49004162868768	0.033	0.015	2.67625904506656e-29	no change
4251	4251	KDM6A	1.25189980392136e-33	0.218002069653755	0.079	0.049	2.73464993168582e-29	no change
4252	4252	TMEM44	1.29038440558293e-33	0.41314790322334	0.044	0.023	2.81871569555535e-29	no change
4253	4253	F8A1	1.38037759512668e-33	0.118132258635996	0.132	0.092	3.01529681879472e-29	no change
4254	4254	MINPP1	1.42228437630825e-33	0.132091309449565	0.092	0.059	3.10683799160774e-29	no change
4255	4255	LINC00470	1.48532252604182e-33	0.606572432820435	0.024	0.009	3.24453852588575e-29	no change
4256	4256	PDE9A	1.51374394287748e-33	0.204471633932903	0.077	0.047	3.30662226882157e-29	no change
4257	4257	C9orf69	1.55105510007156e-33	0.36116988225679	0.054	0.03	3.38812476059631e-29	no change
4258	4258	DDHD1	1.56379693141068e-33	0.10581585024911	0.108	0.072	3.41595801697348e-29	no change
4259	4259	ELF4	1.5873853406224e-33	0.249021462699285	0.098	0.064	3.46748453805557e-29	no change
4260	4260	GPN2	1.68627018591139e-33	0.139001595541517	0.144	0.102	3.68348859410484e-29	no change
4261	4261	ARHGEF28	1.70782547171796e-3	0.21670629213563	0.061	0.035	3.73057396042071e-2	no

			3	5			9	change
4262	4262	GTF3C1	1.7223225904124e-33	0.260438480358801	0.072	0.044	3.76224146649686e-29	no change
4263	4263	NHS	1.72408019381063e-33	0.166666427650809	0.057	0.032	3.76608077535993e-29	no change
4264	4264	EZR	1.77999553550468e-33	-0.156906324	0.78	0.734	3.88822224775642e-29	no change
4265	4265	ADNP2	1.78309684922367e-33	0.128577852505124	0.106	0.071	3.89499675744418e-29	no change
4266	4266	PARP8	1.99313451526392e-33	0.142189411748477	0.074	0.045	4.3538030351425e-29	no change
4267	4267	MYO19	1.99370127189563e-33	0.170918878188973	0.099	0.065	4.35504105832882e-29	no change
4268	4268	HIF1AN	2.20270684964388e-33	0.194653627876431	0.088	0.057	4.81159284236209e-29	no change
4269	4269	METTL2A	2.28180607066913e-33	0.174164201874272	0.093	0.06	4.98437718076964e-29	no change
4270	4270	MCM8	2.30527638999132e-33	0.471263811375113	0.034	0.015	5.03564574629704e-29	no change
4271	4271	NR1D1	2.30553928789608e-33	-1.037992761	0.055	0.083	5.03622002048021e-29	down
4272	4272	DGCR14	2.43466130122119e-33	0.14370571100776	0.079	0.049	5.31827414638758e-29	no change
4273	4273	MTRF1L	2.74131186087195e-33	0.123513132282554	0.124	0.086	5.98812162888868e-29	no change
4274	4274	IFRD1	2.75806666559703e-3	-0.525507651	0.423	0.427	6.02472082433015e-2	no

			3				9	change
4275	4275	RP4-710M16.2	2.75926249102072e-33	-1.252724035	0.031	0.054	6.02733298538567e-29	down
4276	4276	XRCC6BP1	2.79626264174914e-33	0.264614096070829	0.083	0.053	6.10815611463683e-29	no change
4277	4277	DFNA5	2.84053159371544e-33	0.507148345822901	0.043	0.022	6.204857213312e-29	no change
4278	4278	CCDC18	2.90309902627655e-33	0.510021486672174	0.039	0.019	6.34152951299849e-29	no change
4279	4279	OXNAD1	3.04952782884041e-33	0.278545525841587	0.062	0.036	6.661388589319e-29	no change
4280	4280	SENP3	3.28575119597497e-33	0.537508965037616	0.036	0.017	7.17739491248773e-29	no change
4281	4281	AATBC	3.40374624115735e-33	0.498121178845587	0.058	0.033	7.43514328918412e-29	no change
4282	4282	EFNA4	3.43779647769562e-33	0.169653636139047	0.143	0.101	7.50952262587831e-29	no change
4283	4283	SUV39H2	3.48897906648673e-33	0.304338658786034	0.055	0.031	7.6213258728336e-29	no change
4284	4284	MED27	3.57214611781051e-33	0.216876905362164	0.061	0.035	7.80299597974529e-29	no change
4285	4285	METTL16	3.80794536084462e-33	0.165002585099006	0.098	0.064	8.318075846229e-29	no change
4286	4286	CPAMD8	3.94815750674835e-33	0.428994228828497	0.039	0.019	8.6243552577411e-29	no change
4287	4287	ZNF26	4.04128018843184e-3	0.28310863011987	0.066	0.04	8.82777244361052e-2	no

			3	5			9	change
4288	4288	CKMT2-AS1	4.0852453883066e-33	0.574129374170149	0.033	0.015	8.92381002621693e-29	no change
4289	4289	EXOSC9	4.10391621301671e-33	0.12587495566298	0.072	0.043	8.96459457571371e-29	no change
4290	4290	VKORC1L1	4.19703478402499e-33	0.112918617756803	0.117	0.08	9.16800278222419e-29	no change
4291	4291	EBF4	4.24818270060142e-33	0.220999085764918	0.061	0.035	9.27973029119375e-29	no change
4292	4292	RP4-665J23.1	4.37703558901817e-33	0.295807750827542	0.062	0.036	9.5611965406513e-29	no change
4293	4293	KDM2B	4.55263766398743e-33	0.222187118145649	0.083	0.053	9.94478171321414e-29	no change
4294	4294	EEFSEC	4.60028290719057e-33	0.220220958187449	0.068	0.041	1.00488579824671e-28	no change
4295	4295	N4BP2	4.65405562205471e-33	0.309957817798972	0.062	0.036	1.01663191008163e-28	no change
4296	4296	EPT1	4.70225761036199e-33	0.242718601675022	0.09	0.058	1.02716115240747e-28	no change
4297	4297	ARMCX5	4.95545347426158e-33	0.220723684293128	0.073	0.044	1.0824692569177e-28	no change
4298	4298	DLEU1	5.36107668700335e-33	0.101222556894429	0.115	0.078	1.17107359150901e-28	no change
4299	4299	TMCO6	5.59723371926275e-33	0.350230294498346	0.048	0.026	1.22265973363575e-28	no change
4300	4300	FBXL19	5.76653858277575e-3	0.25917470921923	0.055	0.031	1.25964268802153e-2	no

			3	1			8	change
4301	4301	MARS2	6.0431586390198e-33	0.541386690942213	0.031	0.014	1.32006757310748e-28	no change
4302	4302	RRP8	6.12691685336088e-33	0.112930470345242	0.091	0.059	1.33836371744815e-28	no change
4303	4303	CPLX1	7.22689252341488e-33	1.05730055366082	0.018	0.005	1.57864240281475e-28	up
4304	4304	FAM3D	7.91679879688475e-33	0.217832681030004	0.087	0.056	1.7293455291915e-28	no change
4305	4305	LRP1B	8.35588488267569e-33	1.19745331092863	0.02	0.007	1.82525949377168e-28	up
4306	4306	PACSIN3	8.79075292119172e-33	0.183079154126925	0.147	0.105	1.92025206810512e-28	no change
4307	4307	CAAP1	1.01691595041338e-32	0.1587730330968	0.094	0.061	2.22135120208299e-28	no change
4308	4308	C9orf64	1.10488826472468e-32	0.361738806318109	0.043	0.022	2.4135179254646e-28	no change
4309	4309	EPC2	1.10838455433514e-32	0.18938921645018	0.086	0.055	2.42115522048967e-28	no change
4310	4310	NFRKB	1.14001973426777e-32	0.234982799190012	0.092	0.06	2.49025910753453e-28	no change
4311	4311	RP11-392P7.6	1.14080753570516e-32	0.192469644112613	0.101	0.067	2.49197998099436e-28	no change
4312	4312	HLA-DRA	1.16740810767861e-32	-0.542773509	0.462	0.493	2.55008627041316e-28	no change
4313	4313	SH3GL2	1.19023613134608e-3	0.59635929507207	0.042	0.022	2.59995180531239e-2	no

			2				8	change
4314	4314	KC6	1.19701723099731e-32	0.985878472900158	0.016	0.004	2.61476443939053e-28	no change
4315	4315	EFCAB11	1.20219953364768e-32	0.19229922742007	0.086	0.055	2.6260846613e-28	no change
4316	4316	ZNF100	1.2160960602688e-32	0.465842792596796	0.038	0.019	2.65644023405118e-28	no change
4317	4317	ZNF766	1.22694146491507e-32	0.216651631528645	0.077	0.048	2.68013093596049e-28	no change
4318	4318	TRMU	1.24875445177568e-32	0.158585393432254	0.099	0.065	2.72777922445879e-28	no change
4319	4319	STK35	1.26755420107092e-32	0.252312929609053	0.057	0.032	2.76884539681932e-28	no change
4320	4320	NUBPL	1.27632678280027e-32	0.255962410570484	0.059	0.034	2.7880082243489e-28	no change
4321	4321	ADORA2B	1.30278760501007e-32	0.239415259846212	0.05	0.027	2.84580924438399e-28	no change
4322	4322	LINC00094	1.3295409235007e-32	0.43870430132149	0.043	0.022	2.90424919329492e-28	no change
4323	4323	ABCB7	1.33150340478323e-32	0.136080259288744	0.068	0.041	2.90853603740849e-28	no change
4324	4324	SASH1	1.36096932874618e-32	0.105396839143895	0.123	0.085	2.97290140171316e-28	no change
4325	4325	ZNF740	1.37938546732933e-32	0.535718441554358	0.035	0.017	3.01312961483418e-28	no change
4326	4326	UEVLD	1.39682641706818e-3	0.17975712535406	0.084	0.053	3.05122762544373e-2	no

			2	7			8	change
4327	4327	ZNF711	1.42594426493516e-32	1.04501017514047	0.02	0.007	3.11483265232437e-28	up
4328	4328	TBX1	1.50836471928278e-32	0.379394668723992	0.053	0.03	3.29487189280131e-28	no change
4329	4329	CPNE4	1.52060688893811e-32	-2.04951067	0.003	0.014	3.32161368819641e-28	down
4330	4330	NRDE2	1.61081701506436e-32	0.134265515063246	0.105	0.07	3.5186686877066e-28	no change
4331	4331	KCNAB2	1.71688723436709e-32	0.610627674578134	0.032	0.014	3.75036847475147e-28	no change
4332	4332	MAU2	1.80269431356036e-32	0.302002213715248	0.065	0.039	3.93780545854125e-28	no change
4333	4333	NR3C1	1.95870387851163e-32	-0.131303929	0.147	0.103	4.27859275222081e-28	no change
4334	4334	RP11-159D12.2	2.00516785978797e-32	0.649294050744363	0.027	0.011	4.38008867292085e-28	no change
4335	4335	CRLF3	2.14747204814439e-32	0.293295770153328	0.065	0.038	4.6909379419666e-28	no change
4336	4336	FAM84A	2.16938746108416e-32	-0.194694517	0.468	0.361	4.73880996999224e-28	no change
4337	4337	VPS52	2.24513441009363e-32	0.132226443943706	0.097	0.064	4.90427160540853e-28	no change
4338	4338	MRM1	2.31433765585087e-32	0.421740909590635	0.051	0.028	5.05543917544063e-28	no change
4339	4339	KIAA1671	2.37087722731522e-3	0.29286855528205	0.055	0.031	5.17894421534737e-2	no

			2	1			8	change
4340	4340	ANKS6	2.41300245158452e-32	0.507749297559745	0.035	0.017	5.27096255524122e-28	no change
4341	4341	FAM234B	2.53303942381783e-32	0.343612465667811	0.059	0.034	5.53317131738766e-28	no change
4342	4342	FOXJ3	2.61018325401732e-32	0.143066398701219	0.102	0.068	5.70168430007543e-28	no change
4343	4343	ZNF224	2.64728021367439e-32	0.167419489586769	0.089	0.057	5.78271889875033e-28	no change
4344	4344	FBXO5	2.74197202781877e-32	0.436614949293324	0.033	0.015	5.98956369756733e-28	no change
4345	4345	CDS1	2.81875504638279e-32	0.106880793997587	0.169	0.123	6.15728852331856e-28	no change
4346	4346	TBC1D10B	2.8287979902197e-32	0.198160747785466	0.091	0.059	6.1792263298359e-28	no change
4347	4347	MED12	2.96364932686966e-32	0.267419419662269	0.064	0.038	6.47379558961409e-28	no change
4348	4348	C16orf58	3.11748980212297e-32	0.100826507312866	0.09	0.058	6.80984472375741e-28	no change
4349	4349	CCNH	3.161680368577e-32	-0.112328452	0.164	0.116	6.90637459711959e-28	no change
4350	4350	TMEM133	3.47146866076203e-32	0.510035277126051	0.031	0.014	7.58307614256857e-28	no change
4351	4351	USP24	3.54800390435179e-32	0.277488917167136	0.06	0.035	7.75025972866605e-28	no change
4352	4352	SNRPG	3.67235003012999e-3	0.10316085047953	0.914	0.829	8.02188140581594e-2	no

			2	2			8	change
4353	4353	URB2	3.68679531351792e-32	0.454912910864043	0.03	0.013	8.05343568284854e-28	no change
4354	4354	EPS15L1	4.01195639857917e-32	0.116971804077501	0.104	0.07	8.76371755705634e-28	no change
4355	4355	RP11-499P20.2	4.05899286096805e-32	0.161885706188443	0.045	0.024	8.86646400549861e-28	no change
4356	4356	LINC01560	4.11174069418894e-32	0.362738492398179	0.057	0.033	8.98168637238632e-28	no change
4357	4357	MYH10	4.55305559193506e-32	0.25327624273443	0.063	0.037	9.94569463502295e-28	no change
4358	4358	KDM5C	4.77283472559376e-32	0.158078263674022	0.1	0.066	1.0425780174587e-27	no change
4359	4359	MEX3C	5.03463449606667e-32	0.147908518169344	0.105	0.071	1.0997655593208e-27	no change
4360	4360	ITFG2	5.07297766914546e-32	0.11299884413045	0.103	0.069	1.10814124204813e-27	no change
4361	4361	RAD54B	5.17203721027028e-32	0.617195343463356	0.034	0.016	1.12977980821144e-27	no change
4362	4362	DUS3L	5.18303666808833e-32	0.191445131743053	0.077	0.048	1.13218252977722e-27	no change
4363	4363	IPO4	5.64856106482447e-32	0.354003629701618	0.039	0.02	1.23387167900026e-27	no change
4364	4364	TNFRSF25	5.69422523406769e-32	0.23471661592107	0.051	0.028	1.24384656012975e-27	no change
4365	4365	HDAC6	5.88529004677294e-3	0.16975828842486	0.062	0.036	1.28558275781708e-2	no

			2	1			7	change
4366	4366	PCAT6	5.96181960938538e-32	-1.010587061	0.093	0.125	1.30229987547414e-27	down
4367	4367	LINC00205	6.00956236443526e-32	0.547226290475472	0.033	0.015	1.31272880288724e-27	no change
4368	4368	IDE	6.02889728203804e-32	0.273017325125603	0.091	0.06	1.31695232228839e-27	no change
4369	4369	FOXN2	6.20363823005761e-32	0.112998014278746	0.08	0.05	1.35512273497378e-27	no change
4370	4370	USP6NL	6.62938383062222e-32	0.113448782035562	0.096	0.063	1.44812260396112e-27	no change
4371	4371	CPOX	6.7526013314718e-32	0.371237419447247	0.06	0.035	1.4750382348467e-27	no change
4372	4372	IRGQ	6.99824240308047e-32	0.289362294390817	0.058	0.034	1.5286960705289e-27	no change
4373	4373	SPAG17	7.6611720247163e-32	0.349533022227806	0.059	0.034	1.67350641707903e-27	no change
4374	4374	YAE1D1	7.66201766468081e-32	0.165393527217748	0.088	0.057	1.67369113867288e-27	no change
4375	4375	AC010127.3	8.33608130490842e-32	0.80719018788979	0.022	0.008	1.8209336002442e-27	no change
4376	4376	FASTKD5	8.38248321672911e-32	0.289804461282834	0.06	0.035	1.83106963386231e-27	no change
4377	4377	ASB13	8.94600151036925e-32	0.249942748763892	0.059	0.034	1.95416456992506e-27	no change
4378	4378	MTMR1	8.98889408718306e-3	0.27928539709495	0.052	0.029	1.96353402440427e-2	no

			2	8			7	change
4379	4379	GRN	9.20239547119604e-32	-0.287507348	0.758	0.69	2.01017126672806e-27	no change
4380	4380	B3GLCT	9.27402050225236e-32	0.437615882691973	0.034	0.016	2.02581703851201e-27	no change
4381	4381	HOXA10-AS	9.39117275600899e-32	0.134232415293657	0.146	0.105	2.0514077768226e-27	no change
4382	4382	TMEM201	9.43725419532211e-32	0.499670753077475	0.034	0.016	2.06147380642616e-27	no change
4383	4383	CENPQ	9.46684963268436e-32	0.195098468919698	0.08	0.051	2.06793863376357e-27	no change
4384	4384	DGCR8	9.6734260942169e-32	0.221217269180055	0.056	0.032	2.11306319602074e-27	no change
4385	4385	DCAKD	1.03810092985478e-31	-0.108278258	0.134	0.093	2.26762767117479e-27	no change
4386	4386	CDCP1	1.05248649313037e-31	0.151927088404443	0.127	0.089	2.29905149559398e-27	no change
4387	4387	RAD51D	1.10114318917912e-31	0.356921583005811	0.042	0.022	2.40533718244286e-27	no change
4388	4388	PHF19	1.15994838355947e-31	0.59402604588638	0.033	0.015	2.5337912490473e-27	no change
4389	4389	BRIP1	1.17142051725948e-31	1.36168824029051	0.015	0.004	2.55885097790162e-27	up
4390	4390	PIGL	1.18181428659971e-31	0.197174025368103	0.061	0.036	2.58155512764841e-27	no change
4391	4391	MORC4	1.18245491453812e-3	0.15153115756063	0.071	0.043	2.58295451531708e-2	no

			1	6			7	change
4392	4392	CIB2	1.35266327940515e-31	0.384491726005311	0.058	0.034	2.95475766753262e-27	no change
4393	4393	NBAS	1.35876643430645e-31	0.289849310114223	0.059	0.034	2.96808939909901e-27	no change
4394	4394	EFNA1	1.39756337895568e-31	-0.473897449	0.485	0.474	3.0528374449908e-27	no change
4395	4395	ZNF92	1.48942088830044e-31	0.353095771880603	0.045	0.024	3.25349098840349e-27	no change
4396	4396	MRI1	1.50515126275962e-31	0.2223976553335	0.068	0.041	3.28785241837212e-27	no change
4397	4397	MED23	1.60822961705826e-31	0.192822262186723	0.065	0.039	3.51301677550206e-27	no change
4398	4398	ZNF608	1.69367172394549e-31	0.255142559931264	0.049	0.027	3.69965651378653e-27	no change
4399	4399	CYR61	1.78548093114985e-31	-1.648443736	0.201	0.231	3.90020454600374e-27	down
4400	4400	POLE2	1.83030116052334e-31	0.920135997575777	0.022	0.008	3.99810985504718e-27	no change
4401	4401	LINC01541	1.8381277562484e-31	0.436747279708556	0.028	0.012	4.01520627074901e-27	no change
4402	4402	FAM122A	1.93157017345338e-31	0.117260027263102	0.086	0.055	4.21932188689155e-27	no change
4403	4403	E2F5	2.0825018807416e-31	0.649124732166649	0.037	0.018	4.54901710829196e-27	no change
4404	4404	PNPLA6	2.10539604517234e-3	0.11903366597667	0.076	0.047	4.59902712107445e-2	no

			1	4			7	change
4405	4405	PRIM2	2.20340715941322e-31	0.163788069637432	0.06	0.035	4.81312259902224e-27	no change
4406	4406	RP11-796E2.4	2.26117954024752e-31	-1.25289457	0.033	0.056	4.93932058771668e-27	down
4407	4407	TACO1	2.27552025520736e-31	0.131205356817155	0.136	0.096	4.97064644547496e-27	no change
4408	4408	GEMIN2	2.28335113880958e-31	0.134174676888922	0.074	0.046	4.98775222761565e-27	no change
4409	4409	ACAD10	2.3898056516351e-31	0.326303456752232	0.051	0.028	5.22029146543172e-27	no change
4410	4410	HSD17B1	2.40498645728516e-31	0.310861693450931	0.073	0.045	5.25345241729371e-27	no change
4411	4411	HIST1H2AE	2.57762338161102e-31	0.222275875903379	0.086	0.056	5.63056051479111e-27	no change
4412	4412	AXDND1	2.59949435303909e-31	-1.183075759	0.03	0.052	5.67833546477859e-27	down
4413	4413	UHRF1BP1	2.61472564288214e-31	0.582400647381655	0.034	0.016	5.71160669431175e-27	no change
4414	4414	RNF152	2.8601591201701e-31	0.148753013889821	0.071	0.043	6.24773158209956e-27	no change
4415	4415	TAOK2	2.92996975502213e-31	0.164613512306408	0.075	0.047	6.40022593287035e-27	no change
4416	4416	EHD3	3.11109810457384e-31	0.333353763119389	0.066	0.04	6.79588269963109e-27	no change
4417	4417	COX15	3.12428259712832e-3	0.20068819459776	0.084	0.054	6.8246829051671e-27	no

			1	5				change
4418	4418	HAGLROS	3.27771226791817e-31	0.15273908842433	0.119	0.082	7.15983467804045e-27	no change
4419	4419	ANKRD44	3.37323673178911e-31	0.20466030965653	0.05	0.028	7.36849831692013e-27	no change
4420	4420	FGFRL1	3.37436075691144e-31	0.398990264268687	0.038	0.019	7.37095363739735e-27	no change
4421	4421	GABPA	3.45809904554748e-31	0.169696648776439	0.092	0.061	7.55387155509391e-27	no change
4422	4422	WDHD1	3.54074109926395e-31	0.661803052856905	0.024	0.01	7.73439485723217e-27	no change
4423	4423	CLCA4	3.56031515201099e-31	0.104090774200567	0.236	0.182	7.7771524180528e-27	no change
4424	4424	AVL9	3.61675336970231e-31	0.115169507247165	0.129	0.091	7.90043606077773e-27	no change
4425	4425	SOCS7	3.89789049021047e-31	0.425311548183151	0.046	0.025	8.51455198681574e-27	no change
4426	4426	SETD5	3.91611929614784e-31	-0.110398654	0.332	0.255	8.55437099050534e-27	no change
4427	4427	CFHR3	3.94670384338841e-31	0.515112571204749	0.022	0.008	8.62117987549764e-27	no change
4428	4428	LDOC1L	4.15883566846622e-31	0.222061026338602	0.047	0.025	9.0845606341976e-27	no change
4429	4429	CDK19	4.57319009690968e-31	0.156386127575088	0.086	0.055	9.98967644768951e-27	no change
4430	4430	TDP1	4.6176659359076e-31	0.28321476754015	0.058	0.034	1.00868294703966e-2	no

				5			6	change
4431	4431	GNG11	4.64569597126269e-31	-1.53309049	0.03	0.052	1.01480582796262e-26	down
4432	4432	PGAM5	4.97139420352535e-31	0.320120491858345	0.061	0.036	1.08595134981808e-26	no change
4433	4433	MPI	5.46413527783996e-31	0.178308368793419	0.099	0.066	1.19358571009136e-26	no change
4434	4434	ANKRD49	5.49912965658182e-31	0.113504241900156	0.114	0.078	1.20122988218373e-26	no change
4435	4435	ANKRD26	5.54348576167166e-31	0.106594834727192	0.072	0.044	1.21091902977956e-26	no change
4436	4436	COA7	5.74523977348626e-31	0.233786949004999	0.069	0.042	1.25499017612034e-26	no change
4437	4437	SMG6	5.74771827476544e-31	0.304828745073305	0.061	0.036	1.25553157993976e-26	no change
4438	4438	FOXN1	6.03256824035994e-31	-1.076527326	0.037	0.06	1.31775420642423e-26	down
4439	4439	ING5	6.070243504109e-31	0.103991182221006	0.089	0.058	1.32598399103757e-26	no change
4440	4440	CARD10	6.10952218994474e-31	0.718434371142942	0.024	0.01	1.33456402717153e-26	no change
4441	4441	ARHGAP11B	6.15922130863999e-31	0.71032528047521	0.026	0.011	1.34542030265932e-26	no change
4442	4442	BRAP	6.19669134244833e-31	0.189076026638809	0.081	0.052	1.35360525684441e-26	no change
4443	4443	POLR1B	6.39075120112088e-3	0.45141365306999	0.038	0.019	1.39599569237284e-2	no

			1	5			6	change
4444	4444	PTGFRN	6.42332002891535e-31	0.131653413785169	0.086	0.055	1.40311002711627e-26	no change
4445	4445	ACTR3B	6.95928834807911e-31	0.41103206753255	0.048	0.026	1.5201869467544e-26	no change
4446	4446	GYPC	7.00615440722725e-31	-1.225884209	0.044	0.069	1.53042436871472e-26	down
4447	4447	CHAC2	7.66639195029932e-31	0.401222724076787	0.041	0.022	1.67464665762338e-26	no change
4448	4448	LMF2	7.73692833547715e-31	-0.119847197	0.154	0.109	1.69005462560163e-26	no change
4449	4449	CRISPLD2	8.23224920930828e-31	-1.090201418	0.055	0.082	1.7982525172813e-26	down
4450	4450	RAPGEF6	8.23904395065822e-31	0.235378068503334	0.046	0.025	1.79973676058178e-26	no change
4451	4451	RP11-977G19.11	8.36774990720279e-31	0.142058757020088	0.109	0.075	1.82785128972938e-26	no change
4452	4452	SLC2A9	8.55793556150332e-31	0.288802483695047	0.054	0.031	1.86939544405479e-26	no change
4453	4453	SPC24	8.7222361012014e-31	0.23552330949445	0.034	0.016	1.90528525394643e-26	no change
4454	4454	SENP1	9.32707193107709e-31	0.304373943808532	0.052	0.03	2.03740559262448e-26	no change
4455	4455	MTDH	9.82108412865854e-31	-0.119585835	0.735	0.583	2.14531761706417e-26	no change
4456	4456	KLHL12	9.98104406372996e-3	0.16694099733961	0.096	0.064	2.18025926528117e-2	no

			1	3			6	change
4457	4457	OGFOD2	1.0301170261595e-30	0.139243714956757	0.096	0.063	2.25018763194282e-26	no change
4458	4458	SLIT2	1.06505970365974e-30	-1.702394862	0.015	0.031	2.32651641667433e-26	down
4459	4459	TBC1D13	1.07935439549051e-30	0.570945818496337	0.032	0.015	2.35774174150947e-26	no change
4460	4460	FBXO42	1.11989524574304e-30	0.166062258081054	0.075	0.047	2.4462991748011e-26	no change
4461	4461	MAP2K5	1.27770824394901e-30	0.25609940968529	0.064	0.039	2.79102588808223e-26	no change
4462	4462	RBM15	1.35854584317154e-30	0.225954860652266	0.053	0.03	2.96760753982391e-26	no change
4463	4463	DNAJC25	1.37474188104418e-30	0.1144783041067	0.083	0.054	3.0029861649529e-26	no change
4464	4464	LCK	1.40202247903363e-30	0.789150559882334	0.027	0.011	3.06257790320107e-26	no change
4465	4465	FOXP4-AS1	1.40530192075426e-30	1.25561074653489	0.013	0.003	3.0697415156956e-26	up
4466	4466	TTK	1.48655063632014e-30	1.11916824628477	0.015	0.004	3.24722120997771e-26	up
4467	4467	MTX3	1.51227596401929e-30	0.268918808263252	0.048	0.027	3.30341561580373e-26	no change
4468	4468	ARMC6	1.52137726722401e-30	0.115570382100337	0.089	0.058	3.32329650252412e-26	no change
4469	4469	TCF4	1.52198100081641e-3	-0.41783884	0.108	0.071	3.32461529818337e-2	no

			0				6 change
4470	4470	TPT1-AS1	1.58651090589642e-30	0.119432861635818	0.104	0.071	3.46557442284014e-26	no change
4471	4471	NAA40	1.62942441257677e-30	0.378817873297925	0.052	0.03	3.55931468683269e-26	no change
4472	4472	DUSP16	1.66005212048018e-30	0.161860855923152	0.115	0.079	3.6262178519769e-26	no change
4473	4473	DLG5	1.75178289741794e-30	0.146701372576498	0.115	0.079	3.82659456111976e-26	no change
4474	4474	PRPF40B	1.76896111349716e-30	0.207052042510962	0.08	0.051	3.8641186563232e-26	no change
4475	4475	ERC1	1.81418011059086e-30	0.142559714659213	0.07	0.043	3.96289503357468e-26	no change
4476	4476	TAF6L	1.83587039392589e-30	0.199262969277686	0.068	0.041	4.01027528849172e-26	no change
4477	4477	TBC1D14	1.8366848972489e-30	0.329207426128639	0.054	0.031	4.01205448955049e-26	no change
4478	4478	KIZ	1.87477692959338e-30	0.105445898906308	0.228	0.174	4.09526272500378e-26	no change
4479	4479	DMC1	1.92409518311978e-30	1.22573818319922	0.013	0.003	4.20299351800684e-26	up
4480	4480	SND1-IT1	1.93031268902751e-30	0.115340536564226	0.082	0.052	4.2165750379117e-26	no change
4481	4481	TNFRSF11B	1.98784243626734e-30	2.10541295110477	0.011	0.002	4.34224301778238e-26	up
4482	4482	TSC1	2.14680834706259e-3	0.45127613375710	0.043	0.023	4.68948815332353e-2	no

			0	7			6	change
4483	4483	FOXRED1	2.15689929196162e-30	0.205589651829758	0.064	0.038	4.71153081336097e-26	no change
4484	4484	ZNF462	2.32114941931011e-30	-0.10383971	0.086	0.056	5.07031879154101e-26	no change
4485	4485	FAM160A2	2.33934933933496e-30	0.108748535525069	0.089	0.058	5.11007469684328e-26	no change
4486	4486	DPY19L3	2.42247605584997e-30	0.215479110214864	0.054	0.031	5.29165669639867e-26	no change
4487	4487	FAM111B	2.45036722083615e-30	1.03668370881235	0.019	0.007	5.35258215719448e-26	up
4488	4488	ARHGEF39	2.48017550211793e-30	1.07891771744369	0.017	0.005	5.4176953668264e-26	up
4489	4489	UBXN2B	2.52321634852417e-30	0.130370625805641	0.101	0.068	5.5117137917162e-26	no change
4490	4490	SLC25A12	2.75622056492608e-30	0.245915015799146	0.055	0.032	6.02068820202454e-26	no change
4491	4491	AC015849.16	2.81405073251931e-30	-3.175741139	0.002	0.011	6.14701242011517e-26	down
4492	4492	PPP2R5C	2.90669708551995e-30	-0.114732585	0.303	0.232	6.34938911360979e-26	no change
4493	4493	GAS2	2.95806475666489e-30	0.792031550267846	0.024	0.01	6.46159665445878e-26	no change
4494	4494	KIAA1549	3.06948891709342e-30	0.639670358829711	0.027	0.011	6.70499159049886e-26	no change
4495	4495	DHX37	3.13538126251644e-3	0.38603714322070	0.042	0.022	6.84892682984091e-2	no

			0	3			6	change
4496	4496	FAM72D	3.24180926326966e-30	0.732880428840799	0.015	0.004	7.08140815468624e-26	no change
4497	4497	DDR2	3.47663514480425e-30	-1.405348038	0.029	0.05	7.5943618103104e-26	down
4498	4498	FBXO33	3.48765265825065e-30	0.114476076902469	0.105	0.071	7.61842846668272e-26	no change
4499	4499	SYCP2	3.58227811592298e-30	0.716620520497507	0.024	0.01	7.82512831642215e-26	no change
4500	4500	PAQR4	3.72268894192975e-30	0.609759897834877	0.04	0.021	8.13184172475135e-26	no change
4501	4501	LINC00324	3.73374259332081e-30	0.310753410230348	0.044	0.024	8.15598732084999e-26	no change
4502	4502	SRP14-AS1	3.90038751314655e-30	0.317151990248075	0.052	0.029	8.52000648371732e-26	no change
4503	4503	TXLNA	3.94935123857235e-30	0.124815644713507	0.095	0.064	8.62696284553745e-26	no change
4504	4504	VARS2	4.08138238208987e-30	0.275262397324747	0.051	0.029	8.91537167543711e-26	no change
4505	4505	RAB34	4.11912175165441e-30	-0.468849982	0.352	0.372	8.99780955431389e-26	no change
4506	4506	KATNB1	4.14592554903586e-30	0.205951858176522	0.066	0.041	9.05635976931392e-26	no change
4507	4507	RP1-60O19.1	4.20412695850451e-30	-1.999106924	0.006	0.018	9.18349492815724e-26	down
4508	4508	ICA1L	4.36329887499514e-3	0.24294546293820	0.048	0.027	9.53119006253939e-2	no

			0	8			6	change
4509	4509	CADM3	4.47319010118534e-30	-1.844711929	0.014	0.031	9.77123645702926e-26	down
4510	4510	COG8	4.65096833071608e-30	0.2026819520266	0.06	0.036	1.01595752216162e-25	no change
4511	4511	CD74	4.66793925880418e-30	-0.626210913	0.784	0.721	1.01966465169318e-25	no change
4512	4512	ORC5	4.90226326640452e-30	0.260395134745919	0.055	0.032	1.0708503879134e-25	no change
4513	4513	GATAD2B	4.9736520904008e-30	0.14806560577353	0.095	0.063	1.08644456262715e-25	no change
4514	4514	HJURP	5.07947360441973e-30	1.0156890925821	0.019	0.007	1.10956021414945e-25	up
4515	4515	CPVL	5.10815145466346e-30	-1.277124696	0.031	0.052	1.11582460375669e-25	down
4516	4516	SMPD4	5.23825535028536e-30	0.174258432130748	0.074	0.047	1.14424449871633e-25	no change
4517	4517	PRMT3	5.27435692321543e-30	0.192193743106329	0.052	0.03	1.15213052630718e-25	no change
4518	4518	DNA2	5.45340357831832e-30	1.01724227509016	0.02	0.007	1.19124147764785e-25	up
4519	4519	SARS2	5.90309286135702e-30	0.218332037736535	0.06	0.035	1.28947160463483e-25	no change
4520	4520	AIFM2	5.95910310494107e-30	0.157880157360839	0.095	0.064	1.30170648224333e-25	no change
4521	4521	ABCF3	6.06025266089685e-3	0.12049117644455	0.103	0.07	1.32380159124631e-2	no

			0	6			5	change
4522	4522	PPP2R5D	6.0608418741482e-30	0.344749426513864	0.053	0.031	1.32393029898893e-25	no change
4523	4523	SKIV2L	6.1701696905053e-30	0.138630108193875	0.079	0.051	1.34781186719398e-25	no change
4524	4524	PLXNA2	6.17891941433418e-30	0.104242345834065	0.105	0.071	1.34972315686716e-25	no change
4525	4525	F2RL3	6.20897082720608e-30	1.1868002210695	0.015	0.004	1.3562875874949e-25	up
4526	4526	PP7080	6.41280249062514e-30	0.289696905148122	0.081	0.052	1.40081257605216e-25	no change
4527	4527	TSTD2	6.61700789924106e-30	0.310531256652158	0.04	0.021	1.44541920551022e-25	no change
4528	4528	WDR76	6.66768456883538e-30	0.364171845390723	0.034	0.016	1.4564890172164e-25	no change
4529	4529	VPS33A	7.07783622072077e-30	0.233817276541451	0.068	0.042	1.54608254405425e-25	no change
4530	4530	CAMKK2	7.25897133991648e-30	0.315065600728246	0.063	0.038	1.58564969949136e-25	no change
4531	4531	HMOX1	8.05740227757982e-30	-0.995066415	0.095	0.126	1.76005895351454e-25	no change
4532	4532	RNMTL1	8.16860580484038e-30	0.198724740662574	0.063	0.038	1.78435025200933e-25	no change
4533	4533	CCDC88C	8.64134855553906e-30	0.111473368450082	0.071	0.044	1.88761617847195e-25	no change
4534	4534	BIVM	8.66878071386e-30	0.15562525924192	0.074	0.047	1.89360845913558e-2	no

							5	change
4535	4535	MAP3K4	8.77038334609632e-30	0.171919290269456	0.069	0.042	1.91580253812128e-25	no change
4536	4536	CTSW	1.00783767957188e-29	-1.011838673	0.036	0.059	2.20152062725682e-25	down
4537	4537	PNMT	1.00870806835028e-29	0.32339172643267	0.038	0.02	2.20342190450434e-25	no change
4538	4538	TYW1B	1.02941194270939e-29	0.694789530891129	0.022	0.009	2.24864744765439e-25	no change
4539	4539	FRMD3	1.03994984575889e-29	0.318202280697935	0.043	0.023	2.27166644307572e-25	no change
4540	4540	BICC1	1.04262275592856e-29	-1.645979632	0.02	0.038	2.27750514805034e-25	down
4541	4541	ERCC5	1.05562394379362e-29	0.130015363778528	0.068	0.042	2.30590494282277e-25	no change
4542	4542	KIAA1257	1.0840005111901e-29	0.964433524238499	0.018	0.006	2.36789071664365e-25	no change
4543	4543	SMKR1	1.12607881072939e-29	0.466076584087464	0.031	0.014	2.45980655415728e-25	no change
4544	4544	KLC2	1.20208409087498e-29	0.21484674235571	0.086	0.057	2.62583248810732e-25	no change
4545	4545	MIR181A2HG	1.27159877401994e-29	0.765277776658245	0.023	0.009	2.77768036196915e-25	no change
4546	4546	PEX1	1.30946686212087e-29	0.18999879885131	0.058	0.034	2.86039941361684e-25	no change
4547	4547	CCDC138	1.37202495539365e-2	0.46609564166200	0.029	0.013	2.99705131256188e-2	no

			9	1			5	change
4548	4548	SRD5A1	1.40085086038829e-29	0.288222067680149	0.073	0.046	3.06001861943218e-25	no change
4549	4549	CYP4V2	1.41862234580079e-29	0.231814988936835	0.076	0.048	3.09883865216726e-25	no change
4550	4550	KLC4	1.48053669760041e-29	0.214618654685359	0.07	0.043	3.23408436223834e-25	no change
4551	4551	MPND	1.50806647663419e-29	0.160981026063793	0.076	0.049	3.29422041155972e-25	no change
4552	4552	AC093388.3	1.51007401887094e-29	0.87918756208676	0.024	0.01	3.29860568682168e-25	no change
4553	4553	FHL1	1.52792868935062e-29	-1.571028518	0.033	0.054	3.33760742901749e-25	down
4554	4554	VPS54	1.56094412829758e-29	0.18798548159422	0.072	0.045	3.40972635385324e-25	no change
4555	4555	IQSEC1	1.63368154115025e-29	0.267802502889425	0.062	0.037	3.56861395848862e-25	no change
4556	4556	ZNF691	1.64073889926425e-29	0.580065919835564	0.037	0.019	3.58403005155283e-25	no change
4557	4557	PPP1R26	1.66715666399225e-29	0.408979938200623	0.039	0.02	3.64173701682467e-25	no change
4558	4558	NAPEPLD	1.74861543643436e-29	0.103199200934228	0.108	0.074	3.81967555934721e-25	no change
4559	4559	MCAT	1.78509001472096e-29	0.118360859472313	0.098	0.066	3.89935062815647e-25	no change
4560	4560	PTPN9	1.89933330739606e-2	0.11831339851373	0.08	0.052	4.14890367667594e-2	no

			9				5	change
4561	4561	PHF12	1.92652163810249e-29	0.140749499552925	0.084	0.055	4.20829386627107e-25	no change
4562	4562	TRAPPC8	1.93563783662049e-29	0.142361210361576	0.095	0.064	4.22820729031379e-25	no change
4563	4563	FAM73B	1.97288922317186e-29	0.33751067308374	0.049	0.027	4.30957921909662e-25	no change
4564	4564	RP11-797H7.5	1.98075053105971e-29	-0.910702751	0.033	0.055	4.32675146004683e-25	no change
4565	4565	LRR1	2.01584937254976e-29	0.340878579769937	0.054	0.032	4.40342136939769e-25	no change
4566	4566	LEMD2	2.02523053588999e-29	0.114540076312589	0.105	0.072	4.4239135825981e-25	no change
4567	4567	APBA2	2.02646068192073e-29	0.804636779367651	0.017	0.006	4.42660071358764e-25	no change
4568	4568	ZC3H12A	2.20327575942939e-29	-0.639770306	0.429	0.429	4.81283556889756e-25	no change
4569	4569	RPUSD4	2.21960140861484e-29	0.164987181904839	0.067	0.042	4.84849731697826e-25	no change
4570	4570	ULK2	2.2253824311609e-29	0.249690558430531	0.045	0.025	4.86112538262787e-25	no change
4571	4571	EMP1	2.27862364677294e-29	-0.355206948	0.335	0.377	4.97742549401081e-25	no change
4572	4572	ENPP4	2.43616027218231e-29	0.389410722696353	0.048	0.027	5.32154849855503e-25	no change
4573	4573	RP11-385F7.1	2.44432041445516e-2	0.15604173219319	0.116	0.082	5.33937351333585e-2	no

			9				5	change
4574	4574	LGI1	2.54456267512669e-29	-2.001164198	0.004	0.015	5.55834270754674e-25	down
4575	4575	C17orf89	2.66702947082519e-29	0.112388601890331	0.686	0.567	5.82585917607054e-25	no change
4576	4576	AKAP10	2.68637334323954e-29	0.200929543503541	0.079	0.051	5.86811393097245e-25	no change
4577	4577	RP5-1042K10.10	2.96157210850331e-29	0.569550481245546	0.027	0.012	6.46925811381463e-25	no change
4578	4578	FAM72C	2.97527907514769e-29	1.04242798643556	0.013	0.003	6.4991996117526e-25	up
4579	4579	PAPOLG	3.02008734186932e-29	0.211660415372187	0.055	0.032	6.59707878957934e-25	no change
4580	4580	GOLGA1	3.15667388480455e-29	0.378283518263728	0.051	0.029	6.89543843396705e-25	no change
4581	4581	CYP1A1	3.17400657620256e-29	-1.282854439	0.058	0.084	6.93329996505687e-25	down
4582	4582	ZXDC	3.22545526200348e-29	0.372577357066572	0.041	0.022	7.0456844743204e-25	no change
4583	4583	MBNL3	3.27605717612759e-29	0.284479016302995	0.06	0.036	7.15621929553312e-25	no change
4584	4584	PFAS	3.32854235184703e-29	0.756667441880649	0.022	0.009	7.27086791337466e-25	no change
4585	4585	AC073284.4	3.34599606288358e-29	-1.70111928	0.01	0.024	7.3089937997629e-25	down
4586	4586	PDE4A	3.49372686664649e-2	0.16793241211771	0.052	0.03	7.63169696750259e-2	no

			9	3			5	change
4587	4587	PAK6	3.64525504041064e-29	0.222189572630783	0.077	0.049	7.962695110273e-25	no change
4588	4588	OXCT1	3.77479825982052e-29	0.604982684253075	0.028	0.013	8.24566931875195e-25	no change
4589	4589	CD248	3.8358926148771e-29	-1.858729933	0.015	0.031	8.37912382793754e-25	down
4590	4590	CTD-2147F2.1	3.95669590876016e-29	0.339154468923787	0.045	0.025	8.6430065430957e-25	no change
4591	4591	ULK1	3.98000545118572e-29	0.244119946899799	0.083	0.054	8.69392390757009e-25	no change
4592	4592	MOV10L1	4.00776800209915e-29	1.25049656606277	0.014	0.004	8.75456842378538e-25	up
4593	4593	AMPH	4.04283371021831e-29	-1.310261883	0.011	0.026	8.83116595660088e-25	down
4594	4594	STOX2	4.2145440269333e-29	0.349958995051968	0.037	0.019	9.20624997243311e-25	no change
4595	4595	MDC1	4.39460365355094e-29	0.208118110695325	0.04	0.021	9.59957222081668e-25	no change
4596	4596	CD59	4.47116297032965e-29	-0.257248528	0.812	0.751	9.76680839238808e-25	no change
4597	4597	ZNF135	4.5125969865138e-29	0.626967324952571	0.023	0.009	9.85731685734074e-25	no change
4598	4598	ANKRD42	4.60333247939323e-29	0.222274846228403	0.058	0.035	1.00555194679866e-24	no change
4599	4599	TOE1	4.64945357639824e-2	0.39587590905689	0.041	0.022	1.01562663922843e-2	no

			9	8			4	change
4600	4600	TIMM22	4.92288178388569e-29	0.116819760937143	0.109	0.075	1.07535429687199e-24	no change
4601	4601	ADAT2	5.35466645022552e-29	0.303616516917822	0.039	0.02	1.16967333938726e-24	no change
4602	4602	ZFAND5	5.39135092970548e-29	-0.110536077	0.645	0.522	1.17768669708486e-24	no change
4603	4603	CLEC3B	5.45867354611923e-29	-2.200478113	0.009	0.022	1.19239264941428e-24	down
4604	4604	FCGRT	5.73443954711993e-29	-0.584063591	0.492	0.47	1.25263097467288e-24	no change
4605	4605	OPN3	5.77154376051301e-29	0.131719259613663	0.144	0.105	1.26073601904646e-24	no change
4606	4606	VSNL1	6.48973752926336e-29	0.685086420800144	0.019	0.007	1.41761826589229e-24	no change
4607	4607	PDE7B	6.53878255630665e-29	0.416991707793505	0.037	0.019	1.42833166159962e-24	no change
4608	4608	QSOX2	6.86449612090817e-29	0.821177575370367	0.021	0.008	1.49948053265118e-24	no change
4609	4609	DGCR2	6.9944679395055e-29	0.102221625054924	0.131	0.093	1.52787157670558e-24	no change
4610	4610	RP5-862P8.2	7.16456042268039e-29	0.668590699106816	0.029	0.014	1.5650265787303e-24	no change
4611	4611	SMIM5	7.24243481828811e-29	-0.674654485	0.273	0.295	1.58203746170686e-24	no change
4612	4612	CADPS2	7.3596417052784e-29	0.14049700290789	0.054	0.032	1.60764013410101e-2	no

				7			4	change
4613	4613	CLEC2D	7.42975133108831e-29	0.240538848961539	0.073	0.047	1.62295488076293e-24	no change
4614	4614	TMCC1	7.60537849098115e-29	0.107888488917685	0.111	0.077	1.66131887756992e-24	no change
4615	4615	CDC6	7.9999242336234e-29	0.726786821998239	0.043	0.023	1.7475034495927e-24	no change
4616	4616	GLS2	8.02653018933858e-29	0.621416503591737	0.025	0.011	1.75331525455912e-24	no change
4617	4617	NARFL	8.10093523807907e-29	0.135735546872493	0.098	0.066	1.76956829340599e-24	no change
4618	4618	C14orf80	8.15240607630945e-29	0.121577830585245	0.062	0.038	1.78081158330904e-24	no change
4619	4619	GATA2	8.33838316922675e-29	-0.620699806	0.279	0.305	1.82143641948589e-24	no change
4620	4620	NBPF9	8.47084834382209e-29	0.306777680401823	0.035	0.017	1.8503721122245e-24	no change
4621	4621	TCF21	8.5191318046927e-29	-1.659179268	0.018	0.035	1.86091915141707e-24	down
4622	4622	EXO1	8.52035832127035e-29	1.41550388987458	0.011	0.002	1.8611870716983e-24	up
4623	4623	MYLK-AS1	8.65212604983773e-29	0.543222548757167	0.031	0.014	1.88997041432655e-24	no change
4624	4624	TRAF5	9.10325758831803e-29	0.252937997566675	0.062	0.038	1.98851558759219e-24	no change
4625	4625	STRN	1.01469699673544e-2	0.17024698582546	0.081	0.053	2.21650411966889e-2	no

			8				4	change
4626	4626	PTRF	1.01928663399747e-28	-1.198025589	0.058	0.083	2.22652972330408e-24	down
4627	4627	MKNK2	1.03676825647525e-28	-0.636602653	0.293	0.311	2.26471657944454e-24	no change
4628	4628	NOMO3	1.06057409464477e-28	0.656447330129206	0.022	0.009	2.31671805234203e-24	no change
4629	4629	KCNK2	1.06480419372226e-28	0.959381765089761	0.014	0.004	2.3259582807669e-24	no change
4630	4630	RNF223	1.07419529480663e-28	-0.787892685	0.116	0.148	2.34647220197561e-24	no change
4631	4631	TMEM233	1.08207360821659e-28	-2.219815256	0.009	0.023	2.36368158978831e-24	down
4632	4632	EBF1	1.09475044708337e-28	-1.873567449	0.014	0.03	2.39137287660891e-24	down
4633	4633	RP11-369C8.1	1.11371264041395e-28	-4.680866183	0.003	0.013	2.43279389172023e-24	down
4634	4634	SLC25A19	1.15220179733324e-28	0.186871119521741	0.077	0.049	2.51686960609472e-24	no change
4635	4635	SNX30	1.17734801380228e-28	0.312088156961101	0.04	0.021	2.5717990013497e-24	no change
4636	4636	CYP27A1	1.25559283945548e-28	-1.20593464	0.031	0.052	2.74271699850654e-24	down
4637	4637	PARP2	1.27135648770108e-28	0.200154987885911	0.051	0.029	2.77715111173423e-24	no change
4638	4638	RP11-696N14.1	1.27369644414938e-2	0.46294086588552	0.038	0.02	2.7822625125999e-24	no

			8	7				change
4639	4639	MICAL3	1.33546913109286e-28	0.277596048165644	0.047	0.026	2.91719876995925e-24	no change
4640	4640	LTBP4	1.37166521468705e-28	-1.374617793	0.089	0.117	2.99626549496238e-24	down
4641	4641	BCR	1.40252484997549e-28	0.280682797223935	0.06	0.036	3.06367528228646e-24	no change
4642	4642	CBL	1.41190682452976e-28	0.327894012051678	0.053	0.031	3.08416926750281e-24	no change
4643	4643	SERPINB13	1.46437558696701e-28	0.144558826069143	0.05	0.028	3.19878203217073e-24	no change
4644	4644	SQLE	1.47940814353517e-28	-0.516798762	0.343	0.364	3.23161914873822e-24	no change
4645	4645	AAR2	1.4912891103674e-28	0.148214419265778	0.07	0.044	3.25757193268654e-24	no change
4646	4646	C21orf91	1.57136008334436e-28	0.123027533196487	0.075	0.048	3.43247896605742e-24	no change
4647	4647	MYBBP1A	1.57488335499852e-28	0.239278825894804	0.055	0.032	3.44017520065877e-24	no change
4648	4648	RASL11B	1.60239974996144e-28	0.26634736215332	0.03	0.014	3.50028201381576e-24	no change
4649	4649	HOXB9	1.64656938779225e-28	0.770605636809462	0.025	0.011	3.59676617069339e-24	no change
4650	4650	LMBRD2	1.68418112914113e-28	0.200898112297251	0.089	0.06	3.67892525849588e-24	no change
4651	4651	CMB9-22P13.1	1.84122492697488e-2	-1.159389398	0.084	0.112	4.02197173048392e-2	down

			8				4	
4652	4652	MSTO1	2.06153062976121e-28	0.272440878121601	0.048	0.027	4.50320750765038e-24	no change
4653	4653	AIM2	2.18247959698616e-28	0.230013047585408	0.13	0.095	4.76740843165658e-24	no change
4654	4654	ALDH1B1	2.28609882624887e-28	0.367919422930012	0.04	0.021	4.99375427605803e-24	no change
4655	4655	HIST1H1E	2.3244565456959e-28	0.709718815358641	0.022	0.008	5.07754287841813e-24	no change
4656	4656	ATP13A1	2.3324382498193e-28	0.133071505618983	0.085	0.056	5.09497811290529e-24	no change
4657	4657	PPP1R21	2.33379527761276e-28	0.100450432285314	0.112	0.079	5.09794240441731e-24	no change
4658	4658	KIAA1468	2.3491885838653e-28	0.129216538534055	0.091	0.061	5.13156754259537e-24	no change
4659	4659	KLF8	2.41829705467689e-28	0.342494514647886	0.061	0.038	5.28252808623621e-24	no change
4660	4660	RDH12	2.78848816289425e-28	-1.600100262	0.008	0.02	6.09117354302621e-24	down
4661	4661	RRP36	2.84806806697242e-28	0.104252130637691	0.104	0.072	6.22131988549455e-24	no change
4662	4662	LINC00152	2.85639558433147e-28	-0.834840627	0.158	0.19	6.23951051441366e-24	no change
4663	4663	CDK2AP1	2.94941363202177e-28	0.11171634957822	0.111	0.078	6.44269913778836e-24	no change
4664	4664	TGM1	2.99285622529608e-2	-1.995540095	0.005	0.016	6.53759513853675e-2	down

			8				4	
4665	4665	E2F1	3.04463927902919e-28	0.798984668227287	0.033	0.016	6.65071004111137e-24	no change
4666	4666	SETD1B	3.11051800804657e-28	0.27090252009456	0.054	0.032	6.79461553677692e-24	no change
4667	4667	EPB41L4B	3.22677178293751e-28	0.406029982467627	0.055	0.033	7.04856028264871e-24	no change
4668	4668	MGAT1	3.33672454565191e-28	-0.356999616	0.498	0.49	7.28874109752203e-24	no change
4669	4669	NEMP2	3.43412826634403e-28	0.667307262220666	0.024	0.01	7.5015097850019e-24	no change
4670	4670	CTSA	3.47422221346444e-28	-0.53256933	0.353	0.367	7.58909100309172e-24	no change
4671	4671	DUOX2	3.810981951848e-28	-0.132634884	0.111	0.076	8.32470897561678e-24	no change
4672	4672	TRIM65	4.00023066432654e-28	0.4002046130498	0.033	0.016	8.7381038631549e-24	no change
4673	4673	TMBIM1	4.24139834980634e-28	-0.552686246	0.424	0.414	9.26491055531697e-24	no change
4674	4674	SLC5A3	4.27441637965517e-28	-0.164496237	0.206	0.153	9.33703513971875e-24	no change
4675	4675	ZNF169	4.29329957234469e-28	0.617567045090032	0.02	0.008	9.37828358582974e-24	no change
4676	4676	A2M	4.35615884911032e-28	-1.139492492	0.038	0.06	9.51559338999658e-24	down
4677	4677	GPATCH11	4.39623863223918e-2	0.24819969656410	0.05	0.029	9.60314366826327e-2	no

			8	6			4	change
4678	4678	ZER1	4.53733721200251e-28	0.286891433093562	0.041	0.022	9.91135940589829e-24	no change
4679	4679	PDGFRA	4.63245587388074e-28	-1.497515315	0.023	0.042	1.01191366109051e-23	down
4680	4680	RAPGEF1	4.75978331394501e-28	0.274076563016094	0.044	0.025	1.03972706709815e-23	no change
4681	4681	MIS12	5.07018421962321e-28	0.141437072859041	0.067	0.042	1.10753104093449e-23	no change
4682	4682	DNASE1	5.16822192515156e-28	0.344286365904493	0.051	0.03	1.12894639733011e-23	no change
4683	4683	ACOX3	5.19929384444936e-28	0.178072137781394	0.084	0.055	1.13573374738152e-23	no change
4684	4684	PROSER1	5.21387931140946e-28	0.264269148931843	0.07	0.045	1.13891979678428e-23	no change
4685	4685	ARRDC1-AS1	5.46590151524971e-28	0.156104098567234	0.072	0.046	1.19397152699115e-23	no change
4686	4686	REPS1	5.5510328738069e-28	0.103860345278914	0.092	0.062	1.21256762095438e-23	no change
4687	4687	PMM2	5.83535920142082e-28	0.144106233197555	0.065	0.041	1.27467586395836e-23	no change
4688	4688	ALG1	5.92635645693717e-28	0.179243094614729	0.069	0.044	1.29455330445336e-23	no change
4689	4689	THNSL1	6.38021811734903e-28	0.322161015424104	0.035	0.017	1.39369484555372e-23	no change
4690	4690	MCM3AP-AS1	6.45420405815574e-2	0.45610586821564	0.03	0.014	1.40985633446354e-2	no

			8				3	change
4691	4691	SREBF1	6.68843733404559e-28	-0.808337658	0.12	0.151	1.46102225124892e-23	no change
4692	4692	COPS8	6.75871177699758e-28	-0.123612462	0.31	0.238	1.47637300056735e-23	no change
4693	4693	C6orf106	6.88843472118444e-28	0.15461405815685	0.1	0.069	1.50470968049553e-23	no change
4694	4694	THAP10	7.05046494284143e-28	0.360167152110344	0.044	0.024	1.54010356211428e-23	no change
4695	4695	TXNRD3	7.18942095393761e-28	0.381051459672539	0.04	0.021	1.57045711317813e-23	no change
4696	4696	ATF7IP2	7.22039144573468e-28	0.116831401517223	0.317	0.257	1.57722230740628e-23	no change
4697	4697	TAF1A	7.91011939523658e-28	0.252751108977628	0.042	0.023	1.72788648069548e-23	no change
4698	4698	TAF5L	8.31215742370658e-28	0.16871556742591	0.058	0.035	1.81570766763447e-23	no change
4699	4699	LRRC69	8.5600358819612e-28	0.527258302609372	0.029	0.014	1.86985423805561e-23	no change
4700	4700	CD81	8.65961319977047e-28	-0.10855107	0.453	0.357	1.89160590735786e-23	no change
4701	4701	SUSD1	8.68858599670863e-28	0.371038750862647	0.047	0.027	1.89793472512103e-23	no change
4702	4702	TTL	8.73110793071976e-28	0.173687557467205	0.057	0.034	1.90722321638642e-23	no change
4703	4703	ARHGEF26	9.28013802395878e-2	0.17885529495659	0.046	0.026	2.02715334995356e-2	no

			8	7			3	change
4704	4704	RP11-734K2.4	9.31651380191027e-28	0.253331160504154	0.077	0.05	2.03509927488928e-23	no change
4705	4705	CCDC77	9.93413469602487e-28	0.528523986901719	0.032	0.016	2.17001238299967e-23	no change
4706	4706	LINC01136	1.05175371810677e-27	-0.872881186	0.03	0.051	2.29745082183243e-23	no change
4707	4707	FAM21A	1.05615216893821e-27	0.21363129012238	0.077	0.05	2.30705879782862e-23	no change
4708	4708	NCAPH2	1.11288300928672e-27	0.107927543335041	0.09	0.06	2.43098164548591e-23	no change
4709	4709	MTFP1	1.12155884401041e-27	0.31331065845519	0.079	0.052	2.44993313885633e-23	no change
4710	4710	FAM107A	1.12212082553841e-27	-1.440091871	0.021	0.038	2.45116073130611e-23	down
4711	4711	ASB3	1.12367561479168e-27	0.162593926058258	0.055	0.033	2.45455701295095e-23	no change
4712	4712	RP5-968D22.1	1.12401889381193e-27	1.46530516465409	0.011	0.002	2.45530687164279e-23	up
4713	4713	FECH	1.12583690854752e-27	0.207339836962599	0.081	0.053	2.4592781430312e-23	no change
4714	4714	RHEB	1.13373221805948e-27	-0.193692205	0.762	0.695	2.47652465712912e-23	no change
4715	4715	CREB5	1.1622147248352e-27	-1.278270035	0.035	0.056	2.53874184493001e-23	down
4716	4716	FAM83B	1.20506931147468e-2	0.25501029862529	0.057	0.035	2.63235340398528e-2	no

			7	3			3	change
4717	4717	SPNS2	1.20628445877102e-27	-1.767296015	0.005	0.015	2.63500777173942e-23	down
4718	4718	LCLAT1	1.23019055085975e-27	0.131695049326429	0.063	0.039	2.68722823929803e-23	no change
4719	4719	TMEM260	1.24470976651479e-27	0.148572609	0.054	0.032	2.7189440139749e-23	no change
4720	4720	C7orf26	1.28576393299248e-27	0.124970034309504	0.085	0.056	2.80862273522877e-23	no change
4721	4721	CRAMP1	1.29037991328924e-27	0.28710796215887	0.042	0.023	2.81870588258901e-23	no change
4722	4722	CRIP3	1.30552872898389e-27	-2.2700759	0.004	0.013	2.85179695559241e-23	down
4723	4723	CCNO	1.32292032473195e-27	0.107888491202216	0.099	0.068	2.88978715734448e-23	no change
4724	4724	PLEKHH2	1.32802614026439e-27	0.168582828099884	0.035	0.018	2.90094030079353e-23	no change
4725	4725	XK	1.34945024590153e-27	0.546154796937336	0.02	0.007	2.94773911714731e-23	no change
4726	4726	SRRM3	1.45599900802782e-27	0.4771278454375	0.036	0.019	3.18048423313597e-23	no change
4727	4727	ASPN	1.45955377093347e-27	-2.214559142	0.005	0.016	3.18824925722707e-23	down
4728	4728	PRDM4	1.70467981497476e-27	0.16233958676409	0.065	0.04	3.72370258783088e-23	no change
4729	4729	FASTKD3	1.72227085090412e-2	0.25358320720011	0.06	0.037	3.76212844671496e-2	no

			7	2			3	change
4730	4730	CTHRC1	1.73964049838413e-27	-1.577442517	0.056	0.081	3.80007070467029e-23	down
4731	4731	SELM	1.7912583394161e-27	-1.063904652	0.212	0.235	3.91282471662053e-23	down
4732	4732	TAPT1	1.79613496194233e-27	0.333595762910148	0.038	0.02	3.92347721086683e-23	no change
4733	4733	COL5A2	1.84377703361089e-27	-1.285707168	0.029	0.049	4.02754655221963e-23	down
4734	4734	GSAP	1.90482217751927e-27	0.130202062705801	0.071	0.045	4.16089356457309e-23	no change
4735	4735	CRYAB	1.97610587746869e-27	-1.202671575	0.08	0.109	4.31660567874261e-23	down
4736	4736	TRIM37	2.03898977795602e-27	0.233383940906605	0.084	0.056	4.45396927096712e-23	no change
4737	4737	UCK1	2.10884278788892e-27	0.139139493959966	0.086	0.058	4.60655618586455e-23	no change
4738	4738	TOR1B	2.12877203580791e-27	0.254733442463364	0.048	0.028	4.65008963501879e-23	no change
4739	4739	MEDAG	2.13791825877186e-27	-2.088459464	0.012	0.026	4.67006864446125e-23	down
4740	4740	POU2AF1	2.14198719159864e-27	0.42654801397022	0.037	0.019	4.67895682132807e-23	no change
4741	4741	TRRAP	2.15646630651472e-27	0.185925338021122	0.054	0.032	4.71058499995075e-23	no change
4742	4742	GBP4	2.42259338475016e-2	0.14356377770967	0.119	0.085	5.29191298964826e-2	no

			7	6			3	change
4743	4743	ZMYM3	2.42411632800589e-27	0.284926119174822	0.053	0.032	5.29523970689606e-23	no change
4744	4744	RP5-1159O4.1	2.48116248650243e-27	-0.82606058	0.075	0.103	5.41985133551591e-23	no change
4745	4745	FABP3	2.57510216517365e-27	-1.22620558	0.033	0.055	5.62505316960532e-23	down
4746	4746	CTA-276F8.1	2.65688875866489e-27	0.505396753591126	0.022	0.009	5.8037078044276e-23	no change
4747	4747	LINC01456	2.75790946290853e-27	0.95253040727378	0.014	0.004	6.02437743077738e-23	no change
4748	4748	RHOBTB1	2.88965609082323e-27	0.511261773710221	0.026	0.012	6.31216476479425e-23	no change
4749	4749	LAPTM5	3.11435309348947e-27	-0.198812255	0.1	0.069	6.80299289741839e-23	no change
4750	4750	CCNE2	3.30185087342948e-27	0.632260576268643	0.021	0.008	7.21256304791936e-23	no change
4751	4751	HIST1H2BJ	3.32765715251928e-27	0.707702557037562	0.057	0.035	7.26893428396311e-23	no change
4752	4752	TMEM94	3.45035621101606e-27	0.219454993811707	0.081	0.054	7.53695810734348e-23	no change
4753	4753	ZSCAN16	3.52719668742428e-27	0.297918821174672	0.056	0.033	7.70480844400961e-23	no change
4754	4754	ZNF317	3.66992245081784e-27	0.234758993946478	0.059	0.036	8.0165786015665e-23	no change
4755	4755	DSCC1	3.99596087253714e-2	0.59880824284295	0.02	0.008	8.72877692997013e-2	no

			7	3			3	change
4756	4756	PSD4	4.0032845339551e-27	0.180163456753096	0.081	0.054	8.74477473597153e-23	no change
4757	4757	PCNXL2	4.00532549414045e-27	0.223071485204155	0.064	0.04	8.7492330094004e-23	no change
4758	4758	TRNP1	4.19315299325102e-27	0.149431773509249	0.098	0.067	9.15952339845753e-23	no change
4759	4759	BTN3A1	4.2084470561457e-27	0.152430138715331	0.068	0.043	9.19293174944467e-23	no change
4760	4760	S1PR5	4.23048275175564e-27	0.349473400309821	0.037	0.019	9.24106652293501e-23	no change
4761	4761	CNR1	4.70617701942222e-27	1.79154040484182	0.01	0.002	1.02801730812259e-22	up
4762	4762	ZNF627	4.75308435039899e-27	0.314906022421092	0.039	0.021	1.03826374550115e-22	no change
4763	4763	FOXK2	4.84458719644987e-27	0.143053790334227	0.089	0.06	1.05825162719251e-22	no change
4764	4764	LIN54	4.85635718022825e-27	0.490495754062909	0.041	0.023	1.06082266244906e-22	no change
4765	4765	ZNF551	4.90095691779445e-27	0.476787487657087	0.03	0.014	1.07056502912302e-22	no change
4766	4766	LHFP	5.31486232041086e-27	-1.512902175	0.024	0.043	1.16097852527055e-22	down
4767	4767	KLHL5	5.34436610200257e-27	0.23945820247469	0.048	0.028	1.16742333132144e-22	no change
4768	4768	ERICH6-AS1	5.38416074399429e-2	0.45781708438164	0.036	0.019	1.17611607291811e-2	no

			7	2			2	change
4769	4769	TCF20	5.48498495487788e-27	0.191672377466726	0.066	0.041	1.19814011354353e-22	no change
4770	4770	NR4A3	5.69862761096993e-27	-0.180864198	0.145	0.105	1.24480821534027e-22	no change
4771	4771	TYK2	5.77317446377577e-27	0.11903392524606	0.081	0.053	1.26109222986718e-22	no change
4772	4772	LIMD1	5.78539310759457e-27	0.266754104033211	0.058	0.036	1.26376127042296e-22	no change
4773	4773	SCN9A	5.82081855928957e-27	0.895075820728891	0.018	0.007	1.27149960609121e-22	no change
4774	4774	PPM1F	5.8866382947254e-27	0.251528301766092	0.049	0.028	1.28587726909982e-22	no change
4775	4775	CHD1L	6.30959421683597e-27	0.177005361911795	0.067	0.043	1.37826776072565e-22	no change
4776	4776	STXBP4	6.48129043971448e-27	0.395080325607952	0.031	0.015	1.41577308365123e-22	no change
4777	4777	BTBD8	6.79002046372519e-27	0.557652269529547	0.026	0.011	1.48321207009613e-22	no change
4778	4778	GLB1L2	6.8220469809464e-27	1.17797392158518	0.015	0.005	1.49020794251793e-22	up
4779	4779	PYROXD1	7.16358257594947e-27	0.123847224923772	0.076	0.05	1.5648129778904e-22	no change
4780	4780	FOXF1	7.20749518953534e-27	-1.565996561	0.017	0.033	1.5744052492021e-22	down
4781	4781	DHRS11	7.34462642787856e-2	0.16252777703991	0.127	0.092	1.60436019690579e-2	no

			7	3			2	change
4782	4782	ALDOC	7.5263722408525e-27	-0.763999732	0.126	0.156	1.64406075229182e-22	no change
4783	4783	GORASP1	7.81261997093762e-27	0.143082867697227	0.082	0.054	1.70658870645161e-22	no change
4784	4784	HLA-DPB1	7.88418571307963e-27	-0.702273391	0.267	0.295	1.72222152716512e-22	no change
4785	4785	ZNF500	7.92451674080273e-27	0.42618103358784	0.035	0.018	1.73103143686095e-22	no change
4786	4786	ABAT	8.14490665336904e-27	0.134913604355975	0.107	0.075	1.77917340936193e-22	no change
4787	4787	TRABD2A	8.14894653925134e-27	0.555031137532287	0.02	0.008	1.78005588203406e-22	no change
4788	4788	PLXNA1	8.22157206031885e-27	0.214593827374926	0.054	0.032	1.79592020085605e-22	no change
4789	4789	RP11-268G12.1	8.80774167665035e-27	0.626629606364301	0.023	0.01	1.9239630918475e-22	no change
4790	4790	INPPL1	9.08805631190166e-27	0.166448923239971	0.075	0.049	1.9851950207718e-22	no change
4791	4791	ADCY6	9.39834124121474e-27	0.283449542875974	0.059	0.036	2.05297366073095e-22	no change
4792	4792	ITGA2B	9.60370901880641e-27	0.884693173726648	0.016	0.005	2.09783419806807e-22	no change
4793	4793	STRN3	1.06453745872165e-26	-0.170672387	0.19	0.142	2.32537562483158e-22	no change
4794	4794	PRDM1	1.06825874915065e-2	-0.136165572	0.179	0.133	2.33350441164468e-2	no

			6				2	change
4795	4795	AK1	1.08613554744523e-26	-0.216755587	0.221	0.167	2.37255448983936e-22	no change
4796	4796	SIPA1L1	1.13237358048076e-26	0.111055035251162	0.094	0.064	2.47355684920218e-22	no change
4797	4797	ID3	1.13667294038982e-26	-0.486552366	0.833	0.783	2.48294837098752e-22	no change
4798	4798	TLR3	1.14929982950776e-26	0.298869380534168	0.058	0.036	2.51053054757674e-22	no change
4799	4799	PCTP	1.15646485963539e-26	0.18481773744778	0.096	0.066	2.52618183938754e-22	no change
4800	4800	CHML	1.16739825180529e-26	0.321654018205738	0.049	0.029	2.55006474124347e-22	no change
4801	4801	RBM45	1.1791342323637e-26	0.232571259492624	0.052	0.031	2.57570081717527e-22	no change
4802	4802	LZTFL1	1.22184026388886e-26	0.155007021786576	0.071	0.046	2.66898787243883e-22	no change
4803	4803	KLHDC9	1.22381375058838e-26	0.185511909850686	0.102	0.071	2.67329875678525e-22	no change
4804	4804	SLC15A4	1.24423473909269e-26	0.146615759858482	0.055	0.033	2.71790636407407e-22	no change
4805	4805	CHAMP1	1.2570072962598e-26	0.113156315003013	0.062	0.039	2.7458067379499e-22	no change
4806	4806	TEAD4	1.27873197788574e-26	0.451428828369631	0.024	0.01	2.79326213249361e-22	no change
4807	4807	TCIRG1	1.2804303653108e-26	-0.121601949	0.161	0.118	2.79697208998491e-2	no

						2	change
4808	4808	ZFP64	1.3087888942335e-26	0.31218633699254	0.049	0.029	2.85891846056367e-22	no change
4809	4809	MOB1B	1.32291967630449e-26	0.183966654299972	0.057	0.035	2.88978574091953e-22	no change
4810	4810	KCTD18	1.34903836166687e-26	0.262583505491365	0.058	0.035	2.9468393972251e-22	no change
4811	4811	ZC3H12C	1.38991189895232e-26	0.26348559913346	0.061	0.038	3.03612355207145e-22	no change
4812	4812	RSAD2	1.42880207396551e-26	-1.063018952	0.044	0.066	3.12107525037026e-22	down
4813	4813	REV3L	1.49450956750184e-26	-0.175470083	0.112	0.078	3.26460669925103e-22	no change
4814	4814	SLC35E2	1.52090814890632e-26	1.09223335368706	0.017	0.006	3.32227176047096e-22	up
4815	4815	RP13-188A5.1	1.52107303720386e-26	0.122169145913964	0.061	0.038	3.32263194246811e-22	no change
4816	4816	DDX51	1.58216107894447e-26	0.258968010380157	0.048	0.027	3.4560726608463e-22	no change
4817	4817	INTS4	1.61363528508375e-26	0.141305534587746	0.052	0.031	3.52482491673695e-22	no change
4818	4818	PDK4	1.73462372902078e-26	-0.973633991	0.122	0.152	3.78911207367299e-22	no change
4819	4819	MEGF9	1.75876806337624e-26	0.166878188992112	0.066	0.042	3.84185295763907e-22	no change
4820	4820	DLEU2	1.7640050745408e-26	0.10379411765899	0.087	0.059	3.85329268482691e-2	no

				3			2	change
4821	4821	PXN-AS1	1.79687250856522e-26	0.133027814540341	0.072	0.047	3.92508830770987e-22	no change
4822	4822	CAPN2	1.81084619846663e-26	-0.20407428	0.254	0.192	3.9556124359305e-22	no change
4823	4823	CDH8	1.82375132303912e-26	0.328894013261306	0.03	0.015	3.98380239004666e-22	no change
4824	4824	TOP3A	1.91389859626493e-26	0.435794249053657	0.029	0.014	4.18072009368112e-22	no change
4825	4825	POLB	1.98443396441453e-26	0.130863374818686	0.099	0.069	4.3347975518671e-22	no change
4826	4826	COG1	1.98972527048918e-26	0.229039786364849	0.065	0.041	4.34635588085656e-22	no change
4827	4827	ACSS1	2.08143225070963e-26	0.105703529707283	0.095	0.065	4.54668060845011e-22	no change
4828	4828	IRX2	2.08931106538339e-26	0.869064617632438	0.013	0.004	4.56389109122349e-22	no change
4829	4829	CENPJ	2.09607944924314e-26	0.567444914989037	0.024	0.01	4.57867594892671e-22	no change
4830	4830	CCDC189	2.10143204060708e-26	0.267525626306112	0.052	0.031	4.5903681495021e-22	no change
4831	4831	KLHL29	2.15037814865457e-26	0.392734811056783	0.037	0.02	4.69728602792105e-22	no change
4832	4832	SCHLAP1	2.17954494021909e-26	0.253563833757443	0.095	0.066	4.76099796741459e-22	no change
4833	4833	BNIP1	2.18248981549834e-2	0.11286436549450	0.076	0.05	4.76743075297457e-2	no

			6	8			2	change
4834	4834	L3MBTL4	2.21156795727641e-26	0.744309092034246	0.021	0.008	4.83094904587459e-22	no change
4835	4835	CBR3	2.35566520728182e-26	-0.106061761	0.165	0.123	5.14571507878641e-22	no change
4836	4836	GEN1	2.49390431316725e-26	0.629550835651261	0.026	0.012	5.44768458168253e-22	no change
4837	4837	SPRR1A	2.51543476124075e-26	-1.173856349	0.016	0.032	5.49471569245429e-22	down
4838	4838	VCAN	2.55148489988298e-26	-1.344188917	0.035	0.056	5.57346361530437e-22	down
4839	4839	DNAJC14	2.61346802800642e-26	0.177159495957484	0.068	0.044	5.70885956037722e-22	no change
4840	4840	SNX19	2.69918707393275e-26	0.235152531590178	0.061	0.038	5.89610424429871e-22	no change
4841	4841	FANCB	2.80759828835233e-26	0.675060685667796	0.021	0.009	6.13291770107683e-22	no change
4842	4842	CCDC112	2.87202840882084e-26	0.158700716910053	0.09	0.062	6.27365885622824e-22	no change
4843	4843	ARAP3	2.88715323525956e-26	0.179152495597145	0.051	0.03	6.30669752710099e-22	no change
4844	4844	ZBED4	2.89900870370243e-26	0.268913629287256	0.04	0.022	6.33259461236758e-22	no change
4845	4845	DBP	2.97668859238214e-26	-0.964799872	0.042	0.064	6.50227856119954e-22	no change
4846	4846	PHKA2	3.0455489378918e-26	0.13443159128079	0.045	0.026	6.65269709993085e-2	no

				1			2	change
4847	4847	AACS	3.11892140529655e-26	0.152700684557795	0.062	0.039	6.81297191772978e-22	no change
4848	4848	HAUS3	3.2250104504546e-26	0.244561255363466	0.043	0.024	7.04471282797303e-22	no change
4849	4849	PLD2	3.26519272152505e-26	0.212029373679986	0.051	0.031	7.13248698089931e-22	no change
4850	4850	ETNK2	3.29544479836342e-26	0.125825997179966	0.067	0.042	7.19856961754506e-22	no change
4851	4851	HOXA4	3.44465525218245e-26	0.365415879350786	0.036	0.019	7.52450493286734e-22	no change
4852	4852	ZNF429	3.47226941300364e-26	0.323765588122479	0.056	0.034	7.58482530576515e-22	no change
4853	4853	ENOX2	3.54061238831953e-26	0.121698813421423	0.065	0.041	7.73411370104518e-22	no change
4854	4854	ZDHHC17	3.56055451272227e-26	0.138429052410014	0.058	0.036	7.77767527759053e-22	no change
4855	4855	TWIST2	3.57544775283118e-26	-1.528160077	0.018	0.034	7.81020807128443e-22	down
4856	4856	PRKACA	3.64893060100935e-26	0.111307223727207	0.086	0.058	7.97072400484483e-22	no change
4857	4857	ZNF57	3.6724731072871e-26	0.156586203872921	0.048	0.028	8.02215025555795e-22	no change
4858	4858	MLYCD	3.73203904081968e-26	0.137121431014168	0.074	0.048	8.15226608076651e-22	no change
4859	4859	MAP2K3	3.78276325926515e-2	-0.534385877	0.349	0.36	8.2630680635388e-22	no

			6					change
4860	4860	TNFSF15	4.0902175825344e-26	0.276530817826378	0.052	0.031	8.93467128728814e-22	no change
4861	4861	POLA1	4.1014746494706e-26	0.408080442249465	0.028	0.013	8.95926122430357e-22	no change
4862	4862	ASB7	4.11646313951361e-26	0.446380018707396	0.033	0.017	8.99200208195352e-22	no change
4863	4863	POU6F2	4.25050224446715e-26	0.461810674432872	0.022	0.009	9.28479710281405e-22	no change
4864	4864	FILIP1L	4.26220338451497e-26	-1.278245935	0.027	0.046	9.3103570731345e-22	down
4865	4865	FAM120B	4.54905046974402e-26	0.201866380615402	0.057	0.035	9.93694584610883e-22	no change
4866	4866	TBCK	4.57002734624662e-26	0.127621172955818	0.068	0.044	9.98276773514111e-22	no change
4867	4867	RP11-298J20.3	4.59986373085853e-26	-1.449149226	0.015	0.029	1.00479423336874e-21	down
4868	4868	TATDN2	4.67320869691553e-26	0.26608192540374	0.048	0.028	1.02081570775423e-21	no change
4869	4869	ZCCHC10	4.68180752366816e-26	-0.104499984	0.323	0.25	1.02269403547007e-21	no change
4870	4870	RP11-390F4.3	4.83425371110324e-26	0.261399898904454	0.059	0.036	1.05599438065339e-21	no change
4871	4871	RP11-1398P2.1	4.91198402625507e-26	0.294203268309848	0.037	0.02	1.07297379069516e-21	no change
4872	4872	ZNF445	5.18351897388093e-2	0.30860325269125	0.039	0.021	1.13228788465455e-2	no

			6	6			1	change
4873	4873	CTC-498J12.1	5.21012119824125e-26	0.716085438192375	0.026	0.012	1.13809887454382e-21	no change
4874	4874	ZNF586	5.4114123379135e-26	0.294535191603394	0.045	0.025	1.18206891109383e-21	no change
4875	4875	RALGPS1	5.45401451457892e-26	0.230156007049213	0.05	0.029	1.19137493056462e-21	no change
4876	4876	GDF11	5.52725976392169e-26	-0.158425972	0.078	0.051	1.20737462283105e-21	no change
4877	4877	BEND5	5.55810172420797e-26	0.285849681872037	0.058	0.036	1.21411174063599e-21	no change
4878	4878	ZBED8	5.83411595842764e-26	0.894899039217432	0.017	0.006	1.27440428995893e-21	no change
4879	4879	DYNC2H1	6.00404783147291e-26	0.143162883101102	0.048	0.028	1.31152420830694e-21	no change
4880	4880	SNCAIP	6.12121984440314e-26	0.791172618595994	0.016	0.006	1.33711926281142e-21	no change
4881	4881	CORO7	6.13117830104894e-26	0.161310673191907	0.056	0.034	1.33929458808113e-21	no change
4882	4882	SPATA6L	6.31124728123956e-26	0.218717986038071	0.044	0.025	1.37862885611397e-21	no change
4883	4883	NEB	6.38118171511332e-26	1.21631927137877	0.013	0.004	1.39390533384935e-21	up
4884	4884	CDKN1C	7.27322569567067e-26	-0.141446403	0.26	0.202	1.5887634209623e-21	no change
4885	4885	PXYLP1	7.3135512066544e-26	0.26999831704988	0.061	0.038	1.59757212558159e-2	no

							1	change
4886	4886	TBC1D15	7.403678402554e-26	-0.140262069	0.14	0.102	1.6172595102539e-21	no change
4887	4887	CCNL1	7.50031917608372e-26	-0.287787816	0.693	0.642	1.63836972082373e-21	no change
4888	4888	MOV10	7.63250584291412e-26	0.10100631733697	0.071	0.046	1.66724457632616e-21	no change
4889	4889	IKZF4	7.86736497345982e-26	0.216390283	0.038	0.02	1.71854720480256e-21	no change
4890	4890	SLC27A2	7.88480315922337e-26	0.176156731839654	0.043	0.024	1.72235640210075e-21	no change
4891	4891	SYNJ2	8.18269040503873e-26	0.175152014236597	0.068	0.043	1.78742689207666e-21	no change
4892	4892	GLTSCR1L	8.20030382482546e-26	0.128764428496367	0.047	0.027	1.79127436749487e-21	no change
4893	4893	TGFBRAP1	8.21625072129155e-26	0.296852090088755	0.054	0.033	1.79475780755893e-21	no change
4894	4894	SAA2	8.38666334969189e-26	-2.550284011	0.005	0.016	1.8319827421067e-21	down
4895	4895	POLR3A	8.61584243394218e-26	0.365432633783701	0.044	0.025	1.88204462127033e-21	no change
4896	4896	PCDHB5	8.65100908209829e-26	0.828915289920621	0.013	0.003	1.88972642389355e-21	no change
4897	4897	MIER2	8.71464887343819e-26	0.120501327841517	0.084	0.057	1.90362789991384e-21	no change
4898	4898	PDLIM3	8.76573798311792e-2	-1.326448143	0.022	0.039	1.91478780503228e-2	down

			6				1	
4899	4899	PUDP	9.57668955543667e-26	0.164099284750368	0.076	0.051	2.09193206648959e-21	no change
4900	4900	C21orf62-AS1	9.66591873675142e-26	0.643790664089136	0.022	0.009	2.11142328885598e-21	no change
4901	4901	PTDSS2	9.68950053743652e-26	0.183319432450241	0.049	0.029	2.11657449739763e-21	no change
4902	4902	RP11-373D23.3	9.75553040445957e-26	0.317720886911731	0.031	0.015	2.13099806155015e-21	no change
4903	4903	RBM19	9.81900618855265e-26	0.134108232285735	0.069	0.044	2.14486371182744e-21	no change
4904	4904	SUOX	1.00047423212531e-25	0.153973855805354	0.07	0.045	2.18543591265452e-21	no change
4905	4905	POLM	1.07952549580806e-25	0.105296678199663	0.085	0.057	2.35811549304313e-21	no change
4906	4906	RDM1	1.14109317436674e-25	0.974306308237898	0.018	0.007	2.4926039300867e-21	no change
4907	4907	TMEM91	1.23254247308031e-25	-1.120868572	0.088	0.115	2.69236577819663e-21	down
4908	4908	GINS4	1.34078696480288e-25	0.6300802155219	0.022	0.009	2.9288150459154e-21	no change
4909	4909	KCNMB3	1.41277190538846e-25	0.641266938943267	0.025	0.011	3.08605895013054e-21	no change
4910	4910	UNC5B-AS1	1.43070636117448e-25	0.100522429880972	0.065	0.041	3.12523497534953e-21	no change
4911	4911	CCDC191	1.50167579468516e-2	0.36132750748736	0.04	0.022	3.28026060591027e-2	no

			5	6			1	change
4912	4912	HEATR5B	1.54019439555606e-25	0.225396246196762	0.048	0.029	3.36440063765266e-21	no change
4913	4913	HINFP	1.57982940556502e-25	0.322285652183547	0.044	0.025	3.45097935351623e-21	no change
4914	4914	ZBTB39	1.68710173365681e-25	1.02161816744767	0.017	0.006	3.68530502699994e-21	up
4915	4915	FPGT	1.70005435856599e-25	0.108212888925752	0.08	0.053	3.71359874085156e-21	no change
4916	4916	LINC00240	1.71377651374684e-25	0.250634647754401	0.054	0.033	3.7435734166286e-21	no change
4917	4917	TTI2	1.71382984563256e-25	0.215900958896292	0.043	0.025	3.74368991479977e-21	no change
4918	4918	MMS19	1.7994477832864e-25	0.19182926720563	0.056	0.034	3.93071373781082e-21	no change
4919	4919	DLX6	1.80347517003484e-25	0.834228708166961	0.022	0.01	3.9395111614241e-21	no change
4920	4920	MYO1D	1.81825514932535e-25	0.115349454790377	0.087	0.059	3.9717965481863e-21	no change
4921	4921	USP13	1.825474927794e-25	0.83013971284793	0.018	0.007	3.9875674322732e-21	no change
4922	4922	PMS2	1.920945017932e-25	0.130280871166118	0.082	0.055	4.19611229717067e-21	no change
4923	4923	MEX3D	2.0383180496904e-25	0.168936021956886	0.065	0.042	4.45250194774371e-21	no change
4924	4924	MTFR2	2.06567046892798e-2	0.47256904495896	0.023	0.01	4.51225057232629e-2	no

			5	7			1	change
4925	4925	LRRC16A	2.07766696246154e-25	-0.156287967	0.14	0.101	4.53845571280099e-21	no change
4926	4926	CLYBL	2.16321005250815e-25	0.265184593063911	0.048	0.028	4.7253160386988e-21	no change
4927	4927	CDR2	2.21681883299552e-25	0.121413384428531	0.094	0.065	4.84241905879542e-21	no change
4928	4928	CWF19L1	2.2771038286878e-25	0.231019894567843	0.039	0.022	4.97410560338563e-21	no change
4929	4929	LINC00339	2.29945017664088e-25	0.142221566543127	0.042	0.024	5.02291896585434e-21	no change
4930	4930	SMYD5	2.39498969066771e-25	0.279020619529321	0.043	0.024	5.23161548029454e-21	no change
4931	4931	PCDHB9	2.44050773687788e-25	0.559330899225759	0.019	0.007	5.33104510043605e-21	no change
4932	4932	UGDH	2.48732729937187e-25	-0.239682815	0.495	0.387	5.43331775274791e-21	no change
4933	4933	TRBC2	2.57620502654569e-25	0.494916010095709	0.076	0.051	5.62746225998641e-21	no change
4934	4934	LRSAM1	2.58875403860905e-25	0.39109536608701	0.036	0.02	5.6548743219376e-21	no change
4935	4935	YPEL1	2.61835444573179e-25	0.664564709460302	0.026	0.012	5.71953345125651e-21	no change
4936	4936	PINX1	2.62236832651712e-25	0.169824815893685	0.052	0.032	5.728301372444e-21	no change
4937	4937	PCDHA10	2.70026478586727e-2	0.97189350689819	0.013	0.004	5.89845839824847e-2	no

			5	4			1	change
4938	4938	PIGV	2.71736923105494e-25	0.180689776282683	0.089	0.061	5.93582134831641e-21	no change
4939	4939	SPTSSB	2.74582666050225e-25	0.139365730083847	0.452	0.386	5.9979837572011e-21	no change
4940	4940	DOPEY2	2.79086812533404e-25	0.315132340775499	0.051	0.03	6.09637233297967e-21	no change
4941	4941	TIMP1	2.88383135357023e-25	-0.775822931	0.789	0.726	6.29944120873882e-21	no change
4942	4942	CTC-260E6.6	2.91121901143061e-25	0.764958763222684	0.025	0.011	6.35926680856902e-21	no change
4943	4943	POLQ	2.93638390619012e-25	1.0577230205125	0.011	0.003	6.41423700468169e-21	up
4944	4944	LMLN	2.95742060610804e-25	0.306143512228946	0.043	0.025	6.4601895719824e-21	no change
4945	4945	JAG2	3.17743941252411e-25	0.499273730411856	0.029	0.014	6.94079865271767e-21	no change
4946	4946	FAAH2	3.20162807194183e-25	0.316788596086721	0.043	0.025	6.99363636034972e-21	no change
4947	4947	LMF1	3.47249848078104e-25	0.203793005895193	0.045	0.026	7.58532568141811e-21	no change
4948	4948	COL6A1	3.51330910945924e-25	-1.347751587	0.054	0.077	7.67447241870276e-21	down
4949	4949	S100A7	3.56293643462921e-25	-0.788619984	0.078	0.107	7.78287834780404e-21	no change
4950	4950	KLF16	3.57848004318435e-2	0.13492233925925	0.071	0.047	7.8168318063319e-21	no

			5	3				change
4951	4951	GOLIM4	3.6336566919258e-25	-0.280562208	0.252	0.191	7.93735967784272e-21	no change
4952	4952	GINS3	3.71978130139905e-25	0.578093657246144	0.025	0.011	8.12549027477609e-21	no change
4953	4953	OLFM2	3.95294326720009e-25	0.662951464339396	0.015	0.005	8.63480927287188e-21	no change
4954	4954	MAP4K2	4.67899911860725e-25	0.280458017393217	0.057	0.035	1.02208056746857e-20	no change
4955	4955	FES	4.75417557667376e-25	0.130070052538807	0.067	0.043	1.03850211296862e-20	no change
4956	4956	NAPB	4.777943833555e-25	0.116487719335534	0.054	0.033	1.04369405100175e-20	no change
4957	4957	DOCK5	4.84585889413753e-25	-0.100466454	0.095	0.065	1.0585294168354e-20	no change
4958	4958	C2orf44	5.26640886976271e-25	0.971366383922821	0.015	0.005	1.15039435351097e-20	no change
4959	4959	NOMO1	5.32195941663114e-25	0.267757498567125	0.048	0.028	1.16252881496891e-20	no change
4960	4960	PYCRL	5.46759538154694e-25	0.24668703132129	0.04	0.022	1.19434153514511e-20	no change
4961	4961	CLDN9	5.49053192378112e-25	-1.223526803	0.013	0.027	1.19935179343075e-20	down
4962	4962	CUL7	5.66580421786297e-25	0.345827190013757	0.042	0.024	1.23763827334999e-20	no change
4963	4963	SGK2	6.088709432412e-25	-0.872331159	0.039	0.061	1.33001768841608e-2	no

							0	change
4964	4964	BTAF1	6.30151078654702e-25	0.164555719751677	0.06	0.038	1.37650201621333e-20	no change
4965	4965	RFX3	6.35797678375431e-25	0.260692313865511	0.033	0.017	1.38883644864329e-20	no change
4966	4966	DLGAP1	6.45011906381876e-25	0.94238111560322	0.014	0.004	1.40896400830057e-20	no change
4967	4967	AC018890.6	6.52751994217622e-25	-0.921107407	0.025	0.043	1.42587145616897e-20	no change
4968	4968	AC005256.1	6.8749733687016e-25	-1.047411415	0.019	0.035	1.50176918265918e-20	down
4969	4969	EPHX3	6.88520758430826e-25	0.208942867495988	0.113	0.082	1.5040047447163e-20	no change
4970	4970	STK10	6.89417760164643e-25	0.170560553261915	0.05	0.03	1.50596415530365e-20	no change
4971	4971	TYW1	6.90438902171886e-25	0.134681122072782	0.077	0.051	1.50819473790427e-20	no change
4972	4972	EXTL3	7.262323024845e-25	0.423240721004969	0.03	0.015	1.58638184154714e-20	no change
4973	4973	LDAH	7.40324929702995e-25	0.145573726909978	0.08	0.053	1.61716577644322e-20	no change
4974	4974	AF131216.5	7.64590153592399e-25	0.81717045508339	0.026	0.012	1.67017073150724e-20	no change
4975	4975	RP13-192B19.2	7.72300580765904e-25	0.780634885488499	0.016	0.005	1.68701338862504e-20	no change
4976	4976	SUMF2	7.89150703708012e-2	-0.1406559	0.405	0.318	1.72382079717978e-2	no

			5				0	change
4977	4977	ELK1	8.03057964505016e-25	0.209668294431893	0.074	0.049	1.75419981766476e-20	no change
4978	4978	AMPD2	8.18049440931408e-25	0.152354477409006	0.07	0.046	1.78694719877057e-20	no change
4979	4979	ZFP82	8.37512170902376e-25	0.506004078558168	0.027	0.013	1.82946158611915e-20	no change
4980	4980	FKBP9	8.46638113854489e-25	-0.133739809	0.131	0.095	1.84939629590375e-20	no change
4981	4981	PRRG1	9.08211965596482e-25	0.15780577236522	0.05	0.03	1.98389821764895e-20	no change
4982	4982	PAXIP1	9.10646039451424e-25	0.29846691300814	0.043	0.025	1.98921520857769e-20	no change
4983	4983	CLDN3	9.25006831713455e-25	-0.268640937	0.147	0.108	2.02058492319487e-20	no change
4984	4984	RABL2A	9.46534287815107e-25	0.28312173228987	0.036	0.02	2.06760949830332e-20	no change
4985	4985	SEC24C	1.01680957306637e-24	0.141570796196479	0.083	0.056	2.22111883140617e-20	no change
4986	4986	MYOC	1.05509816636136e-24	-2.093676976	0.01	0.022	2.30475643459975e-20	down
4987	4987	AMMECR1L	1.18499121343271e-24	0.141003674551097	0.058	0.036	2.5884948066224e-20	no change
4988	4988	NHSL2	1.23410258659441e-24	0.159419527682314	0.051	0.03	2.69577369015684e-20	no change
4989	4989	RP11-134G8.5	1.27986269945138e-2	0.51021560725883	0.021	0.009	2.79573208068159e-2	no

			4	8			0	change
4990	4990	DECR2	1.31071206443833e-24	0.113555419629607	0.085	0.058	2.86311943355908e-20	no change
4991	4991	FAM201A	1.31143620227899e-24	1.52431318119266	0.01	0.002	2.86470124025824e-20	up
4992	4992	ZNF140	1.33412245355329e-24	0.18685111055472	0.069	0.045	2.91425708754181e-20	no change
4993	4993	SETDB2	1.34963067797323e-24	0.174953428665192	0.046	0.027	2.94813325296473e-20	no change
4994	4994	AKAP13	1.35192563694157e-24	-0.160756877	0.306	0.237	2.95314636133516e-20	no change
4995	4995	GPS2	1.35341084261923e-24	0.104904362110627	0.072	0.047	2.95639064461744e-20	no change
4996	4996	RP11-736N17.10	1.37033080467104e-24	-1.108995899	0.03	0.049	2.99335060972341e-20	down
4997	4997	HAS1	1.45134907092175e-24	-2.321907967	0.007	0.017	3.17032691052148e-20	down
4998	4998	SLC5A6	1.47795108350892e-24	0.107357601819848	0.079	0.053	3.22843634681689e-20	no change
4999	4999	RHPN2	1.51486457907291e-24	0.183238092274506	0.07	0.046	3.30907018652686e-20	no change
5000	5000	SLC25A22	1.62350667171406e-24	0.240532079107629	0.064	0.041	3.5463879736922e-20	no change
5001	5001	PRRX1	1.68151052001339e-24	-1.352627864	0.023	0.04	3.67309157991724e-20	down
5002	5002	PROSER3	1.71345570825683e-2	0.15170298635180	0.06	0.038	3.74287264911621e-2	no

			4	7			0	change
5003	5003	CCDC125	1.71414016634172e-24	0.23930620515607	0.055	0.034	3.74436777935686e-20	no change
5004	5004	ANKRD28	1.72744343606051e-24	-0.336531944	0.141	0.102	3.77342744173059e-20	no change
5005	5005	SLC19A1	1.81147628777414e-24	0.401128711140604	0.039	0.022	3.95698880301383e-20	no change
5006	5006	POLL	1.8443408442599e-24	0.12575562639627	0.07	0.046	4.02877814020132e-20	no change
5007	5007	SDK1	1.89380273331779e-24	0.271189701920289	0.025	0.011	4.13682269065937e-20	no change
5008	5008	TGDS	1.89895007101473e-24	0.139906651320363	0.062	0.039	4.14806653512458e-20	no change
5009	5009	MBLAC2	2.05822233800333e-24	0.481919276335182	0.024	0.011	4.49598087513447e-20	no change
5010	5010	WFDC2	2.32083086464177e-24	-0.352817475	0.598	0.488	5.06962294072348e-20	no change
5011	5011	PRKAR2A	2.33099219628521e-24	-0.101306173	0.197	0.151	5.09181935356541e-20	no change
5012	5012	LYPD6	2.33639742163378e-24	0.304633873911906	0.062	0.04	5.10362652781684e-20	no change
5013	5013	C22orf29	2.34893230628601e-24	0.189189187061409	0.059	0.037	5.13100772985117e-20	no change
5014	5014	HIST1H4E	2.55956135555463e-24	0.557253498006617	0.04	0.022	5.59110582507352e-20	no change
5015	5015	HIPK3	2.56819786386233e-2	-0.167732884	0.141	0.103	5.60997141382087e-2	no

			4				0	change
5016	5016	CLEC7A	2.65094576891494e-24	-0.901873565	0.03	0.049	5.7907259376178e-20	no change
5017	5017	DLG2	2.71923022707381e-24	0.819936270628847	0.026	0.013	5.93988650802003e-20	no change
5018	5018	DDX31	2.82802535439801e-24	0.386225938227753	0.027	0.013	6.17753858414702e-20	no change
5019	5019	SCNN1G	2.89086046875199e-24	0.355381682208873	0.066	0.043	6.31479560794184e-20	no change
5020	5020	KIF18B	2.91700555429956e-24	1.36879817639845	0.01	0.002	6.37190693281195e-20	up
5021	5021	AC074117.10	3.03408322446995e-24	0.104250261040479	0.055	0.034	6.62765139553216e-20	no change
5022	5022	ANXA1	3.09864637143902e-24	-0.416035892	0.599	0.581	6.7686831337714e-20	no change
5023	5023	FOXN3	3.20655787578278e-24	-0.120396743	0.216	0.166	7.00440502385991e-20	no change
5024	5024	KDM4C	3.23757983205976e-24	0.133704919259316	0.058	0.036	7.07216938515135e-20	no change
5025	5025	SFR1	3.24477160459694e-24	0.127798640977066	0.066	0.043	7.08787909308156e-20	no change
5026	5026	PIF1	3.39937339260355e-24	1.07491721144447	0.019	0.008	7.42559123880319e-20	up
5027	5027	IRX5	3.50379932058391e-24	0.401391764528507	0.032	0.017	7.65369923588349e-20	no change
5028	5028	LZTR1	3.58884944346091e-2	0.19208834498845	0.044	0.025	7.83948272429601e-2	no

			4	4			0	change
5029	5029	SLC12A7	3.64349356795033e-24	0.14310942405571	0.096	0.067	7.9588473498307e-20	no change
5030	5030	ZNF346	3.70492348839985e-24	0.375474185212644	0.029	0.015	8.09303486806063e-20	no change
5031	5031	F5	3.79061142539469e-24	0.794786856019418	0.021	0.009	8.28021159763216e-20	no change
5032	5032	TSHZ1	3.93200620252595e-24	0.100096999906953	0.051	0.031	8.58907434879769e-20	no change
5033	5033	ABCB10	4.03774443884542e-24	0.264020584562543	0.046	0.027	8.82004895221394e-20	no change
5034	5034	BCDIN3D	4.17628898599031e-24	0.26941477662866	0.046	0.027	9.12268566099723e-20	no change
5035	5035	ASTN2	4.17818412409296e-24	0.594710010439192	0.022	0.01	9.12682540066867e-20	no change
5036	5036	KDM1B	4.39699786117342e-24	0.298543836251415	0.046	0.027	9.60480212794722e-20	no change
5037	5037	SPATA24	4.43899777255687e-24	0.179838241285565	0.054	0.034	9.69654673437322e-20	no change
5038	5038	WDPCP	4.44737793055299e-24	0.413936937210284	0.029	0.014	9.71485235149995e-20	no change
5039	5039	LIF	4.46753113447852e-24	-1.095004105	0.043	0.065	9.75887501015488e-20	down
5040	5040	PAG1	4.46870460761569e-24	0.112687102777491	0.094	0.066	9.76143834487572e-20	no change
5041	5041	MT1G	4.5947134889669e-24	-0.182322677	0.045	0.026	1.00366921452993e-1	no

							9	change
5042	5042	PLEKHA8	4.67879843696406e-24	0.289938783092447	0.048	0.028	1.02203673057043e-19	no change
5043	5043	C15orf41	4.79794211934252e-24	0.147091043631735	0.072	0.048	1.04806247654918e-19	no change
5044	5044	SLC1A6	4.90922708981033e-24	-0.115747003	0.02	0.008	1.07237156549817e-19	no change
5045	5045	FAM193A	5.04333192493167e-24	0.130075027339866	0.058	0.036	1.10166542568207e-19	no change
5046	5046	TMEM37	5.06695579285677e-24	-0.957858262	0.068	0.093	1.10682582339163e-19	no change
5047	5047	ALG10	5.37329137458403e-24	0.321111637636581	0.032	0.017	1.17374176786414e-19	no change
5048	5048	TMEM102	5.49112173138256e-24	0.208057766696824	0.061	0.039	1.19948063100321e-19	no change
5049	5049	SNAPC4	5.60954028882587e-24	0.483411670220622	0.028	0.014	1.22534798069112e-19	no change
5050	5050	ATP6V0E1	5.73505981124976e-24	-0.155393491	0.907	0.842	1.2527664651694e-19	no change
5051	5051	CDC42SE2	6.00369538025273e-24	-0.169157909	0.336	0.262	1.31144721886241e-19	no change
5052	5052	CLN5	6.32189829289983e-24	-0.133690823	0.182	0.137	1.38095546310104e-19	no change
5053	5053	TMEM106C	6.34582017743048e-24	0.181167459645522	0.341	0.28	1.38618095955791e-19	no change
5054	5054	GTF2IRD2B	6.49290513210181e-2	0.15414060965942	0.05	0.03	1.41831019705632e-1	no

			4				9	change
5055	5055	TMEM52	6.93902551957744e-24	0.384044289134316	0.037	0.02	1.5157607344965e-19	no change
5056	5056	PRICKLE3	7.48072064708631e-24	0.280915676535468	0.046	0.027	1.63408861814953e-19	no change
5057	5057	C22orf46	7.5468723013233e-24	0.214749805394066	0.042	0.025	1.64853878550106e-19	no change
5058	5058	ZNF695	7.67596449282884e-24	1.20443698172821	0.014	0.005	1.67673768381353e-19	up
5059	5059	BDNF-AS	8.16120205686493e-24	0.543149574752966	0.024	0.011	1.78273297730158e-19	no change
5060	5060	KIAA1919	8.45496241950119e-24	0.438985996455266	0.035	0.019	1.84690199091584e-19	no change
5061	5061	INTS7	8.7873894086532e-24	0.216390609541936	0.041	0.024	1.91951734242621e-19	no change
5062	5062	TXNDC15	9.11940036608712e-24	-0.190581417	0.194	0.145	1.99204181596807e-19	no change
5063	5063	CCDC93	9.33873790161763e-24	0.190473179998394	0.055	0.034	2.03995390722936e-19	no change
5064	5064	RP11-385D13.3	9.79019584604449e-24	0.222063990654247	0.035	0.019	2.13857038060996e-19	no change
5065	5065	RGS22	1.0057054813394e-23	-2.45204603	0.004	0.012	2.19686305343779e-19	down
5066	5066	ACTB	1.05321303382755e-23	-0.11194566	0.999	0.998	2.30063855109289e-19	no change
5067	5067	ISLR	1.05517095856358e-2	-1.490087229	0.018	0.033	2.30491544188628e-1	down

			3				9	
5068	5068	IL10RB-AS1	1.08375407749117e-23	-1.121473097	0.02	0.035	2.3673524068717e-19	down
5069	5069	RNF144A	1.08945467861036e-23	0.520958805583902	0.032	0.017	2.37980479995648e-19	no change
5070	5070	RPS4Y1	1.09736164635785e-23	-0.117486239	0.705	0.795	2.39707678030408e-19	no change
5071	5071	RP11-511B23.1	1.11064648434454e-23	1.16829792468088	0.011	0.003	2.42609618040222e-19	up
5072	5072	PCDHB14	1.12743490951595e-23	0.362710289808425	0.025	0.012	2.46276881634664e-19	no change
5073	5073	B4GALT6	1.12948430198506e-23	0.590973310022434	0.018	0.007	2.46724550925616e-19	no change
5074	5074	ZNF708	1.1553642452387e-23	0.391771247908203	0.032	0.016	2.52377765729941e-19	no change
5075	5075	VIT	1.16340793298605e-23	-2.613539912	0.005	0.015	2.54134828881473e-19	down
5076	5076	RP11-902B17.1	1.23065782196345e-23	0.168443341072825	0.047	0.028	2.68824894629696e-19	no change
5077	5077	KANSL3	1.25639771337726e-23	0.193099555032913	0.055	0.035	2.74447516510128e-19	no change
5078	5078	RGS16	1.2618399432734e-23	-0.907673343	0.198	0.224	2.75636317208641e-19	no change
5079	5079	WRAP53	1.26797464728711e-23	0.423808252452372	0.035	0.019	2.76976381953396e-19	no change
5080	5080	RP11-553L6.5	1.37486802275077e-2	-0.209507152	0.078	0.052	3.00326170889679e-1	no

			3				9	change
5081	5081	SPRTN	1.37630701550117e-23	0.109976958505741	0.099	0.07	3.00640504466076e-19	no change
5082	5082	HECTD4	1.39005327151046e-23	0.160865709806182	0.067	0.044	3.03643236628745e-19	no change
5083	5083	JSRP1	1.39438856169867e-23	0.444908039941303	0.019	0.007	3.04590237417458e-19	no change
5084	5084	RBM34	1.43710143439975e-23	0.203329293289729	0.055	0.034	3.13920437330282e-19	no change
5085	5085	FRAT2	1.44999977180849e-23	0.101832124092667	0.105	0.075	3.16737950153847e-19	no change
5086	5086	DANT2	1.45771280560468e-23	0.769715575300716	0.015	0.005	3.18422785256285e-19	no change
5087	5087	C3orf14	1.5012602733818e-23	-0.408714677	0.279	0.309	3.2793529411752e-19	no change
5088	5088	XXyac-YM21GA2.4	1.52112630323753e-23	0.647257888330582	0.03	0.015	3.32274829679207e-19	no change
5089	5089	FANCC	1.55046065281724e-23	0.740893196060945	0.016	0.006	3.38682625001398e-19	no change
5090	5090	IFT122	1.5948086146609e-23	0.316076001513977	0.026	0.013	3.48369993786528e-19	no change
5091	5091	RBP1	1.63105607461265e-23	-1.198618279	0.034	0.053	3.56287888938387e-19	down
5092	5092	DTNB	1.64251976318072e-23	0.143432851199142	0.084	0.058	3.58792017069196e-19	no change
5093	5093	BCL2L15	1.65879645303312e-2	-1.461460308	0.013	0.026	3.62347497200555e-1	down

			3				9	
5094	5094	ERCC2	1.67557809315279e-23	0.12348359631853	0.053	0.033	3.66013278668296e-19	no change
5095	5095	SGCE	1.77587024625571e-23	-0.150843599	0.062	0.039	3.87921096592097e-19	no change
5096	5096	DCAF17	1.82535367958798e-23	0.128112575462032	0.05	0.03	3.98730257769198e-19	no change
5097	5097	NUFIP1	1.83417989441303e-23	0.125909700119667	0.069	0.046	4.00658256135583e-19	no change
5098	5098	NOP10	1.8475605038919e-23	-0.219713118	0.882	0.796	4.03581116470146e-19	no change
5099	5099	TNNI3	1.88701979267866e-23	0.930093374676911	0.012	0.004	4.12200603512727e-19	no change
5100	5100	KNSTRN	1.99506957426559e-23	0.606468289637959	0.037	0.021	4.35802997802576e-19	no change
5101	5101	AFAP1	2.01495938295131e-23	0.319113862854873	0.034	0.018	4.40147727611884e-19	no change
5102	5102	ZNF367	2.05167225236702e-23	0.75827884537283	0.014	0.005	4.48167286807052e-19	no change
5103	5103	CYFIP2	2.07367907218739e-23	0.134525958274954	0.068	0.044	4.52974456528614e-19	no change
5104	5104	TRAF4	2.10079709011082e-23	-0.452959673	0.586	0.536	4.58898116363808e-19	no change
5105	5105	ETF1	2.14162130221076e-23	-0.107837139	0.38	0.3	4.67815757254918e-19	no change
5106	5106	FAM206A	2.17614672375456e-2	0.16424909021192	0.055	0.034	4.75357490336945e-1	no

			3	1			9	change
5107	5107	L2HGDH	2.21024789665627e-23	0.284567956266738	0.047	0.028	4.82806550545596e-19	no change
5108	5108	FAM135A	2.22255489862191e-23	0.198604628240076	0.066	0.043	4.85494892054971e-19	no change
5109	5109	WASIR2	2.31405306379886e-23	0.826779860576765	0.018	0.007	5.05481751256223e-19	no change
5110	5110	RP11-357H14.17	2.37645582153665e-23	0.123202148416975	0.095	0.067	5.19113009656465e-19	no change
5111	5111	MARK4	2.45368832341798e-23	0.196499468788131	0.065	0.043	5.35983677367424e-19	no change
5112	5112	CWF19L2	2.49642254030994e-23	-0.175971223	0.149	0.11	5.45318539705303e-19	no change
5113	5113	CYB5R1	2.5620581694638e-23	-0.496533387	0.454	0.434	5.59655986537673e-19	no change
5114	5114	BCL2L14	2.6881829851151e-23	0.217978212588177	0.051	0.031	5.87206691268543e-19	no change
5115	5115	CTD-2017D11.1	2.69383700031555e-23	0.184224359793392	0.06	0.038	5.88441754348928e-19	no change
5116	5116	HABP2	3.01082476117937e-23	-1.226384777	0.017	0.032	6.57684560832022e-19	down
5117	5117	ACAD8	3.05435668466894e-23	0.132597836385244	0.085	0.059	6.67193674199083e-19	no change
5118	5118	ECM1	3.10156444291789e-23	-1.83677773	0.014	0.028	6.77505736910983e-19	down
5119	5119	CEBPD	3.14741289983114e-2	-0.517302959	0.739	0.68	6.87520873839114e-1	no

			3				9	change
5120	5120	C17orf53	3.17439504090534e-23	1.18807529569639	0.011	0.003	6.93414852735362e-19	up
5121	5121	IL17RE	3.19885182138556e-23	0.19179144292041	0.048	0.029	6.98757191863462e-19	no change
5122	5122	COX18	3.20539021128589e-23	0.163770011140151	0.049	0.03	7.00185437753289e-19	no change
5123	5123	BOK-AS1	3.25591333098865e-23	-0.975595387	0.014	0.027	7.1122170802116e-19	no change
5124	5124	GGT5	3.26977517085116e-23	-1.818167283	0.012	0.024	7.14249688320728e-19	down
5125	5125	PCDHB11	3.30680080054155e-23	0.4386193092503	0.018	0.007	7.22337566870297e-19	no change
5126	5126	TDRD3	3.40153885573475e-23	0.194705892833514	0.07	0.047	7.43032147646699e-19	no change
5127	5127	NEGR1	3.40877160266946e-23	-1.754727115	0.014	0.027	7.44612068887116e-19	down
5128	5128	HSD17B6	3.40992570413602e-23	-1.201665265	0.032	0.05	7.44864170811472e-19	down
5129	5129	NVL	3.47415378576472e-23	0.127704408848851	0.061	0.04	7.58894152962444e-19	no change
5130	5130	CEPT1	3.61172971582812e-23	-0.166030932	0.111	0.079	7.88946239125494e-19	no change
5131	5131	HIC2	3.61182605469404e-23	0.172177231803484	0.054	0.034	7.88967283387366e-19	no change
5132	5132	DVL1	3.64244578627264e-2	0.24109907296123	0.073	0.049	7.95655857553396e-1	no

			3	9			9	change
5133	5133	ZNF362	3.86915446257815e-23	0.100963941958608	0.058	0.037	8.4517810080557e-19	no change
5134	5134	RITA1	3.88325768774135e-23	0.134687222875679	0.123	0.091	8.4825880931022e-19	no change
5135	5135	TBC1D16	3.95525329104962e-23	0.278423554557182	0.037	0.021	8.6398552889688e-19	no change
5136	5136	SIN3B	3.98390953687813e-23	0.114743198310647	0.076	0.052	8.70245199235658e-19	no change
5137	5137	PALB2	3.98519970462271e-23	0.418879257658472	0.03	0.016	8.70527023477784e-19	no change
5138	5138	SLC39A11	4.07499692627353e-23	0.200124851796665	0.113	0.083	8.90142328575191e-19	no change
5139	5139	SCLT1	4.18781384912758e-23	0.166476162685843	0.06	0.038	9.14786057203429e-19	no change
5140	5140	SLC15A3	4.22175675558144e-23	-1.087046512	0.019	0.035	9.2220054568921e-19	down
5141	5141	DICER1-AS1	4.23092807106588e-23	0.100180877511693	0.058	0.037	9.2420392784363e-19	no change
5142	5142	INCENP	4.24556902937186e-23	0.828428302920027	0.016	0.006	9.27402098775989e-19	no change
5143	5143	PCAT1	4.65667242697108e-23	0.54057087728205	0.022	0.01	1.01720352494756e-18	no change
5144	5144	SLC17A9	4.70084166570485e-23	0.633689510952726	0.015	0.006	1.02685185345657e-18	no change
5145	5145	POLD1	4.73268455229407e-2	0.32733254716357	0.029	0.014	1.03380761360312e-1	no

			3	7			8	change
5146	5146	AUNIP	4.84134520021412e-23	1.16214353419509	0.015	0.005	1.05754344553477e-18	up
5147	5147	FN1	4.84309127984908e-23	-1.008460729	0.062	0.085	1.05792485917023e-18	down
5148	5148	ATG4C	4.91298462893515e-23	0.154973420575114	0.065	0.043	1.07319236234459e-18	no change
5149	5149	DHODH	4.9383400085581e-23	0.133777209968181	0.051	0.031	1.07873099146943e-18	no change
5150	5150	CCDC150	5.0440198980363e-23	0.573823375264837	0.02	0.009	1.10181570652705e-18	no change
5151	5151	RGS17	5.06425184938801e-23	0.236488205268774	0.036	0.02	1.10623517398032e-18	no change
5152	5152	FBXL2	5.40144527987054e-23	0.466018613296387	0.026	0.012	1.17989170693492e-18	no change
5153	5153	TP73-AS1	5.42542136558356e-23	0.274806472514346	0.021	0.009	1.18512904309807e-18	no change
5154	5154	ZNF436-AS1	5.47721384298927e-23	0.258981158318521	0.065	0.042	1.19644259186258e-18	no change
5155	5155	CSRP2BP	5.66541642288129e-23	0.281421333465335	0.034	0.019	1.23755356341419e-18	no change
5156	5156	DNAJC15	5.83893222798937e-23	-0.127133501	0.41	0.315	1.275456355882e-18	no change
5157	5157	IFT46	5.85380777618997e-23	0.115365135396864	0.071	0.047	1.27870577063094e-18	no change
5158	5158	GEM	5.92932429016707e-2	-1.05313186	0.055	0.078	1.2952015979441e-18	down

			3					
5159	5159	SMN1	6.08989647733769e-23	0.338473013378131	0.029	0.015	1.33027698650964e-18	no change
5160	5160	CDCA2	6.12887144211536e-23	1.29237040304604	0.011	0.003	1.33879067781568e-18	up
5161	5161	ZBTB17	6.26385772218038e-23	0.125902213659342	0.078	0.053	1.36827708083308e-18	no change
5162	5162	SLC39A2	6.59232794345598e-23	-1.476642351	0.008	0.02	1.44002811596852e-18	down
5163	5163	TYMP	7.02493496627498e-23	-0.555920066	0.366	0.375	1.53452679403311e-18	no change
5164	5164	USP44	7.5148693382686e-23	0.791396487754901	0.015	0.005	1.64154805825139e-18	no change
5165	5165	RQCD1	7.60367565922351e-23	-0.1425477	0.212	0.165	1.66094691100078e-18	no change
5166	5166	OXSM	7.64305757136735e-23	0.28696636467208	0.061	0.039	1.66954949588948e-18	no change
5167	5167	IFNAR2	7.68274998692085e-23	-0.120254016	0.173	0.131	1.67821990714299e-18	no change
5168	5168	AADAT	8.10438832219508e-23	0.336336929643078	0.045	0.027	1.77032258510029e-18	no change
5169	5169	RP11-53O19.3	8.47316745254613e-23	0.61016262279094	0.023	0.01	1.85087869833418e-18	no change
5170	5170	SMCR8	8.97189405784878e-23	0.637715176513972	0.029	0.015	1.95982053799649e-18	no change
5171	5171	DGUOK-AS1	9.81832305032269e-2	0.16718601901397	0.071	0.048	2.14471448711249e-1	no

			3	1			8	change
5172	5172	ZSCAN29	1.00642054158267e-22	0.352656951905434	0.038	0.021	2.19842503103319e-18	no change
5173	5173	DDX20	1.02860060900632e-22	0.396944938662754	0.03	0.016	2.2468751703134e-18	no change
5174	5174	FBXO16	1.04869966915911e-22	0.410268811966431	0.036	0.02	2.29077955731116e-18	no change
5175	5175	CUL9	1.07668018044349e-22	0.302214129632108	0.048	0.029	2.35190018616075e-18	no change
5176	5176	REXO1	1.12554394564276e-22	0.123475286067491	0.066	0.044	2.45863819486204e-18	no change
5177	5177	FKTN	1.25295764397795e-22	0.147750937634425	0.034	0.019	2.73696067750543e-18	no change
5178	5178	CCDC30	1.25730123536188e-22	0.421646192349853	0.025	0.012	2.74644881852449e-18	no change
5179	5179	COL27A1	1.26502732024195e-22	0.383019487119087	0.033	0.018	2.76332567833653e-18	no change
5180	5180	ZNF136	1.32198314074961e-22	0.146755436809431	0.045	0.027	2.88773997265345e-18	no change
5181	5181	IL6	1.32516881417589e-22	-1.569108544	0.016	0.03	2.89469875768582e-18	down
5182	5182	AC013461.1	1.51882753688637e-22	-0.243869592	0.114	0.082	3.31772687157459e-18	no change
5183	5183	MARK1	1.57983811887535e-22	0.27135697838158	0.068	0.046	3.45099838687132e-18	no change
5184	5184	OSGEPL1	1.70846654389381e-2	0.33077820859507	0.03	0.015	3.73197431848163e-1	no

			2	5			8	change
5185	5185	MED22	1.75974368785222e-22	0.29625420540471	0.037	0.021	3.84398411174438e-18	no change
5186	5186	SRSF5	1.80957086757891e-22	-0.219257297	0.806	0.738	3.95282660313937e-18	no change
5187	5187	CASD1	1.88120995750263e-22	0.187546132056987	0.06	0.039	4.10931503116873e-18	no change
5188	5188	RP11-7K24.3	1.9333256958015e-22	0.195839254391455	0.037	0.021	4.22315664990881e-18	no change
5189	5189	ITIH5	1.93636133413084e-22	-1.488380077	0.016	0.029	4.2297876982754e-18	down
5190	5190	LY6G5C	1.96938858418026e-22	0.219896660747164	0.049	0.03	4.30193242328336e-18	no change
5191	5191	C3orf70	1.96942316450286e-22	0.931051371390262	0.012	0.004	4.30200796054005e-18	no change
5192	5192	WSB2	2.01930985633249e-22	-0.107333551	0.244	0.192	4.41098045017269e-18	no change
5193	5193	ZNF620	2.04391779967396e-22	0.426941578426542	0.029	0.015	4.46473404160779e-18	no change
5194	5194	LINC00937	2.07261963645724e-22	0.329402605157195	0.023	0.011	4.52743033387719e-18	no change
5195	5195	MOCOS	2.10004571323522e-22	0.24817946022685	0.057	0.037	4.58733985599101e-18	no change
5196	5196	CTD-2026G6.3	2.11198990145168e-22	-1.497987116	0.007	0.018	4.61343074073104e-18	down
5197	5197	NDUFB10	2.13475937084179e-2	0.10358385093377	0.824	0.713	4.6631683696668e-18	no

			2	2				change
5198	5198	STX8	2.14466275998488e-22	-0.111860088	0.261	0.204	4.68480133291098e-18	no change
5199	5199	TSPAN3	2.18417382872072e-22	-0.112989162	0.271	0.212	4.77110931145755e-18	no change
5200	5200	JOSD1	2.20500925692541e-22	-0.114050028	0.253	0.198	4.81662222082787e-18	no change
5201	5201	TUBGCP5	2.23660354038338e-22	0.24008414006109	0.034	0.019	4.88563677361346e-18	no change
5202	5202	FUT3	2.26949410343692e-22	-0.832820531	0.039	0.059	4.95748291954761e-18	no change
5203	5203	PMEPA1	2.57998905033481e-22	-0.17978422	0.127	0.092	5.63572808155136e-18	no change
5204	5204	ODF3L1	2.59123111898641e-22	0.359219473990965	0.023	0.011	5.6602852563139e-18	no change
5205	5205	ZNF74	2.74885397232164e-22	0.455699926580515	0.031	0.017	6.0045966171394e-18	no change
5206	5206	IPP	2.75551360980732e-22	0.13413449375226	0.05	0.031	6.0191439292631e-18	no change
5207	5207	BLOC1S5	2.76623174941493e-22	0.208150153094777	0.066	0.043	6.04255663342197e-18	no change
5208	5208	BRPF3	2.92400314084298e-22	0.189024005713103	0.045	0.027	6.38719246085742e-18	no change
5209	5209	RTN4	2.94666627942027e-22	-0.112752723	0.947	0.902	6.43669782076563e-18	no change
5210	5210	MYEF2	2.96605186692185e-2	0.85962516361432	0.019	0.008	6.47904369810409e-1	no

			2	7			8	change
5211	5211	PDLIM7	2.99741988454878e-22	-0.194736571	0.132	0.097	6.54756399580836e-18	no change
5212	5212	FOXP4	3.03931041686977e-22	0.241382869335073	0.052	0.032	6.63906967461033e-18	no change
5213	5213	RP5-1033H22.2	3.07045892297815e-22	0.954304970546152	0.012	0.004	6.70711047135347e-18	no change
5214	5214	CREM	3.08381731626715e-22	-0.183646007	0.269	0.213	6.73629054565396e-18	no change
5215	5215	PHYHIP	3.24729251835128e-22	0.168480997742248	0.031	0.016	7.09338577708653e-18	no change
5216	5216	DTD2	3.27986548987916e-22	0.100958702718426	0.054	0.034	7.16453817609204e-18	no change
5217	5217	PRR15L	3.46737983066102e-22	-1.09106952	0.029	0.047	7.57414450209593e-18	down
5218	5218	MTPAP	3.55856462523376e-22	-0.106064576	0.096	0.068	7.77332856736063e-18	no change
5219	5219	TSPAN1	3.57129491365251e-22	-0.291007204	0.375	0.402	7.80113660938255e-18	no change
5220	5220	RGS20	3.59386750335144e-22	1.17876358695024	0.012	0.004	7.85044417432088e-18	up
5221	5221	ZNF93	3.61142320545469e-22	0.24986963384331	0.046	0.028	7.88879284999522e-18	no change
5222	5222	AMT	3.66012294278048e-22	0.131186849737856	0.069	0.046	7.99517255620969e-18	no change
5223	5223	DARS2	3.80927924102811e-2	0.21517106061102	0.04	0.024	8.32098957410181e-1	no

			2	6			8	change
5224	5224	FIGN	4.02410989027214e-22	0.343898076232819	0.048	0.029	8.79026564431046e-18	no change
5225	5225	HOXA6	4.03525968653557e-22	0.395051487633221	0.023	0.011	8.8146212592683e-18	no change
5226	5226	TNFRSF10A	4.22518790598475e-22	0.339748053787124	0.04	0.023	9.2295004618331e-18	no change
5227	5227	HIST1H1C	4.2482627467676e-22	0.347088825712662	0.279	0.237	9.27990514403915e-18	no change
5228	5228	IFNGR1	4.34942990432449e-22	-0.158688137	0.408	0.321	9.50089468300641e-18	no change
5229	5229	TGM2	4.4753412524343e-22	-1.940547667	0.021	0.037	9.77593543181749e-18	down
5230	5230	PVRL3	4.55511263420206e-22	0.339395281979812	0.027	0.014	9.95018803815099e-18	no change
5231	5231	TCF15	4.55916414999278e-22	1.36143073643821	0.014	0.005	9.95903816924424e-18	up
5232	5232	GNPNAT1	4.72230531683744e-22	-0.166852923	0.139	0.103	1.03154037340997e-17	no change
5233	5233	KATNAL2	4.80854350024396e-22	0.842526417484039	0.017	0.007	1.05037824219329e-17	no change
5234	5234	KIAA1456	4.92028403201734e-22	0.666891761755459	0.018	0.008	1.07478684395387e-17	no change
5235	5235	TOR1AIP1	4.9312273347206e-22	-0.105854279	0.185	0.142	1.07717729899637e-17	no change
5236	5236	FXYD5	4.9763983078211e-22	-0.205531476	0.547	0.427	1.08704444636044e-1	no

							7	change
5237	5237	C2orf76	5.14437334297802e-22	-0.1045303	0.197	0.153	1.12373691304012e-17	no change
5238	5238	NEURL3	5.31560208406003e-22	-1.601529185	0.008	0.018	1.16114011924207e-17	down
5239	5239	SIRT3	5.34275406614028e-22	0.10016915921946	0.053	0.034	1.16707119820768e-17	no change
5240	5240	KIAA1211L	5.40140675421019e-22	0.189347610118288	0.065	0.043	1.17988329138967e-17	no change
5241	5241	AC018816.3	5.40478907140894e-22	-0.732167201	0.087	0.114	1.18062212475857e-17	no change
5242	5242	TGFBR3	5.5190921208784e-22	-0.353471007	0.187	0.142	1.20559048288468e-17	no change
5243	5243	RNF125	5.53428478321803e-22	0.52852932187386	0.018	0.007	1.20890916804615e-17	no change
5244	5244	TMEM164	5.87583454508169e-22	0.275226415807603	0.053	0.034	1.28351729802764e-17	no change
5245	5245	LARGE	5.88051610189761e-22	0.17995860782378	0.047	0.029	1.28453993729851e-17	no change
5246	5246	PACRG	5.90017467591537e-22	0.105778504790228	0.091	0.064	1.28883415620695e-17	no change
5247	5247	EXOC2	6.04949447755879e-22	0.173555152733624	0.063	0.042	1.32145157367794e-17	no change
5248	5248	CERCAM	6.38796618831066e-22	-1.11765931	0.058	0.081	1.39538733417458e-17	down
5249	5249	ZZEF1	6.64146209020612e-2	0.22608103834255	0.05	0.031	1.45076097898463e-1	no

			2	9			7 change
5250	5250	ZNF786	6.76032115152748e-22	0.482059479439886	0.026	0.013	1.47672455233966e-17 no change
5251	5251	FAM181B	7.29419441436958e-22	0.51158689278647	0.028	0.014	1.59334382787489e-17 no change
5252	5252	FAM227B	7.3700504120593e-22	0.242627307787183	0.033	0.018	1.60991381201023e-17 no change
5253	5253	DNAL1	7.41874585746465e-22	0.133581470793405	0.075	0.051	1.62055084510458e-17 no change
5254	5254	C9orf40	7.44133463817704e-22	0.469289775587796	0.025	0.012	1.62548513836339e-17 no change
5255	5255	FNTB	7.64473073905343e-22	0.379064307545455	0.036	0.021	1.66991498263883e-17 no change
5256	5256	AC004069.2	7.64718831532196e-22	0.616391755065919	0.016	0.007	1.67045181559893e-17 no change
5257	5257	RP11-195B3.1	7.80913281264465e-22	0.949893289910772	0.016	0.006	1.7058269715941e-17 no change
5258	5258	HIST1H2BN	8.09368064758018e-22	0.1870364224692	0.08	0.056	1.76798360065742e-17 no change
5259	5259	TTC38	8.137377370298e-22	0.244271360842069	0.051	0.032	1.77752871276789e-17 no change
5260	5260	DCAF15	8.15720660722788e-22	0.154563190561941	0.05	0.031	1.78186021128286e-17 no change
5261	5261	HAUS4	8.28784698720012e-22	0.219331065510447	0.053	0.033	1.81039729588399e-17 no change
5262	5262	TEX9	8.36563011762644e-2	0.47373195692717	0.036	0.02	1.82738824289432e-1 no

			2	4			7	change
5263	5263	LPAR1	8.49845461975596e-22	-0.410504303	0.082	0.056	1.85640242713949e-17	no change
5264	5264	ITFG1	8.52405885673665e-22	-0.121768729	0.303	0.239	1.86199541666555e-17	no change
5265	5265	SPECC1L	8.61124215120709e-22	0.305793125572159	0.037	0.021	1.88103973550968e-17	no change
5266	5266	SV2C	9.0726047664597e-22	1.0085783493595	0.012	0.003	1.98181978518546e-17	up
5267	5267	XX-CR54.1	9.19871414019711e-22	-0.65371603	0.027	0.044	2.00936711678466e-17	no change
5268	5268	C5orf30	9.22036419357656e-22	0.196817587890055	0.044	0.026	2.01409635444486e-17	no change
5269	5269	DCP1B	9.38900534967954e-22	0.31386809281369	0.04	0.023	2.050934328584e-17	no change
5270	5270	ZNF669	9.54299278319389e-22	0.176656703065794	0.052	0.033	2.08457134356087e-17	no change
5271	5271	GCNT2	9.57635973321034e-22	0.311186207694484	0.035	0.019	2.09186002012247e-17	no change
5272	5272	NGF	9.78462119528839e-22	-2.561204659	0.003	0.011	2.1373526538988e-17	down
5273	5273	SEPSECS	9.83577683714963e-22	0.286054722567833	0.038	0.022	2.14852709230696e-17	no change
5274	5274	ZNF776	1.01103671132284e-21	0.396576453678026	0.031	0.017	2.20850859221361e-17	no change
5275	5275	ERICH5	1.06908223298636e-2	0.15187271797837	0.095	0.068	2.33530322973539e-1	no

			1	6			7	change
5276	5276	CNTROB	1.08691421580734e-21	0.275956221298041	0.039	0.023	2.37425541300954e-17	no change
5277	5277	RP11-400F19.6	1.0893139279768e-21	0.273683226040133	0.048	0.03	2.37949734427252e-17	no change
5278	5278	MED9	1.09350990551988e-21	0.286254733955142	0.039	0.023	2.38866303761762e-17	no change
5279	5279	POFUT1	1.10182555797877e-21	0.104389646640102	0.054	0.034	2.40682774884883e-17	no change
5280	5280	NPM2	1.13896865618985e-21	0.450251999957488	0.03	0.016	2.4879631325811e-17	no change
5281	5281	MTERF4	1.14607432669124e-21	-0.100449074	0.157	0.119	2.50348475922435e-17	no change
5282	5282	TMEM117	1.15640739284927e-21	0.12976887090083	0.071	0.048	2.52605630893995e-17	no change
5283	5283	ZNF670	1.16571473403603e-21	0.126170423669888	0.071	0.048	2.54638726502831e-17	no change
5284	5284	SUV39H1	1.18503324682653e-21	0.736165023813656	0.015	0.005	2.58858662436787e-17	no change
5285	5285	AGK	1.23695794911396e-21	0.171719032482797	0.05	0.031	2.70201094404453e-17	no change
5286	5286	IFT74	1.25664826443086e-21	0.137531717582513	0.052	0.032	2.74502246882277e-17	no change
5287	5287	HSD11B1	1.32354850086653e-21	-2.237103345	0.007	0.017	2.89115934529284e-17	down
5288	5288	ZBTB6	1.33359993172563e-2	0.54327950933562	0.02	0.009	2.91311569086146e-1	no

			1	5			7	change
5289	5289	FOXJ2	1.38941308423071e-21	0.253510992539866	0.037	0.021	3.03503394119355e-17	no change
5290	5290	GYS1	1.40305330660105e-21	0.105747393781722	0.08	0.055	3.06482964293933e-17	no change
5291	5291	WISP3	1.40346535081563e-21	-0.989322988	0.018	0.032	3.06572971232167e-17	no change
5292	5292	SETDB1	1.46014644063021e-21	0.161564531098499	0.057	0.037	3.18954388491262e-17	no change
5293	5293	GUSB	1.50245244506626e-21	-0.116071359	0.262	0.207	3.28195712100274e-17	no change
5294	5294	ALG13	1.50328678120087e-21	-0.108145664	0.263	0.207	3.28377964485519e-17	no change
5295	5295	POLE	1.52035156845544e-21	0.357595115341462	0.035	0.02	3.32105596613406e-17	no change
5296	5296	FGD2	1.53762396489075e-21	-0.854741221	0.035	0.054	3.35878578890736e-17	no change
5297	5297	ARHGEF10	1.57815052174646e-21	0.13956283189427	0.04	0.024	3.44731199970296e-17	no change
5298	5298	CD99	1.57973138213952e-21	-0.619735196	0.488	0.477	3.45076523114557e-17	no change
5299	5299	NBPF3	1.59817293701311e-21	0.334655362563968	0.025	0.012	3.49104896361144e-17	no change
5300	5300	EPHB2	1.59846664291888e-21	0.385544784254126	0.02	0.009	3.49169053479201e-17	no change
5301	5301	CTBP1-AS2	1.62886990504435e-2	0.28512622268566	0.042	0.025	3.55810342057887e-1	no

			1	2			7	change
5302	5302	LRRC17	1.71386591482845e-21	-0.204623685	0.028	0.015	3.74376870435126e-17	no change
5303	5303	TTC31	1.74828744635826e-21	0.100323301370966	0.053	0.033	3.81895909782498e-17	no change
5304	5304	CLP1	1.75004413149797e-21	0.267540056586221	0.036	0.021	3.82279640084417e-17	no change
5305	5305	TTC7B	1.76141135314737e-21	0.521495456605569	0.025	0.012	3.84762695981512e-17	no change
5306	5306	TRAPPC10	1.77256628933185e-21	0.280255836894569	0.052	0.033	3.8719938024165e-17	no change
5307	5307	FXR2	1.78496159685613e-21	0.127989788524937	0.053	0.033	3.89907011217252e-17	no change
5308	5308	TMEM140	1.82775184221386e-21	-1.140233337	0.023	0.039	3.99254112413195e-17	down
5309	5309	ZSCAN26	1.82969234394596e-21	0.365773551089736	0.033	0.018	3.99677995611556e-17	no change
5310	5310	CEP250	1.86343369093108e-21	0.226345040078926	0.051	0.032	4.07048455446985e-17	no change
5311	5311	GAN	1.90182754477563e-21	-0.212808248	0.218	0.17	4.15435208880789e-17	no change
5312	5312	ARNTL2	1.9537990560608e-21	0.268590309655678	0.055	0.035	4.26787865805922e-17	no change
5313	5313	TUT1	1.96129559677349e-21	0.161694891549756	0.041	0.024	4.28425410159202e-17	no change
5314	5314	SMC1B	1.97530392111381e-2	0.45325088759817	0.02	0.009	4.314853885281e-17	no

			1	4				change
5315	5315	RBBP9	2.01678877747287e-21	0.117158845630045	0.056	0.036	4.40547340551175e-17	no change
5316	5316	OPA3	2.02168568457894e-21	-0.130260115	0.138	0.102	4.41617020939424e-17	no change
5317	5317	LPXN	2.04122498381867e-21	0.128068312930257	0.103	0.074	4.4588518546535e-17	no change
5318	5318	SCAMP1-AS1	2.09777290702852e-21	0.25552746858821	0.055	0.035	4.58237513811309e-17	no change
5319	5319	HELB	2.11334105939105e-21	0.39428155171409	0.021	0.01	4.61638221013381e-17	no change
5320	5320	DGKD	2.13023056571136e-21	0.233602209631099	0.042	0.025	4.65327564773989e-17	no change
5321	5321	RORC	2.17740065421155e-21	-0.852413866	0.041	0.061	4.75631398905971e-17	no change
5322	5322	TLE6	2.22465605001986e-21	-0.815460397	0.034	0.053	4.85953867566338e-17	no change
5323	5323	IPCEF1	2.24816905227369e-21	0.416287721133128	0.026	0.013	4.91090047778664e-17	no change
5324	5324	DTL	2.2940224051126e-21	0.493469352432203	0.018	0.008	5.01106254172796e-17	no change
5325	5325	NPAS1	2.30818619981349e-21	-1.155958295	0.012	0.024	5.04200193487258e-17	down
5326	5326	MSC-AS1	2.31269057153211e-21	0.140165186543785	0.019	0.008	5.05184128445473e-17	no change
5327	5327	HSPG2	2.32526860068933e-2	-0.314699306	0.089	0.062	5.07931673134578e-1	no

			1				7	change
5328	5328	CTC-241N9.1	2.38257352351701e-21	0.45454116112901	0.024	0.012	5.20449360477056e-17	no change
5329	5329	LMTK2	2.45346221172142e-21	0.147541593618505	0.055	0.035	5.35934285528426e-17	no change
5330	5330	TLK1	2.6156417928044e-21	-0.138191129	0.218	0.171	5.71360793220192e-17	no change
5331	5331	ARSG	2.69280222673672e-21	0.134026366780432	0.06	0.039	5.8821571840837e-17	no change
5332	5332	GLT8D2	2.78858011632548e-21	-1.714627306	0.011	0.023	6.09137440610138e-17	down
5333	5333	CCDC61	2.81144925975705e-21	0.119477185604686	0.054	0.034	6.14132976301331e-17	no change
5334	5334	ATXN7L3	2.88687386154929e-21	0.120461273712221	0.062	0.041	6.30608726316826e-17	no change
5335	5335	ATF3	2.94202933647893e-21	-0.269449614	0.664	0.677	6.42656888260457e-17	no change
5336	5336	WNK3	2.97961512472026e-21	0.876113473490579	0.013	0.005	6.50867127843893e-17	no change
5337	5337	INSR	3.19509030408395e-21	-0.201104053	0.28	0.222	6.97935526024097e-17	no change
5338	5338	EDRF1	3.26149373885817e-21	0.263626650842888	0.038	0.022	7.12440692316179e-17	no change
5339	5339	MAGI3	3.51455975622836e-21	0.139184047544402	0.042	0.025	7.67720433150522e-17	no change
5340	5340	ZNF808	3.72890395403302e-2	0.32357306625749	0.033	0.018	8.14541779718974e-1	no

			1	9			7	change
5341	5341	ABHD11-AS1	3.89687120649313e-21	-0.965587479	0.026	0.042	8.51232546346359e-17	no change
5342	5342	TTI1	4.14055121343751e-21	0.194225312019969	0.044	0.027	9.04462007063291e-17	no change
5343	5343	ALMS1	4.31502607666089e-21	0.21050700342733	0.04	0.024	9.42574296185805e-17	no change
5344	5344	THSD7A	4.44287718081354e-21	-1.537734225	0.006	0.015	9.7050209137691e-17	down
5345	5345	NUCB1	4.51696834454909e-21	-0.13197067	0.41	0.325	9.86686565183303e-17	no change
5346	5346	RP11-334C17.5	4.53179459721748e-21	0.406120591544483	0.029	0.016	9.89925211816186e-17	no change
5347	5347	TRAPPC11	4.70885037069637e-21	0.173603109155316	0.055	0.035	1.02860127497491e-16	no change
5348	5348	SIDT1-AS1	4.82110910847707e-21	0.460329184036944	0.02	0.009	1.05312307365573e-16	no change
5349	5349	GRPEL2	4.84239217771353e-21	0.190162774343658	0.059	0.039	1.05777214729974e-16	no change
5350	5350	PSMG3-AS1	4.90646214972188e-21	0.120265234790438	0.042	0.025	1.07176759198525e-16	no change
5351	5351	GTPBP10	5.15044665025396e-21	0.105020765557377	0.057	0.037	1.12506356628148e-16	no change
5352	5352	SMAD9	5.21154448446396e-21	0.115130803473434	0.073	0.05	1.13840977718631e-16	no change
5353	5353	MCF2L-AS1	5.25147785289217e-2	0.12622862607590	0.079	0.055	1.14713282218577e-1	no

			1	9			6	change
5354	5354	RBL1	5.27643285533392e-21	0.21106523398297	0.027	0.014	1.15258399291914e-16	no change
5355	5355	DOLPP1	5.40374075464858e-21	0.209624872705612	0.04	0.023	1.18039313044544e-16	no change
5356	5356	RFX7	5.40888632300277e-21	0.167723551408391	0.059	0.038	1.18151712839672e-16	no change
5357	5357	RP1-197B17.3	5.68686646880381e-21	0.237965906244431	0.035	0.02	1.2422391114455e-16	no change
5358	5358	SORBS3	6.00286996919115e-21	-0.1823575	0.058	0.038	1.31126691607011e-16	no change
5359	5359	C17orf80	6.04358391068261e-21	0.337551518275337	0.036	0.021	1.32016046944951e-16	no change
5360	5360	CHRNA5	6.14567885045185e-21	0.191082964929072	0.048	0.03	1.3424620880927e-16	no change
5361	5361	ZNF629	6.60717753573087e-21	0.315246207080832	0.03	0.016	1.44327186090505e-16	no change
5362	5362	C11orf84	6.62091642477097e-21	0.410006202210469	0.031	0.017	1.44627298382697e-16	no change
5363	5363	DOLK	6.75149866763429e-21	0.139798450712255	0.05	0.032	1.47479736895803e-16	no change
5364	5364	IFNAR1	6.84613676424791e-21	-0.114324122	0.274	0.218	1.49547011478231e-16	no change
5365	5365	SMAD1	6.84997333834681e-21	0.130410608229811	0.05	0.032	1.49630817602848e-16	no change
5366	5366	BLM	7.38031239348503e-2	0.76980014891850	0.015	0.006	1.61215543923287e-1	no

			1	2			6	change
5367	5367	RBM38	7.40220462649177e-21	0.135891664412268	0.062	0.041	1.61693757861086e-16	no change
5368	5368	PRRT3-AS1	7.79054924806782e-21	0.267673232857872	0.041	0.025	1.70176757774793e-16	no change
5369	5369	NRGN	7.91264103825604e-21	0.832022511512575	0.016	0.006	1.72843730839665e-16	no change
5370	5370	WDR66	7.9380280693208e-21	0.429007721458148	0.027	0.014	1.73398285146244e-16	no change
5371	5371	HSD17B8	8.23313446240997e-21	-0.100543432	0.209	0.163	1.79844589196883e-16	no change
5372	5372	RP11-500C11.3	8.59484854648604e-21	0.287575506910192	0.032	0.018	1.87745871649441e-16	no change
5373	5373	PTGER4	8.71543556398207e-21	-0.110947256	0.101	0.073	1.90379974459624e-16	no change
5374	5374	AP2S1	8.7803998559735e-21	-0.240872142	0.703	0.64	1.91799054453885e-16	no change
5375	5375	CCDC102A	9.21328952760316e-21	0.265337587927156	0.03	0.016	2.01255096440963e-16	no change
5376	5376	AC012358.8	9.43224196003987e-21	0.505478469156341	0.02	0.009	2.06037893375111e-16	no change
5377	5377	RIPPLY2	9.48325613480003e-21	0.763088204254532	0.016	0.007	2.07152247008572e-16	no change
5378	5378	UBE2Q2	9.57370288825687e-21	-0.109955641	0.194	0.151	2.09127965891083e-16	no change
5379	5379	NSUN3	9.95998415849335e-2	0.10214460959266	0.055	0.035	2.17565893958129e-1	no

			1	9			6	change
5380	5380	ZDHHC23	1.00233014385088e-20	0.208183017650131	0.031	0.017	2.18948996622785e-16	no change
5381	5381	IQCE	1.0156253227558e-20	0.1958159821858	0.046	0.029	2.21853195502776e-16	no change
5382	5382	SRA1	1.02071052043377e-20	-0.132360276	0.342	0.269	2.22964006083552e-16	no change
5383	5383	SWT1	1.03049735616384e-20	0.206556670150173	0.038	0.022	2.25101842480429e-16	no change
5384	5384	COBLL1	1.0518291644458e-20	-0.109860578	0.287	0.228	2.29761562681541e-16	no change
5385	5385	ETV4	1.10039634708431e-20	0.463038887295365	0.038	0.022	2.40370578057098e-16	no change
5386	5386	C18orf54	1.10686473698875e-20	0.607476813857114	0.014	0.005	2.41783533147823e-16	no change
5387	5387	CAP2	1.11215766043698e-20	0.472666759907389	0.029	0.015	2.42939719345854e-16	no change
5388	5388	FOXJ1	1.14925806493071e-20	-1.062728824	0.049	0.069	2.51043931703465e-16	down
5389	5389	PFDN1	1.1758720695445e-20	-0.108804701	0.37	0.294	2.56857494871301e-16	no change
5390	5390	RP1-101G11.3	1.18647669664204e-20	0.711622872862655	0.01	0.003	2.59173969614487e-16	no change
5391	5391	DNAJC3-AS1	1.18979489679715e-20	0.168643362060382	0.044	0.027	2.5989879725637e-16	no change
5392	5392	ZNF114	1.23874706784618e-2	-1.805545008	0.008	0.018	2.70591909500319e-1	down

			0				6	
5393	5393	NSMF	1.2614763854098e-20	0.131183674332936	0.038	0.023	2.75556901628917e-16	no change
5394	5394	FREM2	1.28833921216173e-20	0.704059388279117	0.026	0.014	2.81424817504609e-16	no change
5395	5395	CDC42EP4	1.29514895233706e-20	-0.615232051	0.32	0.329	2.82912337148507e-16	no change
5396	5396	RIMS2	1.30126960827798e-20	0.512675415049954	0.032	0.018	2.84249333232242e-16	no change
5397	5397	GNGT1	1.35910204615066e-20	0.207896086777001	0.026	0.013	2.96882250961151e-16	no change
5398	5398	VPS39	1.38174668127836e-20	0.153601580275176	0.058	0.038	3.01828745058445e-16	no change
5399	5399	AC124944.5	1.38832967368794e-20	-1.258737905	0.014	0.027	3.03266733920395e-16	down
5400	5400	RP11-121C2.2	1.48345663441305e-20	0.637845650959353	0.016	0.006	3.24046267221186e-16	no change
5401	5401	NBPF11	1.50362971130745e-20	0.197467590012333	0.046	0.028	3.28452874137998e-16	no change
5402	5402	ZNF526	1.51138716093586e-20	0.278455797744332	0.033	0.019	3.3014741143483e-16	no change
5403	5403	KISS1	1.51354642257252e-20	-0.735332872	0.087	0.112	3.30619080546742e-16	no change
5404	5404	BMI1	1.54631826298568e-20	-0.116296738	0.196	0.154	3.37777761366591e-16	no change
5405	5405	FAM160B2	1.62792379332776e-2	-0.142963463	0.084	0.059	3.55603673414517e-1	no

			0				6	change
5406	5406	CAMKMT	1.62805887498115e-20	0.123777223823649	0.068	0.046	3.55633180650882e-16	no change
5407	5407	ZNF420	1.66364416403303e-20	0.282394225097117	0.04	0.024	3.63406431191374e-16	no change
5408	5408	OSBP2	1.70271839627955e-20	0.437667552245911	0.024	0.012	3.71941806483306e-16	no change
5409	5409	TAF4B	1.71700881775413e-20	0.375140796391292	0.019	0.008	3.75063406150213e-16	no change
5410	5410	CHRDL1	1.71928281964811e-20	-2.133594106	0.007	0.017	3.75560139123933e-16	down
5411	5411	GUCA2B	1.72494227163164e-20	0.977648220720224	0.022	0.011	3.76796389815216e-16	no change
5412	5412	RP11-498C9.15	1.75002654602141e-20	0.436063645489376	0.018	0.008	3.82275798712917e-16	no change
5413	5413	GKAP1	1.77886498113043e-20	0.291380690581677	0.042	0.025	3.88575266478132e-16	no change
5414	5414	PRR12	1.80844630157663e-20	0.134537301556232	0.045	0.027	3.95037010116399e-16	no change
5415	5415	MYCN	1.81217585957141e-20	1.16037793877686	0.01	0.003	3.95851694764779e-16	up
5416	5416	ZC3H3	1.86119635131412e-20	0.103209417805614	0.052	0.033	4.06559730981056e-16	no change
5417	5417	LINS1	1.94524311489477e-20	0.130091277255873	0.052	0.033	4.24918906017613e-16	no change
5418	5418	ADPRM	1.9736939761549e-20	0.15634024639896	0.044	0.027	4.31133712151277e-1	no

				6			6	change
5419	5419	C17orf96	2.1188690931633e-20	0.151879176452534	0.072	0.049	4.6284576471059e-16	no change
5420	5420	MINCR	2.13036065264289e-20	-0.109982296	0.11	0.081	4.65355980963313e-16	no change
5421	5421	MAATS1	2.2149782885691e-20	0.847307029940828	0.013	0.004	4.83839857355034e-16	no change
5422	5422	AC068831.16	2.23999214574467e-20	0.350167149419374	0.036	0.021	4.89303884316467e-16	no change
5423	5423	CAPN5	2.30528994136198e-20	0.164773762681711	0.09	0.064	5.03567534791111e-16	no change
5424	5424	PDSS1	2.37113544020304e-20	0.29978640165585	0.037	0.021	5.17950825557951e-16	no change
5425	5425	CHST10	2.41121069156092e-20	0.637370954427158	0.013	0.005	5.26704863464568e-16	no change
5426	5426	ADAMTS5	2.42171866453976e-20	-2.027374067	0.009	0.019	5.29000225082065e-16	down
5427	5427	FADS2	2.43365634733991e-20	-0.973739219	0.043	0.062	5.3160789251293e-16	no change
5428	5428	HLA-B	2.45261810267349e-20	-0.185089896	0.946	0.917	5.35749898347998e-16	no change
5429	5429	AP001372.2	2.46786538442897e-20	0.199441814591146	0.056	0.037	5.39080514574665e-16	no change
5430	5430	SIK3	2.60153360994813e-20	0.101213776004412	0.051	0.032	5.68279001757069e-16	no change
5431	5431	DBR1	2.61557030560699e-2	0.10984377107767	0.041	0.025	5.71345177556792e-1	no

			0	7			6	change
5432	5432	MSN	2.66068882412533e-20	-0.212046735	0.082	0.057	5.81200866741936e-16	no change
5433	5433	AP001462.6	2.82891874264608e-20	0.530634392468486	0.023	0.011	6.17949010143611e-16	no change
5434	5434	SDC3	2.87417685058539e-20	0.10368152229355	0.045	0.028	6.27835191241872e-16	no change
5435	5435	IPO11	2.93312035010125e-20	0.209906894257728	0.032	0.018	6.40710809276118e-16	no change
5436	5436	SCUBE2	2.9913306940334e-20	-0.135288242	0.135	0.101	6.53426276804657e-16	no change
5437	5437	MDM2	3.09350659528609e-20	-0.576368759	0.391	0.394	6.75745580674293e-16	no change
5438	5438	NEK5	3.10239630988857e-20	0.571235620099301	0.017	0.007	6.7768744993206e-16	no change
5439	5439	NDST1	3.20931141564619e-20	0.24969169691493	0.034	0.02	7.01041985633754e-16	no change
5440	5440	PICALM	3.31118990722228e-20	-0.149689047	0.231	0.181	7.23296323333634e-16	no change
5441	5441	GAS1	3.48261398362275e-20	-1.727172861	0.013	0.026	7.60742198582554e-16	down
5442	5442	MTSS1	3.4978816268587e-20	-0.137235429	0.175	0.135	7.64077262571014e-16	no change
5443	5443	SLC37A3	3.53984355065187e-20	-0.155470902	0.124	0.093	7.73243425204395e-16	no change
5444	5444	DSC2	3.54345374489401e-2	-0.110176685	0.061	0.04	7.74032036034648e-1	no

			0				6	change
5445	5445	RNF214	3.54589270419503e-20	0.195486953399181	0.07	0.048	7.74564802304363e-16	no change
5446	5446	SGSM3	3.58797347207065e-20	-0.120155488	0.188	0.145	7.83756925239113e-16	no change
5447	5447	TDRD5	3.75055077995327e-20	1.31670798199004	0.011	0.003	8.19270312372992e-16	up
5448	5448	SPINT2	3.76652414034542e-20	-0.167171362	0.891	0.786	8.22759533217054e-16	no change
5449	5449	SLC5A12	3.81919864312272e-20	0.471659293282578	0.017	0.007	8.34265751603727e-16	no change
5450	5450	DERL1	3.84182021359953e-20	-0.133538043	0.242	0.191	8.39207207458681e-16	no change
5451	5451	ZNF200	3.85042586074132e-20	0.26434592183607	0.033	0.019	8.41087025020333e-16	no change
5452	5452	UPF2	4.02290557674685e-20	-0.105214475	0.385	0.311	8.78763494184583e-16	no change
5453	5453	PLA2G4A	4.03847061904993e-20	-0.101904517	0.062	0.041	8.82163522025266e-16	no change
5454	5454	FRGCA	4.04721447156864e-20	0.440883024717971	0.032	0.018	8.84073529169453e-16	no change
5455	5455	MYEOV2	4.07802530703234e-20	-0.189247567	0.807	0.722	8.90803848068145e-16	no change
5456	5456	HNF4G	4.1536131009665e-20	0.752141214576147	0.012	0.004	9.07315245775123e-16	no change
5457	5457	ACCS	4.31892284253461e-2	0.18270477955507	0.039	0.023	9.43425505723259e-1	no

			0	2			6	change
5458	5458	RP4-625H18.2	4.35985090256502e-20	0.627179685458394	0.022	0.011	9.52365831156302e-16	no change
5459	5459	CCNY	4.52115744701613e-20	-0.126442479	0.155	0.119	9.87601632726204e-16	no change
5460	5460	RP11-149I23.3	4.53105141587208e-20	-2.072863784	0.005	0.013	9.89762871283097e-16	down
5461	5461	ERGIC1	4.56107441473814e-20	-0.112723695	0.228	0.18	9.96321095155399e-16	no change
5462	5462	CTC-529I10.2	4.69995571384555e-20	0.93643017989414	0.012	0.004	1.02665832613242e-15	no change
5463	5463	KDM5B	4.70351318775131e-20	-0.130827065	0.279	0.223	1.0274354207324e-15	no change
5464	5464	CSF1	4.86656601721355e-20	-1.197310878	0.034	0.051	1.06305268080013e-15	down
5465	5465	PRNP	4.97919820185886e-20	-0.216883664	0.325	0.257	1.08765605521405e-15	no change
5466	5466	C3orf67	5.12381195856485e-20	0.596931406011034	0.017	0.007	1.11924548422891e-15	no change
5467	5467	TTC4	5.12584775296102e-20	0.3785190991733	0.019	0.008	1.1196901831568e-15	no change
5468	5468	BANP	5.28002114527804e-20	0.134421671361379	0.079	0.056	1.15336781897454e-15	no change
5469	5469	ATP5L2	5.30282239716053e-20	0.150786398859017	0.048	0.03	1.15834852443575e-15	no change
5470	5470	TRIM23	5.65804011407477e-2	0.14042963492502	0.038	0.022	1.23594228251849e-1	no

			0	2			5	change
5471	5471	AKNA	5.68984275597374e-20	0.147127616459277	0.04	0.024	1.2428892516149e-15	no change
5472	5472	MKNK1	5.72591463183823e-20	-0.135314658	0.19	0.148	1.25076879217874e-15	no change
5473	5473	PLA2G2A	5.76694036294814e-20	-1.210991143	0.132	0.157	1.25973045288239e-15	down
5474	5474	CPNE7	5.88834751707712e-20	0.678270945306749	0.012	0.004	1.28625063163033e-15	no change
5475	5475	ARFRP1	5.94592183359385e-20	-0.131317065	0.171	0.132	1.29882716533024e-15	no change
5476	5476	PC	6.28549304526754e-20	0.630154765362769	0.016	0.007	1.37300310080824e-15	no change
5477	5477	ARRDC4	6.29985017338562e-20	0.305240432091325	0.061	0.041	1.37613927187436e-15	no change
5478	5478	PLCH1	6.39577217909999e-20	0.444027727764814	0.019	0.008	1.3970924748026e-15	no change
5479	5479	TMPRSS13	6.41512538631575e-20	-0.919088269	0.017	0.03	1.40131998938681e-15	no change
5480	5480	GPATCH1	6.52989954059703e-20	0.163254803644489	0.046	0.029	1.42639125564801e-15	no change
5481	5481	PPP2CB	6.80040311394393e-20	-0.162533626	0.238	0.187	1.48548005620991e-15	no change
5482	5482	RP11-809C18.3	6.9052679441601e-20	1.07424534593646	0.011	0.003	1.50838672972233e-15	up
5483	5483	GLIPR1L2	6.95265020774125e-2	0.65672789460916	0.016	0.006	1.518736911379e-15	no

			0	1				change
5484	5484	EARS2	6.98430196735024e-20	0.145993134355113	0.041	0.025	1.52565092174799e-15	no change
5485	5485	STRIP1	6.98831342780762e-20	0.104850567863626	0.063	0.043	1.5265271851703e-15	no change
5486	5486	LCN15	7.09788584212676e-20	-0.632695703	0.043	0.063	1.55046218335417e-15	no change
5487	5487	DBF4B	7.12846658340873e-20	0.674959243819695	0.016	0.006	1.5571422404798e-15	no change
5488	5488	VANGL2	7.14105571772163e-20	0.265056155030994	0.035	0.02	1.55989221097911e-15	no change
5489	5489	PCDHB15	7.34028893899959e-20	0.470969223210308	0.018	0.008	1.60341271583507e-15	no change
5490	5490	IFIT3	7.45267313212302e-20	-0.995371715	0.065	0.086	1.62796191898095e-15	no change
5491	5491	PCGF6	7.60554349230713e-20	0.102198753368341	0.05	0.032	1.66135492045957e-15	no change
5492	5492	C14orf93	7.86985879519562e-20	0.308571429357773	0.039	0.023	1.71909195522253e-15	no change
5493	5493	RFX1	8.56079347553163e-20	-0.117735959	0.107	0.078	1.87001972679513e-15	no change
5494	5494	COX10	8.67871355265717e-20	0.179817738149739	0.064	0.043	1.89577818844243e-15	no change
5495	5495	RARRES1	8.72434470298623e-20	-1.3173671	0.095	0.119	1.90574585692031e-15	down
5496	5496	METTL22	8.73376670072645e-2	0.13876915351764	0.049	0.031	1.90780399810669e-1	no

			0	7			5	change
5497	5497	KCND3	9.04419576701521e-20	0.141442004226263	0.022	0.011	1.9756141233468e-15	no change
5498	5498	RP11-285E9.6	9.30375093455097e-20	0.131956862703727	0.052	0.033	2.03231135414331e-15	no change
5499	5499	PTCD2	9.56442481830839e-20	0.274390995861485	0.03	0.017	2.08925295731129e-15	no change
5500	5500	MAFK	9.59855064579707e-20	-0.1300909	0.248	0.197	2.09670740306791e-15	no change
5501	5501	KCTD2	9.72006297455568e-20	0.252828111612248	0.042	0.026	2.12325055616194e-15	no change
5502	5502	LMAN2L	9.99912307373658e-20	0.13126085969839	0.055	0.036	2.18420844422702e-15	no change
5503	5503	RP3-453C12.15	1.02867177720107e-19	-1.642542901	0.005	0.013	2.24703063011802e-15	down
5504	5504	FKBP14	1.03450628300768e-19	-0.117777566	0.094	0.068	2.25977552460197e-15	no change
5505	5505	RBM7	1.04678204864296e-19	-0.13882459	0.286	0.226	2.28659070705568e-15	no change
5506	5506	SLC39A4	1.11262312909844e-19	-0.149993086	0.414	0.333	2.43041396320264e-15	no change
5507	5507	DGKQ	1.11859549567627e-19	0.294245321718589	0.046	0.029	2.44346000075525e-15	no change
5508	5508	SOGA1	1.1240421854386e-19	0.200322074461445	0.032	0.018	2.45535774987208e-15	no change
5509	5509	TMEM263	1.12635072987189e-1	-0.154644503	0.177	0.137	2.46040053433215e-1	no

			9				5	change
5510	5510	CEP162	1.2039597381417e-19	0.21523083198088	0.037	0.022	2.62992965199672e-15	no change
5511	5511	FOXO1	1.23199516032189e-19	-0.197417753	0.077	0.053	2.69117022820715e-15	no change
5512	5512	TAB1	1.25293006276771e-19	0.18346234534624	0.044	0.027	2.7369004291098e-15	no change
5513	5513	SLC37A2	1.27928741199943e-19	0.133028161056997	0.049	0.031	2.79447542277156e-15	no change
5514	5514	ZFPM1	1.30738151806987e-19	0.517508373348851	0.02	0.009	2.85584418807183e-15	no change
5515	5515	AC010969.1	1.3371521751401e-19	0.89571278617165	0.01	0.003	2.92087521137603e-15	no change
5516	5516	MUM1L1	1.37582760995275e-19	0.443704745246428	0.032	0.018	3.00535783118078e-15	no change
5517	5517	NOSTRIN	1.38711773858359e-19	-1.082153947	0.022	0.037	3.030019988162e-15	down
5518	5518	MIPOL1	1.40656863059043e-19	0.13106323998537	0.065	0.044	3.07250851666173e-15	no change
5519	5519	RP11-701H24.9	1.42123346015816e-19	0.670272653653154	0.015	0.006	3.10454237036948e-15	no change
5520	5520	ARHGEF11	1.43549374263764e-19	0.272466422381057	0.036	0.021	3.13569253141767e-15	no change
5521	5521	ZNF282	1.44983296071293e-19	0.225069776749627	0.049	0.031	3.16701511938133e-15	no change
5522	5522	YEATS2	1.45900192516068e-1	0.29097297615668	0.041	0.025	3.18704380532098e-1	no

			9	1			5	change
5523	5523	C5orf34	1.51591296133352e-19	0.800497781322963	0.014	0.005	3.31136027273693e-15	no change
5524	5524	MIDN	1.56453608272566e-19	-0.286308929	0.597	0.567	3.41757261910593e-15	no change
5525	5525	CPNE2	1.60378906332375e-19	0.221272591252764	0.029	0.016	3.50331682992441e-15	no change
5526	5526	MIPEP	1.62793153285387e-19	0.169501763870482	0.045	0.028	3.556053640366e-15	no change
5527	5527	NUDT11	1.6482896926577e-19	0.706267398303198	0.012	0.004	3.60052400464149e-15	no change
5528	5528	LRRC61	1.74597650019277e-19	-0.678221542	0.156	0.18	3.81391106702109e-15	no change
5529	5529	TMEM187	1.7627054293649e-19	0.155010471513543	0.058	0.038	3.85045373990469e-15	no change
5530	5530	CKMT1A	1.79177970741871e-19	0.101379281107374	0.057	0.038	3.91396359288542e-15	no change
5531	5531	ACTRT3	1.82596956935464e-19	0.549995675893004	0.013	0.005	3.98864792729827e-15	no change
5532	5532	PIP	1.85196524815198e-19	-2.312904464	0.004	0.012	4.04543288806319e-15	down
5533	5533	RP11-112J3.16	1.85609336813921e-19	0.775496577297412	0.017	0.008	4.05445035336328e-15	no change
5534	5534	USP28	1.91642430912413e-19	0.170690853633129	0.044	0.028	4.18623726085075e-15	no change
5535	5535	PGM2L1	1.99521601830942e-1	-0.116268766	0.078	0.055	4.35834987039509e-1	no

			9				5	change
5536	5536	UPP1	2.04390978117992e-19	-0.562695294	0.278	0.299	4.46471652600943e-15	no change
5537	5537	GLIS3	2.0852730687056e-19	0.10849013975104	0.04	0.024	4.55507049128052e-15	no change
5538	5538	ANGPT2	2.14539067872839e-19	-0.466928079	0.025	0.013	4.6863913986143e-15	no change
5539	5539	IGHG3	2.15864172869935e-19	-3.408958823	0.084	0.107	4.71533699217086e-15	down
5540	5540	RP11-134G8.7	2.17590669951768e-19	0.520558995183253	0.017	0.007	4.75305059442641e-15	no change
5541	5541	ZNRF3	2.30356361702172e-19	0.363176108471631	0.026	0.013	5.03190436502225e-15	no change
5542	5542	PARP11	2.32633757175995e-19	0.174820422000865	0.041	0.025	5.08165179175242e-15	no change
5543	5543	SLC24A3	2.34754270625164e-19	0.518515563908432	0.013	0.005	5.12797228753609e-15	no change
5544	5544	EDIL3	2.39075617706257e-19	-1.64604783	0.012	0.023	5.22236779317548e-15	down
5545	5545	BICD1	2.40526049842536e-19	0.184005280234099	0.048	0.03	5.25405103276035e-15	no change
5546	5546	SPATA5L1	2.43556173446454e-19	0.177831764989921	0.042	0.026	5.32024105276434e-15	no change
5547	5547	ZNF324	2.46120346532365e-19	0.235246601478064	0.041	0.025	5.37625284965297e-15	no change
5548	5548	GCSAM	2.52410634184752e-1	0.86550789808345	0.013	0.005	5.51365789313173e-1	no

			9				5	change
5549	5549	KBTBD6	2.597235477689e-19	0.279453379098682	0.033	0.019	5.67340117746386e-15	no change
5550	5550	IQCG	2.62248844107606e-19	0.136524535687951	0.052	0.034	5.72856375068654e-15	no change
5551	5551	LONRF1	2.62668272926473e-19	-0.123724958	0.093	0.067	5.73772575380588e-15	no change
5552	5552	PIK3R4	2.65439516720983e-19	0.217963720134604	0.039	0.024	5.79826080325315e-15	no change
5553	5553	HOXC13-AS	2.83868154175838e-19	0.517574849479406	0.031	0.018	6.20081595981701e-15	no change
5554	5554	HLA-C	3.15250817301868e-19	-0.152108847	0.912	0.869	6.886338853142e-15	no change
5555	5555	LDLRAP1	3.15284239649856e-19	0.162983068301229	0.072	0.05	6.88706893091146e-15	no change
5556	5556	TOR4A	3.44659978803961e-19	0.233747470846855	0.039	0.024	7.52875257699373e-15	no change
5557	5557	ZNF213-AS1	3.61276477845505e-19	0.146204288158001	0.039	0.023	7.8917233820572e-15	no change
5558	5558	SERINC1	3.85430302450056e-19	-0.189446289	0.278	0.22	8.41933952671901e-15	no change
5559	5559	CENPO	3.89450873026544e-19	0.499332407607772	0.015	0.006	8.50716487039182e-15	no change
5560	5560	CRYBG3	3.97734998182511e-19	-0.125223808	0.075	0.052	8.68812330029876e-15	no change
5561	5561	SLC2A5	4.24868898045924e-1	0.54937531336130	0.015	0.006	9.28083620891517e-1	no

			9	9			5	change
5562	5562	TP73	4.46902425971918e-19	0.899668514465206	0.01	0.003	9.76213659293057e-15	no change
5563	5563	NACC2	4.64269090426988e-19	0.238949666302016	0.033	0.019	1.01414940112871e-14	no change
5564	5564	FEZ2	4.6503973123316e-19	-0.13748319	0.325	0.259	1.01583278890572e-14	no change
5565	5565	AGFG1	4.82577167647104e-19	-0.112255498	0.14	0.107	1.05414156500833e-14	no change
5566	5566	CTC-503J8.4	5.065978055447e-19	0.48417286277195	0.02	0.01	1.10661224643184e-14	no change
5567	5567	COCH	5.18352114563413e-19	0.276499766147095	0.027	0.014	1.13228835905232e-14	no change
5568	5568	CD52	5.41711104362212e-19	0.359407326348068	0.069	0.047	1.18331373636882e-14	no change
5569	5569	SEC23IP	5.41847973648927e-19	-0.104864827	0.12	0.09	1.18361271363872e-14	no change
5570	5570	TBC1D8	5.44940316593625e-19	0.115070598393391	0.044	0.027	1.19036762756711e-14	no change
5571	5571	ZFYVE27	5.77684584662321e-19	0.105576234288914	0.059	0.04	1.26189420673637e-14	no change
5572	5572	ZNF783	6.02247149876945e-19	0.153579550460112	0.036	0.021	1.3155486741912e-14	no change
5573	5573	ZNF264	6.11927447378327e-19	-0.103635163	0.095	0.069	1.33669431605322e-14	no change
5574	5574	SLAIN1	6.15212664625879e-1	0.51768252570906	0.017	0.007	1.34387054460877e-1	no

			9	5			4	change
5575	5575	ARSK	6.33270334411913e-19	0.132499187830768	0.035	0.021	1.38331571848938e-14	no change
5576	5576	CDC37L1	6.37287183096738e-19	-0.207993006	0.159	0.122	1.39209012275652e-14	no change
5577	5577	ZNF330	6.52066930618629e-19	-0.100199021	0.181	0.142	1.42437500324333e-14	no change
5578	5578	KLHL22	6.65855492734569e-19	0.328152824043333	0.034	0.02	1.45449473832939e-14	no change
5579	5579	RP11-418J17.1	7.31933965192539e-19	0.3598690492993	0.023	0.012	1.59883655356658e-14	no change
5580	5580	RNF165	7.39667421867126e-19	0.849751228789704	0.011	0.004	1.61572951632655e-14	no change
5581	5581	DHX35	7.52991808820533e-19	0.308475460264855	0.022	0.011	1.64483530718757e-14	no change
5582	5582	B3GAT1	7.59606529729376e-19	0.814370802636811	0.011	0.004	1.65928450354085e-14	no change
5583	5583	INTS9	7.66613808849146e-19	0.228083412254493	0.03	0.017	1.67459120405007e-14	no change
5584	5584	RINT1	7.70101934117369e-19	0.101660980670914	0.05	0.032	1.68221066488598e-14	no change
5585	5585	TRMT5	7.76861274395311e-19	0.126786221064916	0.051	0.033	1.69697576778912e-14	no change
5586	5586	TTC21B	7.85249987230153e-19	0.19524464701965	0.038	0.023	1.71530007210555e-14	no change
5587	5587	ZNF658	7.90981610738529e-1	0.68844366419591	0.015	0.006	1.72782023049724e-1	no

			9	3			4	change
5588	5588	SOX7	8.02115270192932e-19	0.139522960499281	0.034	0.02	1.75214059620944e-14	no change
5589	5589	HSPA8	8.04462574458714e-19	-0.318918462	0.884	0.815	1.75726804764761e-14	no change
5590	5590	SFXN2	8.10206767015437e-19	0.355307887633626	0.025	0.013	1.76981566186852e-14	no change
5591	5591	VMP1	8.12590498899813e-19	-0.255980478	0.771	0.712	1.77502268579675e-14	no change
5592	5592	ZMYM6	8.20562855051439e-19	0.12434660633872	0.052	0.034	1.79243750057436e-14	no change
5593	5593	RP11-722G7.1	8.21069062446409e-19	0.816789570096621	0.013	0.005	1.79354326000794e-14	no change
5594	5594	NUP107	8.36479264929085e-19	-0.28196206	0.219	0.172	1.82720530631109e-14	no change
5595	5595	NFKB1	8.61777037763688e-19	-0.212335949	0.245	0.194	1.882465761291e-14	no change
5596	5596	MAL2	8.76122399167857e-19	-0.276178978	0.541	0.424	1.91380176874227e-14	no change
5597	5597	RILPL1	9.35886930256166e-19	0.276812965328125	0.032	0.018	2.04435141045157e-14	no change
5598	5598	BNIPL	9.38655337700114e-19	-0.700571082	0.047	0.066	2.05039871967213e-14	no change
5599	5599	CTD-3184A7.4	9.60066724792949e-19	0.101715432971876	0.07	0.049	2.09716975363772e-14	no change
5600	5600	RP11-84G21.1	9.63078082141143e-1	0.88128463917284	0.013	0.005	2.10374776262911e-1	no

			9	7			4	change
5601	5601	CELF2	1.02188354972936e-18	-1.168108403	0.037	0.054	2.23220242602881e-14	down
5602	5602	SBSN	1.04316317304134e-18	-0.679268633	0.035	0.052	2.27868563519151e-14	no change
5603	5603	ECHDC3	1.04529626058601e-18	0.251053546877505	0.043	0.026	2.28334515162409e-14	no change
5604	5604	DCLK1	1.08182507525017e-18	-1.850109418	0.01	0.02	2.36313869437648e-14	down
5605	5605	RP11-67L2.2	1.13968088901552e-18	0.59514918318157	0.019	0.009	2.4895189339655e-14	no change
5606	5606	PRKCDBP	1.17720546737165e-18	-1.234167386	0.061	0.08	2.57148762292664e-14	down
5607	5607	KIAA1958	1.18368799562708e-18	0.444051263807326	0.023	0.012	2.58564805764778e-14	no change
5608	5608	RP11-197K6.1	1.18789333814261e-18	-0.896179039	0.015	0.027	2.59483420783871e-14	no change
5609	5609	ZNF48	1.21589696298167e-18	0.35565681164601	0.027	0.015	2.65600532593716e-14	no change
5610	5610	ITPKB	1.27221604436016e-18	0.309847605009004	0.035	0.02	2.77902872730033e-14	no change
5611	5611	TTC12	1.29653448636092e-18	0.228969440359867	0.03	0.017	2.8321499320068e-14	no change
5612	5612	ZNF391	1.32191361326976e-18	0.42547486536396	0.021	0.01	2.88758809682646e-14	no change
5613	5613	PMAIP1	1.32425249399309e-1	-0.46454281	0.519	0.502	2.8926971478785e-14	no

			8					change
5614	5614	MAP3K9	1.3259116720077e-18	0.197556694248965	0.044	0.028	2.89632145633362e-14	no change
5615	5615	SPTBN1	1.38676276016965e-18	-0.165688345	0.366	0.294	3.02924457331459e-14	no change
5616	5616	MBNL2	1.46893934560078e-18	-0.129991402	0.218	0.173	3.20875110653033e-14	no change
5617	5617	FGFBP1	1.47644724929752e-18	0.16656364318808	0.046	0.03	3.2251513713655e-14	no change
5618	5618	RNASE1	1.50381817980154e-18	-1.31847247	0.045	0.064	3.28494043195849e-14	down
5619	5619	CTB-193M12.5	1.51196304971528e-18	0.565257774915643	0.019	0.009	3.30273208579805e-14	no change
5620	5620	ZBTB40	1.51434676491801e-18	0.380720111073748	0.034	0.02	3.30793907328691e-14	no change
5621	5621	PNPO	1.51881677815828e-18	0.12063860729026	0.065	0.045	3.31770337020895e-14	no change
5622	5622	SH2D2A	1.58402323733325e-18	0.43948283249853	0.027	0.015	3.46014035963075e-14	no change
5623	5623	PLPP3	1.58788412665598e-18	-1.077655859	0.147	0.169	3.46857408626731e-14	down
5624	5624	LIN9	1.62317141369044e-18	0.546429815840697	0.018	0.008	3.54565563606539e-14	no change
5625	5625	ECM2	1.65598325524763e-18	-1.989845306	0.008	0.018	3.61732982276293e-14	down
5626	5626	CH507-42P11.8	1.68075139001954e-1	0.33005604952065	0.029	0.016	3.67143333635868e-1	no

			8	1			4	change
5627	5627	RDH5	1.71355119999244e-18	0.11850881331097	0.032	0.019	3.74308124126348e-14	no change
5628	5628	RFXAP	1.75487868688857e-18	0.152160584386991	0.035	0.021	3.8333570036394e-14	no change
5629	5629	CEP76	1.88748620585027e-18	0.351343559156064	0.036	0.022	4.12302486805934e-14	no change
5630	5630	TBC1D24	1.90330676462037e-18	0.320296659893542	0.032	0.019	4.15758329663673e-14	no change
5631	5631	PPP1R16A	1.95067478605596e-18	-0.156182849	0.152	0.117	4.26105400266064e-14	no change
5632	5632	STK32A	2.00100559388779e-18	0.157771022408775	0.051	0.033	4.37099661928849e-14	no change
5633	5633	RNF146	2.00339185638614e-18	-0.138842565	0.14	0.107	4.37620917108989e-14	no change
5634	5634	RECK	2.02464129136671e-18	-1.468014711	0.019	0.032	4.42262643686143e-14	down
5635	5635	SBF1	2.03005543025065e-18	0.103760215073833	0.052	0.034	4.43445308183952e-14	no change
5636	5636	MIR99AHG	2.03173190873231e-18	-1.902557297	0.008	0.017	4.43811518143487e-14	down
5637	5637	MEGF8	2.04166258505995e-18	0.223017135442833	0.032	0.019	4.45980775080495e-14	no change
5638	5638	ITGAV	2.05186536713945e-18	-0.127903341	0.304	0.245	4.48209470797942e-14	no change
5639	5639	C2orf74	2.11187209743419e-1	0.12551520486334	0.035	0.021	4.61317340963524e-1	no

			8	2			4	change
5640	5640	HOXA-AS3	2.13586532026966e-18	0.198253944927882	0.026	0.014	4.66558420559706e-14	no change
5641	5641	ARHGAP21	2.14007830082704e-18	-0.169695853	0.106	0.078	4.67478704032658e-14	no change
5642	5642	FGD3	2.16811240794574e-18	0.15601840492498	0.072	0.051	4.73602474391668e-14	no change
5643	5643	FXYD6	2.27078506824594e-18	-1.259204621	0.019	0.032	4.96030290307643e-14	down
5644	5644	EIF3C	2.32310874850875e-18	0.171627716558607	0.034	0.02	5.07459875024251e-14	no change
5645	5645	NTN4	2.37485521081545e-18	-0.233095181	0.179	0.139	5.18763372250527e-14	no change
5646	5646	ARF6	2.42364482239085e-18	-0.230698783	0.777	0.701	5.29420975003056e-14	no change
5647	5647	HSPA1A	2.45999937441307e-18	0.117033344052754	0.561	0.473	5.3736226334679e-14	no change
5648	5648	POGK	2.72255930192596e-18	-0.108378264	0.223	0.177	5.94715853912707e-14	no change
5649	5649	NEO1	2.73239991020451e-18	0.144035958281244	0.043	0.027	5.96865436385074e-14	no change
5650	5650	ESYT2	2.770044784724e-18	-0.136455207	0.144	0.111	6.05088582775111e-14	no change
5651	5651	PLAGL2	2.79596221551151e-18	0.122134746376701	0.059	0.04	6.10749986356333e-14	no change
5652	5652	FHOD3	2.83572826048473e-1	0.13853240492197	0.048	0.031	6.19436481220285e-1	no

			8	2			4	change
5653	5653	DBN1	2.86254166773258e-18	-0.348625622	0.046	0.029	6.25293601899504e-14	no change
5654	5654	CTD-2325P2.4	2.89772130826943e-18	-0.797016619	0.037	0.054	6.32978242578375e-14	no change
5655	5655	C2CD3	2.93245567862124e-18	0.281858646798896	0.03	0.017	6.40565618438024e-14	no change
5656	5656	ACER2	2.96728141950737e-18	-0.858597718	0.027	0.043	6.4817295327719e-14	no change
5657	5657	ZNF496	2.98143072513289e-18	0.107575466308223	0.031	0.018	6.51263727598028e-14	no change
5658	5658	PSMD6	3.19137027211735e-18	-0.109383863	0.259	0.208	6.97122922241314e-14	no change
5659	5659	DDTL	3.21136146934208e-18	0.624385401587443	0.017	0.008	7.01489799363084e-14	no change
5660	5660	CASKIN2	3.25770883633878e-18	0.217405165088845	0.036	0.022	7.11613918209843e-14	no change
5661	5661	IL7	3.37544143953157e-18	0.155379227603132	0.039	0.024	7.37331428051276e-14	no change
5662	5662	COMT	3.40659170356974e-18	-0.205709585	0.735	0.664	7.44135891727774e-14	no change
5663	5663	RNF2	3.43435431824494e-18	-0.208570692	0.244	0.194	7.50200357277425e-14	no change
5664	5664	SESN3	3.64135053617374e-18	-0.106771897	0.178	0.139	7.95416611121792e-14	no change
5665	5665	ZNF280C	3.78027863557643e-1	0.37804575580347	0.02	0.009	8.25764065155315e-1	no

			8	1			4	change
5666	5666	ZNF730	3.81375575903957e-18	1.05692818745483	0.012	0.005	8.33076808004605e-14	up
5667	5667	RECQL4	4.02746290344134e-18	0.577435998274325	0.014	0.006	8.79758996627727e-14	no change
5668	5668	RP11-440D17.3	4.0888431895477e-18	0.498149113815282	0.021	0.01	8.93166906324801e-14	no change
5669	5669	DENND1A	4.15571153897839e-18	0.360988499403946	0.02	0.01	9.0777362857444e-14	no change
5670	5670	NBPF12	4.17262227218771e-18	0.237215044751969	0.04	0.025	9.11467609136683e-14	no change
5671	5671	HHIP	4.26482309670515e-18	-2.068705028	0.004	0.011	9.31607957244272e-14	down
5672	5672	PRUNE	4.39003105442375e-18	0.108795081129042	0.066	0.046	9.58958383528324e-14	no change
5673	5673	PARG	4.46021296282421e-18	0.145091955549053	0.054	0.036	9.74288919599321e-14	no change
5674	5674	NRBF2	4.49764083875476e-18	-0.118379653	0.209	0.166	9.8246466481759e-14	no change
5675	5675	SYNGR1	4.56321050598427e-18	-0.598183297	0.184	0.207	9.96787702927204e-14	no change
5676	5676	HHEX	4.6846828049672e-18	0.182268979731461	0.029	0.016	1.02332211191704e-13	no change
5677	5677	C6orf52	4.73484142410103e-18	0.400978498561638	0.027	0.015	1.03427876068063e-13	no change
5678	5678	ZNF641	4.81204758579605e-1	0.10150184554444	0.042	0.027	1.05114367464129e-1	no

			8	6			3	change
5679	5679	NUDT16	4.88430330835377e-18	-0.144248579	0.199	0.157	1.0669272146768e-13	no change
5680	5680	NRAV	4.9516655227636e-18	0.208311348007802	0.057	0.039	1.08164181679248e-13	no change
5681	5681	GGA3	5.21287965378492e-18	0.149233524796523	0.055	0.037	1.13870143157278e-13	no change
5682	5682	ZBTB21	5.45326043393294e-18	-0.129467563	0.084	0.061	1.19121020918831e-13	no change
5683	5683	PLTP	5.90153142665466e-18	-0.851230695	0.16	0.181	1.28913052483844e-13	no change
5684	5684	LINC01607	5.93449040926129e-18	0.531234528020453	0.016	0.007	1.29633008499904e-13	no change
5685	5685	ATP6V0A2	6.38888615242506e-18	0.117307839278106	0.06	0.041	1.39558829113573e-13	no change
5686	5686	CYP4X1	6.52754226597586e-18	0.202100878831148	0.039	0.024	1.42587633257977e-13	no change
5687	5687	ARHGAP18	6.59593105771952e-18	-0.13021872	0.122	0.092	1.44081518024825e-13	no change
5688	5688	PHF1	6.70001892041376e-18	-0.110355639	0.191	0.15	1.46355213297518e-13	no change
5689	5689	RBAK	6.75371953932869e-18	0.214360709560099	0.029	0.017	1.47528249617096e-13	no change
5690	5690	UTS2	7.0713660787568e-18	0.495862701006511	0.021	0.011	1.54466920624364e-13	no change
5691	5691	RBM26-AS1	7.18239875640117e-1	0.44355224049570	0.022	0.011	1.56892318434827e-1	no

			8	2			3 change
5692	5692	AXIN1	7.23618978035048e-18	0.162455448726337	0.048	0.032	1.58067329561976e-13	no change
5693	5693	CAPZA1	7.25868308566541e-18	-0.12143495	0.573	0.462	1.58558673323275e-13	no change
5694	5694	ZNF134	7.32278082571758e-18	0.170205298326148	0.054	0.036	1.59958824356975e-13	no change
5695	5695	FDXACB1	7.34269697749833e-18	0.388590864479004	0.022	0.011	1.60393872776474e-13	no change
5696	5696	AXIN2	7.44362186032497e-18	0.482570269480086	0.016	0.007	1.62598475916939e-13	no change
5697	5697	TMEM106A	7.4832511659682e-18	0.212928539812295	0.016	0.007	1.63464138469409e-13	no change
5698	5698	PYGO2	7.55144496936645e-18	0.183101360243288	0.073	0.052	1.64953763910841e-13	no change
5699	5699	LRRN4CL	7.64713210249149e-18	-2.040784273	0.007	0.016	1.67043953646824e-13	down
5700	5700	HILPDA	7.6676895896874e-18	-0.814261092	0.31	0.318	1.67493011397132e-13	no change
5701	5701	RAVER2	7.8825219131774e-18	0.423362696164738	0.031	0.018	1.72185808671447e-13	no change
5702	5702	CTC-534A2.2	7.90690019277156e-18	0.13691080009563	0.034	0.02	1.72718327810902e-13	no change
5703	5703	LACTB	8.26427403940459e-18	-0.11829815	0.192	0.152	1.80524802116754e-13	no change
5704	5704	AK5	8.4319508012761e-18	0.61880790170713	0.013	0.005	1.84187533303075e-1	no

				2			3	change
5705	5705	VPS9D1-AS1	8.66288676867782e-18	0.795225494689453	0.016	0.007	1.89232098574998e-13	no change
5706	5706	CTC-444N24.8	8.74347824585181e-18	0.163157900684139	0.028	0.016	1.90992538802387e-13	no change
5707	5707	RP11-157P1.4	9.16282212178251e-18	0.224371120496754	0.027	0.015	2.00152686428217e-13	no change
5708	5708	ZNF236	9.19552543298216e-18	0.160402409578564	0.048	0.031	2.00867057558062e-13	no change
5709	5709	DEPDC5	9.55036201099125e-18	0.258235059585041	0.028	0.016	2.08618107768093e-13	no change
5710	5710	C1QTNF7	9.59999587798195e-18	-1.739074843	0.009	0.019	2.09702309958638e-13	down
5711	5711	SDR39U1	9.7207564054578e-18	-0.100715407	0.183	0.144	2.1234020292082e-13	no change
5712	5712	DNAJC22	9.79114387887327e-18	0.822214104009143	0.013	0.005	2.13877746890108e-13	no change
5713	5713	CLPB	9.91029722004505e-18	0.10632584694904	0.045	0.029	2.16480532474664e-13	no change
5714	5714	ABCA8	1.04321114082029e-17	-1.175603969	0.026	0.04	2.27879041600785e-13	down
5715	5715	CXorf23	1.05218881072183e-17	0.114449437370848	0.047	0.03	2.29840123814076e-13	no change
5716	5716	RAB38	1.06065370571291e-17	-0.144983431	0.329	0.266	2.31689195475928e-13	no change
5717	5717	MESDC1	1.06798009547114e-1	-0.120252459	0.29	0.234	2.33289572054716e-1	no

			7				3	change
5718	5718	FAM83G	1.06842791393454e-17	0.389934576522612	0.023	0.012	2.3338739351986e-13	no change
5719	5719	RP11-95I16.6	1.08111788459246e-17	0.503111983117109	0.017	0.008	2.36159390710376e-13	no change
5720	5720	PKHD1	1.1413053047607e-17	0.332088250207845	0.024	0.013	2.49306730771928e-13	no change
5721	5721	SLC4A11	1.15135230560701e-17	0.228713491691364	0.036	0.022	2.51501397636795e-13	no change
5722	5722	EDNRB	1.1646043266853e-17	-1.303361132	0.018	0.03	2.54396169121137e-13	down
5723	5723	PCYOX1L	1.18029426446896e-17	0.211241852420095	0.029	0.016	2.578234791306e-13	no change
5724	5724	GATB	1.21488281070699e-17	0.136512359153318	0.074	0.053	2.65379001170836e-13	no change
5725	5725	S100A5	1.24125376450835e-17	-0.747362539	0.036	0.052	2.71139472319205e-13	no change
5726	5726	KCTD12	1.3108946440537e-17	-0.314283506	0.069	0.048	2.8635182604709e-13	no change
5727	5727	FANCM	1.32515811034797e-17	0.566192844126557	0.016	0.007	2.8946753762441e-13	no change
5728	5728	KIAA0753	1.33510081963084e-17	0.528300415595464	0.025	0.013	2.9163942304016e-13	no change
5729	5729	RP11-299J3.8	1.33675568603312e-17	0.391449016680912	0.026	0.014	2.92000912057075e-13	no change
5730	5730	TRIM66	1.34327104501358e-1	0.22730325029106	0.025	0.013	2.93424127072767e-1	no

			7				3	change
5731	5731	SLC9A2	1.3518288636781e-17	0.230633005145283	0.067	0.047	2.95293496981844e-13	no change
5732	5732	ITPKC	1.37701513351831e-17	-0.607275747	0.191	0.212	3.0079518576574e-13	no change
5733	5733	CTB-31O20.2	1.38351890878674e-17	0.194380048417488	0.05	0.033	3.02215870435376e-13	no change
5734	5734	LINC01068	1.4170843483119e-17	0.359530799136275	0.021	0.011	3.09547905045251e-13	no change
5735	5735	PRAC1	1.4541202546498e-17	-0.382605993	0.139	0.166	3.17638028425702e-13	no change
5736	5736	KIF27	1.45420453731687e-17	0.574396860571795	0.014	0.006	3.17656439131496e-13	no change
5737	5737	IGFBP6	1.47382671738252e-17	-1.88244722	0.159	0.177	3.21942708145038e-13	down
5738	5738	PVRL2	1.50062680240833e-17	-0.481361206	0.355	0.356	3.27796918718076e-13	no change
5739	5739	SELL	1.50106421431997e-17	0.325099178303506	0.043	0.028	3.27892466976054e-13	no change
5740	5740	P2RY6	1.50608856831983e-17	-1.347736568	0.005	0.012	3.28989986863784e-13	down
5741	5741	POR	1.5337615331556e-17	-0.111036743	0.256	0.205	3.3503486930251e-13	no change
5742	5742	BMP1	1.53568010049815e-17	-0.134916461	0.045	0.029	3.35453961152816e-13	no change
5743	5743	SCAMP2	1.65145071127168e-1	-0.103035091	0.207	0.165	3.60742893370186e-1	no

			7				3	change
5744	5744	ST5	1.68443978607354e-17	-0.203028755	0.12	0.09	3.67949026869904e-13	no change
5745	5745	MMP19	1.70147314835273e-17	-1.147438032	0.032	0.048	3.71669794526171e-13	down
5746	5746	ZNF483	1.70444552883975e-17	0.612087988381511	0.016	0.007	3.72319081319755e-13	no change
5747	5747	ANKRD18B	1.76998997900697e-17	0.341849217894892	0.022	0.011	3.86636611014282e-13	no change
5748	5748	SCN5A	1.77147800778134e-17	0.607201836624206	0.019	0.009	3.86961656019755e-13	no change
5749	5749	KIAA1324L	1.89435767989153e-17	0.128502995925966	0.057	0.038	4.13803491595506e-13	no change
5750	5750	ARL6IP5	1.89848767399503e-17	-0.380100143	0.484	0.462	4.14705647507475e-13	no change
5751	5751	TAS2R4	1.91315345357227e-17	0.776856643135649	0.012	0.005	4.17909240398326e-13	no change
5752	5752	TLR4	1.91594048173182e-17	0.250439701130999	0.034	0.02	4.18518038829499e-13	no change
5753	5753	ZNF688	1.91823260755235e-17	-0.132616565	0.138	0.106	4.19018730793735e-13	no change
5754	5754	ZSCAN31	1.92702674552837e-17	0.335187125412993	0.043	0.028	4.20939722293217e-13	no change
5755	5755	LINC01123	1.92748920611127e-17	0.290707807231601	0.034	0.021	4.21040742182946e-13	no change
5756	5756	CTA-445C9.14	1.93562741002418e-1	0.48407191306242	0.021	0.011	4.22818451445681e-1	no

			7	9			3 change
5757	5757	JADE3	1.96651138935395e-17	0.315021187423013	0.034	0.021	4.29564747890476e-13	no change
5758	5758	RP11-475O6.1	1.9845460330758e-17	0.701409871756882	0.013	0.005	4.33504235465078e-13	no change
5759	5759	MAN1C1	2.03763325590802e-17	-0.63741511	0.103	0.126	4.45100608420547e-13	no change
5760	5760	LINC00865	2.08265439922561e-17	0.49282165742439	0.034	0.02	4.54935026966842e-13	no change
5761	5761	PIGW	2.14899508594894e-17	0.156219286400862	0.051	0.034	4.69426486574686e-13	no change
5762	5762	TMEM147	2.14960900062284e-17	-0.279074853	0.763	0.674	4.69560590096054e-13	no change
5763	5763	CFAP70	2.17649165740932e-17	0.453179298971632	0.016	0.007	4.75432837644491e-13	no change
5764	5764	CCDC169	2.19014113980236e-17	0.266343826965683	0.046	0.03	4.78414430578427e-13	no change
5765	5765	ZBTB24	2.2450126128542e-17	0.120484702043774	0.043	0.027	4.90400555151873e-13	no change
5766	5766	TTLL5	2.27683129799141e-17	0.128660159621298	0.051	0.034	4.97351028733245e-13	no change
5767	5767	GRAMD1C	2.32798584949095e-17	0.192458457533233	0.049	0.032	5.08525228962802e-13	no change
5768	5768	POLR3GL	2.3918130665253e-17	-0.12136006	0.296	0.238	5.22467646251785e-13	no change
5769	5769	IFT88	2.39416083146923e-1	0.13104596382140	0.045	0.029	5.22980492026139e-1	no

			7	8			3	change
5770	5770	LTB	2.50552476729686e-17	0.584947811069124	0.033	0.02	5.47306830168327e-13	no change
5771	5771	ZDHHC19	2.60164195939706e-17	0.822890117643992	0.01	0.004	5.68302669610693e-13	no change
5772	5772	TRIM21	2.64996708537588e-17	0.187748175162687	0.036	0.022	5.78858810129506e-13	no change
5773	5773	ENPP5	2.66013818386061e-17	0.193652406674917	0.046	0.03	5.81080584882511e-13	no change
5774	5774	LY6K	2.76207324457992e-17	-0.23192382	0.297	0.233	6.03347279546037e-13	no change
5775	5775	TEKT5	2.81372201648316e-17	0.244637251945554	0.043	0.028	6.1462943728058e-13	no change
5776	5776	ANKRD13B	2.81558134260573e-17	0.303787399599161	0.017	0.008	6.15035588478796e-13	no change
5777	5777	DYNLRB1	2.83488421079897e-17	-0.203816981	0.738	0.666	6.19252107006928e-13	no change
5778	5778	NXF1	2.88257445350154e-17	-0.207888571	0.163	0.127	6.29669563622877e-13	no change
5779	5779	CTH	2.91109911339456e-17	0.290848365957468	0.024	0.013	6.35900490329908e-13	no change
5780	5780	PLK4	2.98320769452922e-17	0.486147045552297	0.015	0.007	6.51651888792962e-13	no change
5781	5781	ZNF300	3.00203540202812e-17	0.321830042861393	0.021	0.01	6.55764613219022e-13	no change
5782	5782	ZNF266	3.03239120416702e-1	0.12326275023545	0.048	0.032	6.62395534638244e-1	no

			7	2			3	change
5783	5783	MFSD14C	3.12792779486778e-17	0.353379311107868	0.023	0.012	6.83264547510918e-13	no change
5784	5784	C2orf15	3.17304644061804e-17	0.20872894645851	0.042	0.027	6.93120264488604e-13	no change
5785	5785	CCDC171	3.24633969520212e-17	0.201595831273153	0.022	0.011	7.09130443019951e-13	no change
5786	5786	HMGN4	3.34387627714437e-17	0.264603927599417	0.033	0.019	7.30436333979417e-13	no change
5787	5787	CLASRP	3.43744969195484e-17	-0.119490132	0.126	0.096	7.50876510710616e-13	no change
5788	5788	ZUFSP	3.5476121009725e-17	0.150111734150409	0.044	0.028	7.74940387336432e-13	no change
5789	5789	RNF32	3.58100765615003e-17	0.449702904411135	0.019	0.009	7.82235312409413e-13	no change
5790	5790	WDR89	3.6029871853685e-17	0.146866589670779	0.051	0.034	7.87036520771895e-13	no change
5791	5791	GDAP1	3.66726802378863e-17	0.162481780678572	0.035	0.021	8.01078027116389e-13	no change
5792	5792	MIR210HG	3.67355330613123e-17	-0.693170708	0.088	0.11	8.02450984191307e-13	no change
5793	5793	CRIP1	3.80482013756651e-17	-0.807918256	0.123	0.145	8.31124910850029e-13	no change
5794	5794	PAGR1	3.95101544180855e-17	0.377150188337073	0.024	0.013	8.63059813108659e-13	no change
5795	5795	YJEFN3	3.97222250822783e-1	0.19789598482937	0.025	0.014	8.67692284697287e-1	no

			7	1			3	change
5796	5796	TBC1D31	3.98977759735003e-17	0.4145695828922	0.019	0.009	8.7152701836514e-13	no change
5797	5797	AL589743.1	3.99800165128329e-17	0.344846798506185	0.037	0.023	8.73323480706322e-13	no change
5798	5798	PUS3	4.04334084660243e-17	0.141497195336439	0.047	0.031	8.83227374531835e-13	no change
5799	5799	PRKD2	4.08816514383817e-17	-0.139079215	0.138	0.106	8.9301879402001e-13	no change
5800	5800	PLIN2	4.18536429699854e-17	-0.667538703	0.378	0.37	9.14250977036361e-13	no change
5801	5801	PAPLN	4.20782938624605e-17	0.552129590826411	0.013	0.005	9.19158251131588e-13	no change
5802	5802	RP11-159D12.5	4.23040858777882e-17	0.689237294579711	0.014	0.006	9.24090451914407e-13	no change
5803	5803	CTD-2540F13.2	4.25298464609221e-17	0.171698626082241	0.03	0.017	9.29021966092383e-13	no change
5804	5804	FAM200A	4.3912988627081e-17	0.200747187683008	0.03	0.018	9.59235323569958e-13	no change
5805	5805	GID4	4.41826745737726e-17	0.21139775272895	0.046	0.03	9.65126343389489e-13	no change
5806	5806	EML6	4.50406761993653e-17	0.301989156618629	0.03	0.017	9.83868530898935e-13	no change
5807	5807	OLFML1	4.52467158892255e-17	-1.727692585	0.01	0.02	9.88369261884242e-13	down
5808	5808	ZNF649	4.55124441624573e-1	0.18585958325009	0.025	0.014	9.94173830284718e-1	no

			7	2			3	change
5809	5809	RP13-942N8.1	4.56718591388526e-17	0.238515472023602	0.029	0.017	9.97656091029096e-13	no change
5810	5810	ZNF714	4.56898766157657e-17	0.148950821280446	0.053	0.036	9.98049664794785e-13	no change
5811	5811	UPK2	4.68656394157149e-17	-1.349978234	0.296	0.308	1.02373302739688e-12	down
5812	5812	ZNF789	4.83106841632914e-17	0.281327676540984	0.032	0.019	1.05529858486294e-12	no change
5813	5813	DDX26B	4.87262694425606e-17	0.121883225189325	0.037	0.023	1.06437662970329e-12	no change
5814	5814	CENPT	4.98525713047443e-17	-0.103784997	0.114	0.086	1.08897956758083e-12	no change
5815	5815	FBXW8	5.02502103772327e-17	0.312565798102031	0.029	0.017	1.09766559548027e-12	no change
5816	5816	B3GNT7	5.05330528049859e-17	0.118923017975901	0.044	0.028	1.10384400547211e-12	no change
5817	5817	LARP1B	5.17897581490326e-17	-0.14301856	0.141	0.109	1.13129547700747e-12	no change
5818	5818	RP4-737E23.2	5.19263755353807e-17	-0.901662444	0.046	0.064	1.13427974719486e-12	no change
5819	5819	CDKL3	5.33062896227573e-17	0.101620051834843	0.045	0.029	1.16442259051951e-12	no change
5820	5820	JADE1	5.37586736023159e-17	-0.242592516	0.094	0.069	1.17430446616899e-12	no change
5821	5821	PIGR	5.47751552366006e-1	-1.240105358	0.066	0.086	1.1965084909883e-12	down

			7					
5822	5822	NIPAL2	5.62728265110955e-17	-0.125650255	0.16	0.126	1.22922362230837e-12	no change
5823	5823	RP11-631N16.2	5.77937283731079e-17	0.327864339944418	0.016	0.008	1.26244620258217e-12	no change
5824	5824	RP11-731C17.2	5.93221209105385e-17	0.665744158015576	0.011	0.004	1.2958324091698e-12	no change
5825	5825	TADA2B	5.97924330798195e-17	0.251030058561934	0.035	0.021	1.30610590819558e-12	no change
5826	5826	MORN1	6.16648847612685e-17	0.292273298729534	0.03	0.017	1.34700774272515e-12	no change
5827	5827	PRNCR1	6.20395408268155e-17	0.283340666395098	0.024	0.013	1.35519172982096e-12	no change
5828	5828	TIGD5	6.21818255654938e-17	0.493090586558977	0.017	0.008	1.35829979765265e-12	no change
5829	5829	RNF139	6.25391818584183e-17	-0.13175664	0.258	0.209	1.36610588851529e-12	no change
5830	5830	ERMAP	6.35209801495749e-17	0.266957796248962	0.037	0.023	1.38755229038732e-12	no change
5831	5831	RP11-582E3.6	6.40343357271871e-17	0.22491320370246	0.03	0.017	1.39876602962468e-12	no change
5832	5832	GTF2E1	6.60666196343177e-17	0.192938528906651	0.037	0.023	1.44315923929204e-12	no change
5833	5833	NFKBIZ	6.73217364317196e-17	-0.689779468	0.289	0.297	1.47057601061448e-12	no change
5834	5834	ADPRHL1	7.14045936110365e-1	0.32936291175656	0.025	0.014	1.55976194283948e-1	no

			7	9			2	change
5835	5835	DHRS1	7.42595632703284e-17	-0.101455035	0.163	0.128	1.62212590007705e-12	no change
5836	5836	B4GAT1	7.51836572344552e-17	0.126097816203085	0.029	0.017	1.64231180862944e-12	no change
5837	5837	SMTN	7.61752430580836e-17	-0.130699624	0.12	0.091	1.66397200936078e-12	no change
5838	5838	ZRANB3	7.75653313583198e-17	0.368295581103907	0.018	0.009	1.69433709819114e-12	no change
5839	5839	TNFAIP8	7.88290333479222e-17	-0.172294433	0.177	0.139	1.72194140445201e-12	no change
5840	5840	ZC3H6	8.12600192853347e-17	-0.139518128	0.155	0.121	1.77504386126885e-12	no change
5841	5841	FGF4	8.12813913565672e-17	-1.634674582	0.007	0.015	1.77551071279285e-12	down
5842	5842	SNAI3	8.28894249695502e-17	0.285926716605425	0.016	0.007	1.81063659903485e-12	no change
5843	5843	C12orf60	8.33223061450172e-17	0.501008190370571	0.018	0.008	1.82009245543176e-12	no change
5844	5844	E2F7	8.35199575154886e-17	0.482498999108706	0.045	0.03	1.82440995196833e-12	no change
5845	5845	POU5F2	8.40581757669888e-17	0.374353870550899	0.02	0.01	1.8361667914541e-12	no change
5846	5846	ESPL1	8.46469410083793e-17	1.0673883063977	0.01	0.003	1.84902777938704e-12	up
5847	5847	SGK3	8.87209275591999e-1	0.12120407351638	0.032	0.019	1.93801994160316e-1	no

			7	6			2	change
5848	5848	JRK	9.19729414556443e-17	0.118640493955064	0.053	0.036	2.00905693315709e-12	no change
5849	5849	MT1M	9.27818501655903e-17	-1.645762153	0.043	0.06	2.02672673501716e-12	down
5850	5850	ZBTB16	9.38027775466406e-17	-1.234963296	0.039	0.055	2.04902787272882e-12	down
5851	5851	MOCS3	9.65175498750314e-17	0.116372674010287	0.033	0.02	2.10832935947019e-12	no change
5852	5852	ZNF592	9.66489215896146e-17	0.108309196020145	0.054	0.037	2.11119904320354e-12	no change
5853	5853	RSPO3	9.78560259740524e-17	-1.557172867	0.011	0.021	2.1375670313772e-12	down
5854	5854	TSTA3	9.82208012885671e-17	-0.192411029	0.322	0.26	2.14553518334746e-12	no change
5855	5855	DPM1	9.99760731054311e-17	-0.107121086	0.469	0.377	2.18387734091504e-12	no change
5856	5856	ZNF257	1.01349349320251e-16	0.626373422409093	0.02	0.01	2.21387518655156e-12	no change
5857	5857	DDX19B	1.02260482433636e-16	-0.107561567	0.167	0.131	2.23377797828034e-12	no change
5858	5858	SECTM1	1.07356101163848e-16	-1.316090793	0.012	0.023	2.3450866738231e-12	down
5859	5859	CCNI2	1.07892877534382e-16	0.79756908527524	0.014	0.006	2.35681201686104e-12	no change
5860	5860	C10orf2	1.08935560333423e-1	0.37317342479675	0.02	0.01	2.37958837992329e-1	no

			6	9			2	change
5861	5861	RP11-379H18.1	1.09041522857403e-16	0.341315974906396	0.039	0.025	2.38190302529711e-12	no change
5862	5862	RP11-111F5.4	1.11845048334717e-16	0.506325869557302	0.024	0.013	2.44314323582355e-12	no change
5863	5863	PDIK1L	1.14470895604828e-16	0.205892616799727	0.039	0.024	2.50050224359186e-12	no change
5864	5864	ZNF689	1.16065324139943e-16	0.120358492709461	0.034	0.021	2.53533094051292e-12	no change
5865	5865	B3GAT3	1.18260637234723e-16	-0.12475727	0.184	0.145	2.58328535975529e-12	no change
5866	5866	C1orf233	1.18547194895903e-16	0.324223254740852	0.043	0.028	2.58954492530611e-12	no change
5867	5867	SPATA17	1.19989553837042e-16	-0.943102011	0.035	0.051	2.62105181401634e-12	no change
5868	5868	ZNF502	1.22986251896578e-16	0.413728985513555	0.02	0.01	2.68651168642886e-12	no change
5869	5869	SPATA5	1.29130566221291e-16	0.225039936656406	0.023	0.013	2.82072808853788e-12	no change
5870	5870	AIDA	1.29769724387947e-16	-0.156420072	0.209	0.168	2.83468985953032e-12	no change
5871	5871	FAM71E1	1.30888279532749e-16	0.247423800932914	0.039	0.025	2.85912357811336e-12	no change
5872	5872	ZBED3	1.32461092709284e-16	0.165994212267391	0.032	0.019	2.89348010914159e-12	no change
5873	5873	FOSL1	1.36123772086528e-1	-0.42633061	0.417	0.411	2.97348767745812e-1	no

			6				2 change
5874	5874	CDK17	1.37105842783596e-16	-0.108624429	0.072	0.052	2.99494002976487e-12	no change
5875	5875	CTD-2366F13.1	1.37670991076002e-16	0.395523115559436	0.02	0.01	3.0072851290642e-12	no change
5876	5876	CENPP	1.37855248912254e-16	0.17306108386362	0.033	0.02	3.01131005723928e-12	no change
5877	5877	LINC01569	1.43507225567559e-16	0.381122412610786	0.019	0.01	3.13477183529776e-12	no change
5878	5878	SHANK2	1.505748647295e-16	0.342976983324018	0.03	0.018	3.28915734515121e-12	no change
5879	5879	MICALCL	1.53483508116345e-16	0.306305835987127	0.025	0.014	3.35269375129344e-12	no change
5880	5880	GDF7	1.55520769725041e-16	0.310287861622141	0.026	0.014	3.39719569387379e-12	no change
5881	5881	POLG2	1.61462726408127e-16	-0.145155031	0.071	0.05	3.52699179565912e-12	no change
5882	5882	FUT10	1.61634770328037e-16	0.32125775679504	0.023	0.012	3.53074992304565e-12	no change
5883	5883	RIBC2	1.66141890105339e-16	0.837386400416176	0.01	0.003	3.62920344746102e-12	no change
5884	5884	PDE1B	1.70871676866776e-16	0.993597976903927	0.011	0.004	3.73252090947786e-12	no change
5885	5885	SPTY2D1	1.73448503739778e-16	-0.189277843	0.164	0.129	3.78880911569171e-12	no change
5886	5886	TAC1	1.95253084983198e-1	0.11118977920375	0.018	0.008	4.26510838837299e-1	no

			6	3			2	change
5887	5887	TRAIP	1.95472542318529e-16	0.751190658855179	0.011	0.004	4.26990221440594e-12	no change
5888	5888	HSPA6	1.98841414519467e-16	-0.800905526	0.165	0.19	4.34349185876324e-12	no change
5889	5889	ZNF606	2.07305883753693e-16	0.347252102585802	0.028	0.016	4.52838972471567e-12	no change
5890	5890	TH	2.08007562719777e-16	0.337023029136113	0.043	0.028	4.5437172000508e-12	no change
5891	5891	RP4-635E18.6	2.09998372577284e-16	0.410150784674957	0.03	0.018	4.5872044505782e-12	no change
5892	5892	CTD-2369P2.10	2.12126375475378e-16	0.424545012539608	0.019	0.009	4.63368854588415e-12	no change
5893	5893	AGMO	2.15857394427267e-16	0.115901385785239	0.022	0.012	4.71518892386922e-12	no change
5894	5894	FCMR	2.27759541167236e-16	0.393448451005256	0.021	0.011	4.97517941725711e-12	no change
5895	5895	RP11-727F15.11	2.2826710369285e-16	0.743933556758669	0.012	0.005	4.98626661306662e-12	no change
5896	5896	TCF7	2.29251500356468e-16	-0.240366357	0.121	0.092	5.00776977378668e-12	no change
5897	5897	DHCR24	2.3022213012424e-16	-0.655703692	0.117	0.139	5.0289722104339e-12	no change
5898	5898	GRIP1	2.30975578308783e-16	0.326192855924861	0.025	0.014	5.04543053257705e-12	no change
5899	5899	IL18R1	2.34524342939972e-1	0.57293592752923	0.012	0.004	5.12294974718075e-1	no

			6	4			2	change
5900	5900	TMEM106B	2.4176494486376e-16	-0.159820098	0.299	0.242	5.28111345560398e-12	no change
5901	5901	MFSD6L	2.47089106790984e-16	0.97000693219938	0.01	0.003	5.39741444874224e-12	no change
5902	5902	STX4	2.47744944215209e-16	-0.140215586	0.263	0.212	5.41174056143702e-12	no change
5903	5903	P2RY11	2.52649472185853e-16	0.441306742265284	0.019	0.009	5.51887507042777e-12	no change
5904	5904	CENPBD1	2.55398858186373e-16	0.313381483785041	0.027	0.015	5.57893265822313e-12	no change
5905	5905	LINC00840	2.6555513911961e-16	-0.725209019	0.022	0.035	5.80078645892876e-12	no change
5906	5906	PCED1B	2.66050189879231e-16	0.151067575933822	0.06	0.042	5.81160034772191e-12	no change
5907	5907	RP11-572O17.1	2.69160332465328e-16	0.331650266440605	0.022	0.011	5.87953830237261e-12	no change
5908	5908	GINM1	2.78888904129881e-16	-0.212716406	0.291	0.233	6.09204922181313e-12	no change
5909	5909	ST6GAL2	2.87363992629081e-16	0.686937611084761	0.011	0.004	6.27717905498964e-12	no change
5910	5910	RPS6KA6	2.92514413051047e-16	0.136109515556732	0.028	0.016	6.38968483868707e-12	no change
5911	5911	RELA	2.95181303781076e-16	-0.11152752	0.209	0.168	6.44794039979383e-12	no change
5912	5912	BORA	3.08701982901156e-1	0.33793911368424	0.024	0.013	6.74328611449285e-1	no

			6	8			2	change
5913	5913	CORO1C	3.17305536616609e-16	-0.149922254	0.16	0.126	6.93122214185322e-12	no change
5914	5914	RP11-206L10.9	3.17583239033202e-16	0.185117683107368	0.03	0.018	6.93728827344126e-12	no change
5915	5915	FGF7	3.24889751765906e-16	-1.596460567	0.05	0.066	7.09689173757445e-12	down
5916	5916	MGLL	3.30656201556562e-16	-0.639433442	0.119	0.142	7.22285406680154e-12	no change
5917	5917	CCL28	3.35457776799283e-16	0.400957410538506	0.021	0.011	7.32773967640353e-12	no change
5918	5918	CCDC144A	3.38684669261829e-16	0.966176833547141	0.011	0.004	7.3982279153554e-12	no change
5919	5919	CTD-2566J3.1	3.39405896827033e-16	0.709901593480776	0.012	0.005	7.4139824102897e-12	no change
5920	5920	ADAM22	3.41502657655509e-16	0.664256234355741	0.012	0.005	7.45978405382694e-12	no change
5921	5921	IGSF11	3.41878525366824e-16	0.451046177841352	0.015	0.007	7.46799450811291e-12	no change
5922	5922	HIRA	3.42503095903892e-16	0.190752890049017	0.032	0.019	7.48163762692462e-12	no change
5923	5923	SP4	3.45602132761755e-16	-0.110596107	0.043	0.028	7.54933298804778e-12	no change
5924	5924	ZBTB37	3.46063120900915e-16	0.101226353439129	0.038	0.024	7.55940281295958e-12	no change
5925	5925	PRPS1	3.46778184159153e-1	-0.101078256	0.122	0.094	7.57502265477253e-1	no

			6				2	change
5926	5926	LINC01311	3.488072178241e-16	0.444865551928944	0.02	0.01	7.61934486614963e-12	no change
5927	5927	RP11-457M11.5	3.53280685620292e-16	0.435784011848107	0.02	0.01	7.71706329668965e-12	no change
5928	5928	TIGD7	3.55061612291731e-16	0.703767173201676	0.014	0.006	7.75596585890057e-12	no change
5929	5929	FAN1	3.59414421184644e-16	0.170046567661112	0.034	0.021	7.85104861635736e-12	no change
5930	5930	MTRNR2L6	3.67178011785733e-16	0.672745669080242	0.011	0.004	8.02063648944755e-12	no change
5931	5931	SIGIRR	3.73135642438566e-16	-0.125458617	0.223	0.178	8.15077497342804e-12	no change
5932	5932	GALK2	3.83719578171367e-16	-0.104035201	0.121	0.093	8.38197046557533e-12	no change
5933	5933	ENTPD1-AS1	3.84011747537893e-16	0.264386769137841	0.03	0.018	8.38835261321774e-12	no change
5934	5934	ZSCAN12	3.90758270700892e-16	0.690332590429168	0.016	0.007	8.53572366519028e-12	no change
5935	5935	ALDH1L1-AS2	4.02860129567412e-16	0.594564550725843	0.018	0.009	8.80007667027055e-12	no change
5936	5936	ALG10B	4.09833160271156e-16	0.188044760783495	0.029	0.017	8.95239555296313e-12	no change
5937	5937	PCDHGA10	4.32602766502675e-16	0.144259153733233	0.02	0.011	9.44977483148443e-12	no change
5938	5938	SPA17	4.36400415278124e-1	0.10847938001381	0.044	0.029	9.53273067133535e-1	no

			6	1			2	change
5939	5939	GATM	4.50980877848015e-16	-0.159852966	0.159	0.124	9.85122629571204e-12	no change
5940	5940	CHST12	4.67333800778016e-16	-0.208212261	0.164	0.128	1.0208439544195e-11	no change
5941	5941	CH507-9B2.5	4.69146270452546e-16	0.556226827775441	0.016	0.008	1.02480311317654e-11	no change
5942	5942	SLC45A4	4.75594506112585e-16	0.288778154722168	0.026	0.015	1.03888863915233e-11	no change
5943	5943	PPP2R3C	4.81899908851058e-16	-0.110337407	0.157	0.124	1.05266216089425e-11	no change
5944	5944	UGT1A7	4.94792266123937e-16	-0.789236443	0.08	0.101	1.08082422612113e-11	no change
5945	5945	RP11-879F14.2	4.96852185769767e-16	0.179546177398236	0.037	0.023	1.08532391459548e-11	no change
5946	5946	EHHADH	5.04665791241252e-16	0.199654508600463	0.029	0.017	1.10239195438739e-11	no change
5947	5947	KCNJ14	5.18763661685779e-16	0.536446360059843	0.016	0.007	1.13318734258642e-11	no change
5948	5948	RBM43	5.47350041379405e-16	0.107804746970266	0.041	0.026	1.19563143038917e-11	no change
5949	5949	ARL17B	5.57727208011735e-16	0.186791777367126	0.02	0.011	1.21829931318083e-11	no change
5950	5950	ZNF623	5.61968274424714e-16	0.154927252875366	0.03	0.018	1.22756349865335e-11	no change
5951	5951	MYADM	5.73257282065918e-1	-0.970385984	0.079	0.098	1.25222320694479e-1	no

			6				1	change
5952	5952	TMEM138	6.07242516526657e-16	-0.124057475	0.22	0.177	1.32646055310083e-11	no change
5953	5953	ARHGAP22	6.2196302613796e-16	0.324805396745779	0.019	0.01	1.35861603429576e-11	no change
5954	5954	SYNE4	6.41467874970763e-16	-0.551932787	0.125	0.148	1.40122242608613e-11	no change
5955	5955	ATP2A1-AS1	6.44851579845039e-16	0.431536610610972	0.018	0.009	1.4086137910135e-11	no change
5956	5956	ATG2B	6.51931198486298e-16	0.160084067581367	0.047	0.032	1.42407850997347e-11	no change
5957	5957	LIMCH1	6.53895473495799e-16	-0.113431475	0.33	0.269	1.42836927230422e-11	no change
5958	5958	RP3-406A7.7	6.70426064441111e-16	0.236487991999746	0.019	0.01	1.46447869516516e-11	no change
5959	5959	BACE1-AS	6.8612086039636e-16	0.489285584575976	0.017	0.008	1.49876240744981e-11	no change
5960	5960	GFPT2	6.90354880270212e-16	-1.132058719	0.02	0.032	1.50801120046225e-11	down
5961	5961	ZNF662	6.9041055691242e-16	-0.780582931	0.031	0.046	1.50813282051949e-11	no change
5962	5962	SHPK	7.01502300142377e-16	0.242659132679318	0.027	0.015	1.53236162443101e-11	no change
5963	5963	DCAF4	7.1423618875589e-16	0.335308571027988	0.035	0.022	1.56017753071837e-11	no change
5964	5964	NCCRP1	7.18381839634619e-1	-0.698361045	0.092	0.114	1.56923329049786e-1	no

			6				1	change
5965	5965	ARL11	7.35921471191669e-16	0.3893230852677	0.024	0.013	1.60754686167108e-11	no change
5966	5966	AC007228.11	7.53874529668873e-16	0.745037383762322	0.011	0.004	1.64676352260869e-11	no change
5967	5967	SLC25A26	7.57516352260627e-16	-0.104448666	0.186	0.149	1.65471871987811e-11	no change
5968	5968	RP3-461P17.10	7.62390810403358e-16	-1.320170261	0.006	0.013	1.6653664862451e-11	down
5969	5969	AC009264.1	7.93039716451193e-16	0.342627351742617	0.014	0.006	1.73231595661599e-11	no change
5970	5970	MAPKBP1	8.0188383597242e-16	0.194135984334157	0.03	0.018	1.75163505129815e-11	no change
5971	5971	DLGAP4	8.06285545043716e-16	-0.152455618	0.178	0.142	1.76125014459349e-11	no change
5972	5972	CTD-2287O16.5	8.16718387449664e-16	0.34139708934441	0.024	0.014	1.78403964554505e-11	no change
5973	5973	NMT2	8.18251379229149e-16	-0.167110801	0.049	0.033	1.78738831278815e-11	no change
5974	5974	ANKRD16	8.4641660761095e-16	0.260027031807798	0.037	0.024	1.84891243766536e-11	no change
5975	5975	PHKA1	8.49173352339157e-16	0.182876333992114	0.029	0.017	1.85493427084965e-11	no change
5976	5976	MCM9	8.53246985454454e-16	0.285600647569561	0.027	0.016	1.86383271502671e-11	no change
5977	5977	HEATR3	8.55157330594673e-1	0.10586175341990	0.024	0.014	1.868005672951e-11	no

			6	2				change
5978	5978	C10orf82	8.76791325561938e-16	0.449971548820375	0.023	0.013	1.9152629715575e-11	no change
5979	5979	UBE2H	8.91926847271407e-16	-0.131327875	0.332	0.271	1.94832500517966e-11	no change
5980	5980	LINC01001	9.00695765372723e-16	0.266572542593329	0.019	0.01	1.96747982988018e-11	no change
5981	5981	RAB12	9.19403286071021e-16	-0.169869039	0.065	0.046	2.00834453809354e-11	no change
5982	5982	DOK1	9.54618957095554e-16	0.13548058025019	0.044	0.029	2.08526964987953e-11	no change
5983	5983	FAM229A	9.5929849308358e-16	0.312923854379362	0.022	0.012	2.09549162829177e-11	no change
5984	5984	SFXN3	9.59448092085045e-16	-0.712781891	0.084	0.105	2.09581841235057e-11	no change
5985	5985	NDUFS6	9.72828900277642e-16	0.164683376243213	0.831	0.738	2.12504744976648e-11	no change
5986	5986	TMC5	9.77822378057692e-16	-1.356409436	0.02	0.033	2.13595520262922e-11	down
5987	5987	ZBTB14	9.95629322986778e-16	0.280758165163142	0.034	0.021	2.17485269313232e-11	no change
5988	5988	PSORS1C1	1.01708558545975e-15	-0.956676502	0.024	0.037	2.22172175287829e-11	no change
5989	5989	SCOC-AS1	1.03659914863121e-15	0.132944226256024	0.05	0.034	2.26434718027003e-11	no change
5990	5990	ZCCHC4	1.04011011120187e-1	0.15376454822733	0.033	0.02	2.27201652690936e-1	no

			5	8			1	change
5991	5991	RP11-107N15.1	1.10114816568686e-15	0.892022769982658	0.011	0.004	2.40534805312637e-11	no change
5992	5992	TEX2	1.10770675736041e-15	0.196910284660251	0.036	0.023	2.41967464077808e-11	no change
5993	5993	WDR5B	1.15254637757838e-15	0.234935777092647	0.031	0.019	2.51762230718221e-11	no change
5994	5994	PLPP6	1.16999598831518e-15	0.221661121495716	0.03	0.018	2.55573923687567e-11	no change
5995	5995	DTWD2	1.17907192813119e-15	0.238467449405261	0.036	0.023	2.57556471980978e-11	no change
5996	5996	RTN4IP1	1.18235474609249e-15	0.235442299170173	0.035	0.022	2.58273570736445e-11	no change
5997	5997	UBASH3B	1.18440263019957e-15	0.18845257381226	0.045	0.03	2.58720910540794e-11	no change
5998	5998	KIAA1549L	1.19230850196809e-15	0.756190235757982	0.01	0.003	2.6044786916991e-11	no change
5999	5999	IFT20	1.22953243270012e-15	-0.397433664	0.472	0.44	2.68579064599015e-11	no change
6000	6000	P4HTM	1.24890576626188e-15	-0.103694458	0.218	0.176	2.72810975582246e-11	no change
6001	6001	ITGB5	1.24959998558314e-15	-0.226867928	0.062	0.043	2.7296262085078e-11	no change
6002	6002	PCDHB2	1.26158807024568e-15	0.568681236052154	0.011	0.004	2.75581298064467e-11	no change
6003	6003	HOTAIRM1	1.29866851107783e-1	-0.169433842	0.455	0.369	2.8368114955984e-11	no

			5					change
6004	6004	LRG1	1.33393952426193e-15	-0.160434684	0.139	0.108	2.91385749679776e-11	no change
6005	6005	RP3-399L15.3	1.34274771460595e-15	0.629021931420457	0.017	0.008	2.93309810778525e-11	no change
6006	6006	ARHGAP15	1.34277439692519e-15	0.22410302264357	0.021	0.011	2.93315639264339e-11	no change
6007	6007	MAP7D3	1.34406730526471e-15	-0.362166232	0.059	0.041	2.93598062162023e-11	no change
6008	6008	B3GALT6	1.34628949619292e-15	0.117764733983329	0.081	0.06	2.94083477548382e-11	no change
6009	6009	AC000032.2	1.3758486193275e-15	-1.030933467	0.02	0.033	3.00540372405898e-11	down
6010	6010	ODAM	1.39399127195979e-15	-0.953653164	0.015	0.026	3.04503453446896e-11	no change
6011	6011	MFSD9	1.48316943488167e-15	0.129302134677852	0.065	0.047	3.23983531355553e-11	no change
6012	6012	HACD4	1.50046235311628e-15	-0.13807628	0.05	0.034	3.2776099641472e-11	no change
6013	6013	HOXD4	1.52214162876727e-15	0.133089446603376	0.032	0.02	3.32496617387922e-11	no change
6014	6014	RP11-305K5.1	1.55807910159846e-15	0.498432339408971	0.012	0.005	3.40346798953168e-11	no change
6015	6015	RP11-378A13.2	1.56387056898305e-15	-0.974548414	0.014	0.024	3.41611887088658e-11	no change
6016	6016	BET1L	1.63695681286568e-1	-0.14705867	0.114	0.087	3.57576846202379e-1	no

			5				1	change
6017	6017	UPK1B	1.63870455119248e-15	-0.524678654	0.358	0.375	3.57958622162484e-11	no change
6018	6018	ACSL1	1.66218147091014e-15	-0.659451994	0.148	0.169	3.63086920505611e-11	no change
6019	6019	CLN8	1.67059557767675e-15	-0.191967602	0.199	0.158	3.64924897987709e-11	no change
6020	6020	OS9	1.73681555037724e-15	-0.145718937	0.499	0.41	3.79389988824404e-11	no change
6021	6021	TMEM171	1.78229057255215e-15	0.464160880391375	0.017	0.009	3.89323552668291e-11	no change
6022	6022	ERLEC1	1.80555097229735e-15	-0.142202739	0.408	0.33	3.94404554388633e-11	no change
6023	6023	RP11-175O19.4	1.83307856686933e-15	0.383922586871577	0.03	0.018	4.00417682146937e-11	no change
6024	6024	BDKRB1	1.87047381899547e-15	-1.280411649	0.021	0.033	4.08586301021371e-11	down
6025	6025	RP11-212I21.4	1.88273356631e-15	0.344473284629897	0.016	0.008	4.11264320224756e-11	no change
6026	6026	AC005537.2	1.94936208233042e-15	0.14937705562487	0.057	0.04	4.25818653264257e-11	no change
6027	6027	DNAJC1	1.99301873723177e-15	-0.166986727	0.305	0.248	4.35355012960907e-11	no change
6028	6028	VIMP	2.09926585089168e-15	-0.280724609	0.69	0.625	4.58563632468778e-11	no change
6029	6029	KIF26B	2.10394179631774e-1	0.63634536627174	0.014	0.006	4.59585045987648e-1	no

			5	7			1	change
6030	6030	FAP	2.15656920874084e-15	-1.955030882	0.006	0.014	4.71080977957348e-11	down
6031	6031	FLYWCH1	2.19986861582306e-15	-0.128616497	0.072	0.052	4.80539300440388e-11	no change
6032	6032	CHD2	2.21346979633672e-15	-0.119840957	0.289	0.235	4.83510342311792e-11	no change
6033	6033	TDRD7	2.21978970527037e-15	0.341791948179837	0.017	0.008	4.84890863219259e-11	no change
6034	6034	DFFB	2.26065518077057e-15	0.530132871107488	0.018	0.009	4.93817517687523e-11	no change
6035	6035	GOLT1B	2.28621009454875e-15	-0.120969491	0.202	0.163	4.99399733053229e-11	no change
6036	6036	ZBTB45	2.32003128959586e-15	0.172503485784251	0.041	0.027	5.06787634899319e-11	no change
6037	6037	KIZ-AS1	2.35883864658515e-15	0.714347389808223	0.012	0.005	5.15264713960061e-11	no change
6038	6038	STAM-AS1	2.39560577304937e-15	0.279872552021033	0.037	0.024	5.23296125064904e-11	no change
6039	6039	POLR3B	2.51031770057339e-15	0.26413381146395	0.03	0.018	5.48353798513252e-11	no change
6040	6040	FGFR1OP2	2.5319034130568e-15	-0.163064463	0.269	0.218	5.53068981548128e-11	no change
6041	6041	TTC30A	2.71539380073688e-15	0.38595608648581	0.017	0.008	5.93150621832963e-11	no change
6042	6042	B3GNTL1	2.7238507891086e-15	0.13347049804347	0.042	0.028	5.94997966372883e-1	no

				7			1	change
6043	6043	IL13RA1	2.74113052077272e-15	-0.126329501	0.136	0.107	5.98772550957594e-11	no change
6044	6044	SPIN3	2.76779182512643e-15	0.32692425277832	0.024	0.014	6.04596446280617e-11	no change
6045	6045	MPV17L	2.78810795395339e-15	0.297008056039576	0.026	0.015	6.09034301461578e-11	no change
6046	6046	ZNF383	2.82974177629922e-15	0.330489953382468	0.025	0.014	6.18128793614801e-11	no change
6047	6047	INTS5	2.83667513368491e-15	0.105117129275361	0.045	0.03	6.19643316202132e-11	no change
6048	6048	SLC35F2	2.94972518452983e-15	0.186707500662616	0.04	0.026	6.44337969308697e-11	no change
6049	6049	GARS	2.95599324974145e-15	-0.127365973	0.192	0.155	6.45707165473521e-11	no change
6050	6050	SPOCD1	3.00213499854599e-15	-0.297051202	0.395	0.322	6.55786369082387e-11	no change
6051	6051	C6orf132	3.04941053630004e-15	-0.103063736	0.233	0.19	6.66113237549381e-11	no change
6052	6052	DMTN	3.0669809814464e-15	0.181779969076011	0.03	0.018	6.69951325587152e-11	no change
6053	6053	TMEM9B	3.09481430817201e-15	-0.104665278	0.464	0.377	6.76031237477093e-11	no change
6054	6054	PRKCB	3.11136894940828e-15	0.264275269552943	0.037	0.024	6.79647433308744e-11	no change
6055	6055	RP11-181C3.1	3.14901181601613e-1	0.31099544019932	0.018	0.009	6.87870141090563e-1	no

			5	6			1	change
6056	6056	TMEM132A	3.19001117414091e-15	0.212034837419159	0.039	0.025	6.96826040879341e-11	no change
6057	6057	GYG2	3.19205750920663e-15	0.442987492244837	0.02	0.01	6.97273042311097e-11	no change
6058	6058	MAP1LC3A	3.28107940414661e-15	-0.173355714	0.312	0.253	7.16718985041785e-11	no change
6059	6059	ENKD1	3.35697056709325e-15	0.116076650382212	0.046	0.03	7.3329665067585e-11	no change
6060	6060	RIPPLY3	3.36338038089951e-15	0.470861355757773	0.016	0.008	7.34696810403688e-11	no change
6061	6061	CTD-2626G11.2	3.40285461414715e-15	-0.635550347	0.103	0.124	7.43319561914304e-11	no change
6062	6062	RP11-93H24.3	3.55637907363703e-15	0.218438034623246	0.034	0.021	7.76855444845272e-11	no change
6063	6063	TMPO-AS1	3.5602645202409e-15	0.568232406663325	0.016	0.007	7.77704181801421e-11	no change
6064	6064	ABHD15	3.57924901695801e-15	0.194975561273655	0.041	0.027	7.81851155264308e-11	no change
6065	6065	LYRM1	3.63993617527351e-15	-0.134394406	0.218	0.176	7.95107658126745e-11	no change
6066	6066	IRAK1BP1	3.68289452976979e-15	0.203924603291797	0.03	0.018	8.04491481082914e-11	no change
6067	6067	ZMYM5	3.74849874281245e-15	-0.123680185	0.11	0.084	8.18822065379952e-11	no change
6068	6068	FAM89B	3.94582042573796e-1	-0.105258008	0.253	0.206	8.61925013798199e-1	no

			5				1	change
6069	6069	CROCC	3.97745613804008e-15	0.104254497000975	0.048	0.033	8.68835518793476e-11	no change
6070	6070	CRIPAK	4.13596567318693e-15	0.268778216293302	0.023	0.012	9.03460341650954e-11	no change
6071	6071	SCRN2	4.31738075792076e-15	-0.124949191	0.222	0.179	9.43088652760211e-11	no change
6072	6072	AP4E1	4.32663531078751e-15	0.155912850829694	0.033	0.02	9.45110217288423e-11	no change
6073	6073	RMND5B	4.36182086119283e-15	-0.110075193	0.143	0.113	9.52796148918961e-11	no change
6074	6074	GTF2A1	4.3622863814658e-15	-0.108883103	0.104	0.079	9.52897837167389e-11	no change
6075	6075	RP11-796E10.1	4.36236887995406e-15	0.220415136927213	0.042	0.028	9.52915858137165e-11	no change
6076	6076	ZNF681	4.37097553512465e-15	0.266641181753107	0.038	0.025	9.54795895892629e-11	no change
6077	6077	ZNF790-AS1	4.40176890536735e-15	0.598997740693103	0.012	0.005	9.61522399688443e-11	no change
6078	6078	RP11-88I18.2	4.40717551011098e-15	0.449190243653678	0.012	0.005	9.62703418428642e-11	no change
6079	6079	UNC119B	4.45326950862169e-15	0.250191428016146	0.043	0.029	9.72772191463323e-11	no change
6080	6080	RING1	4.45772692676804e-15	-0.208230918	0.191	0.152	9.7374586988321e-11	no change
6081	6081	LINC00857	4.49885898431121e-1	-1.015735584	0.009	0.018	9.82730756532941e-1	down

			5				1	
6082	6082	CRISPLD1	4.50555355453834e-15	0.744431449679402	0.011	0.005	9.84193118453356e-11	no change
6083	6083	C15orf57	4.57507372487368e-15	-0.125553077	0.146	0.115	9.99379104461407e-11	no change
6084	6084	TNFAIP3	4.70868673216516e-15	-0.76765324	0.253	0.267	1.02856552977416e-10	no change
6085	6085	MPV17	4.72019973171018e-15	-0.159228397	0.281	0.227	1.03108042939477e-10	no change
6086	6086	MNX1-AS1	4.78404559398589e-15	0.280613744095104	0.016	0.008	1.04502691955028e-10	no change
6087	6087	LINC00619	4.83377391659307e-15	-0.591067862	0.041	0.058	1.05588957434059e-10	no change
6088	6088	SLC35G1	4.83435405722711e-15	0.273837501875306	0.037	0.024	1.05601630026069e-10	no change
6089	6089	VPS41	5.0773710905359e-15	-0.106740863	0.157	0.124	1.10910094101666e-10	no change
6090	6090	PDLIM4	5.1714544528397e-15	-0.284938825	0.192	0.153	1.1296525106783e-10	no change
6091	6091	DPYSL2	5.57761558915935e-15	-1.037440485	0.063	0.081	1.21837434929597e-10	down
6092	6092	CRACR2A	5.62974574965087e-15	0.417101309479649	0.026	0.015	1.22976166155374e-10	no change
6093	6093	LAMP3	5.67179128016127e-15	0.290496013713677	0.027	0.016	1.23894608723843e-10	no change
6094	6094	RASSF3	5.69612636477622e-1	-0.11227732	0.103	0.079	1.24426184312172e-1	no

			5				0	change
6095	6095	PLAT	5.74459143698441e-15	-0.431206364	0.165	0.129	1.25484855349487e-10	no change
6096	6096	RP11-345J4.5	5.79169234404068e-15	0.131317747017585	0.051	0.036	1.26513727563225e-10	no change
6097	6097	RP11-742B18.1	5.83008544647023e-15	0.257334059422854	0.027	0.016	1.27352386492696e-10	no change
6098	6098	CDC42EP2	5.9117778845123e-15	-0.768826205	0.102	0.122	1.29136876109287e-10	no change
6099	6099	KLHL31	5.96097431835141e-15	0.645522474730035	0.014	0.007	1.30211523010068e-10	no change
6100	6100	SDR16C5	5.96767736297925e-15	0.334341885254736	0.016	0.008	1.30357944316919e-10	no change
6101	6101	TMEM167B	6.05712070712776e-15	-0.136533457	0.205	0.166	1.32311744726499e-10	no change
6102	6102	CARD16	6.65636106828931e-15	-0.132061243	0.192	0.154	1.45401551175712e-10	no change
6103	6103	ZNRD1-AS1	7.01432981364968e-15	0.218762264813728	0.023	0.013	1.53221020449363e-10	no change
6104	6104	FDPS	7.0518072296414e-15	-0.694641144	0.547	0.491	1.54039677124287e-10	no change
6105	6105	ADHFE1	7.08158712175284e-15	0.273765772544118	0.018	0.009	1.54690189087569e-10	no change
6106	6106	RHBG	7.21797907880673e-15	0.163352432132298	0.055	0.039	1.57669534997454e-10	no change
6107	6107	SMOC2	7.34027273954973e-1	-1.215783821	0.024	0.036	1.60340917722724e-1	down

			5				0	
6108	6108	PDGFD	7.38131161974329e-15	-2.322528459	0.004	0.01	1.61237371021672e-10	down
6109	6109	TMPRSS11E	7.56921052682231e-15	0.220150956937563	0.051	0.035	1.65341834747906e-10	no change
6110	6110	POU5F1B	7.5858498411409e-15	0.638652787921436	0.014	0.006	1.65705303929882e-10	no change
6111	6111	NPIPA1	7.77338534839475e-15	0.175891841635291	0.024	0.014	1.69801829550335e-10	no change
6112	6112	LIPH	7.84920331849852e-15	-0.439490917	0.36	0.368	1.71457997289282e-10	no change
6113	6113	METTL21B	7.9982562420189e-15	-0.228715474	0.091	0.068	1.74713909350661e-10	no change
6114	6114	TMEM159	8.13321199727291e-15	-0.190348164	0.404	0.333	1.77661882868429e-10	no change
6115	6115	RP11-473M20.16	8.20941852741261e-15	0.268663667860205	0.035	0.022	1.79326538312801e-10	no change
6116	6116	LINC00467	8.39111239706848e-15	-0.170652894	0.247	0.201	1.83295459201564e-10	no change
6117	6117	RP1-78B3.1	8.52876679726671e-15	0.387372687491436	0.018	0.009	1.86302381919494e-10	no change
6118	6118	HDAC11	8.92066092644525e-15	0.220909043569663	0.049	0.034	1.9486291727727e-10	no change
6119	6119	RP11-61L19.2	8.97407075690204e-15	-1.218447153	0.011	0.02	1.96029601613768e-10	down
6120	6120	MTBP	9.00959372058635e-1	0.69820572230799	0.012	0.005	1.96805565232488e-1	no

			5	4			0	change
6121	6121	RAPGEF4	9.06976250990215e-15	0.466889540972369	0.017	0.009	1.98119892266303e-10	no change
6122	6122	ERVMER34-1	9.1771522537872e-15	0.297096884219469	0.02	0.011	2.00465713831728e-10	no change
6123	6123	UBE2D1	9.1978310862472e-15	-0.110792312	0.389	0.318	2.00917422247984e-10	no change
6124	6124	RDX	9.3062012595905e-15	-0.131384855	0.489	0.404	2.03284660314495e-10	no change
6125	6125	EIF1AY	9.39900941421161e-15	-0.329086444	0.303	0.32	2.05311961644038e-10	no change
6126	6126	PILRA	9.4272417273315e-15	-1.774826154	0.009	0.018	2.05928668291829e-10	down
6127	6127	IL27RA	9.53829731451032e-15	0.314916839704204	0.018	0.009	2.08354566538163e-10	no change
6128	6128	GOLGA7	9.65882844505418e-15	-0.152100576	0.302	0.246	2.10987448553763e-10	no change
6129	6129	HIST2H2BF	9.68212438243237e-15	-1.070394557	0.015	0.025	2.11496325009853e-10	down
6130	6130	ACHE	9.74466260393617e-15	-1.326976194	0.016	0.026	2.12862409920382e-10	down
6131	6131	STX7	9.77937959216236e-15	-0.127871392	0.221	0.18	2.13620767811195e-10	no change
6132	6132	NSUN7	9.80815635451056e-15	0.139811622092432	0.04	0.026	2.14249367407929e-10	no change
6133	6133	HPS6	1.01285123211822e-1	0.17957539484108	0.03	0.018	2.21247223143904e-1	no

			4				0	change
6134	6134	RAMP2	1.02112846688011e-14	-0.598380982	0.044	0.029	2.23055302305291e-10	no change
6135	6135	STARD3NL	1.03680060203838e-14	-0.12861429	0.27	0.221	2.26478723509264e-10	no change
6136	6136	SLC22A17	1.04267969365346e-14	-0.102877394	0.037	0.024	2.27762952281662e-10	no change
6137	6137	KLHL3	1.06416342337763e-14	0.149317894950688	0.028	0.017	2.32455858202609e-10	no change
6138	6138	VLDLR	1.06570714595186e-14	0.320048276203475	0.027	0.016	2.32793068961724e-10	no change
6139	6139	HBEGF	1.108858034685e-14	-0.376763484	0.297	0.314	2.4221894909659e-10	no change
6140	6140	TUBE1	1.13769819148831e-14	-0.129135002	0.105	0.081	2.48518792948707e-10	no change
6141	6141	CHRDL2	1.14255930976275e-14	-1.47923904	0.006	0.013	2.49580655624575e-10	down
6142	6142	EXO5	1.16436005245025e-14	0.389778620821101	0.02	0.011	2.54342809857233e-10	no change
6143	6143	NMB	1.2295607330262e-14	-0.2452147	0.306	0.248	2.68585246522243e-10	no change
6144	6144	SNURF	1.26333848444519e-14	0.336484705754354	0.021	0.012	2.75963658542208e-10	no change
6145	6145	IFITM1	1.26381213200007e-14	-1.045158082	0.03	0.044	2.76067122114095e-10	down
6146	6146	TEKT4	1.29529988716809e-1	0.60736707141950	0.01	0.004	2.82945307352998e-1	no

				4	9			0	change
6147	6147	SLC27A3		1.29672698519382e-14	-0.107670693	0.063	0.045	2.83257042645739e-10	no change
6148	6148	ZIK1		1.30357294752438e-14	0.520275678798572	0.013	0.006	2.84752474657225e-10	no change
6149	6149	ZNF181		1.30485980503572e-14	0.228631199812523	0.028	0.017	2.85033575812003e-10	no change
6150	6150		3-Mar	1.31491305474779e-14	-0.214211088	0.049	0.034	2.87229607679107e-10	no change
6151	6151	ANAPC11		1.31682364361272e-14	-0.105991131	0.849	0.767	2.87646956710762e-10	no change
6152	6152	CTNNBL1		1.32052334539335e-14	-0.117472986	0.213	0.174	2.88455119567723e-10	no change
6153	6153	LURAP1L		1.32102464903694e-14	-0.722367334	0.09	0.109	2.88564624335629e-10	no change
6154	6154	APBA1		1.32107402861415e-14	0.802066823098556	0.01	0.004	2.88575410810474e-10	no change
6155	6155	NEU3		1.33459368139475e-14	0.515036659372306	0.016	0.008	2.91528643763869e-10	no change
6156	6156	PDP1		1.35369526087717e-14	-0.215788462	0.098	0.074	2.9570119278601e-10	no change
6157	6157	GPBAR1		1.37071936891959e-14	-2.187703231	0.005	0.011	2.99419938946796e-10	down
6158	6158	RP11-160E2.6		1.37782923781878e-14	-0.872991075	0.083	0.102	3.00973018709134e-10	no change
6159	6159	MTHFD1L		1.37830197154288e-1	-0.260598161	0.082	0.061	3.01076282663826e-1	no

			4				0	change
6160	6160	CCDC113	1.3849982065787e-14	0.451647543350173	0.025	0.014	3.02539008245052e-10	no change
6161	6161	TOR1AIP2	1.38906504862516e-14	-0.161228069	0.426	0.344	3.03427369221679e-10	no change
6162	6162	PLA2G6	1.48249652022349e-14	0.182266620313271	0.035	0.022	3.23836539877619e-10	no change
6163	6163	TUBB2A	1.51426106998618e-14	-0.198613711	0.136	0.107	3.30775188127781e-10	no change
6164	6164	RP11-192H23.4	1.52243240384371e-14	0.372030750264308	0.018	0.009	3.32560134295619e-10	no change
6165	6165	TMCO3	1.52797379902545e-14	-0.115304123	0.117	0.091	3.33770596659119e-10	no change
6166	6166	METTL1	1.54240849956057e-14	-0.318720259	0.162	0.128	3.36923712644011e-10	no change
6167	6167	CPXM1	1.55146187747475e-14	-1.661652207	0.012	0.022	3.38901332515584e-10	down
6168	6168	AAED1	1.57433259308073e-14	-0.21065833	0.088	0.066	3.43897211632555e-10	no change
6169	6169	LRRC8C	1.58918453893334e-14	0.198738158897593	0.016	0.007	3.47141470684599e-10	no change
6170	6170	FLNB	1.65986311745754e-14	-0.11883966	0.27	0.22	3.62580499377424e-10	no change
6171	6171	MDM4	1.68812858464309e-14	-0.276900222	0.353	0.289	3.68754808029436e-10	no change
6172	6172	BCORL1	1.689023928274e-14	0.11734820638515	0.026	0.015	3.68950386892173e-1	no

				5			0	change
6173	6173	EPC1	1.73346099894095e-14	-0.152038281	0.273	0.224	3.78657220608662e-10	no change
6174	6174	CYB5D2	1.75139892182534e-14	-0.135926523	0.162	0.129	3.82575580483528e-10	no change
6175	6175	RP11-345P4.9	1.75878319825312e-14	0.1231218296057	0.044	0.03	3.84188601826412e-10	no change
6176	6176	LINC00365	1.84222896608971e-14	0.63279405993945	0.014	0.006	4.02416495352637e-10	no change
6177	6177	FBXO46	1.85025554548811e-14	0.157224713657748	0.033	0.021	4.04169821356422e-10	no change
6178	6178	PARVB	1.87509249026991e-14	-0.126154353	0.036	0.023	4.09595203574559e-10	no change
6179	6179	LDB2	1.88906572907608e-14	-1.623846482	0.009	0.018	4.12647517859379e-10	down
6180	6180	CCBL1	1.91015880153042e-14	0.269775870629418	0.027	0.016	4.17255088606305e-10	no change
6181	6181	MLLT10	1.92383794065017e-14	-0.214350543	0.174	0.14	4.20243159755623e-10	no change
6182	6182	ARL6	1.96407605952122e-14	0.149561365306558	0.034	0.021	4.29032774441816e-10	no change
6183	6183	SH2D3A	1.96701576785182e-14	-0.197323747	0.232	0.187	4.29674924329551e-10	no change
6184	6184	LCA5	1.98050564287188e-14	0.151364513400957	0.033	0.021	4.32621652628933e-10	no change
6185	6185	CCDC142	2.01306797243094e-1	0.17023807228512	0.026	0.015	4.39734567897814e-1	no

			4	9			0	change
6186	6186	GPCPD1	2.03592968210135e-14	-0.255883442	0.092	0.069	4.44728479758219e-10	no change
6187	6187	IQCH	2.0912470648092e-14	0.36341510054586	0.018	0.01	4.56812008836922e-10	no change
6188	6188	LL22NC03-86G7.1	2.15176262661232e-14	0.364834539931078	0.021	0.012	4.70031028157194e-10	no change
6189	6189	TBC1D20	2.21152181823308e-14	-0.101883979	0.159	0.128	4.83084825974833e-10	no change
6190	6190	MMP24-AS1	2.22141360030532e-14	-0.320514697	0.56	0.508	4.85245586850693e-10	no change
6191	6191	RP3-340N1.2	2.27585697075158e-14	0.149072318062691	0.051	0.035	4.97138196690975e-10	no change
6192	6192	CDX2	2.28823733709797e-14	0.286891181443439	0.017	0.009	4.99842563915681e-10	no change
6193	6193	AP001258.4	2.37753695405012e-14	0.106487323749547	0.054	0.038	5.19349172242708e-10	no change
6194	6194	TNIP1	2.38201153658208e-14	-0.133236942	0.259	0.211	5.20326600050989e-10	no change
6195	6195	NPIPB15	2.53670695122248e-14	-0.104675339	0.04	0.026	5.54118266425039e-10	no change
6196	6196	IGHMBP2	2.63108837002017e-14	0.100561521945236	0.038	0.025	5.74734943547207e-10	no change
6197	6197	RP11-111M22.2	2.65930254854289e-14	0.815662543059856	0.01	0.004	5.80898048703709e-10	no change
6198	6198	RP11-22P6.2	2.79817464746716e-1	0.28563870242262	0.022	0.013	6.11233269992727e-1	no

			4	3			0	change
6199	6199	CNP	2.94924441150739e-14	-0.103571823	0.171	0.138	6.44232949249674e-10	no change
6200	6200	TRAPPC5	2.95890313852635e-14	0.101716755007796	0.041	0.028	6.46342801579696e-10	no change
6201	6201	RCBTB1	2.96303864945584e-14	0.112755007377015	0.038	0.025	6.47246162587134e-10	no change
6202	6202	STK11IP	3.05397070271094e-14	0.151216076134434	0.032	0.02	6.67109360300177e-10	no change
6203	6203	PPT1	3.09447085184732e-14	-0.135232133	0.259	0.212	6.75956212877529e-10	no change
6204	6204	TSPAN8	3.17786819745338e-14	-0.510446777	0.274	0.288	6.94173529051716e-10	no change
6205	6205	SEC24D	3.1912231738375e-14	-0.186177217	0.119	0.093	6.97090790093063e-10	no change
6206	6206	NPHP4	3.20351335447337e-14	0.621759109681937	0.016	0.008	6.99775457151163e-10	no change
6207	6207	ZNF594	3.22373551667532e-14	0.771448881716434	0.011	0.005	7.04192786262557e-10	no change
6208	6208	C9orf91	3.24765281372133e-14	0.227475932642852	0.024	0.014	7.09417280629288e-10	no change
6209	6209	KDELC2	3.25218109444084e-14	-0.282834259	0.053	0.037	7.10406438269657e-10	no change
6210	6210	BCL9	3.38820411246294e-14	0.117791424655095	0.038	0.025	7.40119306326406e-10	no change
6211	6211	CCDC186	3.47936015914979e-1	-0.116204526	0.232	0.19	7.6003143316468e-10	no

			4					change
6212	6212	KIAA1107	3.48357176856168e-14	0.319684960022427	0.018	0.01	7.60951417124613e-10	no change
6213	6213	ANKEF1	3.52543169414461e-14	0.173612107840527	0.033	0.021	7.70095299268948e-10	no change
6214	6214	FAM114A1	3.58070324550871e-14	-0.142215137	0.265	0.216	7.82168816948923e-10	no change
6215	6215	SPG20	3.58662946656351e-14	-0.222970554	0.232	0.188	7.83463340676133e-10	no change
6216	6216	CCR7	3.61218511242329e-14	0.529550420188666	0.02	0.011	7.89045715957742e-10	no change
6217	6217	COPZ2	3.62186986412484e-14	-1.163620777	0.023	0.035	7.9116125311943e-10	down
6218	6218	ETV2	3.65615663631679e-14	-0.117989122	0.078	0.058	7.98650855637041e-10	no change
6219	6219	RP11-539I5.1	3.65719123256724e-14	0.231989036122894	0.035	0.023	7.98876852841989e-10	no change
6220	6220	PRR19	3.66473948721467e-14	0.359158217632577	0.021	0.011	8.00525693587171e-10	no change
6221	6221	APPL2	3.70289302966709e-14	-0.144294291	0.09	0.068	8.08859953400479e-10	no change
6222	6222	AC009299.3	3.76719247419771e-14	0.450278604582729	0.024	0.014	8.22905524063748e-10	no change
6223	6223	TOB2	3.82918097765698e-14	-0.130135186	0.391	0.321	8.3644629275939e-10	no change
6224	6224	NGFRAP1	3.86072011992851e-1	-0.108338733	0.909	0.833	8.43335702997183e-1	no

			4				0	change
6225	6225	OGFOD3	4.075282730412e-14	-0.183159274	0.21	0.169	8.90204759631196e-10	no change
6226	6226	CORO1A	4.10504741420704e-14	0.286260494491236	0.055	0.039	8.96706557159386e-10	no change
6227	6227	ADK	4.15976844126643e-14	-0.130184126	0.271	0.221	9.08659818310239e-10	no change
6228	6228	LMBR1L	4.18317087355337e-14	-0.100616269	0.142	0.112	9.13771845618999e-10	no change
6229	6229	ZNF432	4.28497912021327e-14	0.264882772467407	0.05	0.035	9.36010839019387e-10	no change
6230	6230	OTUD7B	4.30956576439466e-14	-0.114643296	0.149	0.119	9.4138154557437e-10	no change
6231	6231	CYP4F12	4.52757921183001e-14	-0.581292598	0.12	0.14	9.89004403032148e-10	no change
6232	6232	RP1-43E13.2	4.537439946225e-14	0.202361736656142	0.024	0.014	9.91158381853388e-10	no change
6233	6233	ZNF182	4.54424267300234e-14	0.307805511146548	0.018	0.01	9.92644369490632e-10	no change
6234	6234	SLC25A25-AS1	4.5978524198929e-14	0.316175954854106	0.015	0.007	1.0043548826014e-09	no change
6235	6235	PTPN12	4.77321890093321e-14	-0.171276576	0.187	0.151	1.04266193671985e-09	no change
6236	6236	RP11-93G5.1	4.82245512264003e-14	0.354801556711258	0.014	0.007	1.05341709698949e-09	no change
6237	6237	DNASE2	4.84434015404439e-1	-0.111966734	0.253	0.208	1.05819766324946e-0	no

			4				9	change
6238	6238	KDM4A-AS1	4.98466388834912e-14	0.718885212684133	0.012	0.005	1.08884997977098e-09	no change
6239	6239	VPREB3	5.08652202455402e-14	0.913630613092179	0.012	0.005	1.11109987104358e-09	no change
6240	6240	SMARCAL1	5.16060678755695e-14	0.101146836018548	0.036	0.024	1.12728294667394e-09	no change
6241	6241	CDKL1	5.16586098901311e-14	0.138189166775433	0.019	0.01	1.12843067444002e-09	no change
6242	6242	ACSL4	5.21965254810361e-14	-0.150570111	0.064	0.046	1.14018090260775e-09	no change
6243	6243	CRY1	5.27892725817825e-14	-0.163752844	0.086	0.065	1.15312887027646e-09	no change
6244	6244	AC092669.3	5.2807426579061e-14	0.220089731970955	0.029	0.018	1.15352542619301e-09	no change
6245	6245	C10orf12	5.34061026419638e-14	0.307582256198487	0.032	0.02	1.16660290611106e-09	no change
6246	6246	ACTN1	5.42670746193778e-14	-0.224127794	0.181	0.145	1.18540997798569e-09	no change
6247	6247	SCRN3	5.64142757485995e-14	0.193450288742882	0.069	0.052	1.23231343945241e-09	no change
6248	6248	FAM26F	5.69903289378381e-14	-0.363656444	0.05	0.035	1.24489674531813e-09	no change
6249	6249	TMEM40	5.79052503531182e-14	-0.398982072	0.358	0.36	1.26488228871351e-09	no change
6250	6250	FJX1	5.93380570340832e-1	0.30431533034230	0.027	0.016	1.29618051785251e-0	no

			4	8			9	change
6251	6251	STMN3	5.96755586412712e-14	-0.102001364	0.026	0.015	1.30355290295993e-09	no change
6252	6252	OSBPL3	6.06944397286617e-14	0.135419520470748	0.032	0.02	1.32580934143289e-09	no change
6253	6253	TLCD1	6.23206055921029e-14	-0.560470838	0.184	0.202	1.3613313085539e-09	no change
6254	6254	LEPROTL1	6.30637106304489e-14	-0.140787148	0.339	0.277	1.37756369501152e-09	no change
6255	6255	RP11-119B16.2	6.32382141784109e-14	0.130765580562367	0.054	0.039	1.38137555051321e-09	no change
6256	6256	KLHL20	6.33624679573271e-14	-0.128519776	0.094	0.072	1.38408975005985e-09	no change
6257	6257	TRIM17	6.41976722858424e-14	-0.826680977	0.012	0.022	1.40233395341194e-09	no change
6258	6258	CEP135	6.67582821363213e-14	0.131397747273643	0.044	0.03	1.4582679149858e-09	no change
6259	6259	CTNNAL1	6.79787832156562e-14	-0.160746826	0.071	0.052	1.4849285405628e-09	no change
6260	6260	TBCE	6.81568682506244e-14	0.120590712725468	0.045	0.03	1.48881863006664e-09	no change
6261	6261	ZNFX1	6.86019375254838e-14	-0.133493012	0.116	0.09	1.49854072330667e-09	no change
6262	6262	MAST2	6.94648306695858e-14	0.15041641002353	0.025	0.015	1.51738976114643e-09	no change
6263	6263	PRKAG2	6.95643856905511e-1	-0.160342835	0.119	0.093	1.5195644410244e-09	no

			4					change
6264	6264	SPTAN1	7.08143742736255e-14	-0.177814738	0.186	0.15	1.54686919163308e-09	no change
6265	6265	FBRS	7.16555400166529e-14	-0.136164172	0.132	0.104	1.56524361612377e-09	no change
6266	6266	MCMDC2	7.24661747939064e-14	0.416767457970557	0.016	0.008	1.58295112219809e-09	no change
6267	6267	SCFD2	7.50340501430695e-14	0.194886681261854	0.032	0.02	1.63904379132521e-09	no change
6268	6268	SH3BP5	7.50899828694272e-14	-0.291070329	0.149	0.118	1.64026558579977e-09	no change
6269	6269	ATF7	7.60085177089005e-14	-0.106877387	0.162	0.13	1.66033006083322e-09	no change
6270	6270	RP11-152N13.5	7.62241789646414e-14	0.245402114037702	0.03	0.019	1.66504096530363e-09	no change
6271	6271	ERCC8	7.64624455386183e-14	0.127667575985773	0.036	0.023	1.67024566034558e-09	no change
6272	6272	HTR2C	7.74058991097852e-14	0.312291087472558	0.013	0.006	1.69085446015415e-09	no change
6273	6273	SNX13	7.75832912602678e-14	-0.107228543	0.125	0.099	1.69472941428929e-09	no change
6274	6274	CTD-2547G23.4	7.79110098808451e-14	0.360439144242935	0.026	0.016	1.70188809983718e-09	no change
6275	6275	RP11-196G18.23	8.05323908751414e-14	0.500891225019277	0.023	0.013	1.75914954627659e-09	no change
6276	6276	TUBA1A	8.08787921314687e-1	-0.986467405	0.105	0.123	1.7667163353198e-09	no

			4					change
6277	6277	PRDM6	8.22721963282459e-14	0.404907852480409	0.012	0.006	1.7971538565942e-09	no change
6278	6278	TMEM30B	8.30833765694526e-14	-0.10087348	0.336	0.277	1.81487327778312e-09	no change
6279	6279	TMEM126B	8.3751472673031e-14	-0.125584372	0.312	0.256	1.82946716906969e-09	no change
6280	6280	RP11-481J2.3	8.39871831526724e-14	0.369555726834427	0.026	0.016	1.83461602878698e-09	no change
6281	6281	LINC01589	8.43545291265965e-14	0.25580166278881	0.02	0.011	1.84264033424137e-09	no change
6282	6282	SETD3	9.04065455430578e-14	-0.10231086	0.118	0.093	1.97484058084255e-09	no change
6283	6283	IQCD	9.04129413944657e-14	-0.917665423	0.029	0.042	1.97498029182071e-09	no change
6284	6284	SZT2	9.18005838317155e-14	0.146515040848872	0.04	0.027	2.00529195321999e-09	no change
6285	6285	IDNK	9.27766108778573e-14	0.103287768146838	0.038	0.025	2.02661228801591e-09	no change
6286	6286	BBC3	9.40415311262055e-14	-0.684057556	0.173	0.189	2.05424320592083e-09	no change
6287	6287	ZFP30	9.73288864191233e-14	0.362737015543304	0.019	0.01	2.12605219493933e-09	no change
6288	6288	USP20	9.80412522977665e-14	0.158946204084818	0.025	0.014	2.14161311519241e-09	no change
6289	6289	COQ7	1.04576792536628e-1	-0.150008105	0.222	0.181	2.28437545617009e-0	no

			3				9	change
6290	6290	NENF	1.0662257285039e-13	-0.241016402	0.728	0.647	2.32906348134392e-09	no change
6291	6291	PRKD1	1.08551188083446e-13	0.165545056007435	0.014	0.007	2.3711921524948e-09	no change
6292	6292	DENND5A	1.1057310030019e-13	-0.168972409	0.053	0.037	2.41535880295735e-09	no change
6293	6293	IGSF1	1.12974025022224e-13	0.44072464108021	0.012	0.005	2.46780460258545e-09	no change
6294	6294	FAM103A1	1.1540207210643e-13	-0.106126625	0.352	0.289	2.52084286309285e-09	no change
6295	6295	SSBP2	1.15518348420552e-13	-0.223673349	0.065	0.047	2.52338280289854e-09	no change
6296	6296	FAM162A	1.15720025366897e-13	-0.194164323	0.789	0.699	2.52778823411451e-09	no change
6297	6297	ASAP2	1.17564885430293e-13	-0.167474996	0.124	0.098	2.56808735733932e-09	no change
6298	6298	ZNF75D	1.19246547547394e-13	0.283915914246099	0.022	0.013	2.60482158462527e-09	no change
6299	6299	AZI2	1.21516936510853e-13	-0.238976279	0.181	0.146	2.65441596114308e-09	no change
6300	6300	IFIT2	1.23350902762172e-13	-1.32246579	0.016	0.027	2.69447711993688e-09	down
6301	6301	LAMB2	1.23435323980609e-13	-0.927236414	0.063	0.079	2.69632121703241e-09	no change
6302	6302	IPO13	1.24600636099962e-1	0.10395020761654	0.047	0.033	2.72177629496757e-0	no

			3	8			9	change
6303	6303	C1QTNF2	1.28940547066141e-13	-1.765624401	0.011	0.019	2.81657731011277e-09	down
6304	6304	TNS1	1.29378536272191e-13	-0.232211946	0.047	0.032	2.82614474632975e-09	no change
6305	6305	EFCAB7	1.29777320724191e-13	0.101992248881817	0.029	0.018	2.83485579389924e-09	no change
6306	6306	ZNF543	1.30262426049133e-13	0.326707008909137	0.015	0.008	2.84545243461727e-09	no change
6307	6307	DUSP6	1.31368611161015e-13	-0.597979667	0.246	0.257	2.86961594220122e-09	no change
6308	6308	WDR81	1.31672619616348e-13	0.13831453977931	0.028	0.017	2.87625670289951e-09	no change
6309	6309	CCDC15	1.31983330552919e-13	0.36320087428324	0.018	0.01	2.88304387259797e-09	no change
6310	6310	GPR160	1.32098428392693e-13	-0.106958336	0.276	0.229	2.88555806980998e-09	no change
6311	6311	F8	1.32289143359188e-13	0.418351253267566	0.018	0.009	2.88972404753809e-09	no change
6312	6312	SPAG9	1.45366651595711e-13	-0.123280659	0.299	0.247	3.17538913745672e-09	no change
6313	6313	LINC00648	1.47524484813988e-13	0.276218203164998	0.013	0.006	3.22252484627675e-09	no change
6314	6314	AC004540.5	1.4753134573535e-13	0.667020086854341	0.01	0.004	3.22267471624298e-09	no change
6315	6315	MFI2	1.61819848086525e-1	0.16451536890224	0.025	0.015	3.53479276160206e-0	no

			3	2			9	change
6316	6316	CPEB4	1.64365024002077e-13	-0.232540106	0.176	0.142	3.59038958430138e-09	no change
6317	6317	CACUL1	1.67798815488613e-13	-0.150276462	0.266	0.218	3.66539732553326e-09	no change
6318	6318	FADS3	1.68398596756926e-13	-0.13986362	0.069	0.051	3.6784989475583e-09	no change
6319	6319	GARNL3	1.70103258445172e-13	0.403147559018153	0.016	0.008	3.71573557747634e-09	no change
6320	6320	RHOD	1.75349302251912e-13	-0.127056199	0.182	0.148	3.83033015839077e-09	no change
6321	6321	ZNF567	1.76569986258694e-13	0.123381699656885	0.053	0.037	3.8569947798349e-09	no change
6322	6322	PIGS	1.77692372898813e-13	-0.102107628	0.143	0.114	3.88151219360167e-09	no change
6323	6323	HOXD-AS2	1.77893883429799e-13	-0.530550389	0.102	0.122	3.88591398964053e-09	no change
6324	6324	CALCOCO2	1.79594292263774e-13	-0.171817131	0.301	0.246	3.92305772020987e-09	no change
6325	6325	LHFPL2	1.84567004302582e-13	-0.26306981	0.138	0.109	4.03168164198559e-09	no change
6326	6326	FA2H	1.85438143793402e-13	-0.65714531	0.08	0.099	4.05071081302308e-09	no change
6327	6327	FAM78B	1.86535528635652e-13	0.467198144057346	0.047	0.033	4.07468208751719e-09	no change
6328	6328	MED18	1.87285111821721e-1	0.14788674829559	0.028	0.017	4.09105598263367e-0	no

			3	1			9	change
6329	6329	RP11-819C21.1	1.91416460085432e-13	0.356073996912475	0.018	0.01	4.18130115410618e-09	no change
6330	6330	NFE2L3	1.92363549526306e-13	0.187763264638527	0.061	0.045	4.20198937585264e-09	no change
6331	6331	TREM2	2.07047656316241e-13	-1.546054951	0.008	0.016	4.52274900457198e-09	down
6332	6332	ZNF678	2.07276043318669e-13	0.130236214331137	0.03	0.019	4.527737890253e-09	no change
6333	6333	RP11-758H9.2	2.08419021153031e-13	-0.668250802	0.086	0.105	4.55270509806681e-09	no change
6334	6334	RP5-866L20.1	2.08442437322997e-13	0.665991964426929	0.014	0.006	4.55321660088355e-09	no change
6335	6335	RP11-191L9.4	2.11539828677388e-13	0.34387134144064	0.01	0.004	4.62087601762885e-09	no change
6336	6336	SLC35C2	2.16340758680466e-13	-0.160586554	0.235	0.193	4.72574753261611e-09	no change
6337	6337	LLNLR-249E10.1	2.16526214998437e-13	-0.860800595	0.011	0.02	4.72979864042586e-09	no change
6338	6338	PPP1R11	2.2464866388966e-13	-0.102993862	0.42	0.344	4.90722541400572e-09	no change
6339	6339	FOCAD	2.24782218147311e-13	0.145313212335402	0.03	0.019	4.91014277320987e-09	no change
6340	6340	TRA2B	2.25008505611122e-13	-0.109189226	0.633	0.515	4.91508579656936e-09	no change
6341	6341	GPR161	2.36186457880963e-1	0.34013105226211	0.016	0.008	5.15925698595176e-0	no

			3				9	change
6342	6342	HBA2	2.36907085111627e-13	-0.155828024	0.024	0.014	5.17499836717838e-09	no change
6343	6343	EIF4E2	2.4137274141664e-13	-0.119422799	0.406	0.332	5.27254616350508e-09	no change
6344	6344	VCAM1	2.46160991010599e-13	-1.560712982	0.01	0.019	5.37714068763553e-09	down
6345	6345	BCL2L13	2.46553381347569e-13	-0.121286201	0.176	0.143	5.3857120621563e-09	no change
6346	6346	SEMA4A	2.53346735387876e-13	-0.206311334	0.159	0.128	5.53410608781277e-09	no change
6347	6347	WASH1	2.55012232413182e-13	-0.110598846	0.189	0.155	5.57048720483354e-09	no change
6348	6348	ANKRD36	2.57545259377006e-13	0.40675671390076	0.016	0.008	5.62581864583133e-09	no change
6349	6349	AC007773.2	2.61083086918784e-13	0.383345060704245	0.019	0.011	5.70309895065391e-09	no change
6350	6350	MAN2A2	2.66266447471793e-13	0.288235385015729	0.026	0.016	5.81632427857385e-09	no change
6351	6351	TOP3B	2.6648380435712e-13	0.171419195761039	0.051	0.036	5.82107222237693e-09	no change
6352	6352	LGALS9	2.7079739845583e-13	-0.227044245	0.316	0.255	5.91529837186915e-09	no change
6353	6353	DAPK3	2.71695199633295e-13	-0.176982275	0.151	0.121	5.9349099407897e-09	no change
6354	6354	RAB22A	2.75863599398954e-1	-0.171552263	0.25	0.205	6.02596446527076e-0	no

			3				9	change
6355	6355	ARFGAP3	2.83404947577499e-13	-0.152244868	0.176	0.142	6.19069767488289e-09	no change
6356	6356	FEZ1	2.84674147828862e-13	-0.297993297	0.057	0.04	6.21842208517365e-09	no change
6357	6357	BRD2	2.89813146414783e-13	-0.385482402	0.588	0.538	6.33067837028452e-09	no change
6358	6358	CTD-2517O10.6	2.9338613612944e-13	0.333623386181542	0.038	0.026	6.40872675761149e-09	no change
6359	6359	GPR143	3.05756249041298e-13	0.234903597170464	0.026	0.015	6.67893950405811e-09	no change
6360	6360	ARTN	3.0588673100026e-13	-1.238900924	0.013	0.022	6.68178975196969e-09	down
6361	6361	TMEM248	3.06504765617803e-13	-0.120805241	0.372	0.306	6.6952901001553e-09	no change
6362	6362	TRAPPC2P1	3.17150096513296e-13	-0.140089486	0.186	0.151	6.92782670823644e-09	no change
6363	6363	TRIM8	3.24283993304421e-13	-0.134831563	0.141	0.112	7.08365954974178e-09	no change
6364	6364	WDR19	3.26388533432559e-13	0.162897703604851	0.027	0.016	7.12963112430081e-09	no change
6365	6365	NEK11	3.39381171390556e-13	0.102094636213611	0.035	0.023	7.41344230785532e-09	no change
6366	6366	ODF3B	3.60429730363536e-13	-0.672688244	0.131	0.15	7.87322703006107e-09	no change
6367	6367	CNN3	3.64106271191167e-1	-0.383758278	0.491	0.463	7.95353738789986e-0	no

			3				9	change
6368	6368	P2RY2	3.65264206293794e-13	-0.604378383	0.045	0.061	7.97883132228163e-09	no change
6369	6369	P3H1	3.66406007291428e-13	-0.123303774	0.04	0.027	8.00377282327395e-09	no change
6370	6370	WIPI2	3.69088148914182e-13	-0.126046728	0.313	0.256	8.0623615248814e-09	no change
6371	6371	AC084219.4	3.70980142325727e-13	0.61896792190207	0.012	0.005	8.10369022896318e-09	no change
6372	6372	LSM11	3.74553379009537e-13	0.398954849863724	0.014	0.007	8.18174401108434e-09	no change
6373	6373	UBR4	3.94780880121038e-13	-0.204169654	0.249	0.203	8.62359354536396e-09	no change
6374	6374	HSD3B1	3.9662787093889e-13	0.650637104278025	0.016	0.008	8.66393921278911e-09	no change
6375	6375	TADA2A	4.25533671534054e-13	0.10588547906683	0.036	0.024	9.29535752098988e-09	no change
6376	6376	RP11-497H16.9	4.26561525412232e-13	0.197246814264027	0.016	0.008	9.31780996110479e-09	no change
6377	6377	GRHL2	4.2941670122773e-13	-0.184126359	0.129	0.103	9.38017842161853e-09	no change
6378	6378	PARS2	4.39581310795323e-13	0.535173718746774	0.013	0.006	9.60221415301303e-09	no change
6379	6379	HN1	4.47094003933007e-13	-0.138827941	0.772	0.708	9.7663214219126e-09	no change
6380	6380	ARHGEF4	4.50906199012513e-1	0.50551090984690	0.016	0.008	9.84959501122934e-0	no

			3	5			9	change
6381	6381	TSC22D3	4.54402588842712e-13	-0.638954259	0.417	0.4	9.92597015068019e-09	no change
6382	6382	RRAS	4.56209172588683e-13	-0.641255881	0.106	0.125	9.9654331660272e-09	no change
6383	6383	FAM104B	4.60278990437139e-13	-0.125032019	0.277	0.23	1.00543342671089e-08	no change
6384	6384	TFPI	4.67796572846742e-13	-0.648841739	0.166	0.13	1.02185483372642e-08	no change
6385	6385	ZNF408	4.8955640181343e-13	-0.106168509	0.049	0.034	1.06938700412126e-08	no change
6386	6386	PPT2	4.95874210708579e-13	0.154063591138829	0.027	0.016	1.08318762587182e-08	no change
6387	6387	LINC00709	5.00320322736457e-13	0.624205649216679	0.012	0.005	1.09289971298552e-08	no change
6388	6388	ADAMTS4	5.07238792593831e-13	-1.385658971	0.013	0.022	1.10801241854196e-08	down
6389	6389	PKD1	5.07976903382794e-13	-0.126061284	0.05	0.036	1.10962474774937e-08	no change
6390	6390	CCPG1	5.09480153258662e-13	-0.330589611	0.15	0.119	1.11290844677822e-08	no change
6391	6391	USP53	5.09729525562779e-13	-0.43269364	0.237	0.192	1.11345317563933e-08	no change
6392	6392	CD40	5.15886749913502e-13	-0.571421888	0.154	0.173	1.12690301651105e-08	no change
6393	6393	SLC9A3R1	5.19780837502383e-1	-0.667134104	0.193	0.208	1.13540926144021e-0	no

			3				8	change
6394	6394	HVCN1	5.45796174501487e-13	0.235697554551887	0.024	0.014	1.19223716358105e-08	no change
6395	6395	CD164	5.48128684447337e-13	-0.196839133	0.758	0.678	1.19733229830676e-08	no change
6396	6396	ZNF343	5.60573981936411e-13	0.415459037006946	0.016	0.008	1.2245178061419e-08	no change
6397	6397	SMG9	5.66754930230658e-13	-0.139261012	0.064	0.047	1.23801946959585e-08	no change
6398	6398	C7	5.70078011399066e-13	-1.3123502	0.016	0.026	1.24527840810012e-08	down
6399	6399	RP4-594I10.3	5.74949301509785e-13	0.164712147836234	0.025	0.015	1.25591925421797e-08	no change
6400	6400	RP5-1112D6.4	5.81333061933586e-13	0.728122763237454	0.012	0.005	1.26986394048773e-08	no change
6401	6401	LPCAT4	5.87180952209094e-13	-0.128421223	0.233	0.193	1.28263807200555e-08	no change
6402	6402	CHST7	5.9093620222648e-13	0.141082042191128	0.02	0.011	1.29084104014352e-08	no change
6403	6403	RP11-776H12.1	5.93769001105582e-13	0.191019843656224	0.038	0.026	1.29702900601503e-08	no change
6404	6404	TTC13	6.14055110512192e-13	0.132513269236438	0.027	0.016	1.34134198340283e-08	no change
6405	6405	NRAS	6.25717230986981e-13	-0.130799451	0.162	0.132	1.36681671936796e-08	no change
6406	6406	CYCS	6.34898200498122e-1	-0.140087723	0.903	0.85	1.3868716291681e-08	no

			3					change
6407	6407	CCDC24	6.40203703053932e-13	-0.122064023	0.1	0.078	1.39846096895101e-08	no change
6408	6408	DNAJC17	6.50252066278286e-13	-0.182460211	0.127	0.1	1.42041061357829e-08	no change
6409	6409	LRRC8A	6.55002555460782e-13	-0.200413301	0.147	0.118	1.43078758214853e-08	no change
6410	6410	TFRC	6.75392397663123e-13	-0.164993131	0.254	0.209	1.47532715345533e-08	no change
6411	6411	BIN1	6.80761242644428e-13	-0.991237948	0.042	0.057	1.48705485843249e-08	no change
6412	6412	MPP5	6.82855225867046e-13	-0.107378562	0.117	0.093	1.49162895538398e-08	no change
6413	6413	AP001505.10	7.21002971361603e-13	0.594336388754694	0.012	0.005	1.57495889064229e-08	no change
6414	6414	LINC01564	7.47760014235798e-13	0.143258735634681	0.057	0.041	1.63340697509668e-08	no change
6415	6415	LIPA	7.69762189267788e-13	-0.249461272	0.25	0.204	1.68146852623656e-08	no change
6416	6416	FITM2	7.7748339331844e-13	0.313144588738433	0.023	0.013	1.6983347243648e-08	no change
6417	6417	EPHA3	7.99791790951973e-13	-1.638143557	0.01	0.018	1.74706518815549e-08	down
6418	6418	RAB32	7.99939710758113e-13	-0.588651785	0.247	0.257	1.74738830418002e-08	no change
6419	6419	ZDHHC6	8.07538879183492e-1	-0.126720826	0.203	0.166	1.76398792768842e-0	no

			3				8	change
6420	6420	PTX3	8.09314120162403e-13	-1.233927845	0.029	0.041	1.76786576408275e-08	down
6421	6421	TBCCD1	8.1475484164213e-13	0.181172813269463	0.024	0.015	1.77975047608307e-08	no change
6422	6422	CTB-152G17.6	8.35147559729784e-13	0.153412405597225	0.023	0.014	1.82429632947374e-08	no change
6423	6423	R3HDM4	8.3719259054718e-13	-0.145927999	0.215	0.176	1.82876349479126e-08	no change
6424	6424	SHF	8.39006204952905e-13	0.137486319574509	0.017	0.009	1.83272515409913e-08	no change
6425	6425	RP11-486G15.2	8.39368650823669e-13	0.402787179727958	0.016	0.008	1.83351688085922e-08	no change
6426	6426	CYP2W1	8.56016594163915e-13	-0.974137269	0.015	0.024	1.86988264829166e-08	no change
6427	6427	PER1	8.86405974487894e-13	-0.297998825	0.118	0.092	1.93626521067136e-08	no change
6428	6428	SERPINF2	9.03445693403497e-13	-1.098454163	0.008	0.016	1.9734867726706e-08	down
6429	6429	ZNHIT1	9.10565802990641e-13	-0.200434313	0.747	0.663	1.98903994005276e-08	no change
6430	6430	RP11-757F18.5	9.11217372590862e-13	0.244413470420659	0.021	0.012	1.99046322868748e-08	no change
6431	6431	SLC1A7	9.1298940860342e-13	-1.693774786	0.005	0.011	1.99433406415331e-08	down
6432	6432	STIL	9.50187400533653e-1	0.39546311964239	0.01	0.004	2.07558935772571e-0	no

			3	9			8	change
6433	6433	PRPF39	9.73705360214683e-13	-0.169891965	0.062	0.045	2.12696198885295e-08	no change
6434	6434	RP4-735C1.4	1.01065754480958e-12	0.372847674953513	0.039	0.026	2.20768034088205e-08	no change
6435	6435	PPP1R1C	1.0117698496805e-12	0.141539923154817	0.039	0.027	2.21011005964207e-08	no change
6436	6436	ZNF506	1.03565516557181e-12	-0.101134895	0.146	0.118	2.26228514367507e-08	no change
6437	6437	AKT1S1	1.04907504189633e-12	-0.147124804	0.269	0.221	2.29159952151833e-08	no change
6438	6438	IL17RC	1.06047063371359e-12	-0.143573046	0.092	0.071	2.31649205228397e-08	no change
6439	6439	SLC31A2	1.06355521246821e-12	-0.820592643	0.045	0.06	2.32323000611557e-08	no change
6440	6440	CYB561D1	1.06619871655131e-12	0.311967233217417	0.02	0.011	2.32900447643467e-08	no change
6441	6441	NUDT6	1.09758401502615e-12	0.181067207629972	0.024	0.014	2.39756252242313e-08	no change
6442	6442	PIK3R2	1.10154219625035e-12	0.329447813686007	0.02	0.011	2.40620877348927e-08	no change
6443	6443	CCDC12	1.11772566748775e-12	-0.324787684	0.483	0.461	2.44155994806023e-08	no change
6444	6444	TTC22	1.12748355717104e-12	0.12339195993561	0.052	0.037	2.46287508228441e-08	no change
6445	6445	MTM1	1.13742798414738e-1	0.15946039941262	0.035	0.023	2.48459768857154e-0	no

			2	3			8	change
6446	6446	ATP6V0B	1.15254049913215e-12	-0.150017378	0.768	0.692	2.51760946630428e-08	no change
6447	6447	ZNF582	1.16343804636165e-12	0.37132664212563	0.022	0.013	2.54141406847238e-08	no change
6448	6448	NR2F2-AS1	1.19567986050783e-12	0.352955443644184	0.023	0.013	2.6118430872933e-08	no change
6449	6449	SHROOM3	1.19708106564299e-12	-1.10999151	0.026	0.038	2.61490387979055e-08	down
6450	6450	ATF4	1.20143300240518e-12	-0.251339018	0.645	0.583	2.62441025045388e-08	no change
6451	6451	SMLR1	1.22695381625194e-12	-1.097358536	0.009	0.017	2.68015791622075e-08	down
6452	6452	CH507-254M2.3	1.25682787364011e-12	0.208784144770711	0.03	0.019	2.74541480717947e-08	no change
6453	6453	MT1E	1.30062739348648e-12	-1.403601059	0.098	0.115	2.84109047833188e-08	down
6454	6454	HYPK	1.31230510151855e-12	-0.178376373	0.055	0.04	2.86659926375712e-08	no change
6455	6455	SYNRG	1.3201023467245e-12	-0.171149293	0.202	0.164	2.883631566185e-08	no change
6456	6456	CLDN16	1.33314552892603e-12	0.244845871300775	0.023	0.014	2.91212309338602e-08	no change
6457	6457	LSAMP	1.33572193053448e-12	-1.293597173	0.015	0.024	2.91775098505952e-08	down
6458	6458	RP11-10C24.3	1.33822462625761e-1	0.28087189344550	0.021	0.012	2.92321787359713e-0	no

			2	6			8	change
6459	6459	RP11-284F21.10	1.34327392286668e-12	0.465559712792113	0.017	0.009	2.93424755710998e-08	no change
6460	6460	RBM6	1.49653898982574e-12	-0.195496168	0.25	0.206	3.26903976937534e-08	no change
6461	6461	INMT	1.50840296062284e-12	-2.022555259	0.005	0.012	3.29495542718452e-08	down
6462	6462	LYSMD3	1.53020114034821e-12	-0.147843345	0.07	0.052	3.34257137097664e-08	no change
6463	6463	EML1	1.55858396537476e-12	-1.07986594	0.022	0.033	3.40457081396462e-08	down
6464	6464	TNKS1BP1	1.56317584694092e-12	-0.187886115	0.199	0.162	3.41460132005775e-08	no change
6465	6465	HLA-DQA1	1.5659081478881e-12	-0.63752215	0.072	0.089	3.42056975824676e-08	no change
6466	6466	RP13-463N16.6	1.6757009050846e-12	0.69481687569504	0.012	0.005	3.66040105706679e-08	no change
6467	6467	PAQR6	1.68014911153005e-12	0.446744187766016	0.016	0.009	3.67011771922624e-08	no change
6468	6468	ZW10	1.68770383388403e-12	0.167150053432969	0.031	0.02	3.68662025473628e-08	no change
6469	6469	HIST3H2BB	1.72272226430491e-12	-1.270635383	0.005	0.011	3.76311451414765e-08	down
6470	6470	SPINK5	1.73060770678558e-12	0.260808382021932	0.029	0.019	3.78033947470243e-08	no change
6471	6471	WARS	1.75542558265117e-1	-0.183387184	0.086	0.066	3.83455164274322e-0	no

			2				8	change
6472	6472	ARFIP2	1.84917628308429e-12	-0.193411718	0.192	0.157	4.03934067276933e-08	no change
6473	6473	HIVEP2	1.88996478712507e-12	-0.108281645	0.087	0.067	4.12843908099601e-08	no change
6474	6474	AVEN	1.93539454180862e-12	0.239026675396037	0.039	0.027	4.22767583712675e-08	no change
6475	6475	SCAND1	1.93708164486697e-12	-0.19996186	0.719	0.641	4.2313611450474e-08	no change
6476	6476	FMNL2	1.95808663274306e-12	-0.275498198	0.03	0.019	4.27724444056394e-08	no change
6477	6477	HIST1H2AG	1.96272042462612e-12	0.372496582087527	0.019	0.01	4.2873664955533e-08	no change
6478	6478	RNF13	2.09482201046738e-12	-0.204602717	0.339	0.278	4.57592919966495e-08	no change
6479	6479	MAN2B1	2.137213570431e-12	0.179303874982344	0.021	0.012	4.66852932324949e-08	no change
6480	6480	RP11-890B15.3	2.17672101282597e-12	0.437829403569591	0.01	0.004	4.75482938041705e-08	no change
6481	6481	RGS10	2.18765448131879e-12	-0.350104442	0.157	0.127	4.77871244899277e-08	no change
6482	6482	CCDC157	2.18790217599276e-12	0.116224714610895	0.031	0.02	4.77925351323859e-08	no change
6483	6483	AGBL2	2.1899466398191e-12	0.379640185886662	0.016	0.008	4.78371944002084e-08	no change
6484	6484	ZNF557	2.19574679608381e-1	-0.195573913	0.063	0.046	4.79638930136547e-0	no

			2				8	change
6485	6485	LLGL1	2.20996952036034e-12	0.130289631508798	0.025	0.015	4.82745742027513e-08	no change
6486	6486	USP19	2.21728708975314e-12	0.139731233055007	0.043	0.03	4.84344191885675e-08	no change
6487	6487	RUNX1	2.24235572829086e-12	-0.126546921	0.359	0.297	4.89820185287855e-08	no change
6488	6488	PRPF18	2.27431498500443e-12	-0.21065716	0.196	0.16	4.96801365324367e-08	no change
6489	6489	TNFRSF14	2.28627232291955e-12	-0.145045741	0.275	0.227	4.99413326218546e-08	no change
6490	6490	PDLIM2	2.3099031488343e-12	-0.735726388	0.099	0.117	5.04575243831364e-08	no change
6491	6491	KMT2E-AS1	2.37821043950484e-12	-0.658250509	0.103	0.121	5.19496288405437e-08	no change
6492	6492	ZNF71	2.46525608276462e-12	0.236159526920925	0.027	0.017	5.38510538719103e-08	no change
6493	6493	GBE1	2.46913763252891e-12	-0.230388681	0.126	0.1	5.39358424449615e-08	no change
6494	6494	DNAJB4	2.48866952056765e-12	-0.889701358	0.183	0.196	5.43624970072797e-08	no change
6495	6495	PCDHB13	2.52504853107081e-12	0.41717109975084	0.01	0.004	5.51571601127108e-08	no change
6496	6496	NHSL1	2.57247421104148e-12	-0.158951387	0.08	0.061	5.61931266659902e-08	no change
6497	6497	C1RL-AS1	2.61534228807539e-1	0.18177695111831	0.022	0.013	5.71295369407188e-0	no

			2	7			8	change
6498	6498	CYB5D1	2.67834798328042e-12	-0.183180617	0.063	0.046	5.85058333467775e-08	no change
6499	6499	MARC1	2.73432602819705e-12	0.128788283464691	0.063	0.047	5.97286177599364e-08	no change
6500	6500	SLC11A1	2.76459238143278e-12	-1.031101181	0.027	0.039	6.03897559800176e-08	down
6501	6501	ALDH1A1	2.77214217788134e-12	-1.02156586	0.024	0.035	6.05546737336399e-08	down
6502	6502	CFAP53	2.82759616256349e-12	0.248862022245536	0.024	0.015	6.17660105750369e-08	no change
6503	6503	DGAT1	2.8461510229515e-12	-0.15235957	0.088	0.068	6.21713229453526e-08	no change
6504	6504	UBXN8	2.88128478307237e-12	-0.190849378	0.082	0.062	6.29387848014328e-08	no change
6505	6505	TNFSF13B	3.00857276926979e-12	-1.162132688	0.029	0.04	6.57192635719293e-08	down
6506	6506	EIF1AD	3.01596792213803e-12	-0.201085428	0.116	0.092	6.58808032911831e-08	no change
6507	6507	MTA2	3.06796533019091e-12	-0.173094115	0.099	0.077	6.70166346726902e-08	no change
6508	6508	LDHD	3.09536119788083e-12	-1.15056518	0.014	0.023	6.76150700065089e-08	down
6509	6509	TRIM73	3.11774284867577e-12	0.247370192089382	0.029	0.018	6.81039747864734e-08	no change
6510	6510	JAK2	3.1948522563506e-12	-0.117177039	0.029	0.019	6.97883526877224e-0	no

							8	change
6511	6511	MAP1A	3.20603960641048e-12	-1.460977582	0.014	0.022	7.00327291624306e-08	down
6512	6512	KRBA2	3.28418675378106e-12	0.567205564265236	0.012	0.006	7.17397754495935e-08	no change
6513	6513	CTD-3185P2.1	3.36734279740717e-12	0.523056819237373	0.011	0.005	7.35562360665623e-08	no change
6514	6514	ZNF414	3.37863333827272e-12	-0.157288387	0.102	0.08	7.38028666412292e-08	no change
6515	6515	PPP1CB	3.45467934326537e-12	-0.15519861	0.716	0.601	7.54640155742887e-08	no change
6516	6516	RAB3A	3.46160653953609e-12	0.160648923400143	0.044	0.031	7.56153332496263e-08	no change
6517	6517	SNN	3.57475888432589e-12	-0.165474797	0.106	0.083	7.80870330692148e-08	no change
6518	6518	SPRR2D	3.65948589887731e-12	-1.662101598	0.007	0.014	7.99378099750759e-08	down
6519	6519	ZP3	3.80480720132925e-12	0.162889644555392	0.035	0.023	8.31122085058361e-08	no change
6520	6520	PPCS	3.8085673613608e-12	-0.122557159	0.43	0.35	8.31943454415653e-08	no change
6521	6521	LINC01138	3.82707157911463e-12	0.286407050504787	0.024	0.014	8.359855157418e-08	no change
6522	6522	SAP30BP	3.88578403797262e-12	-0.174292364	0.325	0.267	8.4881066525474e-08	no change
6523	6523	AMER1	3.9315124395331e-12	0.62358592804342	0.01	0.004	8.5879957729161e-08	no

				2				change
6524	6524	LRRIQ3	4.00711470575733e-12	0.376755171402885	0.021	0.012	8.75314136325631e-08	no change
6525	6525	PEX7	4.07726044175201e-12	-0.101480966	0.112	0.089	8.9063677089631e-08	no change
6526	6526	MFI2-AS1	4.1107740088112e-12	0.278128555340333	0.018	0.01	8.97957474484718e-08	no change
6527	6527	ZSCAN30	4.1274970874795e-12	0.165902062538082	0.024	0.015	9.01610463789022e-08	no change
6528	6528	RP5-894A10.2	4.16555967281547e-12	0.364471741660894	0.012	0.006	9.09924854929811e-08	no change
6529	6529	SYT2	4.1807063541716e-12	-0.765907623	0.041	0.055	9.13233496005244e-08	no change
6530	6530	H2AFX	4.18463371276222e-12	-0.143554	0.207	0.171	9.14091388215779e-08	no change
6531	6531	RAB29	4.22572865223172e-12	-0.18594931	0.15	0.122	9.23068166793496e-08	no change
6532	6532	ANK2	4.24585513048934e-12	-1.142876778	0.017	0.027	9.27464594704091e-08	down
6533	6533	SUCLG2	4.46097504140747e-12	-0.11590384	0.289	0.241	9.74455388045048e-08	no change
6534	6534	EPHX1	4.4760853327646e-12	-0.210842686	0.386	0.316	9.77756080089098e-08	no change
6535	6535	AOX1	4.53190871530665e-12	-1.991281867	0.006	0.012	9.89950139771584e-08	down
6536	6536	CXCL14	4.53265299504036e-1	-2.132337123	0.033	0.045	9.90112720236616e-0	down

			2				8	
6537	6537	PNRC1	4.56882721603238e-12	-0.368598072	0.695	0.646	9.98014617070113e-08	no change
6538	6538	TOLLIP	4.58522585205273e-12	-0.116525525	0.351	0.292	1.0015967351224e-07	no change
6539	6539	ZNF253	4.7084783958349e-12	0.217443371846961	0.032	0.021	1.02852002078618e-07	no change
6540	6540	RP11-107M16.2	4.71056279112825e-12	-0.952290207	0.013	0.022	1.02897533609405e-07	no change
6541	6541	AADAC	4.73505980452747e-12	-0.850859032	0.024	0.035	1.03432646370098e-07	no change
6542	6542	XXbac-BPG299F13.17	4.90846100867856e-12	0.719430260160991	0.01	0.004	1.07220422273574e-07	no change
6543	6543	RNF114	5.00218995821543e-12	-0.184190678	0.323	0.265	1.09267837447258e-07	no change
6544	6544	HIST1H1D	5.16608263476718e-12	0.392816834795538	0.012	0.006	1.12847909073854e-07	no change
6545	6545	C1QA	5.36332576874129e-12	-1.635414244	0.037	0.05	1.17156488092385e-07	down
6546	6546	TBC1D25	5.39094706589955e-12	-0.103827147	0.052	0.038	1.1775984770751e-07	no change
6547	6547	SLC35E3	5.3912161772411e-12	-0.277080703	0.24	0.197	1.17765726175655e-07	no change
6548	6548	MAP1B	5.44327748966903e-12	-1.192067414	0.027	0.039	1.1890295348433e-07	down
6549	6549	G0S2	5.65239158413896e-1	-0.890578007	0.128	0.147	1.23470841763932e-0	no

			2				7	change
6550	6550	MBD1	5.65481012706409e-12	-0.105462315	0.145	0.118	1.23523672415588e-07	no change
6551	6551	ZNF286B	5.6904949039126e-12	0.426506300039033	0.01	0.004	1.24303170681067e-07	no change
6552	6552	CTD-3113P16.11	5.78138627481211e-12	0.192627037342791	0.016	0.009	1.26288601786996e-07	no change
6553	6553	ZNF792	5.805608145813e-12	0.428514561164006	0.013	0.006	1.26817704337139e-07	no change
6554	6554	ANKRD22	5.8759788076813e-12	-0.638244504	0.076	0.093	1.2835488107499e-07	no change
6555	6555	BCKDHA	5.96285081479071e-12	0.3979512638514	0.014	0.007	1.30252513198288e-07	no change
6556	6556	ACVRL1	6.16532996039543e-12	-1.693043723	0.011	0.019	1.34675467654878e-07	down
6557	6557	MED29	6.19471719487571e-12	-0.188013557	0.205	0.168	1.35317402404865e-07	no change
6558	6558	SNAP47	6.27284747964785e-12	-0.114504993	0.117	0.093	1.37024080345428e-07	no change
6559	6559	UBA7	6.27416624372585e-12	-0.168943743	0.074	0.056	1.37052887427947e-07	no change
6560	6560	VIPAS39	6.42348575724821e-12	0.103628216890177	0.041	0.028	1.4031462288133e-07	no change
6561	6561	TAF5	6.46029075012783e-12	0.252505724127508	0.015	0.008	1.41118591145792e-07	no change
6562	6562	KCNK6	6.59060847758184e-1	-0.201487758	0.156	0.127	1.43965251584298e-0	no

			2				7	change
6563	6563	PDZRN3	6.8428293943393e-12	-1.524977991	0.009	0.016	1.49474765289948e-07	down
6564	6564	RP1-223E5.4	6.85301744427903e-12	0.261416346231612	0.023	0.014	1.49697313052831e-07	no change
6565	6565	PITX1	6.87078229933543e-12	-0.11009253	0.118	0.095	1.50085368546683e-07	no change
6566	6566	ZNF667	6.8864823106275e-12	0.345317381119782	0.015	0.008	1.50428319593347e-07	no change
6567	6567	ZNF570	7.14378192496507e-12	0.184274747358001	0.022	0.014	1.56048772368937e-07	no change
6568	6568	CTD-3131K8.2	7.40647002156845e-12	0.178897042739856	0.025	0.015	1.61786931151141e-07	no change
6569	6569	PDE12	7.45428528435637e-12	-0.127397034	0.094	0.073	1.62831407751481e-07	no change
6570	6570	ZMAT3	7.60850815766097e-12	-0.29928898	0.12	0.095	1.66200252195946e-07	no change
6571	6571	ZNRF3-AS1	7.63626886784861e-12	0.400443736781233	0.012	0.006	1.66806657149285e-07	no change
6572	6572	KPNA5	7.72789265025807e-12	-0.108686366	0.065	0.048	1.68808087052237e-07	no change
6573	6573	MEF2A	7.86942776595242e-12	-0.177796897	0.123	0.098	1.71899780119465e-07	no change
6574	6574	RP11-834C11.4	7.90916914426839e-12	-1.335249479	0.011	0.019	1.72767890787399e-07	down
6575	6575	C19orf57	7.96707627524002e-1	0.56449699611622	0.012	0.005	1.74032814156343e-0	no

			2	3			7	change
6576	6576	SUMF1	8.10977331280864e-12	-0.127153033	0.168	0.138	1.77149888244992e-07	no change
6577	6577	COL1A1	8.13842760855288e-12	-1.344437866	0.121	0.136	1.77775812681229e-07	down
6578	6578	GPR82	8.38691830423557e-12	-0.112953451	0.039	0.027	1.83203843437722e-07	no change
6579	6579	CEP85	8.49602998131844e-12	0.130675233577531	0.03	0.019	1.8558727891192e-07	no change
6580	6580	BMP4	8.51269052918132e-12	-1.484511359	0.017	0.026	1.85951211919437e-07	down
6581	6581	LINC00526	8.53596440325382e-12	0.477387082431567	0.014	0.007	1.86459606424676e-07	no change
6582	6582	CEBPG	8.60548389412553e-12	-0.192745904	0.274	0.227	1.87978190183278e-07	no change
6583	6583	MTCP1	8.64787127913826e-12	-1.034885706	0.007	0.013	1.88904100221496e-07	down
6584	6584	WDR53	9.06321072735031e-12	0.204110728209585	0.032	0.021	1.9797677512824e-07	no change
6585	6585	CCDC183	9.06897613241108e-12	-0.787878863	0.016	0.025	1.98102714636388e-07	no change
6586	6586	KB-1460A1.5	9.40532787668445e-12	0.754405540011651	0.014	0.007	2.05449982138295e-07	no change
6587	6587	MIATNB	9.70274435727858e-12	0.334878129734796	0.018	0.01	2.11946747740393e-07	no change
6588	6588	AKAP12	9.71358564716448e-1	-0.975264854	0.06	0.075	2.12183564876661e-0	no

			2				7	change
6589	6589	RP11-196G18.24	9.90916078719546e-12	0.41012182815743	0.016	0.008	2.16455708235498e-07	no change
6590	6590	GNS	9.98260725237425e-12	-0.135985176	0.228	0.19	2.18060072820863e-07	no change
6591	6591	AC011043.1	9.98705476026833e-12	0.228336501731495	0.029	0.019	2.18157224183302e-07	no change
6592	6592	NAAA	1.00830294115627e-11	-0.124654345	0.07	0.053	2.20253694466175e-07	no change
6593	6593	VSIG1	1.02012736056293e-11	0.171234597141268	0.019	0.011	2.22836620641367e-07	no change
6594	6594	VNN2	1.02769265699311e-11	0.286424196548516	0.018	0.01	2.24489183993574e-07	no change
6595	6595	SH3BP5-AS1	1.05626609466373e-11	0.608825277092273	0.011	0.005	2.30730765718345e-07	no change
6596	6596	RAB33B	1.06070570054302e-11	-0.105403163	0.048	0.034	2.31700553226617e-07	no change
6597	6597	PASK	1.10514707897165e-11	0.361774315618254	0.016	0.009	2.41408327930567e-07	no change
6598	6598	ABHD14B	1.1253532414545e-11	-0.1204169	0.338	0.28	2.45822162063321e-07	no change
6599	6599	LBH	1.13335049026098e-11	-0.186395272	0.046	0.032	2.47569081092608e-07	no change
6600	6600	RP4-601P9.2	1.16604136186657e-11	0.625406324427256	0.015	0.008	2.54710075086133e-07	no change
6601	6601	OVOL1-AS1	1.1669764926376e-11	-0.966062112	0.008	0.016	2.54914345051758e-0	no

							7	change
6602	6602	CTC-479C5.12	1.21672505779642e-11	0.165861838646461	0.018	0.01	2.6578142162505e-07	no change
6603	6603	LINC01468	1.24545421684123e-11	-1.368585971	0.005	0.011	2.72057019126798e-07	down
6604	6604	AC021218.2	1.26139004967469e-11	0.648990746746874	0.01	0.004	2.7553804245094e-07	no change
6605	6605	CLIC2	1.28427031845022e-11	-1.184760914	0.016	0.025	2.80536008362265e-07	down
6606	6606	ARL10	1.2907684269542e-11	0.11555963688092	0.013	0.006	2.81955455183876e-07	no change
6607	6607	PACSIN2	1.2955623841054e-11	-0.145063508	0.132	0.107	2.83002647183984e-07	no change
6608	6608	LYZ	1.29844836758636e-11	-0.404616252	0.063	0.079	2.83633061415564e-07	no change
6609	6609	MYL9	1.32118778742889e-11	-0.306553089	0.132	0.105	2.88600260285967e-07	no change
6610	6610	BROX	1.33941442908209e-11	-0.203650857	0.307	0.253	2.92581687888691e-07	no change
6611	6611	ARHGAP44	1.3395243203957e-11	0.495025305225663	0.012	0.006	2.92605692547237e-07	no change
6612	6612	CYHR1	1.38706268786435e-11	-0.180473622	0.286	0.236	3.0298997353709e-07	no change
6613	6613	C21orf2	1.38955875728356e-11	-0.185866202	0.159	0.129	3.0353521494102e-07	no change
6614	6614	DUSP9	1.40937444194911e-1	0.16281798018194	0.033	0.022	3.07863753099364e-0	no

			1	8			7	change
6615	6615	P2RY1	1.45443939081321e-11	0.145490769171071	0.018	0.01	3.17707740529237e-07	no change
6616	6616	CHST8	1.46660756428845e-11	0.556356960196153	0.012	0.006	3.20365756343169e-07	no change
6617	6617	AGTPBP1	1.48411516788773e-11	0.155828105628164	0.018	0.01	3.24190117273395e-07	no change
6618	6618	SCNN1A	1.5197572006699e-11	-0.479074728	0.138	0.157	3.31975762914333e-07	no change
6619	6619	AREL1	1.52504986866899e-11	0.124593205590805	0.034	0.023	3.33131893312054e-07	no change
6620	6620	SLC8B1	1.52986101146789e-11	-0.118341229	0.088	0.068	3.34182839345046e-07	no change
6621	6621	AC093375.1	1.54295738856163e-11	0.428479708422787	0.018	0.01	3.37043611957403e-07	no change
6622	6622	PTPRQ	1.56016940667653e-11	0.223477385053789	0.016	0.009	3.40803405194422e-07	no change
6623	6623	THSD1	1.56253760612135e-11	0.339046724515415	0.01	0.004	3.41320714681147e-07	no change
6624	6624	RP11-346C20.3	1.57556033672861e-11	0.272443862517816	0.012	0.006	3.44165399954997e-07	no change
6625	6625	ZNF853	1.57644091815801e-11	0.211226817166123	0.01	0.005	3.44357754162436e-07	no change
6626	6626	ZNF829	1.60758918860103e-11	0.493537078153927	0.013	0.006	3.51161782358008e-07	no change
6627	6627	MFSD1	1.63407613140745e-1	-0.206689592	0.229	0.189	3.56947590144642e-0	no

			1				7	change
6628	6628	POFUT2	1.66243088262778e-11	-0.137784136	0.061	0.045	3.63141402001213e-07	no change
6629	6629	TMEM163	1.7024773023627e-11	0.404873448797129	0.032	0.022	3.71889141928108e-07	no change
6630	6630	CCL3	1.74672462437495e-11	-1.997109978	0.05	0.064	3.81554526948463e-07	down
6631	6631	ZBTB2	1.75747222571094e-11	-0.151475042	0.071	0.054	3.83902232984297e-07	no change
6632	6632	CHIC1	1.76105633593503e-11	0.152598654919267	0.036	0.024	3.84685146021648e-07	no change
6633	6633	MRPL53	1.79793939787094e-11	-0.113798445	0.091	0.071	3.92741882070927e-07	no change
6634	6634	SMIM7	1.84128826995971e-11	-0.102405596	0.431	0.356	4.02211009689999e-07	no change
6635	6635	RNF103	1.8506897597468e-11	-0.193968465	0.16	0.131	4.0426467111909e-07	no change
6636	6636	CITED2	1.8550806396247e-11	-0.573198192	0.346	0.341	4.05223814919619e-07	no change
6637	6637	MAFG	1.87394614920726e-11	-0.14466484	0.222	0.184	4.09344796832834e-07	no change
6638	6638	IL15RA	1.88458147331572e-11	-0.215551062	0.048	0.034	4.11667977031086e-07	no change
6639	6639	RABGEF1	1.88832334777234e-11	-0.22435594	0.067	0.051	4.12485352087389e-07	no change
6640	6640	C10orf88	1.90629681755688e-1	0.11660881685071	0.031	0.021	4.16411476827125e-0	no

			1	9			7	change
6641	6641	NWD2	1.91585834111951e-11	-0.871287386	0.01	0.017	4.18500096034145e-07	no change
6642	6642	ADAMTSL4	1.92257395400899e-11	-1.414421578	0.027	0.038	4.19967054513723e-07	down
6643	6643	DUSP19	1.92933772084898e-11	0.236084683194299	0.021	0.012	4.2144453174225e-07	no change
6644	6644	RP11-116D17.3	1.93546187299012e-11	0.30477364082217	0.013	0.007	4.22782291535962e-07	no change
6645	6645	ERAP2	1.96268695057802e-11	-0.200930794	0.079	0.06	4.28729337484262e-07	no change
6646	6646	RP13-890H12.2	1.96966296443727e-11	-1.031476507	0.014	0.023	4.30253177951677e-07	down
6647	6647	CEP72	1.9708775237392e-11	0.395665380179687	0.01	0.005	4.30518486285591e-07	no change
6648	6648	NAPA	1.99326646990831e-11	-0.116849686	0.44	0.362	4.35409127686771e-07	no change
6649	6649	CXCR2	2.03405001997335e-11	0.309042438394703	0.018	0.01	4.44317886362978e-07	no change
6650	6650	SSX2IP	2.07429322900801e-11	-0.128145349	0.055	0.04	4.5310861294451e-07	no change
6651	6651	HLA-DMB	2.1113501463139e-11	-0.800550337	0.078	0.094	4.61203325960809e-07	no change
6652	6652	CCDC146	2.13020687644915e-11	0.198684787438357	0.02	0.012	4.65322390091553e-07	no change
6653	6653	KCNK5	2.15901584021652e-1	-0.969620669	0.018	0.028	4.71615420136897e-0	no

			1				7	change
6654	6654	MANEA-AS1	2.16172792719809e-11	0.352735017529265	0.014	0.008	4.7220784841715e-07	no change
6655	6655	TMEM185B	2.1644434563303e-11	-0.146020739	0.081	0.062	4.72801028600791e-07	no change
6656	6656	PRKCD	2.16823440272306e-11	-0.156117777	0.168	0.138	4.73629122930825e-07	no change
6657	6657	TMEM242	2.18994726041272e-11	-0.157595042	0.174	0.142	4.78372079564554e-07	no change
6658	6658	SLC35F5	2.24188541435559e-11	-0.114842854	0.197	0.164	4.89717449911836e-07	no change
6659	6659	PPP2R3B	2.26814837303225e-11	0.192416708225182	0.027	0.018	4.95454330605165e-07	no change
6660	6660	FGR	2.32701061870437e-11	-0.61769829	0.031	0.043	5.08312199549784e-07	no change
6661	6661	RP4-773N10.4	2.39408154710574e-11	0.267074206995373	0.014	0.007	5.22963173149777e-07	no change
6662	6662	RP11-191L17.1	2.39739523011258e-11	-0.803122374	0.024	0.035	5.23687014065793e-07	no change
6663	6663	RP11-884K10.7	2.41073575988272e-11	0.558100530421881	0.01	0.004	5.26601119388781e-07	no change
6664	6664	EIF5AL1	2.42589480384848e-11	-0.2546036	0.105	0.083	5.29912460952663e-07	no change
6665	6665	ZNF304	2.4347137785006e-11	0.140157414022125	0.017	0.01	5.31838877775671e-07	no change
6666	6666	MUCL1	2.45827718383922e-1	1.71643779567675	0.011	0.005	5.3698606803784e-07	up

			1					
6667	6667	IFNLR1	2.46962441394939e-11	0.146708427346939	0.032	0.021	5.39464756983105e-07	no change
6668	6668	MRPL18	2.51474215320083e-11	-0.218994951	0.604	0.494	5.49320275945189e-07	no change
6669	6669	PRTFDC1	2.53741851747728e-11	-0.161028776	0.033	0.022	5.54273700957736e-07	no change
6670	6670	CLCN5	2.54509147490729e-11	0.300633063254693	0.022	0.014	5.55949781778748e-07	no change
6671	6671	TRAM1L1	2.5592338213936e-11	0.498981736538342	0.014	0.007	5.59039035945219e-07	no change
6672	6672	ZNF665	2.57874102676244e-11	0.293096677223071	0.017	0.009	5.63300189885987e-07	no change
6673	6673	FAM149A	2.64772014311804e-11	0.107786111886987	0.023	0.014	5.78367988062705e-07	no change
6674	6674	AC104667.3	2.68981938925395e-11	-0.918088964	0.014	0.023	5.87564147388633e-07	no change
6675	6675	SLC2A3	2.70981566097979e-11	-1.026386335	0.035	0.047	5.91932132984425e-07	down
6676	6676	ADIPOR2	2.72223431265868e-11	-0.127447924	0.117	0.094	5.94644863257163e-07	no change
6677	6677	CTDSP1	2.72393451193088e-11	-0.168794884	0.216	0.179	5.95016254786182e-07	no change
6678	6678	SHISA5	2.72951198309561e-11	-0.326578571	0.424	0.408	5.96234597587405e-07	no change
6679	6679	ZFY-AS1	2.74440618102477e-1	-0.812474607	0.017	0.027	5.99488086183051e-0	no

			1				7	change
6680	6680	SGCG	2.79028808723928e-11	-1.926369506	0.006	0.012	6.09510529776548e-07	down
6681	6681	AAMDC	2.79194069710233e-11	-0.156828389	0.386	0.318	6.09871525875033e-07	no change
6682	6682	HSBP1	2.82368947250109e-11	-0.10910447	0.895	0.815	6.16806728373138e-07	no change
6683	6683	CLN3	2.930069132435e-11	-0.150973867	0.244	0.203	6.400443012891e-07	no change
6684	6684	RP11-626G11.1	2.93192534688552e-11	0.433971678564109	0.012	0.006	6.40449772773674e-07	no change
6685	6685	MFSD11	2.94951863553336e-11	-0.165899061	0.158	0.129	6.44292850745908e-07	no change
6686	6686	ACSL3	3.12983606380346e-11	-0.149360941	0.297	0.248	6.83681389777227e-07	no change
6687	6687	CSTA	3.13886137961814e-11	-0.348920049	0.52	0.491	6.85652879763787e-07	no change
6688	6688	RP5-1085F17.3	3.159018115911e-11	-0.291513671	0.068	0.052	6.90055917239598e-07	no change
6689	6689	FKBP10	3.17644875358066e-11	-1.238100999	0.033	0.045	6.9386346573216e-07	down
6690	6690	DNAJC3	3.23375395547522e-11	-0.178857025	0.411	0.34	7.06381214034008e-07	no change
6691	6691	SHOC2	3.4064832141949e-11	-0.17097468	0.21	0.174	7.44112193308735e-07	no change
6692	6692	HES5	3.44056219393934e-1	0.57863772187023	0.014	0.007	7.51556405644109e-0	no

			1	8			7	change
6693	6693	SGSM1	3.45361002835912e-11	0.335044820705065	0.019	0.011	7.54406574594765e-07	no change
6694	6694	RP11-164P12.4	3.45647630055934e-11	0.372246823796416	0.015	0.008	7.55032683094182e-07	no change
6695	6695	ZNF746	3.47651304356097e-11	0.132470115902589	0.026	0.017	7.59409509235457e-07	no change
6696	6696	ZNF407	3.55047891398195e-11	0.152525262834146	0.021	0.013	7.75566613970218e-07	no change
6697	6697	PABPC3	3.6591186446044e-11	0.241006069313412	0.015	0.008	7.99297876727386e-07	no change
6698	6698	KIAA0556	3.7433383784991e-11	0.145537694685911	0.028	0.018	8.17694835399343e-07	no change
6699	6699	RP13-143G15.4	3.77358132275258e-11	0.299479501423423	0.012	0.006	8.24301104142074e-07	no change
6700	6700	RP11-159G9.5	3.78699922455217e-11	0.133668789222375	0.021	0.013	8.27232110611177e-07	no change
6701	6701	SH3PXD2B	3.88128871816348e-11	-0.285152948	0.042	0.029	8.47828707595631e-07	no change
6702	6702	TACSTD2	3.88914833481407e-11	-0.11537124	0.97	0.905	8.49545562256786e-07	no change
6703	6703	RP11-1017G21.5	3.92254029917282e-11	-0.985782692	0.01	0.018	8.56839702951311e-07	no change
6704	6704	RP11-176H8.1	3.95050329943484e-11	0.178882194713838	0.018	0.01	8.62947940728546e-07	no change
6705	6705	CTD-2336O2.1	3.99770087043763e-1	-0.222116502	0.198	0.162	8.73257778138396e-0	no

			1				7	change
6706	6706	TMEM99	4.29986334054762e-11	-0.140635648	0.337	0.281	9.39262148109222e-07	no change
6707	6707	UPK1A	4.30601042962848e-11	-0.991647822	0.321	0.318	9.40604918248044e-07	no change
6708	6708	CACHD1	4.53846431530819e-11	-0.133325966	0.022	0.014	9.91382145035921e-07	no change
6709	6709	PLEKHA4	4.55408603172365e-11	-0.827886862	0.033	0.045	9.94794552769715e-07	no change
6710	6710	CTA-243E7.4	4.59700428434364e-11	0.131327056433505	0.011	0.005	1.00416961587203e-06	no change
6711	6711	ABL1	4.72652343536897e-11	-0.282888102	0.047	0.034	1.032461779222e-06	no change
6712	6712	MMEL1	4.79116950623066e-11	-0.116810347	0.202	0.169	1.04658306694103e-06	no change
6713	6713	SNAPC5	4.97829860294578e-11	-0.101947322	0.197	0.164	1.08745954682748e-06	no change
6714	6714	RP11-12G12.7	5.00972523525912e-11	-0.174594218	0.281	0.234	1.09432438039e-06	no change
6715	6715	GMFB	5.04763322463253e-11	-0.1837338	0.169	0.139	1.10260500158873e-06	no change
6716	6716	SMPDL3B	5.10697775040761e-11	0.100880563084407	0.032	0.021	1.11556821979904e-06	no change
6717	6717	CTC-523E23.11	5.17277901076837e-11	0.349980964359009	0.01	0.005	1.12994184711224e-06	no change
6718	6718	NXPH4	5.20312147460098e-1	0.70073310622178	0.011	0.006	1.13656985491184e-0	no

				1	4			6	change
6719	6719	AKR1B1		5.20314760418681e-11	-0.538348124	0.397	0.381	1.13657556265857e-06	no change
6720	6720	FKBP1B		5.20986435510799e-11	-0.122420954	0.142	0.115	1.13804276972979e-06	no change
6721	6721	ARL2		5.24696400811437e-11	-0.163000984	0.403	0.336	1.1461468179325e-06	no change
6722	6722	FAM195B		5.27708429513985e-11	-0.462682052	0.309	0.31	1.15272629343035e-06	no change
6723	6723	ADAM33		5.31929952145475e-11	-1.805544573	0.007	0.013	1.16194778746658e-06	down
6724	6724	DCLRE1A		5.44480239851291e-11	0.436563196425223	0.017	0.01	1.18936263593116e-06	no change
6725	6725	CWC25		5.5012047458938e-11	-0.205212658	0.252	0.208	1.20168316469304e-06	no change
6726	6726	ATP10D		5.55448137590248e-11	-0.143312676	0.069	0.052	1.21332091175214e-06	no change
6727	6727		10-Mar	5.57431057853468e-11	0.588016149003294	0.01	0.004	1.21765240277512e-06	no change
6728	6728	DUSP22		5.81900690423386e-11	-0.152027746	0.124	0.101	1.27110386816084e-06	no change
6729	6729	IDUA		5.85726321667124e-11	-0.119332845	0.069	0.053	1.27946057704967e-06	no change
6730	6730	COL4A1		5.85732509813873e-11	-0.85147863	0.023	0.033	1.27947409443742e-06	no change
6731	6731	ALKBH8		5.9944422298471e-11	0.14118792695525	0.018	0.01	1.3094259606878e-06	no

				1				change
6732	6732	AP001059.6	6.06966016695304e-11	-1.084710518	0.006	0.012	1.32585656686922e-06	down
6733	6733	GALC	6.15852111138903e-11	-0.1536401	0.089	0.07	1.34526735157182e-06	no change
6734	6734	SERF1A	6.33657897704562e-11	0.289994307377081	0.013	0.007	1.38416231174584e-06	no change
6735	6735	SOCS1	6.34283307279214e-11	-0.919476155	0.092	0.107	1.38552845642072e-06	no change
6736	6736	ADGRF5	6.40110912194747e-11	-1.034982001	0.018	0.027	1.3982582765982e-06	down
6737	6737	LINC00987	6.46411616140108e-11	-0.838916963	0.037	0.049	1.41202153429645e-06	no change
6738	6738	RP11-802E16.3	6.47557106264079e-11	0.226998585992157	0.014	0.007	1.41452374292325e-06	no change
6739	6739	CAV1	6.51990547309661e-11	-0.925639845	0.087	0.102	1.42420815154322e-06	no change
6740	6740	CD276	6.53205071592328e-11	-0.164982429	0.101	0.081	1.42686115838628e-06	no change
6741	6741	ACOT4	6.5675730973211e-11	0.176394889342614	0.036	0.025	1.43462066737882e-06	no change
6742	6742	SEMA7A	6.6588293172433e-11	-0.651173653	0.058	0.073	1.45455467605863e-06	no change
6743	6743	TCEAL7	6.70289005564743e-11	-1.255432158	0.017	0.026	1.46417930375562e-06	down
6744	6744	PIH1D1	6.76330432928166e-1	-0.133679308	0.369	0.307	1.47737619768829e-0	no

			1				6	change
6745	6745	MATR3	6.79588969369302e-11	0.117284595895285	0.023	0.014	1.4844941446903e-06	no change
6746	6746	ADAMTS13	6.80005840150102e-11	0.510068108168957	0.012	0.006	1.48540475722388e-06	no change
6747	6747	HYLS1	6.82078416068452e-11	0.292916335614233	0.027	0.017	1.48993209205993e-06	no change
6748	6748	PLCH2	6.8835462171114e-11	-0.101854143	0.089	0.07	1.50364183566582e-06	no change
6749	6749	SMAD7	6.90666036413346e-11	-0.232819429	0.081	0.063	1.50869088994131e-06	no change
6750	6750	USP4	6.95128990808863e-11	-0.123305102	0.096	0.076	1.51843976752288e-06	no change
6751	6751	NBL1	7.45429492914407e-11	-0.434251239	0.28	0.229	1.62831618432223e-06	no change
6752	6752	ZNF286A	7.48220217640475e-11	0.115544682694768	0.021	0.013	1.63441224341385e-06	no change
6753	6753	ZNF316	7.6894017488777e-11	-0.230923631	0.074	0.057	1.67967291802484e-06	no change
6754	6754	ANO7	7.75634491391319e-11	0.194834632856909	0.027	0.018	1.6942959829952e-06	no change
6755	6755	NUAK1	7.78089379406297e-11	0.202720396305113	0.023	0.014	1.69965844037512e-06	no change
6756	6756	GPRASP2	7.89021666206745e-11	0.104983529299084	0.023	0.014	1.72353892766201e-06	no change
6757	6757	SDF2L1	7.96069940973571e-1	-0.141033227	0.482	0.403	1.73893517906267e-0	no

			1				6	change
6758	6758	SCAMP5	8.00029282830343e-11	-0.63302697	0.046	0.06	1.7475839654146e-06	no change
6759	6759	PPL	8.00180946686738e-11	-0.127576033	0.15	0.123	1.74791525994251e-06	no change
6760	6760	VGF	8.07365954454293e-11	0.407039277196456	0.016	0.009	1.76361019090996e-06	no change
6761	6761	EMX2OS	8.2309664506048e-11	0.186199699887641	0.047	0.034	1.79797231147011e-06	no change
6762	6762	RAB9A	8.37499219049197e-11	-0.133669422	0.258	0.216	1.82943329409107e-06	no change
6763	6763	APTR	8.42634421116638e-11	-0.167427729	0.079	0.061	1.84065062948718e-06	no change
6764	6764	GIGYF1	8.46687815972306e-11	-0.227216886	0.102	0.081	1.84950486520991e-06	no change
6765	6765	RP11-481J2.4	8.58317555916679e-11	0.485684946635648	0.01	0.004	1.87490886914439e-06	no change
6766	6766	TRANK1	8.58360157264297e-11	0.120750194962388	0.024	0.015	1.87500192752813e-06	no change
6767	6767	AC005253.2	8.66560069458208e-11	0.204131062067776	0.02	0.012	1.89291381572451e-06	no change
6768	6768	LARS2	8.75336948081942e-11	0.198030742932391	0.023	0.014	1.91208602939019e-06	no change
6769	6769	ZSCAN25	8.80153485414805e-11	0.148599036879777	0.02	0.012	1.9226072735401e-06	no change
6770	6770	NEXN	8.88948726023822e-1	-1.424677729	0.018	0.027	1.94181959712644e-0	down

			1				6	
6771	6771	RP5-832C2.5	8.90216181459026e-11	0.181510507381134	0.022	0.014	1.9445882267791e-06	no change
6772	6772	MAP4	8.9091970962975e-11	-0.181390452	0.224	0.186	1.94612501371522e-06	no change
6773	6773	ASL	8.98769853847923e-11	-0.1956279	0.223	0.184	1.9632728687454e-06	no change
6774	6774	CRTC1	9.03057433220464e-11	-0.101695629	0.05	0.037	1.97263865712678e-06	no change
6775	6775	ADAMTSL3	9.08422419109557e-11	-1.749274839	0.009	0.016	1.98435793230292e-06	down
6776	6776	SLX4	9.12438005538087e-11	0.381948356973636	0.013	0.007	1.9931295792974e-06	no change
6777	6777	RNF157	9.29174961989521e-11	0.352802160769414	0.013	0.006	2.02968978696991e-06	no change
6778	6778	CTC-338M12.5	9.52073806968904e-11	-0.131781715	0.037	0.025	2.07971002394287e-06	no change
6779	6779	RANBP9	9.62344339368075e-11	-0.155476066	0.124	0.1	2.10214497491562e-06	no change
6780	6780	COL4A2	9.79592177126659e-11	-0.931895006	0.023	0.034	2.13982115171547e-06	no change
6781	6781	CH507-154B10.1	1.01040286633401e-10	0.172551171156657	0.032	0.022	2.20712402122002e-06	no change
6782	6782	CXCL6	1.0139203121094e-10	-1.219386918	0.037	0.049	2.21480752977178e-06	down
6783	6783	LPIN3	1.02777585592525e-1	-0.170343095	0.133	0.108	2.24507357968312e-0	no

			0				6	change
6784	6784	FASTK	1.02803619888082e-10	-0.132316915	0.335	0.279	2.24564227283525e-06	no change
6785	6785	MAN2B2	1.03295633184075e-10	-0.101717488	0.08	0.063	2.25638981127295e-06	no change
6786	6786	WDR47	1.03480081751153e-10	-0.14401672	0.046	0.033	2.26041890577219e-06	no change
6787	6787	LINC01348	1.05551781990899e-10	-1.078692534	0.008	0.015	2.30567312580919e-06	down
6788	6788	USP40	1.0598886863813e-10	-0.151405439	0.141	0.115	2.31522084653131e-06	no change
6789	6789	LCMT2	1.11243580111576e-10	0.293208325006378	0.019	0.012	2.43000476395727e-06	no change
6790	6790	B3GALT5	1.11780142418287e-10	0.355773883908006	0.014	0.007	2.44172543098506e-06	no change
6791	6791	GAREM2	1.12471944881038e-10	0.515477675329318	0.011	0.005	2.45683716398138e-06	no change
6792	6792	CCNJ	1.12791096415791e-10	0.326056800471343	0.021	0.013	2.46380871010653e-06	no change
6793	6793	HIST1H2BK	1.15507373615305e-10	0.522837203620714	0.011	0.005	2.52314306925271e-06	no change
6794	6794	INA	1.15704411753002e-10	-0.703678234	0.075	0.09	2.52744717033258e-06	no change
6795	6795	NOTCH2NL	1.18795045258142e-10	0.238889383377913	0.022	0.014	2.59495896861885e-06	no change
6796	6796	SLC1A1	1.21579451272971e-1	0.35798296519255	0.012	0.006	2.65578153360679e-0	no

			0				6	change
6797	6797	NFE2L1	1.26488708248876e-10	-0.128186654	0.151	0.125	2.76301934298845e-06	no change
6798	6798	ABHD11	1.2695807933546e-10	-0.566480956	0.362	0.346	2.77327228500379e-06	no change
6799	6799	ZNF232	1.28229222328665e-10	0.24562244623394	0.021	0.013	2.80103913254735e-06	no change
6800	6800	IGHG4	1.3179442264039e-10	-2.712405827	0.122	0.138	2.87891736815668e-06	down
6801	6801	RNF187	1.38562126796377e-10	-0.118711163	0.414	0.343	3.02675109774005e-06	no change
6802	6802	C2CD4B	1.38986186218822e-10	-1.539396613	0.008	0.014	3.03601425176394e-06	down
6803	6803	ZNF319	1.39683429884825e-10	0.415317221963769	0.016	0.009	3.05124484240411e-06	no change
6804	6804	RP11-110G21.1	1.41205888076146e-10	0.161443892714593	0.035	0.024	3.08450141913533e-06	no change
6805	6805	PRDM10	1.41806965443741e-10	0.388018961844239	0.016	0.009	3.09763135315307e-06	no change
6806	6806	RP11-342K6.1	1.41828644107678e-10	0.356755187452642	0.02	0.012	3.09810490188812e-06	no change
6807	6807	PDE6B	1.42908221053872e-10	0.43845230954549	0.013	0.007	3.12168718070078e-06	no change
6808	6808	P4HB	1.43944990321958e-10	0.14495446546508	0.755	0.671	3.14433436859285e-06	no change
6809	6809	KDM6B	1.46312893041705e-1	-0.204170942	0.36	0.299	3.19605883560301e-0	no

			0				6	change
6810	6810	CHCHD7	1.47742688714221e-10	-0.135289786	0.379	0.314	3.22729129227344e-06	no change
6811	6811	CD82	1.50551678943466e-10	-0.257736923	0.256	0.21	3.28865087484108e-06	no change
6812	6812	MRPL38	1.51703235498176e-10	0.198009034574635	0.023	0.015	3.31380547622216e-06	no change
6813	6813	PIP5K1A	1.52545195393692e-10	-0.165871324	0.14	0.115	3.33219724817981e-06	no change
6814	6814	KB-1562D12.1	1.54549141938903e-10	-0.148718237	0.105	0.085	3.37597145651341e-06	no change
6815	6815	PLXDC2	1.57902763144293e-10	-0.388989302	0.114	0.091	3.44922795812394e-06	no change
6816	6816	CBX2	1.5937885777551e-10	0.228425635578988	0.029	0.02	3.48147176924823e-06	no change
6817	6817	GPX8	1.62460227751758e-10	-1.26246152	0.035	0.047	3.54878121500941e-06	down
6818	6818	GNA14	1.71293500330984e-10	0.169931741941257	0.025	0.016	3.74173522123002e-06	no change
6819	6819	PCP4L1	1.75209150489111e-10	-0.140448095	0.348	0.293	3.82726868328413e-06	no change
6820	6820	XYLB	1.77141962676607e-10	0.16265988512477	0.039	0.028	3.8694890327078e-06	no change
6821	6821	ZDHHC7	1.79059208005805e-10	-0.103931753	0.117	0.095	3.9113693396788e-06	no change
6822	6822	PGBD4	1.80120579995717e-1	0.11810442657375	0.024	0.015	3.93455394942645e-0	no

			0				6	change
6823	6823	YY2	1.86203386523741e-10	0.297934506181567	0.015	0.008	4.0674267752246e-06	no change
6824	6824	SLC25A45	1.86295356674854e-10	-0.732308442	0.042	0.054	4.0694357712055e-06	no change
6825	6825	HSPA5	1.93970624746812e-10	-0.132044106	0.575	0.476	4.23709432696937e-06	no change
6826	6826	RP11-147L13.15	1.96023485627305e-10	0.462506241620335	0.016	0.009	4.28193702004285e-06	no change
6827	6827	AARS2	2.03270586431291e-10	0.129280421556201	0.017	0.01	4.44024269000513e-06	no change
6828	6828	EVC2	2.03340835846693e-10	0.218117211464316	0.012	0.006	4.44177721823515e-06	no change
6829	6829	MFSD2A	2.06834974750243e-10	-0.23050409	0.234	0.193	4.51810318844431e-06	no change
6830	6830	KITLG	2.08363336754755e-10	-1.519973254	0.01	0.017	4.55148872807087e-06	down
6831	6831	YY1AP1	2.09196681217874e-10	-0.186206026	0.137	0.112	4.56969230452325e-06	no change
6832	6832	NEURL4	2.10359209406613e-10	0.138437491905982	0.017	0.01	4.59508657027804e-06	no change
6833	6833	PRDM5	2.10755110808395e-10	0.462842510057223	0.011	0.005	4.60373464049858e-06	no change
6834	6834	GTSF1	2.10892025912649e-10	-0.869058037	0.007	0.013	4.6067254140359e-06	no change
6835	6835	SRSF6	2.1442579527748e-10	-0.22964835	0.501	0.415	4.68391707204127e-0	no

							6	change
6836	6836	SENP8	2.17854954886643e-10	0.257435123056331	0.019	0.011	4.75882363454383e-06	no change
6837	6837	ISYNA1	2.2027605634105e-10	-0.117706764	0.321	0.27	4.8117101747139e-06	no change
6838	6838	RNF24	2.20819314757288e-10	-0.165162805	0.134	0.11	4.8235771115582e-06	no change
6839	6839	PINLYP	2.23667366486799e-10	-0.616977467	0.025	0.036	4.88578995353763e-06	no change
6840	6840	SRD5A3	2.27163511527239e-10	-0.220084279	0.263	0.218	4.96215974580101e-06	no change
6841	6841	LSM10	2.30779760636299e-10	-0.130211019	0.347	0.29	5.04115309133931e-06	no change
6842	6842	DLX2	2.37958420411167e-10	0.300564856031122	0.016	0.009	5.19796373546154e-06	no change
6843	6843	ARL9	2.4217178713323e-10	0.442461428940618	0.011	0.005	5.29000051813827e-06	no change
6844	6844	FAM50B	2.42840862749188e-10	-0.161288067	0.045	0.033	5.30461580589326e-06	no change
6845	6845	KAZALD1	2.45306569986709e-10	-1.067259038	0.037	0.048	5.35847671478968e-06	down
6846	6846	RP13-1032I1.7	2.47252263178544e-10	0.110698132741093	0.038	0.027	5.40097843687212e-06	no change
6847	6847	CYP2C18	2.50667669954395e-10	-1.156918835	0.008	0.015	5.47558458248381e-06	down
6848	6848	VCPKMT	2.51401744174792e-1	-0.143378105	0.061	0.046	5.49161969975417e-0	no

			0				6	change
6849	6849	GABARAPL2	2.52145199082692e-10	-0.186168939	0.732	0.652	5.50785972876233e-06	no change
6850	6850	NDRG2	2.55536226457823e-10	-0.51261249	0.272	0.277	5.58193333074468e-06	no change
6851	6851	ZSCAN32	2.58037285546985e-10	0.162558554640357	0.022	0.014	5.63656646548835e-06	no change
6852	6852	PPP1R12C	2.61248349373609e-10	-0.101085806	0.061	0.047	5.70670894371712e-06	no change
6853	6853	TTC30B	2.65830783245729e-10	0.277728730232496	0.02	0.013	5.8068076292197e-06	no change
6854	6854	ABCB9	2.67369081707467e-10	0.178134044293326	0.016	0.009	5.84041022081791e-06	no change
6855	6855	BNC2	2.71083942985431e-10	-1.401256413	0.011	0.018	5.92155765057375e-06	down
6856	6856	RP11-126L15.4	2.75408250060249e-10	0.34844153811702	0.016	0.009	6.01601781431609e-06	no change
6857	6857	MZF1	2.83284014536722e-10	-0.118676843	0.092	0.073	6.18805601354015e-06	no change
6858	6858	ZNF69	2.84637921792486e-10	0.12074785040474	0.031	0.021	6.21763076363507e-06	no change
6859	6859	GPC2	2.94358259953741e-10	0.382172638804291	0.012	0.006	6.42996183042951e-06	no change
6860	6860	GLIPR2	2.95874709910573e-10	-1.167151682	0.026	0.036	6.46308716328656e-06	down
6861	6861	FNDC4	2.97665215129845e-1	-0.747441645	0.04	0.053	6.50219895929634e-0	no

			0				6	change
6862	6862	MPZ	2.99304915689704e-10	0.367658506248913	0.018	0.01	6.53801657832589e-06	no change
6863	6863	GNG10	3.00904572818082e-10	0.117264160016158	0.026	0.017	6.57295948863818e-06	no change
6864	6864	COL5A1	3.06234463356773e-10	-1.269991832	0.012	0.02	6.68938561756536e-06	down
6865	6865	TRPS1	3.07832318051365e-10	-0.190584502	0.023	0.015	6.72428915551402e-06	no change
6866	6866	ZNF394	3.09729866856872e-10	-0.21335062	0.184	0.152	6.76573921162151e-06	no change
6867	6867	HERC3	3.098448400114e-10	-0.167239326	0.088	0.07	6.76825068520903e-06	no change
6868	6868	C11orf74	3.12038171808632e-10	-0.211438827	0.135	0.11	6.81616182498776e-06	no change
6869	6869	RP11-663N22.1	3.15684657129558e-10	-0.935226218	0.01	0.017	6.89581565033807e-06	no change
6870	6870	TRIM45	3.25136870383225e-10	0.182671079729157	0.023	0.015	7.10228979665116e-06	no change
6871	6871	VEPH1	3.2547247761606e-10	-1.398062984	0.005	0.01	7.10962080104521e-06	down
6872	6872	PIEZO2	3.34656915537564e-10	-1.533350182	0.008	0.014	7.31024566300255e-06	down
6873	6873	IFI44L	3.47050841513027e-10	-0.117798075	0.214	0.182	7.58097858201056e-06	no change
6874	6874	ZKSCAN7	3.49511303698713e-1	0.40629812015623	0.011	0.005	7.63472491799469e-0	no

			0	3			6	change
6875	6875	MATN2	3.54863209711347e-10	-0.589965099	0.14	0.113	7.75163195293467e-06	no change
6876	6876	LINC01285	3.60789296672771e-10	-0.899389182	0.018	0.027	7.88108139652001e-06	no change
6877	6877	RASSF8	3.61969648358088e-10	-0.260856088	0.033	0.023	7.90686499873409e-06	no change
6878	6878	SCAANT1	3.66515516884832e-10	-0.933104177	0.011	0.018	8.00616495083227e-06	no change
6879	6879	THAP9-AS1	3.67890314690136e-10	-0.210231728	0.175	0.145	8.03619603409132e-06	no change
6880	6880	SEMA3C	3.68265594106551e-10	-0.41455931	0.25	0.206	8.04439363766349e-06	no change
6881	6881	RP11-458F8.4	3.70286057585726e-10	0.106493206717822	0.019	0.012	8.08852864190261e-06	no change
6882	6882	ITGB1BP1	3.72976789974009e-10	-0.155262451	0.382	0.316	8.14730500019226e-06	no change
6883	6883	HINT3	3.74397693321718e-10	-0.147894207	0.149	0.123	8.1783432129196e-06	no change
6884	6884	RP11-493L12.4	3.75967060417281e-10	0.515775047371489	0.01	0.005	8.21262446775508e-06	no change
6885	6885	MOGAT2	3.82377520638946e-10	0.228047135263826	0.011	0.005	8.35265456083714e-06	no change
6886	6886	GCNT3	3.83063257039976e-10	-0.799057384	0.023	0.033	8.36763378678123e-06	no change
6887	6887	ZNF700	3.85615554437054e-1	0.14299661963726	0.026	0.017	8.42338617112301e-0	no

			0	2			6	change
6888	6888	CCDC159	3.88471601599902e-10	-0.192356163	0.104	0.083	8.48577366534827e-06	no change
6889	6889	NXPE3	4.01005290669273e-10	0.118628396773521	0.024	0.015	8.7595595693796e-06	no change
6890	6890	HOXC13	4.02241696475771e-10	0.38835024882783	0.016	0.009	8.78656761781675e-06	no change
6891	6891	IPPK	4.121780701603e-10	0.127950504782644	0.025	0.016	9.00361776458159e-06	no change
6892	6892	AP000253.1	4.15599372821903e-10	0.424109479415516	0.013	0.007	9.07835269992166e-06	no change
6893	6893	TRMT10A	4.18704578403869e-10	-0.105076563	0.076	0.06	9.1461828106541e-06	no change
6894	6894	STK17A	4.19358050022039e-10	-0.18686322	0.233	0.195	9.16045724468143e-06	no change
6895	6895	FCER1G	4.328254272313e-10	-1.108627882	0.048	0.061	9.45463863244051e-06	down
6896	6896	FAM131A	4.3358867227145e-10	-0.165016198	0.069	0.054	9.47131095709755e-06	no change
6897	6897	BORCS8	4.41235887394124e-10	-0.152333503	0.169	0.141	9.63835672423724e-06	no change
6898	6898	SDCCAG8	4.46637113828793e-10	-0.153132113	0.231	0.192	9.75634111447615e-06	no change
6899	6899	ZBTB34	4.47849391342615e-10	0.123901608888226	0.015	0.009	9.78282210448808e-06	no change
6900	6900	DHX38	4.52613747389455e-1	-0.124362593	0.077	0.06	9.88689469797525e-0	no

			0				6	change
6901	6901	ZNF318	4.56645160526575e-10	0.107122676078585	0.04	0.029	9.97495688654251e-06	no change
6902	6902	SEC23A	4.70778983556459e-10	-0.269302711	0.189	0.156	1.02836961168073e-05	no change
6903	6903	SPON1	4.73293004390097e-10	-1.261099178	0.035	0.047	1.03386123878973e-05	down
6904	6904	MOB3A	4.73590260822905e-10	-0.153942451	0.062	0.047	1.03451056574155e-05	no change
6905	6905	UNC13B	4.7527831207911e-10	0.109739848632829	0.023	0.015	1.03819794490561e-05	no change
6906	6906	GPR85	4.89788335592379e-10	0.112850587806876	0.015	0.009	1.06989364026799e-05	no change
6907	6907	VSIG4	4.92820371508913e-10	-1.721541096	0.005	0.011	1.07651681952407e-05	down
6908	6908	EIF5A	4.94519417057124e-10	-0.273083645	0.646	0.532	1.08022821461958e-05	no change
6909	6909	SBK1	5.04409418404205e-10	0.62687991077095	0.012	0.006	1.10183193356215e-05	no change
6910	6910	DYNC1I1	5.09188406082205e-10	0.280716629891326	0.014	0.008	1.11227115424597e-05	no change
6911	6911	MOB3B	5.11703303396527e-10	-0.191248552	0.036	0.025	1.11776469593937e-05	no change
6912	6912	UFM1	5.26397068376677e-10	-0.124736047	0.478	0.397	1.14986175616201e-05	no change
6913	6913	SMN2	5.30670481201946e-1	0.2962194712617	0.015	0.009	1.15919659913753e-0	no

			0				5	change
6914	6914	MEIS2	5.31399571741025e-10	-0.161504695	0.146	0.121	1.1607892245111e-05	no change
6915	6915	SPAG16	5.41663562258744e-10	-0.130003749	0.41	0.34	1.183209885398e-05	no change
6916	6916	KDSR	5.48781139539846e-10	-0.232819912	0.248	0.206	1.19875752121084e-05	no change
6917	6917	C19orf45	5.591648902763e-10	0.220063876212212	0.018	0.011	1.22143978631955e-05	no change
6918	6918	ICAM2	5.64813667310029e-10	-0.5984531	0.119	0.134	1.23377897487203e-05	no change
6919	6919	TFR2	5.69552690305478e-10	0.469656637460393	0.014	0.008	1.24413089670329e-05	no change
6920	6920	CEP85L	5.73342023574646e-10	-0.127014419	0.056	0.042	1.25240831629646e-05	no change
6921	6921	MTMR6	5.78951958760842e-10	-0.222693784	0.076	0.059	1.26466265871718e-05	no change
6922	6922	SLC24A1	5.87865957162998e-10	0.103541703735918	0.019	0.012	1.28413439682685e-05	no change
6923	6923	LIPT2	5.88821562165827e-10	0.163119077496894	0.027	0.018	1.28622182039503e-05	no change
6924	6924	BRPF1	5.93013037288909e-10	0.140492271382103	0.031	0.021	1.29537767865389e-05	no change
6925	6925	ARHGAP25	5.99943959715813e-10	0.133154573022585	0.014	0.008	1.31051758560322e-05	no change
6926	6926	AC068831.10	6.00025273576774e-1	0.34745854984283	0.013	0.007	1.3106952076011e-05	no

			0	8				change
6927	6927	ZNF44	6.00292225420546e-10	-0.147001198	0.118	0.096	1.31127833720864e-05	no change
6928	6928	MEPCE	6.04014851932188e-10	-0.247922569	0.108	0.087	1.31941004256067e-05	no change
6929	6929	SH3RF2	6.04246404591487e-10	0.170825440793963	0.018	0.011	1.31991584618964e-05	no change
6930	6930	FANCE	6.1289495575186e-10	0.27456466962143	0.019	0.012	1.33880774134436e-05	no change
6931	6931	IL1R1	6.16678727668625e-10	-0.689843964	0.095	0.111	1.34707301271934e-05	no change
6932	6932	ARL15	6.2036015184097e-10	-0.158731679	0.062	0.047	1.35511471568141e-05	no change
6933	6933	RAG1	6.30951219215655e-10	0.200313070718021	0.011	0.006	1.37824984325468e-05	no change
6934	6934	ZBED6CL	6.3788142586403e-10	-1.013383418	0.014	0.022	1.39338818665739e-05	down
6935	6935	TMSB15B	6.44066538137613e-10	0.491654992550914	0.011	0.006	1.4068989459078e-05	no change
6936	6936	DAGLB	6.49317830529626e-10	-0.108994892	0.045	0.033	1.41836986900891e-05	no change
6937	6937	PPP5D1	6.51457144429038e-10	0.130954503727844	0.017	0.01	1.42304298629079e-05	no change
6938	6938	MYC	6.61713762716995e-10	-0.660231788	0.391	0.378	1.445447543279e-05	no change
6939	6939	CTD-3010D24.3	6.63524550195937e-1	0.42039664113740	0.012	0.006	1.449403027448e-05	no

			0	9				change
6940	6940	TFAP2C	6.86563651006784e-10	-0.331692117	0.15	0.123	1.49972963925922e-05	no change
6941	6941	MAPKAPK2	6.88601204379877e-10	-0.118904742	0.16	0.133	1.5041804708474e-05	no change
6942	6942	GNAI2	6.9331061503723e-10	-0.116269618	0.288	0.244	1.51446770748733e-05	no change
6943	6943	GBA	6.99462424329538e-10	-0.127638679	0.134	0.111	1.52790571970544e-05	no change
6944	6944	SDCBP2	7.08026897023159e-10	-0.329612071	0.333	0.339	1.54661395385739e-05	no change
6945	6945	FAM174B	7.25921284815217e-10	-0.147183315	0.321	0.267	1.58570245455036e-05	no change
6946	6946	YIPF1	7.36090259971138e-10	-0.10067023	0.143	0.118	1.60791556388095e-05	no change
6947	6947	RP11-97C16.1	7.51639125775317e-10	0.352744768371934	0.012	0.006	1.6418805063436e-05	no change
6948	6948	CAPN13	7.68280114520513e-10	0.189642416739534	0.026	0.017	1.67823108215861e-05	no change
6949	6949	STARD3	7.73153432730025e-10	-0.130547671	0.187	0.156	1.68887635845547e-05	no change
6950	6950	ZNF655	7.7943010138382e-10	-0.436491358	0.267	0.272	1.70258711346282e-05	no change
6951	6951	LINC01116	8.12011741520065e-10	-0.114255598	0.07	0.054	1.77375844817643e-05	no change
6952	6952	SEC11C	8.12567883056838e-1	-0.180638184	0.328	0.273	1.77497328374936e-0	no

			0				5	change
6953	6953	EMILIN1	8.32342138284882e-10	-1.286341177	0.014	0.021	1.8181681668695e-05	down
6954	6954	ZNF556	8.3370610481903e-10	0.18450644375562	0.018	0.011	1.82114761536669e-05	no change
6955	6955	CTA-384D8.35	8.34616949979814e-10	-1.141953736	0.005	0.01	1.82313726553591e-05	down
6956	6956	ITGB7	8.39978873029186e-10	0.169533885360739	0.022	0.014	1.83484985024495e-05	no change
6957	6957	UPRT	8.51498345482261e-10	-0.109674082	0.047	0.035	1.86001298587145e-05	no change
6958	6958	ACSM6	8.52261834422543e-10	0.270822498969734	0.022	0.014	1.8616807511126e-05	no change
6959	6959	KIF1C	8.58656832379966e-10	-0.105925531	0.12	0.099	1.8756499846508e-05	no change
6960	6960	KLHL21	8.58817914431202e-10	-0.199254675	0.091	0.073	1.87600185228352e-05	no change
6961	6961	FAM49B	8.65878853942625e-10	-0.11941638	0.403	0.339	1.89142576855227e-05	no change
6962	6962	STXBP6	8.70144329126246e-10	-1.456057648	0.013	0.02	1.90074327254337e-05	down
6963	6963	SH3BP2	8.82887631286295e-10	-0.20222715	0.128	0.105	1.92857974178178e-05	no change
6964	6964	DHX30	8.91172933899315e-10	-0.125962555	0.24	0.203	1.94667815680966e-05	no change
6965	6965	IQGAP3	8.97020017610296e-1	-0.168052185	0.05	0.037	1.95945052646793e-0	no

			0				5	change
6966	6966	C1QTNF1	9.00615563322407e-10	-1.310442278	0.006	0.012	1.96730463652147e-05	down
6967	6967	RP1-30M3.5	9.10027505326785e-10	0.360434841979798	0.013	0.007	1.98786408263583e-05	no change
6968	6968	DHRSX	9.11090824157758e-10	-0.123929184	0.147	0.122	1.99018679629021e-05	no change
6969	6969	TTC6	9.1376502480545e-10	0.317872212205375	0.018	0.011	1.99602832018503e-05	no change
6970	6970	LIPG	9.3124278451009e-10	-0.679623165	0.044	0.057	2.03420673848384e-05	no change
6971	6971	H1FX-AS1	9.5077033807224e-10	0.34253122977471	0.014	0.007	2.076862726485e-05	no change
6972	6972	MICALL2	9.66373057647479e-10	-0.622497037	0.139	0.154	2.11094530712515e-05	no change
6973	6973	TAP1	9.73221565196326e-10	-0.122143127	0.236	0.2	2.12590518701485e-05	no change
6974	6974	SPRR2A	9.80801151649875e-10	0.602750504428668	0.011	0.006	2.14246203566399e-05	no change
6975	6975	CTA-292E10.6	9.84945034414762e-10	-0.105710917	0.067	0.052	2.15151393317561e-05	no change
6976	6976	RP11-347C12.10	9.86347256965387e-10	-0.105283183	0.047	0.035	2.15457694811519e-05	no change
6977	6977	LENG1	9.89593021253468e-10	-0.190308495	0.131	0.107	2.16166699562608e-05	no change
6978	6978	ATP6V1C1	9.95978049309226e-1	-0.19481066	0.21	0.175	2.17561445091107e-0	no

			0				5	change
6979	6979	MEIG1	1.00286427807312e-09	0.261306263193367	0.01	0.005	2.19065672902293e-05	no change
6980	6980	C14orf105	1.04383336507079e-09	-0.874145732	0.028	0.038	2.28014960266064e-05	no change
6981	6981	ANKRD20A4	1.0484045666824e-09	0.319634414682469	0.012	0.007	2.29013493546105e-05	no change
6982	6982	FAAP100	1.05905180909727e-09	-0.116253968	0.062	0.047	2.31339277179209e-05	no change
6983	6983	EML3	1.0661250376953e-09	-0.220469686	0.155	0.128	2.32884353234161e-05	no change
6984	6984	CCK	1.06866050610399e-09	0.118278074086536	0.044	0.032	2.33438200953357e-05	no change
6985	6985	DDX11-AS1	1.07125299658503e-09	0.300798146664879	0.018	0.011	2.34004504574034e-05	no change
6986	6986	UBAP1	1.08262031529989e-09	-0.194826649	0.128	0.105	2.36487581674108e-05	no change
6987	6987	ABCA1	1.08299613254202e-09	-0.369124626	0.102	0.082	2.36569675192479e-05	no change
6988	6988	RP11-894P9.1	1.11106516114607e-09	0.193995944510939	0.02	0.013	2.42701073800748e-05	no change
6989	6989	ADGRL1	1.13985713649539e-09	0.108326441864267	0.015	0.009	2.48990392896052e-05	no change
6990	6990	ASAH2B	1.18114871625882e-09	0.152988759988868	0.021	0.014	2.58010125579577e-05	no change
6991	6991	TCTE3	1.19018224777195e-0	0.18194702662659	0.033	0.023	2.59983410203305e-0	no

				9	5			5	change
6992	6992	FXYD4		1.23583560660267e-09	-0.707876797	0.113	0.131	2.69955929906287e-05	no change
6993	6993	CSRP2		1.24465612504107e-09	-0.233877646	0.208	0.173	2.71882683953971e-05	no change
6994	6994		9-Mar	1.25885995294142e-09	-0.2293164	0.109	0.088	2.74985368120523e-05	no change
6995	6995	CARD9		1.27493455501714e-09	0.19096680799763	0.035	0.025	2.78496704197943e-05	no change
6996	6996	GDPD2		1.29999698425257e-09	0.216431364581496	0.027	0.018	2.8397134124013e-05	no change
6997	6997	RAB36		1.310593968954e-09	0.23837951693405	0.017	0.01	2.86286146578311e-05	no change
6998	6998	INPP5A		1.37684830900802e-09	-0.112601989	0.054	0.041	3.00758744619712e-05	no change
6999	6999	GNAL		1.3794967034142e-09	0.208725001066268	0.011	0.006	3.01337259893797e-05	no change
7000	7000	RP11-354P11.3		1.41125907322679e-09	0.212295966759673	0.017	0.01	3.0827543195566e-05	no change
7001	7001	NCF1		1.41701917804016e-09	-0.247048303	0.038	0.027	3.09533669251094e-05	no change
7002	7002	MPRIP-AS1		1.42952401737443e-09	-0.150733924	0.099	0.08	3.1226522635527e-05	no change
7003	7003	EIF4EBP3		1.44283744512255e-09	0.103337683817935	0.014	0.008	3.1517341151257e-05	no change
7004	7004	DTX2		1.44520833713212e-0	-0.169449703	0.098	0.079	3.1569130916314e-05	no

			9					change
7005	7005	ZNF749	1.46075167358552e-09	0.202934614936077	0.012	0.007	3.19086595578022e-05	no change
7006	7006	LMNA	1.46756484181572e-09	-0.123771215	0.919	0.888	3.20574864046226e-05	no change
7007	7007	CABLES2	1.47096345631338e-09	0.155290551984921	0.015	0.009	3.21317257397094e-05	no change
7008	7008	PLEKHG5	1.48354227328901e-09	0.117039933479138	0.03	0.02	3.24064974177251e-05	no change
7009	7009	LINC00926	1.49650120694866e-09	-0.904142342	0.027	0.037	3.26895723645866e-05	no change
7010	7010	SAP18	1.5033359489504e-09	-0.131946292	0.867	0.788	3.28388704688726e-05	no change
7011	7011	TMEM52B	1.50869184997089e-09	0.629821228993591	0.01	0.005	3.29558647707642e-05	no change
7012	7012	BAIAP2-AS1	1.51358559573618e-09	0.136658711517794	0.026	0.017	3.30627637532611e-05	no change
7013	7013	ARNT2	1.52109322197421e-09	0.311620144050903	0.019	0.011	3.32267603408047e-05	no change
7014	7014	OSR2	1.53003462198956e-09	-1.074493831	0.023	0.032	3.342207628274e-05	down
7015	7015	MMP10	1.54036772860403e-09	0.594116312103358	0.026	0.017	3.36477926636265e-05	no change
7016	7016	RNF207	1.54221456021528e-09	-0.139212227	0.124	0.102	3.36881348533427e-05	no change
7017	7017	AC159540.1	1.54538608553132e-0	0.31444450988761	0.01	0.005	3.37574136523463e-0	no

			9				5	change
7018	7018	ZNF697	1.55249373105545e-09	0.13756556017074	0.015	0.009	3.39126730611752e-05	no change
7019	7019	TCFL5	1.55744743365237e-09	0.107685883311643	0.021	0.013	3.40208817407023e-05	no change
7020	7020	KAZN	1.55854962456333e-09	-0.167497726	0.068	0.053	3.40449579989614e-05	no change
7021	7021	MYLIP	1.565546779284e-09	-0.199982102	0.332	0.281	3.41978038466797e-05	no change
7022	7022	ARHGEF35	1.5732062653069e-09	-0.175496981	0.202	0.17	3.43651176593639e-05	no change
7023	7023	NISCH	1.59825326000123e-09	-0.202026894	0.11	0.089	3.49122442114668e-05	no change
7024	7024	IQCC	1.61268462211897e-09	0.258892448100246	0.012	0.007	3.52274828855667e-05	no change
7025	7025	TLDC1	1.63084170579815e-09	-0.117969102	0.12	0.098	3.56241062214548e-05	no change
7026	7026	CIDEA	1.63581962841708e-09	0.535241766347197	0.014	0.008	3.57328439631426e-05	no change
7027	7027	IGLC2	1.642503410606e-09	-3.472698408	0.144	0.157	3.58788445012775e-05	down
7028	7028	FCGR2A	1.65043041434855e-09	-1.34472401	0.016	0.024	3.60520019710298e-05	down
7029	7029	CTSS	1.73252899834527e-09	-0.243882637	0.392	0.324	3.78453634398541e-05	no change
7030	7030	TMEM125	1.75600449454785e-0	-0.598386322	0.073	0.088	3.83581621789032e-0	no

			9				5	change
7031	7031	PGF	1.76368293973511e-09	-0.532154682	0.085	0.101	3.85258901355738e-05	no change
7032	7032	AC004381.6	1.79743708958198e-09	0.159263305849358	0.015	0.008	3.92632157848288e-05	no change
7033	7033	NECAB3	1.80726083261896e-09	-0.112595656	0.102	0.083	3.94778056277285e-05	no change
7034	7034	XRCC3	1.8247412797557e-09	0.173284471393758	0.017	0.01	3.98596485149836e-05	no change
7035	7035	NAT8	1.88077235163596e-09	-1.011781693	0.009	0.016	4.10835912491358e-05	down
7036	7036	KLRB1	1.9137978050739e-09	0.161968735618278	0.03	0.02	4.18049992540344e-05	no change
7037	7037	PYROXD2	1.97479921689928e-09	-0.109821842	0.058	0.044	4.31375140939479e-05	no change
7038	7038	DEGS2	1.9777277774985e-09	-0.188521962	0.221	0.185	4.32014855716773e-05	no change
7039	7039	BAHCC1	1.98107261870027e-09	0.259357544301164	0.013	0.007	4.32745502828887e-05	no change
7040	7040	EFR3A	2.00259314127317e-09	-0.185663604	0.134	0.11	4.37446445779711e-05	no change
7041	7041	FAM183A	2.00532503786564e-09	-0.105551763	0.054	0.041	4.3804320127137e-05	no change
7042	7042	PAM16	2.0208384781587e-09	0.302322166991801	0.021	0.013	4.41431957168987e-05	no change
7043	7043	RP11-135A1.2	2.02292027185704e-0	-0.936528168	0.011	0.019	4.41886704184451e-0	no

			9				5	change
7044	7044	TSC22D2	2.04593252421722e-09	-0.486747965	0.29	0.292	4.46913500590009e-05	no change
7045	7045	ANKRD12	2.06791770051118e-09	-0.160321467	0.45	0.37	4.51715942499662e-05	no change
7046	7046	USP21	2.07122136256371e-09	0.158720906601291	0.034	0.025	4.52437594438416e-05	no change
7047	7047	CHPF2	2.09549118117876e-09	-0.135326582	0.104	0.085	4.57739093616689e-05	no change
7048	7048	SLC38A11	2.12319792195616e-09	-0.988221717	0.019	0.028	4.63791354072103e-05	no change
7049	7049	AP001059.5	2.18035471529153e-09	-0.959051908	0.005	0.01	4.76276684008281e-05	no change
7050	7050	ZNF45	2.18081345998954e-09	0.273604218893293	0.016	0.009	4.76376892200115e-05	no change
7051	7051	RP3-395M20.8	2.183747739107e-09	-0.696437852	0.049	0.061	4.77017856130534e-05	no change
7052	7052	ZNF587B	2.21081197358713e-09	0.244577253744495	0.012	0.007	4.82929767510374e-05	no change
7053	7053	GLS	2.22449325006058e-09	-0.155847042	0.211	0.178	4.85918305543232e-05	no change
7054	7054	ASB1	2.23239224037349e-09	-0.148893045	0.083	0.066	4.87643760987186e-05	no change
7055	7055	USF1	2.23693332234336e-09	0.218006192386227	0.02	0.013	4.88635714932684e-05	no change
7056	7056	MAP3K10	2.24802666754377e-0	0.19429036087582	0.02	0.013	4.91058945258261e-0	no

			9	4			5	change
7057	7057	GSE1	2.27144374143981e-09	-0.203858947	0.08	0.064	4.96174170880112e-05	no change
7058	7058	POMT2	2.30830431683401e-09	0.131107134117228	0.023	0.015	5.0422599496922e-05	no change
7059	7059	FAM173A	2.34000921488407e-09	-0.170028419	0.317	0.266	5.11151612899277e-05	no change
7060	7060	ZNF256	2.37857136650081e-09	0.386337175123963	0.011	0.006	5.19575129298437e-05	no change
7061	7061	CSF2RB	2.37877594308375e-09	-1.097956925	0.006	0.011	5.19619817007215e-05	down
7062	7062	CBLB	2.38436143424717e-09	-0.299254604	0.08	0.063	5.20839911696952e-05	no change
7063	7063	CTC-480C2.1	2.44734963058892e-09	0.122295959532141	0.012	0.006	5.34599053305843e-05	no change
7064	7064	ANXA6	2.45544296417319e-09	-1.10503789	0.022	0.031	5.36366961093991e-05	down
7065	7065	MICB	2.50466147309832e-09	-1.04723037	0.014	0.022	5.47118252183597e-05	down
7066	7066	CNEP1R1	2.5134754020984e-09	-0.166794793	0.057	0.044	5.49043566834374e-05	no change
7067	7067	MIEN1	2.5715503980528e-09	-0.24647645	0.661	0.577	5.61729468950655e-05	no change
7068	7068	RP11-452L6.1	2.60082536894767e-09	0.116669197975406	0.014	0.008	5.68124293592929e-05	no change
7069	7069	SLC16A5	2.61863652792918e-0	-0.60469364	0.093	0.108	5.72014963160851e-0	no

			9				5	change
7070	7070	PERM1	2.63887890399026e-09	0.213601570332735	0.026	0.018	5.76436707787632e-05	no change
7071	7071	ROGDI	2.64404052385637e-09	-0.135243842	0.189	0.159	5.77564212031186e-05	no change
7072	7072	MFSD7	2.64457949243479e-09	-0.810690695	0.02	0.029	5.77681944327456e-05	no change
7073	7073	SAMD12	2.64643464115448e-09	-0.273944993	0.134	0.11	5.78087183013785e-05	no change
7074	7074	ZNF385C	2.70060367235512e-09	-1.315007096	0.007	0.013	5.89919866189251e-05	down
7075	7075	WDR78	2.77869806529946e-09	0.252301411032083	0.011	0.006	6.06978805384015e-05	no change
7076	7076	BAIAP2	2.78427087369441e-09	-0.48894093	0.335	0.33	6.08196129649807e-05	no change
7077	7077	ZNF14	2.82267618261234e-09	0.169619214993238	0.017	0.01	6.16585385329839e-05	no change
7078	7078	SLC7A7	2.82460311270122e-09	-1.214090308	0.008	0.014	6.17006303938454e-05	down
7079	7079	SNX21	2.86435730161551e-09	-0.70054252	0.076	0.09	6.25690208964893e-05	no change
7080	7080	RP11-468E2.5	2.90400839202864e-09	0.12740875736224	0.014	0.008	6.34351593154736e-05	no change
7081	7081	UFSP1	2.92007448671989e-09	0.380631339906728	0.013	0.007	6.37861070879092e-05	no change
7082	7082	JUP	2.99772261987803e-0	-0.287552927	0.604	0.536	6.54822529086157e-0	no

			9				5	change
7083	7083	CHDH	3.01226527817795e-09	0.21907402794506	0.031	0.022	6.57999227365192e-05	no change
7084	7084	CEP19	3.07018527162496e-09	0.177918654578928	0.016	0.01	6.70651270733755e-05	no change
7085	7085	ISCA2	3.08740079730567e-09	-0.139902638	0.251	0.212	6.7441183016345e-05	no change
7086	7086	ARL1	3.14719497607266e-09	-0.11366061	0.444	0.37	6.87473270573311e-05	no change
7087	7087	POU2F2	3.31629928144769e-09	0.124227276023858	0.019	0.012	7.24412415039434e-05	no change
7088	7088	DIXDC1	3.36120786646995e-09	-1.247026648	0.024	0.033	7.34222246351697e-05	down
7089	7089	ZNF724P	3.38569531918126e-09	0.243037603387202	0.013	0.007	7.39571285521955e-05	no change
7090	7090	RP11-1275H24.1	3.42198592255959e-09	-0.118684195	0.054	0.041	7.47498604923917e-05	no change
7091	7091	PCDH9	3.42457445652975e-09	-1.47802124	0.012	0.019	7.48064044284359e-05	down
7092	7092	ADCY1	3.4639895311913e-09	0.658783607048223	0.01	0.005	7.56673873193428e-05	no change
7093	7093	AP000704.5	3.46936599199891e-09	0.190458454528724	0.021	0.013	7.57848307292242e-05	no change
7094	7094	TMCC1-AS1	3.58488134575961e-09	0.223013466651628	0.013	0.007	7.83081481167729e-05	no change
7095	7095	CFAP69	3.63787480591397e-0	0.11510623232241	0.014	0.008	7.94657372603847e-0	no

			9	6			5	change
7096	7096	SETBP1	3.66521654547333e-09	-0.331919764	0.029	0.02	8.00629902193195e-05	no change
7097	7097	CCZ1B	3.69754994947605e-09	-0.181541703	0.131	0.108	8.07692810963549e-05	no change
7098	7098	IRF6	3.71928659705208e-09	-0.135701098	0.451	0.375	8.12440964260056e-05	no change
7099	7099	ASB15	3.74382538157886e-09	0.278597645714043	0.01	0.005	8.17801216352086e-05	no change
7100	7100	CALM1	3.74478358982614e-09	-0.111769089	0.883	0.802	8.18010527361622e-05	no change
7101	7101	KCTD21-AS1	3.74930838991856e-09	0.41743066123997	0.012	0.006	8.18998924693809e-05	no change
7102	7102	CTC-559E9.1	3.75819388807707e-09	0.15946689253669	0.018	0.011	8.20939872911555e-05	no change
7103	7103	PLA2G16	3.83073516296425e-09	-0.444190104	0.333	0.275	8.36785788997911e-05	no change
7104	7104	RP11-218M22.1	3.85273012058406e-09	0.175995073858355	0.033	0.023	8.41590367540382e-05	no change
7105	7105	TTC21A	3.86858056479838e-09	0.125893589230144	0.022	0.014	8.45052738574558e-05	no change
7106	7106	GPSM3	3.89053121837473e-09	-0.104520029	0.196	0.166	8.49847639341776e-05	no change
7107	7107	EIF2AK3	3.90632704516374e-09	-0.214435526	0.087	0.07	8.53298079745567e-05	no change
7108	7108	ZFP37	3.91628868284402e-0	0.34751308808747	0.01	0.005	8.55474099880447e-0	no

			9				5	change
7109	7109	LINC00294	3.94941537443522e-09	0.222437423124544	0.017	0.01	8.6271029439163e-05	no change
7110	7110	ATP6V1E1	3.95878250324394e-09	-0.116192377	0.458	0.381	8.64756450008605e-05	no change
7111	7111	PROCR	4.00600019103517e-09	-0.681828156	0.154	0.166	8.75070681729723e-05	no change
7112	7112	SGCD	4.08220043302157e-09	-1.455090795	0.008	0.014	8.91715862589231e-05	down
7113	7113	SP110	4.08900099948059e-09	-0.187631379	0.063	0.049	8.93201378326539e-05	no change
7114	7114	TCTA	4.12715242201494e-09	-0.103983132	0.096	0.078	9.01535175064944e-05	no change
7115	7115	C11orf96	4.13957544675943e-09	-0.541193386	0.239	0.249	9.0424886059013e-05	no change
7116	7116	RAB11FIP1	4.18238213888895e-09	-0.289013911	0.501	0.475	9.13599554418902e-05	no change
7117	7117	COX17	4.27838995182657e-09	-0.158151582	0.725	0.663	9.34571501076996e-05	no change
7118	7118	RP11-89K21.1	4.28058737404738e-09	0.253132969830884	0.064	0.051	9.3505150598691e-05	no change
7119	7119	RP11-513M16.8	4.3775280366611e-09	0.299788016852018	0.012	0.007	9.5622722432825e-05	no change
7120	7120	SLC25A33	4.40183605931991e-09	-0.185518763	0.281	0.236	9.61537068797842e-05	no change
7121	7121	FAM3C	4.40300249903054e-0	-0.319922844	0.508	0.468	9.61791865888231e-0	no

			9				5	change
7122	7122	ACBD3	4.43357002004823e-09	-0.226156134	0.252	0.213	9.68469035179335e-05	no change
7123	7123	ARHGAP40	4.43621796037039e-09	-0.797086827	0.021	0.03	9.69047451263308e-05	no change
7124	7124	MRGPRF	4.45997977590187e-09	-1.534881158	0.009	0.015	9.74237982248005e-05	down
7125	7125	UBAC2	4.48799076210884e-09	-0.149691481	0.272	0.229	9.80356702075055e-05	no change
7126	7126	ZNF83	4.49271853525022e-09	-0.147403124	0.154	0.128	9.81389436840059e-05	no change
7127	7127	RP11-400K9.4	4.49978979985396e-09	0.154990462146722	0.022	0.014	9.82934083880099e-05	no change
7128	7128	ABHD13	4.51607343467095e-09	-0.178648574	0.171	0.143	9.86491081069523e-05	no change
7129	7129	CDADC1	4.5160736691662e-09	-0.150912277	0.06	0.046	9.86491132292665e-05	no change
7130	7130	RAI2	4.52704119648919e-09	-0.203497589	0.041	0.03	9.88886878961098e-05	no change
7131	7131	CHSY1	4.64760188167178e-09	-0.267515105	0.071	0.056	0.000101522215503238	no change
7132	7132	PARP6	4.66155729638525e-09	-0.108897792	0.068	0.054	0.000101827057582239	no change
7133	7133	RP11-522I20.3	4.68485276936023e-09	0.343854123940711	0.012	0.006	0.000102335923893905	no change
7134	7134	ATP6AP1L	4.72118019000216e-0	0.12383707991625	0.019	0.012	0.00010312946007040	no

			9				7	change
7135	7135	COL15A1	4.73267093424662e-09	-1.374754493	0.012	0.019	0.000103380463887683	down
7136	7136	GRAMD4	4.73861876893077e-09	0.242014499649952	0.022	0.014	0.000103510388388524	no change
7137	7137	KCNK1	4.74450662665252e-09	-0.18776081	0.177	0.148	0.000103639002752598	no change
7138	7138	NTAN1	4.75920659353496e-09	-0.408185552	0.385	0.365	0.000103960108829178	no change
7139	7139	PACS2	4.8148845953391e-09	-0.1186505	0.09	0.073	0.000105176339100587	no change
7140	7140	FKBP2	4.90161380080091e-09	-0.14361047	0.83	0.756	0.000107070851864695	no change
7141	7141	SMIM4	5.05135415265749e-09	-0.133558735	0.453	0.376	0.00011034178011065	no change
7142	7142	ZC3H10	5.11777003315465e-09	0.182919029742248	0.016	0.01	0.00011179256860423	no change
7143	7143	RP11-505K9.1	5.16117992711274e-09	-0.645344401	0.034	0.045	0.000112740814327851	no change
7144	7144	MLKL	5.17269292727275e-09	-0.103754555	0.038	0.027	0.000112992304303346	no change
7145	7145	PHGR1	5.26647192454829e-09	-0.632260147	0.17	0.187	0.000115040812719833	no change
7146	7146	ETV7	5.40762568472189e-09	-0.100104122	0.09	0.073	0.000118124175457065	no change
7147	7147	DPH3	5.47147143861656e-0	-0.18860422	0.239	0.201	0.00011951882210514	no

			9					change
7148	7148	MFAP3	5.49520069472861e-09	0.373854666326976	0.012	0.007	0.000120037163975652	no change
7149	7149	CARD8	5.59083567079971e-09	-0.138142637	0.078	0.062	0.000122126214392949	no change
7150	7150	AC091729.9	5.60713941529283e-09	-0.131799681	0.087	0.07	0.000122482353387657	no change
7151	7151	IKZF1	5.60888018297482e-09	0.238314255317482	0.011	0.006	0.000122520378716902	no change
7152	7152	CREB3	5.66710966823237e-09	-0.230538922	0.194	0.162	0.000123792343592868	no change
7153	7153	SH3D19	5.69701997629149e-09	-0.291127657	0.106	0.087	0.000124445704362111	no change
7154	7154	SERPINE2	5.91844316709956e-09	-1.23946225	0.075	0.087	0.000129282472542123	down
7155	7155	ZNF180	5.99533464891641e-09	0.238212894552922	0.019	0.012	0.00013096209007093	no change
7156	7156	P2RX4	6.06999183885276e-09	-0.214072617	0.096	0.077	0.0001325929017279	no change
7157	7157	FBXL12	6.15373646493575e-09	-0.170347687	0.137	0.114	0.000134422219340056	no change
7158	7158	PARP10	6.16731326888725e-09	-0.184788335	0.069	0.054	0.000134718791045573	no change
7159	7159	RP11-138A9.2	6.27271617745455e-09	-0.312983622	0.083	0.065	0.000137021212180317	no change
7160	7160	RHOU	6.28487421275395e-0	-0.247853526	0.228	0.19	0.00013728679230339	no

			9				7	change
7161	7161	ARMCX1	6.44017439920213e-09	-0.644842554	0.056	0.069	0.000140679169576171	no change
7162	7162	RP5-1142A6.8	6.44310667601535e-09	0.277405902298145	0.011	0.006	0.000140743222230879	no change
7163	7163	FHL2	6.46058875125707e-09	-0.267923739	0.448	0.367	0.00014112510068246	no change
7164	7164	CACNA1H	6.52406522280974e-09	0.548571741228568	0.017	0.01	0.000142511680727056	no change
7165	7165	CTA-293F17.1	6.58497993740522e-09	-0.199204971	0.132	0.109	0.00014384230175268	no change
7166	7166	CHMP2A	7.00745307446332e-09	-0.177011607	0.727	0.644	0.000153070804958577	no change
7167	7167	SMDT1	7.07021887729118e-09	-0.114954651	0.803	0.722	0.000154441861155549	no change
7168	7168	TBRG1	7.07151726574127e-09	-0.177946295	0.229	0.193	0.000154470223152852	no change
7169	7169	NR2F6	7.2004468675138e-09	-0.105650355	0.257	0.218	0.000157286561373972	no change
7170	7170	SGSM2	7.27771983041671e-09	-0.168888702	0.055	0.042	0.000158974511975623	no change
7171	7171	RNASE7	7.36197428010419e-09	-1.18753284	0.018	0.026	0.000160814966174596	down
7172	7172	USP12	7.40804797373746e-09	-0.222970216	0.128	0.106	0.000161821399938321	no change
7173	7173	FDX1L	7.5420374354637e-09	-0.163653761	0.14	0.117	0.00016474826574026	no

							9	change
7174	7174	ZBED6	7.548291092058e-09	-0.155389258	0.084	0.068	0.000164884870614915	no change
7175	7175	SLC39A1	7.5561427004131e-09	-0.15619137	0.344	0.289	0.000165056381147824	no change
7176	7176	C1orf115	7.62559871540468e-09	-0.796187758	0.01	0.016	0.0001665735783393	no change
7177	7177	TNFSF9	7.72971535157024e-09	-1.053889419	0.02	0.029	0.0001688479021397	down
7178	7178	ZNF239	7.7402994865074e-09	0.409820610595565	0.01	0.005	0.000169079101983268	no change
7179	7179	BCAT1	7.81501610191985e-09	-1.347537958	0.009	0.015	0.000170711211730337	down
7180	7180	EFNB3	7.92292245512219e-09	0.242985210053088	0.01	0.005	0.000173068318109689	no change
7181	7181	LINC01268	8.12781176262645e-09	0.227209236569758	0.021	0.014	0.000177543920142812	no change
7182	7182	CTSV	8.17280899906362e-09	0.279008419620103	0.011	0.006	0.000178526839775546	no change
7183	7183	NSFL1C	8.25814753635701e-09	-0.131419333	0.404	0.34	0.000180390974784183	no change
7184	7184	ZNF660	8.28197182195005e-09	0.183614043727555	0.012	0.006	0.000180911392478677	no change
7185	7185	CLK3	8.28816517685651e-09	-0.169485449	0.255	0.215	0.000181046680123254	no change
7186	7186	FRRS1	8.36999328521177e-0	0.13291273699457	0.016	0.01	0.00018283413332216	no

			9	4			6	change
7187	7187	APH1B	8.43159049856136e-09	-0.252092964	0.08	0.064	0.000184179662850574	no change
7188	7188	IL1B	8.48897999623799e-09	-1.174510449	0.071	0.085	0.000185433279037823	down
7189	7189	ALPK1	8.53611466210841e-09	-0.106460805	0.052	0.04	0.000186462888679096	no change
7190	7190	GKN1	8.64737296092787e-09	0.162415010226184	0.016	0.01	0.000188893214958508	no change
7191	7191	CD3D	8.65821899434312e-09	0.27007208717807	0.039	0.029	0.000189130135712431	no change
7192	7192	LCE1E	8.70328724302949e-09	-0.801816685	0.029	0.038	0.000190114606536736	no change
7193	7193	PRKG2	8.70963149857463e-09	0.212770155019243	0.011	0.006	0.000190253190454864	no change
7194	7194	CAV2	8.76928431481488e-09	-0.283261879	0.256	0.213	0.000191556246572816	no change
7195	7195	FAM8A1	8.8186857861384e-09	-0.220609406	0.069	0.055	0.000192635372312407	no change
7196	7196	EBAG9	8.82754858584378e-09	-0.120734658	0.276	0.233	0.000192828971309172	no change
7197	7197	TAF1C	8.97934768079044e-09	-0.135934975	0.054	0.041	0.000196144870739186	no change
7198	7198	IGHG1	9.26591027273993e-09	-3.363656583	0.068	0.081	0.000202404543997731	down
7199	7199	KIAA0895	9.28101487558896e-0	0.37165508031129	0.013	0.007	0.00020273448894236	no

			9	8			5	change
7200	7200	SCGB2A1	9.33915981435194e-09	-2.400556539	0.009	0.016	0.000204004606984704	down
7201	7201	CCDC40	9.48368216390992e-09	0.2262524261702	0.018	0.011	0.000207161553188448	no change
7202	7202	LSM1	9.57556567379301e-09	-0.135809937	0.411	0.338	0.000209168656578335	no change
7203	7203	TRIM35	9.74322525940446e-09	-0.179295889	0.036	0.026	0.000212831012566431	no change
7204	7204	ZNF417	9.81000529275572e-09	0.331640482345931	0.012	0.006	0.000214289755614956	no change
7205	7205	HIST1H2AC	9.83802981309243e-09	-0.1071915	0.17	0.144	0.000214901923237191	no change
7206	7206	ZFP57	1.0069057025207e-08	-0.159467659	0.064	0.049	0.000219948481658622	no change
7207	7207	CAMTA2	1.01499684386282e-08	-0.159910494	0.045	0.034	0.000221715910573395	no change
7208	7208	RHOBTB3	1.01546158287892e-08	-0.17061033	0.442	0.375	0.000221817428164072	no change
7209	7209	FAM110B	1.04262964350185e-08	-0.131893036	0.012	0.006	0.000227752019326544	no change
7210	7210	TNNT2	1.04756061597519e-08	0.239545302214294	0.017	0.01	0.00022882914095362	no change
7211	7211	SLIT3	1.0551784830752e-08	-0.876856305	0.099	0.079	0.000230493187842947	no change
7212	7212	RAB11FIP2	1.06334724809836e-0	-0.103117253	0.069	0.055	0.00023227757287460	no

			8				7	change
7213	7213	TCEAL2	1.066971815463e-08	-1.224423565	0.02	0.028	0.000233069323369738	down
7214	7214	ASAH1	1.07394241002917e-08	-0.197053967	0.757	0.669	0.000234591980046771	no change
7215	7215	GPR78	1.08618786378029e-08	-0.717283138	0.013	0.02	0.000237266876964166	no change
7216	7216	PNP	1.089107156683e-08	-0.202938545	0.158	0.132	0.000237904567305835	no change
7217	7217	DOCK1	1.09609287275544e-08	-0.133501162	0.063	0.049	0.000239430527124697	no change
7218	7218	PROS1	1.10424094494956e-08	-1.18281821	0.02	0.028	0.000241210392014781	down
7219	7219	SBNO2	1.14032509631626e-08	-0.111906764	0.053	0.041	0.000249092614039323	no change
7220	7220	TMEM51-AS1	1.14142077215519e-08	0.202670643556292	0.032	0.023	0.000249331953469581	no change
7221	7221	SVEP1	1.15488113900907e-08	-1.405630169	0.015	0.023	0.00025227223600514	down
7222	7222	CROT	1.19438565758192e-08	-0.196659197	0.453	0.375	0.000260901603042194	no change
7223	7223	KLHL7	1.20389615977169e-08	-0.154778246	0.114	0.094	0.000262979077140529	no change
7224	7224	MYEOV	1.20643598140289e-08	-0.851989008	0.066	0.079	0.000263533875777647	no change
7225	7225	STAC3	1.2165315050001e-08	0.18194495210850	0.011	0.006	0.00026573914195222	no

				3			1	change
7226	7226	HAPLN3	1.22161044622836e-08	0.118810378963284	0.017	0.01	0.000266848585874123	no change
7227	7227	TCN1	1.22192857210895e-08	0.21063185364071	0.104	0.086	0.000266918077291479	no change
7228	7228	PARD6B	1.2332423139706e-08	-0.533112377	0.17	0.183	0.000269389451063739	no change
7229	7229	INTS6-AS1	1.25270212910624e-08	0.249591992517049	0.021	0.013	0.000273640253081968	no change
7230	7230	GEMIN7	1.2920850420692e-08	-0.177456015	0.191	0.162	0.000282243056589595	no change
7231	7231	COQ10B	1.29865195110668e-08	-0.240147984	0.365	0.302	0.000283677532199744	no change
7232	7232	AC096772.6	1.31709382027141e-08	0.189333443480228	0.016	0.01	0.000287705974100086	no change
7233	7233	INAFM1	1.3366412508139e-08	-0.20773087	0.145	0.121	0.000291975914827789	no change
7234	7234	FUCA1	1.35496740433064e-08	-0.140439179	0.381	0.32	0.000295979079801985	no change
7235	7235	THEMIS2	1.3585468262136e-08	-0.815530232	0.019	0.028	0.000296760968718098	no change
7236	7236	LENG9	1.37489422449732e-08	-0.233777285	0.066	0.052	0.000300331894399195	no change
7237	7237	SNX25	1.40756608547502e-08	0.114818902467564	0.021	0.014	0.000307468735711163	no change
7238	7238	LINC00623	1.43095630078635e-0	0.16352740199571	0.018	0.011	0.00031257809434377	no

			8	1				change
7239	7239	JAM2	1.4509274949914e-08	-1.622244559	0.006	0.01	0.000316940602005921	down
7240	7240	GPC5	1.47832610800137e-08	-0.913644852	0.009	0.015	0.00032292555503182	no change
7241	7241	LAT	1.48230453692009e-08	0.248114847174593	0.019	0.012	0.000323794603044824	no change
7242	7242	ZNF263	1.55624294411508e-08	-0.123135451	0.075	0.06	0.000339945708712497	no change
7243	7243	TMEM251	1.61412670175675e-08	-0.153016316	0.246	0.208	0.000352589836731744	no change
7244	7244	PTGDS	1.65538281495857e-08	-1.466618803	0.035	0.045	0.000361601822099549	down
7245	7245	SDCBP2-AS1	1.67669750536594e-08	0.276440302778747	0.018	0.011	0.000366257803072136	no change
7246	7246	CCL17	1.71247166492333e-08	0.855624809414678	0.016	0.009	0.000374072310485852	no change
7247	7247	PPP1R3E	1.73309026950177e-08	-0.146658192	0.084	0.068	0.000378576238469967	no change
7248	7248	IRX3	1.76211142333436e-08	-0.323372164	0.126	0.103	0.000384915619313158	no change
7249	7249	PCDH7	1.7677872483932e-08	-0.571350194	0.121	0.135	0.000386155446539012	no change
7250	7250	ASPHD2	1.77632573122824e-08	0.286655518687875	0.013	0.007	0.000388020592729497	no change
7251	7251	TMEM41A	1.82572020994197e-0	-0.172105869	0.302	0.255	0.00039881032265972	no

			8				5	change
7252	7252	ARHGAP19	1.85560039654084e-08	0.203806260134424	0.013	0.007	0.00040533735062038	no change
7253	7253	FLVCR1	1.85932615147708e-08	-0.207409921	0.072	0.057	0.000406151204528654	no change
7254	7254	SPTLC1	1.86737564317981e-08	-0.149107849	0.056	0.043	0.000407909535496198	no change
7255	7255	MOB3C	1.92925013583734e-08	-0.256391558	0.108	0.088	0.000421425399672308	no change
7256	7256	SH3KBP1	1.94936302823382e-08	-0.180685453	0.094	0.076	0.000425818859887397	no change
7257	7257	CLTCL1	1.95187088092168e-08	0.215114141768991	0.012	0.006	0.000426366675228532	no change
7258	7258	SEL1L	1.97155461609925e-08	-0.20871481	0.129	0.107	0.00043066639034072	no change
7259	7259	RALA	1.99579274888127e-08	-0.148408377	0.303	0.259	0.000435960968065624	no change
7260	7260	KMO	1.99600664594394e-08	-1.178494096	0.006	0.011	0.000436007691739994	down
7261	7261	RP11-725P16.2	2.02021589544733e-08	0.324912036296144	0.011	0.006	0.000441295960201515	no change
7262	7262	CSF2	2.02240151434693e-08	-1.422605014	0.013	0.02	0.000441773386793943	down
7263	7263	UBE2F	2.05715328145511e-08	-0.183345832	0.247	0.209	0.000449364562801054	no change
7264	7264	RP11-304L19.13	2.06107044307004e-0	0.14775705263172	0.019	0.012	0.00045022022758422	no

			8	4				change
7265	7265	RP11-1379J22.5	2.07085624825573e-08	0.229820822059354	0.011	0.006	0.000452357838868982	no change
7266	7266	FERMT2	2.12720687121917e-08	-1.084092208	0.055	0.066	0.000464667068949115	down
7267	7267	HHIP-AS1	2.12949099693612e-08	-1.544123343	0.009	0.015	0.000465166013370726	down
7268	7268	PINK1	2.13804197784903e-08	-0.107807649	0.181	0.154	0.000467033889641341	no change
7269	7269	FAM171A1	2.14751623309914e-08	-0.317229083	0.016	0.01	0.000469103445958176	no change
7270	7270	ANO1	2.17670940755779e-08	-0.235512934	0.021	0.014	0.000475480402986924	no change
7271	7271	SEC24B-AS1	2.18111410011682e-08	0.171600645024926	0.014	0.008	0.000476442564029518	no change
7272	7272	OSER1	2.18166231713254e-08	-0.211029536	0.312	0.261	0.000476562316554433	no change
7273	7273	PCLO	2.21482383435327e-08	0.217248406050109	0.018	0.012	0.000483806118376127	no change
7274	7274	ALG9	2.21818438665354e-08	0.132868184534992	0.017	0.01	0.000484540197420599	no change
7275	7275	KRT15	2.27858308216332e-08	-0.407222437	0.219	0.185	0.000497733688467756	no change
7276	7276	ADGRB2	2.28685784459941e-08	0.222674189912683	0.013	0.007	0.000499541227574296	no change
7277	7277	CLDN11	2.2876258355228e-08	-0.964318368	0.033	0.042	0.00049970898751160	no

							1	change
7278	7278	JOSD2	2.29557367261672e-08	-0.3749786	0.399	0.378	0.000501445113046396	no change
7279	7279	PHC1	2.38274052934074e-08	-0.121054854	0.043	0.033	0.000520485841229192	no change
7280	7280	CYB5RL	2.48340770783197e-08	0.183847789047275	0.016	0.01	0.000542475579698816	no change
7281	7281	FAM76A	2.48599968220636e-08	-0.271990962	0.129	0.107	0.000543041770581158	no change
7282	7282	CCND2-AS1	2.48647892715459e-08	0.110563509953546	0.014	0.008	0.000543146456847649	no change
7283	7283	RP11-277B15.3	2.50294608741678e-08	0.284054496382225	0.012	0.007	0.000546743543335321	no change
7284	7284	RP4-816N1.6	2.51851203198639e-08	0.136507221089547	0.023	0.015	0.000550143768267108	no change
7285	7285	ZNF234	2.52091389383828e-08	0.144521967436662	0.026	0.018	0.000550668430970034	no change
7286	7286	CCDC60	2.52232270009853e-08	-0.106235133	0.049	0.037	0.000550976170609522	no change
7287	7287	CTD-2270P14.5	2.56282083692984e-08	-0.977340764	0.006	0.011	0.000559822583618953	no change
7288	7288	TMEM165	2.58782067429618e-08	-0.229091133	0.644	0.572	0.000565283548093258	no change
7289	7289	TMEM229B	2.59561292598929e-08	0.136376326837405	0.027	0.019	0.000566985687553099	no change
7290	7290	MT-ND6	2.65031901012405e-0	-0.168932295	0.437	0.37	0.00057893568457149	no

			8				8	change
7291	7291	MORN2	2.66780767721667e-08	-0.179608446	0.252	0.213	0.000582755909011209	no change
7292	7292	ARPIN	2.6722625728942e-08	-0.12811517	0.062	0.049	0.000583729036423009	no change
7293	7293	SLC25A23	2.71169196240546e-08	-0.178560607	0.072	0.057	0.000592341992267849	no change
7294	7294	TVP23C	2.72653233808893e-08	0.201860663590994	0.019	0.012	0.000595583723932146	no change
7295	7295	THTPA	2.75739238598161e-08	-0.106270775	0.066	0.052	0.000602324792793822	no change
7296	7296	CPT1A	2.78466826778296e-08	-0.139732539	0.054	0.042	0.00060828293641451	no change
7297	7297	PRADC1	2.7879397955048e-08	-0.166586758	0.208	0.176	0.000608997568930069	no change
7298	7298	LGALS7	2.83724378110971e-08	-0.653165574	0.047	0.058	0.000619767531545605	no change
7299	7299	PRR34-AS1	2.84720640281736e-08	-0.208644727	0.088	0.072	0.000621943766631425	no change
7300	7300	RP5-851M4.1	2.85410570666297e-08	-0.882836937	0.009	0.015	0.000623450850563459	no change
7301	7301	RP1-122P22.4	2.88286535531794e-08	-1.270641443	0.006	0.011	0.00062973310821565	down
7302	7302	ZBTB22	2.90328979869793e-08	-0.130183339	0.062	0.048	0.000634194623627577	no change
7303	7303	PLEKHS1	2.90761163223429e-0	-1.264328286	0.005	0.01	0.00063513868494525	down

			8				9	
7304	7304	PSPN	2.94616714332706e-08	0.475539240015702	0.01	0.005	0.000643560750788364	no change
7305	7305	HPCA	2.95967233646929e-08	0.389521485564082	0.011	0.006	0.000646510825178352	no change
7306	7306	C11orf98	2.96615738124629e-08	0.131000617304529	0.014	0.008	0.00064792741835944	no change
7307	7307	CLK1	2.99488069528057e-08	-0.236800422	0.513	0.421	0.000654201739077087	no change
7308	7308	PRRT1	3.03058064519696e-08	-0.109573199	0.041	0.03	0.000662000036136824	no change
7309	7309	GDPGP1	3.07252781263248e-08	-0.129384019	0.031	0.022	0.000671162975391439	no change
7310	7310	SKIL	3.09483120532253e-08	-0.142844768	0.486	0.409	0.000676034928490653	no change
7311	7311	CYP4F22	3.14883345495082e-08	0.109934906087821	0.075	0.061	0.000687831179899456	no change
7312	7312	HOOK2	3.1882894443055e-08	-0.106796975	0.536	0.447	0.000696449946214092	no change
7313	7313	PYGB	3.19207569895133e-08	-0.197891245	0.08	0.064	0.000697277015678929	no change
7314	7314	FRAS1	3.21720075738289e-08	0.162752439978329	0.027	0.019	0.000702765333442718	no change
7315	7315	LINC01629	3.25046797056115e-08	-0.867351789	0.01	0.017	0.000710032223489378	no change
7316	7316	SREBF2	3.27537234872383e-0	-0.15857982	0.109	0.09	0.00071547233585523	no

			8				4	change
7317	7317	RFX2	3.30451957110271e-08	-0.247886261	0.111	0.092	0.000721839255111675	no change
7318	7318	ZNF646	3.33269693258263e-08	0.184679034576607	0.018	0.012	0.000727994317953349	no change
7319	7319	SIK2	3.36426210895196e-08	-0.136343526	0.038	0.028	0.000734889415079466	no change
7320	7320	CTXN1	3.36972627652061e-08	0.122723483318203	0.035	0.025	0.000736083007843161	no change
7321	7321	TRIM47	3.42489196555159e-08	-0.234531877	0.132	0.11	0.000748133400955089	no change
7322	7322	ACADL	3.43967428343327e-08	-1.174092746	0.005	0.01	0.000751362450473163	down
7323	7323	PDGFRB	3.50261351686844e-08	-1.392437682	0.011	0.017	0.000765110896624741	down
7324	7324	HIVEP3	3.55634756599421e-08	-0.170205833	0.045	0.034	0.000776848562315774	no change
7325	7325	FUK	3.68280542222636e-08	-0.120770535	0.034	0.025	0.000804472016431126	no change
7326	7326	CDH24	3.70262622728069e-08	0.41482111100305	0.011	0.006	0.000808801673087193	no change
7327	7327	SMARCD3	3.73896643838201e-08	-0.235956685	0.052	0.04	0.000816739828800166	no change
7328	7328	CPQ	3.78309868935694e-08	-0.459272444	0.128	0.106	0.000826380077703129	no change
7329	7329	ZNF197	3.78399370145487e-0	0.11186933984806	0.03	0.021	0.00082657558414580	no

			8	3			3	change
7330	7330	SVIL	3.91049061257486e-08	-0.188758667	0.174	0.147	0.000854207569410852	no change
7331	7331	ZNF18	3.95407406824781e-08	0.118277634659538	0.017	0.01	0.000863727939468052	no change
7332	7332	AC016831.7	3.99216166098381e-08	-0.193731283	0.037	0.027	0.000872047793225304	no change
7333	7333	NFKBIB	4.07865872483333e-08	-0.216839278	0.176	0.148	0.000890942211852594	no change
7334	7334	ACOT8	4.19798821255166e-08	-0.163248923	0.165	0.139	0.000917008545149784	no change
7335	7335	RSRP1	4.20208075569101e-08	-0.116639061	0.535	0.453	0.000917902520273145	no change
7336	7336	BAMBI	4.2506692502447e-08	-0.621371249	0.251	0.255	0.000928516191023453	no change
7337	7337	CSGALNACT1	4.2731378180081e-08	-1.05475687	0.009	0.015	0.000933424224965689	down
7338	7338	NBPF20	4.28147516713725e-08	0.267312157677117	0.013	0.008	0.000935245435509461	no change
7339	7339	SULF2	4.28522976465043e-08	-0.209028563	0.088	0.072	0.00093606558979024	no change
7340	7340	ZNF229	4.31757257198934e-08	0.480539636982273	0.01	0.006	0.000943130552625351	no change
7341	7341	ZNF8	4.3870710403102e-08	0.183456180916013	0.017	0.01	0.00095831179804536	no change
7342	7342	HAS2	4.40599574674852e-0	-1.135356562	0.03	0.039	0.00096244571091974	down

			8				6	
7343	7343	ANGPTL1	4.4394084842707e-08	-1.765212324	0.005	0.01	0.000969744389304091	down
7344	7344	TIAM2	4.45719159025811e-08	0.132695184777609	0.017	0.011	0.000973628930975982	no change
7345	7345	PIK3C2B	4.4754488669957e-08	-0.129339209	0.095	0.079	0.000977617050506542	no change
7346	7346	MACC1	4.52138138620409e-08	-0.106498076	0.089	0.073	0.000987650550002422	no change
7347	7347	PAM	4.5908284144467e-08	-0.957642598	0.104	0.116	0.00100282055885174	no change
7348	7348	PARP12	4.63906627996855e-08	-0.16893997	0.098	0.081	0.00101335763819633	no change
7349	7349	MICA	4.7006863946942e-08	-0.187669085	0.12	0.1	0.001026817936057	no change
7350	7350	OPHN1	4.72792173194401e-08	-0.111325947	0.027	0.019	0.00103276722312585	no change
7351	7351	STAMBPL1	4.72969068811246e-08	-0.110180685	0.032	0.023	0.00103315363391129	no change
7352	7352	TRAM2-AS1	4.73898351596275e-08	0.1863449123917	0.026	0.018	0.0010351835592269	no change
7353	7353	RP11-315O6.1	4.78228281389177e-08	0.137468826726882	0.02	0.013	0.00104464185786652	no change
7354	7354	FZD4	4.88731348150784e-08	-0.810901527	0.022	0.03	0.00106758475690057	no change
7355	7355	SETD7	4.92678890325931e-0	-0.237492215	0.084	0.068	0.00107620776802796	no

			8					change
7356	7356	HOGA1	4.93692904743173e-08	0.288350293266754	0.017	0.011	0.00107842278112099	no change
7357	7357	SSBP3	5.02262768315682e-08	-0.193179749	0.08	0.064	0.00109714279110878	no change
7358	7358	MAP3K11	5.08167188197318e-08	-0.22213062	0.214	0.181	0.00111004040589822	no change
7359	7359	TMEM178B	5.12437581257894e-08	0.362594555322627	0.016	0.01	0.00111936865249974	no change
7360	7360	ZNF467	5.15032001252829e-08	-0.965434058	0.007	0.011	0.00112503590353668	no change
7361	7361	HPS5	5.49033324727218e-08	-0.187946056	0.044	0.033	0.00119930839453414	no change
7362	7362	BLNK	5.50191638745051e-08	-0.544369355	0.097	0.11	0.00120183861567469	no change
7363	7363	TMEM199	5.52668686714029e-08	-0.142946041	0.154	0.13	0.00120724947925813	no change
7364	7364	CHODL	5.55421602409944e-08	-1.105543346	0.006	0.011	0.00121326294830428	down
7365	7365	PRKCE	5.56714311362674e-08	-0.171922858	0.05	0.038	0.00121608674174063	no change
7366	7366	CD3E	5.57893489617137e-08	0.288833844224643	0.015	0.01	0.00121866253871967	no change
7367	7367	TSHZ3	5.6560371279862e-08	-0.185518912	0.017	0.011	0.00123550475023731	no change
7368	7368	PPP1R13L	5.78522603451377e-0	-0.183294544	0.235	0.201	0.00126372477497919	no

			8					change
7369	7369	LINC01535	5.7942532550463e-08	0.28764961198516	0.019	0.012	0.00126569668103231	no change
7370	7370	B4GALT7	5.81370992543589e-08	-0.246119079	0.208	0.175	0.00126994679611222	no change
7371	7371	LINC00680	5.86842786790548e-08	0.126074207259895	0.029	0.021	0.00128189938346527	no change
7372	7372	SGMS2	5.90239380242567e-08	-0.198557659	0.095	0.078	0.00128931890220186	no change
7373	7373	TTC39B	5.90471246908737e-08	-0.106380169	0.033	0.024	0.00128982539174744	no change
7374	7374	IGF2	6.03611969264911e-08	0.420534921397551	0.159	0.14	0.00131852998566227	no change
7375	7375	HSPA2	6.16322345137561e-08	-0.166969851	0.054	0.042	0.00134629453071849	no change
7376	7376	FRMD6-AS2	6.18300680074408e-08	-0.30840059	0.032	0.023	0.00135061600555454	no change
7377	7377	STC1	6.18840562371382e-08	-0.455648752	0.012	0.007	0.00135179532444405	no change
7378	7378	CYTH3	6.22559669948192e-08	-0.167197048	0.015	0.009	0.00135991934303483	no change
7379	7379	LRRC36	6.27340163537003e-08	0.381211265364791	0.01	0.005	0.00137036185323023	no change
7380	7380	FAM213B	6.30706027327318e-08	-0.177536317	0.133	0.111	0.00137771424609379	no change
7381	7381	ADCK5	6.32712654184979e-0	-0.152887488	0.043	0.033	0.00138209752180167	no

			8					change
7382	7382	ANKRD36C	6.32883245190765e-08	-0.111878925	0.017	0.011	0.00138247016079471	no change
7383	7383	CMC2	6.49354749661209e-08	-0.17520843	0.337	0.284	0.00141845051515994	no change
7384	7384	BBIP1	6.49472851595644e-08	-0.161069122	0.204	0.174	0.00141870849702552	no change
7385	7385	ZNF837	6.53347873054041e-08	0.138626065928563	0.019	0.012	0.00142717309389925	no change
7386	7386	RGS2	6.61475501123606e-08	-0.804803916	0.076	0.088	0.00144492708465441	no change
7387	7387	RP4-555D20.2	6.64026452620662e-08	0.258817912782784	0.011	0.006	0.00145049938310457	no change
7388	7388	KB-1615E4.2	6.6621190701147e-08	-0.796240947	0.017	0.025	0.00145527328967585	no change
7389	7389	ZNF616	6.82877192282564e-08	0.155333826330077	0.025	0.017	0.00149167693882203	no change
7390	7390	DEF8	6.85246293180247e-08	-0.176082715	0.179	0.152	0.00149685200282293	no change
7391	7391	RP11-390P24.1	7.05839514834268e-08	0.329458025209963	0.01	0.005	0.00154183583620398	no change
7392	7392	RDH10	7.07200621663211e-08	-0.287097092	0.142	0.12	0.00154480903796112	no change
7393	7393	TMEM170B	7.10771270478766e-08	0.349838701631863	0.012	0.007	0.00155260876323382	no change
7394	7394	LYST	7.15866629674842e-0	-0.189374123	0.139	0.117	0.00156373906586172	no

			8					change
7395	7395	ZNF480	7.18158210547995e-08	-0.177446507	0.154	0.13	0.00156874479512104	no change
7396	7396	DMRTA1	7.26076901735733e-08	0.374092568861235	0.018	0.012	0.00158604238415153	no change
7397	7397	F3	7.26161409307473e-08	-0.679972521	0.101	0.082	0.00158622698249124	no change
7398	7398	LINC01290	7.31796836955946e-08	0.111180032720299	0.011	0.006	0.00159853701064657	no change
7399	7399	AGAP6	7.3512974089943e-08	0.118245996874404	0.019	0.012	0.00160581740602071	no change
7400	7400	CD163	7.35497814027141e-08	-1.559482609	0.005	0.01	0.00160662142496089	down
7401	7401	CUEDC1	7.39304520036515e-08	-0.165649061	0.033	0.024	0.00161493679356776	no change
7402	7402	SPRED1	7.516700159914e-08	-0.129081732	0.067	0.053	0.00164194798293161	no change
7403	7403	LINC00839	7.57542136736052e-08	-0.559030409	0.036	0.046	0.00165477504348623	no change
7404	7404	CABP4	7.59134977571492e-08	0.106210853337608	0.031	0.022	0.00165825444500717	no change
7405	7405	EHD4	7.70008247031866e-08	-0.16166772	0.394	0.332	0.00168200601481641	no change
7406	7406	CRACR2B	7.72231994912081e-08	-0.469868953	0.272	0.274	0.00168686356968595	no change
7407	7407	ITGA5	7.78422943185722e-0	-1.228341732	0.027	0.036	0.00170038707709489	down

			8					
7408	7408	PGBD2	7.82176828965446e-08	-0.121879809	0.046	0.035	0.00170858706519212	no change
7409	7409	NPR1	8.08624471185681e-08	-1.907333048	0.006	0.01	0.001766359294858	down
7410	7410	SLC22A18	8.15215436827978e-08	-0.24893404	0.138	0.115	0.00178075660020703	no change
7411	7411	PDE4C	8.28605604427615e-08	-0.588078034	0.065	0.077	0.00181000608231168	no change
7412	7412	SLC46A2	8.31898764984298e-08	-0.991208229	0.011	0.017	0.0018171996622317	no change
7413	7413	PPP2R3A	8.36938557061363e-08	-0.139649139	0.032	0.023	0.00182820858404484	no change
7414	7414	KRTAP5-9	8.52853755834804e-08	0.283946534111052	0.01	0.006	0.00186297374424555	no change
7415	7415	MLF1	8.54651365554331e-08	-0.244322331	0.204	0.173	0.00186690044291688	no change
7416	7416	CTD-2292M14.1	8.67760730544576e-08	-0.883710901	0.006	0.01	0.00189553653980157	no change
7417	7417	IRAK3	8.69307453783257e-08	-0.47728683	0.045	0.034	0.00189891520204415	no change
7418	7418	MRC2	8.94458665659319e-08	-0.588017498	0.054	0.041	0.00195385550926622	no change
7419	7419	NMU	8.96840805701086e-08	0.18795570163257	0.018	0.011	0.00195905905597345	no change
7420	7420	YME1L1	9.00566666819408e-0	-0.230812724	0.334	0.282	0.00196719782700031	no

			8					change
7421	7421	AC008079.10	9.04865832956773e-08	0.180732810642132	0.011	0.006	0.00197658892551077	no change
7422	7422	APOBEC3C	9.23344791506256e-08	-0.165911945	0.382	0.326	0.00201695436256627	no change
7423	7423	HMGA1P4	9.30935347192445e-08	0.248950425715502	0.011	0.006	0.00203353517240718	no change
7424	7424	CEP128	9.33007709577334e-08	0.126532986593872	0.01	0.006	0.00203806204080073	no change
7425	7425	CTA-228A9.4	9.37393767163757e-08	0.171433304259589	0.011	0.006	0.00204764294499251	no change
7426	7426	BCAP29	9.54559541341039e-08	-0.203151781	0.296	0.251	0.00208513986210537	no change
7427	7427	ZFC3H1	1.00361828187518e-07	-0.300668871	0.247	0.209	0.00219230377492814	no change
7428	7428	EIF1B	1.00557426676379e-07	-0.131096113	0.585	0.485	0.00219657642831883	no change
7429	7429	CASP3	1.01116223912451e-07	-0.156713435	0.172	0.146	0.00220878279514357	no change
7430	7430	KDELR2	1.0126127540853e-07	-0.1960029	0.729	0.656	0.00221195130002394	no change
7431	7431	HCFC2	1.0270737391425e-07	-0.110229521	0.037	0.027	0.00224353987578287	no change
7432	7432	ZNF217	1.02845721295086e-07	-0.237336764	0.196	0.167	0.00224656193596986	no change
7433	7433	AC004510.3	1.05930318144962e-0	-0.499510533	0.038	0.048	0.00231394186955855	no

			7					change
7434	7434	OVOL2	1.0601066100641e-07	-0.104159171	0.044	0.033	0.00231569687902402	no change
7435	7435	HDAC4	1.06433316148227e-07	-0.163566199	0.018	0.012	0.00232492935794188	no change
7436	7436	ZBTB25	1.09994326765479e-07	-0.121769881	0.058	0.046	0.00240271607386513	no change
7437	7437	ZNF577	1.11089054139248e-07	-0.143966438	0.033	0.024	0.00242662929861774	no change
7438	7438	TMSB4Y	1.11122544488111e-07	-0.513823549	0.057	0.069	0.00242736086179829	no change
7439	7439	GOSR2	1.14844463350359e-07	-0.1696333	0.119	0.1	0.00250866245742524	no change
7440	7440	POLDIP2	1.15591431021473e-07	-0.152302378	0.349	0.294	0.00252497921923305	no change
7441	7441	HELZ2	1.15608093531527e-07	-0.616315127	0.111	0.123	0.00252534319510268	no change
7442	7442	PDK2	1.24312584823069e-07	-0.136711852	0.144	0.122	0.00271548410287511	no change
7443	7443	AP000695.4	1.26196268090223e-07	-0.752469394	0.01	0.016	0.00275663128016283	no change
7444	7444	COL28A1	1.26405925317767e-07	0.409476334192657	0.01	0.006	0.0027612110326413	no change
7445	7445	LAMP2	1.26610153258378e-07	-0.298101862	0.314	0.263	0.00276567218777602	no change
7446	7446	RP11-1391J7.1	1.27061601172224e-0	-0.116178402	0.074	0.06	0.00277553361600607	no

			7					change
7447	7447	ANKFN1	1.27584415891602e-07	0.313179894518804	0.011	0.006	0.00278695398073615	no change
7448	7448	EMC7	1.29798804167904e-07	-0.192810946	0.362	0.303	0.0028353250782437	no change
7449	7449	HSPB2	1.3088825468737e-07	-1.420211	0.009	0.015	0.0028591230353909	down
7450	7450	TET1	1.32027177729925e-07	-0.816666673	0.026	0.035	0.00288400167033249	no change
7451	7451	FAM118B	1.33958801804304e-07	-0.102628172	0.054	0.042	0.00292619606661322	no change
7452	7452	C5AR1	1.3471078414626e-07	-1.421807168	0.009	0.014	0.00294262236889091	down
7453	7453	ZNF334	1.36046836133219e-07	0.331484301543343	0.011	0.006	0.00297180708849403	no change
7454	7454	DGCR6L	1.36246165127059e-07	-0.130122597	0.452	0.376	0.00297616123103548	no change
7455	7455	CRLF1	1.37112421174611e-07	-0.576732151	0.029	0.02	0.00299508372813821	no change
7456	7456	RRS1-AS1	1.37883680573861e-07	0.359416168225904	0.011	0.006	0.00301193111845542	no change
7457	7457	SLC38A5	1.38064303048553e-07	0.181550649153024	0.045	0.035	0.0030158766357926	no change
7458	7458	DNAJB6	1.40065252366226e-07	-0.175101283	0.795	0.711	0.00305958537268784	no change
7459	7459	ASMTL	1.42151867439896e-07	-0.210084225	0.065	0.052	0.0031051653923571	no change
7460	7460	LLNLR-304G9.1	1.43212281402193e-0	0.15551494455938	0.018	0.012	0.00312832907494951	no

			7	1				change
7461	7461	PHF20	1.43952816356746e-07	-0.265629239	0.274	0.231	0.00314450532049677	no change
7462	7462	PTCH1	1.45082190469331e-07	-0.332077434	0.024	0.017	0.00316917536861206	no change
7463	7463	NPL	1.46111681876447e-07	-0.215017161	0.117	0.097	0.00319166357890911	no change
7464	7464	LINC00685	1.46245070667605e-07	-0.615326963	0.129	0.141	0.00319457732366316	no change
7465	7465	KRT6A	1.46879811223033e-07	-0.343253539	0.09	0.104	0.00320844259635592	no change
7466	7466	PPP3CC	1.48250316765124e-07	-0.172834217	0.08	0.065	0.00323837991941737	no change
7467	7467	FCGR3A	1.5007096040241e-07	-1.661193654	0.009	0.015	0.00327815005903024	down
7468	7468	TOM1	1.51341105373271e-07	-0.225255206	0.083	0.067	0.00330589510577372	no change
7469	7469	HERPUD2	1.51790845691606e-07	-0.254255464	0.143	0.12	0.00331571923328744	no change
7470	7470	ZNF430	1.51809869300234e-07	-0.212525424	0.188	0.16	0.00331613478499432	no change
7471	7471	EFHC1	1.52497859300446e-07	-0.194669125	0.17	0.145	0.00333116323855894	no change
7472	7472	POLR2K	1.52553021336804e-07	-0.203730327	0.747	0.653	0.00333236819808115	no change
7473	7473	ZGLP1	1.53511279061522e-07	0.162494039573979	0.011	0.006	0.00335330037981988	no change

7474	7474	TOMM34	1.53715984634964e-07	-0.179715656	0.186	0.158	0.00335777196836616	no change
7475	7475	CAPN8	1.55047580783227e-07	-0.527456899	0.114	0.127	0.00338685935462881	no change
7476	7476	TMEM54	1.55913820803658e-07	-0.103547728	0.569	0.473	0.0034057815016351	no change
7477	7477	NR1H2	1.56220558497344e-07	-0.184790341	0.289	0.246	0.00341248187981597	no change
7478	7478	QPCT	1.57673409256672e-07	-0.190767878	0.26	0.219	0.00344421795180273	no change
7479	7479	TXNL4B	1.6333139919492e-07	-0.134763066	0.055	0.043	0.00356781108401383	no change
7480	7480	U47924.31	1.64856635227779e-07	0.125434158359387	0.015	0.01	0.00360112833991559	no change
7481	7481	ADCK4	1.67093919322739e-07	-0.167688695	0.074	0.06	0.00364999957368591	no change
7482	7482	EPHA2	1.67259006436004e-07	-0.117412779	0.435	0.373	0.00365360573658808	no change
7483	7483	PIGBOS1	1.67823356468424e-07	-0.187859717	0.163	0.139	0.00366593339869626	no change
7484	7484	THRA	1.69437353551643e-07	-0.154618622	0.056	0.044	0.0037011895509821	no change
7485	7485	LACC1	1.71617813622865e-07	-0.180765382	0.044	0.034	0.00374881952077785	no change
7486	7486	ZNF777	1.71870715997284e-07	0.369203257553884	0.015	0.009	0.00375434392024466	no change

7487	7487	TMEM116	1.72014713696342e-07	-0.20242415	0.149	0.125	0.00375748940598289	no change
7488	7488	RP11-677M14.3	1.72191828573297e-07	0.320838853622196	0.011	0.006	0.0037613583033551	no change
7489	7489	GPR34	1.7257757570768e-07	-1.648817431	0.007	0.012	0.00376978456375856	down
7490	7490	TPCN1	1.74346619879979e-07	-0.233824079	0.085	0.07	0.00380842756465826	no change
7491	7491	PGM3	1.7442167652352e-07	-0.433487444	0.095	0.078	0.00381006710197977	no change
7492	7492	RP11-483F11.7	1.75276676109489e-07	-0.129972792	0.021	0.014	0.00382874371293569	no change
7493	7493	ZNF672	1.76212082842672e-07	-0.139030026	0.117	0.098	0.00384917673761533	no change
7494	7494	RP11-398C13.6	1.79172955148869e-07	0.165220684071254	0.019	0.013	0.00391385403227189	no change
7495	7495	ZNF525	1.80193016451191e-07	0.124976145968349	0.019	0.013	0.00393613625135983	no change
7496	7496	RPS6KA2	1.81012628647543e-07	-0.180722597	0.046	0.036	0.00395403986017693	no change
7497	7497	DNMT3B	1.81447205446886e-07	0.235000897345705	0.014	0.009	0.00396353275578177	no change
7498	7498	LRRC59	1.81592257032434e-07	-0.114929289	0.387	0.33	0.0039667012626165	no change
7499	7499	ZNF573	1.83490860013581e-07	0.119099640556412	0.02	0.014	0.00400817434613667	no change
7500	7500	CREB3L2	1.84611350842121e-0	-0.207380167	0.181	0.154	0.00403265034779528	no

			7					change
7501	7501	WTAP	1.84754717945847e-07	-0.300347581	0.542	0.507	0.00403578205880908	no change
7502	7502	MFSD5	1.84889420597778e-07	-0.151972914	0.139	0.118	0.00403872450353787	no change
7503	7503	CEBPA	1.86369034041768e-07	-0.559014319	0.179	0.189	0.00407104517960837	no change
7504	7504	TBC1D17	1.89170518264613e-07	-0.174909987	0.113	0.095	0.00413224080097221	no change
7505	7505	SP100	1.90065885252048e-07	-0.182398257	0.327	0.276	0.00415179919744573	no change
7506	7506	HEBP2	1.92025302359904e-07	-0.185849741	0.708	0.633	0.00419460070474975	no change
7507	7507	BRICD5	1.94386629964804e-07	0.109898867452403	0.013	0.008	0.00424618154495119	no change
7508	7508	PRICKLE2-AS1	1.94594476909439e-07	-1.260375504	0.01	0.015	0.00425072175360978	down
7509	7509	PRR15	1.95479387489297e-07	-0.551681673	0.152	0.163	0.00427005174031619	no change
7510	7510	HCFC1R1	1.98284260761285e-07	-0.491821513	0.401	0.372	0.00433132139206951	no change
7511	7511	LINC-PINT	2.0182044467316e-07	-0.143946942	0.052	0.041	0.0044085657934405	no change
7512	7512	BCL9L	2.03723241820549e-07	-0.167413764	0.078	0.063	0.00445013049432807	no change
7513	7513	WDR86	2.04492492525098e-0	0.11840195365739	0.012	0.007	0.00446693400671824	no

			7	9				change
7514	7514	MBNL1-AS1	2.05039892377434e-07	-0.154541541	0.028	0.02	0.00447889140909267	no change
7515	7515	SERPINB3	2.06446218242855e-07	-0.298463666	0.09	0.074	0.00450961119129693	no change
7516	7516	BAIAP2L2	2.06707658541452e-07	0.333032179515948	0.015	0.009	0.00451532209317947	no change
7517	7517	ZXDA	2.06875577776296e-07	0.128758080289854	0.017	0.011	0.00451899012094541	no change
7518	7518	STOX1	2.08073760528749e-07	0.241903337068788	0.014	0.009	0.00454516322498999	no change
7519	7519	MYO5A	2.12331996204669e-07	-0.122883627	0.019	0.013	0.00463818012509478	no change
7520	7520	RIMKLB	2.13607078679001e-07	-0.171106486	0.064	0.051	0.0046660330266641	no change
7521	7521	BNIP3L	2.14386480604251e-07	-0.106640442	0.483	0.408	0.00468305828231927	no change
7522	7522	RP5-892K4.1	2.16461506658325e-07	0.323324946610537	0.01	0.006	0.00472838515144444	no change
7523	7523	BBS7	2.18541823032222e-07	-0.194267563	0.08	0.066	0.00477382758231586	no change
7524	7524	RIMKLA	2.2005478588564e-07	-0.642572774	0.015	0.022	0.00480687674288593	no change
7525	7525	SLC41A1	2.22988253742993e-07	-0.166818161	0.08	0.065	0.00487095541476194	no change
7526	7526	IFI16	2.23229569862093e-0	-0.160161187	0.423	0.357	0.00487622672406755	no

			7						change
7527	7527	ZNF503-AS2	2.24340863604559e-07	0.128818905332859	0.037	0.028	0.00490050182457799	no change
7528	7528	TMEM53	2.27173617730253e-07	-0.16961475	0.144	0.123	0.00496238050569965	no change
7529	7529	FUT11	2.37096597644877e-07	-0.112065215	0.06	0.048	0.0051791380789547	no change
7530	7530	MVD	2.38411145198063e-07	-0.522120702	0.187	0.196	0.00520785305570649	no change
7531	7531	DRAM1	2.39487062149846e-07	-0.581673591	0.096	0.108	0.00523135538560123	no change
7532	7532	RASSF1-AS1	2.39689655049555e-07	-0.665301508	0.009	0.015	0.00523578082490248	no change
7533	7533	PRDM15	2.41554953385548e-07	0.222947779958876	0.015	0.009	0.00527652640175391	no change
7534	7534	SAMD9L	2.51160749979385e-07	-0.763314245	0.051	0.061	0.0054863554225497	no change
7535	7535	BCL6	2.59531263767612e-07	-0.273846001	0.17	0.144	0.00566920092573972	no change
7536	7536	SLC35D2	2.59795367493787e-07	-0.101196878	0.072	0.059	0.00567497000753428	no change
7537	7537	FILIP1	2.62197917342675e-07	-1.127057208	0.011	0.017	0.0057274513064334	down
7538	7538	MX1	2.74213467356618e-07	-0.20428141	0.266	0.228	0.00598991898093796	no change
7539	7539	NME6	2.77105117925321e-0	-0.199800325	0.094	0.078	0.00605308419596071	no

			7					change
7540	7540	GPD1L	2.81719996046545e-07	-0.200260003	0.145	0.123	0.00615389159364074	no change
7541	7541	SPDYA	2.83501217633028e-07	0.384831353199602	0.01	0.005	0.00619280059797587	no change
7542	7542	NCOA7	2.83507860205141e-07	-0.383042716	0.381	0.373	0.00619294569832111	no change
7543	7543	TAX1BP3	2.91972672169928e-07	-0.105265516	0.566	0.468	0.0063778510508799	no change
7544	7544	KCTD9	2.94758856331749e-07	-0.300286534	0.083	0.068	0.00643871245771073	no change
7545	7545	PAQR3	2.97641931837334e-07	-0.103053961	0.051	0.04	0.00650169035905472	no change
7546	7546	LINC01436	2.99981780773164e-07	-0.181936582	0.03	0.022	0.006552802019209	no change
7547	7547	SVIL-AS1	3.0001336039924e-07	-0.183862935	0.082	0.068	0.00655349184456101	no change
7548	7548	THBS2	3.00266609565114e-07	-0.842281594	0.063	0.074	0.00655902381934034	no change
7549	7549	PRICKLE4	3.03915793898414e-07	0.135637180162371	0.016	0.01	0.00663873660191696	no change
7550	7550	DNAJA1	3.06312014062158e-07	-0.341082531	0.714	0.635	0.00669107963517377	no change
7551	7551	HMGCS2	3.10537315062673e-07	0.266801081418777	0.426	0.408	0.00678337711022903	no change
7552	7552	PEAK1	3.12466675116638e-0	-0.313821808	0.049	0.038	0.00682552205124784	no

			7					change
7553	7553	DISC1	3.13619836787799e-07	0.164948786676709	0.014	0.009	0.00685071171479268	no change
7554	7554	RP11-49I11.1	3.19642190458507e-07	-0.111692841	0.086	0.072	0.00698226400837563	no change
7555	7555	RAB30-AS1	3.20992681926903e-07	-0.168829579	0.167	0.143	0.00701176414401127	no change
7556	7556	COL9A3	3.21335672373542e-07	-0.124197387	0.063	0.05	0.00701925642732764	no change
7557	7557	FAM188A	3.22288524744287e-07	-0.226299512	0.077	0.063	0.0070400705345142	no change
7558	7558	MAPK8IP2	3.26122760702369e-07	0.125101957036581	0.011	0.006	0.00712382558478256	no change
7559	7559	PTPRD	3.33840867875206e-07	-1.452448664	0.009	0.014	0.00729241991786599	down
7560	7560	HTRA1	3.37497450461257e-07	-1.251567626	0.105	0.114	0.00737229430787571	down
7561	7561	PCIF1	3.45864257754833e-07	-0.202743993	0.145	0.123	0.00755505884639657	no change
7562	7562	LOX	3.4818431780507e-07	-1.368452171	0.012	0.018	0.00760573823813396	down
7563	7563	UST	3.50503755714965e-07	-0.247059356	0.053	0.042	0.0076564040398377	no change
7564	7564	TFPI2	3.54685714711187e-07	-0.101121125	0.044	0.034	0.00774775475215116	no change
7565	7565	ALDH3B1	3.57387529245637e-07	-0.702672665	0.031	0.04	0.0078067731888417	no change

7566	7566	C15orf62	3.61638568436257e-07	-1.027981032	0.005	0.01	0.0078996328889216	down
7567	7567	TMEM184A	3.62357187205196e-07	-0.541412162	0.142	0.154	0.0079153303973103	no change
7568	7568	CPED1	3.65526200309488e-07	-1.405690256	0.009	0.014	0.00798455431956046	down
7569	7569	NR2F1	3.75425138991641e-07	-1.056612535	0.029	0.037	0.00820078673613341	down
7570	7570	XXbac-B476C20.9	3.85455600853602e-07	0.243659109244176	0.018	0.012	0.00841989214504608	no change
7571	7571	RP11-21B23.2	3.91214949584312e-07	-1.280073859	0.005	0.01	0.00854569935871971	down
7572	7572	UBE2B	3.93416591182677e-07	-0.198901758	0.69	0.608	0.0085937920177944	no change
7573	7573	TTLL1	3.98468440905814e-07	-0.150482356	0.047	0.037	0.00870414462314659	no change
7574	7574	PIGH	4.04044042101332e-07	-0.179059635	0.151	0.129	0.00882593805566151	no change
7575	7575	CLCN2	4.06678791197897e-07	0.353012771037543	0.01	0.005	0.00888349151492687	no change
7576	7576	ARHGEF17	4.12349852348506e-07	0.101345846928735	0.018	0.012	0.00900737017470076	no change
7577	7577	CCL15	4.13974311886446e-07	-0.317606164	0.117	0.098	0.00904285486884754	no change
7578	7578	TMEM208	4.15938275612773e-07	-0.330593392	0.501	0.459	0.00908575569248541	no change

7579	7579	OSBPL1A	4.17437390578584e-07	-0.554567163	0.136	0.147	0.00911850235979859	no change
7580	7580	AC009005.2	4.1931634276284e-07	0.113030515599315	0.023	0.017	0.00915954619131149	no change
7581	7581	BLOC1S1	4.1949760219942e-07	-0.147498394	0.757	0.666	0.00916350562244413	no change
7582	7582	RASSF4	4.20096364038982e-07	-1.144503338	0.022	0.03	0.00917658497606751	down
7583	7583	CLMP	4.20308180819692e-07	-0.961107517	0.016	0.023	0.00918121190182535	no change
7584	7584	RP3-394A18.1	4.23137858548809e-07	-0.117056574	0.032	0.024	0.00924302338214019	no change
7585	7585	C2orf42	4.27421402832475e-07	-0.118621464	0.027	0.02	0.00933659312347258	no change
7586	7586	LACE1	4.31335300172289e-07	-0.122709847	0.024	0.017	0.00942208829696348	no change
7587	7587	RNF25	4.315993549062e-07	-0.170622751	0.047	0.037	0.00942785630857104	no change
7588	7588	LAMC1	4.36400531978586e-07	-0.323378099	0.163	0.138	0.00953273322054022	no change
7589	7589	GPC6	4.38964751324416e-07	-1.328883894	0.008	0.013	0.00958874602793054	down
7590	7590	LLGL2	4.44354891882273e-07	-0.125577515	0.178	0.154	0.00970648825827638	no change
7591	7591	MTERF2	4.47644499857548e-07	-0.219903442	0.07	0.057	0.00977834645488828	no change

7592	7592	CNIH4	4.55998599294459e-07	-0.103673524	0.609	0.506	0.00996083340298817	no change
7593	7593	CNRIP1	4.59110557519963e-07	-1.348455966	0.01	0.016	0.0100288110184661	down
7594	7594	RP11-195F19.5	4.72048801309088e-07	-0.24089972	0.038	0.029	0.0103114340157957	no change
7595	7595	DFNB59	4.73866457984218e-07	0.31714727762203	0.01	0.006	0.0103511389082073	no change
7596	7596	PRICKLE2	4.7604750494013e-07	-0.934402784	0.023	0.03	0.0103987816979122	no change
7597	7597	CSF3R	4.84568940803913e-07	-0.981350315	0.015	0.022	0.0105849239429207	no change
7598	7598	DTX3	4.88096181969431e-07	-0.2348955	0.084	0.069	0.0106619729989403	no change
7599	7599	MMP3	4.93448933226173e-07	0.180824796494056	0.011	0.007	0.0107788984973925	no change
7600	7600	SOCS6	4.93901040998851e-07	-0.125912731	0.085	0.07	0.0107887743395789	no change
7601	7601	CCDC92	4.97113960572829e-07	-0.141513925	0.077	0.063	0.0108589573547529	no change
7602	7602	GALNT5	5.00143354872673e-07	-0.612050406	0.027	0.035	0.0109251314438387	no change
7603	7603	HS1BP3	5.08995827927868e-07	-0.11271706	0.079	0.065	0.0111185048652563	no change
7604	7604	DLGAP1-AS1	5.12552294030153e-07	-0.222124256	0.159	0.136	0.0111961923107947	no change

7605	7605	RP11-480A16.1	5.20259149151279e-07	0.193310141185369	0.018	0.012	0.0113645408540605	no change
7606	7606	CTAG2	5.22463670277806e-07	-0.493269435	0.058	0.069	0.0114126964135484	no change
7607	7607	FMOD	5.23746167663219e-07	-1.44023721	0.012	0.017	0.0114407112864353	down
7608	7608	RYBP	5.25045874687984e-07	-0.220226909	0.25	0.214	0.0114691020866843	no change
7609	7609	BTBD9	5.28188735865828e-07	-0.160029127	0.05	0.039	0.0115377547462531	no change
7610	7610	RP11-890B15.2	5.29384539224136e-07	0.235963058484257	0.01	0.005	0.011563875874812	no change
7611	7611	POU3F1	5.3719293091371e-07	-0.286315012	0.04	0.03	0.0117344423828791	no change
7612	7612	RAPGEF5	5.38849944060902e-07	0.216429623579934	0.011	0.006	0.0117706381780663	no change
7613	7613	ZNF846	5.58300543539774e-07	-0.126537812	0.108	0.091	0.0121955170730828	no change
7614	7614	TXK	5.61390329676678e-07	0.179047323007532	0.024	0.017	0.0122630103614574	no change
7615	7615	AGER	5.69265281713161e-07	0.166788195373664	0.013	0.008	0.0124350308137423	no change
7616	7616	AIF1	5.74207267405637e-07	-0.87403971	0.042	0.052	0.0125429835492087	no change
7617	7617	TMC8	5.78828768163918e-07	-0.969617938	0.016	0.022	0.0126439356117726	no change

7618	7618	RAPGEFL1	5.84395454679463e-07	-0.214786863	0.157	0.134	0.0127655343120182	no change
7619	7619	NAGA	5.85723244523164e-07	-0.145385407	0.08	0.066	0.012794538553364	no change
7620	7620	CCDC134	5.86100451261061e-07	0.262182665387883	0.014	0.008	0.0128027782573466	no change
7621	7621	IKZF5	5.86690084291501e-07	-0.148298388	0.056	0.045	0.0128156582012636	no change
7622	7622	ZNF510	5.8885506302534e-07	0.146961096576744	0.01	0.006	0.0128629499967255	no change
7623	7623	LIN7B	5.90541329150693e-07	-0.161100151	0.101	0.085	0.0128997847939677	no change
7624	7624	CA5B	5.91490087857222e-07	-0.155138161	0.026	0.018	0.0129205094791532	no change
7625	7625	C8orf37	5.9184911399009e-07	0.172950436263573	0.012	0.007	0.0129283520459995	no change
7626	7626	LINC01139	5.94291708807314e-07	-0.831524822	0.007	0.011	0.012981708087187	no change
7627	7627	ISL2	6.03919375223249e-07	0.297130888805057	0.01	0.006	0.0131920148323766	no change
7628	7628	RP11-66B24.4	6.05665619814063e-07	0.330840793198589	0.011	0.007	0.0132301597992184	no change
7629	7629	PLEKHG6	6.109366175027e-07	-0.11224093	0.089	0.075	0.013345299472729	no change
7630	7630	RP4-660H19.1	6.16827809559486e-07	0.238553018037294	0.016	0.01	0.0134739866720174	no change

7631	7631	ETV3	6.20126452487153e-07	-0.214583105	0.184	0.158	0.0135460422281294	no change
7632	7632	UCHL1	6.25805482360797e-07	-0.660571782	0.045	0.035	0.0136700949566893	no change
7633	7633	CSF3	6.35561792806247e-07	-1.444364778	0.014	0.02	0.0138832118020596	down
7634	7634	P4HA1	6.41066987143935e-07	-0.16760563	0.285	0.244	0.0140034672671721	no change
7635	7635	CSRNP1	6.73222727233133e-07	-0.535873909	0.299	0.292	0.0147058772536806	no change
7636	7636	TSPAN31	6.95965097607227e-07	-0.284362992	0.324	0.277	0.0152026615921323	no change
7637	7637	PDGFA	7.11645841808047e-07	-0.270250819	0.077	0.063	0.015545191768455	no change
7638	7638	RP11-319G6.1	7.14502316218438e-07	0.250988317651592	0.013	0.008	0.0156075885954756	no change
7639	7639	LYRM5	7.20695184107867e-07	-0.202609186	0.232	0.199	0.0157428656016522	no change
7640	7640	FENDRR	7.22666699848471e-07	-1.585970092	0.006	0.01	0.01578593139149	down
7641	7641	SPTSSA	7.25068959501825e-07	-0.339974476	0.557	0.501	0.0158384063513579	no change
7642	7642	FGD4	7.42738369148449e-07	-0.302500423	0.046	0.036	0.0162243769356787	no change
7643	7643	OLFM4	7.5272430892597e-07	-0.515622276	0.057	0.068	0.0164425098041789	no change

7644	7644	RAP2C	7.61670222448749e-07	-0.22591437	0.116	0.098	0.0166379243391705	no change
7645	7645	OTUD1	7.61817296674753e-07	-0.244787344	0.089	0.074	0.0166411370285633	no change
7646	7646	ZNF862	7.70965887993313e-07	0.188527742326436	0.011	0.006	0.0168409788573259	no change
7647	7647	STX11	7.77377510931444e-07	-0.130555515	0.06	0.048	0.0169810343487865	no change
7648	7648	RP11-428J1.5	7.82299652431633e-07	0.192906203776634	0.017	0.011	0.0170885536077166	no change
7649	7649	SNRK	7.83526558115612e-07	-0.205931949	0.059	0.047	0.0171153541354774	no change
7650	7650	CD83	7.83759220614589e-07	-0.941481858	0.074	0.085	0.0171204364151051	no change
7651	7651	EDEM1	7.86100293697188e-07	-0.157655481	0.101	0.085	0.0171715748155214	no change
7652	7652	RP5-1068E13.7	7.88735379286246e-07	0.368912878713299	0.01	0.006	0.0172291356251287	no change
7653	7653	PFKFB4	8.09913452863957e-07	-0.663585762	0.019	0.026	0.0176917494643603	no change
7654	7654	TPPP3	8.22061309385698e-07	-1.146101719	0.06	0.07	0.0179571072422212	down
7655	7655	MCOLN1	8.46774700813819e-07	-0.172964059	0.131	0.111	0.0184969465645771	no change
7656	7656	HAGH	8.47843135581318e-07	-0.127618211	0.352	0.301	0.0185202854536383	no change

7657	7657	ALAS1	8.57465883451127e-07	-0.423330282	0.269	0.27	0.0187304847581064	no change
7658	7658	IRF2BPL	8.62703590893395e-07	-0.286149943	0.347	0.295	0.0188448972394753	no change
7659	7659	OAF	8.75585982137986e-07	-0.284306512	0.126	0.106	0.0191263001938222	no change
7660	7660	ATXN7L2	8.77971924912249e-07	0.261033338092019	0.015	0.01	0.0191784187277832	no change
7661	7661	ARMC5	8.79717220743319e-07	-0.259224167	0.037	0.028	0.0192165429699171	no change
7662	7662	TPRN	8.80229196929913e-07	-0.242269109	0.04	0.03	0.019227726577737	no change
7663	7663	RP11-303E16.6	8.81646456733015e-07	-0.3353996	0.011	0.017	0.019258685200876	no change
7664	7664	RP11-1275H24.3	8.83385971154502e-07	0.129198480611916	0.022	0.016	0.019296683153899	no change
7665	7665	SHC2	8.97618467852305e-07	-0.103286901	0.014	0.009	0.0196075778117658	no change
7666	7666	ZNF471	8.98699447161082e-07	0.274031548231403	0.014	0.009	0.0196311907237867	no change
7667	7667	CRIPT	8.9906465703162e-07	-0.16566649	0.484	0.406	0.0196391683681987	no change
7668	7668	YPEL3	9.03051689832043e-07	-0.430742979	0.371	0.347	0.0197262611126912	no change
7669	7669	ZFAT	9.06933812528803e-07	0.405052066181796	0.011	0.007	0.0198110622008792	no change

7670	7670	IRAK2	9.19710119919307e-07	-0.170362692	0.088	0.073	0.0200901478595173	no change
7671	7671	LINC00115	9.32778893957372e-07	0.113121798578883	0.025	0.018	0.0203756221596048	no change
7672	7672	DEPDC4	9.58350124317576e-07	0.278680906953228	0.01	0.006	0.0209342001155931	no change
7673	7673	GM2A	9.72826740048232e-07	-0.263905826	0.163	0.139	0.0212504273096136	no change
7674	7674	PDGFRL	9.80982233028557e-07	-1.58997705	0.014	0.02	0.0214285758982758	down
7675	7675	PTGES	1.00328232208334e-06	-0.443001313	0.245	0.248	0.0219156990435885	no change
7676	7676	CDC42EP5	1.0067940113362e-06	-0.326717172	0.494	0.449	0.0219924083836279	no change
7677	7677	MIR181A1HG	1.00869377479697e-06	-0.907084087	0.007	0.012	0.0220339068166651	no change
7678	7678	TINF2	1.02548976651761e-06	-0.148324083	0.158	0.136	0.0224007984598108	no change
7679	7679	NPRL2	1.03264306904231e-06	-0.179688709	0.1	0.084	0.0225570552001603	no change
7680	7680	PLD6	1.0340254201303e-06	0.202330829967181	0.011	0.006	0.0225872512773262	no change
7681	7681	RPS6KC1	1.034941623103e-06	-0.151402358	0.054	0.043	0.0226072648150619	no change
7682	7682	BMP8B	1.04696852401844e-06	0.515624417122889	0.01	0.006	0.0228699804386588	no change

7683	7683	SP2-AS1	1.04859379441004e-06	0.214070640377093	0.015	0.01	0.0229054828450929	no change
7684	7684	ZNF880	1.06517871410909e-06	-0.121451137	0.078	0.064	0.0232677638309989	no change
7685	7685	RP11-127B20.2	1.07675075854146e-06	-0.157426165	0.021	0.014	0.0235205435695796	no change
7686	7686	FAM160B1	1.07675532501898e-06	-0.127775367	0.045	0.036	0.0235206433197146	no change
7687	7687	TMC6	1.07736818016317e-06	-0.448542179	0.184	0.193	0.0235340305274842	no change
7688	7688	ZNF329	1.07762421159147e-06	0.161979451429644	0.015	0.01	0.023539623278004	no change
7689	7689	ZKSCAN3	1.07980108745615e-06	0.24123728729221	0.018	0.012	0.0235871749543922	no change
7690	7690	STK36	1.09550147587469e-06	0.135948012843946	0.028	0.02	0.0239301342390067	no change
7691	7691	PIM3	1.09948494186428e-06	-0.291624262	0.595	0.539	0.0240171490700834	no change
7692	7692	UPK3A	1.1045199986332e-06	-0.239466284	0.354	0.371	0.0241271348501436	no change
7693	7693	INPP4B	1.11524695797029e-06	-0.169343349	0.157	0.135	0.0243614545499029	no change
7694	7694	LRRC3	1.12308826317824e-06	0.370324004273296	0.011	0.007	0.0245327400208654	no change
7695	7695	WDSUB1	1.12741704008481e-06	-0.164867891	0.054	0.043	0.0246272978236126	no change

7696	7696	RASSF1	1.13106681592847e-06	-0.266559163	0.062	0.05	0.0247070235271414	no change
7697	7697	ZSCAN2	1.1347089482117e-06	0.118528370981144	0.036	0.028	0.0247865822647364	no change
7698	7698	RWDD3	1.13483825791522e-06	0.371598243858489	0.01	0.006	0.0247894069059	no change
7699	7699	HLA-DRB1	1.1460471406353e-06	-0.2762485	0.408	0.421	0.0250342537400375	no change
7700	7700	GMPPA	1.15213887662889e-06	-0.230860446	0.127	0.108	0.0251673216210814	no change
7701	7701	RCAN2	1.16709419378863e-06	-1.177073539	0.027	0.035	0.0254940055691188	down
7702	7702	BBS4	1.17778106952927e-06	-0.129460399	0.042	0.032	0.0257274496827973	no change
7703	7703	RP11-637A17.2	1.19226318532068e-06	-0.267267121	0.03	0.022	0.0260437970201449	no change
7704	7704	PRKRIP1	1.21976726527175e-06	-0.173450919	0.162	0.139	0.0266445961425961	no change
7705	7705	GRB10	1.22126246116859e-06	-0.213538832	0.055	0.044	0.0266772572017667	no change
7706	7706	SOX12	1.23381551625986e-06	-0.148475896	0.025	0.018	0.0269514661371804	no change
7707	7707	ABCB8	1.24435899516222e-06	-0.196332913	0.064	0.052	0.0271817778903235	no change
7708	7708	SIKE1	1.25541139031328e-06	-0.190215302	0.197	0.17	0.0274232064100032	no change

7709	7709	LAMP5	1.26324482034608e-06	-0.714882707	0.007	0.012	0.0275943198556397	no change
7710	7710	EGR3	1.26400075455036e-06	-0.324653829	0.083	0.068	0.027610832482398	no change
7711	7711	C1QB	1.28077009573636e-06	-1.565436808	0.041	0.05	0.027977141971265	down
7712	7712	MAP10	1.28139454418332e-06	0.342125566128235	0.01	0.006	0.0279907824231405	no change
7713	7713	TMEM147-AS1	1.31820956815247e-06	0.196882036351272	0.02	0.014	0.0287949698067226	no change
7714	7714	TWSG1	1.3263625763643e-06	-0.561070406	0.291	0.243	0.0289730641181017	no change
7715	7715	PPP1R12B	1.3295030631054e-06	-0.249322691	0.09	0.075	0.0290416649104743	no change
7716	7716	ZNF485	1.33502662422559e-06	0.193736287237151	0.015	0.01	0.0291623215795839	no change
7717	7717	RNF170	1.34435740654902e-06	-0.240640249	0.113	0.095	0.0293661431886567	no change
7718	7718	ASAP1	1.35053842582185e-06	-0.186700042	0.103	0.087	0.0295011613736525	no change
7719	7719	TMEM86A	1.37073376596942e-06	-0.880119255	0.01	0.015	0.029942308383836	no change
7720	7720	KLHL15	1.37134124690463e-06	-0.29712642	0.037	0.028	0.0299555781973847	no change
7721	7721	RP11-399K21.13	1.37225742756671e-06	0.293007372911832	0.011	0.006	0.0299755912477671	no change

7722	7722	PAQR7	1.38688204163545e-06	-0.503803457	0.178	0.189	0.0302950513174848	no change
7723	7723	LRIG2	1.41251481973303e-06	-0.180674883	0.029	0.021	0.0308549737222484	no change
7724	7724	SIX3	1.41520190542781e-06	0.170331147523673	0.056	0.046	0.030913670422165	no change
7725	7725	MUC4	1.42118671889682e-06	-0.823714186	0.006	0.01	0.031044402687582	no change
7726	7726	LRRC6	1.42619021425116e-06	-0.648324357	0.021	0.028	0.0311536990401024	no change
7727	7727	ROR1	1.432990978474e-06	-0.472307182	0.016	0.011	0.0313022549337861	no change
7728	7728	RP11-231D20.2	1.43710701072937e-06	-0.156123459	0.04	0.031	0.0313921655423724	no change
7729	7729	PON3	1.46325946479077e-06	0.186573235576056	0.01	0.006	0.0319634397488896	no change
7730	7730	ZNF251	1.487141223251e-06	0.186037524200636	0.016	0.01	0.0324851128806948	no change
7731	7731	ZNF335	1.49533876865225e-06	-0.267067688	0.062	0.05	0.0326641800624398	no change
7732	7732	FUZ	1.49830706604583e-06	-0.120703675	0.094	0.079	0.0327290195507052	no change
7733	7733	TM2D1	1.49916030360747e-06	-0.117761264	0.557	0.465	0.0327476576720016	no change
7734	7734	MXRA7	1.52814749418314e-06	-0.787019927	0.055	0.065	0.0333808538629366	no change

7735	7735	RP11-983P16.4	1.56374402634639e-06	-0.207793596	0.047	0.037	0.0341584245115104	no change
7736	7736	RP11-390E23.6	1.60162806953052e-06	-0.477246539	0.145	0.156	0.0349859635508248	no change
7737	7737	PEX13	1.61992823113159e-06	-0.203819878	0.296	0.253	0.0353857122808385	no change
7738	7738	ENTPD3-AS1	1.65521576452601e-06	-0.255266949	0.153	0.132	0.0361565331603062	no change
7739	7739	MPP6	1.6886541771984e-06	0.190581482127839	0.023	0.016	0.0368869618467218	no change
7740	7740	RIN1	1.69443222851992e-06	-0.247587998	0.039	0.03	0.0370131775997891	no change
7741	7741	RP11-479G22.8	1.73396969456046e-06	0.318162564750016	0.01	0.006	0.0378768340079788	no change
7742	7742	CDH11	1.73434242781936e-06	-1.259982839	0.008	0.013	0.0378849759932861	down
7743	7743	RP11-44F14.2	1.74573413452726e-06	0.258971526938923	0.012	0.007	0.0381338164346135	no change
7744	7744	RP11-115C21.2	1.7705457734037e-06	-0.143274966	0.075	0.062	0.0386758018742305	no change
7745	7745	RP11-90L1.8	1.79446003107633e-06	-0.797061395	0.019	0.026	0.0391981849188315	no change
7746	7746	CCDC167	1.80586088298199e-06	-0.132945538	0.361	0.314	0.0394472251278585	no change
7747	7747	VTI1A	1.81309567988732e-06	-0.128318221	0.064	0.052	0.0396052620314586	no change

7748	7748	OSBPL10-AS1	1.82344320349557e-06	0.170348456166201	0.012	0.007	0.0398312933371572	no change
7749	7749	FAM71D	1.82540763749586e-06	0.148448738801941	0.015	0.01	0.0398742044334596	no change
7750	7750	RP11-166P13.3	1.83381281081759e-06	-0.10468599	0.051	0.041	0.0400578070394994	no change
7751	7751	ANGEL1	1.86807757496106e-06	0.187974885955808	0.02	0.014	0.0408062865474495	no change
7752	7752	L3HYPDH	1.92914918735314e-06	-0.119490748	0.021	0.015	0.042140334848542	no change
7753	7753	GABRA3	1.94557669804216e-06	0.507536456775152	0.013	0.008	0.0424991773920329	no change
7754	7754	ZBTB20-AS2	1.95397558597632e-06	0.155478584390175	0.011	0.006	0.0426826427000667	no change
7755	7755	RP11-498M15.1	1.96247952749057e-06	-0.900572426	0.025	0.033	0.0428684027985041	no change
7756	7756	ZNF750	2.00911892146791e-06	-0.174678126	0.174	0.15	0.043887193720545	no change
7757	7757	XYLT2	2.0285501747447e-06	-0.235166985	0.047	0.037	0.0443116500171232	no change
7758	7758	ARPC1B	2.04448412653206e-06	-0.190567074	0.692	0.62	0.0446597112599664	no change
7759	7759	SERTAD2	2.06330034439308e-06	-0.224353076	0.204	0.176	0.0450707327229225	no change
7760	7760	INO80B	2.07041772367275e-06	-0.117877999	0.03	0.022	0.0452262047559077	no change

7761	7761	RP11-820I16.4	2.08297682713683e-06	-0.610707677	0.023	0.031	0.045500545811977	no change
7762	7762	KHDC1	2.08324374719131e-06	0.494048651976558	0.011	0.006	0.045506376413647	no change
7763	7763	ZNF17	2.1257154381274e-06	0.160105004413016	0.013	0.008	0.046434128030455	no change
7764	7764	RP11-680A11.5	2.17286045153215e-06	0.358990355576299	0.01	0.006	0.0474639637032682	no change
7765	7765	RERG	2.21415263840773e-06	-0.567165435	0.023	0.016	0.0483659502333785	no change
7766	7766	LBX2-AS1	2.2341079937877e-06	-0.705193081	0.015	0.021	0.0488018550162984	no change
7767	7767	SAV1	2.23483558856383e-06	-0.267550985	0.077	0.064	0.0488177485965883	no change
7768	7768	BUD31	2.24110302429794e-06	-0.183620159	0.646	0.58	0.0489546544627643	no change
7769	7769	MAF	2.31191273640131e-06	-0.8535337	0.084	0.094	0.0505014218139503	no change
7770	7770	HSDL1	2.31339096355751e-06	-0.119828841	0.059	0.048	0.0505337122079503	no change
7771	7771	NLRP1	2.31763430965172e-06	-0.248375848	0.055	0.044	0.0506264038600321	no change
7772	7772	MYH14	2.33155281663748e-06	-0.161896787	0.215	0.187	0.0509304397266291	no change
7773	7773	FYCO1	2.36408616585899e-06	-0.115336244	0.047	0.038	0.0516410982070237	no change

7774	7774	CCDC126	2.37356358710058e-06	-0.197830761	0.097	0.082	0.0518481229966252	no change
7775	7775	SCARB2	2.39171654105115e-06	-0.197537141	0.379	0.319	0.0522446561227213	no change
7776	7776	CRB3	2.3973673072823e-06	-0.434815488	0.409	0.373	0.0523680914602746	no change
7777	7777	EFCAB2	2.41725038822559e-06	-0.13194383	0.093	0.079	0.0528024174803997	no change
7778	7778	FBXL19-AS1	2.41887886737785e-06	0.203417160908216	0.012	0.008	0.0528379899790018	no change
7779	7779	NUMBL	2.42740984269531e-06	-0.282620498	0.055	0.044	0.0530243406038364	no change
7780	7780	ADA	2.43598038679854e-06	-0.289435026	0.033	0.025	0.0532115555692273	no change
7781	7781	PPM1D	2.44461983019643e-06	-0.213247797	0.046	0.037	0.0534002755708108	no change
7782	7782	PLCG1	2.44644008791079e-06	-0.206094739	0.038	0.029	0.0534400372803234	no change
7783	7783	C9orf72	2.45060927997258e-06	-0.279408266	0.062	0.05	0.0535311091117211	no change
7784	7784	BDKRB2	2.45276726030702e-06	-1.22337811	0.013	0.019	0.0535782480341465	down
7785	7785	PBX3	2.4545744627877e-06	-0.308975847	0.038	0.029	0.0536177245651345	no change
7786	7786	ZNF713	2.46861405947713e-06	0.103999176211066	0.011	0.007	0.0539244055152185	no change

7787	7787	WNT2B	2.47188262369004e-06	-1.270315525	0.03	0.037	0.0539958040318853	down
7788	7788	CAMK1	2.49312384549755e-06	-0.31283216	0.051	0.04	0.0544597972810485	no change
7789	7789	GJB1	2.494399768244e-06	-1.15588784	0.007	0.011	0.0544876685375219	down
7790	7790	RARRES3	2.4956045286962e-06	-0.5444865	0.339	0.28	0.0545139853248398	no change
7791	7791	U47924.27	2.49640366662558e-06	-0.200050184	0.284	0.244	0.0545314416937692	no change
7792	7792	ZNF25	2.54911622561088e-06	-0.152251732	0.024	0.018	0.0556828948322442	no change
7793	7793	SLC6A11	2.55628312979016e-06	0.494681346079465	0.018	0.012	0.0558394486871362	no change
7794	7794	LINC01089	2.56812807318062e-06	-0.124016629	0.095	0.08	0.0560981896305574	no change
7795	7795	NEBL	2.59650968760375e-06	-0.21983275	0.082	0.068	0.0567181576160163	no change
7796	7796	CD36	2.61953334991465e-06	-0.751812414	0.048	0.057	0.0572210864955355	no change
7797	7797	NOL3	2.62139413378879e-06	-0.267140998	0.169	0.145	0.0572617334584823	no change
7798	7798	FSIP2	2.63195667016315e-06	0.18630864195103	0.026	0.019	0.0574924615030438	no change
7799	7799	TFPT	2.65394785338329e-06	-0.18188311	0.307	0.262	0.0579728369093045	no change
7800	7800	PDZD7	2.75642601334538e-0	-0.719018821	0.009	0.014	0.0602113698355165	no

			6					change
7801	7801	NCAM1	2.76593014309403e-06	-1.171389257	0.011	0.016	0.0604189780457461	down
7802	7802	LINC01134	2.79585491333975e-06	0.424424739945295	0.01	0.006	0.0610726547269935	no change
7803	7803	RP11-10L12.4	2.80184005562701e-06	-0.134122356	0.034	0.026	0.0612033941751163	no change
7804	7804	DYX1C1	2.84004601205829e-06	-0.135567513	0.033	0.025	0.0620379650874013	no change
7805	7805	WASL	2.85078473685141e-06	-0.172919854	0.358	0.304	0.0622725417917823	no change
7806	7806	ZNF77	2.85320995579666e-06	-0.116153582	0.022	0.016	0.0623255182744223	no change
7807	7807	ZNF226	2.92418354860224e-06	-0.479765772	0.157	0.167	0.0638758654356673	no change
7808	7808	TBC1D12	3.02194275629701e-06	-0.240451206	0.046	0.036	0.0660113175685519	no change
7809	7809	RILP	3.0290249403545e-06	-0.229825536	0.133	0.114	0.0661660207971037	no change
7810	7810	SMPDL3A	3.16969361312506e-06	-0.69292468	0.154	0.161	0.0692387872851037	no change
7811	7811	HIST2H2BE	3.22634045134984e-06	-0.531092578	0.086	0.098	0.070476180819286	no change
7812	7812	TNNT1	3.24679566856568e-06	-0.597984543	0.03	0.038	0.0709230045841488	no change
7813	7813	DCUN1D2	3.24869919259972e-0	0.15089282634033	0.018	0.013	0.0709645851631484	no

			6	2				change
7814	7814	ZSWIM4	3.34447317610626e-06	-0.292780485	0.061	0.049	0.0730566720588652	no change
7815	7815	NFATC2	3.37319724928681e-06	-0.66533522	0.042	0.051	0.0736841207134211	no change
7816	7816	GSTK1	3.39198669420942e-06	-0.219603127	0.757	0.654	0.0740945573483106	no change
7817	7817	SPTBN2	3.39925150904682e-06	0.284913631766923	0.012	0.008	0.0742532499636187	no change
7818	7818	GCC1	3.46718073043649e-06	-0.15990253	0.042	0.033	0.0757370958756547	no change
7819	7819	ZNF415	3.52045187750432e-06	0.127209984520437	0.028	0.021	0.0769007508122044	no change
7820	7820	RP11-598F7.4	3.53354536646189e-06	0.318666124856912	0.014	0.009	0.0771867649849936	no change
7821	7821	FDX1	3.65014033680193e-06	-0.175038963	0.52	0.429	0.0797336655171014	no change
7822	7822	PTCHD1	3.68302183586597e-06	-0.521721878	0.031	0.039	0.0804519289826563	no change
7823	7823	CTD-2015H6.3	3.8015571766321e-06	-0.186004257	0.264	0.229	0.0830412149663517	no change
7824	7824	STAB1	3.83265198248261e-06	-1.494587563	0.008	0.012	0.0837204499053502	down
7825	7825	SMIM3	3.84945026451212e-06	-0.240169625	0.048	0.038	0.0840873915780027	no change
7826	7826	NUPR2	3.93608890449958e-0	-0.109876215	0.126	0.108	0.0859799260298888	no

			6					change
7827	7827	ZNF625	4.02864182592845e-06	0.320880660534406	0.011	0.007	0.0880016520455811	no change
7828	7828	NUDT17	4.03029870427369e-06	0.230102984588096	0.01	0.006	0.0880378448961545	no change
7829	7829	CTB-58E17.3	4.08236558379702e-06	-0.75810693	0.015	0.02	0.0891751938124622	no change
7830	7830	HSF4	4.18706642308287e-06	-0.217812573	0.028	0.021	0.0914622789458222	no change
7831	7831	USP32	4.19305333054131e-06	-0.146016197	0.066	0.055	0.0915930569523444	no change
7832	7832	RP1-313I6.12	4.21247775943656e-06	-0.525052778	0.276	0.275	0.0920173641771322	no change
7833	7833	YIPF2	4.22730230044321e-06	-0.198036699	0.28	0.241	0.0923411914508815	no change
7834	7834	COL17A1	4.35809722042032e-06	-0.521923486	0.071	0.081	0.0951982756828614	no change
7835	7835	TMEM136	4.39599966991651e-06	-0.255375773	0.032	0.024	0.0960262167896562	no change
7836	7836	SOSTDC1	4.51930859575368e-06	-1.413484589	0.006	0.011	0.0987197769656434	down
7837	7837	FAM110C	4.70453668681115e-06	-0.213070603	0.187	0.162	0.102765899386703	no change
7838	7838	IGFBP7	4.74126811209786e-06	-1.002866002	0.433	0.4	0.103568260640666	down
7839	7839	HIST3H2A	4.79151137605881e-0	-0.778680863	0.187	0.19	0.104665774498629	no

			6					change
7840	7840	CNN1	4.79988633770618e-06	-0.800699051	0.011	0.016	0.104848717160854	no change
7841	7841	COL12A1	4.89750075225788e-06	-0.313575782	0.062	0.051	0.106981006432321	no change
7842	7842	MUC15	4.9125457027621e-06	-0.289301774	0.03	0.023	0.107309648331135	no change
7843	7843	HOXA11	4.9257849606181e-06	0.14131207615219	0.018	0.012	0.107598846679742	no change
7844	7844	SGSH	4.93780457703937e-06	-0.140158929	0.046	0.037	0.107861403180848	no change
7845	7845	GPR65	4.96208031766549e-06	0.146778503057915	0.017	0.012	0.108391682459085	no change
7846	7846	CRY2	5.00408848948944e-06	-0.272941717	0.047	0.037	0.109309308964407	no change
7847	7847	CEP164	5.06573014149239e-06	-0.174741288	0.086	0.072	0.11065580921076	no change
7848	7848	DNASE1L1	5.10868665650569e-06	-0.253762089	0.085	0.072	0.11159415132471	no change
7849	7849	FAM177A1	5.11868410221971e-06	-0.255640337	0.598	0.53	0.111812535528887	no change
7850	7850	MGMT	5.17891263579177e-06	-0.138150991	0.553	0.462	0.113128167616235	no change
7851	7851	RP11-452L6.5	5.18333089405902e-06	0.287904772675162	0.012	0.008	0.113224680049825	no change
7852	7852	ZKSCAN2	5.27716079662458e-0	0.15755459305335	0.01	0.006	0.115274300441467	no

			6	6				change
7853	7853	GPAM	5.27996762377315e-06	0.133081123321347	0.019	0.013	0.115335612773701	no change
7854	7854	SLC25A15	5.33440457828743e-06	0.102488885287038	0.013	0.008	0.116524733608111	no change
7855	7855	AP1G1	5.43791733564863e-06	-0.371001358	0.193	0.165	0.118785866279909	no change
7856	7856	AC240274.1	5.51042716858342e-06	0.136341343055077	0.012	0.008	0.120369771070536	no change
7857	7857	RASSF7	5.51554375884329e-06	-0.175856904	0.374	0.319	0.120481537868173	no change
7858	7858	ESR1	5.53180074590304e-06	-1.122134268	0.006	0.01	0.120836655493506	down
7859	7859	RHOG	5.54524934792997e-06	-0.237278447	0.225	0.195	0.121130426756182	no change
7860	7860	RNH1	5.58805534877233e-06	-0.133906919	0.601	0.498	0.122065481038583	no change
7861	7861	TRAPPC2L	5.64065995546398e-06	-0.124313828	0.54	0.454	0.123214576067155	no change
7862	7862	CASC10	5.6713990393871e-06	0.119564502492449	0.03	0.023	0.123886040616372	no change
7863	7863	RP4-621F18.2	5.74107568125847e-06	-1.219698478	0.009	0.013	0.12540805718141	down
7864	7864	SPIN4	5.75887024649844e-06	-0.156549772	0.026	0.019	0.125796761664512	no change
7865	7865	SULT1A2	5.82005316458562e-0	0.17646154007720	0.024	0.018	0.127133241327208	no

			6	5				change
7866	7866	RP11-73E17.2	5.83298568277453e-06	-0.123441316	0.039	0.031	0.127415739254527	no change
7867	7867	TNIK	5.86287657763283e-06	0.132864691700251	0.018	0.013	0.128068675961811	no change
7868	7868	MSRB3	5.90672000959818e-06	-1.218790956	0.012	0.017	0.129026391889663	down
7869	7869	IL20RB	5.98260954748624e-06	-0.137639449	0.015	0.01	0.13068412295529	no change
7870	7870	CD14	6.09309964523423e-06	-1.654734467	0.018	0.025	0.133097668650497	down
7871	7871	RP5-864K19.4	6.12927518193922e-06	-0.868777769	0.011	0.016	0.13388788707428	no change
7872	7872	TMUB1	6.13420613351639e-06	-0.120427554	0.364	0.315	0.133995598780532	no change
7873	7873	MS4A4A	6.17899746701294e-06	-1.546064066	0.008	0.012	0.134974020669431	down
7874	7874	CHMP6	6.17972949110008e-06	-0.165958942	0.112	0.096	0.13499001100359	no change
7875	7875	RP11-72M17.1	6.22193287259158e-06	0.127855775768804	0.01	0.006	0.13591190166889	no change
7876	7876	IFIT5	6.24303222759346e-06	-0.139863228	0.055	0.045	0.136372795979552	no change
7877	7877	CD2	6.27444844913092e-06	0.541532184817903	0.016	0.011	0.137059051922816	no change
7878	7878	ZNF584	6.27892622557741e-0	-0.126389874	0.032	0.024	0.137156864471513	no

			6					change
7879	7879	SSPN	6.36779954945622e-06	-0.51474164	0.222	0.188	0.139098213358322	no change
7880	7880	SERPINB9	6.54643653241176e-06	-0.270342495	0.038	0.03	0.143000359614003	no change
7881	7881	MEF2D	6.56871559097007e-06	-0.262538351	0.068	0.056	0.14348702336915	no change
7882	7882	LGI4	6.6347659582412e-06	-0.95721294	0.012	0.017	0.144929827591821	no change
7883	7883	DHDDS	6.64800013428194e-06	-0.232847812	0.086	0.073	0.145218914933255	no change
7884	7884	BCL2L1	6.65293626753036e-06	-0.496230319	0.226	0.228	0.145326739827933	no change
7885	7885	ADAMTS2	6.85793969390216e-06	-1.293256673	0.008	0.013	0.149804834673599	down
7886	7886	TCEA3	6.89981899138359e-06	-0.264272721	0.246	0.21	0.150719646047783	no change
7887	7887	PLSCR2	6.90176186927473e-06	0.185217620160674	0.01	0.006	0.150762086272437	no change
7888	7888	CALU	6.92064516333077e-06	-0.291683144	0.34	0.291	0.151174572947797	no change
7889	7889	CCDC53	6.92694560022038e-06	-0.183261228	0.342	0.292	0.151312199691214	no change
7890	7890	PRRG2	6.94503296578481e-06	-0.171526826	0.121	0.104	0.151707300104603	no change
7891	7891	SLC38A6	6.98562347226448e-0	-0.196743313	0.085	0.072	0.152593959128145	no

			6					change
7892	7892	CHPF	7.01153080715378e-06	-0.444922463	0.263	0.261	0.153159878951467	no change
7893	7893	WBSCR27	7.01266734078077e-06	-0.550613235	0.111	0.121	0.153184705392015	no change
7894	7894	WDR44	7.04797835074038e-06	-0.185335418	0.05	0.04	0.153956039093573	no change
7895	7895	INVS	7.11241140267711e-06	0.199595850789032	0.013	0.008	0.155363514680079	no change
7896	7896	OPLAH	7.1881740698861e-06	-0.104047627	0.022	0.016	0.157018474382592	no change
7897	7897	CLCA2	7.19462779289025e-06	-0.328311878	0.159	0.172	0.157159449507895	no change
7898	7898	JHDM1D-AS1	7.2465282892597e-06	-0.727041583	0.026	0.034	0.158293163950589	no change
7899	7899	ZFHX2	7.2790138230478e-06	0.150461778477206	0.012	0.008	0.159002777950656	no change
7900	7900	MPZL3	7.29797640292778e-06	-0.221905407	0.084	0.071	0.159416996545555	no change
7901	7901	AGAP9	7.36115193536032e-06	0.228877178882258	0.01	0.006	0.160797002876011	no change
7902	7902	C10orf95	7.38156652676848e-06	0.102857694963888	0.013	0.008	0.161242939210731	no change
7903	7903	AC079922.3	7.58838282136572e-06	-0.620480652	0.031	0.038	0.165760634349913	no change
7904	7904	PIAS3	7.76110175893934e-0	-0.212781889	0.103	0.088	0.169533506822271	no

			6					change
7905	7905	NDFIP1	7.79784140491163e-06	-0.113106439	0.622	0.522	0.17033604764889	no change
7906	7906	FRMD4B	7.80875696822722e-06	-0.255810963	0.132	0.113	0.170574487213955	no change
7907	7907	NRBP2	7.87095513855449e-06	-0.176811391	0.026	0.019	0.171933144046584	no change
7908	7908	TSPAN9	7.99311317974972e-06	-0.200429519	0.046	0.037	0.174601564298453	no change
7909	7909	CCDC57	8.13443643569021e-06	-0.209531344	0.311	0.266	0.177688629501217	no change
7910	7910	DNAJB2	8.15584547824406e-06	-0.203241995	0.231	0.2	0.178156288626763	no change
7911	7911	ARL5B	8.21303014072537e-06	-0.23263321	0.337	0.289	0.179405430394005	no change
7912	7912	TBX19	8.29190671796701e-06	0.158636977891146	0.011	0.007	0.181128410347271	no change
7913	7913	GUCA2A	8.32181715225912e-06	0.688759910676129	0.01	0.006	0.181781773873948	no change
7914	7914	LDLRAD4	8.41830715770182e-06	-0.221770505	0.17	0.147	0.183889501552838	no change
7915	7915	CCDC85B	8.45695454047783e-06	-0.446121005	0.395	0.37	0.184733714982198	no change
7916	7916	TRAM2	8.47731523475187e-06	-0.306618248	0.022	0.016	0.18517847398792	no change
7917	7917	LINC00346	8.74470742395853e-0	-0.816816206	0.017	0.024	0.19101938896895	no

			6					change
7918	7918	TNS2	8.83126138094974e-06	-0.15770282	0.021	0.015	0.192910073605466	no change
7919	7919	CTA-29F11.1	8.88126123329139e-06	-0.28021965	0.162	0.14	0.194002270380017	no change
7920	7920	RP11-596C23.2	8.89165964468709e-06	-0.697518451	0.052	0.062	0.194229413278545	no change
7921	7921	PELI3	8.95025275512214e-06	0.14957243592448	0.017	0.012	0.195509321182888	no change
7922	7922	CREB3L1	9.00109798191231e-06	-1.154873327	0.017	0.023	0.196619984316893	down
7923	7923	RAD51B	9.02837470698622e-06	-0.162496347	0.04	0.031	0.197215817099407	no change
7924	7924	KLRC1	9.08741148703069e-06	0.152160796756792	0.011	0.007	0.198505416522698	no change
7925	7925	ANTXR1	9.1614113640573e-06	-0.61230496	0.08	0.066	0.200121869836468	no change
7926	7926	GNE	9.16693314120166e-06	-0.184042406	0.024	0.017	0.200242487536409	no change
7927	7927	HECTD2	9.35174040201763e-06	-0.144115117	0.018	0.013	0.204279417341673	no change
7928	7928	CCDC88B	9.55226334142791e-06	-0.23137264	0.124	0.106	0.208659640430151	no change
7929	7929	AC009410.1	9.56065075849446e-06	-0.225674316	0.022	0.016	0.208842855168553	no change
7930	7930	ADGRF4	9.6310438175875e-06	-0.185140182	0.111	0.095	0.210380521151381	no

								change
7931	7931	TRHDE	9.93388005141365e-06	-0.237470191	0.032	0.025	0.21699567584308	no change
7932	7932	GJB5	9.97645576169491e-06	-0.174776148	0.159	0.138	0.217925699658464	no change
7933	7933	MAP3K12	1.00496584631953e-05	-0.20360791	0.028	0.021	0.219524739470038	no change
7934	7934	WNT4	1.01669203193946e-05	0.159209400710163	0.012	0.007	0.222086207456855	no change
7935	7935	CTB-55O6.8	1.01717556036388e-05	-0.130412814	0.046	0.037	0.222191829405885	no change
7936	7936	PXDN	1.02900480444821e-05	-1.354642662	0.006	0.01	0.224775809483667	down
7937	7937	MB21D2	1.03194526827085e-05	-0.17187522	0.052	0.043	0.225418124401084	no change
7938	7938	RP11-386I14.4	1.03532395137004e-05	-0.724168012	0.061	0.071	0.226156163937271	no change
7939	7939	RNF123	1.04624808099164e-05	-0.1348774	0.042	0.033	0.228542430811813	no change
7940	7940	RP4-550H1.7	1.04762715582449e-05	-0.89881377	0.008	0.013	0.228843675918302	no change
7941	7941	B3GNT2	1.05418631396851e-05	-0.225292796	0.19	0.165	0.230276458423281	no change
7942	7942	SLC29A3	1.0558373229082e-05	-0.123987365	0.1	0.085	0.230637104816067	no change
7943	7943	METTL18	1.06367785657486e-0	-0.178750956	0.042	0.033	0.232349790990211	no

			5					change
7944	7944	RP13-270P17.3	1.06801703919826e-05	-0.510466327	0.039	0.047	0.233297642042467	no change
7945	7945	DUOXA2	1.07392023006877e-05	-0.566154303	0.041	0.033	0.234587135056223	no change
7946	7946	NAV2	1.07632034416052e-05	-0.219377867	0.031	0.024	0.235111415978425	no change
7947	7947	IRF5	1.08358686680225e-05	-0.189434456	0.043	0.034	0.236698715184283	no change
7948	7948	SLC35B1	1.08452043224824e-05	-0.168181998	0.266	0.231	0.236902643220306	no change
7949	7949	ZNF549	1.09589968077236e-05	0.254970353812134	0.01	0.006	0.239388326267915	no change
7950	7950	A4GALT	1.0977291159196e-05	-0.18655869	0.16	0.139	0.239787948081478	no change
7951	7951	KIFC3	1.10751677955439e-05	-0.150959016	0.052	0.042	0.241925965325862	no change
7952	7952	SERTAD3	1.12973074401557e-05	-0.429562526	0.346	0.332	0.246778383722761	no change
7953	7953	PIP4K2A	1.14333998812987e-05	-0.339803205	0.063	0.052	0.249751187007089	no change
7954	7954	RP11-422P24.11	1.15016834154448e-05	0.260397131663142	0.012	0.007	0.251242772526976	no change
7955	7955	HEXA	1.16846457218585e-05	-0.303904452	0.27	0.23	0.255239401148277	no change
7956	7956	KPNA7	1.17064492619653e-0	-0.818036578	0.006	0.01	0.25571567767837	no

			5					change
7957	7957	ZNF267	1.17312374364671e-05	-0.234463926	0.072	0.061	0.256257150562187	no change
7958	7958	CCDC151	1.17705478889796e-05	-0.724905586	0.013	0.018	0.25711584808687	no change
7959	7959	ZNF860	1.17709541587932e-05	0.255423494501171	0.011	0.007	0.257124722644679	no change
7960	7960	ENGASE	1.17784852559634e-05	-0.239515662	0.087	0.074	0.257289231931266	no change
7961	7961	KRBA1	1.1786622804543e-05	0.121397642676624	0.02	0.014	0.257466988542438	no change
7962	7962	PPP3CA	1.17907690818502e-05	-0.383588167	0.361	0.308	0.257557559823935	no change
7963	7963	PANX2	1.18228438969787e-05	0.165855347074066	0.03	0.023	0.258258202085602	no change
7964	7964	HBB	1.18256416758122e-05	-0.447406534	0.024	0.018	0.258319316766441	no change
7965	7965	GLRX	1.18681526337738e-05	-0.593268492	0.423	0.39	0.259247926132155	no change
7966	7966	SLC7A6	1.20317404955582e-05	-0.294748638	0.022	0.016	0.262821339384973	no change
7967	7967	SAPCD2	1.22683594504493e-05	0.24821643842167	0.021	0.015	0.267990043835615	no change
7968	7968	TATDN3	1.23336703369847e-05	-0.245848845	0.079	0.066	0.269416694841094	no change
7969	7969	CARD6	1.24420877146682e-0	-0.146715407	0.062	0.051	0.271784964039211	no

			5					change
7970	7970	UQCR10	1.26306987777849e-05	0.217564780824354	0.966	0.911	0.275904984101934	no change
7971	7971	ZNF175	1.28657233676139e-05	-0.477326325	0.021	0.028	0.281038861242157	no change
7972	7972	THBS1	1.31313540736317e-05	-0.737586873	0.233	0.233	0.286841298384411	no change
7973	7973	SMIM2-AS1	1.31721506425985e-05	-0.132068432	0.034	0.026	0.287732458636921	no change
7974	7974	WWOX	1.36604118054199e-05	-0.244951281	0.058	0.048	0.298398035477593	no change
7975	7975	DENND6B	1.37379131032959e-05	-0.207073462	0.03	0.023	0.300090973828396	no change
7976	7976	TRIM54	1.37771070905862e-05	-0.169846819	0.014	0.009	0.300947127286765	no change
7977	7977	RP11-395N3.2	1.37824268147245e-05	0.35922971459594	0.01	0.006	0.301063331340843	no change
7978	7978	TMEM19	1.38071933433505e-05	-0.399539279	0.317	0.272	0.301604331392147	no change
7979	7979	CAPN10	1.38232498658417e-05	-0.169185823	0.038	0.03	0.301955070069447	no change
7980	7980	MST1R	1.39278684203856e-05	-0.150313696	0.055	0.045	0.304240357774902	no change
7981	7981	MSRB1	1.39695917757952e-05	-0.252455736	0.245	0.212	0.305151762750471	no change
7982	7982	NOS1AP	1.40490908819715e-0	0.17817081387729	0.02	0.014	0.306888341225786	no

			5	1				change
7983	7983	C1orf226	1.46755388350337e-05	0.449014013280078	0.011	0.007	0.320572470312476	no change
7984	7984	TEF	1.49207123504851e-05	-0.158309423	0.024	0.018	0.325928040583997	no change
7985	7985	SLC39A8	1.5374475821717e-05	-0.183203208	0.034	0.027	0.335840049849587	no change
7986	7986	FAM212B	1.54815412963526e-05	0.116913042151712	0.015	0.01	0.338178788077527	no change
7987	7987	SUSD6	1.54869235860292e-05	-0.214190162	0.08	0.067	0.338296358813222	no change
7988	7988	RPTOR	1.54928411414943e-05	-0.122677769	0.028	0.021	0.338425621894801	no change
7989	7989	C1orf53	1.56338303619731e-05	-0.203958794	0.155	0.135	0.34150539042694	no change
7990	7990	RPP25	1.58098231525277e-05	-0.493906543	0.119	0.129	0.345349776943814	no change
7991	7991	RP11-347I19.7	1.58759798296983e-05	-0.649016431	0.013	0.018	0.346794903399929	no change
7992	7992	AC142472.6	1.60127686836082e-05	0.160992796323978	0.024	0.018	0.349782919124737	no change
7993	7993	AKR1B10	1.61320866685013e-05	-0.50806487	0.213	0.218	0.352389301186743	no change
7994	7994	NBPF19	1.62295580179154e-05	-0.167665308	0.033	0.026	0.354518465343343	no change
7995	7995	ZNF607	1.63100032126207e-0	0.18349801659833	0.011	0.007	0.356275710176487	no

			5	7				change
7996	7996	SPINK4	1.64103641004673e-05	-0.453879418	0.047	0.038	0.358467993410609	no change
7997	7997	AZIN1-AS1	1.6540998855479e-05	-0.532804269	0.038	0.047	0.361321578999082	no change
7998	7998	SNCA	1.68320257706086e-05	-0.454422791	0.055	0.064	0.367678770933175	no change
7999	7999	CCDC181	1.74436031257213e-05	-0.836243673	0.026	0.033	0.381038066678256	no change
8000	8000	HNF1B	1.7484754375469e-05	-0.331416104	0.08	0.067	0.381936974577746	no change
8001	8001	LINC00162	1.75799567433183e-05	-0.621890903	0.008	0.012	0.384016575101045	no change
8002	8002	CAPN10-AS1	1.78863978269609e-05	-0.123837772	0.035	0.027	0.390710474132134	no change
8003	8003	CTD-2325M2.1	1.83239316269117e-05	0.22954435646539	0.01	0.007	0.400267962458259	no change
8004	8004	DIO3OS	1.86365739645302e-05	-1.362988484	0.01	0.015	0.407097321681198	down
8005	8005	MTRNR2L12	1.8764856292675e-05	-0.312851512	0.14	0.12	0.409899520857192	no change
8006	8006	TSPYL2	1.87803431766042e-05	-0.240694804	0.19	0.164	0.410237816349743	no change
8007	8007	TMED3	1.884972480918e-05	-0.106331817	0.567	0.475	0.411753388731728	no change
8008	8008	PFKFB3	1.88669456986118e-0	-0.587093228	0.158	0.165	0.412129561840477	no

			5					change
8009	8009	C14orf1	1.90124681212595e-05	-0.327958841	0.454	0.412	0.415308353640792	no change
8010	8010	FAM127A	1.90264890657263e-05	-0.222229987	0.327	0.279	0.415614627151726	no change
8011	8011	RARA	1.90680825711595e-05	-0.297553332	0.105	0.089	0.416523195684409	no change
8012	8012	ZNF513	1.91310849300959e-05	-0.17245278	0.071	0.06	0.417899419213014	no change
8013	8013	SCG5	1.92592137461122e-05	-1.103298666	0.011	0.016	0.420698265070075	down
8014	8014	SC5D	1.93666515291673e-05	-0.453468303	0.243	0.244	0.423045136003131	no change
8015	8015	C1orf116	1.94961648985471e-05	-0.597029347	0.161	0.167	0.425874226043864	no change
8016	8016	ZHX2	1.96219800175029e-05	-0.182202014	0.027	0.021	0.428622531502333	no change
8017	8017	TMEM175	1.97372630439923e-05	-0.271261514	0.085	0.072	0.431140773932967	no change
8018	8018	BIK	1.98822081980136e-05	-0.236365742	0.215	0.186	0.43430695587741	no change
8019	8019	KDELR3	2.0226831100086e-05	-0.728402697	0.078	0.087	0.44183489855028	no change
8020	8020	LHFPL4	2.03159615946431e-05	-0.112352762	0.016	0.011	0.443781865073385	no change
8021	8021	ZSCAN16-AS1	2.03865641757366e-0	-0.313196894	0.119	0.102	0.445324107854791	no

			5					change
8022	8022	ZKSCAN5	2.04830442159151e-05	-0.160169782	0.024	0.018	0.44743161785245	no change
8023	8023	RP3-467N11.1	2.05017418795674e-05	0.111429809708173	0.014	0.01	0.447840049617271	no change
8024	8024	FGF2	2.06762258754487e-05	-1.091883015	0.012	0.017	0.4516514780233	down
8025	8025	RAB17	2.07424652387868e-05	-0.557513631	0.077	0.087	0.45309841067606	no change
8026	8026	FPR3	2.07561232587529e-05	-1.034498299	0.008	0.012	0.453396756464199	down
8027	8027	CGRRF1	2.08188318606645e-05	-0.202734699	0.115	0.099	0.454766563164355	no change
8028	8028	P3H3	2.08519167929467e-05	-1.250919405	0.015	0.02	0.455489270425128	down
8029	8029	TMEM141	2.10323678304664e-05	-0.436258096	0.507	0.447	0.459431042888707	no change
8030	8030	LMO2	2.111924173178e-05	-0.109244984	0.024	0.018	0.461328716389003	no change
8031	8031	MAGEA9	2.11222973609296e-05	-0.548012903	0.007	0.011	0.461395463552146	no change
8032	8032	RUNX3	2.13258546004812e-05	0.318698241778772	0.011	0.007	0.465841967892911	no change
8033	8033	GRINA	2.14486531465789e-05	-0.226860507	0.326	0.279	0.46852437933387	no change
8034	8034	GPAT2	2.16352960518539e-0	0.24525396343981	0.01	0.006	0.472601406956697	no

			5	4				change
8035	8035	FZD5	2.17351626700784e-05	-0.175277046	0.045	0.036	0.474782893365193	no change
8036	8036	C1orf54	2.18690340219567e-05	-0.900796127	0.032	0.039	0.477707179175623	no change
8037	8037	C3orf62	2.19077826593461e-05	-0.121973547	0.048	0.039	0.478553604410756	no change
8038	8038	HCAR3	2.21012889082555e-05	-0.571002976	0.072	0.082	0.482780554911932	no change
8039	8039	MB21D1	2.21321126574398e-05	-0.246274514	0.036	0.028	0.483453868889115	no change
8040	8040	UGT2A3	2.22647464174584e-05	-0.942341402	0.008	0.012	0.486351120742962	no change
8041	8041	ADAMTS7	2.24018102228276e-05	0.119769818267719	0.019	0.014	0.489345142507446	no change
8042	8042	MEGF6	2.26139169870105e-05	-0.402383237	0.021	0.016	0.493978402664258	no change
8043	8043	ENTPD2	2.2730124733118e-05	-0.766874883	0.009	0.014	0.49651684467023	no change
8044	8044	CLIP2	2.27569752404818e-05	-0.256589465	0.018	0.013	0.497103367153085	no change
8045	8045	TGFBI	2.28134600210005e-05	-0.291225448	0.069	0.057	0.498337220698734	no change
8046	8046	RAVER1	2.29598379770761e-05	0.101047617197025	0.011	0.007	0.501534700771251	no change
8047	8047	RGS3	2.3702617611041e-05	-0.29886997	0.046	0.037	0.517759979095579	no

								change
8048	8048	SEMA3F	2.40779340621384e-05	-0.333123784	0.097	0.083	0.525958391653351	no change
8049	8049	FLCN	2.42831788735223e-05	-0.272427732	0.111	0.095	0.530441759313222	no change
8050	8050	PDE3B	2.44053223932021e-05	-0.210998798	0.013	0.009	0.533109862357107	no change
8051	8051	ITPRIP	2.45864290737112e-05	-0.180952333	0.026	0.019	0.537065956686147	no change
8052	8052	CCDC7	2.4867891142398e-05	-0.173744189	0.016	0.011	0.543214214114541	no change
8053	8053	FUT9	2.50925824657951e-05	0.159043897188704	0.025	0.019	0.548122371382828	no change
8054	8054	SLC27A5	2.52699850024541e-05	-0.230787023	0.127	0.11	0.551997552393607	no change
8055	8055	CYP7B1	2.55352900038666e-05	-0.218548469	0.015	0.01	0.557792874844462	no change
8056	8056	RP11-712L6.5	2.57200252279624e-05	0.128653427857699	0.013	0.009	0.561828231079611	no change
8057	8057	MSANTD2	2.62617124189029e-05	-0.11424781	0.024	0.018	0.573660846078516	no change
8058	8058	S1PR3	2.64994710639288e-05	-0.416083634	0.014	0.009	0.578854445920462	no change
8059	8059	FIZ1	2.71887381723599e-05	-0.109188792	0.022	0.017	0.59391079663703	no change
8060	8060	HOPX	2.71989939280738e-0	-0.815028764	0.16	0.168	0.594134823364844	no

			5					change
8061	8061	CHIC2	2.78085295009543e-05	-0.30107288	0.331	0.282	0.607449518418845	no change
8062	8062	CCL3L3	2.82891193755112e-05	-2.217772062	0.04	0.048	0.617947523638666	down
8063	8063	RP4-569M23.2	2.84218638293893e-05	0.132215106996408	0.01	0.006	0.620847193489179	no change
8064	8064	RP11-538P18.2	2.85675328365825e-05	-0.309995635	0.095	0.081	0.624029187282308	no change
8065	8065	LSP1	2.86165556693321e-05	-0.264285454	0.14	0.121	0.625100042040891	no change
8066	8066	TPK1	2.89827985534697e-05	-0.21594786	0.045	0.036	0.633100251601992	no change
8067	8067	KBTBD11	2.93462261547007e-05	-0.112594614	0.012	0.008	0.641038964123281	no change
8068	8068	SWSAP1	2.93592539123405e-05	-0.174601763	0.086	0.073	0.641323542461165	no change
8069	8069	SAMHD1	3.05518793653282e-05	-0.796686368	0.05	0.058	0.66737525285623	no change
8070	8070	GSDMB	3.10342040041071e-05	-0.285529452	0.13	0.111	0.677911152265716	no change
8071	8071	PLEKHH3	3.10858003403104e-05	-0.219238749	0.168	0.146	0.679038222633741	no change
8072	8072	RNF215	3.18205513423879e-05	0.180588149657164	0.015	0.01	0.69508812352312	no change
8073	8073	AURKC	3.21562492243364e-0	-0.561294267	0.012	0.017	0.702421108056404	no

			5					change
8074	8074	TMEM254	3.22435063382108e-05	-0.162092563	0.116	0.101	0.704327152451878	no change
8075	8075	ESPN	3.24683339210071e-05	-0.736292822	0.011	0.015	0.709238286170479	no change
8076	8076	GIPR	3.29986182659266e-05	-0.164192755	0.018	0.013	0.720821817400902	no change
8077	8077	CTD-2331H12.7	3.37530211854672e-05	0.291940981015181	0.01	0.006	0.737300994775345	no change
8078	8078	SLURP1	3.39052432622088e-05	-0.102526348	0.027	0.021	0.740626133819689	no change
8079	8079	CHN2	3.3952293676212e-05	-0.560907517	0.04	0.048	0.741653903063176	no change
8080	8080	RP11-626G11.3	3.41381196346365e-05	-0.274637517	0.016	0.011	0.745713085	no change
8081	8081	GCH1	3.45931361847353e-05	-0.265545703	0.155	0.135	0.755652466819358	no change
8082	8082	FASN	3.48126754408464e-05	-0.157376459	0.198	0.175	0.760448082329848	no change
8083	8083	IL6R	3.48189549945941e-05	-0.140432971	0.033	0.026	0.760585252901913	no change
8084	8084	STX3	3.54963286431568e-05	-0.167886152	0.214	0.189	0.775381802881116	no change
8085	8085	DYNLRB2	3.55694498669194e-05	-0.655367402	0.017	0.022	0.776979062892988	no change
8086	8086	PIGT	3.60143154264964e-0	-0.333719355	0.348	0.294	0.786696706176388	no

			5					change
8087	8087	RDH13	3.60947251668103e-05	-0.269829286	0.141	0.122	0.788453176543805	no change
8088	8088	TTLL4	3.60988436081619e-05	-0.156190665	0.021	0.015	0.78854313977669	no change
8089	8089	MSX1	3.61622554383685e-05	-1.087690276	0.022	0.028	0.789928307795721	down
8090	8090	KSR2	3.64896198430237e-05	-0.336224585	0.124	0.108	0.79707925585101	no change
8091	8091	RGS5	3.67289261445681e-05	-0.242996182	0.028	0.022	0.802306662701946	no change
8092	8092	ZNF780A	3.6902402453532e-05	-0.203388135	0.072	0.061	0.806096079194952	no change
8093	8093	TPCN2	3.76887162548926e-05	-0.656058719	0.05	0.058	0.823272317871874	no change
8094	8094	RTKN2	3.79059666040717e-05	-0.103422773	0.045	0.037	0.828017934499341	no change
8095	8095	CXCL16	3.79213152928066e-05	-0.241671004	0.23	0.201	0.828353211256067	no change
8096	8096	FAM180A	3.80088213171214e-05	-1.335863817	0.006	0.01	0.830264692851199	down
8097	8097	MFAP2	3.87403792244837e-05	-0.429694194	0.05	0.041	0.846244843779621	no change
8098	8098	FAM43A	3.90218409862036e-05	-1.023507145	0.021	0.027	0.852393094502631	down
8099	8099	EMC9	3.92350093404142e-0	-0.169053892	0.125	0.109	0.857049544032007	no

			5					change
8100	8100	CNGA1	3.96612540024059e-05	-0.30335982	0.148	0.128	0.866360432428554	no change
8101	8101	GALT	3.98368624182118e-05	-0.17724458	0.066	0.055	0.870196422663418	no change
8102	8102	CCDC51	4.00854303180001e-05	-0.215881513	0.079	0.067	0.875626139866394	no change
8103	8103	MSR1	4.0094472852296e-05	-1.119492962	0.016	0.021	0.875823664985554	down
8104	8104	INTS12	4.03346750280143e-05	-0.194136151	0.069	0.058	0.881070641311944	no change
8105	8105	LTC4S	4.03504402737582e-05	-0.647719521	0.049	0.04	0.881415017339975	no change
8106	8106	PARD6G-AS1	4.04026626074033e-05	-0.745943388	0.029	0.035	0.882555761996117	no change
8107	8107	RP11-156K23.3	4.06730690537408e-05	-0.123463566	0.03	0.023	0.888462520409913	no change
8108	8108	SH3BP4	4.08369372582723e-05	-0.24187713	0.033	0.026	0.892042057469699	no change
8109	8109	MAPK3	4.09004380823177e-05	-0.251335189	0.115	0.1	0.893429169470147	no change
8110	8110	ETFDH	4.09115038536762e-05	-0.249824636	0.11	0.095	0.893670890179704	no change
8111	8111	ACACB	4.13055115168015e-05	-0.239956102	0.019	0.014	0.902277593573013	no change
8112	8112	NOVA1	4.13600564309034e-05	-1.295879834	0.012	0.017	0.903469072676655	down

8113	8113	TMEM254-AS1	4.14073708385589e-05	0.261788038640817	0.015	0.011	0.904502608597481	no change
8114	8114	OSGIN1	4.15150795760481e-05	-0.959759106	0.031	0.038	0.906855398259195	no change
8115	8115	GABRE	4.24067759223728e-05	-0.225714851	0.085	0.072	0.926333613248311	no change
8116	8116	C2orf27B	4.27246367425827e-05	0.192675136891762	0.01	0.007	0.933276965004977	no change
8117	8117	CYP4B1	4.27730198074974e-05	-0.187643833	0.651	0.551	0.934333844674974	no change
8118	8118	ZNF365	4.28357270666097e-05	-0.806313822	0.009	0.013	0.935703622043022	no change
8119	8119	MAGEA6	4.30348114390538e-05	-0.445893787	0.021	0.027	0.940052421074692	no change
8120	8120	ZNF554	4.33953636447148e-05	0.118914055345632	0.014	0.01	0.947928323455149	no change
8121	8121	SIDT2	4.4845745807621e-05	-0.180659153	0.029	0.023	0.979610471421672	no change
8122	8122	COL8A1	4.53302657397052e-05	-1.493675857	0.007	0.01	0.99019432481812	down
8123	8123	C10orf11	4.55622517740301e-05	-0.199189286	0.242	0.21	0.995261827751913	no change
8124	8124	SLC35C1	4.61213618281351e-05	-0.212651994	0.091	0.078	1	no change
8125	8125	TRIP6	4.62014984255551e-05	-0.190576797	0.426	0.36	1	no change

8126	8126	CAPN9	4.62053796381898e-05	-0.503995703	0.017	0.022	1	no change
8127	8127	SNHG12	4.65045243119739e-05	-0.280739994	0.151	0.132	1	no change
8128	8128	RNASEK	4.65201533680775e-05	-0.212807638	0.066	0.056	1	no change
8129	8129	ROM1	4.69442955580206e-05	-0.142949024	0.027	0.021	1	no change
8130	8130	RIN3	4.69957820089024e-05	-0.224996407	0.027	0.02	1	no change
8131	8131	SCPEP1	4.72885510300573e-05	-0.344814036	0.445	0.413	1	no change
8132	8132	FGFBP3	4.80476347572946e-05	-0.156361015	0.033	0.026	1	no change
8133	8133	ATG4A	4.81147638747812e-05	-0.157883889	0.067	0.056	1	no change
8134	8134	XXbac-BPG181B23.7	4.83489964688956e-05	0.283253920341708	0.013	0.009	1	no change
8135	8135	CTD-2515O10.5	4.84867559063892e-05	-0.825576335	0.008	0.012	1	no change
8136	8136	PPP1R3F	4.93000597156809e-05	0.212920476366911	0.012	0.008	1	no change
8137	8137	TTLL7	4.93570653863474e-05	-0.180832797	0.051	0.042	1	no change
8138	8138	PLIN5	4.94204021452876e-05	-0.202671089	0.116	0.101	1	no change

8139	8139	IER3IP1	4.95295296172442e-05	-0.147505761	0.565	0.467	1	no change
8140	8140	PDE4B	4.98603146242578e-05	-1.007312585	0.014	0.019	1	down
8141	8141	NEDD4	4.98985212309448e-05	-0.206825183	0.033	0.026	1	no change
8142	8142	C19orf60	4.99079854358047e-05	-0.130942093	0.632	0.527	1	no change
8143	8143	AC144652.1	5.01030020110815e-05	-1.030249738	0.011	0.016	1	down
8144	8144	ATP6V1D	5.02280036338063e-05	-0.290092996	0.436	0.402	1	no change
8145	8145	MACROD1	5.02903357163976e-05	-0.202875382	0.215	0.187	1	no change
8146	8146	C17orf67	5.04438859592685e-05	-0.105602822	0.04	0.032	1	no change
8147	8147	ACADSB	5.05588772180777e-05	-0.119559043	0.068	0.057	1	no change
8148	8148	CABYR	5.14509980531177e-05	-0.120850787	0.045	0.037	1	no change
8149	8149	STK3	5.18588409145233e-05	-0.250267587	0.129	0.112	1	no change
8150	8150	ABHD5	5.24123138617942e-05	-0.543719909	0.247	0.244	1	no change
8151	8151	RP11-47A8.5	5.25184096924709e-05	-0.995048381	0.015	0.02	1	no change

8152	8152	NPDC1	5.25499336607781e-05	-0.292928354	0.268	0.231	1	no change
8153	8153	RP11-488C13.5	5.2621593451792e-05	0.11003662163666	0.012	0.008	1	no change
8154	8154	FAM134B	5.29257367868532e-05	-0.211670274	0.032	0.025	1	no change
8155	8155	SLC9A9	5.32138775868511e-05	-0.203650458	0.025	0.019	1	no change
8156	8156	PLK2	5.36387253137336e-05	-0.670462358	0.136	0.143	1	no change
8157	8157	IFI44	5.38434870175617e-05	-0.222915375	0.205	0.179	1	no change
8158	8158	SLC2A1-AS1	5.41765644341337e-05	-0.568840287	0.027	0.033	1	no change
8159	8159	LINC01504	5.43534419347087e-05	-0.163098799	0.029	0.022	1	no change
8160	8160	TRAF3IP2	5.43730431489167e-05	-0.21903085	0.077	0.066	1	no change
8161	8161	FMO5	5.44410114448704e-05	-0.531363933	0.023	0.029	1	no change
8162	8162	SPPL2A	5.50319070302798e-05	-0.326281022	0.315	0.268	1	no change
8163	8163	COMMD1	5.51735731667157e-05	-0.227931089	0.321	0.275	1	no change
8164	8164	DKK1	5.53558437283855e-05	-0.625308741	0.283	0.284	1	no change

8165	8165	RP11-452F19.3	5.54160435436572e-05	-0.199471949	0.086	0.073	1	no change
8166	8166	CLDN23	5.55127915712163e-05	-0.273487655	0.273	0.236	1	no change
8167	8167	RP11-707G18.1	5.560475682784e-05	-0.855223053	0.014	0.019	1	no change
8168	8168	SGK223	5.57215316852664e-05	-0.250269299	0.022	0.017	1	no change
8169	8169	MAP7D1	5.59179767047849e-05	-0.463785663	0.286	0.279	1	no change
8170	8170	ZDHHC1	5.6198704025183e-05	-0.841019026	0.025	0.031	1	no change
8171	8171	GYLTL1B	5.62072100233964e-05	-0.179777787	0.033	0.026	1	no change
8172	8172	DNM1	5.68169111892361e-05	-1.575186603	0.006	0.01	1	down
8173	8173	AATK	5.70912535969373e-05	-0.833790424	0.01	0.015	1	no change
8174	8174	EPB41L2	5.78015486631761e-05	-0.638069849	0.064	0.053	1	no change
8175	8175	RDH16	5.79968968943433e-05	-0.678064508	0.015	0.02	1	no change
8176	8176	DUSP3	5.84481667171985e-05	-0.249955291	0.138	0.12	1	no change
8177	8177	DEDD	6.05263738599234e-05	-0.228726745	0.124	0.108	1	no change

8178	8178	DHFRL1	6.08185979474652e-05	-0.168447688	0.021	0.015	1	no change
8179	8179	PLAC1	6.12566392953122e-05	-0.788058644	0.007	0.01	1	no change
8180	8180	TSPAN12	6.38917541359103e-05	-0.204750924	0.189	0.166	1	no change
8181	8181	RIPK3	6.398706367704e-05	-0.211767067	0.025	0.019	1	no change
8182	8182	CTD-3252C9.4	6.44473165913749e-05	-0.35132127	0.264	0.228	1	no change
8183	8183	CCL4	6.4471340193075e-05	-1.74486258	0.06	0.068	1	down
8184	8184	FBXO17	6.5509306973571e-05	-1.210138014	0.016	0.021	1	down
8185	8185	CCDC64B	6.56250304474312e-05	-0.230003151	0.32	0.276	1	no change
8186	8186	TCF25	6.57533750622031e-05	-0.114853081	0.634	0.532	1	no change
8187	8187	LINC00900	6.58623574310003e-05	0.243514965835204	0.017	0.012	1	no change
8188	8188	WNT5B	6.58665109626216e-05	-0.29148283	0.01	0.006	1	no change
8189	8189	AP000769.1	6.6310416743252e-05	-0.211879392	0.075	0.064	1	no change
8190	8190	TYROBP	6.73429510745593e-05	-1.118044997	0.067	0.076	1	down
8191	8191	BATF2	6.75702773436534e-05	-0.926807448	0.007	0.011	1	no change

8192	8192	FLJ21408	6.8122858301442e-05	0.178386183373081	0.01	0.007	1	no change
8193	8193	RAB30	6.86437462322188e-05	-0.11566016	0.023	0.017	1	no change
8194	8194	BMP5	6.86749854317282e-05	-1.108907809	0.007	0.011	1	down
8195	8195	TM4SF1-AS1	6.88634164850503e-05	-0.557301159	0.015	0.02	1	no change
8196	8196	LITAF	6.95057791314815e-05	-0.229047369	0.672	0.598	1	no change
8197	8197	RAB4B	7.01193334376499e-05	-0.227584556	0.171	0.15	1	no change
8198	8198	RP3-512B11.3	7.12610320293469e-05	-0.310146635	0.06	0.05	1	no change
8199	8199	LPP-AS2	7.25586692173654e-05	-0.113478499	0.024	0.018	1	no change
8200	8200	UBXN10-AS1	7.31341267713406e-05	-0.721232859	0.016	0.021	1	no change
8201	8201	CDK5	7.34129899349041e-05	-0.240605131	0.151	0.132	1	no change
8202	8202	WWP2	7.34567505826948e-05	-0.175989717	0.077	0.066	1	no change
8203	8203	ZNF165	7.37938694751618e-05	-0.514554275	0.121	0.13	1	no change
8204	8204	MS4A6A	7.43698205942957e-05	-1.201629872	0.022	0.027	1	down

8205	8205	AGBL3	7.44818728444153e-05	0.142504146107287	0.013	0.009		1	no change
8206	8206	ARHGDIA	7.45268252927159e-05	-0.104633909	0.598	0.507		1	no change
8207	8207	CERS4	7.46268268612427e-05	-0.599102049	0.046	0.054		1	no change
8208	8208	TTN-AS1	7.48640127063602e-05	-0.332910116	0.058	0.048		1	no change
8209	8209	C1QTNF3	7.50625000266442e-05	-1.429220564	0.016	0.021		1	down
8210	8210	C4orf47	7.53747598896585e-05	-0.642247125	0.009	0.013		1	no change
8211	8211	IGFBP4	7.74412620331655e-05	-0.352304416	0.516	0.47		1	no change
8212	8212	C3orf52	7.78509416666229e-05	-0.221326718	0.181	0.158		1	no change
8213	8213	N6AMT1	7.97011481140911e-05	-0.21856471	0.08	0.068		1	no change
8214	8214	ESRP2	8.0087102815064e-05	-0.182053812	0.088	0.076		1	no change
8215	8215	TIGD2	8.08565432870221e-05	0.121627988832238	0.035	0.028		1	no change
8216	8216	VPS50	8.08877328454465e-05	-0.143206264	0.04	0.033		1	no change
8217	8217	SUN1	8.1458846533353e-05	-0.220236029	0.279	0.244		1	no change

8218	8218	COLEC12	8.1528122000649e-05	-1.095421101	0.014	0.018		1	down
8219	8219	KTN1	8.17480277386781e-05	-0.116001081	0.905	0.821		1	no change
8220	8220	SYNJ1	8.18795827523626e-05	-0.20568579	0.025	0.019		1	no change
8221	8221	UGDH-AS1	8.18937609415911e-05	0.161220361261265	0.01	0.006		1	no change
8222	8222	FOXP1	8.2456865176649e-05	-0.177766617	0.441	0.38		1	no change
8223	8223	ZNF614	8.26449499270063e-05	-0.100818308	0.028	0.022		1	no change
8224	8224	AP4S1	8.49700922685508e-05	-0.153436232	0.03	0.024		1	no change
8225	8225	RP11-46C24.7	8.63265980392259e-05	0.103170662835811	0.012	0.008		1	no change
8226	8226	CASC2	8.73679892056069e-05	0.178463868740507	0.012	0.008		1	no change
8227	8227	TMEM45B	8.85761425129706e-05	-0.202103033	0.218	0.191		1	no change
8228	8228	RHBDF1	9.01730544301734e-05	-0.596559767	0.057	0.066		1	no change
8229	8229	TRA2A	9.04848007046971e-05	-0.351311624	0.53	0.452		1	no change
8230	8230	FRS3	9.10502317760108e-05	-0.149140605	0.01	0.007		1	no change
8231	8231	SPIRE1	9.16999112194996e-0	-0.110873529	0.056	0.047		1	no

			5					change
8232	8232	NLRP2	9.25509507488567e-05	-0.420565138	0.034	0.041	1	no change
8233	8233	ZNF117	9.27127170444794e-05	-0.299525995	0.144	0.127	1	no change
8234	8234	PEPD	9.54011188741204e-05	-0.215026093	0.319	0.274	1	no change
8235	8235	ANO8	9.6417651477942e-05	-0.663535466	0.008	0.012	1	no change
8236	8236	LINC00936	9.66383475709124e-05	-0.699039101	0.07	0.078	1	no change
8237	8237	CCDC28B	9.70787583768e-05	-0.286601448	0.066	0.056	1	no change
8238	8238	MKL1	9.86132319244667e-05	-0.293047476	0.048	0.039	1	no change
8239	8239	RFTN1	9.87352173683212e-05	-0.315018268	0.031	0.025	1	no change
8240	8240	ZNF764	9.90677634826581e-05	-0.103719493	0.017	0.013	1	no change
8241	8241	TP53INP2	9.94759424799623e-05	-0.686497498	0.064	0.072	1	no change
8242	8242	ZNF532	0.000101025960579114	-0.424028397	0.039	0.032	1	no change
8243	8243	SRR	0.000101916212664069	-0.116917462	0.015	0.011	1	no change
8244	8244	C7orf49	0.00010280871484089	-0.230806662	0.217	0.19	1	no

			6					change
8245	8245	SF3A2	0.00010354808992123 9	-0.252694792	0.228	0.198	1	no change
8246	8246	SLC39A14	0.0001043634467549	-0.51310947	0.043	0.035	1	no change
8247	8247	RIT1	0.00010489060822100 9	-0.272839014	0.273	0.238	1	no change
8248	8248	NDUFA3	0.00010528207936742 1	-0.138600285	0.789	0.697	1	no change
8249	8249	FAM216A	0.00010601055656425 1	-0.342502813	0.015	0.011	1	no change
8250	8250	IL15	0.00010623828557408 5	-0.123164903	0.027	0.021	1	no change
8251	8251	SESN1	0.00010706212817053 2	-0.358933673	0.074	0.063	1	no change
8252	8252	DDAH2	0.00010749449290817	-0.491338623	0.201	0.172	1	no change
8253	8253	RDH11	0.00010794662026885 9	-0.250263452	0.283	0.246	1	no change
8254	8254	ACVR1	0.00010871489775884 3	-0.256308848	0.077	0.066	1	no change
8255	8255	SYS1	0.00011045838485219 2	-0.249899528	0.162	0.142	1	no change
8256	8256	SGCB	0.00011438294204199 1	-0.428428877	0.066	0.056	1	no change
8257	8257	PTPRE	0.00011520762475953	-0.201758388	0.078	0.067	1	no

			2					change
8258	8258	EGLN2	0.000116495037498671	-0.241170305	0.199	0.175	1	no change
8259	8259	APBB3	0.000116656018779035	-0.14468188	0.03	0.024	1	no change
8260	8260	RP3-323A16.1	0.000120317084513857	-0.176667972	0.021	0.016	1	no change
8261	8261	ACSS2	0.000120648494697653	-0.54311053	0.071	0.079	1	no change
8262	8262	PTPN20	0.000121335663306781	0.101608131547771	0.039	0.032	1	no change
8263	8263	ADGRL2	0.000121547502701324	-0.991943176	0.012	0.016	1	no change
8264	8264	PLBD2	0.000121857938843818	-0.419035866	0.057	0.048	1	no change
8265	8265	RP11-306G20.1	0.000122363655334743	-0.163803446	0.01	0.006	1	no change
8266	8266	CXCL10	0.000123018755936518	-0.975161097	0.017	0.022	1	no change
8267	8267	PNPLA3	0.000124033866883444	-0.579533686	0.008	0.012	1	no change
8268	8268	GCHFR	0.000124846133683674	-0.393233966	0.222	0.226	1	no change
8269	8269	RP4-563E14.1	0.000125782409331734	0.256460920480684	0.011	0.008	1	no change
8270	8270	PLAGL1	0.00012626136134358	-1.035725438	0.029	0.035	1	down

			8					
8271	8271	DYNLT3	0.000127410477176813	-0.219917186	0.401	0.341	1	no change
8272	8272	GLIPR1	0.000128071413297802	-0.760296608	0.081	0.089	1	no change
8273	8273	GJB7	0.000129105000791108	0.14999265243844	0.026	0.02	1	no change
8274	8274	GBF1	0.00012939665875714	-0.252566314	0.055	0.046	1	no change
8275	8275	MMP7	0.000130660397433561	-0.419116369	0.189	0.165	1	no change
8276	8276	GZMA	0.000131273824909274	0.459498144276859	0.011	0.007	1	no change
8277	8277	NKIRAS1	0.000133140333328799	-0.164854617	0.056	0.047	1	no change
8278	8278	MOSPD1	0.000133226049761184	-0.159719011	0.147	0.13	1	no change
8279	8279	CCDC71	0.000134982144599966	-0.178062135	0.062	0.052	1	no change
8280	8280	RTTN	0.0001350581185915	0.116616719072294	0.011	0.007	1	no change
8281	8281	TIPARP	0.000135245311495878	-0.15029668	0.413	0.358	1	no change
8282	8282	RASA3	0.000135884188135906	-0.168420295	0.015	0.011	1	no change
8283	8283	VCX3B	0.00013601394323331	-0.938361956	0.018	0.023	1	no

			4					change
8284	8284	SPRY1	0.000136143381773315	-0.430258094	0.148	0.129	1	no change
8285	8285	TAF1A-AS1	0.000136509837204994	-0.10182936	0.023	0.018	1	no change
8286	8286	POLH	0.000136959186994692	-0.116701002	0.022	0.017	1	no change
8287	8287	MUC3A	0.00013828232379585	-0.202325459	0.036	0.029	1	no change
8288	8288	SESN2	0.000139033614376149	-0.567911729	0.085	0.094	1	no change
8289	8289	UBE2D4	0.000139611793660502	-0.247733698	0.164	0.144	1	no change
8290	8290	PREX1	0.000139743923538796	-0.24935031	0.012	0.008	1	no change
8291	8291	PPIP5K1	0.000141976189837949	-0.107738569	0.025	0.019	1	no change
8292	8292	GTF3A	0.000144067765043112	-0.243553385	0.449	0.419	1	no change
8293	8293	PTPN7	0.000144266105088271	0.232927022192514	0.01	0.007	1	no change
8294	8294	ADNP-AS1	0.000148659466774075	0.123182266331316	0.014	0.01	1	no change
8295	8295	SLC10A3	0.000151229927382974	-0.215474555	0.206	0.181	1	no change
8296	8296	SPTY2D1-AS1	0.00015144670241795	0.23654987350631	0.013	0.009	1	no

			6	1				change
8297	8297	ATP11A	0.000151734378112123	-0.262912177	0.02	0.015	1	no change
8298	8298	RAP1A	0.000151951167716129	-0.172967957	0.397	0.34	1	no change
8299	8299	FRS2	0.000152831787334517	-0.341084387	0.15	0.131	1	no change
8300	8300	MFSD4	0.000153624175108313	-0.756997417	0.015	0.02	1	no change
8301	8301	CTC-425F1.4	0.00015453227472302	-0.587523902	0.071	0.079	1	no change
8302	8302	ZNF836	0.000154824275741572	-0.582023178	0.045	0.052	1	no change
8303	8303	SOCS3	0.000156459628804508	-0.543074395	0.421	0.391	1	no change
8304	8304	CTB-55O6.12	0.000156733387854134	-0.725722644	0.012	0.016	1	no change
8305	8305	SFMBT1	0.000157442684350574	-0.147788291	0.062	0.053	1	no change
8306	8306	WI2-85898F10.1	0.000158202092406414	-0.496395268	0.034	0.027	1	no change
8307	8307	HOXB13	0.000159612174621041	-0.429407853	0.029	0.036	1	no change
8308	8308	C1GALT1	0.000161394751984255	-0.445954911	0.146	0.154	1	no change
8309	8309	ZKSCAN4	0.0001618106841538	0.17668572637787	0.012	0.008	1	no

				4				change
8310	8310	AC129778.2	0.000163213867859082	-0.124422569	0.012	0.008	1	no change
8311	8311	EDA2R	0.000163690093457334	-0.959170282	0.007	0.01	1	no change
8312	8312	SMPD2	0.000168423627794202	-0.201976313	0.102	0.089	1	no change
8313	8313	PLCB4	0.000172773154225972	0.152066198475965	0.013	0.009	1	no change
8314	8314	DUSP10	0.000173012547598052	-0.46655188	0.162	0.169	1	no change
8315	8315	ZNF66	0.000174322972510886	0.315049805415771	0.01	0.006	1	no change
8316	8316	TREM1	0.000174795632005614	-0.173285529	0.013	0.009	1	no change
8317	8317	PDCD4	0.000176428470853121	-0.303081705	0.274	0.236	1	no change
8318	8318	ZNF707	0.000179524213567734	-0.107169172	0.015	0.011	1	no change
8319	8319	WIPF1	0.000183761854826995	-0.24837601	0.042	0.034	1	no change
8320	8320	ZNF527	0.00018653673587729	-0.160467161	0.017	0.013	1	no change
8321	8321	VWA1	0.000189068450119292	-0.23697907	0.11	0.096	1	no change
8322	8322	PDP2	0.00018928852582771	-0.187515817	0.037	0.03	1	no

			9					change
8323	8323	TMEM55A	0.000191028999288828	-0.836995789	0.063	0.07	1	no change
8324	8324	C1QC	0.000191403552118607	-1.41071035	0.029	0.035	1	down
8325	8325	DNMBP	0.000192069754532045	-0.241339201	0.104	0.091	1	no change
8326	8326	TGFB3	0.000194086558803975	-1.170888648	0.01	0.013	1	down
8327	8327	POLR2J2	0.00019410221695424	-0.192584527	0.043	0.035	1	no change
8328	8328	RP11-368L12.1	0.000196073350290883	0.204533421130723	0.01	0.007	1	no change
8329	8329	RP11-147L13.13	0.000199209577527129	-0.161531161	0.013	0.009	1	no change
8330	8330	CCDC85C	0.000205552514971075	-0.172524289	0.04	0.033	1	no change
8331	8331	RP6-65G23.3	0.000210841288647395	-0.470296187	0.05	0.058	1	no change
8332	8332	LINC00893	0.000211168605484891	-0.118105992	0.02	0.015	1	no change
8333	8333	EPB41L5	0.000211909603068904	-0.216636305	0.085	0.073	1	no change
8334	8334	AP000692.10	0.000212976999854805	-0.198872406	0.022	0.017	1	no change
8335	8335	FHIT	0.00021541788377729	-0.179568523	0.165	0.146	1	no

								change
8336	8336	VAC14	0.000216744651505986	-0.232576033	0.088	0.076	1	no change
8337	8337	TFAP2B	0.000218859718247856	0.305479514526512	0.011	0.008	1	no change
8338	8338	LINC00630	0.000220472243899529	0.171037029745295	0.01	0.007	1	no change
8339	8339	TK2	0.000220925116377198	-0.335593823	0.072	0.062	1	no change
8340	8340	PNMA1	0.000223901982272743	-0.408870319	0.021	0.016	1	no change
8341	8341	SLC35F6	0.000227376518649581	-0.256006243	0.139	0.122	1	no change
8342	8342	ZNF227	0.000229296579246881	-0.136834743	0.033	0.027	1	no change
8343	8343	ABCA9	0.000229382380635313	-0.628291935	0.019	0.014	1	no change
8344	8344	MRPL54	0.000230117434433897	-0.135462631	0.563	0.472	1	no change
8345	8345	ZG16B	0.000231653635151748	-0.410894331	0.264	0.227	1	no change
8346	8346	NOD1	0.000231870120245265	-0.244055385	0.014	0.01	1	no change
8347	8347	ANO9	0.000232099955190486	-0.271105695	0.053	0.044	1	no change
8348	8348	IAH1	0.00023351459704212	-0.279244743	0.435	0.373	1	no

			3					change
8349	8349	HLA-DQB1	0.000234484792433365	-0.470427668	0.173	0.179	1	no change
8350	8350	NRG2	0.000235326241128377	-0.128318379	0.021	0.016	1	no change
8351	8351	MMP13	0.000239169770017475	-0.545783481	0.036	0.043	1	no change
8352	8352	GPR155	0.000241007218073233	-0.229478227	0.022	0.017	1	no change
8353	8353	NAV1	0.00024292956665484	-0.384747782	0.026	0.02	1	no change
8354	8354	SLC25A21-AS1	0.000243556357350222	0.280632631120137	0.01	0.007	1	no change
8355	8355	MAST3	0.000244645338411623	-0.165021026	0.018	0.013	1	no change
8356	8356	CHST11	0.000244675418127752	-0.894067713	0.009	0.012	1	no change
8357	8357	ALDH3B2	0.000245487880779444	-0.295763268	0.091	0.079	1	no change
8358	8358	IP6K1	0.000248126169491189	-0.147241248	0.055	0.047	1	no change
8359	8359	AC137932.4	0.000248599511647004	-0.110005421	0.017	0.013	1	no change
8360	8360	VAMP4	0.000249189411288361	-0.213063742	0.117	0.103	1	no change
8361	8361	TMEM144	0.00025385540287551	-0.340980094	0.096	0.083	1	no

			4					change
8362	8362	MUC12	0.000256021505749851	-1.04027558	0.025	0.03	1	down
8363	8363	SLC13A3	0.000257252900393053	0.174400155395573	0.021	0.016	1	no change
8364	8364	FHDC1	0.000259232301505119	-0.181244	0.016	0.012	1	no change
8365	8365	RIPK2	0.000260957818186517	-0.319240783	0.181	0.158	1	no change
8366	8366	PNCK	0.000265602573342882	0.247188685007121	0.106	0.095	1	no change
8367	8367	PPP1R13B	0.000266957725236956	-0.157381747	0.066	0.057	1	no change
8368	8368	ZNF813	0.000267999915651528	-0.138000499	0.016	0.011	1	no change
8369	8369	ZFYVE28	0.000268878345913567	-0.23388154	0.049	0.041	1	no change
8370	8370	IL11RA	0.000269002826961855	-1.026235525	0.023	0.028	1	down
8371	8371	CH17-340M24.3	0.000273144358020875	-0.444131259	0.189	0.193	1	no change
8372	8372	KRT14	0.000276733934020067	-0.279587608	0.054	0.062	1	no change
8373	8373	QSOX1	0.000278357723640919	-0.366215724	0.446	0.38	1	no change
8374	8374	NR2F1-AS1	0.00028382215345166	-0.563648797	0.016	0.012	1	no

			3					change
8375	8375	PQLC2L	0.00028415113067156 9	-0.237368947	0.022	0.017	1	no change
8376	8376	RRM2B	0.00028722936454787 5	-0.164233743	0.104	0.092	1	no change
8377	8377	LINC00278	0.00029809014141733 5	0.32067219228112	0.01	0.007	1	no change
8378	8378	CHUK	0.00030173360945876 8	-0.197142002	0.046	0.039	1	no change
8379	8379	GLTSCR1	0.00030676189782418 8	-0.101918706	0.021	0.016	1	no change
8380	8380	ELMSAN1	0.00030682812784177	-0.426125706	0.16	0.139	1	no change
8381	8381	SULT1C2	0.00030811064264295 4	-0.820055874	0.008	0.012	1	no change
8382	8382	GAL3ST4	0.00030957612404958 2	-0.888394769	0.017	0.022	1	no change
8383	8383	CTSO	0.00030965242158713 8	-0.488408297	0.111	0.096	1	no change
8384	8384	IFI30	0.00031266293662110 4	-0.702987278	0.015	0.019	1	no change
8385	8385	FBXO27	0.00031383809915306 1	-0.182762627	0.061	0.052	1	no change
8386	8386	KCTD6	0.00031390336661406	-0.165584497	0.025	0.02	1	no change
8387	8387	FLVCR1-AS1	0.00031648872763246	-0.610694916	0.021	0.026	1	no

			5					change
8388	8388	MSC	0.000322726881677713	-1.153442196	0.016	0.02	1	down
8389	8389	NKX3-1	0.000326772215791244	-0.19921925	0.017	0.012	1	no change
8390	8390	SNX8	0.000327170040270826	-0.218974323	0.075	0.065	1	no change
8391	8391	RELB	0.000329842875747666	-0.519203368	0.118	0.125	1	no change
8392	8392	ADM5	0.000329869826346051	-0.124795342	0.012	0.008	1	no change
8393	8393	ZNRF1	0.000329973423828167	-0.175070205	0.131	0.116	1	no change
8394	8394	SNHG21	0.000334127736598388	-0.201761976	0.071	0.061	1	no change
8395	8395	EIF5	0.000335507758586035	-0.172223384	0.585	0.498	1	no change
8396	8396	SIM2	0.000335571144450699	-0.544012016	0.027	0.033	1	no change
8397	8397	GS1-600G8.5	0.000336782687138334	-0.561611087	0.031	0.037	1	no change
8398	8398	ADAMTSL5	0.000338600057259847	-0.599401204	0.018	0.023	1	no change
8399	8399	TRPV6	0.000341786716363299	-0.652387925	0.017	0.021	1	no change
8400	8400	PRKAB2	0.00034586720653154	-0.141060649	0.062	0.053	1	no

			6					change
8401	8401	PTPRN2	0.000346821005659027	-0.232331476	0.17	0.149	1	no change
8402	8402	FGD5	0.000347963247643743	-0.498217239	0.025	0.031	1	no change
8403	8403	EXOC8	0.000348228715289306	-0.117229963	0.039	0.033	1	no change
8404	8404	TMEM8B	0.000353422264919136	-0.15439878	0.031	0.025	1	no change
8405	8405	NFIX	0.000354318763543629	-0.569382344	0.05	0.042	1	no change
8406	8406	ARRDC3	0.00035650002460649	-0.622772123	0.149	0.153	1	no change
8407	8407	FRMD8	0.000360356426503539	-0.224996931	0.209	0.185	1	no change
8408	8408	UGT1A10	0.000360965581137476	-0.663664543	0.021	0.026	1	no change
8409	8409	KIF12	0.000360979457318514	0.129937475891746	0.011	0.008	1	no change
8410	8410	ANKRD65	0.000365632662431495	-0.111070684	0.225	0.202	1	no change
8411	8411	RNF43	0.000366353136247681	-0.100367239	0.047	0.039	1	no change
8412	8412	TIGD6	0.00036638145739324	-0.130557556	0.027	0.022	1	no change
8413	8413	MESP1	0.00037406921699653	-0.647276754	0.037	0.043	1	no

			5					change
8414	8414	NAV3	0.000375616241864576	-0.337674435	0.011	0.008	1	no change
8415	8415	TNFAIP8L3	0.000375769297421936	-1.143411464	0.012	0.016	1	down
8416	8416	OASL	0.00037876998219245	-0.499223828	0.088	0.096	1	no change
8417	8417	KLC3	0.00037877522053406	-0.705826755	0.025	0.03	1	no change
8418	8418	ABHD8	0.000383074739456098	-0.108133343	0.047	0.04	1	no change
8419	8419	AGPAT4	0.00038479402177098	-0.276410947	0.018	0.014	1	no change
8420	8420	ALDH16A1	0.000403281761477785	-0.243207129	0.195	0.172	1	no change
8421	8421	FEM1C	0.000413377600788159	-0.399510604	0.098	0.085	1	no change
8422	8422	MAMDC4	0.000417996634718922	-0.369850343	0.014	0.01	1	no change
8423	8423	ZSCAN1	0.000420026113491685	-0.188481012	0.015	0.011	1	no change
8424	8424	C1orf21	0.000420521283997864	-0.299316005	0.306	0.264	1	no change
8425	8425	PPP1R15B	0.000421914435915777	-0.422037518	0.283	0.275	1	no change
8426	8426	FBLL1	0.00042328276042339	0.17020701803093	0.011	0.008	1	no

			3	7				change
8427	8427	LRRN1	0.000428224820479751	0.191012109173451	0.014	0.01	1	no change
8428	8428	RP11-38M8.1	0.000430013236855127	-0.280904518	0.06	0.051	1	no change
8429	8429	RNF208	0.000431811105076541	-0.513514917	0.027	0.033	1	no change
8430	8430	PPIL6	0.000434881556488656	-0.134079836	0.016	0.012	1	no change
8431	8431	TEX14	0.000439398822646173	-0.301775606	0.011	0.008	1	no change
8432	8432	CAMK2N1	0.000441343581468035	-0.155023754	0.657	0.575	1	no change
8433	8433	DALRD3	0.000444334362009947	-0.35065484	0.173	0.152	1	no change
8434	8434	FAM71F2	0.000445361659710393	0.184679118909021	0.011	0.008	1	no change
8435	8435	SNX29	0.000446430299277536	-0.355059759	0.059	0.05	1	no change
8436	8436	LINC01431	0.000446893339971073	-0.799596267	0.007	0.011	1	no change
8437	8437	DUS2	0.000454586648610274	-0.321967509	0.047	0.04	1	no change
8438	8438	UGP2	0.000456191349374671	-0.60778849	0.462	0.419	1	no change
8439	8439	RP11-355B11.2	0.00046100222992864	0.22284839004404	0.012	0.009	1	no

				6				change
8440	8440	SEMA6B	0.000464622337373232	-0.137564967	0.011	0.008	1	no change
8441	8441	NOTCH4	0.000467762023704621	-0.783232538	0.026	0.032	1	no change
8442	8442	ZNF784	0.000468119876845917	-0.227867702	0.023	0.018	1	no change
8443	8443	STX1A	0.000468850038721789	-0.19625043	0.035	0.029	1	no change
8444	8444	MISP	0.000472125124004933	-0.131538439	0.091	0.08	1	no change
8445	8445	DNAJC27-AS1	0.000473821815235262	-0.271902709	0.049	0.042	1	no change
8446	8446	RP1-102K2.8	0.000485561086421156	-0.829752056	0.009	0.012	1	no change
8447	8447	UBE2L6	0.000492286197063828	-0.169461526	0.386	0.337	1	no change
8448	8448	SLC2A13	0.000493994718754176	-0.448812916	0.021	0.016	1	no change
8449	8449	RUNX2	0.000495643237820488	-0.181420762	0.02	0.015	1	no change
8450	8450	CELF4	0.000497214065230063	0.464262813828344	0.011	0.008	1	no change
8451	8451	C21orf33	0.000500908056279012	0.111505664383197	0.023	0.018	1	no change
8452	8452	PFKFB2	0.00051021289983923	-0.512966644	0.053	0.06	1	no

			6					change
8453	8453	SHC3	0.00051142296950842 6	-0.293326759	0.014	0.011	1	no change
8454	8454	SCLY	0.00051935188687746 3	-0.158446294	0.024	0.019	1	no change
8455	8455	BBOX1-AS1	0.00052299283875556 5	-0.209554615	0.148	0.131	1	no change
8456	8456	SSC5D	0.00052361150646770 1	-1.071106624	0.011	0.015	1	down
8457	8457	CSGALNACT2	0.00052530408730013	-0.364279621	0.047	0.039	1	no change
8458	8458	LPAR2	0.00053002866560675 1	-0.225919227	0.128	0.113	1	no change
8459	8459	RP11-588G21.2	0.00053302376329768 5	-0.230340211	0.017	0.013	1	no change
8460	8460	MTHFR	0.00053510589470761 5	-0.321299902	0.07	0.06	1	no change
8461	8461	DOK5	0.00053609846354181 7	-1.210251502	0.007	0.01	1	down
8462	8462	VASP	0.00053659240360382 7	-0.130859948	0.49	0.425	1	no change
8463	8463	GABARAPL1	0.00053757250423335 7	-0.259916731	0.324	0.282	1	no change
8464	8464	PPP1R3G	0.00054162742829670 3	-0.142865898	0.057	0.049	1	no change
8465	8465	KEL	0.00054370433083959	0.11613706363785	0.013	0.009	1	no

			5	2				change
8466	8466	WBP4	0.000543862681694394	-0.271706671	0.284	0.249	1	no change
8467	8467	PLEKHH1	0.000544311192518132	-0.1602732	0.031	0.025	1	no change
8468	8468	PTS	0.000545265448762562	-0.206912681	0.364	0.313	1	no change
8469	8469	SLC38A7	0.000545931620140459	-0.126378804	0.043	0.036	1	no change
8470	8470	SLC30A1	0.000546261213945987	-0.290819786	0.123	0.109	1	no change
8471	8471	MYZAP	0.000548100654914011	-0.441750765	0.073	0.081	1	no change
8472	8472	TRIM3	0.000549733982732704	-0.280692717	0.018	0.013	1	no change
8473	8473	RTP4	0.000551777557468751	-0.285456659	0.11	0.096	1	no change
8474	8474	FIS1	0.000561453689317077	-0.149665717	0.748	0.651	1	no change
8475	8475	CATSPER1	0.000562044092393912	-0.105148914	0.018	0.014	1	no change
8476	8476	CCDC121	0.000574182413012733	-0.226016443	0.017	0.013	1	no change
8477	8477	HDX	0.000574302442400571	-0.276397428	0.032	0.026	1	no change
8478	8478	AC003075.4	0.00057567045479199	-0.272190405	0.047	0.04	1	no

								change
8479	8479	TM9SF1	0.000578853469835376	-0.190703149	0.033	0.027	1	no change
8480	8480	PRKAG2-AS1	0.000582677309075115	-0.464732629	0.033	0.026	1	no change
8481	8481	UHRF1BP1L	0.000591326604674631	-0.144350741	0.047	0.04	1	no change
8482	8482	RAET1E	0.000603521440968304	-0.389824809	0.023	0.028	1	no change
8483	8483	C2orf81	0.000603595479392507	-0.208717702	0.01	0.007	1	no change
8484	8484	RGS19	0.000608210999155906	-0.260701722	0.108	0.095	1	no change
8485	8485	NDST2	0.000617776591684457	-0.490494831	0.02	0.024	1	no change
8486	8486	TEX264	0.000618030846422451	-0.325129806	0.371	0.35	1	no change
8487	8487	FKRP	0.000621210887855301	-0.173791515	0.037	0.03	1	no change
8488	8488	CERK	0.000640030733278294	-0.110103525	0.015	0.011	1	no change
8489	8489	EVPLL	0.000644976274580491	-0.480982037	0.02	0.025	1	no change
8490	8490	POMZP3	0.000645377929336888	-0.290283659	0.03	0.024	1	no change
8491	8491	TMEM62	0.00064886398531213	-0.151631069	0.065	0.056	1	no

								change
8492	8492	RP11-356I2.4	0.000652635770189749	-0.177605498	0.012	0.009	1	no change
8493	8493	LARP6	0.00065432849407276	-0.375654449	0.172	0.151	1	no change
8494	8494	HCLS1	0.000655141587656403	-0.247977766	0.055	0.047	1	no change
8495	8495	CST4	0.000658617396219373	-0.303042994	0.04	0.034	1	no change
8496	8496	HRH2	0.000664380453745634	-0.888936665	0.007	0.01	1	no change
8497	8497	TIMP4	0.000666129944589561	-0.524950264	0.023	0.029	1	no change
8498	8498	C5orf45	0.00066795390838638	-0.357775819	0.14	0.123	1	no change
8499	8499	CCNYL1	0.000669922549030708	-0.279651391	0.062	0.053	1	no change
8500	8500	TEC	0.000671552757125466	0.140073490448583	0.01	0.007	1	no change
8501	8501	CCR10	0.000677829685423631	-0.639159818	0.009	0.012	1	no change
8502	8502	PLXNA3	0.000696631141850858	-0.334444199	0.078	0.068	1	no change
8503	8503	SLC9A1	0.000698041348956478	-0.475964692	0.164	0.168	1	no change
8504	8504	IPMK	0.00070800048429118	-0.281977356	0.047	0.04	1	no

			6					change
8505	8505	B4GALT1	0.000708715318498135	-0.350814811	0.34	0.333	1	no change
8506	8506	GPRIN2	0.000709116848627074	-0.443564132	0.011	0.014	1	no change
8507	8507	ODF4	0.000710850556795654	-0.412131326	0.008	0.011	1	no change
8508	8508	AC013463.2	0.000713294893918753	-0.717523522	0.012	0.016	1	no change
8509	8509	GAB1	0.000720410691398252	-0.134853023	0.041	0.035	1	no change
8510	8510	EPB42	0.000721538304444338	-0.400767144	0.02	0.015	1	no change
8511	8511	RNF19B	0.000721836978995998	-0.346462091	0.112	0.098	1	no change
8512	8512	HLA-DMA	0.000724372972581627	-0.270395852	0.359	0.318	1	no change
8513	8513	RP11-326K13.4	0.00073112766627775	0.109495767412317	0.011	0.008	1	no change
8514	8514	ARHGAP30	0.000736557902920408	-0.229773558	0.101	0.089	1	no change
8515	8515	ZNF765	0.000759074201290818	-0.113550229	0.031	0.026	1	no change
8516	8516	GAS7	0.000763861850941691	-1.238924122	0.007	0.01	1	down
8517	8517	CTD-2095E4.5	0.00076905732132796	-0.120805276	0.037	0.031	1	no

			7					change
8518	8518	IGHGP	0.000769675512524262	-2.804853572	0.01	0.013	1	down
8519	8519	GAP43	0.000772181670569855	-0.256084915	0.012	0.008	1	no change
8520	8520	RP11-27M24.1	0.000783854583444058	-0.236119619	0.03	0.024	1	no change
8521	8521	SACS	0.000786330342590154	-0.317397773	0.011	0.007	1	no change
8522	8522	ALDH1A2	0.000789322001873007	-0.298643887	0.062	0.054	1	no change
8523	8523	C19orf33	0.000789764509147709	-0.281984776	0.726	0.622	1	no change
8524	8524	FST	0.000798606998177163	-0.488707353	0.095	0.083	1	no change
8525	8525	RP11-656D10.3	0.00080330295285597	0.574154917380486	0.011	0.008	1	no change
8526	8526	C7orf50	0.000807810956235514	-0.175643688	0.434	0.371	1	no change
8527	8527	C14orf28	0.000811170452891853	-0.246325664	0.049	0.042	1	no change
8528	8528	SHISA4	0.000814197669523743	-0.502886494	0.159	0.163	1	no change
8529	8529	LINC01297	0.000816294438884474	-0.299014765	0.034	0.028	1	no change
8530	8530	C7orf60	0.00081724010367998	-0.172499299	0.058	0.05	1	no

			8					change
8531	8531	MICAL1	0.000834465228394263	-0.385896166	0.036	0.03	1	no change
8532	8532	GTPBP2	0.000835982452625496	-0.240111856	0.058	0.05	1	no change
8533	8533	TRBC1	0.000838831181387203	0.1364889648621	0.013	0.009	1	no change
8534	8534	FRMD6-AS1	0.000844938787633032	-0.279792913	0.041	0.034	1	no change
8535	8535	IL16	0.000847299473581711	-1.063735803	0.01	0.013	1	down
8536	8536	ANKRD35	0.000853304698357506	-0.728168899	0.033	0.039	1	no change
8537	8537	YTHDF3-AS1	0.000853982126271864	-0.130254038	0.059	0.051	1	no change
8538	8538	SIMC1	0.000855830980198715	-0.234166805	0.015	0.011	1	no change
8539	8539	PDXK	0.000857237807383779	-0.246918366	0.382	0.322	1	no change
8540	8540	C11orf80	0.00086241422340667	-0.212456087	0.041	0.034	1	no change
8541	8541	CTD-2021H9.3	0.000873161377744483	-0.421765722	0.035	0.029	1	no change
8542	8542	GATA6	0.00088737025591561	-1.198416315	0.017	0.021	1	down
8543	8543	CD109	0.000887554662308523	-0.937667083	0.011	0.015	1	no change

8544	8544	ZNF653	0.000927986414870843	-0.156640329	0.013	0.01	1	no change
8545	8545	GAS8	0.00092867138132946	-0.170359231	0.043	0.037	1	no change
8546	8546	RAB40B	0.000930182730202644	-0.239188008	0.308	0.267	1	no change
8547	8547	RP4-533D7.5	0.000934402426592592	0.111942782872672	0.016	0.013	1	no change
8548	8548	C5orf17	0.000937344978935021	0.304373721373189	0.016	0.012	1	no change
8549	8549	ACTL10	0.000939055244665858	-0.179005005	0.022	0.017	1	no change
8550	8550	PLD1	0.000940379145458107	-0.190147045	0.051	0.043	1	no change
8551	8551	FRZB	0.000940438790356079	-1.13892623	0.008	0.011	1	down
8552	8552	A2ML1	0.000946108754797273	-0.535508726	0.016	0.021	1	no change
8553	8553	OPTN	0.000952703891751637	-0.241944649	0.288	0.251	1	no change
8554	8554	WFS1	0.000978369871425628	-0.187075391	0.076	0.067	1	no change
8555	8555	LRTOMT	0.000995883285077894	-0.151033104	0.047	0.04	1	no change
8556	8556	VKORC1	0.000995910088119404	-0.338942224	0.478	0.432	1	no change

8557	8557	ATP2B4	0.000996827459190905	-0.337742329	0.105	0.093	1	no change
8558	8558	KLHDC1	0.00101568227930854	-0.139028039	0.013	0.01	1	no change
8559	8559	CD4	0.00102417267825829	-0.263025613	0.029	0.023	1	no change
8560	8560	HEMK1	0.00102448198146456	-0.224282266	0.086	0.076	1	no change
8561	8561	NPHP1	0.00102718301900614	-0.254174125	0.061	0.052	1	no change
8562	8562	CASP10	0.00102928672527582	-0.276476389	0.072	0.062	1	no change
8563	8563	NFATC4	0.00103771293699403	-0.677287823	0.024	0.029	1	no change
8564	8564	LRRC24	0.00104497914331365	-0.163693447	0.013	0.009	1	no change
8565	8565	PHF7	0.0010454340451703	-0.167777632	0.019	0.015	1	no change
8566	8566	TCF7L1	0.00104987441597188	-0.311318132	0.014	0.01	1	no change
8567	8567	PTAFR	0.00105116159269309	-0.312955966	0.033	0.028	1	no change
8568	8568	ATP6V0A1	0.00106484969207202	-0.556360675	0.118	0.124	1	no change
8569	8569	RALBP1	0.00107457598433919	-0.139362253	0.618	0.536	1	no change

8570	8570	VCX3A	0.00108100668382053	-0.794670993	0.022	0.027	1	no change
8571	8571	NFKBIE	0.00111099166662526	-0.582554941	0.12	0.126	1	no change
8572	8572	TCEAL3	0.0011330971147923	-0.266401985	0.335	0.291	1	no change
8573	8573	EPAS1	0.00113714787449645	-0.313327603	0.206	0.182	1	no change
8574	8574	INSIG2	0.00115202036588083	-0.524899388	0.203	0.202	1	no change
8575	8575	PNPLA8	0.00115401372257293	-0.244813989	0.285	0.25	1	no change
8576	8576	RP11-967K21.1	0.00116768976274839	0.119230395803311	0.013	0.01	1	no change
8577	8577	ENO2	0.00117729558748582	-0.435229841	0.064	0.071	1	no change
8578	8578	LSS	0.00117736387059732	-0.635907039	0.052	0.058	1	no change
8579	8579	CTD-2139B15.2	0.0011853992461746	-0.674778626	0.013	0.017	1	no change
8580	8580	TMEM42	0.00118667616891945	-0.22531841	0.116	0.103	1	no change
8581	8581	THEM5	0.0011952610277429	0.200513830185549	0.02	0.016	1	no change
8582	8582	CPM	0.00119988012702689	-0.737644722	0.063	0.07	1	no change

8583	8583	CGN	0.0012010542874494	-0.190682764	0.098	0.087	1	no change
8584	8584	MILR1	0.00120970796014821	-0.250618071	0.013	0.01	1	no change
8585	8585	P4HA2	0.00121084032914004	-0.478699316	0.1	0.088	1	no change
8586	8586	NT5DC3	0.00121649237120061	-0.152796369	0.013	0.01	1	no change
8587	8587	CTD-2396E7.11	0.00121923700902265	-0.213185055	0.049	0.042	1	no change
8588	8588	RP11-51J9.5	0.00123534983117108	-0.318737872	0.083	0.073	1	no change
8589	8589	CARD14	0.00123758377537065	-0.136899534	0.02	0.015	1	no change
8590	8590	ATP1A4	0.0012547954794872	-0.426807512	0.251	0.246	1	no change
8591	8591	KDM5D	0.00125646490442963	-0.189478703	0.063	0.055	1	no change
8592	8592	MME	0.00125757305795129	-0.317211118	0.038	0.044	1	no change
8593	8593	EIF4EBP1	0.00126492181279475	-0.140670792	0.473	0.413	1	no change
8594	8594	FAM69A	0.00128604394451997	-0.735032215	0.02	0.025	1	no change
8595	8595	ACKR3	0.00128677842901149	-0.789955566	0.057	0.048	1	no change

8596	8596	C11orf63	0.00128789299526109	-0.1500686	0.02	0.016	1	no change
8597	8597	SEZ6L2	0.00129586952388712	-0.440795399	0.199	0.2	1	no change
8598	8598	RNF135	0.00129891435225193	-0.24235743	0.154	0.137	1	no change
8599	8599	LRP10	0.00129980853016611	-0.205904371	0.398	0.342	1	no change
8600	8600	HOMER3	0.0013038642466536	-0.201317974	0.2	0.179	1	no change
8601	8601	KDM7A	0.00131050931136525	-0.25504327	0.062	0.054	1	no change
8602	8602	CNTN1	0.00131194061042945	-1.070390295	0.011	0.014	1	down
8603	8603	POLD4	0.00132298891295797	-0.265381328	0.127	0.113	1	no change
8604	8604	LRRC75B	0.00133528397373084	-0.360150273	0.015	0.011	1	no change
8605	8605	UVSSA	0.00133843115245256	-0.128148468	0.041	0.034	1	no change
8606	8606	MYO3B	0.00134803637875408	-0.589388866	0.009	0.013	1	no change
8607	8607	COL22A1	0.00135536750732827	-0.65124929	0.014	0.018	1	no change
8608	8608	PNPLA2	0.00135809594979008	-0.198677725	0.428	0.364	1	no change
8609	8609	ATG16L2	0.00136252802382073	-0.224906357	0.051	0.043	1	no

									change
8610	8610	PARP3	0.00136480290862214	-0.209777464	0.069	0.06		1	no change
8611	8611	AC027119.1	0.00137242493495808	-0.696207842	0.01	0.013		1	no change
8612	8612	TOX2	0.00137786908989568	-1.130048233	0.012	0.016		1	down
8613	8613	KRT8	0.00138008755514084	-0.121826347	0.945	0.861		1	no change
8614	8614	RAPGEF3	0.00138782135039799	-0.218454462	0.033	0.027		1	no change
8615	8615	ISG20	0.00138851363908151	-0.380997861	0.179	0.183		1	no change
8616	8616	TMEM88	0.00139621615877644	-0.390513077	0.038	0.032		1	no change
8617	8617	ZNF583	0.00140630151800784	-0.10410401	0.027	0.022		1	no change
8618	8618	PAPOLA	0.00141845853659588	-0.172861721	0.663	0.575		1	no change
8619	8619	YKT6	0.00143670388416615	-0.231086341	0.141	0.126		1	no change
8620	8620	FAM72A	0.00143961227432128	-0.651749778	0.012	0.016		1	no change
8621	8621	MAST1	0.00144980887082135	0.295124394945502	0.012	0.009		1	no change
8622	8622	FAM71A	0.0014685287278191	-0.336553034	0.021	0.017		1	no change

8623	8623	RIC3		0.00148001603333625	-0.120914258	0.081	0.072	1	no change
8624	8624	TMEM27		0.00148721370514938	-0.15134561	0.013	0.01	1	no change
8625	8625	C5orf49		0.00148823416705525	-0.146748578	0.012	0.009	1	no change
8626	8626	PLEKHG2		0.00150966247389237	-0.389904698	0.021	0.016	1	no change
8627	8627	SERHL2		0.00152470707558482	-0.226019875	0.014	0.011	1	no change
8628	8628	SAP30		0.0015362366922837	-0.234420737	0.071	0.063	1	no change
8629	8629	RAB7A		0.00154057948115861	-0.153425487	0.707	0.621	1	no change
8630	8630	IL23A		0.00155489665137085	-0.817218847	0.063	0.069	1	no change
8631	8631	KCNQ1OT1		0.00157937923668753	-0.177013626	0.34	0.305	1	no change
8632	8632	SBF2-AS1		0.0015887578600383	-0.151076668	0.043	0.037	1	no change
8633	8633	UBB		0.00160737591412852	-0.113968735	0.98	0.96	1	no change
8634	8634	RP11-381K20.2		0.00161081081089529	0.144204334109885	0.014	0.01	1	no change
8635	8635		2-Mar	0.00161525121092613	-0.264445908	0.123	0.11	1	no change

8636	8636	VEGFB	0.00161929745997512	-0.536212316	0.091	0.097	1	no change
8637	8637	MAFG-AS1	0.00161964896289418	-0.109448998	0.013	0.01	1	no change
8638	8638	GATS	0.00164710129360821	-0.486218046	0.02	0.024	1	no change
8639	8639	VPS18	0.00165114046033805	-0.591609937	0.048	0.054	1	no change
8640	8640	CTB-50L17.14	0.00166744731509298	-0.582994203	0.039	0.045	1	no change
8641	8641	LIX1L	0.00167367786100375	-0.422936074	0.038	0.032	1	no change
8642	8642	HK2	0.00167446767974879	-0.242725399	0.143	0.128	1	no change
8643	8643	CSRNP3	0.00168437972193195	-0.19206182	0.017	0.013	1	no change
8644	8644	CHAC1	0.00170699613132499	-0.67488425	0.015	0.019	1	no change
8645	8645	LINC00174	0.0017133111183742	-0.109652053	0.011	0.008	1	no change
8646	8646	SLC2A6	0.00172491254480753	-0.39601541	0.063	0.07	1	no change
8647	8647	EGR4	0.00173318318340017	-0.59377033	0.028	0.033	1	no change
8648	8648	NAPA-AS1	0.00173705673928788	-0.160229689	0.027	0.023	1	no change

8649	8649	NCKIPSD	0.00176158425857323	-0.159320867	0.033	0.027	1	no change
8650	8650	AC079305.10	0.00176187122020621	-0.739368246	0.01	0.013	1	no change
8651	8651	SLC43A3	0.00176847935051943	-1.06239559	0.014	0.017	1	down
8652	8652	ALDH1A3	0.00179647004904497	-0.301926189	0.153	0.161	1	no change
8653	8653	BLOC1S3	0.00180331481059481	-0.172846935	0.033	0.028	1	no change
8654	8654	HLA-A	0.00183507494592243	-0.144263466	0.895	0.839	1	no change
8655	8655	LAG3	0.00184737567070089	-1.047806236	0.008	0.01	1	down
8656	8656	RP11-774O3.3	0.00185862389018273	0.109861241378678	0.025	0.02	1	no change
8657	8657	LRRC8E	0.00186202048239821	-0.199719027	0.034	0.029	1	no change
8658	8658	DNAJC12	0.00186272364388964	-1.073978423	0.01	0.013	1	down
8659	8659	PAOX	0.00187809078596246	-0.204627669	0.035	0.029	1	no change
8660	8660	EPB41L3	0.00188288281921809	-0.47118048	0.069	0.076	1	no change
8661	8661	APOM	0.00188932956823268	-0.121441099	0.032	0.027	1	no change
8662	8662	SERTAD4	0.00189002588056767	-0.30071275	0.059	0.051	1	no change
8663	8663	STKLD1	0.00190655104138111	0.30287193537180	0.01	0.007	1	no

			5					change
8664	8664	LRRC28	0.00190770414520582	-0.220902728	0.117	0.104	1	no change
8665	8665	SVOPL	0.00191757480052815	-0.815469704	0.009	0.012	1	no change
8666	8666	ATP1B1	0.00193277768481529	-0.278990709	0.556	0.501	1	no change
8667	8667	RP11-114B7.6	0.00193913308008148	-0.644722635	0.008	0.011	1	no change
8668	8668	KRT20	0.00194172680883558	-0.143810086	0.189	0.199	1	no change
8669	8669	FBXO41	0.00194350044291978	-0.156678742	0.015	0.012	1	no change
8670	8670	ASGR1	0.00194489318034331	-0.270628132	0.01	0.007	1	no change
8671	8671	CYB561D2	0.0019510726793325	-0.246535951	0.176	0.157	1	no change
8672	8672	ABL2	0.00196722149997384	-0.687288148	0.108	0.094	1	no change
8673	8673	DNM3OS	0.00197836653841655	-1.205855041	0.007	0.01	1	down
8674	8674	TMPRSS12	0.00199509795338725	-0.600060636	0.009	0.012	1	no change
8675	8675	ANTXR2	0.00199589631852026	-0.473751734	0.054	0.046	1	no change
8676	8676	CD163L1	0.00199645825520892	-0.672363864	0.016	0.02	1	no change

8677	8677	RAB3D	0.00199741507601148	-0.188111016	0.065	0.057	1	no change
8678	8678	PART1	0.00201353095057734	-0.380477247	0.091	0.098	1	no change
8679	8679	SERTAD4-AS1	0.00203242102684245	-0.260742531	0.073	0.064	1	no change
8680	8680	NID1	0.00203720311149541	-0.602926996	0.044	0.038	1	no change
8681	8681	TMEM60	0.00204933451339042	-0.244718892	0.254	0.224	1	no change
8682	8682	BCL10	0.00205350237457016	-0.399124583	0.316	0.304	1	no change
8683	8683	C9orf106	0.00207032598581881	-0.799868461	0.007	0.01	1	no change
8684	8684	SLC41A2	0.00209498943388593	-1.060323534	0.008	0.011	1	down
8685	8685	SMOX	0.00210083097614059	-0.202974762	0.049	0.043	1	no change
8686	8686	MIR3945HG	0.00211843759333341	-0.898659422	0.009	0.012	1	no change
8687	8687	GOLGA6L4	0.00212374728087309	-0.231768676	0.014	0.011	1	no change
8688	8688	FBXO2	0.00213275484723769	-0.299240431	0.052	0.045	1	no change
8689	8689	SDF2	0.00213813549742316	-0.215060154	0.397	0.342	1	no change
8690	8690	CHKB	0.00213921589133844	-0.293899616	0.041	0.035	1	no

								change
8691	8691	DNAJA4	0.00215016649815322	-0.454165904	0.307	0.293	1	no change
8692	8692	ENTPD6	0.00215214921399261	-0.254467833	0.12	0.107	1	no change
8693	8693	BCL7B	0.00215704978480063	-0.227301891	0.312	0.273	1	no change
8694	8694	SLC9A3R2	0.00216132948944606	-0.438954749	0.085	0.075	1	no change
8695	8695	APBB2	0.00218269633268901	-0.298275536	0.025	0.02	1	no change
8696	8696	MIR4435-2HG	0.00218865450927115	-0.5686917	0.159	0.163	1	no change
8697	8697	THAP7-AS1	0.0021890379144942	-0.213648	0.031	0.025	1	no change
8698	8698	AC245100.1	0.00219594010080014	-0.230999971	0.098	0.088	1	no change
8699	8699	RP5-875H18.9	0.00219949300567284	-0.70786383	0.014	0.018	1	no change
8700	8700	TMEM65	0.0022005247798932	-0.552938607	0.049	0.055	1	no change
8701	8701	DOK4	0.0022162072775389	-0.140721883	0.074	0.065	1	no change
8702	8702	RP11-532F12.5	0.0022387906044223	-0.177027714	0.513	0.428	1	no change
8703	8703	GIPC1	0.00227658280974266	-0.255850457	0.499	0.446	1	no

								change
8704	8704	MFSD8	0.00232522803564542	-0.266361961	0.037	0.031	1	no change
8705	8705	MMP11	0.00233887322134992	-1.603058958	0.016	0.012	1	down
8706	8706	C4orf32	0.00234047544180571	-0.537266354	0.152	0.155	1	no change
8707	8707	RP13-270P17.1	0.00234374009768571	-0.667385039	0.009	0.012	1	no change
8708	8708	CXXC5	0.00235158906332919	-0.358626836	0.222	0.222	1	no change
8709	8709	TMEM238	0.00236372463564383	-0.351073648	0.047	0.053	1	no change
8710	8710	DENND2D	0.00240663933700695	-0.195802665	0.239	0.213	1	no change
8711	8711	IGF2BP2	0.00241768960311889	-0.408085879	0.025	0.02	1	no change
8712	8712	SLC10A7	0.00245915375757866	-0.245976409	0.037	0.032	1	no change
8713	8713	RIPK4	0.00246729173343	-0.453412254	0.176	0.178	1	no change
8714	8714	RNF11	0.00247439972048013	-0.170410534	0.508	0.428	1	no change
8715	8715	MARC2	0.00248854900625073	-0.186999536	0.035	0.03	1	no change
8716	8716	SYT7	0.00249648035898343	0.151309634511906	0.02	0.016	1	no change

8717	8717	ARHGAP27	0.00252190943770256	-0.235855342	0.168	0.151	1	no change
8718	8718	MAGEA9B	0.00252282350767732	-0.568651517	0.014	0.018	1	no change
8719	8719	NRP2	0.00253418061374109	-0.937093234	0.017	0.02	1	no change
8720	8720	GNPTG	0.0025465084280952	-0.284124843	0.214	0.189	1	no change
8721	8721	RAB11FIP5	0.00256648270467608	-0.175710013	0.036	0.031	1	no change
8722	8722	MYLK	0.00256918027106351	-0.728966404	0.02	0.024	1	no change
8723	8723	GS1-124K5.4	0.00260705268986524	-0.228292746	0.072	0.064	1	no change
8724	8724	STOM	0.00261099010394723	-0.61199957	0.137	0.139	1	no change
8725	8725	CYBB	0.00262087090051501	-0.917180771	0.011	0.015	1	no change
8726	8726	INPP5D	0.00262494525146113	-0.177963695	0.045	0.039	1	no change
8727	8727	PLXND1	0.00263979367521559	-0.276426257	0.017	0.013	1	no change
8728	8728	CCRL2	0.0026519308165926	-0.661590467	0.038	0.043	1	no change
8729	8729	PQLC1	0.00265442008965152	-0.232249315	0.102	0.091	1	no change

8730	8730	VDR	0.00268226763786181	-0.163620723	0.072	0.064	1	no change
8731	8731	PPP1R3B	0.0027017163810768	-0.205362484	0.057	0.049	1	no change
8732	8732	CDC42EP3	0.00271359326451511	-0.438930708	0.124	0.13	1	no change
8733	8733	SAMD4A	0.00275008753171465	-0.501330529	0.058	0.05	1	no change
8734	8734	HFE	0.00276720533973794	-0.109042455	0.024	0.02	1	no change
8735	8735	PAXIP1-AS2	0.00278286777313684	-0.150274775	0.02	0.016	1	no change
8736	8736	NBPF15	0.00278535425974185	-0.224263086	0.011	0.008	1	no change
8737	8737	ID2	0.00279608093418555	-0.286518752	0.655	0.607	1	no change
8738	8738	KB-1732A1.1	0.00279668140160996	-0.580799957	0.032	0.038	1	no change
8739	8739	SERPINA1	0.0028099027130727	-0.781686818	0.071	0.077	1	no change
8740	8740	RP11-793H13.11	0.00281105669350999	-0.129261609	0.018	0.014	1	no change
8741	8741	GMPPB	0.00283696564707684	-0.252260068	0.134	0.12	1	no change
8742	8742	PITPNM2	0.00289807393470193	-0.190792445	0.01	0.007	1	no change

8743	8743	RNF181	0.00290064190748179	-0.131695315	0.723	0.636	1	no change
8744	8744	STAT3	0.00295895144958583	-0.272534994	0.355	0.307	1	no change
8745	8745	ACKR2	0.00298725153075327	-0.584510913	0.019	0.023	1	no change
8746	8746	ZNF503-AS1	0.00303740863731743	-0.50831654	0.02	0.025	1	no change
8747	8747	BACE1	0.00303937703267838	-0.342973247	0.016	0.013	1	no change
8748	8748	ACE2	0.00304560458169119	0.160479167888109	0.01	0.007	1	no change
8749	8749	PLEKHO2	0.00304897056414385	-0.812659708	0.025	0.029	1	no change
8750	8750	FZD1	0.00308637332459287	-0.559603376	0.034	0.039	1	no change
8751	8751	ANGPTL2	0.00309760160281697	-1.383826547	0.016	0.02	1	down
8752	8752	PVRL4	0.0031246775292092	-0.226138566	0.498	0.423	1	no change
8753	8753	NICN1	0.00312612638626082	-0.282414615	0.026	0.022	1	no change
8754	8754	MORF4L1	0.00314032472232777	-0.117299209	0.901	0.823	1	no change
8755	8755	RP11-127B20.3	0.00314269852836337	-0.277831413	0.049	0.042	1	no change
8756	8756	CFB	0.00314763194750511	-1.040315552	0.009	0.012	1	down

8757	8757	OSMR-AS1	0.00314782700593715	-0.243143188	0.011	0.008	1	no change
8758	8758	SEC16B	0.00315237710462832	-0.25083354	0.011	0.008	1	no change
8759	8759	PDCD4-AS1	0.0031768392547903	-0.475109012	0.02	0.025	1	no change
8760	8760	RP11-386G11.10	0.00322301452970111	-0.170266571	0.024	0.02	1	no change
8761	8761	FAM126A	0.0032558391699601	-0.450931402	0.069	0.06	1	no change
8762	8762	CPA4	0.00329086022896934	-0.715558993	0.009	0.012	1	no change
8763	8763	GJB3	0.00329716442047168	-0.221068557	0.15	0.136	1	no change
8764	8764	LYNX1	0.00332987835410208	-0.763484093	0.059	0.065	1	no change
8765	8765	ANXA8L1	0.00333234348100786	-0.160775474	0.053	0.046	1	no change
8766	8766	REXO2	0.00334604610729664	-0.299876549	0.543	0.477	1	no change
8767	8767	PRRX2	0.00337025594145148	-1.394396384	0.008	0.011	1	down
8768	8768	ZNF585B	0.00337103917978449	-0.152545633	0.028	0.023	1	no change
8769	8769	ARHGAP4	0.00337959395791165	-0.257397939	0.029	0.024	1	no change
8770	8770	BANK1	0.00345312214706152	-0.145978554	0.074	0.066	1	no

								change
8771	8771	AP001046.5	0.00348332027442298	-0.101528731	0.01	0.007	1	no change
8772	8772	KALRN	0.00351089076770877	-0.1202078	0.013	0.01	1	no change
8773	8773	IGHA2	0.00352374418964028	-2.740002703	0.022	0.018	1	down
8774	8774	AC113189.5	0.00354396711671529	-0.178142785	0.062	0.055	1	no change
8775	8775	NEK8	0.00355534244426665	-0.267740272	0.113	0.102	1	no change
8776	8776	OSCP1	0.00360812675591215	-0.307547044	0.042	0.036	1	no change
8777	8777	LAGE3	0.00361230288383293	-0.115612303	0.515	0.443	1	no change
8778	8778	LINC00592	0.00365476029975466	-0.421247514	0.019	0.023	1	no change
8779	8779	RALY-AS1	0.00365861705405629	-0.205738152	0.086	0.077	1	no change
8780	8780	PER3	0.00366611329922087	-0.191931324	0.042	0.036	1	no change
8781	8781	HAP1	0.00370527263620759	-0.492873145	0.03	0.035	1	no change
8782	8782	CTD-2371O3.2	0.0037680657780709	-0.72416767	0.01	0.013	1	no change
8783	8783	MED14OS	0.00378159620715734	-0.28167814	0.016	0.012	1	no change

8784	8784	SLC25A43	0.0038112793712596	-0.141118502	0.048	0.042	1	no change
8785	8785	LINC00449	0.00383399042550488	-0.100683766	0.011	0.008	1	no change
8786	8786	NYNRIN	0.00387055501468665	-0.424379917	0.069	0.075	1	no change
8787	8787	ACTA2	0.00387738998206358	-0.275767748	0.046	0.051	1	no change
8788	8788	CD99L2	0.00388724876634305	-0.300653589	0.022	0.018	1	no change
8789	8789	HARBI1	0.00389586193784147	-0.169108723	0.021	0.017	1	no change
8790	8790	MXD1	0.00392434324759708	-0.542810386	0.258	0.249	1	no change
8791	8791	KANK2	0.00393386868031617	-0.508071921	0.051	0.044	1	no change
8792	8792	PARD3B	0.00393967011049973	-0.77391622	0.014	0.018	1	no change
8793	8793	RP3-428L16.2	0.00394829691662973	-0.109488379	0.013	0.01	1	no change
8794	8794	PRSS3	0.00395570621821025	-0.126237608	0.06	0.053	1	no change
8795	8795	MB	0.00395757997378486	-0.152175842	0.014	0.011	1	no change
8796	8796	NPB	0.00397015099679934	-0.143642567	0.029	0.025	1	no change

8797	8797	LPAR5	0.00398596343661739	-0.557050391	0.025	0.03	1	no change
8798	8798	JAZF1	0.00402720347941743	-0.517145663	0.028	0.023	1	no change
8799	8799	TMEM110	0.00403586191774339	-0.473718985	0.023	0.027	1	no change
8800	8800	COMMD7	0.00410100567201339	-0.187832083	0.48	0.407	1	no change
8801	8801	HOXD1	0.00420504304731268	-0.608131197	0.095	0.101	1	no change
8802	8802	APOBEC3H	0.00422216929110607	-0.535157332	0.013	0.016	1	no change
8803	8803	RARA-AS1	0.00424292780173392	-0.269533719	0.073	0.065	1	no change
8804	8804	USP2	0.00424887574241779	-0.463818755	0.016	0.012	1	no change
8805	8805	ARHGAP5-AS1	0.0042794424325954	-0.498643033	0.054	0.06	1	no change
8806	8806	CHMP2B	0.00428184125216064	-0.24988484	0.549	0.486	1	no change
8807	8807	PPM1K	0.0042967196827311	-0.416533122	0.073	0.064	1	no change
8808	8808	TRIM22	0.0043570617919927	-0.565477382	0.102	0.107	1	no change
8809	8809	KLHL26	0.00436419755710564	-0.23402351	0.017	0.013	1	no change

8810	8810	PHYH	0.00437348491569245	-0.264002072	0.247	0.22	1	no change
8811	8811	GLCE	0.00442423189357959	-0.346515922	0.046	0.039	1	no change
8812	8812	VSIG10L	0.00444157640006656	-0.608954904	0.011	0.014	1	no change
8813	8813	MOSPD3	0.00444271618606324	-0.238317423	0.272	0.24	1	no change
8814	8814	P2RX6	0.00446258800669658	-0.169233951	0.012	0.009	1	no change
8815	8815	MED8	0.00451342918323226	-0.235649625	0.188	0.169	1	no change
8816	8816	ZNF185	0.00458254508621179	-0.145703219	0.06	0.053	1	no change
8817	8817	NOXA1	0.00472591112946019	-0.350223936	0.034	0.029	1	no change
8818	8818	WNT9A	0.00480990365843904	-0.213209773	0.013	0.01	1	no change
8819	8819	MZF1-AS1	0.00485884897659611	-0.139050433	0.025	0.021	1	no change
8820	8820	CXorf40A	0.00488248083940414	-0.258672627	0.079	0.069	1	no change
8821	8821	RP11-407N17.5	0.00492482161793653	-0.646349657	0.01	0.012	1	no change
8822	8822	NLGN2	0.00496108806832661	-0.27591476	0.016	0.013	1	no change

8823	8823	AC093323.3	0.0049613455793088	-0.470848723	0.096	0.085	1	no change
8824	8824	NR1H4	0.00496844662804387	-0.781888278	0.02	0.023	1	no change
8825	8825	RP11-160O5.1	0.00506151934995826	-0.11327064	0.078	0.07	1	no change
8826	8826	CD93	0.00506980899613347	-1.139146304	0.007	0.01	1	down
8827	8827	RASSF8-AS1	0.0052050730075451	-0.625683068	0.018	0.014	1	no change
8828	8828	PPIC	0.00521203546253898	-0.20726096	0.6	0.512	1	no change
8829	8829	RP3-508I15.21	0.00525242584715572	-0.30682461	0.021	0.017	1	no change
8830	8830	ZFYVE1	0.00527330839879096	-0.238807501	0.028	0.023	1	no change
8831	8831	KLF2	0.00528022113966882	-0.724539655	0.111	0.098	1	no change
8832	8832	ACBD4	0.00538401346722782	-0.207409958	0.051	0.045	1	no change
8833	8833	UAP1	0.00539335977452738	-1.043229059	0.256	0.244	1	down
8834	8834	ZNF35	0.00543348615667651	-0.153470291	0.035	0.03	1	no change
8835	8835	C1orf210	0.00543724356758595	-0.203829047	0.093	0.083	1	no change
8836	8836	UBOX5	0.00546693867617972	-0.151113221	0.018	0.014	1	no change

8837	8837	KLF7	0.00551416369898089	-0.291029734	0.061	0.054	1	no change
8838	8838	ASPA	0.00556351456597892	-1.039023685	0.017	0.02	1	down
8839	8839	NFASC	0.00562442637627582	-0.903168425	0.009	0.012	1	no change
8840	8840	AGAP4	0.00564425350123142	-0.215794795	0.02	0.016	1	no change
8841	8841	RP11-393I2.4	0.00572700200857638	-0.470387836	0.012	0.015	1	no change
8842	8842	FGF22	0.0057296750355731	-0.475982944	0.011	0.013	1	no change
8843	8843	PI4K2A	0.00573842065184129	-0.278782519	0.058	0.051	1	no change
8844	8844	CTC-250I14.6	0.00575662312277582	-0.765879119	0.012	0.015	1	no change
8845	8845	MYO1F	0.0058146715412353	-0.212019202	0.022	0.018	1	no change
8846	8846	LURAP1	0.00591238461912813	-0.551135306	0.022	0.025	1	no change
8847	8847	MPPE1	0.00591607959419556	-0.258281309	0.173	0.156	1	no change
8848	8848	AC062017.1	0.00601617049623744	-0.605977403	0.028	0.033	1	no change
8849	8849	SAR1A	0.00606492286162987	-0.230524516	0.493	0.426	1	no change
8850	8850	ARMCX4	0.00608158416625602	-0.315683811	0.034	0.029	1	no

								change
8851	8851	CDKN2C	0.00608975763577508	-0.568753501	0.063	0.069	1	no change
8852	8852	FAS	0.00609133727443813	-0.389774278	0.058	0.051	1	no change
8853	8853	SLC9A4	0.00610881026928573	0.134162920460225	0.017	0.014	1	no change
8854	8854	ZEB2	0.00615151540142308	-0.775364944	0.037	0.042	1	no change
8855	8855	ZNF124	0.00616395536413469	-0.158335136	0.067	0.06	1	no change
8856	8856	LINC01239	0.00619284159366723	-0.399449896	0.012	0.009	1	no change
8857	8857	GPR137B	0.00623613107864683	-0.257771995	0.252	0.223	1	no change
8858	8858	U2AF1L4	0.00623875517684866	-0.354513158	0.121	0.109	1	no change
8859	8859	MLLT11	0.00631462811666176	-0.285560734	0.126	0.114	1	no change
8860	8860	C16orf86	0.00637188515835843	-0.889882624	0.01	0.013	1	no change
8861	8861	LTBP2	0.00642227009572389	-0.696685465	0.03	0.025	1	no change
8862	8862	ZNF449	0.0064323775275718	-0.169886835	0.01	0.008	1	no change
8863	8863	DAB2	0.00665055556282412	-0.57272594	0.093	0.099	1	no

								change
8864	8864	CST7	0.00665218694204724	-0.301077718	0.018	0.014	1	no change
8865	8865	MRAS	0.00666650197989745	-0.641727562	0.016	0.019	1	no change
8866	8866	AC013264.2	0.00671610591723637	-0.508653645	0.03	0.035	1	no change
8867	8867	KISS1R	0.00673276131729075	-0.458736735	0.012	0.015	1	no change
8868	8868	LINC01612	0.00678545265859049	-0.313943815	0.126	0.132	1	no change
8869	8869	PKIG	0.00697843816344649	-0.474500338	0.169	0.17	1	no change
8870	8870	NBEA	0.0069791639987561	-0.20612017	0.025	0.021	1	no change
8871	8871	GTF2A2	0.00699548966821992	-0.10650918	0.597	0.499	1	no change
8872	8872	FOXO3	0.00700441322190661	-0.447730584	0.27	0.236	1	no change
8873	8873	STX12	0.00702968840706158	-0.28341802	0.274	0.242	1	no change
8874	8874	CCDC69	0.00710749441352929	-0.293831368	0.08	0.072	1	no change
8875	8875	BCAS4	0.00716578672442789	-0.264702553	0.143	0.127	1	no change
8876	8876	ARL4A	0.00717654398681733	-0.285719502	0.336	0.297	1	no

								change
8877	8877	SH3TC2	0.00719556357990662	-0.37830944	0.016	0.019	1	no change
8878	8878	MAP3K14	0.00734608562567967	-0.300325494	0.025	0.021	1	no change
8879	8879	ALOX5AP	0.00736187127740837	-0.418555822	0.041	0.046	1	no change
8880	8880	RANGRF	0.00738997596220341	-0.224592894	0.21	0.189	1	no change
8881	8881	ZNF423	0.00743223322758893	-0.556504693	0.013	0.01	1	no change
8882	8882	FAM89A	0.00745093244854835	-0.464546832	0.051	0.056	1	no change
8883	8883	RP11-350J20.12	0.00748538444856786	-0.458973628	0.037	0.032	1	no change
8884	8884	PPM1M	0.00749262962869856	-0.25833364	0.04	0.034	1	no change
8885	8885	C17orf100	0.00752189702497432	-0.204230385	0.026	0.022	1	no change
8886	8886	IGSF6	0.00752546669248603	-0.92531712	0.011	0.014	1	no change
8887	8887	TANGO2	0.00752686845077691	-0.262704494	0.047	0.041	1	no change
8888	8888	RP11-568N6.1	0.00753128131611414	-0.713342658	0.008	0.01	1	no change
8889	8889	RP11-490O6.2	0.00756237230260584	-0.204178789	0.01	0.007	1	no

								change
8890	8890	ANXA5	0.00756415941102149	-0.762839359	0.267	0.254	1	no change
8891	8891	RWDD2A	0.00759715375615524	-0.277099871	0.02	0.016	1	no change
8892	8892	TAF13	0.00761087450734145	-0.347398915	0.118	0.106	1	no change
8893	8893	PHACTR1	0.00772999218257333	-0.526341067	0.018	0.015	1	no change
8894	8894	RSPH1	0.00775847247366162	-0.759725037	0.022	0.026	1	no change
8895	8895	DSEL	0.00783430350620329	-0.56534509	0.025	0.02	1	no change
8896	8896	INAFM2	0.00783544748436806	-0.183077077	0.027	0.023	1	no change
8897	8897	RP5-882C2.2	0.00785559278198769	-0.245298324	0.07	0.063	1	no change
8898	8898	ESAM	0.00792486112253766	-0.467847363	0.017	0.014	1	no change
8899	8899	CLCN7	0.00798749781596235	-0.285813559	0.072	0.065	1	no change
8900	8900	C20orf195	0.00802450369479407	-0.126600976	0.016	0.013	1	no change
8901	8901	PBX4	0.008055955012023	-0.276247613	0.024	0.02	1	no change
8902	8902	CKS2	0.00805930530866123	-0.308417751	0.518	0.445	1	no

								change
8903	8903	KIT	0.00816708936994151	-0.305251317	0.034	0.038	1	no change
8904	8904	HIST1H2BG	0.00824445241573466	-0.381729395	0.049	0.043	1	no change
8905	8905	RP11-467D6.1	0.00825986806148507	-0.570576633	0.011	0.013	1	no change
8906	8906	CFI	0.00832832888224082	-0.64481654	0.019	0.023	1	no change
8907	8907	RP11-327P2.5	0.00832862282764318	-0.540577327	0.037	0.042	1	no change
8908	8908	RP11-342M1.3	0.00838603410452938	-0.793760518	0.011	0.014	1	no change
8909	8909	PALLD	0.00840136285532447	-0.646134376	0.144	0.128	1	no change
8910	8910	RP11-109G23.3	0.00848712121661266	-0.878093783	0.009	0.011	1	no change
8911	8911	ABHD3	0.00853709983016068	-0.30299606	0.167	0.15	1	no change
8912	8912	HIST1H2BC	0.00864975198973621	-0.187159352	0.033	0.028	1	no change
8913	8913	PTPN21	0.00865632665266712	-0.531663541	0.038	0.042	1	no change
8914	8914	IZUMO4	0.0086569956612493	-0.574116639	0.036	0.041	1	no change
8915	8915	TRAFD1	0.00873978448334651	-0.230844197	0.118	0.107	1	no

								change
8916	8916	SYP	0.00884494886810215	-0.150955837	0.024	0.02	1	no change
8917	8917	C7orf73	0.00886881571481706	-0.144556913	0.695	0.604	1	no change
8918	8918	AMY2B	0.00894948789562733	-0.271623451	0.01	0.008	1	no change
8919	8919	MZB1	0.00896807975851113	-2.052358357	0.009	0.011	1	down
8920	8920	SCIN	0.00900774579374084	-0.116834152	0.066	0.059	1	no change
8921	8921	APOBEC3F	0.00906329221823653	-0.112826021	0.043	0.038	1	no change
8922	8922	C11orf71	0.00906939471933703	-0.190183995	0.068	0.061	1	no change
8923	8923	LCA5L	0.00921200518410609	-0.145593087	0.011	0.008	1	no change
8924	8924	TSTD3	0.00933789800380342	-0.181604124	0.055	0.049	1	no change
8925	8925	OLMALINC	0.00933962413729231	-0.662558139	0.01	0.013	1	no change
8926	8926	C14orf132	0.00941198735517139	-1.021496983	0.015	0.019	1	down
8927	8927	S1PR2	0.00945395330554388	-0.867531876	0.042	0.046	1	no change
8928	8928	LINC00853	0.0094807861781178	-0.303516084	0.013	0.016	1	no change
8929	8929	AC007950.2	0.00949102053034963	-0.76784984	0.008	0.01	1	no

								change
8930	8930	XYLT1	0.00967631513164526	-0.207387388	0.021	0.017	1	no change
8931	8931	STX2	0.00969260582274419	-1.265473103	0.01	0.012	1	down
8932	8932	LY86	0.00974190091392737	-1.050137712	0.011	0.014	1	down
8933	8933	NOXO1	0.00976326366550612	-0.320883814	0.019	0.022	1	no change
8934	8934	CPPED1	0.00983246806601952	-0.167734307	0.032	0.028	1	no change
8935	8935	MMRN2	0.00984780980512271	-0.122782969	0.104	0.095	1	no change
8936	8936	ETHE1	0.009879504570893	-0.176710402	0.498	0.427	1	no change
8937	8937	IGSF22	0.00993542982843952	-0.248026658	0.06	0.053	1	no change
8938	8938	RP11-420L9.5	0.009935711229748	-0.784630856	0.013	0.016	1	no change
8939	8939	CAPN3	0.00999048961725956	-0.275422426	0.01	0.008	1	no change
8940	8940	CTD-2619J13.13	0.0101263248204605	-0.581917893	0.009	0.011	1	no change
8941	8941	OSTM1	0.0102225550197162	-0.3355332	0.105	0.095	1	no change
8942	8942	LINC01220	0.0102669424957983	-0.254125416	0.016	0.013	1	no change
8943	8943	SFT2D1	0.0103566840821273	-0.172399706	0.669	0.575	1	no

								change
8944	8944	ACACA	0.0103940270683804	-0.255255859	0.117	0.106	1	no change
8945	8945	SRGAP2	0.0104885569247909	-0.309531229	0.043	0.037	1	no change
8946	8946	RP11-81A22.5	0.0104906870856379	-0.241064093	0.013	0.01	1	no change
8947	8947	ZNF595	0.0104998658634234	-0.495828779	0.039	0.034	1	no change
8948	8948	SAA1	0.0106985615098727	-1.591435494	0.01	0.012	1	down
8949	8949	COG3	0.0107701339501697	-0.246388925	0.134	0.122	1	no change
8950	8950	AHNAK2	0.0107849145301673	-0.798533433	0.028	0.032	1	no change
8951	8951	NLRX1	0.0110082176441585	-0.161682569	0.023	0.019	1	no change
8952	8952	NKG7	0.0110370751412464	-0.120161496	0.016	0.013	1	no change
8953	8953	RP5-1180E21.5	0.0111311975754867	-0.305712628	0.022	0.019	1	no change
8954	8954	PLPP1	0.0111828191070598	-0.211830895	0.491	0.418	1	no change
8955	8955	HYAL2	0.0112177142215254	-0.249948168	0.162	0.147	1	no change
8956	8956	LINC01451	0.0112222903800176	-0.341315334	0.061	0.054	1	no change

8957	8957	LPIN2	0.0112654503020783	-0.245024516	0.073	0.066	1	no change
8958	8958	DEGS1	0.0112938549595284	-0.622732068	0.071	0.075	1	no change
8959	8959	NCALD	0.0113085310998306	-0.735480901	0.017	0.02	1	no change
8960	8960	TNFRSF1B	0.0114069553076209	-0.805262593	0.02	0.024	1	no change
8961	8961	ARHGAP24	0.0114325372055643	-0.331657348	0.01	0.008	1	no change
8962	8962	ORAI3	0.0114460360655751	-0.186286795	0.153	0.139	1	no change
8963	8963	B3GNT9	0.0114725379332858	-0.203336341	0.053	0.047	1	no change
8964	8964	RP11-290F5.2	0.0114823319182576	-0.178495841	0.011	0.009	1	no change
8965	8965	DHRS2	0.0114871184561093	-0.139074641	0.838	0.757	1	no change
8966	8966	RAB23	0.0115860872532487	-0.698544715	0.042	0.046	1	no change
8967	8967	PTK6	0.0116820314273421	-0.211988793	0.191	0.175	1	no change
8968	8968	RNASE6	0.0116951227120839	-1.14935585	0.018	0.021	1	down
8969	8969	VPS28	0.0117066423829735	-0.121989533	0.665	0.565	1	no change
8970	8970	SLC22A18AS	0.0117105350888591	-0.474855417	0.031	0.035	1	no

8971	8971	PCSK5	0.0118361161732263	-1.188953435	0.014	0.017	1	change down
8972	8972	PLCXD1	0.0119035837899501	-0.406101185	0.063	0.068	1	no change
8973	8973	RP11-486L19.2	0.0119086610716487	-0.372625995	0.02	0.023	1	no change
8974	8974	NAALADL1	0.0119893665090144	-0.795142405	0.017	0.02	1	no change
8975	8975	NFKB2	0.0120438277051421	-0.321598967	0.123	0.111	1	no change
8976	8976	TRIM11	0.0122171649393073	-0.240258172	0.109	0.099	1	no change
8977	8977	CTD-2314B22.1	0.0122890776205365	-0.328882225	0.063	0.056	1	no change
8978	8978	BBS5	0.0123005330937709	-0.144839471	0.01	0.008	1	no change
8979	8979	SGPP2	0.0123294437050675	-0.209628233	0.068	0.061	1	no change
8980	8980	MOK	0.0123511408774718	-0.353333106	0.032	0.027	1	no change
8981	8981	SCCPDH	0.0123554490375828	-0.209580599	0.76	0.663	1	no change
8982	8982	AL592183.1	0.0124269433873287	-0.411615585	0.02	0.024	1	no change
8983	8983	HSD3B7	0.0124338547946473	-0.551141644	0.039	0.034	1	no change

8984	8984	VAT1	0.0124659243223472	-0.394873018	0.23	0.224	1	no change
8985	8985	BCO1	0.0125703280929643	-0.641585381	0.007	0.01	1	no change
8986	8986	AC116366.6	0.0126154199851447	-0.923221588	0.015	0.017	1	no change
8987	8987	PLXNC1	0.0126503799641739	-0.299758538	0.011	0.008	1	no change
8988	8988	ABHD12	0.012824589524017	-0.124507063	0.472	0.413	1	no change
8989	8989	DDX60	0.0128928367464053	-0.257379297	0.055	0.049	1	no change
8990	8990	PAQR5	0.0129226800317168	-0.835454107	0.015	0.018	1	no change
8991	8991	MRPL57	0.0129497423664155	-0.126078841	0.744	0.644	1	no change
8992	8992	COX7B2	0.0130035418438684	-0.619679235	0.024	0.028	1	no change
8993	8993	SLC1A3	0.0130267380148414	-0.848114476	0.017	0.02	1	no change
8994	8994	C6orf120	0.0130465406810961	-0.2390489	0.104	0.094	1	no change
8995	8995	NAP1L5	0.0130798922666156	-0.356732752	0.031	0.027	1	no change
8996	8996	GJB4	0.0131072557809314	-0.548367513	0.107	0.11	1	no change

8997	8997	ABCD1	0.0131695343995636	-0.150371496	0.027	0.023	1	no change
8998	8998	PTPRC	0.0133051144698562	-0.109426818	0.029	0.025	1	no change
8999	8999	AL358852.1	0.0133281121437525	-0.528928115	0.011	0.014	1	no change
9000	9000	AC007009.1	0.0133787965739788	-0.390107318	0.011	0.014	1	no change
9001	9001	ST3GAL3	0.0133997852844102	-0.248343419	0.017	0.014	1	no change
9002	9002	AGR3	0.0134263405446305	-0.482196913	0.111	0.117	1	no change
9003	9003	LINC01003	0.0134757789182789	-0.47727894	0.073	0.078	1	no change
9004	9004	EGR2	0.0135428883266427	-0.73117793	0.068	0.072	1	no change
9005	9005	ELL	0.0136320748413135	-0.286479183	0.077	0.069	1	no change
9006	9006	TMTC1	0.0136625471961971	-0.974100429	0.012	0.014	1	no change
9007	9007	STARD13	0.013799182579184	-0.503477522	0.018	0.014	1	no change
9008	9008	RP11-96C23.15	0.0138641697133868	-0.432050764	0.017	0.02	1	no change
9009	9009	CCDC84	0.0139216615262704	-0.185746076	0.073	0.066	1	no change

9010	9010	COL18A1	0.0139342609592923	-0.663350636	0.031	0.034	1	no change
9011	9011	ARF4-AS1	0.013951788719377	-0.273283135	0.022	0.019	1	no change
9012	9012	VPS37C	0.0142798768901109	-0.234541684	0.067	0.06	1	no change
9013	9013	B3GNT3	0.0142933034529494	-0.372301708	0.131	0.135	1	no change
9014	9014	DUSP23	0.0143559984596458	-0.228779458	0.565	0.498	1	no change
9015	9015	RP11-542K23.7	0.0144870845144875	-0.17904243	0.012	0.009	1	no change
9016	9016	ZNF597	0.0145861250177441	-0.283867027	0.018	0.015	1	no change
9017	9017	IL7R	0.0150067461981826	-0.242699062	0.018	0.015	1	no change
9018	9018	PANX1	0.0150411699596455	-0.338361716	0.058	0.051	1	no change
9019	9019	GOLGA6L9	0.0151403098032946	-0.239801607	0.017	0.014	1	no change
9020	9020	ARHGAP29	0.015178662831212	-0.384151047	0.087	0.078	1	no change
9021	9021	NANOS3	0.0152276979352232	-0.482684704	0.025	0.028	1	no change
9022	9022	ATF5	0.0153395901325937	-0.26668646	0.142	0.129	1	no change

9023	9023	SLC26A6	0.0155444662652765	-0.201543603	0.018	0.015	1	no change
9024	9024	ENTPD5	0.0155500326954234	-0.266367184	0.087	0.078	1	no change
9025	9025	ANK1	0.0156168694481078	-0.695285467	0.012	0.015	1	no change
9026	9026	RP11-295M3.4	0.0156970079473874	-0.387792683	0.056	0.061	1	no change
9027	9027	ZNF473	0.0157355041288761	-0.247848405	0.015	0.012	1	no change
9028	9028	TRIB3	0.0157944798307348	-0.4943181	0.046	0.051	1	no change
9029	9029	PLAUR	0.0158706633122715	-0.312035973	0.399	0.384	1	no change
9030	9030	CYP4F3	0.0160054872869525	-0.232903045	0.064	0.058	1	no change
9031	9031	TMEM50B	0.0160166356790454	-0.250398636	0.559	0.499	1	no change
9032	9032	DHX58	0.0161391992200586	-0.264452706	0.042	0.037	1	no change
9033	9033	RP11-293A21.1	0.0161700369670904	-0.212393672	0.028	0.024	1	no change
9034	9034	DCUN1D3	0.0162142214121353	-0.395957311	0.067	0.06	1	no change
9035	9035	LGALSL	0.0162515549661149	-0.369680348	0.086	0.078	1	no change

9036	9036	FYN	0.0163247933007654	-0.848936235	0.022	0.026	1	no change
9037	9037	AMOTL1	0.016336498366143	-0.249177992	0.03	0.026	1	no change
9038	9038	ATP6V0D1	0.0163762903814898	-0.242564591	0.357	0.314	1	no change
9039	9039	AREG	0.0164023792109203	-0.203567349	0.393	0.361	1	no change
9040	9040	FAM45A	0.0166641816889481	-0.300028785	0.295	0.258	1	no change
9041	9041	TPM1	0.0166712129508406	-0.527903674	0.425	0.383	1	no change
9042	9042	DUSP2	0.0167006645696739	-0.417133054	0.596	0.553	1	no change
9043	9043	LINC00998	0.0167303171194899	-0.109802745	0.691	0.586	1	no change
9044	9044	NDUFB2-AS1	0.0167424695520999	-0.178663177	0.014	0.011	1	no change
9045	9045	RP11-511B23.2	0.0167608544608188	-0.165129821	0.027	0.024	1	no change
9046	9046	PXDC1	0.0167678179140188	-0.671713937	0.091	0.095	1	no change
9047	9047	CCDC68	0.0169575272855856	-0.582349644	0.043	0.047	1	no change
9048	9048	FBXO30	0.0170079235462491	-0.346511687	0.036	0.031	1	no change

9049	9049	ATP8A1	0.0170507763404884	-0.156523705	0.018	0.015	1	no change
9050	9050	VCL	0.0170793544060763	-0.247448439	0.252	0.227	1	no change
9051	9051	LAT2	0.0170872257167927	-0.821797781	0.008	0.01	1	no change
9052	9052	FURIN	0.0171369038599141	-0.289843167	0.088	0.08	1	no change
9053	9053	ANKRD9	0.0171468337808277	-0.340383853	0.178	0.16	1	no change
9054	9054	OLR1	0.0172403309919087	-1.168123411	0.014	0.017	1	down
9055	9055	RP4-697K14.15	0.0173353895987094	-0.140040498	0.013	0.01	1	no change
9056	9056	IRF9	0.017381904459896	-0.368304075	0.096	0.087	1	no change
9057	9057	RP11-245D16.4	0.0175093779638902	-0.135446093	0.03	0.026	1	no change
9058	9058	SIRT2	0.0175742939520769	-0.27092375	0.194	0.175	1	no change
9059	9059	COL7A1	0.0175959999240905	-0.384849478	0.042	0.037	1	no change
9060	9060	VIPR1	0.0176111905190216	-0.291872788	0.075	0.068	1	no change
9061	9061	TBX6	0.0177225715177637	-0.410476013	0.07	0.075	1	no change
9062	9062	SELENBP1	0.0177706039618581	-0.392841117	0.143	0.147	1	no

								change
9063	9063	GANC	0.0178672990038997	-0.257051501	0.049	0.044	1	no change
9064	9064	PTP4A1	0.0179933291485823	-0.179997919	0.5	0.432	1	no change
9065	9065	PLEKHO1	0.0180048653763717	-0.497821018	0.054	0.048	1	no change
9066	9066	STK40	0.0181296580548955	-0.317861172	0.074	0.067	1	no change
9067	9067	BET1	0.018156336441501	-0.220850235	0.272	0.244	1	no change
9068	9068	SOX9	0.0181936719343318	-0.572598848	0.074	0.079	1	no change
9069	9069	ANKRD37	0.0184471432697402	-0.455672389	0.315	0.283	1	no change
9070	9070	HSD11B2	0.0185142075365499	-0.127536229	0.014	0.012	1	no change
9071	9071	AMOTL2	0.018544637158033	-0.559917656	0.073	0.078	1	no change
9072	9072	NEURL2	0.0185976012172321	-0.223190694	0.021	0.017	1	no change
9073	9073	AC016700.5	0.0188310418066336	-0.102099252	0.012	0.009	1	no change
9074	9074	KCP	0.0188944022318037	-0.274244571	0.042	0.037	1	no change
9075	9075	NRP1	0.0189074102294527	-0.733671058	0.023	0.019	1	no

								change
9076	9076	METRNL	0.0189386168198239	-0.431571812	0.204	0.2	1	no change
9077	9077	RP11-79P5.9	0.019289634603673	-0.109829148	0.01	0.008	1	no change
9078	9078	ZNF790	0.0194295137261695	-0.27046587	0.048	0.043	1	no change
9079	9079	TMEM135	0.0194378016438936	-0.461414354	0.067	0.072	1	no change
9080	9080	BAIAP3	0.0195026678247682	-0.123988444	0.022	0.018	1	no change
9081	9081	RAB11FIP3	0.0195405139566468	-0.278868524	0.013	0.011	1	no change
9082	9082	CTD-2538C1.2	0.0195536330753032	-0.198916933	0.019	0.016	1	no change
9083	9083	ARHGEF25	0.019698120080485	-0.479532392	0.014	0.011	1	no change
9084	9084	ANKRD36B	0.0197179409120004	-0.172572926	0.014	0.011	1	no change
9085	9085	SLC25A35	0.0198317662751637	-0.527796045	0.023	0.026	1	no change
9086	9086	CYP27B1	0.0202863533147777	-0.371684689	0.047	0.041	1	no change
9087	9087	GREM1	0.0203743722984763	-0.996484248	0.01	0.013	1	no change
9088	9088	SPHK2	0.0204722968752565	-0.465859611	0.061	0.066	1	no

								change
9089	9089	DCBLD2	0.0209785915735238	-0.288315946	0.04	0.035	1	no change
9090	9090	IQGAP2	0.0210048914885126	-0.566907105	0.017	0.014	1	no change
9091	9091	DDX47	0.0210580317283115	-0.19600706	0.013	0.011	1	no change
9092	9092	POMGNT2	0.0210602978781179	-0.172511787	0.046	0.041	1	no change
9093	9093	CXCL13	0.0210985839775361	-0.288474552	0.013	0.011	1	no change
9094	9094	HERC6	0.0212011834348886	-0.267569725	0.044	0.039	1	no change
9095	9095	FAM214B	0.0213774533902194	-0.460636828	0.045	0.049	1	no change
9096	9096	DAG1	0.0214266093979096	-0.237314366	0.14	0.128	1	no change
9097	9097	TLR5	0.0215755842648618	-0.27060367	0.044	0.039	1	no change
9098	9098	RNF144B	0.0216907018927478	-0.454361481	0.065	0.07	1	no change
9099	9099	B3GNT8	0.0217063481618858	-0.411448543	0.04	0.044	1	no change
9100	9100	RP11-499E18.1	0.0217767245593129	-0.220073437	0.017	0.014	1	no change
9101	9101	BORCS6	0.0217894671307041	-0.360364891	0.057	0.051	1	no

								change
9102	9102	FERMT3	0.0221598520085007	-0.155432629	0.026	0.022	1	no change
9103	9103	AC002467.7	0.022175854139049	-0.234946195	0.049	0.044	1	no change
9104	9104	KRT4	0.0223216343289325	-0.768613256	0.019	0.022	1	no change
9105	9105	RBCK1	0.022516527023481	-0.179171809	0.55	0.466	1	no change
9106	9106	DUSP8	0.0225271741812539	-0.354230733	0.015	0.013	1	no change
9107	9107	ZNF438	0.0226443429255537	-0.411382362	0.016	0.014	1	no change
9108	9108	ERF	0.022954251713766	-0.511464272	0.198	0.193	1	no change
9109	9109	NDUFAF3	0.0233415681101859	-0.215212658	0.552	0.49	1	no change
9110	9110	TIMM10B	0.0233434033631777	-0.30666086	0.172	0.156	1	no change
9111	9111	ANXA2R	0.0233464307150137	-0.594177781	0.024	0.028	1	no change
9112	9112	ZNF761	0.023470739626707	-0.600170689	0.047	0.051	1	no change
9113	9113	RABIF	0.0235068832693764	-0.268787632	0.23	0.208	1	no change
9114	9114	ABCA7	0.0236154951263053	-0.223768774	0.032	0.028	1	no

								change
9115	9115	ADGRD1	0.023658681887195	-1.220808465	0.009	0.012	1	down
9116	9116	MREG	0.0238640155059314	-0.228912204	0.057	0.052	1	no change
9117	9117	C16orf46	0.0239326885164435	-0.134469841	0.025	0.022	1	no change
9118	9118	CTNNB1	0.0239569929338268	-0.344599377	0.55	0.48	1	no change
9119	9119	RAB8B	0.0240144831462918	-0.437468875	0.133	0.12	1	no change
9120	9120	FAM47E	0.0240601685858706	-0.216589812	0.033	0.029	1	no change
9121	9121	CSF1R	0.0241265054791232	-1.046601294	0.009	0.011	1	down
9122	9122	XKR9	0.0241654684407543	-0.167159357	0.019	0.016	1	no change
9123	9123	GATSL3	0.0242501986019787	-0.31451998	0.043	0.038	1	no change
9124	9124	CTD-3065J16.9	0.0246188342539487	-0.182668243	0.02	0.017	1	no change
9125	9125	KLHDC8B	0.0246458550586123	-0.549006495	0.058	0.062	1	no change
9126	9126	TUSC2	0.0247556697386848	-0.298054502	0.364	0.339	1	no change
9127	9127	NDUFV2-AS1	0.0247798729582323	-0.105743832	0.027	0.024	1	no change
9128	9128	LAMP1	0.0249020345441247	-0.187417481	0.555	0.471	1	no

								change
9129	9129	RASSF2	0.0249442481735607	-0.626515921	0.014	0.012	1	no change
9130	9130	ELOVL1	0.0251394886799333	-0.215202389	0.349	0.307	1	no change
9131	9131	CEACAM1	0.0251450064929241	-0.423464866	0.078	0.082	1	no change
9132	9132	HES4	0.0251868419939287	-0.104493991	0.733	0.638	1	no change
9133	9133	BAG2	0.0251868590537103	-1.10829959	0.039	0.042	1	down
9134	9134	MX2	0.0252815245551588	-0.449203433	0.225	0.2	1	no change
9135	9135	GPR35	0.0253268403502221	-0.627397893	0.01	0.012	1	no change
9136	9136	S100B	0.0253271156455669	0.909332134658551	0.014	0.011	1	no change
9137	9137	LPIN1	0.0257462212352547	-0.257091659	0.06	0.054	1	no change
9138	9138	FAM63A	0.0259597919143506	-0.385544879	0.054	0.058	1	no change
9139	9139	RHOC	0.0259917290419576	-0.276874047	0.538	0.48	1	no change
9140	9140	ABHD4	0.0262480018705139	-0.501556941	0.049	0.053	1	no change
9141	9141	KLHDC7A	0.026497182396627	-0.155108566	0.044	0.04	1	no change

9142	9142	SLC46A1	0.0269263196987693	-0.233700803	0.02	0.017	1	no change
9143	9143	UBTD1	0.0271174614973862	-0.455830783	0.06	0.065	1	no change
9144	9144	ALOX15	0.027268532191069	-0.408466382	0.012	0.014	1	no change
9145	9145	MAFB	0.0273053017216885	-0.694128509	0.153	0.152	1	no change
9146	9146	AGFG2	0.0274249966721208	-0.581679174	0.038	0.041	1	no change
9147	9147	CP	0.0276546994243035	-0.564835162	0.051	0.055	1	no change
9148	9148	PHF11	0.027699554279944	-0.26373469	0.196	0.178	1	no change
9149	9149	LAMTOR3	0.0277333595220832	-0.25366383	0.329	0.29	1	no change
9150	9150	RNF139-AS1	0.0278581171412692	-0.203568913	0.023	0.02	1	no change
9151	9151	DSE	0.0279355452879743	-0.491556885	0.052	0.046	1	no change
9152	9152	FOXN3-AS1	0.0281695670364411	-0.648896465	0.016	0.019	1	no change
9153	9153	APBB1IP	0.0283878284583214	-0.911365458	0.008	0.01	1	no change
9154	9154	DDIT4	0.0284773635622831	-0.460944564	0.634	0.575	1	no change

9155	9155	TMEM55B	0.028630880302749	-0.295584135	0.173	0.157	1	no change
9156	9156	CCDC107	0.0286851049597451	-0.426112047	0.205	0.202	1	no change
9157	9157	RP11-108M9.4	0.0286959411998154	-0.380311816	0.164	0.165	1	no change
9158	9158	RP1-315G1.3	0.0288390370927674	0.11040116316861	0.013	0.01	1	no change
9159	9159	EPDR1	0.0289754554861057	-0.64313001	0.02	0.022	1	no change
9160	9160	APOBEC3A	0.0289916197250409	-0.45475004	0.03	0.026	1	no change
9161	9161	TRIM72	0.0292216266278101	-0.145268006	0.012	0.01	1	no change
9162	9162	CABLES1	0.0292937903916557	-0.239767185	0.017	0.014	1	no change
9163	9163	IFT172	0.0293220795177599	-0.33585915	0.105	0.095	1	no change
9164	9164	TINCR	0.0294048972439314	-0.393897043	0.046	0.051	1	no change
9165	9165	RP11-1260E13.1	0.0296027784261797	-0.157298734	0.023	0.02	1	no change
9166	9166	TRAM1	0.0297493330133789	-0.148523755	0.632	0.534	1	no change
9167	9167	ARL4D	0.0299205056755662	-0.549054851	0.227	0.219	1	no change

9168	9168	EPS8L3	0.0303159450987125	-0.576827799	0.011	0.014	1	no change
9169	9169	CTC1	0.0303490618497975	-0.241589644	0.016	0.013	1	no change
9170	9170	LGALS7B	0.0305203630438944	-0.284020511	0.032	0.035	1	no change
9171	9171	CADM4	0.0306168963325405	-0.224206863	0.027	0.024	1	no change
9172	9172	RNASE4	0.030820340462133	-0.852576729	0.012	0.014	1	no change
9173	9173	SLC22A15	0.0312701913023421	-0.798077792	0.008	0.01	1	no change
9174	9174	LINC00525	0.0312854229934901	-0.629958608	0.01	0.012	1	no change
9175	9175	FUT6	0.0313764644584943	-0.539560845	0.008	0.01	1	no change
9176	9176	GFOD1	0.0314414243389144	-0.460704673	0.022	0.019	1	no change
9177	9177	GPLD1	0.0315418770848736	-0.296452578	0.027	0.024	1	no change
9178	9178	EVA1B	0.0316382439698781	-0.558675094	0.064	0.068	1	no change
9179	9179	LINC01209	0.0318705616183083	-0.198568119	0.01	0.008	1	no change
9180	9180	RWDD1	0.0319239440272167	-0.102846876	0.702	0.602	1	no change

9181	9181	AGTR1	0.0321090439079348	-0.970417256	0.013	0.015		1	no change
9182	9182	MANF	0.0321461640249366	-0.232862799	0.535	0.481		1	no change
9183	9183	RP11-128A17.1	0.0323086544110311	-0.560085852	0.028	0.032		1	no change
9184	9184	NCKAP5	0.0323642018461648	-0.222567682	0.018	0.015		1	no change
9185	9185	FMNL1	0.0324237145347892	-0.303996203	0.02	0.017		1	no change
9186	9186	MORN4	0.0329718398872199	-0.195413153	0.062	0.056		1	no change
9187	9187	ICA1	0.0330028350859322	-0.31919125	0.344	0.32		1	no change
9188	9188	NOP14-AS1	0.0332238755494419	-0.310812223	0.078	0.071		1	no change
9189	9189	AC058791.1	0.0333840859676346	-0.241303943	0.02	0.017		1	no change
9190	9190	RP3-402G11.26	0.0334544854297347	-0.53660325	0.008	0.01		1	no change
9191	9191	TBCEL	0.0336232498120704	-0.166351334	0.018	0.015		1	no change
9192	9192	TTC9	0.0337086037234752	-0.134052865	0.036	0.032		1	no change
9193	9193	SRI	0.0337419656364237	-0.164120364	0.858	0.765		1	no change

9194	9194	CDR2L	0.0337495052598449	-0.437518253	0.065	0.069	1	no change
9195	9195	C6orf1	0.0337838885507838	-0.348429051	0.311	0.292	1	no change
9196	9196	AC002451.3	0.0340098261540443	-0.153332333	0.011	0.009	1	no change
9197	9197	NUDT14	0.034203695324579	-0.2050726	0.375	0.329	1	no change
9198	9198	IGHG2	0.0342971617688652	-3.241583377	0.008	0.01	1	down
9199	9199	ROBO3	0.0345397139859974	-0.625860471	0.015	0.013	1	no change
9200	9200	LRIF1	0.0346086553235124	-0.336479698	0.18	0.164	1	no change
9201	9201	PALMD	0.0348029635775164	-0.195428963	0.032	0.029	1	no change
9202	9202	ZFAND4	0.0355354581494179	-0.196437268	0.015	0.013	1	no change
9203	9203	C16orf45	0.0355391125030112	-0.333978507	0.105	0.095	1	no change
9204	9204	MAN1B1-AS1	0.0357559961495865	-0.295586943	0.02	0.017	1	no change
9205	9205	FMO4	0.0362274651951877	-0.228754177	0.014	0.011	1	no change
9206	9206	HAAO	0.0362620766608111	-0.666067784	0.022	0.025	1	no change
9207	9207	RP11-156E8.1	0.0364351820906369	-0.398621174	0.012	0.014	1	no

								change
9208	9208	ZCCHC24	0.0365937943981139	-0.518009215	0.027	0.023	1	no change
9209	9209	STARD4	0.0367884193723088	-0.669826988	0.019	0.022	1	no change
9210	9210	RIN2	0.0369428481021611	-0.396372591	0.303	0.271	1	no change
9211	9211	CD48	0.0372840322212048	-0.28164833	0.014	0.012	1	no change
9212	9212	HES2	0.0373461786746718	-0.496144274	0.03	0.034	1	no change
9213	9213	LOXL1	0.0374334355180283	-0.150402438	0.114	0.106	1	no change
9214	9214	AQP7	0.0375180223582319	-0.156050984	0.025	0.022	1	no change
9215	9215	SLC39A13	0.0377427063754319	-0.468093603	0.072	0.065	1	no change
9216	9216	IFT140	0.0379427185759791	-0.216078721	0.13	0.12	1	no change
9217	9217	TCEB1	0.0380390766986135	-0.117984575	0.752	0.654	1	no change
9218	9218	LRFN2	0.0380472352276295	0.120343059023898	0.01	0.008	1	no change
9219	9219	FNIP1	0.038163375360074	-0.308350936	0.251	0.226	1	no change
9220	9220	CYP2U1	0.0382355915937955	-0.575110908	0.016	0.014	1	no

								change
9221	9221	GATA3-AS1	0.0386384414910532	-0.271528985	0.231	0.208	1	no change
9222	9222	PLAG1	0.0386874700914992	-0.438209117	0.032	0.035	1	no change
9223	9223	FUT1	0.038695163256739	-0.275916502	0.01	0.008	1	no change
9224	9224	MATN1-AS1	0.0392622368166097	-0.19412367	0.011	0.009	1	no change
9225	9225	AP5Z1	0.0393610541471921	-0.275009811	0.057	0.051	1	no change
9226	9226	RAB24	0.0396748644315967	-0.22327282	0.024	0.021	1	no change
9227	9227	MANBA	0.0397515397686571	-0.253380028	0.072	0.065	1	no change
9228	9228	GMPR	0.0398190847365821	-0.535407081	0.042	0.045	1	no change
9229	9229	ZNF844	0.0400031283756835	-0.370056758	0.034	0.031	1	no change
9230	9230	ADAD2	0.0403081880874056	-0.491305839	0.02	0.023	1	no change
9231	9231	DDX60L	0.0404602375248845	-0.305310513	0.051	0.046	1	no change
9232	9232	PCBP4	0.0407089394755457	-0.200691103	0.087	0.08	1	no change
9233	9233	SPSB1	0.0407833393720581	-0.504706925	0.21	0.204	1	no

change

9234	9234	PPP1R18	0.0408100433292118	-0.356179229	0.079	0.072	1	no change
9235	9235	RINL	0.0409832249549983	-0.330838674	0.013	0.011	1	no change
9236	9236	RTN2	0.0412720570763939	-0.313526502	0.051	0.046	1	no change
9237	9237	FSTL1	0.0412861181043	-1.01813255	0.145	0.128	1	down
9238	9238	OSR1	0.0413382589621429	-0.359521317	0.124	0.114	1	no change
9239	9239	ARRB1	0.0418983040813258	-0.280395567	0.033	0.03	1	no change
9240	9240	NPC1	0.0419696040334832	-0.304941675	0.047	0.042	1	no change
9241	9241	ATG9B	0.0421915926805099	-0.620744239	0.008	0.01	1	no change
9242	9242	ST3GAL1	0.042306132259292	-0.305784719	0.136	0.125	1	no change
9243	9243	RAB10	0.042404965019962	-0.111985431	0.681	0.576	1	no change
9244	9244	FHOD1	0.042496691921569	-0.451768167	0.027	0.023	1	no change
9245	9245	UMAD1	0.0425572090167939	-0.438081822	0.143	0.144	1	no change
9246	9246	BBS12	0.0427703432100176	-0.263569695	0.011	0.009	1	no change

9247	9247	ASH1L-AS1	0.0427901489051677	-0.166263583	0.041	0.037	1	no change
9248	9248	GPC3	0.042932541422152	-0.602060286	0.054	0.048	1	no change
9249	9249	GGT1	0.0431075972467224	-0.288897857	0.01	0.008	1	no change
9250	9250	TINAGL1	0.0431535539728978	-0.127563144	0.622	0.557	1	no change
9251	9251	TMEM191B	0.0432306091854112	-0.275423096	0.065	0.059	1	no change
9252	9252	ZNF341	0.0435602199875281	-0.182208334	0.021	0.018	1	no change
9253	9253	VEGFA	0.0443425799900274	-0.383594263	0.407	0.371	1	no change
9254	9254	MNDA	0.0444909821253891	-0.703498673	0.011	0.013	1	no change
9255	9255	ZNF823	0.044523309533425	-0.274518953	0.053	0.048	1	no change
9256	9256	SLC9B2	0.0445485531906055	-1.133629871	0.009	0.011	1	down
9257	9257	RP11-105N14.1	0.044564216195381	-0.173756827	0.015	0.013	1	no change
9258	9258	SYT14	0.0446738853732553	-0.522921163	0.031	0.034	1	no change
9259	9259	ZYX	0.0446901212348762	-0.325372591	0.231	0.208	1	no change
9260	9260	EGFL7	0.0452043238242865	-0.330391725	0.024	0.021	1	no

								change
9261	9261	DHRS12	0.0453329200916222	-0.290536628	0.098	0.09	1	no change
9262	9262	INPP5J	0.0455417680627674	-0.179135039	0.027	0.024	1	no change
9263	9263	EVI2A	0.0455766571270566	-0.779189021	0.011	0.013	1	no change
9264	9264	RP11-178C3.2	0.0455941233167538	-0.635082324	0.008	0.01	1	no change
9265	9265	CCDC64	0.0457677902154309	-0.220839366	0.019	0.016	1	no change
9266	9266	VWF	0.045985932004732	-1.008532649	0.008	0.01	1	down
9267	9267	ACOT2	0.0466395403997918	-0.110811346	0.024	0.021	1	no change
9268	9268	TBKBP1	0.046697995948558	-0.757089048	0.011	0.013	1	no change
9269	9269	OTUB2	0.0468471332508672	-0.347978078	0.027	0.03	1	no change
9270	9270	UBALD2	0.046870595307525	-0.236334135	0.439	0.403	1	no change
9271	9271	ATP1A1	0.04730863611428	-0.311109915	0.509	0.451	1	no change
9272	9272	PAPSS2	0.0476423625808222	-0.633370308	0.023	0.02	1	no change
9273	9273	KIAA1683	0.0476547498755338	-0.43413749	0.018	0.015	1	no change

9274	9274	RP5-1148A21.3	0.0479300725164929	-0.243046784	0.015	0.013	1	no change
9275	9275	TMEM179B	0.0479922209278661	-0.149184676	0.58	0.492	1	no change
9276	9276	EPHX4	0.0480564909949064	-0.286147496	0.042	0.045	1	no change
9277	9277	UGT1A6	0.0482908429494338	-0.166008464	0.038	0.034	1	no change
9278	9278	LAMA4	0.0483097885563119	-0.933802075	0.028	0.03	1	no change
9279	9279	SLC44A4	0.0483858817889844	-0.224009025	0.199	0.183	1	no change
9280	9280	RP11-431K24.1	0.0483966996311445	-0.635619447	0.02	0.023	1	no change
9281	9281	RP11-66N24.3	0.0485149976892449	-0.185430051	0.016	0.014	1	no change
9282	9282	RP11-792A8.4	0.048761941195199	-0.408460668	0.151	0.152	1	no change
9283	9283	SNX22	0.0488305513211069	-0.107072736	0.012	0.01	1	no change
9284	9284	HOXA11-AS	0.0488336383627455	-0.32705621	0.039	0.042	1	no change
9285	9285	ADTRP	0.0493471103277599	-0.320397934	0.01	0.008	1	no change
9286	9286	THBS3	0.0494809832767231	-0.793181525	0.032	0.035	1	no change

9287	9287	C19orf73	0.0496689337681223	-0.225251971	0.028	0.025	1	no change
9288	9288	GRK5	0.0499392807571223	-1.110743893	0.008	0.01	1	down

Table S4. Lactylation-related survival genes (LRSGs).

	coef	HR	HR_l	HR_H	p_val
A2M	0.177713078425067	1.19448254924855	1.02683479034899	1.38950157695222	0.0212699619549504
AACS	0.164991097757358	1.17938261951441	1.01648108979205	1.36839079170399	0.0295922489995051
AATBC	-0.226947196	0.796962864092048	0.682239595718649	0.930977637075951	0.00421184105936944
ABCA10	-0.171666095	0.842260361443556	0.718120642320314	0.987859803287209	0.0348494551068814
ABCA4	0.184224062422811	1.20228517984912	1.06434053620292	1.35810824122294	0.00304854314775625
ABCB10	0.160759288733883	1.17440224293676	1.00612372078594	1.37082607210324	0.0416165367766629
ABCB8	0.162481144693194	1.17642613636383	1.00612035841944	1.37555953692666	0.0417062911958132
ABCB9	0.204134279141184	1.22646283077521	1.05319824122141	1.4282316627577	0.00861473539308597
ABCC1	0.18526075563405	1.20353222702402	1.02837291723911	1.4085258345525	0.0209653928917749
ABCC3	-0.178452255	0.836563997484914	0.722684505658453	0.968388441163964	0.0168387634719461
ABCF2	0.204065794967395	1.2263788403576	1.04924685530049	1.43341393160157	0.0103482864085319
ABHD4	0.20936787703907	1.23289847015292	1.06082545058127	1.43288289027427	0.00633619230349212
ABL2	0.220241095804541	1.24637719067764	1.07888822050769	1.43986751538586	0.00277854972727215
ACACA	0.16785259106963	1.18276224807895	1.02745042387541	1.3615513731594	0.0194390133231612
ACADS	-0.151982181	0.859003587408767	0.742621019473716	0.99362547494826	0.0407509081013706
ACBD3	0.17427019659874	1.19037715823119	1.01055030115444	1.40220410326908	0.0370182857661705
ACKR3	0.230435000219015	1.25914762030613	1.08235395990205	1.46481907810091	0.00283434135875772
ACLY	0.28086280444102	1.324271907	1.12053083554397	1.56505829977651	0.000983852322380559
ACSL5	-0.231669372	0.793208336989907	0.683096332687254	0.921069892726764	0.00237971674216565

ACSM6	-0.225855095	0.797833703353659	0.675258466479809	0.942659218366197	0.00795886331979406
ACTA2	0.201101791715046	1.22274923123007	1.04943854681902	1.42468149946046	0.00991596686223047
ACTN1	0.192311550217648	1.2120480719888	1.03489125377216	1.41953130191898	0.0170622450680724
ACVR1	0.213985196118732	1.23860431850646	1.05257681896294	1.45750944746663	0.00996403693133594
ADAMTS2	0.167802686718405	1.18270322456909	1.02012954916029	1.3711855700656	0.0261404645958959
ADAMTS4	0.17467088672244	1.1908542261738	1.01397180657447	1.39859291826555	0.0332468422867874
ADAMTSL4	0.202745944784067	1.22476127172927	1.05607900362532	1.42038632297256	0.00732601415520608
ADAMTSL5	0.158358903568615	1.17158660587341	1.0163654990175	1.35051335015686	0.0289752616021233
ADCY3	0.200409700883402	1.22190327047234	1.05397795337867	1.41658333327072	0.00788556059920376
ADGRL2	0.185426329579853	1.20373151710192	1.04292434370135	1.38933334332007	0.0112636005732267
ADIPOR2	0.184956221515632	1.20316576620102	1.0351744004337	1.39841930050782	0.015930281642323
ADIRF	-0.238886102	0.787504572255167	0.621771602040123	0.997413598961335	0.0475414227117458
ADK	0.226851153272634	1.25464310452094	1.08129471391273	1.45578194313543	0.00278787415292606
ADSL	0.204097168966928	1.22641731737035	1.04677702828297	1.43688617127284	0.0115468767901267
AEBP1	0.210924710118625	1.23481938215668	1.06003260610677	1.43842641987204	0.00675615476008223
AGAP2-AS1	0.161240707502935	1.17496775833286	1.00552384840451	1.37296518159395	0.0424284551328291
AGAP6	-0.322048872	0.724662774087448	0.615155639407804	0.853663857578632	0.000116787206348516
AGAP9	-0.212811869	0.808308190913307	0.693689958445413	0.941864767599856	0.00637922937907871
AGBL2	-0.215488909	0.806147210931066	0.682515438283285	0.952173810641628	0.0111830668674481
AGBL3	-0.175508825	0.83902999278551	0.718596082347385	0.979648158523272	0.0264150149031935
AGER	-0.291173395	0.747386073770275	0.640519098146892	0.872083197646739	0.000216877774009072
AGFG1	0.186747082137038	1.20532239893066	1.03044632543221	1.40987652583903	0.0195452477710994
AGL	0.164807226378937	1.17916578474194	1.01022583561673	1.37635754193243	0.0367151630677041
AGO1	0.173559025235858	1.18953089703926	1.02608861946613	1.37900735683749	0.0213647445885552
AGPAT5	0.176623312617365	1.19318155202838	1.03329057387983	1.37781399742686	0.016124462477714
AGPS	0.183266579598907	1.20113456337564	1.0200573165582	1.41435605226922	0.0279379278152423

AGTR1	0.163084613861775	1.17713628752185	1.0113155229546	1.37014592177177	0.0352728068277056
AHCY	0.251715484331275	1.28623003262241	1.10034764425859	1.50351364448513	0.00157384321601004
AHNAK2	0.283312749518131	1.32752027822737	1.1432682269106	1.54146686457568	0.00020223199486644
AK2	0.1625846380794	1.1765478949888	1.00412792985213	1.37857429123242	0.0443344504488925
AKAP12	0.200506836387253	1.22202196642688	1.03985319173459	1.43610434463231	0.0149145679758264
AKAP13	0.157298098719164	1.17034444008458	1.00415996513156	1.36403178377804	0.0441048924417966
AKAP7	-0.231038071	0.793709247893193	0.678961115216586	0.927850441	0.00373303075875087
AKNA	-0.169020116	0.844491915266045	0.724593625036154	0.984229739689097	0.0305081771140893
AKR1B1	0.249832573764693	1.28381045514179	1.10109706024276	1.49684287084374	0.00142541423531324
AKR7A2	0.245957742021963	1.27884553097352	1.09607725936619	1.49208997642798	0.00177293797095375
ALDH1A1	0.15437757836106	1.16693141165676	1.01844150943549	1.33707130639836	0.0262082766480518
ALDH1A2	-0.160426118	0.851780752533968	0.727847792913422	0.996816171528373	0.045531181277717
ALDH1A3	0.149662108729029	1.16144173539548	1.00280988674993	1.34516713740265	0.0457803846711234
ALDH1L1-AS2	-0.181295496	0.834188822660032	0.704728763200931	0.987430949590071	0.0351207341384449
ALDH3A1	0.153357100081014	1.16574119089736	1.01557539555354	1.33811091732299	0.0292851205848916
ALG1	0.284631300262792	1.32927183558364	1.10968523486554	1.59231064572108	0.00200333988282594
ALG3	0.215116763521838	1.24000667606004	1.05205950410852	1.46153002816736	0.0103140481505038
ALG9	0.199995799026228	1.221397627	1.04516895099232	1.42734068214015	0.0118806843198841
ALKBH5	0.205861088066819	1.22858252736713	1.05664904278273	1.42849230485903	0.00744319776571022
ALKBH7	-0.156872108	0.854813375362207	0.730904455929366	0.99972835131921	0.0496038566079963
ALOX5	-0.19210238	0.825222377959553	0.711283308612863	0.957413121943325	0.011275412120834
ALPK1	-0.212181395	0.808817968476582	0.701849605961843	0.932089297441526	0.00337170728582431
ALYREF	0.224427126747682	1.25160549946124	1.04896832603619	1.4933876337345	0.0127569603632393
AMIGO2	-0.220919592	0.8017811472721	0.694068283574914	0.926210033413203	0.00268762996145558
AMOTL1	0.231031750409976	1.25989924113163	1.07048918710308	1.48282310267858	0.00544541513390057
AMOTL2	0.172777474520273	1.18860158151638	1.02113258463791	1.38353602738492	0.0257568495143315

AMPH	0.168835364628499	1.18392520691179	1.03547593678454	1.35365665754991	0.0135127270653055
AMT	-0.17694252	0.837827941228414	0.71919198163252	0.976033767102974	0.0231253235159513
AMY2B	-0.274592175	0.759881959073872	0.642555741831213	0.898631128998025	0.0013316744143676
ANAPC4	-0.264567264	0.767538019753057	0.656304978095938	0.897623256607887	0.000926155926430726
ANGEL1	0.151191390798812	1.16321926624312	1.00190695711921	1.35050370869737	0.0471470984327794
ANGPTL1	0.149410399364472	1.16114942642429	1.00144312808209	1.34632507096793	0.0478086161086244
ANKRD22	-0.16670315	0.846450843041954	0.727753066762089	0.984508430688154	0.0305800004995454
ANKRD35	0.178001136784004	1.19482667949389	1.03666600246152	1.37711740390885	0.0140094186242622
ANKS3	-0.166965162	0.846229091794581	0.723000586032791	0.990460712803632	0.037586694916857
ANLN	0.273599556196896	1.31468823805814	1.10185275216731	1.56863533706177	0.00239433287161685
ANO9	-0.247499451	0.78075064919349	0.670553737756261	0.909057010487695	0.00143164535442063
ANXA1	0.329410874875965	1.39014891552573	1.19020316132028	1.62368414917805	3.21492449251823e-05
ANXA2	0.223737784797179	1.25074301259333	1.06964746378277	1.4624987545137	0.00505248672110245
ANXA5	0.305209867134665	1.35690974388625	1.14143697681924	1.61305800534358	0.000541445921251023
ANXA6	0.226889977513624	1.25469181603278	1.0712202990429	1.46958711912588	0.00490963675957356
AP2B1	0.176438120281903	1.19296060440967	1.0131432731133	1.40469274330791	0.034293156468848
AP2M1	0.173358960376016	1.18929293751153	1.01611695191293	1.39198316547326	0.0308430723085761
AP3B1	0.167332402266672	1.1821471483988	1.00531828246685	1.39007904744187	0.0429572110228859
AP4E1	0.190435252327826	1.20977604091907	1.03673253299436	1.41170265483488	0.0156058933023556
APBA1	0.153952966697616	1.16643602415003	1.02352876269977	1.32929630120616	0.0209590832038299
APEX1	0.173326614434658	1.18925446933406	1.0162702626612	1.39168314256042	0.0306795290144266
APOBEC3F	-0.166436398	0.846676665421971	0.72905493467899	0.983274842088148	0.0291845923432403
APOBEC3H	-0.283486393	0.753153370275904	0.641984095977559	0.883573288983441	0.000503192293479122
APOL1	-0.263524545	0.768338763403364	0.662938840044843	0.890496105656259	0.000464305250521954
APOL2	-0.272186132	0.761712469052047	0.657902446414366	0.881902611354533	0.000271415147957843
APOL6	-0.204210478	0.815290752274374	0.702195803639413	0.946600659387373	0.00735720306233347

ARF4	0.214821120029503	1.23964013034191	1.04432145223061	1.47148911809844	0.0140604492016084
ARF6	0.192661141090468	1.2124718670053	1.03316082045228	1.42290338462097	0.0183000198034509
ARFIP2	0.167737954638464	1.18262666820726	1.01829510662435	1.37347791151761	0.0279864957537868
ARGLU1	-0.178222077	0.836756578135177	0.719868501143875	0.97262426393144	0.0202580172464051
ARHGAP19	0.145672012522128	1.15681670442801	1.00092089007696	1.33699366344607	0.0485608505557424
ARHGAP21	0.162722035191065	1.17670956037723	1.0065917095319	1.37557797900709	0.0411089668794084
ARHGAP24	0.177153932768768	1.19381484620864	1.02519468263371	1.39016901976789	0.0225934652422051
ARHGAP29	0.220377200631419	1.2465468401742	1.07772384161069	1.44181557905034	0.00299674690221493
ARHGAP5	0.155205689991749	1.16789816136406	1.00005283346323	1.36391405501447	0.0499220385785602
ARHGAP6	-0.205478659	0.814257471512848	0.685353566348418	0.967406113383286	0.019448817050403
ARHGDIA	0.217110536341066	1.24248143389909	1.05179419357893	1.46773971848144	0.0106495020182243
ARHGDIB	-0.174405407	0.839956304299162	0.726163581587415	0.971580799452382	0.0188678242651643
ARHGEF17	0.201105160048876	1.22275334986461	1.04782314438796	1.42688750731731	0.0106798854303523
ARHGEF25	0.21501245980717	1.23987734550246	1.06439483305901	1.44429095683612	0.00575422213845766
ARHGEF26	0.21573698822056	1.24077599737849	1.06977919736368	1.43910545228822	0.00435108540596049
ARHGEF4	0.179462015772792	1.19657345228638	1.03578071381322	1.38232736680858	0.0147915256737381
ARL10	0.157189352053257	1.17021717594866	1.01667556892098	1.34694712919937	0.0284937590416627
ARL14	-0.178650447	0.83639821343913	0.712668981759184	0.981608557899263	0.0287258985619636
ARL2	0.157703716629671	1.17081924903994	1.00698973360685	1.36130257158872	0.0403126082939667
ARL4A	0.174523971941682	1.19067928493728	1.02927387465227	1.37739545760619	0.0188652653469249
ARL6IP4	-0.23914489	0.78730080187973	0.669363612270863	0.926017699912916	0.0038737960181056
ARPIN	0.152047860548185	1.16421595511256	1.00567614805762	1.34774876858369	0.0417758457980659
ARSK	0.203275797566178	1.22541038684906	1.05222893831665	1.42709496148229	0.00892642481517615
ASAP1	0.241338914979619	1.27295238484026	1.08678706053308	1.49100760665632	0.00277496261024011
ASAP2	0.156734689309678	1.16968524273113	1.00685342783195	1.35885078129897	0.0404361992330097
ASB15	-0.219745524	0.802723045548124	0.665906231573697	0.967650184518123	0.0211728780877503

ASCC3	0.164883586852094	1.17925582983709	1.01496671862008	1.37013784461371	0.0312369901278327
ASPN	0.15807433442548	1.17125325590968	1.01789647701624	1.34771484178851	0.0272652109839691
ATAD3A	0.234557266319016	1.26434887496866	1.0774106506584	1.48372217840768	0.00406164615768586
ATAT1	-0.208989089	0.811404088511645	0.698117548626985	0.943074122901321	0.00645230968518936
ATF7	0.160237343309346	1.17378942900112	1.00286916076573	1.3738398562209	0.0459724414969165
ATF7IP2	-0.184558953	0.831470920728279	0.715232136657199	0.966600711271009	0.0163016488441242
ATIC	0.214715978131065	1.23950979907698	1.04944977258618	1.4639905426076	0.0114616159713531
ATOH8	-0.207170791	0.812880805626843	0.691166150260527	0.956029463982683	0.0123038191393103
ATP11A	0.19091178533855	1.21035267652019	1.04576225173287	1.40084765837751	0.0104680543366433
ATP1A1	0.240171490842501	1.27146717660344	1.08570789726144	1.48900895467158	0.00287840959982235
ATP1A4	-0.23643489	0.789437280643563	0.67519843598465	0.92300453741583	0.00303101353180721
ATP2B4	0.226561694379181	1.2542799894722	1.06618803910146	1.47555424962021	0.00627406085049831
ATP6V0A1	0.292652015217535	1.33997641788883	1.15664986571574	1.55235984	9.67263737685169e-05
ATP6V0B	0.226668672181006	1.25441417676575	1.05719640003645	1.48842251715637	0.00939613998095915
ATP6V0D1	0.244775842629549	1.27733495706651	1.08298298951216	1.50656530005061	0.00365391582913637
ATP6V1D	0.172699480918055	1.18850888181247	1.02251960365766	1.3814437953994	0.0244413767292544
ATPAF2	0.183659270316877	1.20160633039291	1.04083741317635	1.38720779534054	0.0122058592925788
ATRN	0.182922175222148	1.20072095860267	1.0295990757552	1.40028371662069	0.0197110987180593
ATXN7L2	-0.155526842	0.855964101230616	0.735616323117183	0.996000930880408	0.0442413264213156
AURKC	-0.164944749	0.847940552633762	0.722314834270079	0.995415221573602	0.0437860742487198
AVEN	0.187830559801598	1.20662904656243	1.0332164191551	1.40914684379363	0.0176562872840355
AZI2	0.208972582551677	1.23241120849642	1.05542690510021	1.43907397043606	0.00824231635508167
B3GALNT2	0.171633661189657	1.18724282041532	1.01346532773269	1.39081769850097	0.0335354233669789
B3GALT6	0.175249797992693	1.19154382469547	1.01474406394746	1.39914756499963	0.0324703299159569
B3GAT3	0.213182354080135	1.23761031395808	1.05016972946436	1.45850641685955	0.0109539128962212
B3GNT3	-0.201800013	0.817258352527572	0.703333751544647	0.949636233593551	0.00842286029845602

B3GNT9	0.191245614870256	1.21075679543705	1.03541109461945	1.41579709287912	0.0165787535803439
B4GAT1	0.231433693270429	1.26040575042357	1.08266164144175	1.46733069214983	0.00284505063249982
B9D1	0.192687280000095	1.21250356011207	1.05247203227885	1.396868362	0.00762776951198372
BACE1	0.242520453287726	1.27445731573902	1.09090810213474	1.48888934499827	0.00223864998524885
BAG2	0.224373022593931	1.25153778423672	1.06049381025937	1.47699761207382	0.00793180802362848
BAG3	0.165847809283217	1.18039344312824	1.00687869929767	1.38380986860884	0.0409071326415392
BAIAP2	0.337630947716075	1.40162313572389	1.2037071239792	1.63208090694196	1.37917499368144e-05
BCAT1	0.169749008615762	1.185007387	1.02937674777303	1.36416769769175	0.0181269041393979
BCL2L14	-0.396255067	0.67283505590314	0.566456158121683	0.799191616087847	6.39941620898836e-06
BCORL1	0.181400428851454	1.19889515539874	1.03138166097737	1.39361562069707	0.0181588320793417
BDKRB2	0.221166631506303	1.24753129126374	1.06578615330664	1.46026885210845	0.00590427825830266
BDNF-AS	-0.194943032	0.822881534900194	0.704446327833406	0.961228689433717	0.0139443367799272
BEND5	-0.153535187	0.85767058461531	0.744380745023628	0.988202390553532	0.033657273450534
BFAR	0.208733826313689	1.23211699775634	1.05333088494453	1.44124920085302	0.00906700222902157
BGN	0.226730855130106	1.25449218236394	1.07932653842533	1.45808574104761	0.0031286271205276
BHMT	-0.173328606	0.840861257201874	0.717184460078525	0.985865831205685	0.0327338119274072
BICD2	0.186374552255884	1.20487346394667	1.04026245989746	1.39553249308444	0.0128965071485053
BLNK	-0.230330727	0.79427087253117	0.681038608537113	0.926329595772172	0.00333403548226608
BLOC1S3	-0.18313699	0.832654082786731	0.703014691284254	0.986199620259545	0.0339303535865257
BMP1	0.161036994154193	1.17472842609456	1.0120325423305	1.36357954646081	0.0342414914862874
BNIP1	0.170719021016127	1.18615741688853	1.02274897134147	1.37567424369467	0.0239830357628823
BNIP3L	0.164664291681614	1.1789972530822	1.0191098483538	1.36396927673768	0.0267913415570173
BRICD5	-0.161441333	0.850916450611711	0.72872557502219	0.993595985566424	0.0412315545802316
BTBD16	-0.179045883	0.836067537218102	0.715944472825464	0.976345168266073	0.0236687635725991
BTN3A1	-0.148543876	0.861962186911018	0.748076665378833	0.993185386003416	0.0399233657798352
C10orf95	-0.168678662	0.84478031991204	0.717749609535361	0.994293524412611	0.0424791094666994

C11orf96	0.163306921496983	1.17739800299584	1.00264096535769	1.38261462014377	0.0463559234105503
C14orf132	0.183035401681008	1.20085691968194	1.04401081321363	1.38126667204635	0.0103748394047806
C16orf74	-0.188636368	0.828087570934612	0.716350881325819	0.957252992928886	0.0107507740467148
C17orf67	-0.167515989	0.845763094177053	0.717496389389902	0.996960015478524	0.0459071644883748
C18orf54	0.198941291946396	1.22011033349433	1.04766492526457	1.4209402166668	0.0105005882355706
C1QTNF7	0.195431978479343	1.21583608808973	1.02289055221506	1.44517650485694	0.0266455686098673
C1RL-AS1	-0.192524654	0.824873981123058	0.707851323327101	0.961242936632018	0.0136501989993442
C1orf159	-0.223145038	0.799998810363163	0.67738383444878	0.944808635864885	0.00856911427005551
C1orf226	-0.14441471	0.865528730321549	0.75189867638372	0.996331030418945	0.0443093119709885
C3orf62	-0.240828498	0.785976411184439	0.675777581293464	0.914145328343028	0.00178029289862893
C6orf120	0.211074512797132	1.23500437526348	1.06098094945316	1.43757134160238	0.00645278024061898
C6orf136	-0.244402896	0.783172034156426	0.668045376221467	0.918138882352471	0.00258866241854395
C7	0.166211642773129	1.18082298793078	1.0195341085377	1.36762754394323	0.0265454000171685
C7orf50	0.157778880093094	1.17090725517712	1.00046970217804	1.37038012969477	0.0493200108061201
C9orf64	0.161458511094419	1.17522369840183	1.00230853283317	1.37796965309798	0.0467718019819059
CAD	0.243879451952734	1.27619047894621	1.08621579518984	1.49939095506185	0.0030208770820443
CALD1	0.247948041832675	1.28139335161416	1.09370939901817	1.50128445731103	0.00215128749688113
CALM1	0.299090676432759	1.34863190705914	1.14409003960397	1.58974202884204	0.000365276573745247
CALU	0.369971726139064	1.44769368219482	1.21015578542651	1.7318571895503	5.21045624987517e-05
CAP2	0.205408144548234	1.22802617488195	1.05538209090515	1.42891214394382	0.00787730038962085
CAPG	0.175888042140889	1.19230456331157	1.01131623065995	1.4056831370796	0.0362665917463808
CAPN10	-0.166918482	0.846268594554306	0.720504284135124	0.993985115561935	0.0420059981604941
CAPN2	0.232912429963212	1.26227093737483	1.07620301020095	1.48050869978854	0.00420249967506391
CAPN8	-0.184551614	0.831477023278529	0.710931496974745	0.972462245915493	0.0209218321886472
CAPS	-0.162847591	0.849720683443423	0.731962830991086	0.986423366462377	0.0323902153332367
CARD11	-0.282556903	0.753853744124381	0.64982574268738	0.874535171814123	0.000191898722481968

CARD8	-0.188719349	0.828018858024558	0.709779724784514	0.965954936867832	0.0163694749923076
CARM1	0.278832821034086	1.32158638395509	1.10676616646766	1.57810260484371	0.00206453565384255
CASP9	-0.149811571	0.860870174027125	0.74223884583398	0.998462234480325	0.047669063499773
CAST	0.248499224818892	1.28209982850936	1.10244466558741	1.49103172392592	0.00125465815106079
CATSPER2	-0.227656983	0.79639739094728	0.675606865673288	0.938783834997834	0.00667427095328543
CAV1	0.192872283818904	1.21272789865212	1.04025920314225	1.41379086262991	0.0137297948700182
CAV2	0.156194081318843	1.1690530724357	1.00401462136378	1.36122030206587	0.0442659981358796
CCDC125	-0.178114704	0.836846427944811	0.721300829629457	0.970901342680766	0.0187987702728183
CCDC14	-0.212111577	0.808874440990978	0.689493643504312	0.948925153193781	0.00922975553195672
CCDC146	-0.21297646	0.808175161258951	0.688208149359642	0.949054572927778	0.00938363083524323
CCDC159	-0.214419955	0.807009406322106	0.691393951284811	0.941958171144135	0.00656994630050403
CCDC191	-0.164476172	0.848337970852206	0.728745410837679	0.987556562397262	0.0338822107414001
CCDC24	-0.218247356	0.803926561142001	0.686432117802292	0.941532161663436	0.00678303745114996
CCDC80	0.290738183851635	1.33741438142558	1.14591889450526	1.5609108430106	0.000226456289574516
CCDC85C	0.174386718733535	1.19051587160031	1.02235703560128	1.38633372801965	0.0247967270378186
CCDC86	0.172587616959537	1.18837593794017	1.02242199113794	1.38126662191937	0.0245184207895049
CCDC88A	0.159654681105963	1.17310570547538	1.00528812214589	1.36893788547038	0.0426702515159476
CCL15	-0.199337769	0.819273121375202	0.691282670889787	0.970960904522483	0.0214492703653859
CCL17	-0.222715526	0.800342493498029	0.686243511935727	0.933412259289435	0.00453883662252038
CCNL1	-0.202692581	0.816529219313132	0.695961802466064	0.957983561209343	0.0128990175404688
CCNL2	-0.254773452	0.775092073286363	0.656358866612235	0.915303734940288	0.00267197401015525
CCNT2	-0.196830232	0.821330057238753	0.706998739550211	0.95415030492555	0.0100632204385381
CCNYL1	0.224206751743746	1.25132970728434	1.08027653837234	1.44946778043662	0.00279363004499675
CCRL2	-0.264699926	0.767436203196606	0.654929349876817	0.899270014525379	0.00106554527545209
CCT6A	0.216659599305299	1.24192127931101	1.06683202819805	1.44574630610844	0.00520055983119701
CD109	0.305077488992883	1.35673013058449	1.16537378127012	1.579507517	8.39270762702061e-05

CD248	0.19473693334076	1.21499132073712	1.04700656758597	1.40992803213274	0.0103173923232112
CD276	0.225294278352883	1.2526913018888	1.05489639043418	1.4875731039159	0.0101852805564143
CD2AP	-0.176128527	0.838510205388747	0.718699914601512	0.978293374267225	0.0251600623505018
CD3D	-0.182759127	0.832968771667846	0.716936774437288	0.967779864714599	0.0169461759626089
CD93	0.187089086348259	1.20573469476641	1.03059556141244	1.41063692547929	0.0194754915347131
CD96	-0.296675334	0.743285292793694	0.637659246404965	0.866407928055893	0.000148457284792825
CD99	0.195082383524639	1.21541111221663	1.0428464458042	1.41653085901874	0.0125265735242533
CD99L2	0.184379724857623	1.20247234505447	1.037353124	1.39387418533824	0.0144219266381284
CDC42BPB	0.184750220260289	1.20291793807013	1.02896562732988	1.40627784572923	0.0204350385775994
CDC42EP1	0.181402683336003	1.19889785829239	1.02791170030311	1.39832640701942	0.0208539481487951
CDC42EP5	-0.198333104	0.820096630351513	0.707645296190244	0.950417513879856	0.00839390857035779
CDC73	0.248476489354557	1.28207067970579	1.07976850951493	1.52227557414105	0.00456997595205838
CDCA4	0.182880926530083	1.20067143145506	1.01529475767465	1.41989493732254	0.0325684411633889
CDCA7L	0.174819683979876	1.19103143520044	1.02951909047816	1.37788205459771	0.0187112947329151
CDH11	0.213821189209471	1.23840119549763	1.0707865044668	1.43225331530829	0.00395513216040131
CDK5RAP2	0.188855985335073	1.20786698939683	1.03999099879568	1.40284162628719	0.0133780101199228
CDK6	0.234111465043174	1.263785352	1.09251151029908	1.46190992176762	0.00162855151823945
CDKL1	0.221987060783311	1.24855522243319	1.04713803667851	1.48871503933708	0.0133939587561332
CEACAM1	-0.149492664	0.861144755350168	0.742022582465294	0.9993904595239	0.0490691072717101
CEBPZ	0.17201992840833	1.18770150197857	1.01209181575839	1.39378150859279	0.0351005030739523
CERCAM	0.395302905932958	1.48483388780362	1.25871036868767	1.75157981471835	2.73851918988873e-06
CERK	0.226790771391721	1.25456734909757	1.08255421318598	1.45391262095739	0.00257628091290133
CES1	0.27903863913232	1.32185841834513	1.15589600308615	1.51164955453148	4.57320115968913e-05
CFAP44	-0.239052205	0.787373776429752	0.67233034063606	0.922102464129071	0.00301425499390347
CFAP69	-0.169890404	0.843757283701579	0.720610447791734	0.98794897573456	0.0348079187428969
CHD8	0.161917291186755	1.17576299133767	1.01216504346059	1.36580355222782	0.0341630077470751

CHKB	-0.19471028	0.823073084187859	0.699528982123717	0.968436361075175	0.0189520414118508
CHMP4C	-0.315244053	0.729610788483606	0.617861385999316	0.861571729087241	0.000201955068279782
CHMP6	0.188408276944628	1.20732633824719	1.02905152638696	1.41648581207902	0.020817910575753
CHPF	0.229824712742498	1.25837941271995	1.07827752114754	1.46856325509983	0.00354232156217164
CHPF2	0.248276677807278	1.28181453277097	1.08745610931753	1.51091017131165	0.0030834356767395
CHRDL1	0.168811880692027	1.1838974040139	1.02238713265494	1.37092204945023	0.0240817615805407
CHRDL2	0.161385969025727	1.17513844833572	1.02159631721622	1.35175739133425	0.0238804851665554
CHST11	0.166397206148186	1.18104212576111	1.01528106950063	1.37386635555846	0.0310439551555568
CHSY1	0.221719197993807	1.24822082573668	1.06264675385419	1.46620240842193	0.00693664287458492
CIB2	0.163176752673998	1.17724475245805	1.01411292256442	1.36661822993581	0.0320241967563173
CILP	0.172194454471288	1.18790880493504	1.0289385312056	1.37143987327288	0.0188162486917746
CLASRP	-0.289160723	0.748891831772497	0.641663437868297	0.874039165389814	0.000244911760022087
CLCN5	0.148628928187845	1.1602423760788	1.00783762425672	1.33569370586038	0.0385814707117119
CLDN10	0.223496161385697	1.25044084030716	1.0963946740059	1.42613087438224	0.000862479672939732
CLEC11A	0.170351211514143	1.18572121714387	1.0226518662514	1.37479317369136	0.0240273510471197
CLEC2D	-0.375144322	0.687190095139358	0.580748809526334	0.813140240860411	1.2482072131041e-05
CLIC3	0.248900403573493	1.28261428290902	1.09782899966024	1.49850240723406	0.00171338873318857
CLIC4	0.264999214731688	1.30342995223573	1.09505771000142	1.55145215167065	0.00286651753924469
CLIP1	0.183434834721126	1.20133667742128	1.02130732929766	1.41310041661024	0.0267949769342759
CLK1	-0.176482668	0.838213307257803	0.720986163762566	0.974500737708253	0.0216754432067446
CLMP	0.161816537365061	1.17564453469047	1.01227968699125	1.3653737101607	0.0340184926239385
CLSPN	0.156485835825433	1.16939419869817	1.00215750437384	1.36453879353361	0.0468853651712142
CLSTN1	0.164548104837503	1.17886027706971	1.01632053233319	1.36739493953004	0.0297176935904435
CLTC	0.279264216898723	1.32215663384877	1.11662721113837	1.5655163576465	0.0011966631478435
CLUAP1	0.189557288282047	1.20871436717558	1.02975049989109	1.41878097808274	0.020419695950734
CMSS1	0.156823761554677	1.16978943386184	1.00574650863037	1.36058868495433	0.04192201850505

CMTM3	0.168100404857529	1.18305538919254	1.01561763549865	1.37809743054562	0.0308502439286639
CMTM8	0.18079899385354	1.1981743146846	1.02780379038582	1.39678575015859	0.0208650867226954
CNN1	0.194403281896198	1.21458600474872	1.04134255142551	1.41665118832608	0.0132898320039861
CNN3	0.309539797372402	1.36279780666746	1.15223717401973	1.61183643761337	0.00030058543536408
CNP	0.217334080791028	1.24275921477485	1.06196357834196	1.45433468473482	0.00673919305960845
CNRIP1	0.165344967527359	1.17980004122283	1.01161904910019	1.37594101110242	0.0351014977285784
CNTN1	0.299020408531051	1.34853714485427	1.16726559207302	1.55795942534553	4.90991352220782e-05
CNTNAP3B	0.259032612832918	1.29567605980587	1.15667721052706	1.45137851483138	7.68339025153433e-06
COA7	0.165151977793323	1.17957237389609	1.00796348599626	1.38039820349605	0.0395089371488315
COG8	0.164227336662959	1.17848219685188	1.01765694022118	1.3647234479578	0.0282519846993835
COL12A1	0.169153941321557	1.18430243797436	1.01497046081798	1.38188481215665	0.0316565503160604
COL14A1	0.272927628497859	1.31380515933114	1.13331174632194	1.52304430117041	0.000294973557677937
COL15A1	0.198533016428704	1.21961229399187	1.04288211445727	1.42629174192924	0.012931089879063
COL16A1	0.192491053628474	1.21226565827996	1.03968981858968	1.41348698426076	0.014021092501007
COL18A1	0.263020678357427	1.30085361818968	1.11558651771903	1.51688829963378	0.000792762229538489
COL1A1	0.236293172724987	1.26654557235902	1.08724326162188	1.47541745576724	0.00241372743377691
COL1A2	0.25275739774612	1.28757087134561	1.10446467420588	1.50103374734886	0.00124004183375362
COL3A1	0.223904486861115	1.25095153141476	1.07309337185307	1.45828850964443	0.00421572728013429
COL4A1	0.239337715635036	1.27040750062166	1.08555710946339	1.48673451038753	0.00285240726718264
COL4A2	0.275601049697149	1.31732221308411	1.12895413043042	1.53711985838008	0.000464294808287369
COL5A1	0.261081014444702	1.29833284488757	1.11586145833265	1.51064288808095	0.000728456267532138
COL5A2	0.26033960421955	1.29737060439227	1.1128241565296	1.5125215203723	0.000882675286652493
COL6A1	0.258194942358698	1.29459116468216	1.1133932833066	1.50527788230927	0.000790284147394142
COL6A2	0.228301825594167	1.25646450135164	1.07949325304929	1.4624482725551	0.00320333784956663
COL6A3	0.253442549747681	1.28845335538859	1.10422826661282	1.503413831	0.00128464447834501
COL8A1	0.228854435109543	1.25715902747401	1.08321428258107	1.45903617204302	0.00259564108623287

COLGALT1	0.243324431787445	1.27548236402286	1.08724866367075	1.49630467738702	0.00281957078981452
COPB1	0.171197553988254	1.18672516815566	1.00927632192286	1.39537269838153	0.0382932913980195
COPB2	0.184703941659479	1.2028622699992	1.02135630019441	1.41662379750554	0.0268862046590085
COPS3	0.240548127386267	1.27194614779972	1.09285931085473	1.480379942	0.00189095795243765
COPS8	0.184251045423234	1.20231762154832	1.02174109488783	1.41480818410687	0.0264871931117958
COPZ1	0.174541309362341	1.19069992842387	1.01570874063361	1.39583943982227	0.0313853546054944
COPZ2	0.22006784995088	1.24616127970064	1.06398550230344	1.45952922447082	0.00635056267150037
CORO1C	0.227678506194131	1.25568156668702	1.06443162043205	1.48129402269878	0.00692130480182415
COX7A1	0.165193479842564	1.17962132958271	1.00882666776766	1.3793315796118	0.0384440800230465
COX7B	-0.16256612	0.84995988890969	0.737282428034155	0.979857630245739	0.0250657649685856
CPA4	0.152567518639818	1.16482110657635	1.0164042934533	1.33490995567905	0.0282392542850617
CPED1	0.221026852560564	1.24735692484173	1.06153990259491	1.46570024748693	0.00723989297878163
CPM	0.167510358402102	1.18235753745629	1.02358069309072	1.36576369192596	0.0228014945532497
CPNE7	-0.192482072	0.824909107053555	0.700004063296506	0.972101549947231	0.0215770296334445
CPSF2	0.191784915374559	1.21140993329038	1.03487210825211	1.41806317396381	0.017009336994355
CPVL	0.18542602501334	1.20373115048567	1.03799041266616	1.3959364797289	0.0141558200423566
CPXM1	0.166732387018096	1.18143805483844	1.01648069503698	1.3731651611639	0.0297799960295784
CRB3	-0.232036718	0.792917008550362	0.686353464649571	0.916025655628411	0.00162662402260022
CREB3L1	0.163371252365968	1.17747374846888	1.01366090746313	1.36775959112716	0.0325582916149114
CREB3L2	0.174379757875373	1.19050758461703	1.02348284349937	1.38478951360316	0.0237648675115316
CREB5	0.181549843321666	1.19907430106636	1.03243640401771	1.39260798426198	0.0174021463629939
CRIP2	0.196708401359665	1.21738899996658	1.05095307401868	1.41018282726225	0.00872783099197594
CRISPLD2	0.214431986049059	1.23915783808791	1.05941319267512	1.44939874103076	0.00732330988838549
CRK	0.158070865616766	1.17124919306322	1.00952479871522	1.35888159854727	0.037068889437997
CROT	-0.159673702	0.852421887570807	0.734761506204215	0.988923709631332	0.0351233169828025
CRTAC1	-0.270314181	0.76313969275827	0.636449837316862	0.915048062732464	0.00351752678378177

CRYAB	0.256979580565447	1.29301872377858	1.11274060279811	1.50250419175665	0.000795459157427186
CRYBG3	0.179809269722121	1.19698903929629	1.02356952256515	1.39979036949516	0.0243416907526591
CSAD	-0.229415119	0.794998446082397	0.680631636034924	0.928582357639626	0.00379181028234628
CSDE1	0.175925479093773	1.19234920039686	1.01164485855822	1.40533172650451	0.0359020771116604
CSE1L	0.219391082331464	1.24531820341351	1.0642602792099	1.45717871656769	0.00620196362660327
CSGALNACT2	0.204236172824232	1.22658780595714	1.04089555887395	1.44540692185329	0.0147472977052629
CSRNP3	0.159890354834836	1.17338220825229	1.01269908692589	1.35956062804644	0.0333448704646945
CSRP1	0.190229755316878	1.20952746110083	1.03353977595507	1.41548173877016	0.0177317825634552
CTDSPL	0.162037492818529	1.17590432846211	1.0185885081473	1.35751677800784	0.027014326638633
CTHRC1	0.250632695804998	1.28483807123684	1.09869487237818	1.50251804281779	0.0016972105871699
CTNNB1	0.172350381875693	1.18809404691344	1.01064499802582	1.39669959982831	0.0367742913253142
CTNND1	0.159527367889939	1.17295636312211	1.00222337747846	1.37277443402902	0.0468520210837629
CTPS1	0.227178153867205	1.25505344064852	1.07993929301187	1.45856267021332	0.00304649066008084
CTSA	0.168283862141499	1.18327244923104	1.02114700369819	1.37113822401525	0.0252013454198245
CTSE	-0.330655989	0.718452282221251	0.607269884706762	0.849990580511287	0.000115875503104716
CTSH	-0.16079961	0.851462678709111	0.735894338415463	0.985180419780848	0.0307280440894442
CTSS	-0.183849911	0.832060677297065	0.723544558878674	0.956851879001059	0.00992071347301959
CTSV	0.2078632043349	1.23104475644381	1.06673648571474	1.42066125295453	0.00445753136546334
CUEDC1	0.188574334606384	1.20752684068292	1.03633785527353	1.40699392919969	0.0156250693985977
CUL1	0.239299599504441	1.27035907852629	1.08457575771781	1.48796631024649	0.00301314346896846
CXADR	-0.155646281	0.855861871461197	0.736282262809425	0.994862405385228	0.0426577641465849
CXCL12	0.201239957895714	1.22291818549288	1.04741678959066	1.42782596505223	0.0108935959787349
CXorf38	-0.298419405	0.741990080002606	0.635439409870472	0.866407198342479	0.00016127914894085
CYBRD1	0.172629070992259	1.18842520193628	1.01508810231427	1.39136145658421	0.0318618453275455
CYP1B1	0.194624493652248	1.21485471517156	1.05780464622163	1.39522168317773	0.00585813109439855
CYP3A5	-0.15732491	0.854426401918867	0.73294585047768	0.996041489040743	0.0443618858995166

CYP4F12	-0.233063011	0.79210366043032	0.681371998463982	0.920830633312674	0.00241740153926358
CYP4F35P	-0.430213397	0.650370293012876	0.52643883429444	0.803477043255282	6.64831053672383e-05
CYP4F8	-0.203641169	0.815755036670582	0.696280326097609	0.955730407583187	0.0117233382753274
CYP7B1	0.162405510295839	1.1763371614468	1.01943159533433	1.35739280961456	0.0261858539522115
CYTH3	0.316701007003403	1.37259211513705	1.18109690583704	1.59513508605902	3.61092700980434e-05
DAPL1	0.156883441939405	1.1698592494286	1.01645169527722	1.34641977561008	0.0287067565695105
DARS2	0.231985387493443	1.26110130084266	1.07653595820866	1.47730921467168	0.00406039318983951
DBN1	0.177570193375423	1.19431188774298	1.02129302181166	1.39664215336944	0.0261597847806912
DCBLD2	0.243625630182568	1.2758665951259	1.09710283494726	1.48375841963476	0.00156031437407826
DCLK1	0.153930669066517	1.16641001567983	1.01683327324022	1.33798958047747	0.0279228778379355
DCN	0.224419369877694	1.25159579095776	1.06885917834779	1.46557381521915	0.00532102344495894
DCTN2	0.160576780344289	1.17418792423274	1.00809324070624	1.36764857231668	0.0390603535369654
DCUN1D5	0.163788651510066	1.1779653275889	1.01021969097381	1.37356490414876	0.0366456857615548
DDB1	0.314905193965856	1.37012940826385	1.16263431308665	1.61465610833864	0.000170960076650143
DDOST	0.170951173485458	1.18643281822819	1.00778880777449	1.39674386271203	0.0400584028153513
DDR2	0.185101954490624	1.20334111990464	1.03190006874794	1.40326558230617	0.0182554347166603
DDTL	-0.248750581	0.779774439990038	0.659033902353727	0.92263565666371	0.00375461978914696
DDX10	0.280231974602171	1.3234367804523	1.11865516700298	1.56570582563562	0.00108587879830085
DDX21	0.185539435766361	1.20386767428336	1.02704558954838	1.41113246766555	0.0220662498367591
DDX39B	-0.247719022	0.780579238226418	0.669308149570328	0.910348914085817	0.00159401371871953
DDX46	0.172474217796162	1.18824118474363	1.02183714371444	1.38174377571414	0.025052318202287
DDX47	-0.213286443	0.807924679475282	0.687392521350677	0.949591779704914	0.00966950382381674
DEGS1	0.250875296926562	1.28514981220678	1.10215311600892	1.49853048167742	0.00136945188553171
DEGS2	-0.186822383	0.829591072700502	0.712641873560493	0.96573240141768	0.0159651039139497
DENND2D	-0.271665093	0.762109453808045	0.647117605182929	0.897535185152953	0.00113248327861474
DHCR24	0.206332347491787	1.22916164490908	1.03752304180224	1.45619739364167	0.0170365445143618

DHRS2	-0.170494489	0.843247736879593	0.727729436768066	0.977103178497888	0.0233228788404821
DHRS4	0.183461954168645	1.20136925745003	1.02870737270837	1.40301132376079	0.020477201430865
DHRS4L2	0.154114542458653	1.16662450716507	1.00078054260997	1.35995124082718	0.0488455664110606
DIAPH3	0.18609738875351	1.20453956327217	1.03144946523797	1.40667634080663	0.0187139290058745
DICER1-AS1	-0.200408934	0.818396015039033	0.695446683708952	0.96308179062663	0.0158278988214695
DIP2B	0.149462589446001	1.16121002848893	1.00240468537709	1.34517400999179	0.0463738951146672
DIXDC1	0.229840072963446	1.25839874185422	1.07700414063051	1.47034475890985	0.00380310587139116
DKK3	0.224364459034452	1.25152706666435	1.08401052884633	1.4449306135988	0.00221162609400919
DLAT	0.198849695444721	1.21999858077428	1.04075698397432	1.43010958370661	0.0141786470615099
DLEU1	0.216219589101459	1.24137494148167	1.07859259412836	1.42872457471671	0.00257060551616313
DLGAP5	0.184787031159959	1.20296221937667	1.01200358991026	1.42995352553638	0.0361479165249579
DLX2	0.132458019450367	1.1416310887826	1.01194710543239	1.28793445416107	0.0313183459964058
DNAAF5	0.213867077768166	1.23845802524748	1.05897280066139	1.44836418776947	0.00742279413788082
DNAJB4	0.272478555172776	1.31321529693537	1.11172317573758	1.55122646873032	0.00134500549845416
DNASE1	-0.201901887	0.817175099168218	0.698773198756091	0.955639317434206	0.0114673134422365
DNM1	0.186125011386443	1.20457283628592	1.04262451098175	1.39167620042965	0.0115177284677358
DNTTIP2	0.230004456833728	1.25860561931295	1.08609349450995	1.45851909892977	0.00222825567729503
DOCK8	-0.253833286	0.775821131451499	0.663107484748064	0.907693612047471	0.00152900844318588
DOLK	0.223897848186476	1.25094322678212	1.06829667100319	1.46481684264968	0.00542976857094545
DPAGT1	0.17443822928048	1.19057719730345	1.01592969888616	1.39524817937011	0.0311439754281966
DPH5	0.18381145309699	1.20178920809994	1.03176651940567	1.39982958696647	0.0181873852209492
DPT	0.163515260771051	1.17764332679548	1.00811483680842	1.37568038333462	0.0392194850201506
DPYSL2	0.274679492296359	1.31610878425745	1.1258819217734	1.53847601467061	0.000563618467917355
DSC2	0.224935971870361	1.2522425348776	1.08084408194156	1.45082106878896	0.0027431444793445
DSC3	0.181152570490928	1.19859803603436	1.03455733054256	1.38864924115127	0.0158484599857156
DSEL	0.228295118857817	1.25645607460375	1.10419594976123	1.42971169904222	0.000532533262109947

DSTN	0.247679473495098	1.28104925614058	1.08486465	1.51271146715805	0.00349571737014564
DTD2	0.269443460404531	1.30923560648191	1.11213354166397	1.54126982872527	0.00120948475422025
DTX4	-0.1919914	0.825313965767119	0.70788941577907	0.962216875838741	0.0142133864410358
DUSP1	0.179336783149105	1.19642361163618	1.01226861805625	1.41408064316879	0.0354697983550832
DUSP2	-0.22765755	0.796396939345116	0.671974896181166	0.94385681459635	0.00862391608702853
DYM	0.353353222005397	1.4238339841164	1.21274010404099	1.67167161996999	1.59028644011175e-05
DYNC1H1	0.203588402868834	1.22579351651498	1.04752008610108	1.43440661908717	0.011119115839403
DYNC2H1	0.153348405080324	1.16573105482097	1.021320407	1.33056079402288	0.0230497331760383
DYRK4	0.223096340645551	1.24994098805718	1.07554739290816	1.4526114645687	0.00361544445266589
EBPL	0.166464586478522	1.18112170745078	1.01537856749919	1.37391957292082	0.0309441007611791
ECHDC3	0.170329610353958	1.18569560446656	1.03488300762879	1.35848598932219	0.0141292193195508
ECM1	0.289071463795863	1.33518714266054	1.15829033591599	1.53910004309603	6.7089347073245e-05
ECM2	0.191870978255506	1.21151419520572	1.04383575961118	1.40612795803402	0.0115888627106058
EDIL3	0.151537547563487	1.16362199216006	1.01062604410532	1.33977958369084	0.0351240487966497
EFEMP1	0.293379841046315	1.34095204233499	1.15299302607525	1.55955182657369	0.000140339593932584
EFEMP2	0.210275704160459	1.2340182370225	1.06024724121657	1.43626972097263	0.0066186760272924
EFHD1	0.153173109340505	1.16552672504288	1.02000477480819	1.33180998789406	0.024382042290383
EFNB2	0.193865752258377	1.21393330421214	1.04426462431493	1.41116919290659	0.0116083698963452
EFTUD2	0.203483840467242	1.22566535130178	1.03958136741542	1.44505817482723	0.0154352016459404
EGFL6	-0.213533219	0.807725328095061	0.695540096582993	0.938005168719165	0.00512893482468077
EGR1	0.244436058369365	1.27690101248106	1.0831279070416	1.50534039892716	0.00360380673113786
EGR2	0.227213046871684	1.25509723399788	1.07258003901737	1.46867274187974	0.00459899464372936
EGR3	0.172219622139406	1.18793870220582	1.01124251780258	1.39550932180439	0.036079563751396
EIF1	0.215981168018956	1.24107900680427	1.06094482589866	1.45179755207874	0.00694754627429436
EIF2B3	0.220239222025664	1.24637485524458	1.0700651453285	1.45173430474557	0.00465191900567946
EIF2D	0.240007318190263	1.2712584535986	1.07827393128816	1.4987824605156	0.00427561572711359

EIF2S1	0.204793130082005	1.22727115321791	1.05192342366456	1.43184803155511	0.00922803243156357
EIF3A	0.189338190494646	1.20844956954148	1.02539814857953	1.42417885593806	0.0238686255702097
EIF3B	0.213361753360626	1.23783236027482	1.05828285363961	1.44784444619313	0.00762056076640492
EIF3C	0.2459246961766	1.27880327114012	1.09895133050194	1.48808938202181	0.00147220300943324
EIF3I	0.164432505812923	1.1787240098479	1.0093409704043	1.37653214536151	0.0377619434551311
EIF3J	0.236355072372291	1.26662397350972	1.07686916842453	1.48981541798313	0.00431295650929989
EIF4B	0.169382539302917	1.18457319806742	1.01279353411028	1.38548836887311	0.0340906684308426
EIF4E2	0.187712961783561	1.20648715772114	1.01199455724283	1.43835878496405	0.0363579620487909
EIF4G1	0.238371489818134	1.26918059292697	1.07495059311362	1.49850550135208	0.0049109901097314
EIF4H	0.193011879188261	1.21289720166774	1.02206168721765	1.43936480567846	0.02711880088808
EIF5A2	0.167286989598204	1.18209346516123	1.02099069599667	1.36861674240118	0.0252303498956569
ELAC2	0.193866524073816	1.21393424114497	1.03964916611554	1.41743617929325	0.0142185509096275
ELF4	-0.183935205	0.831989711148534	0.714229719559016	0.969165606668409	0.0181674121520791
ELMOD3	-0.23145725	0.793376611560532	0.679854941357396	0.925854045444636	0.00330571009648426
ELN	0.244537149742725	1.2770301026829	1.1021845001263	1.47961242693162	0.00113370827951255
EMC1	0.263964955090201	1.3020825641353	1.11295226007119	1.52335285586886	0.000979416737814396
EMC7	0.215847436371119	1.24091304636092	1.05899091682962	1.45408724867892	0.00761716529677471
EMILIN1	0.186623086815367	1.20517295385752	1.03513845819827	1.40313775148277	0.0161715503441204
EML1	0.196832394924396	1.21753995772705	1.03890757748535	1.42688683843283	0.0150374435966036
EMP1	0.422141321068834	1.52522405574715	1.29545789537334	1.79574220708993	4.03397778127663e-07
EMP3	0.201602024562908	1.22336104357179	1.04714467059031	1.42923159040227	0.0110701770659056
ENGASE	-0.275867407	0.758913550842272	0.645263331697097	0.892580981065867	0.000859536224106946
ENO1	0.2021575707082	1.2240408659027	1.04751825885916	1.43031019147254	0.0109520886537949
ENTPD1-AS1	0.154048138891702	1.16654704170852	1.01003254784201	1.34731499833979	0.0361023047909547
ENTPD2	0.21322053904488	1.23765757296658	1.07757483683127	1.42152193570698	0.00255123769634324
EPDR1	0.269943838868025	1.30989088371267	1.12284154941179	1.52809996043734	0.000595241015988744

EPHA3	0.242718079584088	1.27470920690754	1.0933440750437	1.48615938867177	0.00193777847007832
EPHB6	-0.213770518	0.807533678324321	0.699502238540221	0.932249542172855	0.00352979531710688
EPHX1	0.186447814157656	1.20496173850158	1.0439965156761	1.39074486308267	0.0108193019893872
EPS8L3	-0.182492808	0.8331906359845	0.707480810798023	0.981237406438213	0.0287464582870617
ERC1	0.26011262831007	1.29707616593589	1.10723326707031	1.51946895949985	0.00127480524475832
ERCC4	0.184065773283308	1.20209488622367	1.03244925323149	1.39961563337108	0.0177218654591753
ERCC5	-0.166058182	0.846996952797817	0.731377158667853	0.980894507774188	0.0265841009500244
ERI3	0.226711751638332	1.25446821741176	1.07364413328388	1.46574685196939	0.00430741885905094
ERLEC1	0.194914407333048	1.21520696923284	1.02588497815266	1.43946739597577	0.024088451624596
ESPN	0.145304963421724	1.15639217381388	1.00022372934288	1.3369437461122	0.0496477891085379
ESYT2	0.275261675856802	1.31687522423813	1.13098084510198	1.53332425011661	0.000392245416405701
ETF1	0.234544240560826	1.26433240597321	1.06956080348097	1.49457275134937	0.00599944833866262
ETV6	-0.186014236	0.830261774900908	0.720015647227997	0.957388381093507	0.0104962423508695
ETV7	-0.203723072	0.815688226925659	0.704791345185111	0.944034412582598	0.00628706475038023
EVC2	0.226503476684061	1.25420697030771	1.08520183491006	1.44953231165409	0.00216075002998998
EXOC4	0.165828605349172	1.18037077514807	1.00880551876165	1.3811137438403	0.038511695437171
EXT2	0.165667453773064	1.18018057186345	1.0120112256675	1.37629519009067	0.0346701887055516
EXTL3	0.205825488560826	1.22853879121458	1.05739985280782	1.42737636808929	0.0071628126968947
F10	0.211139455076816	1.2350845818674	1.04594441080445	1.45842733954986	0.0127873258676685
F2RL3	0.157589932806637	1.17068603632857	1.01046055362613	1.35631795891142	0.0358576589732633
FA2H	-0.177052219	0.837736037569626	0.718495301814339	0.976765842268799	0.0238185128657248
FAAH	-0.189873062	0.827064113062339	0.712523936716859	0.960016936788769	0.0125439122929669
FAAH2	-0.215134223	0.806433191157737	0.695824771087745	0.934623943876299	0.00426010461469799
FADS2	0.231469569538312	1.26045096988906	1.09177476839153	1.45518718099237	0.00158938850439244
FAF1	0.182551303188052	1.20027572734539	1.03913965527861	1.38639865617315	0.0130664165073841
FAM110B	0.262257801642582	1.29986160569467	1.13184429907073	1.49282034229122	0.000204222058044672

FAM111A	-0.162884569	0.849689263098534	0.731620045565503	0.986812551406903	0.0328529359210621
FAM114A1	0.211574403420859	1.23562189670467	1.04897598174348	1.45547800730237	0.0113339027005315
FAM117B	-0.158996866	0.852999031862647	0.737417237242542	0.986696962874624	0.0323356828198516
FAM162A	-0.175785159	0.838798172140772	0.722413462988949	0.97393308629059	0.021080859804636
FAM168B	0.194778868621812	1.21504227280797	1.03420269998184	1.42750325901902	0.0178363213435802
FAM180A	0.263839392117397	1.30191908104164	1.12207021888525	1.51059467139609	0.000504380688584046
FAM229A	-0.179726851	0.835498396118637	0.71549838572209	0.975624241572991	0.0230917183319406
FAM3C	0.150514850545691	1.16243256773292	1.00054022706476	1.35051988713162	0.0491806626551268
FAM43A	0.299356242525557	1.3489901055259	1.16040468123543	1.56822385693011	9.769044370717e-05
FAM89A	0.152115707674694	1.16429494649938	1.00077773078336	1.35452926333894	0.0488346610239731
FAM98A	0.17730136602486	1.19399086719394	1.01868844898538	1.39946044579425	0.0286320818261221
FAP	0.176785575951558	1.19337517735403	1.0307799285922	1.38161820425608	0.0179994575797399
FARSA	0.175038328179833	1.19129187578658	1.01461462551095	1.3987343545343	0.0325892029516541
FARSB	0.205215972232661	1.22779020492251	1.051845897	1.43316505864018	0.00930910718229869
FASN	0.271608039152378	1.31207261941286	1.12804482819112	1.52612246924044	0.000427345343387137
FBN1	0.267594535190939	1.30681716420696	1.11865922019745	1.52662318410471	0.00074186301788646
FBP1	-0.293891782	0.745357148332973	0.642770929892341	0.86431612373029	0.000100229680195956
FBXO17	0.146235736426885	1.15746901350156	1.0072395028194	1.33010521674951	0.0392400934829792
FBXO46	-0.192506439	0.824889007006181	0.699529738385022	0.97271329086102	0.0220843912580732
FBXO6	-0.211701311	0.809206362700188	0.694890613827548	0.942328079275187	0.00644157220494747
FBXW9	0.177045335151054	1.19368520779969	1.01354280741592	1.40584528339025	0.0339110765128313
FECH	0.200333828140076	1.22181056483609	1.047753398	1.42478283487412	0.0106226635571915
FERMT2	0.219285216546944	1.24518637380318	1.06215597371997	1.45975651775026	0.00686437082187917
FGF7	0.172071208782022	1.18776240931709	1.02217425736552	1.38017518130693	0.0246866616385304
FGFBP1	0.156977363562032	1.16996912966753	1.01178511386937	1.35288387386942	0.0341710705474797
FGFR1	0.233892785783069	1.26350901881553	1.09249134902651	1.46129764968002	0.00162081465308932

FGR	-0.157675982	0.854126489158722	0.735501958802069	0.991883231243614	0.0387538123351599
FHL1	0.201435163129642	1.22315692882457	1.05072435910951	1.42388711136335	0.00937224789361029
FILIP1	0.171195478503566	1.1867227051283	1.00490005941296	1.40144362185603	0.0436359505800361
FILIP1L	0.217870786319582	1.24342638953826	1.06136685880825	1.45671515307741	0.00699034030771135
FITM2	0.149848131317964	1.16165780989075	1.0034307083872	1.34483513011987	0.0448767912759908
FJX1	0.173944560786333	1.18998959190436	1.02192504624245	1.38569382759285	0.025146102520976
FKBP10	0.303364672110186	1.35440828932366	1.16067317092597	1.58048093135912	0.000117244733538291
FKBP14	0.220325982939974	1.24648299655774	1.07187560364608	1.44953374759389	0.00421774189478101
FKBP9	0.338778592839556	1.4032326250652	1.18574108064007	1.66061700332123	8.0566912531931e-05
FKTN	0.148770626110025	1.16040679166111	1.0044760347138	1.34054360243338	0.0433188652479716
FLII	0.155842218390086	1.16864179835806	1.00627955939539	1.35720102840023	0.041152796325341
FLNA	0.215978865442035	1.24107614912768	1.05301060612456	1.46272981390218	0.00999363016317552
FMOD	0.185936650350061	1.20434596306579	1.03568506012742	1.40047322742534	0.0157153153170853
FN1	0.304143274447369	1.35546324542557	1.1510624358229	1.59616068817861	0.000265453206128215
FNDC4	0.200277901551803	1.22174223505043	1.06320431339372	1.40392027205148	0.00473986106430876
FNTB	0.23867140385373	1.2695612950866	1.0930891099215	1.47452377610616	0.00177448419659292
FOCAD	0.177699540878766	1.19446637899519	1.02919692270887	1.38627496747138	0.0193496470644012
FOXA1	-0.159675221	0.852420592113031	0.737587889897169	0.985131230882373	0.030550311817857
FOXJ2	0.170919534261297	1.18639528100813	1.0089261875949	1.39508100801078	0.0386911262081332
FOXK2	0.208372174983833	1.23167148157118	1.05049299400894	1.44409781613722	0.0102657181422307
FOXO3	0.162712021984513	1.17669777780034	1.00871248230355	1.37265839827647	0.0384204115378919
FRMD3	-0.17368097	0.840565020103747	0.71355024907055	0.990188923544408	0.0377164001926514
FSIP2	-0.380337808	0.683630434367038	0.559341695839165	0.835536800258939	0.000203171894703437
FSIP2-AS1	-0.158561767	0.853370251782476	0.72950942446105	0.998260971289437	0.0475108761506438
FSTL1	0.225717582698158	1.25322168380874	1.06927638441271	1.46881069446889	0.00531957244034151
FTH1	0.174393735858834	1.19052422562866	1.01658963578012	1.39421835706703	0.0304532535372484

FTL	0.166991844449204	1.18174462749072	1.01516972118964	1.37565210570569	0.0312258329992481
FUT10	0.161283738263696	1.1750183191772	1.02242693375633	1.35038309811493	0.0230584372734543
FUT11	0.184984089750732	1.20319929677468	1.03631939198914	1.39695209695955	0.0151709555858231
FXN	0.22157770589017	1.24804422484035	1.06482633398609	1.4627872521959	0.0062309470270393
FXR1	0.184688122930815	1.20284324239782	1.02591841967257	1.41027964605984	0.0228946907997431
FXR2	0.152062838921401	1.16423339330423	1.00184426305972	1.35294420905806	0.0472553477632173
FXYD6	0.208699618443634	1.23207485037908	1.06383143758011	1.42692571709447	0.00533675861080954
FZD1	0.180550229394922	1.19787628857056	1.03976120356734	1.38003572146809	0.0124257559165914
G6PD	0.228656030242284	1.25690962574612	1.09254627375399	1.44599990430152	0.0013847406533172
GAMT	0.180636346470634	1.19797945061554	1.03132675023146	1.39156165955652	0.0180994958006393
GAN	0.168263866926721	1.18324878968082	1.03112005545506	1.35782219623615	0.0165560884356667
GANAB	0.300074813970632	1.34995979965095	1.14007935974431	1.59847772446503	0.000500350719458286
GAP43	0.148400418394135	1.15997727962251	1.01390969414413	1.32708790241547	0.0306850762023721
GARNL3	-0.230098702	0.794455184134321	0.67008454602816	0.941909559530975	0.00807502452006129
GAS1	0.162120679333705	1.17600215191412	1.01200726609972	1.3665722644825	0.0343678150907882
GAS6	0.222882529117495	1.24967376483327	1.08335143526588	1.44153085293996	0.00222358470767667
GAS7	0.260585219825675	1.2976892979961	1.11419574414416	1.5114018546419	0.000807691446179947
GATA2	-0.174344271	0.840007657375361	0.71887465817327	0.98155200830458	0.0282123111991894
GATA3-AS1	-0.204886772	0.814739562472305	0.702294818054616	0.945187886330012	0.00685303758300912
GBE1	0.174912036298707	1.19114143479456	1.02067485470196	1.39007825180404	0.0264421939902416
GBF1	0.172634074402276	1.18843114812971	1.01593359927823	1.39021742646205	0.0309645953382693
GBP2	-0.219022871	0.803303345742573	0.697833460745766	0.924713848762127	0.00228930402002696
GBP4	-0.153673325	0.857552115913678	0.741844268927901	0.991307289562006	0.0377071774048024
GCH1	-0.202268757	0.816875357250903	0.697063170195389	0.957281029632863	0.0124385129547357
GCLM	0.235306103729434	1.2652960212911	1.09312887087421	1.46457939603656	0.00161498070411368
GDF7	-0.162254046	0.850225181106961	0.732483142717615	0.986893508438116	0.03288887613726

GDPGP1	-0.195983674	0.822025655303176	0.705470566993987	0.957837519509691	0.0120005318462432
GEM	0.210800029439347	1.23466543363473	1.05457097009724	1.44551554730533	0.00878063362389546
GEMIN2	0.165147261363038	1.17956681053834	1.01243363774515	1.37429043114611	0.0341347036885113
GEMIN5	0.22140253315151	1.24782562066282	1.06357517627006	1.463995037	0.00660569353918656
GEMIN7	-0.178392692	0.836613827398044	0.708546546464569	0.987828816167413	0.0353415464044948
GFPT2	0.232664451127277	1.26195795970467	1.0836605686173	1.46959106770303	0.00275531529269949
GGH	0.169928728726557	1.18522037614456	1.0019581815589	1.4020019656336	0.0473908445860573
GID4	0.279833473408898	1.32290949438511	1.12979537168549	1.54903230637544	0.000509417289743836
GINM1	0.234193478116606	1.26388900341721	1.07313176385887	1.48855477654935	0.0050241733028253
GIPR	-0.187154771	0.829315372505068	0.70356737409536	0.977538203725748	0.0256980678907788
GLCE	0.232067390239609	1.26120471885275	1.08146742387887	1.47081392165411	0.00309279520981295
GLT8D2	0.189297966540338	1.20840096189882	1.04240101937768	1.40083600991656	0.012047370938095
GMPS	0.174132150584673	1.19021284275107	1.01727467783261	1.39255074555457	0.0297224028250451
GNB1	0.164058981006622	1.17828380940845	1.00152972385103	1.38623218308055	0.0478839469806023
GNG11	0.16957980605436	1.18480689802391	1.01474792786897	1.38336560938147	0.0319399835331971
GNPNAT1	0.173653036753671	1.18964273190117	1.01221150454606	1.39817599702147	0.0350958188357942
GPC2	-0.177101065	0.83769511834709	0.713399698587203	0.983646492551424	0.0306816866018217
GPC6	0.162004145911639	1.17586511634377	1.01391007496209	1.36368974525264	0.0321395094247167
GPR107	0.185524382262147	1.20384955199266	1.03442596605061	1.40102220110165	0.0165151817832181
GPR160	-0.181863552	0.833715091636676	0.718047270635379	0.968015453088059	0.0170073945944794
GPR35	-0.15691479	0.854776891490166	0.731892181489353	0.998293946437272	0.0475322607301161
GPR85	0.15298921100602	1.16531240632638	1.02046553594949	1.33071912426192	0.0238760556780103
GPRASP2	0.209231822987715	1.23273074073153	1.06709769146252	1.42407306407137	0.00448152643042282
GPRIN2	-0.15520368	0.856240760839903	0.735394202178928	0.996945907856526	0.0455685875333668
GPS2	-0.212147151	0.808845666561183	0.691826711009923	0.94565778091997	0.00779691980373203
GPSM2	0.157394696603992	1.17045749834252	1.00222116290475	1.36693457106376	0.046813076233173

GPX8	0.175147209840585	1.19142159268623	1.02268662829952	1.38799645193294	0.0245841513889603
GRK5	0.249764703119022	1.2837233250541	1.09350019747951	1.507037291	0.00227099660075051
GRN	0.161365899105693	1.1751148636377	1.00026568331021	1.38052815945102	0.0496234169148923
GSAP	-0.276698632	0.758282985171392	0.651785513284454	0.882181444480031	0.000339119482331586
GSDMB	-0.441617393	0.64299560299845	0.547213283778573	0.755543327860143	8.04399006392288e-08
GSN	0.275252313489515	1.31686289522633	1.11454095517427	1.55591221369942	0.00122012905688754
GSTM1	0.144154252062827	1.15506226552117	1.0047161211248	1.32790627041723	0.0427552113252353
GTF2IRD2B	-0.224439155	0.798964188454421	0.684709664295693	0.932283868213319	0.00436505397778247
GTF3C1	0.162998556865719	1.1770349910677	1.01602111379052	1.36356553165426	0.0298759293796809
GULP1	0.258061898974314	1.2944189393492	1.11889845781085	1.49747314320558	0.000518423745785795
HACD2	0.170624791129131	1.18604565067512	1.00864630594692	1.39464575162921	0.0390071000509507
HACD4	0.155278900655263	1.1679836670933	1.01354809243977	1.34595078099638	0.0318777329449377
HADHA	0.158088094014077	1.1712693719835	1.00468603615075	1.36547328457223	0.0434153755499146
HAS3	-0.162360952	0.850134291230176	0.734755428142863	0.98363113145305	0.0291285411759592
HAT1	0.165468681142591	1.17994600758001	1.00187454675734	1.38966758394074	0.0474343768467678
HAUS1	0.151804356459378	1.16393249828008	1.00263953943912	1.351172388	0.0460865300556513
HAUS4	0.242616889168011	1.27458022507849	1.0913312224699	1.4885991683482	0.00218726136660109
HDAC10	-0.225579931	0.798053268389753	0.682776289325627	0.932793111220394	0.00459678252154247
HDAC4	0.21627405750328	1.24144255903229	1.05277819080474	1.46391674983177	0.0101259959186089
HEATR3	0.159528647129398	1.17295786361513	1.00586515841141	1.36780774074078	0.0418965044708465
HERC2	0.162051601575999	1.17592091912813	1.01273650408334	1.3653995905823	0.0335030268542854
HERC3	0.151207579061327	1.16323809689439	1.00405353333954	1.34766008498163	0.0440261879875043
HERPUD2	0.196171763266942	1.21673587791629	1.03425150388323	1.43141797817088	0.0179726883945413
HES1	-0.168407677	0.845009274001282	0.734153828444123	0.972603622678676	0.0189192346001593
HES2	0.148767481608711	1.16040314276617	1.00475745106074	1.34015970951003	0.042913193289403
HEXA	0.161637659135099	1.17543425628471	1.00184798508881	1.37909713989704	0.0474110069882267

HIPK2	0.197013909116277	1.21776097856716	1.04576967078532	1.41803863924189	0.0112112310565702
HIPK3	0.195239829779905	1.21560248921026	1.03222810099138	1.43155317158578	0.0192755750431833
HK1	0.161437753200899	1.17519930348663	1.00730743865661	1.37107436112787	0.0401178350756436
HLA-E	-0.150650798	0.860148011878364	0.743463541112731	0.995145775717492	0.0428279274537999
HMBS	0.164005637562408	1.17822095736818	1.00044702984633	1.38758433277053	0.0493772520443342
HMG20B	-0.152292733	0.858736863697844	0.738789151791294	0.99815894600728	0.0472591691032424
HMGA1	0.194034206610764	1.21413781378552	1.02416306544507	1.43935148669248	0.025418921765215
HNF1B	-0.265418843	0.766884678469318	0.658480364314817	0.893135379492978	0.000641526108475589
HNMT	-0.212830787	0.808292898972631	0.699962263298985	0.933389476527408	0.00374519376573632
HOXB-AS3	-0.211313295	0.809520408271482	0.680242423539731	0.963367277209743	0.017295440363815
HOXB3	-0.255461146	0.774559230906961	0.660050645899471	0.90893328551421	0.00174937034155679
HOXB5	-0.240219668	0.786455082896723	0.672115665643525	0.920245768742633	0.00272777560623636
HOXB6	-0.193810586	0.823813931060286	0.699299064879843	0.970499500275484	0.020442712393346
HS6ST2	-0.146540675	0.863690601177692	0.747423229061043	0.998044247968857	0.0469776027407536
HSD17B1	0.160842371597797	1.17449981969191	1.02882739985295	1.34079810340732	0.0172845185460119
HSD17B2	-0.231164616	0.793608814225772	0.678653518781139	0.928036078186086	0.00378658046033213
HSD3B1	-0.169037281	0.844477419714371	0.714828042048103	0.997641489223295	0.0468414741550944
HSP90AB1	0.166249172730824	1.18086730499916	1.01032736881184	1.38019382139067	0.036701315767143
HSP90B1	0.16532094880837	1.17977170427748	1.0066571625741	1.38265670375275	0.0411599050110066
HSPA5	0.197697279550923	1.2185934448254	1.02759480725772	1.44509292308928	0.0230308421963508
HSPA8	0.182652491643278	1.20039718753716	1.01033873495913	1.42620821907359	0.0378088489515343
HSPB2	0.176443700802964	1.19296726177002	1.02730684875753	1.38534157479462	0.0207142617024344
HSPB6	0.249534485203589	1.28342782296246	1.10429379911823	1.49162023554729	0.00114032790859763
HSPB8	0.173494226262804	1.18945381915602	1.01755347105626	1.39039414453201	0.0293718091751381
HSPG2	0.30201273202835	1.35257844766699	1.15031488594258	1.590406661	0.000257751361354615
HTRA1	0.227059738931183	1.25490483237453	1.08211917474158	1.45527976499726	0.00266330654726617

HTRA3	0.152670203685109	1.16494072242571	1.00554613366522	1.34960181470654	0.0419882176231004
HYOU1	0.216408909886642	1.24160998180861	1.04948960994548	1.46890005610142	0.0116309435003369
HYPK	-0.18894732	0.827830115338788	0.706690061846522	0.969735867052089	0.0192494896135002
IAH1	0.161013586937305	1.17470092929332	1.00605901556371	1.37161165690603	0.0417140171579073
IARS2	0.214630749466627	1.23940416181397	1.05323115314405	1.45848579557892	0.00975297100854029
ICMT	0.239629743560111	1.27077854926371	1.07864910951416	1.49713016682148	0.00416736345713595
ID1	-0.145538001	0.864557035253175	0.75294447078673	0.992714464620162	0.0390512047095785
ID2	-0.173947517	0.840340999939152	0.723339068463025	0.976268290995582	0.0229696811641592
IDI1	0.193983568310638	1.21407633346716	1.04315824475162	1.41299879562954	0.0122188227819748
IDUA	-0.197635936	0.820668574717652	0.710184432146866	0.948340852098293	0.00738572389927799
IER3IP1	0.264671042987154	1.3030022735343	1.11892713235997	1.51735968834237	0.00065907260329586
IFT122	0.215196733901326	1.24010584382968	1.06968239666404	1.43768141711649	0.00433042693234523
IFT88	0.146692081448629	1.15799733926394	1.00337060517532	1.33645318173145	0.0448585217766977
IGF1	0.216990287115539	1.24233203545164	1.08190455407933	1.42654810028302	0.00209885075147662
IGF2	0.149301210450171	1.16102264870057	1.00453722646279	1.34188515396509	0.0432538562649862
IGF2BP2	0.170162668058723	1.18549767824249	1.02624333474955	1.3694654060397	0.0207819961691333
IGF2BP3	0.19063259797829	1.21001480851788	1.0468426807297	1.39862069419254	0.00989784644194343
IKBIP	0.168528250069534	1.18356166207183	1.01287264254047	1.3830151482942	0.0339274654542804
IKZF3	-0.2598675	0.771153756944264	0.664002633430022	0.895596021626208	0.000662657102747932
IL1R1	0.169146261167541	1.18429334238416	1.01591234595433	1.38058241579682	0.0306392857611015
IMPDH1	0.285190350487129	1.33001517306366	1.13033908460838	1.56496434093706	0.000590110345498975
IMPDH2	0.156399225926417	1.16929292197056	1.01135132372955	1.3519000818909	0.0346506262912087
INHBB	0.218068802341743	1.24367263226497	1.06624338723053	1.45062715958534	0.00549167077876227
INSIG1	0.180012391200728	1.19723219817438	1.02963190659223	1.39211394593381	0.01931114482096
INTS5	0.213363561882785	1.23783459892409	1.0523946365607	1.45595049714528	0.00997513785958044
IPCEF1	-0.214237089	0.80715699402931	0.687336317747149	0.947865544404568	0.00897475034700117

IPMK	0.165794902933469	1.18033099447188	1.02250117597275	1.36252288921387	0.0235868174551766
IPO11	0.203663582179876	1.22588567429116	1.04410747393882	1.43931130074485	0.0128809794126232
IPO13	0.185670992741358	1.20402606189116	1.03800052479762	1.39660696028625	0.0141815561356224
IPO7	0.18211898688083	1.19975694072333	1.02198558888437	1.40845109018133	0.0260311091447497
IPO9	0.179619742527118	1.19676219881812	1.0259334670493	1.39603571432293	0.0222672832762674
IPPK	0.183796186497792	1.20177086100583	1.04241730975631	1.3854846699546	0.011330672053499
IRAK2	-0.230841724	0.793865105715891	0.680915123350769	0.925551194944819	0.00319873894853968
IRF5	-0.183755663	0.832139101045545	0.710314141022771	0.974858085313959	0.0228909572751824
IRF9	-0.189504031	0.827369381475955	0.705654785360305	0.970077873211588	0.0195881259238844
ISLR	0.225676140681852	1.25316974885144	1.07840870157958	1.45625162068529	0.00322894170879206
ITFG1	0.205126409715471	1.22768024586535	1.04657655987597	1.4401228193632	0.0117682460378606
ITGA5	0.216189814128828	1.24133798012703	1.05923269916946	1.45475114402538	0.00756492868286841
ITGAE	-0.149867797	0.860821771816461	0.743010743904363	0.997312796500572	0.0459559373702069
ITGB7	-0.27201336	0.761844083092761	0.650005182543626	0.892925814333018	0.000784817985095478
ITGBL1	0.218066030469067	1.24366918496756	1.07633745926831	1.43701496990481	0.00309887826394768
ITPRIP	0.213684046966817	1.23823137002576	1.05707480945432	1.45043369873448	0.00810358858675387
IZUMO4	-0.165152815	0.847764142742091	0.727849450445529	0.987435026954134	0.0337986109154542
JUN	0.241784423947021	1.27351962288821	1.07700940537377	1.50588492708518	0.00469040596178141
KCNJ15	-0.256607497	0.773671822804663	0.672513630243655	0.890046033988969	0.000331663830034285
KCNK5	-0.196547685	0.82156215416513	0.708322514987873	0.952905433435228	0.00939121988534799
KCNK6	0.186758256063034	1.20533586718919	1.03855106264925	1.39890526810177	0.013980389500877
KCTD16	-0.344125129	0.708840226380071	0.556982554719225	0.902100904736303	0.00514956997776177
KCTD3	0.186371065598004	1.20486926297244	1.03035127970344	1.4089466082612	0.0195700945753761
KCTD9	0.178966965064769	1.19598123435267	1.02330488441121	1.39779564694128	0.0244784972026815
KDELR2	0.292354104613738	1.33957728416114	1.13134899109744	1.58613064082008	0.000694631011422972
KDELR3	0.210725828849167	1.23457382412966	1.04821207663964	1.45406884846464	0.0116052281667063

KDSR	0.23359494602597	1.26313275163272	1.07713394119348	1.48124971949116	0.0040502869222594
KIF12	-0.155994569	0.855563837110455	0.732491660628685	0.999314420512188	0.0489968795949991
KIF16B	0.183813360411416	1.20179150029202	1.02900341571236	1.40359379582261	0.0202882525806198
KITLG	0.187324838113277	1.20601898235818	1.04045919568305	1.39792294771676	0.0129039322633965
KLHDC10	0.166297494750091	1.18092436827052	1.01488340433631	1.37413062191823	0.031471256797481
KLHDC7A	-0.20898222	0.811409662480713	0.698276875928421	0.942871893747996	0.00637603563173886
KLHL20	0.169302341243843	1.18447820140543	1.01446425812856	1.3829847610332	0.0322239359758964
KLHL21	0.15789180537905	1.1710394876798	1.00488227494602	1.3646707837284	0.0431406091541838
KLHL3	-0.173987984	0.840306994810842	0.720254358771595	0.980370110820739	0.0269658065996794
KLHL7	0.176007205632696	1.19244665095229	1.01785510202128	1.39698569329134	0.0293264633965645
KLRC1	-0.271112117	0.762530999108236	0.631073122377694	0.921372665041196	0.00498205970220718
KNSTRN	0.176514828559987	1.19305211787333	1.01035564599318	1.40878448257975	0.0373928274803081
KPNA2	0.230557430411357	1.25930178742863	1.05321795925106	1.50571016938283	0.0114509041051139
KPNA6	0.220135533627266	1.24624562733187	1.06496828829319	1.45837972897106	0.00605463737903602
KPNB1	0.180341951693731	1.19762682363076	1.02297345454846	1.40209895213069	0.0249364431492085
KRT23	0.203502619510216	1.2256883683402	1.05042296310665	1.43019719584324	0.00974390599669091
KRT4	0.21481312483409	1.23963021921644	1.0727249083641	1.4325043339751	0.0035977070916718
KRT5	0.155330187765166	1.16804357113613	1.00720169139218	1.35457058475215	0.0398891255143266
KRTAP5-10	-0.212685828	0.808410077081598	0.673860533741227	0.969825089916958	0.0220306894528257
L2HGDH	0.175619386652111	1.19198428717023	1.02582059927923	1.38506337449165	0.021860438474956
LAMA4	0.187154414199319	1.2058134653959	1.03064772330754	1.41074984250094	0.0194444369745601
LAMB2	0.180735607490596	1.1980983691796	1.02352559214766	1.40244632204929	0.0244894008580574
LAMC1	0.221385082694225	1.24780384572512	1.06272828441558	1.4651105651739	0.00687786379118418
LAMP1	0.158300180012608	1.17151780816179	1.00122444100186	1.37077554106337	0.0482417206155105
LAMP2	0.198900321551659	1.22006034611635	1.03697560187593	1.43546988518603	0.0165009144869357
LAMP5	0.139855492809456	1.15010758803182	1.01030389045323	1.30925702310714	0.0344308809063571

LAT	-0.209316478	0.811138487693434	0.688019140921867	0.956289741206936	0.0126994803579268
LCMT1	0.150474886165087	1.16238611276363	1.01561903459349	1.33036249727887	0.0288880074151479
LCOR	-0.171953398	0.842018412074615	0.726786147081521	0.975520803636245	0.022019712868557
LDB2	0.190152826791585	1.20943441751584	1.03591053749277	1.41202503240497	0.0161081610759948
LDLR	0.269266725201633	1.30900423890736	1.11743598173539	1.53341410647648	0.000851906187982542
LEAP2	-0.237314703	0.78874302895519	0.648744001542714	0.958953861994896	0.0172956730823657
LEO1	0.1786211175645	1.19556767875011	1.01838449959819	1.40357799537983	0.02906797907828
LGALS1	0.246865032126636	1.28000634138545	1.09650135944105	1.49422179907023	0.00176704697965963
LGALS3	0.168649753952119	1.18370547814598	1.02162885760195	1.37149479340446	0.0247821442242621
LGALS7	0.165129157847148	1.17954545642514	1.04538197368992	1.33092737275943	0.00735388872192258
LGALS7B	0.165129157847148	1.17954545642514	1.04538197368992	1.33092737275943	0.00735388872192258
LGALS9	-0.176694071	0.838036125140596	0.727814688083971	0.964949675431167	0.0140543598867358
LGALSL	0.164667885311771	1.1790014899699	1.02018586045795	1.3625404617226	0.0257016370950236
LINC-PINT	-0.164771957	0.848087081953622	0.727254447532933	0.988995943602035	0.0356358909520341
LINC00240	-0.20734736	0.812737288798064	0.692064033612266	0.954451999412676	0.0114573454447325
LINC01089	-0.213185352	0.808006357459132	0.688207638569634	0.948658859775669	0.00922308685684186
LINC01116	0.226693246007819	1.25444500290124	1.08359842985258	1.45222826275042	0.00240705694808539
LINC01138	-0.267238881	0.765490188471568	0.65678768640502	0.892183639820684	0.000626414715525482
LINC01139	0.175248975956427	1.19154284520364	1.04652956603418	1.35665001547565	0.00812428217473579
LINC01589	-0.214880309	0.806637982062557	0.686795504761044	0.947392389139675	0.00883135088503333
LIX1L	0.188899263266676	1.20791926451295	1.03627597530353	1.40799264322821	0.0157073493461138
LLGL1	0.157422501704607	1.17049004348349	1.0050082670434	1.36321957422746	0.0429494169355815
LMAN1	0.291212465941412	1.33804884355843	1.13082559954814	1.58324564677651	0.000693651403817147
LMBR1L	-0.168438745	0.844983021632021	0.730762564238651	0.977056491105646	0.0230134141576384
LMCD1	0.176253116883313	1.1927399230575	1.02483792235663	1.38814976790071	0.0227915731947433
LMNA	0.187768788212203	1.20655451347045	1.02188301646149	1.42459926481299	0.0267351409117607

LOX	0.265801212527551	1.30447571947994	1.11907436294857	1.5205932322756	0.00067800056837874
LRFN2	-0.179784793	0.83544998654814	0.707077347822492	0.987129176422876	0.0346731528609774
LRP10	0.168131177603446	1.1830917956156	1.01094956022183	1.38454602675311	0.0361083506173004
LRP8	0.193933619006448	1.21401569271356	1.04549553588766	1.4096990867622	0.010975745163828
LRPPRC	0.164847459728897	1.179213227	1.00617829341141	1.38200540101434	0.0417465825562656
LRRC17	0.174877060048384	1.19109977386214	1.04073866331907	1.3631843625084	0.0110878258039947
LRRC36	0.131222242510036	1.14022115876765	1.00127847508892	1.29844426225779	0.0477895534731687
LRRC59	0.32471516657392	1.38363648392799	1.15930990038807	1.65137028418005	0.000320820647683548
LRRC69	-0.181394349	0.834106364845695	0.710723742768631	0.9789083803024	0.0263530345339668
LRRC8A	0.159205812239822	1.1725792530104	1.01193260788959	1.35872892509897	0.0341961031548111
LRRN4CL	0.155377231127805	1.16809852112594	1.00213963348612	1.36154095643349	0.0468890483893916
LSAMP	0.149670644840713	1.16145164963417	1.00750010395838	1.33892783647163	0.039116962925896
LSM3	-0.145815325	0.86431730659168	0.747722802577057	0.999092717112782	0.0485820888982605
LSS	0.22844202519271	1.25664066951938	1.07877335791823	1.46383460501609	0.00334916330677164
LTA4H	0.192814874501125	1.21265827876924	1.03897796444842	1.41537178976482	0.0144933909944764
LTBP2	0.201321947507771	1.223018456	1.05257824591189	1.421057437	0.00855940324922322
LUC7L	-0.209470531	0.81101353901134	0.692675284011077	0.949568976463117	0.00924108901957814
LUM	0.197287278748652	1.21809392294456	1.04118948996633	1.42505549605812	0.0137355788191738
LURAP1L	0.157237683097345	1.17027373513335	1.00616944760534	1.36114311401766	0.0413751642609367
LYPD6	-0.181263709	0.834215339573684	0.72189387273624	0.96401321449407	0.0140223716920775
LYPD6B	-0.179826094	0.835415482891788	0.719754045461614	0.969663225175357	0.0180231365221882
MAFG	0.302935541416814	1.35382719584679	1.15157774307238	1.5915973430716	0.000242977453099208
MAGI3	0.201795513112164	1.22359777282678	1.06122897627469	1.41080911201876	0.00546780571980387
MAML2	0.158478570805611	1.17172681479448	1.00513513482267	1.36592949638621	0.0428237100616644
MAN1B1	0.245548364508716	1.27832210751643	1.09476580867468	1.4926547738493	0.00190418395225988
MAN2A1	0.217908864993492	1.24347373846777	1.06845685564356	1.44715898455972	0.00487007743208701

MAP1A	0.315689365620002	1.371204246	1.17880661923649	1.59500384061263	4.26651111076693e-05
MAP1B	0.341797767578681	1.40747563152582	1.2064384385357	1.64201304439819	1.38301752206967e-05
MAP3K4	0.148610542614921	1.16022104455408	1.00137931185765	1.34425872023359	0.0478934279107302
MAP4	0.199313688660518	1.22056478318593	1.03968040156124	1.43291956616338	0.0148731081841904
MAP7D1	0.229671871999813	1.25818709577324	1.07642060194333	1.47064703621646	0.00391410971624576
MAP7D3	0.325094967563578	1.38416209024049	1.1915669925442	1.60788667699509	2.11141938406487e-05
MAPK3	0.246983792852182	1.28015836489429	1.09660233873036	1.49443912467505	0.00176125535531085
MAPKBP1	0.172679292089975	1.18848488745319	1.01973282785435	1.38516314187581	0.0271011674532506
MARVELD1	0.195744351456794	1.21621594175358	1.03895934515646	1.42371424240167	0.0148711451908256
MAZ	0.208567737896217	1.2319123743873	1.0429047191054	1.45517425548747	0.0141154084067528
ME1	0.227084416054335	1.25493580019772	1.07144059742108	1.46985644	0.00486993500309688
MECOM	-0.155744426	0.855777877291988	0.742885632901208	0.985825735251193	0.0309476716801735
MECR	0.218353702784582	1.24402700562681	1.06051030675172	1.45930047155225	0.00733085110051112
MED8	0.176258074603969	1.19274583634351	1.02177416770595	1.39232589262738	0.0255595481940354
MEST	0.208947866401794	1.2323807484127	1.05897676873388	1.4341790621848	0.00692165623597356
METTL8	0.174535019483493	1.19069243908913	1.02155276401452	1.38783676619162	0.0255654464743547
MFAP2	0.166948259465134	1.18169312229239	1.00818638863057	1.3850599958703	0.0393417027541148
MFAP3	0.158412183906212	1.17164903006627	1.00497579457083	1.36596468996696	0.0430359392773811
MFAP3L	-0.186456549	0.829894621098557	0.715020619578068	0.963224085110626	0.0141717524893698
MFAP4	0.20057976794445	1.22211109364188	1.04384619337189	1.43081953518265	0.0126524651985415
MFAP5	0.241877356557946	1.27363797989136	1.10058936889245	1.47389548697364	0.00116890955802991
MFSD11	0.30109628488128	1.35133944883263	1.14832112816615	1.59025055028602	0.000288927661939176
MFSD2A	-0.25863516	0.772104666577741	0.66124429547534	0.901551242453563	0.00107360763680109
MGLL	0.153297537030489	1.16567175786375	1.00105530213789	1.35735822404545	0.0484337631583374
MGP	0.161659008435886	1.17545935125208	1.0118073428718	1.36558080565445	0.0345647459336017
MIB1	0.175096566084818	1.19136125614992	1.00974328754913	1.40564602920032	0.0379993941523285

MIPEP	0.210802030208862	1.23466790391816	1.0598560181921	1.43831313574648	0.00680424205977691
MIR181A2HG	-0.174923257	0.839521445458608	0.720742268806382	0.97787557062821	0.0246138518892243
MIR4435-2HG	0.190972913339706	1.21042666522137	1.041485149	1.40677254384006	0.0127766275842692
MIR99AHG	0.170009495895374	1.18531610690466	1.04246153814232	1.34774686823584	0.00946973675016897
MLF1	0.198183729994386	1.21918637435076	1.04650385660681	1.42036305553818	0.0109815301311969
MLLT11	0.196788734222741	1.21748680023866	1.05407019418377	1.40623851896617	0.0074494637675706
MMEL1	-0.187448205	0.829072058814706	0.708770326766841	0.969792967833105	0.0191075995162543
MMGT1	0.174036487380198	1.19009898862244	1.00923894740122	1.40336994164484	0.0385168500886194
MMS19	0.252725249917934	1.28752947940379	1.09848837161641	1.50910305759038	0.00181211369314502
MOGAT2	-0.212667069	0.808425242199299	0.67838100531199	0.963398690569798	0.0174693067852479
MORC4	0.1589405883288	1.17226829819308	1.00518352783802	1.36712642506608	0.0427788482714771
MORF4L1	0.232997336744929	1.26237811728786	1.06824757024209	1.49178762994622	0.00624006369521383
MPI	0.144557850002532	1.15552854035922	1.00037895383953	1.33474040258437	0.0494009873974071
MPRIP	0.16821663891291	1.18319290851023	1.02302596411374	1.36843590275998	0.0234072881701506
MRC2	0.17996478679388	1.19717520600228	1.03046757452953	1.39085256954442	0.0186603628982515
MRFAP1L1	-0.177115022	0.837683427218605	0.737156067254215	0.951919892419085	0.00661947883705558
MRGPRF	0.182690616546817	1.20044295343655	1.03180647579773	1.39664105455563	0.0180125402309334
MRPL37	0.232997896059634	1.2623788233547	1.07350761209374	1.48447973326085	0.00483570834529164
MRPS27	0.160265556465796	1.17382254577308	1.00824151928502	1.36659653724862	0.0388512891841055
MRTO4	0.189274594090826	1.2083727189384	1.02888699305412	1.41916909994193	0.0210503867799192
MSANTD3	0.182440295094139	1.20014249441983	1.02105223591365	1.41064478020892	0.0269228209149976
MSC	0.17066010464526	1.18608753485687	1.0236983004045	1.3742365692968	0.0231019573401705
MSC-AS1	0.180567871287185	1.1978974215614	1.0449154468367	1.37327688754869	0.00959149369974737
MSH6	0.187587103586962	1.2063353209784	1.03296144370672	1.40880854315142	0.017806291636904
MSRB3	0.250858670244012	1.28512844460646	1.10282817210916	1.4975634109691	0.00130917820886353
MST1R	-0.229450224	0.794970537814323	0.687341074291787	0.919453499332864	0.00199240141434299

MSX1	0.237029383609296	1.26747836031705	1.09936631038164	1.46129763910476	0.00109533605182117
MT1A	0.204942218222516	1.22745413843217	1.05884395722055	1.42291378411335	0.00656034222047507
MTERF2	-0.192724234	0.824709369515905	0.706982509459439	0.962040128386434	0.0141903340662255
MTHFD1	0.178821747129242	1.19580756903685	1.03042046614597	1.38774004316337	0.0185457874005149
MTHFD1L	0.222695222083836	1.2494397140677	1.06744646268232	1.46246172868172	0.00556152725778403
MUC4	0.137754154173389	1.14769335996517	1.00044336539547	1.31661630639877	0.0492649894337904
MXRA5	0.172269214025733	1.1879976157877	1.02242874682841	1.38037818233815	0.0244731497442334
MXRA7	0.373019254834805	1.45211229974604	1.23789183641006	1.70340418205589	4.6368219897883e-06
MXRA8	0.196375301934697	1.21698355592106	1.05347977176637	1.40586370528881	0.00763693152136449
MYADM	0.233228474663322	1.26266993446183	1.07137449573601	1.48812144561884	0.00539485372936616
MYC	0.218334008582461	1.24400250574877	1.05958461504249	1.46051784099108	0.00765489287803145
MYH10	0.261232974472773	1.29853015457434	1.11946590187992	1.50623664330218	0.000559302944326502
MYH9	0.168488462693757	1.18351457219603	1.01491675188607	1.38011983741261	0.0316504053033666
MYLIP	-0.183240771	0.832567673818113	0.715394084035984	0.968932993653379	0.0178957510303724
MYLK	0.177769210550083	1.19454959997417	1.02472408828349	1.39251996036193	0.0230788090501589
MYO1D	0.196247677663467	1.21682824919231	1.0390041783876	1.42508665396343	0.0149061124849542
MYO5A	0.262211450926256	1.29980135757441	1.11025559167311	1.52170687706809	0.0011121328086176
MYO9A	0.20388607116936	1.22615845069981	1.04213102579806	1.44268283834195	0.0139968518042162
MZF1	-0.273012497	0.761083276483889	0.649507440658186	0.89182620164669	0.000737125371110656
NADSYN1	-0.245732876	0.782131122928753	0.660471013980891	0.926201272280954	0.00438980425863197
NAMPT	0.183098784468172	1.2009330357527	1.01738087084695	1.41760101618733	0.0304966003098011
NAT8	-0.296522246	0.743399089680208	0.616149749159683	0.896928396531957	0.00196430331213662
NAV3	0.211936751522208	1.23606970307892	1.07384707675138	1.42279878015009	0.0031518537970544
NBAS	0.325765267344187	1.38509020480865	1.18612739916173	1.61742733269016	3.83207383581373e-05
NBPF11	-0.197581141	0.820713544766619	0.70331723991031	0.95770540567054	0.0121190285632288
NBPF12	-0.178993738	0.836111135108315	0.719681876264082	0.971376177876115	0.0193068291603911

NCAM1	0.241322022701694	1.2729308819564	1.09956323284261	1.47363333170876	0.00123549535681716
NCAPG2	0.173303917236893	1.1892274768965	1.01247199429357	1.39684060376642	0.0347774319006113
NCBP1	0.162523714252104	1.1764762173715	1.00670185631443	1.37488202823821	0.0409566850636422
NCDN	0.178957038088444	1.1959693619342	1.03151384076944	1.38664422924114	0.0177373229534962
NCL	0.197900448103748	1.21884104984399	1.03286871521054	1.43829848160517	0.0191387268489082
NCOR1	0.182875031637188	1.20066435364644	1.03245574145705	1.39627766327568	0.0175621207373339
NDC1	0.176781394086381	1.19337018683037	1.01132192902561	1.40818898704953	0.0363246973025313
NDUFA3	-0.199223211	0.819366981025958	0.693086629410255	0.968655606827754	0.019654538535247
NDUFS1	0.168255404659286	1.18323877675548	1.0129413964461	1.3821668338661	0.0338258348667792
NEBL	0.288553317258449	1.33449549926756	1.14106918790867	1.5607101273406	0.000304146621810425
NEGR1	0.202774148251174	1.22479581473062	1.04558955321805	1.43471669467686	0.0119944687503458
NEMF	0.179700948027669	1.19685938643754	1.01706912175257	1.40843169875738	0.0304830205649385
NEURL3	-0.188503291	0.828197777653167	0.705468742352633	0.972277746314077	0.0212498878892573
NFASC	0.152820221509906	1.16511549740826	1.00042645988753	1.35691545228972	0.0493624559269031
NFE2L1	0.170291263055677	1.18565013711533	1.01037158892157	1.39133588380296	0.036944166033145
NFIA	0.164619108897659	1.17894398390747	1.00484507562955	1.38320717382309	0.0434611225476058
NFIC	0.230023999718783	1.25863021633825	1.07205318699284	1.47767857108217	0.00495616833854019
NGF	0.235784574925101	1.26590157384945	1.10319821974819	1.45260096144852	0.000781678604709867
NHS	-0.220950845	0.801756089767652	0.689496467542092	0.932293141067141	0.00409296992922879
NHSL1	0.184285883250441	1.20235950841149	1.04334468543264	1.38560957625239	0.0108893297129508
NHSL2	0.170083778005024	1.18540415795595	1.02480328692538	1.37117341018206	0.0220321566395374
NID1	0.250738082818698	1.28497348361949	1.10303399526266	1.49692290599984	0.00128692727338355
NIN	0.179451923144391	1.19656137577611	1.03031091189436	1.38963793304561	0.0187128994304111
NIPAL4	-0.178845659	0.836234954445141	0.719419167022564	0.97201872161676	0.0198249293604566
NKIRAS1	0.212422623858485	1.23667042107767	1.06766089285555	1.43243396906486	0.00460894370178567
NLE1	0.209010254432482	1.23245763661908	1.05350138795625	1.44181283805178	0.00902607070828128

NLGN2	0.157861536429035	1.17100404208053	1.00672677505637	1.36208800693929	0.0406685025243097
NLN	0.227281128293234	1.25518268571056	1.05781797291326	1.4893711534969	0.00921598820535056
NLRX1	0.156275925067408	1.16914875603692	1.00223087537873	1.36386619822119	0.0467767470366
NOL12	-0.197300523	0.820943883689394	0.694102057355571	0.970965080747165	0.0212198502029974
NOL9	0.155605375152894	1.16836504622614	1.00926001002292	1.35255223399966	0.0372171291039616
NOLC1	0.165787380570688	1.18032211562733	1.00705976503647	1.38339386102723	0.0406759661590923
NOMO1	0.260767113411117	1.29792536082381	1.11495882559982	1.51091699853879	0.000769396508870463
NOTCH3	0.254902195220702	1.2903354137308	1.10451357780919	1.50741965819045	0.00131395257833839
NPAS2	-0.198543875	0.819923795612847	0.708508500955824	0.948859512208017	0.00771179957282019
NPC1	0.214312306306235	1.23900954487055	1.06946927314315	1.43542660909609	0.00430997129378434
NPHP4	0.189616882326507	1.20878640149971	1.04071385693757	1.40400221896754	0.013049543857303
NPM1	0.201385119815339	1.22309571952951	1.04848663274488	1.42678322489915	0.0103946833509879
NR2C1	-0.168653559	0.844801526444486	0.729533970731919	0.978281543718808	0.0242379686715158
NR2F1	0.214186831740031	1.23885409043838	1.0668517939715	1.43858731462835	0.00497743427188436
NR2F1-AS1	0.275740342974731	1.31750571999315	1.13901303174406	1.52396967711314	0.000205322316326243
NR2F2-AS1	0.165026388047174	1.17942424100327	1.02258406552074	1.36031998460466	0.0234075863388985
NR2F6	-0.16391839	0.848811290389055	0.72257218555848	0.997105370358352	0.046015716286229
NRBF2	0.186119025515363	1.20456562588979	1.02885693573059	1.41028193200149	0.0206897217423749
NRDC	0.202549708623676	1.22452095286031	1.05832479604834	1.41681605646271	0.00649519847467325
NREP	0.208873828911078	1.23228950941208	1.06472443315265	1.42622577985805	0.0050947595129169
NRP2	0.251427297605997	1.28585941160795	1.10339183296499	1.49850159936177	0.00128176360157193
NSMF	0.183331233933279	1.20121222444186	1.02800487839494	1.40360307472609	0.0210202610088598
NT5C3B	0.192817428266595	1.21266137561803	1.02248313005513	1.43821210217574	0.0267306387034171
NT5DC2	0.167741658826979	1.1826310488875	1.0172865092995	1.37484984319297	0.0290350454384466
NT5DC3	0.235677666119184	1.26576624505783	1.0727775129311	1.49347294086197	0.0052335676085458
NUBPL	0.170299976370214	1.18566046810291	1.02352115647787	1.37348479484254	0.023217635051359

NUDT1	0.175821298789983	1.19222498756532	1.01045978208887	1.40668678374981	0.0372279674684351
NUDT11	0.217838868298223	1.24338670246157	1.08190002445917	1.4289772223927	0.00214793246763067
NUDT15	0.161283856687694	1.17501845832758	1.00620265936316	1.3721573527587	0.0415395994959485
NUFIP1	0.154481725032513	1.16705295000789	1.00624225439916	1.35356330164787	0.0411287891567792
NUMBL	0.255881968922542	1.29160026997098	1.11754821165513	1.49275999012016	0.000530520583994006
NUP188	0.244007856029935	1.27635435752811	1.09406143217872	1.48902099833354	0.00191400952472478
NUP205	0.17576746764436	1.19216081045577	1.02206888126615	1.39055930968753	0.0252281029385219
NXPE3	0.180483631828209	1.19779651558089	1.02988288996638	1.39308702641381	0.019177595343857
NXPH4	0.228364445249083	1.25654318318862	1.08954533392052	1.44913728879588	0.00169719243663925
OCIAD2	-0.248449448	0.780009290880168	0.658412837551149	0.924062319504999	0.00406183640569447
ODF2	0.162897589798985	1.17691615529655	1.00347229276215	1.38033869653274	0.0452227566992899
OFD1	-0.267323699	0.765425263981417	0.651121696320723	0.899794674408203	0.00119728479880992
OGFOD1	0.215759317325982	1.24080370310586	1.05563672378406	1.45845042612989	0.00888133214062857
OGN	0.199569477041032	1.2208770294079	1.04778825945416	1.42255909768673	0.0105154177358844
OGT	-0.174724944	0.839687950151105	0.718893192752321	0.98077970515974	0.0274633909788626
OLFML1	0.184162718864301	1.20221142965991	1.03022933394736	1.40290348370025	0.01938427555236
OLFML3	0.173073166674642	1.18895309364583	1.02015113007495	1.38568631373875	0.0267371698328931
OMD	0.165494998205898	1.1799770607024	1.02126580204818	1.36335306733221	0.0247374215021059
OPHN1	0.147247785526186	1.15864102193915	1.00080187110334	1.3413734091446	0.0487591699157314
OS9	0.175527397268566	1.19187464231362	1.0248553971588	1.38611277935253	0.0226854324606382
OSBPL10-AS1	0.146704564217295	1.15801179436706	1.01723466406587	1.31827135199394	0.0265307755637264
OSCP1	0.166609735289014	1.1812931583043	1.02328026718159	1.36370608386697	0.0229613605168437
OSR2	0.20416303667767	1.22649810133196	1.0509877636306	1.43131789410598	0.00956662280695945
OXA1L	0.176315382221232	1.19281419172401	1.03170199110001	1.37908592621907	0.017241325865834
OXCT1	0.20434814162757	1.22672515321512	1.05247281308645	1.42982752886278	0.00894286467414236
P2RX6	-0.207736166	0.812421352524946	0.682346779577131	0.967291813771732	0.0196207961063469

P2RY1	0.16431750411417	1.17858846237865	1.02239305855339	1.35864651273894	0.023496296354739
P2RY11	-0.185966306	0.830301570941509	0.714333668267402	0.965096185904356	0.0154002358819165
P3H1	0.235338040122919	1.26533643092797	1.09616782086155	1.46061237427601	0.00130937606509101
P3H3	0.225311393889317	1.2527127425559	1.07465645	1.46027059610209	0.00397113043791738
P4HA2	0.251418926796156	1.28584864796838	1.1064003796015	1.49440182411871	0.00104384297608832
P4HB	0.37706774664872	1.45800308084802	1.22174735460777	1.73994482226219	2.90964533765928e-05
PACRG	-0.175017151	0.839442623546392	0.70701896122734	0.99666905255723	0.0457107328261583
PAFAH1B1	0.189020225261194	1.20806538567378	1.03123933608185	1.41521170207505	0.0192358352234332
PAG1	-0.171625794	0.842294305405212	0.720946052550387	0.984067662772125	0.0305941674310704
PAK6	0.157828239930891	1.17096505239573	1.01934570113551	1.34513654435852	0.0256950107596104
PALLD	0.240115344910472	1.2713957908978	1.07722552025766	1.50056531962407	0.00451528341058368
PAM	0.18776806734505	1.20655364370525	1.02618327582591	1.41862738307322	0.0230371188118992
PAM16	-0.243719336	0.783707562092969	0.670207570338159	0.916428834982879	0.00226354005363747
PANX1	0.171943806860239	1.18761109574254	1.01510814744521	1.38942842521805	0.0317755751146559
PAPSS2	0.172290980337591	1.18802347439572	1.02727479366247	1.37392622151597	0.0201928822069254
PAQR4	0.198097494756095	1.21908124205636	1.03941035275558	1.42980976742604	0.0148871359377807
PAQR6	-0.255208279	0.774755116169177	0.663074877255948	0.905245411369459	0.00131161292932184
PARD3	0.265355948506289	1.30389501266897	1.1142871822321	1.5257666346456	0.000933994533446917
PARN	0.238973171655278	1.2699444656189	1.0825311808416	1.48980368815083	0.00335276030678595
PARP2	0.158756187945577	1.17205215139904	1.0012220183675	1.3720296002269	0.048250176016537
PARP8	-0.206794427	0.813186802352177	0.706502049535203	0.935981397300687	0.00395157227225527
PARVB	0.179892099320407	1.19708818952379	1.02610081227326	1.39656855969404	0.0221596271478686
PATZ1	-0.161074156	0.851228945238041	0.734898369561847	0.985974043789314	0.0316849561673561
PBX4	-0.202496807	0.816689090305803	0.697803831465844	0.955828902262438	0.0116427939264384
PCDHB11	0.203063530387918	1.22515030004895	1.05794636798866	1.41878010372462	0.00668031487291716
PCDHB13	0.184670143472576	1.2028216161224	1.04247896596549	1.38782640940036	0.0114100803862887

PCDHB2	0.184061996306489	1.20209034594772	1.04451409377128	1.38343868066288	0.010244542461715
PCDHB5	0.187877909688487	1.20668618166396	1.05678326975737	1.37785256701978	0.00550292119862526
PCED1B	-0.315453257	0.729458167064928	0.629566549551327	0.845199316699629	2.68994999200056e-05
PCLO	0.243643014607231	1.2758887755254	1.11715015226638	1.45718296167187	0.000325402526581463
PCMT1	0.220445701398473	1.24663223251361	1.065346921	1.45876605305554	0.00596921895083311
PCOLCE	0.156070040144246	1.16890807071271	1.009124784	1.35399119991867	0.0374261412330911
PCOLCE2	0.290403294687896	1.33696657082946	1.14352006709063	1.56313794830311	0.000270766978192511
PCSK5	0.202019700537954	1.22387211881299	1.07811811919798	1.38933103575151	0.00179273941039663
PDCD11	0.161948466984156	1.17579964725786	1.01020807057695	1.36853471156902	0.0365187439196866
PDCL3	-0.160732236	0.851520046762079	0.738412961771659	0.981952413589827	0.0270756548006386
PDGFD	0.245793831223196	1.27863593155931	1.09645518614733	1.491086791	0.00172381651369084
PDGFRA	0.263437035861704	1.30139535112504	1.11306060238933	1.52159716757046	0.000956770527169487
PDGFRB	0.26084533699417	1.29802689316712	1.11317291572146	1.51357780232472	0.000875459302243906
PDGFRL	0.15541990575645	1.16814837036019	1.01039640609132	1.35052995730057	0.0357559175619928
PDLIM2	0.252959673289452	1.2878313417857	1.1059623911412	1.49960756185768	0.0011276884783047
PDLIM3	0.199093060615198	1.22029552206799	1.04883694009858	1.41978328970682	0.00996075008834589
PDZRN3	0.179469007840608	1.19658181883835	1.02734652366417	1.39369532693581	0.0210698111756267
PEAK1	0.206084587606186	1.22885714568346	1.05661951122241	1.42917092525603	0.00747782051995187
PEF1	0.152873804243502	1.16517792915418	1.00150282258292	1.35560237672283	0.0477700993383746
PEMT	0.172486567975062	1.18825585982545	1.02398361333065	1.37888142937851	0.0230761327999799
PFKM	0.168822951073681	1.18391051028255	1.00488342904839	1.39483253065962	0.0435703258753341
PGAP2	0.158090856779249	1.17127260793019	1.00120546435556	1.37022776136227	0.048266483104355
PGD	0.202829151604557	1.22486318446041	1.05268857427124	1.42519816146491	0.00868199108457624
PGF	0.154359818168997	1.1669106869148	1.0092456559498	1.34920625440245	0.0371393406978753
PGM2L1	0.195341576262259	1.21572617877986	1.04210929176379	1.41826788557767	0.0129700305634352
PGM3	0.227184927883566	1.25506194242985	1.07679910257407	1.46283598822692	0.00365333033929289

PHGDH	0.267751993880654	1.3070229501263	1.12936219177097	1.51263164696353	0.000328177204040783
PHYKPL	-0.157931311	0.853908433716943	0.740010598341884	0.985336716537204	0.0306026024688957
PICK1	-0.183181746	0.832616817514141	0.710412589850639	0.97584245368333	0.0237021725545438
PID1	0.265573255723956	1.30417838925507	1.12578424685341	1.51084124311914	0.00040215678536798
PIEZO2	0.16312050556282	1.17717853770378	1.02039812212653	1.35804768705619	0.0252949434437005
PIGS	0.27741212626806	1.31971014619272	1.11415724141375	1.56318588187253	0.00132135293513902
PIGU	0.172293451517101	1.18802641021861	1.02572073238556	1.37601464688577	0.0215163016970513
PIGV	0.185674406269084	1.20403017187452	1.03106917158754	1.40600523682819	0.018942679655941
PIN4	-0.200639253	0.818207544028548	0.704130137253808	0.950766839374633	0.00881957958890416
PITPNA	0.166385474636345	1.1810282704327	1.01047540138371	1.38036786808588	0.0365351670321687
PITPNM2	0.170103129216301	1.18542709718421	1.01697414436357	1.38178282164488	0.0296151421945612
PITX1	0.185885622623412	1.20428450959712	1.03895274716322	1.39592602648726	0.0136195386461203
PKIG	0.160612797852587	1.17423021631767	1.01048405493259	1.36451099270974	0.0360756577269917
PKM	0.163313205082862	1.17740540130055	1.007974224	1.37531639796422	0.0393834381034919
PLA2G2F	-0.204834271	0.814782338099522	0.701223948028425	0.946730727530732	0.00747780637607443
PLA2G6	-0.231601117	0.793262479127105	0.682653671827739	0.921792977551976	0.00250411600915618
PLAC9	0.181087920060345	1.19852054866005	1.02925743559561	1.39561926480439	0.0197428614850302
PLAGL1	0.176734158189864	1.19331381825104	1.02482104753838	1.38950880473164	0.0228662160271344
PLAU	0.157236945896741	1.17027287240717	1.00262685585115	1.36595044098385	0.046237388653801
PLD1	0.217837549525516	1.2433850627182	1.07108951457361	1.44339608702658	0.00420492287735192
PLEKHH1	-0.20150634	0.817498394721169	0.700972296189561	0.953395204068039	0.0102216840226485
PLEKHS1	-0.249109129	0.779494903161098	0.663626791851712	0.915593390011748	0.00241360413466704
PLIN5	-0.244723438	0.782921034238783	0.669174376812337	0.916002415952377	0.00224805652240117
PLK2	0.174988856252852	1.1912329417397	1.03348492669922	1.37305913693195	0.015760940442029
PLOD1	0.260636045238695	1.29775525526678	1.11956340579735	1.50430845975453	0.000542833109450974
PLXND1	0.162116077531262	1.17599674019699	1.01095936831604	1.36797617816982	0.0356199754242483

PM20D1	-0.158219044	0.85366277195684	0.731759717198999	0.995873523913667	0.0441607530984678
PMEPA1	0.21220773542697	1.23640470346142	1.06632311097297	1.43361479743852	0.00494751526662208
PMM2	0.146685047192342	1.15798919364252	1.0043071303495	1.33518814321891	0.0434742851908584
PMPCA	0.213080291513114	1.2374840067182	1.05846912156391	1.44677500333756	0.00752398572909201
PMS1	-0.162986761	0.849602436358512	0.729566289481641	0.989388230066356	0.0359722294448818
PNMA1	0.275498293145181	1.31718685655017	1.12156725171536	1.54692570812404	0.000783376691463799
PNP	0.163351342403442	1.17745030524405	1.0011400229479	1.38481050556446	0.0484123687399252
PODN	0.174164938623926	1.19025186813626	1.02003791178142	1.38886946577082	0.0269712368304702
POLA2	0.176077137729246	1.19253004416251	1.0131680129524	1.40364469470973	0.0342367948748557
POLB	-0.223031321	0.800089789195534	0.681579684426157	0.939205914439646	0.00639543289390964
POLD2	0.202571611395524	1.22454777355708	1.0521999359213	1.42512577555961	0.00885961436165804
POLDIP2	0.162336511934241	1.17625599891005	1.00350049157703	1.37875186567924	0.0451685554694639
POLE2	0.196780714498338	1.21747703636921	1.03699617606114	1.42936914166494	0.0162291167493634
POLR3A	0.164527958755338	1.17883652789293	1.01778952958389	1.36536633469084	0.0281466260651483
POLR3B	0.167840697042137	1.18274818035592	1.02288247656201	1.36759920146157	0.023492715759831
POLR3D	0.250249383091992	1.28434567084768	1.09906640739481	1.50085908469825	0.00164181505861964
POMT2	0.165419568872495	1.17988805917598	1.00972414736388	1.37872886948437	0.0373655087726455
POU5F1	-0.301671997	0.739580609452351	0.625316013014005	0.874724885488676	0.000426549760134319
PPARG	-0.151340453	0.859555010631667	0.748042196863118	0.987691362065236	0.0327892482411105
PPFIBP2	-0.209859981	0.810697751392371	0.696225248218318	0.943991683430816	0.00689046947212603
PPIB	0.191451392299655	1.21100596749416	1.02487286905311	1.43094377614016	0.0245436310997851
PPME1	0.221758633093022	1.24827005041937	1.07794691653637	1.44550542783746	0.00304888494053732
PPP1CB	0.177841100748114	1.19463547946838	1.00476929713247	1.4203797158986	0.0440260916498323
PPP1R1C	0.175333826355038	1.19164395237845	1.03480820383506	1.37224975988544	0.0148841157137113
PPP2CB	0.21499873277088	1.23986032577796	1.04863228830272	1.46596060848591	0.0118836834096185
PPP2R3A	0.280214148435174	1.32341318885752	1.13842500141529	1.53846100205516	0.000264726790854727

PPP2R3C	0.171704060560324	1.1873264045048	1.01536185306917	1.38841535810412	0.0314810164809994
PPP3CC	0.200757654671286	1.22232851032135	1.04453365028482	1.43038664837367	0.0123060635184836
PPT2	0.244000341174814	1.27634476594607	1.09756388813338	1.48424704855081	0.00152940822004778
PRDX1	0.295494363551362	1.34379051555683	1.14863162034752	1.57210799155448	0.000223623753565491
PRDX4	0.174641032474338	1.19081867464696	1.00508207046424	1.41087892975043	0.0435334527734318
PRELP	0.228450905944023	1.25665182948221	1.08172402508868	1.45986756687919	0.00281608469809777
PRICKLE3	-0.213355208	0.807869124781261	0.694172824013721	0.940187371498054	0.00583470536272247
PRKACA	0.203510989426886	1.22569862729264	1.05340517642035	1.42617214968724	0.00846025287770807
PRKAR2A	0.252643591468376	1.28742434603532	1.10078275171331	1.50571168033356	0.00156918705567626
PRKCSH	0.205531291500139	1.22817741187424	1.04074718139414	1.44936232545688	0.014987383210162
PRKDC	0.190833897432093	1.21025840835536	1.0347920980101	1.41547796684138	0.016944139388489
PRMT5	0.278730064899596	1.32145058982384	1.11893541240885	1.56061881854867	0.00102345466721236
PRMT6	0.240854467756034	1.27233585594158	1.08900541293516	1.48652936990587	0.00241298060271156
PROS1	0.248167638421076	1.28167477212094	1.11005412278316	1.4798289450722	0.000715835944746656
PRPF19	0.308201180096618	1.36097476243154	1.16270016182402	1.59306101847428	0.000124856088821698
PRPF6	0.217572566036847	1.24305562985559	1.06005913260944	1.4576425516113	0.00741068478430419
PRPF8	0.182835293194066	1.20061664206231	1.03374390484165	1.39442691216426	0.016637609017124
PRPSAP1	0.175632024717012	1.19199935164021	1.01501767038837	1.39984011683957	0.0322148443043344
PRR11	0.235575445079787	1.26563686372949	1.06505360015033	1.50399629709239	0.0074539200770516
PRRX1	0.176584322981066	1.19313503122055	1.03561722857383	1.37461135586368	0.0145080969272529
PRSS27	0.183964989049681	1.20197374011672	1.03060953948512	1.40183145660767	0.0190703953481681
PRTFDC1	0.174161601709211	1.19024789637391	1.02650873630994	1.3801052097376	0.0210854360906203
PSD4	-0.160562232	0.851664821226951	0.729343176567844	0.994501616000329	0.0423910486366037
PSMB5	0.29549700540865	1.34379406566429	1.13191710474449	1.59533103912427	0.000737116179712661
PSMB8	-0.149518284	0.86112269332166	0.747447521079479	0.992086095733671	0.0384559485053961
PSMC1	0.200851320232074	1.22244300576879	1.03617328458286	1.44219786843341	0.0172538786296605

PSMD1	0.267053026770725	1.30610970327418	1.10247728688865	1.54735392490609	0.00201432457531262
PSMD2	0.209952107977929	1.23361897803466	1.05184072111626	1.4468120052932	0.00984066905987171
PSMG1	0.219711329291874	1.24571707664846	1.06611273923788	1.4555787375385	0.00567691836944512
PTDSS2	0.1584350218452	1.17167578842089	1.00505029271934	1.36592582790788	0.0429362766571134
PTGER4	-0.163059474	0.849540660933119	0.729171699063206	0.989779684957467	0.0364612470108911
PTGES3	0.163316610225486	1.1774094105407	1.00013936953968	1.38609973994709	0.0498046598369782
PTPN14	0.187016628501394	1.2056473329916	1.0468477210204	1.38853575583361	0.00945016486606786
PTPN21	0.190117002328911	1.20939109095377	1.03484895086992	1.41337227007752	0.016815016917681
PTPRD	0.222907138867373	1.24970451937048	1.1009642833267	1.41853955608437	0.000565465370325659
PWP1	0.202104343042913	1.22397571479912	1.04249893641407	1.43704372070744	0.0135767493149828
PXDN	0.261261598040468	1.29856732367208	1.11543480320273	1.51176661268509	0.000755910042655799
PYCR1	0.247312611852583	1.28057937450237	1.07391090088266	1.52702010292758	0.00588672236086238
PYGB	0.2140630832407	1.23870079358912	1.05901064000458	1.44888030212018	0.00742914830217788
PYGL	0.182869405985811	1.20065759914636	1.02779625591862	1.40259186787896	0.0211307363573282
PYROXD2	-0.181226547	0.834246341487808	0.720555322362268	0.965875813676783	0.0153316715175432
QPRT	0.15151829610314	1.16359959095305	1.00597147781997	1.34592683581871	0.0413375551594769
QSOX2	0.200602284002965	1.22213861107656	1.04954974252921	1.42310814262582	0.00980672060912785
QTRT1	-0.182409088	0.833260393624302	0.71013785226882	0.977729720172829	0.0253486046306446
RAB11FIP5	0.158382186129143	1.17161388372703	1.00339596205254	1.36803330335711	0.0451949853561338
RAB17	-0.239361082	0.78713061250139	0.676639211975389	0.915664641024898	0.00192462689540858
RAB22A	0.186954337325667	1.20557223414074	1.02578196631372	1.41687459856024	0.0232750062649446
RAB23	0.232486349563223	1.26173322303184	1.07173622836648	1.48541281330834	0.00523906183971679
RAB4B	-0.244327815	0.783230837143765	0.662737431271515	0.925631351583642	0.0041481093981435
RAB6A	0.174696620316477	1.19088487152732	1.0208947413853	1.38918021588416	0.0262075296477521
RABEPK	0.180129583850785	1.19737251321021	1.01809925936797	1.4082133173158	0.0295012050959022
RABL2A	-0.166200037	0.846876810593745	0.732816578235056	0.978690102847793	0.0243344172379571

RAC3	0.281533074563168	1.32515982467905	1.1221452384655	1.56490309876887	0.000905830955563467
RAD23A	0.205369543828819	1.22797877310301	1.04878974670936	1.43778280815845	0.010713937448893
RAD51C	0.200545045056144	1.2220686591516	1.04637731786423	1.42725934725811	0.0113274937851906
RAD54B	-0.194038871	0.823625888405935	0.697833525269942	0.972093743690599	0.0217519920827441
RAE1	0.166155042243431	1.1807561546156	1.00204586227484	1.39133860948994	0.0472143241133391
RALGPS1	-0.150546626	0.860237619769721	0.740777205405087	0.998962653099467	0.0484306580565851
RAPGEF5	0.203964759267548	1.22625493857254	1.058880784	1.42008543025954	0.00644826797573282
RASA1	0.215041558856529	1.23991342527948	1.06626756283739	1.44183819875118	0.00521430981395939
RASD1	0.211979896544325	1.23612303448408	1.05535982080544	1.44784757412498	0.00859027476022336
RASSF7	-0.18350484	0.832347846783344	0.696379037227401	0.994864723101416	0.043743423274244
RBCK1	-0.151051013	0.859803836111714	0.746264157826814	0.990617904985838	0.0365803486099466
RBFA	0.269195171258678	1.30891057784367	1.12253646584477	1.52622828114696	0.000592811314817497
RBM34	-0.153864692	0.857388024365973	0.73520734204055	0.999873344961292	0.0498115451274636
RBM6	-0.203324058	0.816013762457125	0.711648145354087	0.935684951708993	0.00359058843067381
RBMS3	0.254048782789483	1.28923469519852	1.11008841909525	1.49729163074983	0.00087414518413278
RBP1	0.219977415543054	1.24604858893889	1.08358883048196	1.43286553194353	0.00202688041467466
RCAN1	0.222374019922947	1.2490384557776	1.05807249510808	1.47447086208581	0.00862029092854229
RCN3	0.209974963245723	1.23364717304895	1.06505209095472	1.42893043494938	0.00510166444599116
RDH12	0.152140740695299	1.16432409268358	1.00777373757535	1.34519341222868	0.0389161615503842
RDX	0.243332217225481	1.27549229425042	1.08123907983019	1.50464464616624	0.00389539603649859
RECK	0.156803874852395	1.16976617083895	1.00383148626656	1.36313008025718	0.044541494459923
RECQL	0.182913157118794	1.20071013042579	1.02124740231904	1.41170965432403	0.0267937721484149
REPS1	0.154000005117374	1.16649089274781	1.00244645123929	1.35738023829742	0.0464189807226842
REV3L	0.202759469004673	1.2247778357829	1.05166608945278	1.42638501143039	0.00911004067731561
REXO2	0.252294404316784	1.2869748724748	1.09741107497446	1.5092834035961	0.00191349703924965
RFT1	0.167854063987354	1.18276399019172	1.00843450143335	1.38723006254332	0.0390933324605861

RGMB	0.149343972046625	1.16107229694406	1.00874975299013	1.3363957460559	0.0373993984543922
RGS2	0.260012661226974	1.29694650749592	1.10270170127244	1.52540822360651	0.00168407170522098
RHBG	-0.206601614	0.813343610310353	0.69640155582793	0.949922961682946	0.0090903197410539
RHOA	0.176140296290624	1.19260536502305	1.01257488711434	1.40464431300987	0.034889852369194
RHOD	0.155942306489461	1.16875877134821	1.00902119609077	1.35378431186146	0.0375497161064221
RIIAD1	-0.262445948	0.769167938350163	0.633944974366884	0.933234494013752	0.00780433562932628
RILP	-0.199088572	0.819477307407492	0.707618052079782	0.94901911473581	0.00784274216578446
RIOK2	0.179823956560052	1.19700661940941	1.0298610768059	1.39127973585897	0.0191085352701666
RNASEH2B	0.152904895634906	1.16521415672041	1.00366168469205	1.35277061158137	0.044647355772871
RNF144A	0.185794991069233	1.20417536836624	1.03462474254994	1.4015113481688	0.0164137881026235
RNF187	0.203717332000854	1.22595156719755	1.0472187695909	1.4351893689809	0.0112825417631973
RNF20	0.177793878301512	1.19457906719021	1.01845815909533	1.40115638038251	0.0289102973078645
RNF217	0.246564884522371	1.27962220819985	1.1015931501649	1.48642263749838	0.00125593358099369
RNF40	0.249740885856996	1.2836927506434	1.08897290244531	1.51323056281207	0.00292535923726459
RNH1	0.180391648393201	1.19768634321004	1.02117447411742	1.4047086105943	0.0265860145957829
RPA1	0.165047029	1.17944858583499	1.01086379813678	1.3761487642472	0.0359699706465346
RPL17	0.193180224975135	1.21310140498946	1.04609972337779	1.40676360570638	0.0105773824444239
RPL21	0.161423194968411	1.17518219478649	1.00753433132315	1.37072568944575	0.0398263231137978
RPN1	0.184565887356639	1.20269622114924	1.01567462244242	1.42415510676862	0.0323284733074976
RPN2	0.256925720959447	1.29294908417497	1.09564204347359	1.52578786495717	0.00235696414591498
RPP21	-0.217517728	0.804513342762886	0.693174253267616	0.933735948258931	0.0042084807175486
RPTOR	0.230952056500801	1.25979883883671	1.06973360042915	1.48363397550346	0.00564307529558771
RRM1	0.171595267636501	1.18719723882001	1.01781598208251	1.38476631205777	0.0289030910862195
RRP36	0.152151505923897	1.16433662696607	1.00438388617359	1.34976257540278	0.0435933191049128
RSRP1	-0.287039195	0.750482313006628	0.638103753536093	0.882652231732909	0.000524056230642541
RTP4	-0.145218658	0.864833169882697	0.752763664734364	0.993587292757133	0.0402847117996702

RTTN	0.145739626556104	1.15689492411632	1.00229774911121	1.33533759467477	0.0464453419214337
RUNX2	0.311298898813033	1.36519721603596	1.17358911652562	1.58808855026704	5.47368437068582e-05
RUVBL1	0.159884756611034	1.17337563941447	1.00088178978241	1.37559740343627	0.0487434810579824
S100A10	0.221076185864621	1.24741846259809	1.06849544910299	1.45630271250748	0.00513153348483143
S100A16	0.174443810515918	1.19058384221364	1.0178860927778	1.39258203388148	0.0291332158215492
S100A7	0.139907831231342	1.15016778442325	1.00237750539252	1.31974822380622	0.0461733427607353
S100A8	0.159974711804446	1.17348119539464	1.01404806100607	1.35798111440459	0.0317767852078486
SACS	0.207208837372181	1.23023946493238	1.05566602935133	1.43368176960956	0.00796027481717855
SAFB2	-0.160570035	0.851658175840932	0.731126478774203	0.992060429397617	0.0391748028387495
SAMD12	-0.167218565	0.846014681905931	0.729453169646427	0.981201908201072	0.0270450348694297
SAPCD2	0.184929296454211	1.20313337132499	1.01809109076026	1.42180785426075	0.0299773848861879
SAR1A	0.158696852077906	1.17198260873089	1.00508041047982	1.36660034445596	0.0429069645947514
SART1	0.189828101111268	1.20904174686039	1.03422252361993	1.41341143928559	0.017206952823662
SATB1	-0.184261409	0.831718356406153	0.716496519251956	0.965469343947639	0.0154422099811502
SAV1	0.191963656004322	1.2116264808171	1.03520362984594	1.41811590173394	0.0168080996129718
SBF2-AS1	0.148059383946932	1.1595817548598	1.00694058076836	1.33536166074239	0.0397814861950583
SBSN	0.173188889915547	1.18909069111257	1.0398838584554	1.35970633661991	0.0113525520847748
SCAMP1-AS1	-0.166694055	0.846458541104707	0.729835035436421	0.981717822549677	0.0275302993590785
SCAMP2	0.206521998544347	1.22939477881515	1.04356402753644	1.44831700048723	0.0135132415686105
SCD	0.240050327548469	1.27131313078461	1.08994888964104	1.48285584018316	0.00223767801471585
SCRN2	-0.179413665	0.83576010315854	0.716688877850918	0.974613910747573	0.0221454020804139
SDCBP2	-0.211125345	0.809672572067715	0.695960262117398	0.941964232216701	0.00625176991079939
SEC23A	0.266588678928037	1.30550335484034	1.10320552054912	1.54489710008978	0.00191353074852629
SEC24B-AS1	-0.239081231	0.787350922662831	0.670127722773913	0.925079584608021	0.00365162462377292
SEC24D	0.184250443701158	1.20231689808748	1.01944122881405	1.41799829413255	0.0286195481335337
SEC61A1	0.180156106579971	1.19740427121826	1.01566062561535	1.41166936333981	0.0319546188673447

SEL1L3	-0.151780843	0.859176554934021	0.745495712405499	0.990192619842691	0.0360758952381472
SENP3	0.166524150470475	1.18119206186993	1.01524407201452	1.3742652880071	0.0310973366861541
SERBP1	0.218147890446463	1.24377099586599	1.05704395342435	1.46348341064342	0.00857938200313126
SERINC1	0.213756549639445	1.23832114836396	1.04081010603675	1.47331319862425	0.0159021846866052
SERPINE2	0.229382408700126	1.25782294949091	1.09008329496471	1.4513740184572	0.00168310684961326
SERPINF1	0.232031398411943	1.26115932660673	1.08168121309447	1.47041737235779	0.00305246570280323
SETBP1	0.297990904294517	1.34714953454842	1.15950991429697	1.56515424840879	9.86674557322975e-05
SETD3	0.276140120470555	1.31803253442788	1.11395018528606	1.55950399286865	0.00129441502678272
SETD7	0.183966436077584	1.20197547940752	1.02016095780781	1.41619324091907	0.0279133403487375
SF3A1	0.170042866650683	1.18535566245842	1.00803980399295	1.39386167188699	0.0397031667334879
SF3A3	0.173581304617386	1.18955739934718	1.02386690136435	1.38206128595036	0.0233190592323806
SF3B2	0.196877466278893	1.21759483513879	1.03175148186006	1.43691306348687	0.019812859680879
SF3B3	0.174226246662363	1.19032484238047	1.01617404286903	1.39432141603198	0.0308682828856618
SFI1	-0.240966374	0.785868051689519	0.665735677271149	0.927678380100907	0.00441509575108841
SFRP2	0.212651864845246	1.23695394912217	1.06110117691145	1.44195021694583	0.00656788243070785
SFRP4	0.167571111288238	1.18242937127117	1.02241319314854	1.36748941368718	0.0238994155571771
SFXN1	0.196975209921487	1.21771385310971	1.03455393148219	1.43330084873474	0.017864140737438
SGCB	0.23180463163341	1.26087336999298	1.07733649126308	1.47567790384001	0.00387627612470473
SGCD	0.205498918107824	1.22813765224863	1.05707779403617	1.42687898788571	0.00724637470994952
SGK2	-0.189011489	0.82777699612585	0.707671765099014	0.968266347632597	0.0181202544458741
SGMS2	0.181421165559806	1.19892001679569	1.0189118562448	1.41072968958369	0.0288392055091906
SH2D2A	-0.264984604	0.767217762035277	0.657119097886156	0.895763182467111	0.000800106894451165
SH2D3A	-0.221159815	0.801588563888086	0.68936443738019	0.932082061265071	0.00405367799126431
SH3BP5-AS1	-0.28303262	0.753495208127041	0.645864937518521	0.879061543194752	0.000319349422694405
SH3D19	0.167375878212318	1.1821985444812	1.00053362773039	1.39684800174461	0.0492719273338703
SH3PXD2B	0.234647648656109	1.26446315493925	1.08462288624618	1.47412256414071	0.00271979241375889

SHC3	-0.225073006	0.798457924324057	0.685714891050855	0.929737803912734	0.0037554717549835
SHMT2	0.165926277178681	1.1804860697516	1.00721225239029	1.38356871411211	0.0404920285055217
SIDT2	0.236791162463071	1.26717645613065	1.08762775261324	1.4763655737119	0.0023857112528994
SIGIRR	-0.159665162	0.852429166604725	0.736595366344906	0.98647848911146	0.032140702231715
SIGMAR1	0.185828632049686	1.20421587868767	1.04767049301933	1.3841526435514	0.00891265713284086
SIK3	0.193370261245917	1.21333196016277	1.03597005798548	1.42105887540339	0.0164740565875
SIPA1L2	0.172772749370507	1.18859596520916	1.01954450467357	1.38567797877919	0.0272969780764003
SIRT6	-0.206243098	0.81363525936521	0.695105905177181	0.952376221165247	0.0102469714180219
SLAMF7	-0.163239741	0.84938753065906	0.732351166356038	0.985127368375559	0.0309231889350274
SLC12A8	0.239099251341566	1.27010458991273	1.0818760701004	1.4910817550181	0.00348282789265919
SLC16A3	0.178170122892403	1.19502860566554	1.02603403288181	1.39185769924986	0.0220013479958312
SLC1A6	0.217825701611106	1.24337033128567	1.07903178219958	1.43273794731979	0.00259866249942975
SLC1A7	-0.188076262	0.828551517702632	0.698950015125635	0.982184137107342	0.03022747493504
SLC22A17	0.1492640201949	1.16097947077479	1.00311910721384	1.34368224258456	0.0453139768043095
SLC22A5	-0.157859869	0.853969441426109	0.7324727068963	0.995619085903861	0.0437962370243501
SLC24A3	0.204416175497587	1.22680861491382	1.05738872718596	1.4233737687295	0.00701980640870655
SLC25A15	0.234932136885249	1.26482293099661	1.08208266206339	1.47842406394747	0.00316937388496971
SLC25A21-AS1	0.166342835698969	1.18097791371583	1.01631660132719	1.37231727875275	0.0299145015180804
SLC25A43	0.16762766555819	1.182496245	1.01097119201345	1.38312286197724	0.0360442889910544
SLC27A2	-0.16820828	0.845177782647833	0.728652140134797	0.98033814070643	0.0262617940229101
SLC2A3	0.257431024577235	1.29360258111809	1.10819310991704	1.51003252312287	0.00110822031805531
SLC31A1	0.235733370740801	1.26583675605144	1.06618925044212	1.50286892529294	0.00710656114099158
SLC35F6	0.162883378600979	1.17689943002688	1.00924186888278	1.37240864762267	0.037775153226218
SLC38A11	0.143960881048909	1.1548389315536	1.01789823945965	1.310202637	0.0253890854536077
SLC39A14	0.204707999274667	1.22716667908086	1.05162876030803	1.43200539495063	0.00934634925798759
SLC44A4	-0.16606338	0.846992550010681	0.729756086869467	0.983063235349098	0.0289117091154355

SLC7A1	0.166818783805378	1.18154013170025	1.01548641567707	1.37474717659068	0.0308624773371004
SLC7A11	0.190732815390311	1.21013607914711	1.05724870979096	1.38513229336841	0.00564328403907001
SLC7A6	0.209288431259091	1.23280052546302	1.08166084327617	1.40505884541283	0.00171099676330115
SLC9A3R1	0.233744474921417	1.2633216405997	1.07567977700067	1.48369580030371	0.00438266219176501
SLC9A3R2	0.152069894111009	1.16424160722055	1.0040824709223	1.34994739898051	0.0440179708002337
SLC9B2	0.286785356374275	1.33213824780294	1.14853121804687	1.54509714962495	0.000150538776254199
SLCO3A1	0.184742619827553	1.202908795	1.03700277883081	1.39535746635256	0.0146945685391421
SLIT2	0.224773542095066	1.25203914992238	1.07624564354159	1.45654669298346	0.00359293262779888
SLITRK4	0.142780671214155	1.15347678325754	1.01825391124717	1.30665708701727	0.0248130290671951
SMAD4	0.181823448793409	1.19940241924158	1.02560050005169	1.40265743163157	0.022819015394017
SMAD6	-0.267734893	0.765110590276999	0.655123774267455	0.893562771414598	0.000721676946496932
SMARCA1	0.188572841284689	1.20752503745824	1.02836836306773	1.41789340128942	0.0213716698624244
SMC2	0.157472114097999	1.17054811573652	1.00048592570176	1.36951740754699	0.0492952305230598
SMG6	0.161483278027913	1.17525280544945	1.00547574	1.37369714892383	0.0425028286362374
SMIM3	0.205992826857078	1.22874439000458	1.05400989487792	1.43244649154133	0.00848619757974269
SMOC2	0.22174015071809	1.24824697963748	1.07239784928603	1.45293141273217	0.00420732385493856
SMYD2	0.189165130219252	1.20824045302158	1.02808899685599	1.41995974743642	0.021662821543186
SNAI3	-0.173228375	0.840945541765114	0.710457685822473	0.995399751916163	0.0440558142991643
SNCG	-0.144788872	0.865204943192354	0.749536610709564	0.998723188472176	0.047994986430981
SND1	0.291130214897014	1.33793879216957	1.12809147101209	1.58682186470762	0.000823908188576886
SND1-IT1	0.133152784119646	1.14242452932375	1.01293490331464	1.28846760135303	0.0300560760832103
SNHG25	-0.178660977	0.836389406218796	0.708940832152282	0.98674982044872	0.0341665669382453
SNHG7	0.171133611269398	1.18664928814789	1.01978653305625	1.3808149915863	0.026871697548129
SNRNP70	-0.182697174	0.833020378007257	0.706841725780471	0.981723241379316	0.0292520566913107
SNX17	0.266608104985435	1.30552871586977	1.09587474870247	1.55529199845021	0.00283565075538854
SNX19	0.172456680429921	1.18822034630551	1.01698665640531	1.3882852665588	0.0298468747762376

SNX25	0.169536740993615	1.18475587534153	1.01756520624934	1.37941674453473	0.0289395303271361
SNX29	0.248928742049133	1.28265063075765	1.10815723631866	1.48462022054595	0.000848526867913947
SNX8	0.163730866567163	1.17789726089634	1.01188371050277	1.37114763566825	0.0346540272798823
SOCS6	0.158226879450318	1.17143193839491	1.01445359548083	1.35270138762851	0.0311267119045515
SOD3	0.161107006442207	1.17481067439863	1.00700611712924	1.37057769283028	0.0404867673325628
SOGA1	0.253905020468932	1.28904936514907	1.0983141543757	1.51290799555954	0.0018849743398505
SORBS2	0.174635821777492	1.19081246966801	1.01887810098089	1.39176054186626	0.0281610917239583
SORBS3	0.270547421458765	1.31068174969831	1.11441496517511	1.5415143395192	0.0010799168143563
SOSTDC1	0.221811419028451	1.24833594326074	1.10462407552075	1.41074476083824	0.000378645657935264
SOX7	0.15464606179994	1.16724475547703	1.01009257337327	1.34884698205294	0.036075711316303
SPAG4	-0.180742603	0.834650167878446	0.714708432780029	0.974720418548537	0.0224042094514953
SPAG7	0.169563261961231	1.18478729663039	1.01784891543356	1.37910540255262	0.02864924440142
SPAG9	0.228812756965369	1.25710663251069	1.07767003260023	1.4664201821492	0.00359245847061036
SPARC	0.227725603558514	1.25574070737199	1.08178283667925	1.45767215996112	0.00276053496013457
SPARCL1	0.202309350960893	1.22422666523461	1.0483746919483	1.4295756463619	0.0105560815730633
SPATA20	-0.170035721	0.843634680325479	0.734889611197321	0.968471268342333	0.0157365620005226
SPDYA	-0.275518003	0.759178764195607	0.642750595256993	0.896696790728174	0.00118006468888789
SPNS2	0.141744676829272	1.15228240657759	1.00485569555348	1.32133872593209	0.0424264006008321
SPOCD1	-0.298458789	0.741960857911563	0.628723267506143	0.875593354221592	0.000411952999274042
SPON1	0.216766530499237	1.24205408653668	1.06967862082147	1.44220733578627	0.00446096292714313
SPON2	0.194161263593981	1.21429208827397	1.04926565610529	1.40527355209346	0.00918102856719119
SPP1	0.160372393792033	1.17394796053472	1.01022257129166	1.36420809948992	0.0363777408567982
SPRR1B	0.169752137495687	1.18501109509862	1.02994773969884	1.36341994975148	0.0176753230156802
SPRR2A	0.191198238824995	1.21069943592706	1.06510284255207	1.37619867828157	0.00344710797782864
SPRR2D	0.173809780950049	1.18982921610994	1.04660066445334	1.35265876622411	0.00790806359362714
SPRR3	0.192003479049196	1.21167473243357	1.0388491789312	1.41325197824043	0.0144698213406238

SPSB3	-0.16002629	0.852121386378501	0.732568563424181	0.99118484381806	0.03800875800737
SPTAN1	0.235171077657912	1.26512518487397	1.08107338178583	1.4805116473763	0.00337010295268984
SPTBN1	0.210251772910921	1.2339887057775	1.05666608108014	1.44106842573187	0.00789941749897552
SPTBN2	0.210089044767627	1.23378791742396	1.0442820376433	1.4576834325492	0.0135405184783709
SQLE	0.225311638991692	1.25271304959881	1.06327022298447	1.47590889946146	0.00707448726829399
SRC	-0.15028635	0.860461547802206	0.744750519721891	0.994150464671914	0.0413918818380878
SREBF1	0.197864207754957	1.2187968794196	1.05358553019231	1.409914801	0.00776045512151908
SREBF2	0.18924049419331	1.20833151425506	1.04388096645648	1.39868921386528	0.0112344993364832
SRGAP2	0.220655565784435	1.24689388367627	1.07222722726324	1.45001387543351	0.00416153573873572
SRGAP2C	0.215686800531166	1.24071372726074	1.08408787240351	1.41996842894324	0.00173262049177279
SRM	0.206997511742864	1.22997951127165	1.04950861632647	1.44148373306679	0.010562788158693
SRP68	0.227957017207384	1.2560313365376	1.06490038214461	1.48146694734699	0.00679841795872249
SRPRA	0.229231984802539	1.25763375709026	1.0725082961979	1.47471368993597	0.00477868250720037
SRPX	0.270628923370798	1.31078857712024	1.12227946639202	1.53096153441404	0.00063502215308067
SSC5D	0.183155775325425	1.20100147990623	1.03580934340083	1.39253865967377	0.0152671226277991
SSH3	-0.150058439	0.860657678701231	0.743562688911849	0.996192588672517	0.0443161168854144
SSRP1	0.206370163637008	1.22920812794324	1.04840982575516	1.44118510212685	0.011010709543242
ST3GAL5	-0.236505842	0.789381270976999	0.677249972040379	0.92007798699785	0.00248173604392902
ST6GAL2	0.149039111879688	1.16071838619917	1.01414840228191	1.32847142393495	0.0304682010761446
STAC3	-0.221324192	0.801456811918988	0.684184122509283	0.938830645492849	0.00610757165369405
STAMBPL1	-0.174717338	0.839694336605053	0.722091794907861	0.976450063411355	0.0232360602889157
STARD4	0.204168898327448	1.22650529065535	1.0666667888749	1.41029536467737	0.00415850807351756
STAT6	-0.137987729	0.8711093811188	0.75939616531321	0.999256499484957	0.04877127525721
STC1	0.19371127133981	1.21374578916435	1.04288151258359	1.41260423445862	0.0123368897571183
STIM2	-0.15954238	0.852533836396157	0.732366662399264	0.99241811447189	0.0395761886885637
STIP1	0.173888871206845	1.18992332372963	1.01508324910917	1.39487822067637	0.0319849126041969

STK32A	-0.156876653	0.854809490528493	0.735790201092128	0.993080995116556	0.0402937308030181
STK40	0.204888773152415	1.2273885388127	1.04292440034051	1.44447922084948	0.0136721177104665
STMN3	0.184904790068197	1.20310388723544	1.03839569208266	1.3939377585224	0.0138343453823046
STRAP	0.205350947090476	1.22795593691542	1.03899794968662	1.45127888217741	0.0160092835639694
STRN	0.177171621332933	1.19383596326591	1.0148024767429	1.40445489624887	0.0325803260864104
STRN3	0.165837210212825	1.18038093212135	1.00228011788132	1.39012948581771	0.046894042877094
STT3A	0.238295288471698	1.26908388334166	1.0775719295242	1.49463238492924	0.00430243788472548
STX2	0.160443588643376	1.17403154256052	1.00803447490019	1.36736401110043	0.0391267022033379
STXBP6	0.299211252604143	1.34879452973512	1.17738035577967	1.54516480125801	1.59861356608607e-05
SULF2	0.206442024228166	1.22929646273983	1.05804862310324	1.42826119736579	0.00699301673220248
SULT1C2	-0.272352648	0.761585642023973	0.633016229494643	0.9162682773554	0.00389093946994216
SUMF2	0.298803764083402	1.34824502341377	1.15804087963152	1.56968952921455	0.000117613377291534
SUN1	0.184866628295244	1.2030579755341	1.03581389198809	1.39730554271505	0.0154896205087265
SUPT16H	0.211506841733727	1.23553841882465	1.06006609901713	1.44005660194879	0.00680285550866466
SURF4	0.296397676889364	1.34500492786786	1.12909538345523	1.60220144595123	0.000900319160155921
SVEP1	0.210052304856745	1.23374258899851	1.03512811794152	1.47046607036015	0.0190026999723562
SVIL	0.307126906606178	1.35951348836763	1.14951534530576	1.60787494712556	0.000333683461999166
SYNC	0.202311016859382	1.22422870467366	1.05302472807249	1.42326754670833	0.00848399099227315
SZRD1	0.163866024664954	1.17805647400874	1.00206926049933	1.38495123107794	0.0471439998592583
TAF13	0.202051796440905	1.22391140072413	1.03582974089263	1.44614414675101	0.017620930256425
TAF1C	-0.218376981	0.803822358611722	0.690012472349606	0.936403920358037	0.00505465814181571
TAF4B	0.21911090992277	1.24496934848491	1.0768169406051	1.43937991706926	0.00307989799924772
TAGLN	0.180090355136373	1.19732554274714	1.02560939514168	1.39779185146475	0.0226004534482958
TALDO1	0.273479743509148	1.31453073116263	1.12937171745324	1.53004632262938	0.000414609249366385
TBC1D16	0.152049279251872	1.1642176067912	1.00224883181012	1.35236140262163	0.0466621659544574
TBCD	0.254078849811227	1.28927345922889	1.10485041312328	1.50448063640862	0.0012555798178076

TBX1	-0.193454743	0.824107132051931	0.711734336805622	0.954222003882804	0.0096967208606353
TBX3	-0.173818171	0.840449702303806	0.730897349541118	0.966422579786382	0.0147172752356188
TCEAL7	0.170088647008081	1.18540992970647	1.02071226708298	1.37668248610601	0.0258423611110841
TCF4	0.284181730933812	1.32867437004764	1.14572424361392	1.54083811306377	0.000170089336183242
TCF7L1	0.18077257113564	1.19814265608094	1.04265050891921	1.37682359720779	0.0108076713067205
TCIRG1	-0.227097656	0.796842962314676	0.69179016329502	0.91784870655879	0.001641734532851
TEAD1	0.196958659513301	1.21769369961516	1.03238208444727	1.43626857577282	0.0193709552052444
TEAD4	0.213457370297117	1.23795072367168	1.05412315252867	1.45383581658648	0.00925101025948796
TEKT5	-0.195456884	0.822458804305015	0.698677066198191	0.96817044311993	0.0188413823282526
TET1	0.199038255805934	1.22022864583725	1.06169084941752	1.40244021971057	0.00506308546241041
TEX10	0.180313008063772	1.19759216046479	1.02911681866796	1.39364837576275	0.0197535833510061
TEX2	0.253939735711415	1.28909411558711	1.09585620503212	1.51640665190429	0.0021791559115792
TFDP1	0.156911124712185	1.16989163482464	1.00046963849892	1.36800396990172	0.0493163531828739
TFRC	0.195181160358989	1.21553117260822	1.05211051372156	1.40433539282485	0.00806022404558302
TGFB3	0.224865498013018	1.25215428762544	1.07971076813865	1.45213922680581	0.00293532495630319
TGFBI	0.21687512144165	1.24218896968388	1.06773898741978	1.44514104531583	0.00497197367346216
THAP9-AS1	-0.176693875	0.838036289345109	0.717517820344019	0.978797741807436	0.0257150679132869
THBS1	0.221708868018245	1.24820793171265	1.05896361315098	1.47127155403804	0.00821983678949792
THBS2	0.161933943956325	1.17578257121086	1.00862972182645	1.37063644353036	0.038471695403954
THBS3	0.193545334098317	1.21354440024565	1.04812913376749	1.40506542936556	0.0096343614347624
THOP1	0.177351420177041	1.19405063289026	1.00933159225527	1.41257533683245	0.038613225278867
THRAP3	0.164858484327496	1.17922622791016	1.00383490687787	1.3852621452627	0.0447975233765078
THSD7A	0.153902519657015	1.16637718238877	1.02060462723289	1.33297037392986	0.0238599059948073
THY1	0.207990230905708	1.23120114177007	1.0621526084353	1.42715485463901	0.00577753323845457
TIA1	-0.258905738	0.771895780216872	0.661227470021687	0.901086422645253	0.00104139083770442
TIMM21	0.176670021549146	1.19323728556572	1.02830850490316	1.3846187334591	0.0199243468677951

TIMM22	0.238571780654963	1.2694348236292	1.08863074627882	1.48026755348482	0.00234070827053404
TIMP2	0.224741342407392	1.25199883530186	1.07548261848638	1.45748620819487	0.00374994988822886
TIPIN	0.209288685449132	1.23280083882867	1.05794317777633	1.43655910841177	0.00732492183345966
TKT	0.214765978240863	1.23957177625245	1.06136226203232	1.44770380806593	0.00668829798515779
TLE2	-0.145797623	0.864332606871285	0.749481609765398	0.996783437467877	0.0450429939184172
TM4SF1	0.300598589835315	1.3506670612194	1.16050922333829	1.5719836375064	0.000103295441246373
TM4SF1-AS1	0.224469226549366	1.25165819291374	1.09715663267375	1.42791665768834	0.000839716030559366
TM9SF1	0.160153833456783	1.17369141011179	1.01418647683848	1.35828228598002	0.0316356643561528
TMC4	-0.168709602	0.844754182661993	0.723707202613359	0.986047432646844	0.0325145042979487
TMC6	-0.166177644	0.846895774451892	0.720369677315534	0.99564497975157	0.0441325759165332
TMC7	-0.214192856	0.807192697666415	0.69317391283808	0.939966203428351	0.00583692794652915
TMCO3	0.21791408126054	1.24348022477577	1.06596564341979	1.45055619658416	0.0055575058794602
TMCO4	-0.161623015	0.850761868697279	0.730853977808406	0.99034250234187	0.0370543376879056
TMED3	0.204599032808688	1.22703296634991	1.043071773	1.44343844735815	0.0135562511941098
TMEM132A	0.216736258368764	1.24201648748242	1.04621057455176	1.47446890014383	0.0132844617639053
TMEM147-AS1	-0.177696564	0.837196420772546	0.716756835740679	0.977873962276303	0.024941915085567
TMEM179B	0.192337630834872	1.21207968336284	1.02611688528506	1.43174445317976	0.0236139815530594
TMEM208	0.176309890143519	1.19280764071376	1.0148304881486	1.40199775663106	0.0324739470596229
TMEM214	0.173913903996017	1.18995311120216	1.01823176068288	1.39063468803021	0.0287289318625378
TMEM229B	-0.222441442	0.800561884763582	0.687107436385014	0.932749810871058	0.0043332969446807
TMEM238	-0.182369766	0.833293159652432	0.705599456053224	0.984095840730292	0.0316447488561153
TMEM51-AS1	-0.25456689	0.775252194752526	0.657707493149995	0.913804345743621	0.00241008217524782
TMEM9	0.228646474118134	1.25689761461908	1.0636837043663	1.4852080624628	0.00725430604306022
TMPRSS11E	0.180209420353015	1.19746811105957	1.03204047836054	1.38941243785561	0.0175140952855461
TMTC1	0.252068817429697	1.28668458056381	1.11202787	1.48877312717905	0.000707705920112006
TMX2	0.285855286093738	1.33089984160077	1.11954932761304	1.58214948165748	0.00119588167625286

TNFAIP2	-0.245501609	0.782312025148627	0.671814035469527	0.910984398032728	0.0015778879462563
TNFAIP6	0.212673704217817	1.23698096371531	1.06305692948428	1.43936026581038	0.00594287437388789
TNFAIP8L3	0.258357567472401	1.29480171483743	1.1108937415091	1.50915557276295	0.000948224632595271
TNFRSF12A	0.183847407987504	1.20183241907615	1.02026652135842	1.41570965361023	0.0278023227468357
TNFRSF14	-0.298474622	0.741949110689014	0.64182777730503	0.857688779322802	5.44688330089936e-05
TNFSF9	0.147479042820795	1.15890899711172	1.01501017332491	1.32320847503127	0.0292416291111119
TNPO1	0.182091338793259	1.19972377019692	1.02498751517943	1.40424844542965	0.0233739542309121
TNS1	0.170514104636045	1.18591437870656	1.01470378558356	1.3860132716605	0.0320767689827859
TOM1	0.172609173900302	1.18840155596599	1.01357284855979	1.39338604050924	0.0335019710462937
TOP3A	0.152638011146873	1.1649032206306	1.01173369724482	1.34126155640655	0.0338251680915051
TOR1AIP1	0.167406969659794	1.18223530131656	1.0073690444463	1.38745608214247	0.040377542492372
TOR1AIP2	0.148676639853233	1.16029773449543	1.005453423	1.33898876047968	0.0419131067430781
TOR1B	0.162803249846142	1.17680513031909	1.01363037601478	1.36624784291699	0.0325384545132119
TOX3	-0.190122772	0.826857613126411	0.713537597158539	0.958174474768702	0.0114685064835006
TP53INP2	0.160188592865843	1.17373220764067	1.00167056480575	1.37534968447456	0.0476359324873308
TPD52L1	0.239134095867965	1.2701488468767	1.08412532133806	1.48809188519925	0.00307974228467002
TPGS2	0.200640390201811	1.22218518302083	1.05127054027736	1.42088697853319	0.00904102893451255
TPM1	0.245848515673249	1.27870585497389	1.08987381722311	1.50025501824657	0.00256461850839391
TPPP3	0.244500409726347	1.27698318543788	1.11158530671745	1.4669913735245	0.00055091319589692
TRAF5	-0.195173535	0.822691879524024	0.70847796458544	0.95531824907329	0.0104856964503073
TRAM2	0.218492627254892	1.24419984342504	1.07158980687944	1.44461363895099	0.00413916031654126
TRANK1	-0.160260754	0.851921618246629	0.736547007884251	0.985368803168108	0.0308904607479893
TRAPPC2	-0.178458762	0.836558554247956	0.719225817942643	0.973032665439218	0.0206392686329451
TRBC1	-0.164907794	0.847971888785818	0.724530828866113	0.992444069352172	0.039931700140807
TRBC2	-0.185885041	0.830369047679692	0.712930447262406	0.967152908102257	0.0168828328779407
TRIB3	0.233205726869163	1.26264121183275	1.08437634230617	1.47021174072087	0.00267239473487558

TRIM16L	0.294158103132557	1.34199606067626	1.16718652102624	1.54298682723573	3.61087909513399e-05
TRIM27	-0.175557596	0.838989073173868	0.722322308315409	0.974499412245456	0.0215558285787274
TRIM31	-0.208148155	0.812086713444358	0.693262361303922	0.951277419579604	0.00991436015835646
TRIM38	-0.254282135	0.775472983218826	0.676340605966594	0.889135359310376	0.00026865595200013
TRIM52	-0.159324304	0.852719773321891	0.735777272472332	0.988248807102804	0.0342570023443968
TRIM65	0.232051165567976	1.26118425638632	1.06884758731696	1.48813146741476	0.00598495378827792
TRIM73	-0.18868324	0.828048757864002	0.694268719130479	0.987607141884285	0.0358462397492087
TRIP11	0.168804605631639	1.18388879112013	1.01415823931466	1.38202562026911	0.0325142622598766
TRPS1	0.187117732281892	1.20576923465717	1.04502537083362	1.39123842140403	0.0103691085798039
TSC22D1	0.178590574185235	1.19553116263073	1.02184833627652	1.39873473399129	0.0257595891299874
TSEN2	-0.162049947	0.85039872864362	0.73269428904298	0.98701192092445	0.0330104039953261
TSGA10	-0.185814906	0.830427287840575	0.708700161463735	0.97306240055872	0.021583519105581
TSHZ1	0.182685931808157	1.20043732968821	1.03589908546394	1.39111019859967	0.0151450949969684
TSHZ3	0.169160995216733	1.18431079194907	1.01930881091334	1.37602268999349	0.0271193287845474
TSPAN7	0.21828539073051	1.24394202648932	1.08560626371631	1.42537107327406	0.00167552853649289
TSPAN8	-0.28689978	0.75058694923351	0.631892210058932	0.891577328207805	0.00108821503826672
TSPAN9	0.191297255383868	1.21081932115425	1.04118308619627	1.40809378092803	0.0129911578699291
TSR1	0.176715689861681	1.19329177994333	1.02726650026627	1.38614981770671	0.0207829332913995
TSTD3	0.18092924119693	1.19833038386958	1.04307051539371	1.37670050846274	0.0106008043002441
TTC21A	-0.181878015	0.833703033242062	0.714368309662617	0.97297253844488	0.0210215549337985
TTC3	0.147554372097514	1.15899630017645	1.00135274024602	1.3414577798955	0.0479189703993241
TTC4	0.176814131915776	1.19340925581947	1.03184803212366	1.38026686831428	0.0172005499308201
TTC5	0.189303129813283	1.20840720121892	1.03403692599029	1.41218164192663	0.0172687816942739
TTC6	-0.176027266	0.838595117745288	0.721414146421808	0.974810065749738	0.0218950798560746
TTC7B	0.202734786577943	1.22474760566679	1.05988998786061	1.41524754	0.0059864661965363
TTLL5	0.187825980606554	1.20662352118533	1.04579779099317	1.39218148519421	0.0100668722307271

TTLL7	0.233652693185445	1.26320569606731	1.08751671533953	1.46727733750624	0.00222831948617039
TUBA1A	0.235908388354283	1.26605831916771	1.08843467854282	1.47266868571268	0.00222324191513545
TUBA1B	0.25918353005324	1.29587161439116	1.08868753147941	1.5424841310553	0.00354602016797661
TUBB2A	0.235094357237889	1.26502812766159	1.08885993278745	1.46969882497034	0.00212204027639934
TUBG1	0.158701345406925	1.17198787484619	1.00272318019695	1.36982529766261	0.0461372876411118
TUBGCP5	0.283506686170886	1.32777775803317	1.13188185833975	1.55757755258447	0.000499452024488706
TUT1	0.196054051493152	1.21659266220712	1.02377412592387	1.44572681439918	0.0259581491009601
TVP23C	0.138831610193157	1.14893061550923	1.00373913606365	1.31512413118732	0.043998134997726
TXNRD1	0.218789320211525	1.24456904352206	1.06349124271904	1.45647847567902	0.00638551863518793
TYK2	-0.184667436	0.831380724911398	0.710146697374509	0.973311447211713	0.021656100497128
U2AF1L4	-0.188294606	0.828370628453948	0.71627874638242	0.958003991533806	0.01113837335958
UBA7	-0.179523308	0.835668473327101	0.728083017184936	0.959151334160931	0.0106771278937097
UBC	0.164964536442485	1.17935129397732	1.00742977475915	1.38061183960789	0.0401654305064923
UBE2E2	0.161361537958173	1.1751097387996	1.0128672790952	1.36334041657977	0.0332855807695421
UBE2G1	0.18065472328627	1.19800146586533	1.02112971903505	1.40550949155775	0.0266565730852385
UBE3C	0.183325644922731	1.20120551087283	1.02368372713015	1.4095121775515	0.0246496773369422
UBE4B	0.182231373656835	1.19989178511511	1.02993489517886	1.39789447151094	0.01936325840411
UBL7	0.188998243625237	1.20803883071212	1.03370939170547	1.41176797678174	0.0174579650168472
UBTD1	0.200012919116026	1.22141853770605	1.0479150444467	1.42364903735081	0.0105066998728639
UBXN8	0.190026748881879	1.20928194416452	1.0468369603352	1.39693464779278	0.00982632947694619
UCHL1	0.23621298847396	1.26644401942243	1.09988477100519	1.45822589475901	0.00102602507703602
UCHL5	0.241570843006243	1.27324765241395	1.07826382718929	1.50349065182266	0.0043928933783417
UGGT1	0.189061259619127	1.20811495887832	1.0300140600387	1.4170114860478	0.0201586952492741
UGT1A7	0.163893521436475	1.1780888672038	1.01556742250275	1.36661864911856	0.0304704535198015
UGT2B7	-0.184632722	0.831409586113227	0.710347211970957	0.973104262580297	0.0214750436254825
ULK2	0.157657152342238	1.17076473194518	1.01712072437572	1.34761786356084	0.028058316840492

UNC93B1	-0.253528645	0.776057514576008	0.659856208571851	0.9127219811016	0.00218795989341297
URB1	0.158575915608281	1.17184088186188	1.00633633668559	1.36456471096487	0.0412238714799567
USF1	-0.23604802	0.789742749695627	0.672624807289805	0.927253357201986	0.0039493951505275
USP13	0.23840119226936	1.26921829126149	1.09197080221314	1.47523639607197	0.00189370911316765
USP2	0.146649666934877	1.15794822441147	1.00345658298693	1.33622531672118	0.044729156517984
USP32	0.18041863821458	1.19771866898675	1.02673352455694	1.39717850418727	0.0216956020482104
USP5	0.235635872532447	1.26571334525192	1.06855306410749	1.4992519568384	0.00638286933047268
UTP18	0.218833208333948	1.24462366651924	1.05629608492573	1.46652827116057	0.00894249808805299
UTP6	0.216021051640381	1.24112850651664	1.05363120123623	1.46199160378021	0.00973343664986905
VANGL1	0.177197911784481	1.19386735016505	1.02465686918232	1.39102102631442	0.0230672408086972
VAPB	0.187496158310718	1.20622561546806	1.03323534221415	1.40817892687777	0.0176003252747783
VAT1	0.169888752720199	1.18517299671429	1.0186831733758	1.37887330315463	0.0278330061579359
VCAN	0.19750116222135	1.21835448096641	1.0461813204545	1.41886268877946	0.0110613823497047
VCL	0.23393409921947	1.26356121979332	1.07010765926956	1.49198722421572	0.00579507534666524
VDAC2	0.2522821080523	1.28695904758867	1.10337910280644	1.50108297860422	0.0013145152288468
VEGFA	-0.169628749	0.843978085873695	0.729881713335843	0.975910200818079	0.0220783043101995
VEGFB	0.17697186195543	1.19359750715476	1.02557131442558	1.38915255238396	0.0222458050728564
VIM	0.2192445924027	1.24513579019978	1.07022779271704	1.44862911109835	0.00452895146656766
VIPAS39	0.169493306482531	1.18470441716687	1.0094417622204	1.3903967604505	0.0379854289728525
VKORC1	0.237562325870702	1.26815403313199	1.08946007107671	1.47615749713483	0.00217186045993746
VPS18	0.222915017173622	1.24971436496419	1.06345898283034	1.46859072067004	0.00678594607913576
VPS37C	0.224952619150489	1.25226338148339	1.06812348871718	1.46814819931315	0.00556974508118644
VSIG10L	0.215214738479757	1.24012817161361	1.07298792148021	1.4333040020694	0.00357141519383083
VTCN1	0.151897800206351	1.16404126557568	1.00530776630832	1.3478380585269	0.0422817448875194
VWF	0.228109480533869	1.25622284985234	1.07756616217514	1.4645001893022	0.00356341804527217
WASIR2	-0.244011413	0.783478692647004	0.669542334910848	0.916803657999599	0.00233995958654969

WBP4	0.15641951529763	1.16931664642939	1.00386294396786	1.3620399356634	0.0444846682463499
WDR81	0.15279556190941	1.16508676647981	1.00186922644425	1.35489456866944	0.0472318493804968
WDR83	-0.155720666	0.855798210199014	0.732552251738679	0.999779298802975	0.0496756870588428
WNK1	0.186076484456104	1.20451438348208	1.02621885659994	1.41378702085263	0.02280928467484
WNT5B	0.206500720168164	1.22936861956889	1.05995537547303	1.42585927460033	0.00634040732493182
WWC3	0.184236167354799	1.20229973351754	1.02810083904092	1.40601446309958	0.021055570518055
XDH	-0.164066395	0.848685671478398	0.724578923406353	0.994049572387029	0.0419563783161107
XG	0.152231891316768	1.16443022638522	1.01038126455339	1.34196644344833	0.0354999020800128
XPO5	0.190380960222689	1.20971036141402	1.03925331784575	1.40812555840178	0.014016836320068
XPO6	0.246620910761913	1.27969390262858	1.09467533721557	1.49598372115537	0.00196628782881004
XPOT	0.262142210203591	1.29971136150482	1.11058244679625	1.52104837249839	0.00108623647498379
YDJC	-0.19893263	0.819605108008776	0.686577028434647	0.978408110457222	0.0276983153328982
YJEFN3	-0.163951411	0.848783262654237	0.729663182941037	0.987350114141895	0.0335895991230079
YKT6	0.231163925333065	1.26006577922276	1.07420197195313	1.47808867366104	0.00452474939331385
YME1L1	0.172599295700962	1.18838981675651	1.0135688922022	1.39336395131672	0.0335054552619551
YRDC	0.158189924836878	1.17138864938032	1.00733882606522	1.36215475110479	0.0398838208280882
YWHAQ	0.207411121996504	1.2304883486322	1.0458586580377	1.44771147084104	0.0124007766613819
ZC3H12A	-0.160641619	0.851597212623214	0.732808969309364	0.989641015490173	0.0360983570143029
ZC3H14	0.155080055018268	1.16775144172628	1.00214069516622	1.36073052040624	0.0468816414805665
ZC3H8	-0.149710646	0.860957061809462	0.742588409774346	0.998193686466003	0.0472644940264174
ZCCHC24	0.173989305611713	1.19004283897211	1.02142086182039	1.38650189312251	0.0256257255297139
ZCWPW1	-0.163709401	0.848988701095477	0.735878545986276	0.979484751280177	0.0248253670104477
ZDHHC1	0.169091949897092	1.18422902365478	1.02809860283775	1.36406992149923	0.0190718871445954
ZDHHC21	-0.192792699	0.824652907707993	0.712432841162854	0.954549508247326	0.0097880449320472
ZDHHC23	-0.175176168	0.839309148142422	0.715582782837823	0.984428165476431	0.0313311857417447
ZDHHC5	0.200530828484339	1.22205128564826	1.04215911819258	1.43299551736839	0.0135779100972046

ZFP14	-0.180487726	0.834862927697425	0.714041199035023	0.976128700956551	0.0236429106206565
ZFP64	0.182562574584168	1.2002892562048	1.0204974312225	1.4117569084273	0.027451735916612
ZFPM1	-0.174689563	0.839717659918743	0.721577463645629	0.977200347717207	0.0239399895300492
ZFY-AS1	-0.180150983	0.835144109299173	0.715489567514022	0.974809018837916	0.0224103650242658
ZGLP1	-0.179192789	0.835944722550338	0.710952627363292	0.982911592508507	0.0301167552878382
ZKSCAN7	-0.162803553	0.84975810418759	0.731084962638513	0.987694826913743	0.033897225472872
ZMAT3	0.175296228060063	1.19159914943988	1.0330868284522	1.37443290712864	0.0160881471933628
ZNF100	-0.184832385	0.831243601195318	0.716683529475976	0.964115814177257	0.0145668443230095
ZNF124	-0.179095976	0.836025657167448	0.716793273748056	0.975091319966727	0.0225330573657994
ZNF165	-0.255050281	0.774877535471749	0.66396252413081	0.904320911432159	0.00121232826188096
ZNF182	-0.241263816	0.785634336349904	0.677835620668392	0.910576682062432	0.0013553006441968
ZNF224	-0.319328285	0.726636966115471	0.615141739591701	0.858340844950558	0.000171746702246637
ZNF226	-0.16156732	0.850809253039595	0.725515188486971	0.997741186600663	0.0468353711422741
ZNF251	-0.154993419	0.85642081335622	0.741496666750613	0.989156988073707	0.0350086138221803
ZNF266	-0.198973406	0.819571688660249	0.70497247275692	0.952799972779957	0.00962226954831999
ZNF286A	0.143758815365218	1.1546056018101	1.00040032744917	1.332580627	0.0493637881354437
ZNF302	-0.193375784	0.824172204963511	0.703969556500829	0.964899429473576	0.0162076369217425
ZNF346	0.156105376892959	1.16894937685328	1.01007548998359	1.35281239788114	0.036218073434847
ZNF383	-0.153658756	0.857564609606138	0.738627216539259	0.99565388761956	0.0436807462994005
ZNF407	0.170838879056783	1.18629959591291	1.01716840772681	1.38355332369023	0.0294901636853951
ZNF432	-0.308381308	0.734635142613547	0.610171599969614	0.884486909567248	0.00113009206344109
ZNF436-AS1	-0.189087887	0.827713758158106	0.712709744237391	0.961275008492122	0.0132335083752195
ZNF44	-0.244677154	0.782957272345686	0.6676020806963	0.918244726978117	0.00262290577739686
ZNF462	0.1490483828782	1.16072914726749	1.00328658003288	1.34287867507622	0.0450612353476355
ZNF519	-0.167300428	0.845945427821689	0.716580094934771	0.998665287957185	0.0481845930865011
ZNF532	0.208530047956061	1.23186594455861	1.04948094810161	1.44594688270259	0.0107503645666889

ZNF552	-0.159443713	0.852617957588938	0.729458548441619	0.996571200866959	0.0451649994034015
ZNF577	-0.229740278	0.794739987391957	0.679598575739497	0.929389304373523	0.00401511271034957
ZNF584	0.135824288947093	1.14548060230811	1.00215675193529	1.30930197070496	0.0464200383630865
ZNF736	-0.17828833	0.836701142402981	0.719756457560605	0.972646781205857	0.0202858535405535
ZNF770	0.166961975102561	1.18170933007796	1.00805247632221	1.38528199036629	0.0395072583116975
ZNF784	-0.178315406	0.836678488222588	0.716480132782814	0.977041596304296	0.0242284874452867
ZNF789	-0.181594926	0.833939078933739	0.713208402814934	0.975106833609914	0.0228523559450031
ZNF814	-0.23414627	0.791246071502088	0.672474822996381	0.930994476310463	0.00477838213321924
ZNF823	-0.191990363	0.825314821754642	0.710516182588887	0.958661564225082	0.0119903941427012
ZNF83	-0.236592979	0.789312489555393	0.67860165884498	0.918085297976636	0.00215243840217651
ZNF860	0.198347441905403	1.21938598602095	1.05705099487473	1.40665132535114	0.00650578076918219
ZNF862	-0.176028733	0.83859388793477	0.718824387939533	0.978319212147695	0.0251732790970357
ZSCAN16	-0.281892285	0.754354935429902	0.64730257913591	0.879111851164076	0.000306263985427674
ZSCAN26	-0.160559069	0.851667514624892	0.731694335551386	0.991312246418779	0.0382094123924807
ZW10	0.233193887302172	1.26262626279604	1.07297739436078	1.48579558887355	0.00498203778417372

Table S5. The result of CUT&Tag assay.

ENSG00000237330
ENSG00000162585
ENSG00000287586
ENSG00000237728
ENSG00000225126
ENSG00000227634
ENSG00000171621
ENSG00000251503

ENSG00000225158
ENSG00000120948
ENSG00000235829
ENSG00000171729
ENSG00000215720
ENSG00000278480
ENSG00000233485
ENSG00000142634
ENSG00000271742
ENSG00000116786
ENSG00000224621
ENSG00000142623
ENSG00000179051
ENSG00000074964
ENSG00000236936
ENSG00000233431
ENSG00000076864
ENSG00000070831
ENSG00000265422
ENSG00000227868
ENSG00000169641
ENSG00000230023
ENSG00000232298
ENSG00000176092
ENSG00000228943
ENSG00000233399

ENSG00000231251
ENSG00000215899
ENSG00000284543
ENSG00000184007
ENSG00000189433
ENSG00000116560
ENSG00000054116
ENSG00000196182
ENSG00000232273
ENSG00000230955
ENSG00000284623
ENSG00000275350
ENSG00000287400
ENSG00000127129
ENSG00000127124
ENSG00000283973
ENSG00000274975
ENSG00000188396
ENSG00000132128
ENSG00000142961
ENSG00000227947
ENSG00000284645
ENSG00000275816
ENSG00000123091
ENSG00000227742
ENSG00000233079

ENSG00000184292
ENSG00000185839
ENSG00000231740
ENSG00000232453
ENSG00000241042
ENSG00000235215
ENSG00000226476
ENSG00000237928
ENSG00000287224
ENSG00000162604
ENSG00000132849
ENSG00000162437
ENSG00000234784
ENSG00000233877
ENSG00000288804
ENSG00000223152
ENSG00000118473
ENSG00000152763
ENSG00000289394
ENSG00000116761
ENSG00000272855
ENSG00000276081
ENSG00000162614
ENSG00000162613
ENSG00000285409
ENSG00000235089

ENSG00000215875
ENSG00000230817
ENSG00000233008
ENSG00000171517
ENSG00000055732
ENSG00000097096
ENSG00000122417
ENSG00000016602
ENSG00000267272
ENSG00000284240
ENSG00000171492
ENSG00000272672
ENSG00000162664
ENSG00000122482
ENSG00000239794
ENSG00000224093
ENSG00000178596
ENSG00000232857
ENSG00000231987
ENSG00000228502
ENSG00000117569
ENSG00000232542
ENSG00000099260
ENSG00000223656
ENSG00000156875
ENSG00000031698

ENSG00000198758
ENSG00000224927
ENSG00000121931
ENSG00000173947
ENSG00000081026
ENSG00000233730
ENSG00000173218
ENSG00000118729
ENSG00000203864
ENSG00000228453
ENSG00000228217
ENSG00000092607
ENSG00000134240
ENSG00000286581
ENSG00000186207
ENSG00000163207
ENSG00000160712
ENSG00000228264
ENSG00000287064
ENSG00000169231
ENSG00000273002
ENSG00000160789
ENSG00000196189
ENSG00000290592
ENSG00000226520
ENSG00000289721

ENSG00000233473
ENSG00000290105
ENSG00000279430
ENSG00000018625
ENSG00000143256
ENSG00000118217
ENSG00000171722
ENSG00000185860
ENSG00000224702
ENSG00000162763
ENSG00000198842
ENSG00000143190
ENSG00000287218
ENSG00000143164
ENSG00000000457
ENSG00000235378
ENSG00000135845
ENSG00000224000
ENSG00000232751
ENSG00000203739
ENSG00000224977
ENSG00000235750
ENSG00000236720
ENSG00000075391
ENSG00000213057
ENSG00000266417

ENSG00000116199
ENSG00000162779
ENSG00000289589
ENSG00000237292
ENSG00000289573
ENSG00000232096
ENSG00000287808
ENSG00000135862
ENSG00000224468
ENSG00000058085
ENSG00000289099
ENSG00000286966
ENSG00000198860
ENSG00000290066
ENSG00000135842
ENSG00000229739
ENSG00000228238
ENSG00000252553
ENSG00000230987
ENSG00000283795
ENSG00000162630
ENSG00000288950
ENSG00000287989
ENSG00000223881
ENSG00000236278
ENSG00000207759

ENSG00000118193
ENSG00000116852
ENSG00000081277
ENSG00000264802
ENSG00000227109
ENSG00000143862
ENSG00000226862
ENSG00000117139
ENSG00000188770
ENSG00000058668
ENSG00000219133
ENSG00000225063
ENSG00000263961
ENSG00000237605
ENSG00000182795
ENSG00000196352
ENSG00000283044
ENSG00000231648
ENSG00000230937
ENSG00000196878
ENSG00000117595
ENSG00000143469
ENSG00000279333
ENSG00000224535
ENSG00000286213
ENSG00000288007

ENSG00000260805
ENSG00000162772
ENSG00000162771
ENSG00000228646
ENSG00000225233
ENSG00000213036
ENSG00000185221
ENSG00000196482
ENSG00000162814
ENSG00000116141
ENSG00000232436
ENSG00000143507
ENSG00000200033
ENSG00000226601
ENSG00000178395
ENSG00000162909
ENSG00000229991
ENSG00000229400
ENSG00000234277
ENSG00000271475
ENSG00000143641
ENSG00000232964
ENSG00000287423
ENSG00000135750
ENSG00000265744
ENSG00000286109

ENSG00000236101
ENSG00000287633
ENSG00000227630
ENSG00000258082
ENSG00000280587
ENSG00000168243
ENSG00000235674
ENSG00000206803
ENSG00000229291
ENSG00000116962
ENSG00000235371
ENSG00000288099
ENSG00000230019
ENSG00000223963
ENSG00000201861
ENSG00000225140
ENSG00000107929
ENSG00000287043
ENSG00000205696
ENSG00000231743
ENSG00000233321
ENSG00000228353
ENSG00000067082
ENSG00000288755
ENSG00000287175
ENSG00000230573

ENSG00000226005
ENSG00000224382
ENSG00000230096
ENSG00000225269
ENSG00000231298
ENSG00000215267
ENSG00000287235
ENSG00000226990
ENSG00000225778
ENSG00000107611
ENSG00000107614
ENSG00000236582
ENSG00000136738
ENSG00000260314
ENSG00000207264
ENSG00000206842
ENSG00000179133
ENSG00000287124
ENSG00000286442
ENSG00000151023
ENSG00000099246
ENSG00000230500
ENSG00000165322
ENSG00000229327
ENSG00000099250
ENSG00000287278

ENSG00000226412
ENSG00000271335
ENSG00000169813
ENSG00000243660
ENSG00000165512
ENSG00000266412
ENSG00000204175
ENSG00000265763
ENSG00000286401
ENSG00000225303
ENSG00000238523
ENSG00000227972
ENSG00000233805
ENSG00000252076
ENSG00000170312
ENSG00000289989
ENSG00000183346
ENSG00000233643
ENSG00000150347
ENSG00000235816
ENSG00000286373
ENSG00000228566
ENSG00000198739
ENSG00000225922
ENSG00000239942
ENSG00000138347

ENSG00000222371
ENSG00000108187
ENSG00000156510
ENSG00000232734
ENSG00000099282
ENSG00000235645
ENSG00000042286
ENSG00000148734
ENSG00000180644
ENSG00000237512
ENSG00000289362
ENSG00000214688
ENSG00000107738
ENSG00000197746
ENSG00000230526
ENSG00000289592
ENSG00000289506
ENSG00000168209
ENSG00000148719
ENSG00000122861
ENSG00000222047
ENSG00000156650
ENSG00000165637
ENSG00000226051
ENSG00000227271
ENSG00000225497

ENSG00000199592
ENSG00000288090
ENSG00000229569
ENSG00000108175
ENSG00000108179
ENSG00000237523
ENSG00000122378
ENSG00000108219
ENSG00000226659
ENSG00000287358
ENSG00000165678
ENSG00000229404
ENSG00000225928
ENSG00000182771
ENSG00000173267
ENSG00000272734
ENSG00000122376
ENSG00000289952
ENSG00000107796
ENSG00000232110
ENSG00000119917
ENSG00000152778
ENSG00000226159
ENSG00000222451
ENSG00000180628
ENSG00000107854

ENSG00000214549
ENSG00000221042
ENSG00000105988
ENSG00000138119
ENSG00000186188
ENSG00000234026
ENSG00000107438
ENSG00000196233
ENSG00000234855
ENSG00000155265
ENSG00000284415
ENSG00000289758
ENSG00000107554
ENSG00000235470
ENSG00000280693
ENSG00000107957
ENSG00000107960
ENSG00000228261
ENSG00000207068
ENSG00000229775
ENSG00000287047
ENSG00000200079
ENSG00000234544
ENSG00000270294
ENSG00000119953
ENSG00000237642

ENSG00000199364
ENSG00000150593
ENSG00000284482
ENSG00000278601
ENSG00000197142
ENSG00000225292
ENSG00000227560
ENSG00000165806
ENSG00000043591
ENSG00000228169
ENSG00000151553
ENSG00000165868
ENSG00000165650
ENSG00000258114
ENSG00000107560
ENSG00000165669
ENSG00000213574
ENSG00000107581
ENSG00000242853
ENSG00000265719
ENSG00000151923
ENSG00000151929
ENSG00000271670
ENSG00000222979
ENSG00000285973
ENSG00000202245

ENSG00000188620
ENSG00000231138
ENSG00000182022
ENSG00000258539
ENSG00000264572
ENSG00000175029
ENSG00000233135
ENSG00000237675
ENSG00000107949
ENSG00000222740
ENSG00000213170
ENSG00000287326
ENSG00000232259
ENSG00000108010
ENSG00000286295
ENSG00000188385
ENSG00000171813
ENSG00000180066
ENSG00000177156
ENSG00000198788
ENSG00000215182
ENSG00000184545
ENSG00000214026
ENSG00000226416
ENSG00000240801
ENSG00000205339

ENSG00000166483
ENSG00000289518
ENSG00000133800
ENSG00000072952
ENSG00000255558
ENSG00000222162
ENSG00000256206
ENSG00000284094
ENSG00000110693
ENSG00000070081
ENSG00000197149
ENSG00000270607
ENSG00000254542
ENSG00000254560
ENSG00000255450
ENSG00000241255
ENSG00000110422
ENSG00000255202
ENSG00000110429
ENSG00000254708
ENSG00000166016
ENSG00000220204
ENSG00000255427
ENSG00000135373
ENSG00000271369
ENSG00000270491

ENSG00000251862
ENSG00000289526
ENSG00000026508
ENSG00000255521
ENSG00000215380
ENSG00000149090
ENSG00000254669
ENSG00000179431
ENSG00000166326
ENSG00000254919
ENSG00000263389
ENSG00000280321
ENSG00000110442
ENSG00000251838
ENSG00000254516
ENSG00000279004
ENSG00000255477
ENSG00000283483
ENSG00000287984
ENSG00000254746
ENSG00000255426
ENSG00000255498
ENSG00000289990
ENSG00000285658
ENSG00000109920
ENSG00000254879

ENSG00000254952
ENSG00000167995
ENSG00000167996
ENSG00000279491
ENSG00000244176
ENSG00000168003
ENSG00000133321
ENSG00000133328
ENSG00000213465
ENSG00000290057
ENSG00000173156
ENSG00000261070
ENSG00000172927
ENSG00000260877
ENSG00000213275
ENSG00000259854
ENSG00000255980
ENSG00000255774
ENSG00000285094
ENSG00000149716
ENSG00000162344
ENSG00000186895
ENSG00000260679
ENSG00000254605
ENSG00000248844
ENSG00000255143

ENSG00000254902
ENSG00000168040
ENSG00000246889
ENSG00000227726
ENSG00000286708
ENSG00000162105
ENSG00000158483
ENSG00000263742
ENSG00000227467
ENSG00000265064
ENSG00000289625
ENSG00000175581
ENSG00000246211
ENSG00000264402
ENSG00000077514
ENSG00000166439
ENSG00000199090
ENSG00000137486
ENSG00000149273
ENSG00000158555
ENSG00000149257
ENSG00000254630
ENSG00000247867
ENSG00000254915
ENSG00000255362
ENSG00000254755

ENSG00000254632
ENSG00000078124
ENSG00000149269
ENSG00000074201
ENSG00000254563
ENSG00000255345
ENSG00000254885
ENSG00000266570
ENSG00000149256
ENSG00000255209
ENSG00000279295
ENSG00000254471
ENSG00000254434
ENSG00000200146
ENSG00000255014
ENSG00000254437
ENSG00000255316
ENSG00000182103
ENSG00000255382
ENSG00000254441
ENSG00000242279
ENSG00000254698
ENSG00000137494
ENSG00000213305
ENSG00000179071
ENSG00000137501

ENSG00000073921
ENSG00000200877
ENSG00000254699
ENSG00000278989
ENSG00000151376
ENSG00000254731
ENSG00000255250
ENSG00000150687
ENSG00000280339
ENSG00000254794
ENSG00000123892
ENSG00000266581
ENSG00000288018
ENSG00000249489
ENSG00000255429
ENSG00000110172
ENSG00000165323
ENSG00000242807
ENSG00000110218
ENSG00000288789
ENSG00000255929
ENSG00000087053
ENSG00000285921
ENSG00000266192
ENSG00000200411
ENSG00000248027

ENSG00000110318
ENSG00000270868
ENSG00000225678
ENSG00000256254
ENSG00000149968
ENSG00000170967
ENSG00000256422
ENSG00000196954
ENSG00000254811
ENSG00000287802
ENSG00000289232
ENSG00000137760
ENSG00000110723
ENSG00000203334
ENSG00000201243
ENSG00000287245
ENSG00000289465
ENSG00000149289
ENSG00000137710
ENSG00000290078
ENSG00000204381
ENSG00000235286
ENSG00000137713
ENSG00000197580
ENSG00000150787
ENSG00000213233

ENSG00000227487
ENSG00000166682
ENSG00000180425
ENSG00000255663
ENSG00000252870
ENSG00000236437
ENSG00000160584
ENSG00000137731
ENSG00000160588
ENSG00000289553
ENSG00000181264
ENSG00000285509
ENSG00000109927
ENSG00000207994
ENSG00000109943
ENSG00000109944
ENSG00000196248
ENSG00000229386
ENSG00000154133
ENSG00000287666
ENSG00000278215
ENSG00000273415
ENSG00000272575
ENSG00000276176
ENSG00000199260
ENSG00000043039

ENSG00000281386
ENSG00000149418
ENSG00000255155
ENSG00000224700
ENSG00000080854
ENSG00000254573
ENSG00000266043
ENSG00000290204
ENSG00000256969
ENSG00000256417
ENSG00000255983
ENSG00000255775
ENSG00000010278
ENSG00000256103
ENSG00000008323
ENSG00000235868
ENSG00000166532
ENSG00000255734
ENSG00000139112
ENSG00000245648
ENSG00000060138
ENSG00000134551
ENSG00000111266
ENSG00000256658
ENSG00000207983
ENSG00000213782

ENSG00000234498
ENSG00000289766
ENSG00000134531
ENSG00000180861
ENSG00000121316
ENSG00000084463
ENSG00000111348
ENSG00000023734
ENSG00000008394
ENSG00000257046
ENSG00000139163
ENSG00000133703
ENSG00000278743
ENSG00000286752
ENSG00000278095
ENSG00000029153
ENSG00000087494
ENSG00000247934
ENSG00000064763
ENSG00000257258
ENSG00000257456
ENSG00000242986
ENSG00000177340
ENSG00000225349
ENSG00000253063
ENSG00000212475

ENSG00000175548
ENSG00000165966
ENSG00000173157
ENSG00000111371
ENSG00000278896
ENSG00000272369
ENSG00000271642
ENSG00000274723
ENSG00000276454
ENSG00000257925
ENSG00000135451
ENSG00000066084
ENSG00000207136
ENSG00000271490
ENSG00000123358
ENSG00000259884
ENSG00000258279
ENSG00000167767
ENSG00000135480
ENSG00000257830
ENSG00000170523
ENSG00000241146
ENSG00000170421
ENSG00000161638
ENSG00000076067
ENSG00000276727

ENSG00000179912
ENSG00000111087
ENSG00000155980
ENSG00000258133
ENSG00000257288
ENSG00000061987
ENSG00000213352
ENSG00000184575
ENSG00000127334
ENSG00000279505
ENSG00000135678
ENSG00000257139
ENSG00000286259
ENSG00000258245
ENSG00000258091
ENSG00000243420
ENSG00000091039
ENSG00000286476
ENSG00000258084
ENSG00000067715
ENSG00000221788
ENSG00000257741
ENSG00000258375
ENSG00000257124
ENSG00000258358
ENSG00000133640

ENSG00000258206
ENSG00000289369
ENSG00000242850
ENSG00000258205
ENSG00000258179
ENSG00000139324
ENSG00000289174
ENSG00000257629
ENSG00000070961
ENSG00000252823
ENSG00000196243
ENSG00000258125
ENSG00000258224
ENSG00000257242
ENSG00000239201
ENSG00000277851
ENSG00000102189
ENSG00000202534
ENSG00000257210
ENSG00000257322
ENSG00000177889
ENSG00000173588
ENSG00000257847
ENSG00000057704
ENSG00000180263
ENSG00000074527

ENSG00000199506
ENSG00000252827
ENSG00000188596
ENSG00000139350
ENSG00000258204
ENSG00000257458
ENSG00000111647
ENSG00000151572
ENSG00000241917
ENSG00000257327
ENSG00000198431
ENSG00000213442
ENSG00000257732
ENSG00000289557
ENSG00000074590
ENSG00000258355
ENSG00000238609
ENSG00000166046
ENSG00000120832
ENSG00000008405
ENSG00000255650
ENSG00000289311
ENSG00000089009
ENSG00000276972
ENSG00000257726
ENSG00000258018

ENSG00000257465
ENSG00000176871
ENSG00000111707
ENSG00000256884
ENSG00000256609
ENSG00000213144
ENSG00000135114
ENSG00000089041
ENSG00000274292
ENSG00000214760
ENSG00000257097
ENSG00000130779
ENSG00000252192
ENSG00000280138
ENSG00000139722
ENSG00000280444
ENSG00000279931
ENSG00000265345
ENSG00000255853
ENSG00000286016
ENSG00000125207
ENSG00000256250
ENSG00000132341
ENSG00000280217
ENSG00000280319
ENSG00000256955

ENSG00000277011
ENSG00000090615
ENSG00000293217
ENSG00000150457
ENSG00000127863
ENSG00000252695
ENSG00000102699
ENSG00000260704
ENSG00000139514
ENSG00000132965
ENSG00000120694
ENSG00000277151
ENSG00000231910
ENSG00000261728
ENSG00000120669
ENSG00000120664
ENSG00000284584
ENSG00000241406
ENSG00000133106
ENSG00000229928
ENSG00000151773
ENSG00000227258
ENSG00000289852
ENSG00000276527
ENSG00000270381
ENSG00000199788

ENSG00000266611
ENSG00000271564
ENSG00000286820
ENSG00000234765
ENSG00000279754
ENSG00000230902
ENSG00000232132
ENSG00000284196
ENSG00000202398
ENSG00000226317
ENSG00000271395
ENSG00000215417
ENSG00000139793
ENSG00000286334
ENSG00000224418
ENSG00000102452
ENSG00000198542
ENSG00000174418
ENSG00000284966
ENSG00000201847
ENSG00000229938
ENSG00000236242
ENSG00000231428
ENSG00000265885
ENSG00000290808
ENSG00000068650

ENSG00000232487
ENSG00000258768
ENSG00000255804
ENSG00000258464
ENSG00000287481
ENSG00000258098
ENSG00000257845
ENSG00000240179
ENSG00000258282
ENSG00000252686
ENSG00000196792
ENSG00000257472
ENSG00000259045
ENSG00000287153
ENSG00000258897
ENSG00000259090
ENSG00000282625
ENSG00000258661
ENSG00000266327
ENSG00000258827
ENSG00000289903
ENSG00000129514
ENSG00000258680
ENSG00000258671
ENSG00000258940
ENSG00000258536

ENSG00000259149
ENSG00000258700
ENSG00000244053
ENSG00000165506
ENSG00000198513
ENSG00000258942
ENSG00000252400
ENSG00000186469
ENSG00000258928
ENSG00000271075
ENSG00000100526
ENSG00000100528
ENSG00000285774
ENSG00000168175
ENSG00000289523
ENSG00000131981
ENSG00000258413
ENSG00000131966
ENSG00000100612
ENSG00000027075
ENSG00000205767
ENSG00000221537
ENSG00000126822
ENSG00000222985
ENSG00000255002
ENSG00000244677

ENSG00000258477
ENSG00000287385
ENSG00000185650
ENSG00000273797
ENSG00000100650
ENSG00000242071
ENSG00000197555
ENSG00000259015
ENSG00000156030
ENSG00000119718
ENSG00000252013
ENSG00000289340
ENSG00000170345
ENSG00000271423
ENSG00000211448
ENSG00000258452
ENSG00000258605
ENSG00000042317
ENSG00000283627
ENSG00000258920
ENSG00000230997
ENSG00000133943
ENSG00000221102
ENSG00000015133
ENSG00000260711
ENSG00000100599

ENSG00000066455
ENSG00000258730
ENSG00000100605
ENSG00000241499
ENSG00000288028
ENSG00000259140
ENSG00000258572
ENSG00000247092
ENSG00000250366
ENSG00000260806
ENSG00000258393
ENSG00000258511
ENSG00000066629
ENSG00000259166
ENSG00000080823
ENSG00000266015
ENSG00000131323
ENSG00000213176
ENSG00000265291
ENSG00000203485
ENSG00000185567
ENSG00000140104
ENSG00000182809
ENSG00000213145
ENSG00000265929
ENSG00000259523

ENSG00000104067
ENSG00000169926
ENSG00000215296
ENSG00000261635
ENSG00000259423
ENSG00000104081
ENSG00000166143
ENSG00000128908
ENSG00000259740
ENSG00000273925
ENSG00000259216
ENSG00000285410
ENSG00000273674
ENSG00000069869
ENSG00000239703
ENSG00000137878
ENSG00000259771
ENSG00000157470
ENSG00000259223
ENSG00000277413
ENSG00000259466
ENSG00000103642
ENSG00000035664
ENSG00000270490
ENSG00000166949
ENSG00000259202

ENSG00000138604
ENSG00000245750
ENSG00000207965
ENSG00000166173
ENSG00000278408
ENSG00000166233
ENSG00000277391
ENSG00000138621
ENSG00000260152
ENSG00000260892
ENSG00000182950
ENSG00000269951
ENSG00000259708
ENSG00000169594
ENSG00000259630
ENSG00000259367
ENSG00000259620
ENSG00000207150
ENSG00000181026
ENSG00000172183
ENSG00000140511
ENSG00000261407
ENSG00000239151
ENSG00000259699
ENSG00000140548
ENSG00000259177

ENSG00000289066
ENSG00000258954
ENSG00000176463
ENSG00000258761
ENSG00000289017
ENSG00000258489
ENSG00000251729
ENSG00000264480
ENSG00000274294
ENSG00000259341
ENSG00000259579
ENSG00000279588
ENSG00000260710
ENSG00000260425
ENSG00000288935
ENSG00000261392
ENSG00000263033
ENSG00000153066
ENSG00000175604
ENSG00000274834
ENSG00000260009
ENSG00000215003
ENSG00000279620
ENSG00000011638
ENSG00000158486
ENSG00000283213

ENSG00000168447
ENSG00000279756
ENSG00000122257
ENSG00000289080
ENSG00000103507
ENSG00000169896
ENSG00000156886
ENSG00000166123
ENSG00000069345
ENSG00000280067
ENSG00000279842
ENSG00000121281
ENSG00000264947
ENSG00000261630
ENSG00000278928
ENSG00000159461
ENSG00000125124
ENSG00000051108
ENSG00000140853
ENSG00000260965
ENSG00000103034
ENSG00000261807
ENSG00000260834
ENSG00000261234
ENSG00000159713
ENSG00000159714

ENSG00000102901
ENSG00000262141
ENSG00000157349
ENSG00000279412
ENSG00000224470
ENSG00000251868
ENSG00000168404
ENSG00000103089
ENSG00000050820
ENSG00000261783
ENSG00000244485
ENSG00000275040
ENSG00000260876
ENSG00000260237
ENSG00000168589
ENSG00000260737
ENSG00000260064
ENSG00000166455
ENSG00000284473
ENSG00000260229
ENSG00000277255
ENSG00000275109
ENSG00000140945
ENSG00000277387
ENSG00000260832
ENSG00000261103

ENSG00000260228
ENSG00000153786
ENSG00000278716
ENSG00000260498
ENSG00000260177
ENSG00000225614
ENSG00000261253
ENSG00000267200
ENSG00000267195
ENSG00000280280
ENSG00000127804
ENSG00000243049
ENSG00000285471
ENSG00000285593
ENSG00000167858
ENSG00000219200
ENSG00000283025
ENSG00000065325
ENSG00000265593
ENSG00000007237
ENSG00000242692
ENSG00000170425
ENSG00000260328
ENSG00000154803
ENSG00000108557
ENSG00000072310

ENSG00000230273
ENSG00000188522
ENSG00000230607
ENSG00000225681
ENSG00000109046
ENSG00000265801
ENSG00000263709
ENSG00000010244
ENSG00000108671
ENSG00000283417
ENSG00000264622
ENSG00000132141
ENSG00000277268
ENSG00000167258
ENSG00000278834
ENSG00000171431
ENSG00000234477
ENSG00000108516
ENSG00000006059
ENSG00000278954
ENSG00000108861
ENSG00000267446
ENSG00000073861
ENSG00000136436
ENSG00000167080
ENSG00000108829

ENSG00000108846
ENSG00000262006
ENSG00000279743
ENSG00000108924
ENSG00000108960
ENSG00000236319
ENSG00000283209
ENSG00000274213
ENSG00000180891
ENSG00000284190
ENSG00000267416
ENSG00000108510
ENSG00000008283
ENSG00000007314
ENSG00000286629
ENSG00000270714
ENSG00000120063
ENSG00000263520
ENSG00000154240
ENSG00000289587
ENSG00000289877
ENSG00000265100
ENSG00000108932
ENSG00000267009
ENSG00000154262
ENSG00000237560

ENSG00000263680
ENSG00000179604
ENSG00000182938
ENSG00000267084
ENSG00000267737
ENSG00000279339
ENSG00000266711
ENSG00000167291
ENSG00000141519
ENSG00000287403
ENSG00000207736
ENSG00000182612
ENSG00000263098
ENSG00000263745
ENSG00000262001
ENSG00000266513
ENSG00000265545
ENSG00000200637
ENSG00000154845
ENSG00000285594
ENSG00000267069
ENSG00000128789
ENSG00000267393
ENSG00000273348
ENSG00000270855
ENSG00000289624

ENSG00000266495
ENSG00000267301
ENSG00000263862
ENSG00000275805
ENSG00000265374
ENSG00000266479
ENSG00000134757
ENSG00000266521
ENSG00000046604
ENSG00000265641
ENSG00000141425
ENSG00000286890
ENSG00000279678
ENSG00000279543
ENSG00000286328
ENSG00000280365
ENSG00000152229
ENSG00000141622
ENSG00000134030
ENSG00000280335
ENSG00000266997
ENSG00000267717
ENSG00000242945
ENSG00000178690
ENSG00000041353
ENSG00000267524

ENSG00000267311
ENSG00000258609
ENSG00000267726
ENSG00000074657
ENSG00000197641
ENSG00000197632
ENSG00000265217
ENSG00000081138
ENSG00000264104
ENSG00000287646
ENSG00000170677
ENSG00000133313
ENSG00000264116
ENSG00000265778
ENSG00000266256
ENSG00000275178
ENSG00000131196
ENSG00000099875
ENSG00000289471
ENSG00000105426
ENSG00000267262
ENSG00000267299
ENSG00000279827
ENSG00000181143
ENSG00000267332
ENSG00000267154

ENSG00000130813
ENSG00000079999
ENSG00000129353
ENSG00000141837
ENSG00000104957
ENSG00000267519
ENSG00000123146
ENSG00000141867
ENSG00000214049
ENSG00000289333
ENSG00000267275
ENSG00000127528
ENSG00000127511
ENSG00000127533
ENSG00000130313
ENSG00000130475
ENSG00000267178
ENSG00000169021
ENSG00000289030
ENSG00000270212
ENSG00000267580
ENSG00000243864
ENSG00000153879
ENSG00000130402
ENSG00000267375
ENSG00000207296

ENSG00000179134
ENSG00000288919
ENSG00000086544
ENSG00000028277
ENSG00000104783
ENSG00000073008
ENSG00000275726
ENSG00000142230
ENSG00000105327
ENSG00000289915
ENSG00000087074
ENSG00000104973
ENSG00000161652
ENSG00000105357
ENSG00000167754
ENSG00000179820
ENSG00000228323
ENSG00000080031
ENSG00000231274
ENSG00000143727
ENSG00000233684
ENSG00000284452
ENSG00000233633
ENSG00000115705
ENSG00000231482
ENSG00000203635

ENSG00000130508
ENSG00000242282
ENSG00000134326
ENSG00000272002
ENSG00000289191
ENSG00000236008
ENSG00000225642
ENSG00000213772
ENSG00000240960
ENSG00000243491
ENSG00000264030
ENSG00000115758
ENSG00000234818
ENSG00000226080
ENSG00000224177
ENSG00000223360
ENSG00000261117
ENSG00000229370
ENSG00000287291
ENSG00000197872
ENSG00000214843
ENSG00000236348
ENSG00000214842
ENSG00000212455
ENSG00000240857
ENSG00000237992

ENSG00000235911
ENSG00000183891
ENSG00000232800
ENSG00000290108
ENSG00000269976
ENSG00000233416
ENSG00000224165
ENSG00000268412
ENSG00000173557
ENSG00000213699
ENSG00000115163
ENSG00000205334
ENSG00000227938
ENSG00000230730
ENSG00000115295
ENSG00000119801
ENSG00000172954
ENSG00000278908
ENSG00000158125
ENSG00000230876
ENSG00000223951
ENSG00000152689
ENSG00000237790
ENSG00000171055
ENSG00000115808
ENSG00000236213

ENSG00000138061
ENSG00000199603
ENSG00000235653
ENSG00000289013
ENSG00000233128
ENSG00000231826
ENSG00000207087
ENSG00000230587
ENSG00000143919
ENSG00000286728
ENSG00000233829
ENSG00000232696
ENSG00000116016
ENSG00000180398
ENSG00000233845
ENSG00000226087
ENSG00000170802
ENSG00000272663
ENSG00000228033
ENSG00000251942
ENSG00000207456
ENSG00000289065
ENSG00000115306
ENSG00000237887
ENSG00000234943
ENSG00000115380

ENSG00000287875
ENSG00000162924
ENSG00000289410
ENSG00000228541
ENSG00000234488
ENSG00000143952
ENSG00000236148
ENSG00000226756
ENSG00000288986
ENSG00000287123
ENSG00000138069
ENSG00000230749
ENSG00000224071
ENSG00000237576
ENSG00000283586
ENSG00000169564
ENSG00000231327
ENSG00000229229
ENSG00000163235
ENSG00000144036
ENSG00000272702
ENSG00000224646
ENSG00000159399
ENSG00000229385
ENSG00000115423
ENSG00000213399

ENSG00000176407
ENSG00000115561
ENSG00000153561
ENSG00000168658
ENSG00000040933
ENSG00000238830
ENSG00000135945
ENSG00000230393
ENSG00000175874
ENSG00000071054
ENSG00000205716
ENSG00000277527
ENSG00000238273
ENSG00000227470
ENSG00000230696
ENSG00000233307
ENSG00000172965
ENSG00000237583
ENSG00000285016
ENSG00000136689
ENSG00000115084
ENSG00000125629
ENSG00000163075
ENSG00000163083
ENSG00000226708
ENSG00000186684

ENSG00000287742
ENSG00000201470
ENSG00000237532
ENSG00000136002
ENSG00000263813
ENSG00000115839
ENSG00000144224
ENSG00000231890
ENSG00000212425
ENSG00000232915
ENSG00000283506
ENSG00000244125
ENSG00000115919
ENSG00000121964
ENSG00000286225
ENSG00000226218
ENSG00000232238
ENSG00000228236
ENSG00000223604
ENSG00000286081
ENSG00000115963
ENSG00000232359
ENSG00000080345
ENSG00000227400
ENSG00000235548
ENSG00000153234

ENSG00000115159
ENSG00000136542
ENSG00000234584
ENSG00000115170
ENSG00000204380
ENSG00000188668
ENSG00000226878
ENSG00000123636
ENSG00000285373
ENSG00000283734
ENSG00000153250
ENSG00000115267
ENSG00000200902
ENSG00000182263
ENSG00000277998
ENSG00000236283
ENSG00000223318
ENSG00000169507
ENSG00000235192
ENSG00000144285
ENSG00000283657
ENSG00000238259
ENSG00000138398
ENSG00000213160
ENSG00000128692
ENSG00000288048

ENSG00000278924
ENSG00000228016
ENSG00000232292
ENSG00000091436
ENSG00000229337
ENSG00000222043
ENSG00000237178
ENSG00000187231
ENSG00000163510
ENSG00000221023
ENSG00000242121
ENSG00000061676
ENSG00000138448
ENSG00000213115
ENSG00000144366
ENSG00000231689
ENSG00000187699
ENSG00000151689
ENSG00000198130
ENSG00000290089
ENSG00000228509
ENSG00000115415
ENSG00000288900
ENSG00000224670
ENSG00000234919
ENSG00000201813

ENSG00000276200
ENSG00000196141
ENSG00000201649
ENSG00000244301
ENSG00000289698
ENSG00000055044
ENSG00000212534
ENSG00000240761
ENSG00000237843
ENSG00000223890
ENSG00000225216
ENSG00000114933
ENSG00000228577
ENSG00000118246
ENSG00000277590
ENSG00000221628
ENSG00000144401
ENSG00000202164
ENSG00000178568
ENSG00000223373
ENSG00000196096
ENSG00000265734
ENSG00000197756
ENSG00000287498
ENSG00000224819
ENSG00000234446

ENSG00000235225
ENSG00000123983
ENSG00000278083
ENSG00000226539
ENSG00000152056
ENSG00000228802
ENSG00000135919
ENSG00000224826
ENSG00000228446
ENSG00000144460
ENSG00000230621
ENSG00000235070
ENSG00000169047
ENSG00000115009
ENSG00000287791
ENSG00000279964
ENSG00000067066
ENSG00000226125
ENSG00000135932
ENSG00000201044
ENSG00000173692
ENSG00000177673
ENSG00000239202
ENSG00000163283
ENSG00000115468
ENSG00000204120

ENSG00000182600
ENSG00000222001
ENSG00000167165
ENSG00000261298
ENSG00000130147
ENSG00000157985
ENSG00000222007
ENSG00000144476
ENSG00000232328
ENSG00000222032
ENSG00000115648
ENSG00000283635
ENSG00000132326
ENSG00000204104
ENSG00000065802
ENSG00000229915
ENSG00000233608
ENSG00000220256
ENSG00000227479
ENSG00000063660
ENSG00000260942
ENSG00000286158
ENSG00000101255
ENSG00000125826
ENSG00000101266
ENSG00000234684

ENSG00000125775
ENSG00000286288
ENSG00000258713
ENSG00000215251
ENSG00000228210
ENSG00000277425
ENSG00000125772
ENSG00000171984
ENSG00000202380
ENSG00000228482
ENSG00000224711
ENSG00000101333
ENSG00000285723
ENSG00000235036
ENSG00000234900
ENSG00000217809
ENSG00000232900
ENSG00000282478
ENSG00000235698
ENSG00000172296
ENSG00000239923
ENSG00000125848
ENSG00000235914
ENSG00000089177
ENSG00000233625
ENSG00000149443

ENSG00000132669
ENSG00000225127
ENSG00000204684
ENSG00000215553
ENSG00000178726
ENSG00000233746
ENSG00000279164
ENSG00000201527
ENSG00000167390
ENSG00000277938
ENSG00000274414
ENSG00000101337
ENSG00000088305
ENSG00000125999
ENSG00000101421
ENSG00000289720
ENSG00000198646
ENSG00000244462
ENSG00000025293
ENSG00000219608
ENSG00000201221
ENSG00000259399
ENSG00000197122
ENSG00000101445
ENSG00000222612
ENSG00000198900

ENSG00000206801
ENSG00000124191
ENSG00000241229
ENSG00000132824
ENSG00000196839
ENSG00000124134
ENSG00000275894
ENSG00000124145
ENSG00000124155
ENSG00000221046
ENSG00000226769
ENSG00000289848
ENSG00000289895
ENSG00000124151
ENSG00000196562
ENSG00000238950
ENSG00000238785
ENSG00000234967
ENSG00000287443
ENSG00000229522
ENSG00000237423
ENSG00000287335
ENSG00000287107
ENSG00000288083
ENSG00000158470
ENSG00000197818

ENSG00000158480
ENSG00000293596
ENSG00000244687
ENSG00000172216
ENSG00000277449
ENSG00000203999
ENSG00000233077
ENSG00000244376
ENSG00000234693
ENSG00000124217
ENSG00000232358
ENSG00000026559
ENSG00000228820
ENSG00000266761
ENSG00000101096
ENSG00000215444
ENSG00000054793
ENSG00000286250
ENSG00000182463
ENSG00000171940
ENSG00000238468
ENSG00000287886
ENSG00000225563
ENSG00000236352
ENSG00000265595
ENSG00000019186

ENSG00000101132
ENSG00000275923
ENSG00000237000
ENSG00000231078
ENSG00000235032
ENSG00000275852
ENSG00000254995
ENSG00000289577
ENSG00000283078
ENSG00000229882
ENSG00000130589
ENSG00000249493
ENSG00000155304
ENSG00000223662
ENSG00000155313
ENSG00000279648
ENSG00000279534
ENSG00000229025
ENSG00000197934
ENSG00000154734
ENSG00000234052
ENSG00000236056
ENSG00000156282
ENSG00000166979
ENSG00000289939
ENSG00000180509

ENSG00000234380
ENSG00000286153
ENSG00000234703
ENSG00000223671
ENSG00000273199
ENSG00000237646
ENSG00000270652
ENSG00000212136
ENSG00000287637
ENSG00000286717
ENSG00000223806
ENSG00000157557
ENSG00000229986
ENSG00000227721
ENSG00000205622
ENSG00000288266
ENSG00000235888
ENSG00000263973
ENSG00000183486
ENSG00000227702
ENSG00000160179
ENSG00000223262
ENSG00000252619
ENSG00000287507
ENSG00000160213
ENSG00000226543

ENSG00000160216
ENSG00000228930
ENSG00000228404
ENSG00000099968
ENSG00000183628
ENSG00000223461
ENSG00000232926
ENSG00000289564
ENSG00000279085
ENSG00000278960
ENSG00000280025
ENSG00000231405
ENSG00000238195
ENSG00000227880
ENSG00000289489
ENSG00000226169
ENSG00000235954
ENSG00000100219
ENSG00000100263
ENSG00000185340
ENSG00000242156
ENSG00000233010
ENSG00000100325
ENSG00000289846
ENSG00000232530
ENSG00000128342

ENSG00000099995
ENSG00000237977
ENSG00000241878
ENSG00000100170
ENSG00000236054
ENSG00000229673
ENSG00000228194
ENSG00000273082
ENSG00000221963
ENSG00000128284
ENSG00000128335
ENSG00000100342
ENSG00000228719
ENSG00000226233
ENSG00000128283
ENSG00000100139
ENSG00000284266
ENSG00000279080
ENSG00000185022
ENSG00000184381
ENSG00000235246
ENSG00000100211
ENSG00000100242
ENSG00000284633
ENSG00000100311
ENSG00000289292

ENSG00000100243
ENSG00000274717
ENSG00000100266
ENSG00000186976
ENSG00000138944
ENSG00000220702
ENSG00000186654
ENSG00000056487
ENSG00000077942
ENSG00000238120
ENSG00000251985
ENSG00000130638
ENSG00000188064
ENSG00000075275
ENSG00000075240
ENSG00000054611
ENSG00000285722
ENSG00000100425
ENSG00000278869
ENSG00000073150
ENSG00000177989
ENSG00000224239
ENSG00000234325
ENSG00000235831
ENSG00000236202
ENSG00000071282

ENSG00000227110
ENSG00000132170
ENSG00000163520
ENSG00000255021
ENSG00000207163
ENSG00000232953
ENSG00000215016
ENSG00000170142
ENSG00000223791
ENSG00000197885
ENSG00000272166
ENSG00000206559
ENSG00000230111
ENSG00000289460
ENSG00000231382
ENSG00000201315
ENSG00000114742
ENSG00000168026
ENSG00000283849
ENSG00000233096
ENSG00000229339
ENSG00000114812
ENSG00000181061
ENSG00000144647
ENSG00000114648
ENSG00000164086

ENSG00000163931
ENSG00000213509
ENSG00000136068
ENSG00000163689
ENSG00000241804
ENSG00000163635
ENSG00000280620
ENSG00000163636
ENSG00000288039
ENSG00000163380
ENSG00000244203
ENSG00000290077
ENSG00000113805
ENSG00000240573
ENSG00000114480
ENSG00000242457
ENSG00000242911
ENSG00000286516
ENSG00000271671
ENSG00000080224
ENSG00000248839
ENSG00000207331
ENSG00000154175
ENSG00000081154
ENSG00000144802
ENSG00000214407

ENSG00000241280
ENSG00000170044
ENSG00000243635
ENSG00000279277
ENSG00000114446
ENSG00000221206
ENSG00000144824
ENSG00000286492
ENSG00000240893
ENSG00000206530
ENSG00000121579
ENSG00000242290
ENSG00000241295
ENSG00000289153
ENSG00000243359
ENSG00000241155
ENSG00000289134
ENSG00000244308
ENSG00000244139
ENSG00000163430
ENSG00000065518
ENSG00000288868
ENSG00000275348
ENSG00000138463
ENSG00000221955
ENSG00000287143

ENSG00000187715
ENSG00000285619
ENSG00000240854
ENSG00000249993
ENSG00000091527
ENSG00000286982
ENSG00000241438
ENSG00000249407
ENSG00000242244
ENSG00000286988
ENSG00000184432
ENSG00000248790
ENSG00000114120
ENSG00000114124
ENSG00000114127
ENSG00000251787
ENSG00000188313
ENSG00000293384
ENSG00000243885
ENSG00000252581
ENSG00000082996
ENSG00000264330
ENSG00000229619
ENSG00000287706
ENSG00000241671
ENSG00000290079

ENSG00000242925
ENSG00000240596
ENSG00000163659
ENSG00000174912
ENSG00000230457
ENSG00000240875
ENSG00000197980
ENSG00000241544
ENSG00000243629
ENSG00000243150
ENSG00000178053
ENSG00000241211
ENSG00000252763
ENSG00000243687
ENSG00000179674
ENSG00000271052
ENSG00000240138
ENSG00000114200
ENSG00000241479
ENSG00000239219
ENSG00000201342
ENSG00000173889
ENSG00000289233
ENSG00000239739
ENSG00000075651
ENSG00000287899

ENSG00000075420
ENSG00000279862
ENSG00000114346
ENSG00000228221
ENSG00000225790
ENSG00000240063
ENSG00000176597
ENSG00000177383
ENSG00000273403
ENSG00000073792
ENSG00000136514
ENSG00000145012
ENSG00000288794
ENSG00000207651
ENSG00000230115
ENSG00000073282
ENSG00000216058
ENSG00000230077
ENSG00000163347
ENSG00000113946
ENSG00000198398
ENSG00000196083
ENSG00000180611
ENSG00000229155
ENSG00000214146
ENSG00000242201

ENSG00000233058
ENSG00000227433
ENSG00000176945
ENSG00000145113
ENSG00000235836
ENSG00000223783
ENSG00000145107
ENSG00000075711
ENSG00000122068
ENSG00000182899
ENSG00000244459
ENSG00000280310
ENSG00000074211
ENSG00000186222
ENSG00000266690
ENSG00000272620
ENSG00000273267
ENSG00000249631
ENSG00000250634
ENSG00000224569
ENSG00000251644
ENSG00000137440
ENSG00000109606
ENSG00000168214
ENSG00000250954
ENSG00000154274

ENSG00000287659
ENSG00000121895
ENSG00000249241
ENSG00000239010
ENSG00000109220
ENSG00000251923
ENSG00000251339
ENSG00000283043
ENSG00000249066
ENSG00000083896
ENSG00000187689
ENSG00000222236
ENSG00000056291
ENSG00000156140
ENSG00000163734
ENSG00000163738
ENSG00000250560
ENSG00000138772
ENSG00000201901
ENSG00000248196
ENSG00000152595
ENSG00000276003
ENSG00000184305
ENSG00000250908
ENSG00000070190
ENSG00000286150

ENSG00000286124
ENSG00000109320
ENSG00000251577
ENSG00000169836
ENSG00000200917
ENSG00000164023
ENSG00000222691
ENSG00000206820
ENSG00000248488
ENSG00000249002
ENSG00000224062
ENSG00000244083
ENSG00000151458
ENSG00000248715
ENSG00000251555
ENSG00000249463
ENSG00000249311
ENSG00000151012
ENSG00000249091
ENSG00000250577
ENSG00000252362
ENSG00000251248
ENSG00000249806
ENSG00000286771
ENSG00000170180
ENSG00000237136

ENSG00000279481
ENSG00000151612
ENSG00000221369
ENSG00000249004
ENSG00000279016
ENSG00000249317
ENSG00000252451
ENSG00000109686
ENSG00000170006
ENSG00000249022
ENSG00000121211
ENSG00000121210
ENSG00000287216
ENSG00000249013
ENSG00000286133
ENSG00000248988
ENSG00000250997
ENSG00000145416
ENSG00000252959
ENSG00000137628
ENSG00000207171
ENSG00000248551
ENSG00000286119
ENSG00000251878
ENSG00000164323
ENSG00000109775

ENSG00000154553
ENSG00000249539
ENSG00000248370
ENSG00000214278
ENSG00000188242
ENSG00000271781
ENSG00000249908
ENSG00000113504
ENSG00000273742
ENSG00000153395
ENSG00000288108
ENSG00000250481
ENSG00000251568
ENSG00000286986
ENSG00000215217
ENSG00000250198
ENSG00000266415
ENSG00000248537
ENSG00000279698
ENSG00000248525
ENSG00000279011
ENSG00000249396
ENSG00000279450
ENSG00000164236
ENSG00000278865
ENSG00000207312

ENSG00000212305
ENSG00000242608
ENSG00000039139
ENSG00000038382
ENSG00000287581
ENSG00000249485
ENSG00000289112
ENSG00000253093
ENSG00000243538
ENSG00000185296
ENSG00000293053
ENSG00000248693
ENSG00000248766
ENSG00000214132
ENSG00000145526
ENSG00000249490
ENSG00000253766
ENSG00000223004
ENSG00000251593
ENSG00000252601
ENSG00000241668
ENSG00000243914
ENSG00000214108
ENSG00000056097
ENSG00000113387
ENSG00000251281

ENSG00000240376
ENSG00000201623
ENSG00000151388
ENSG00000271874
ENSG00000152582
ENSG00000168685
ENSG00000279737
ENSG00000152620
ENSG00000079215
ENSG00000164190
ENSG00000251257
ENSG00000113594
ENSG00000251189
ENSG00000249740
ENSG00000145623
ENSG00000164327
ENSG00000286809
ENSG00000251215
ENSG00000082074
ENSG00000283286
ENSG00000248120
ENSG00000182836
ENSG00000151876
ENSG00000249266
ENSG00000250722
ENSG00000287263

ENSG00000177453
ENSG00000151882
ENSG00000172239
ENSG00000248092
ENSG00000249276
ENSG00000249203
ENSG00000250418
ENSG00000112996
ENSG00000272335
ENSG00000151883
ENSG00000251873
ENSG00000289152
ENSG00000062194
ENSG00000276214
ENSG00000202601
ENSG00000249279
ENSG00000248185
ENSG00000153006
ENSG00000229666
ENSG00000197822
ENSG00000212260
ENSG00000272081
ENSG00000288883
ENSG00000244326
ENSG00000213763
ENSG00000248909

ENSG00000152409
ENSG00000152348
ENSG00000186468
ENSG00000264342
ENSG00000248323
ENSG00000207578
ENSG00000249180
ENSG00000164307
ENSG00000176183
ENSG00000174132
ENSG00000226926
ENSG00000253776
ENSG00000289260
ENSG00000272523
ENSG00000151422
ENSG00000251201
ENSG00000287148
ENSG00000268223
ENSG00000271918
ENSG00000250847
ENSG00000113083
ENSG00000164185
ENSG00000186687
ENSG00000273217
ENSG00000253067
ENSG00000131435

ENSG00000238160
ENSG00000272023
ENSG00000279691
ENSG00000270177
ENSG00000239615
ENSG00000250994
ENSG00000224186
ENSG00000222285
ENSG00000152377
ENSG00000177733
ENSG00000113013
ENSG00000120306
ENSG00000113068
ENSG00000250347
ENSG00000225147
ENSG00000248125
ENSG00000156463
ENSG00000248493
ENSG00000113649
ENSG00000283653
ENSG00000169252
ENSG00000289430
ENSG00000272411
ENSG00000250072
ENSG00000173210
ENSG00000276365

ENSG00000230551
ENSG00000222213
ENSG00000113721
ENSG00000183876
ENSG00000171992
ENSG00000289970
ENSG00000145901
ENSG00000254226
ENSG00000249484
ENSG00000253886
ENSG00000264760
ENSG00000253370
ENSG00000285868
ENSG00000113282
ENSG00000253422
ENSG00000253315
ENSG00000252458
ENSG00000283413
ENSG00000253311
ENSG00000270612
ENSG00000038274
ENSG00000254130
ENSG00000253469
ENSG00000254365
ENSG00000254187
ENSG00000253357

ENSG00000253660
ENSG00000113645
ENSG00000040275
ENSG00000253269
ENSG00000204767
ENSG00000168269
ENSG00000253219
ENSG00000249601
ENSG00000253858
ENSG00000253591
ENSG00000156427
ENSG00000253110
ENSG00000072803
ENSG00000072786
ENSG00000168246
ENSG00000214357
ENSG00000253295
ENSG00000120129
ENSG00000290019
ENSG00000288737
ENSG00000113719
ENSG00000254295
ENSG00000204758
ENSG00000113732
ENSG00000252169
ENSG00000113739

ENSG00000145919
ENSG00000287003
ENSG00000254011
ENSG00000249306
ENSG00000213376
ENSG00000248355
ENSG00000145920
ENSG00000278970
ENSG00000183718
ENSG00000263667
ENSG00000272463
ENSG00000270937
ENSG00000054598
ENSG00000285603
ENSG00000272465
ENSG00000280423
ENSG00000164385
ENSG00000021355
ENSG00000230438
ENSG00000124570
ENSG00000137267
ENSG00000272320
ENSG00000238158
ENSG00000260239
ENSG00000260673
ENSG00000217030

ENSG00000287497
ENSG00000226281
ENSG00000259040
ENSG00000216781
ENSG00000137203
ENSG00000238076
ENSG00000137434
ENSG00000111837
ENSG00000287920
ENSG00000290062
ENSG00000111859
ENSG00000242753
ENSG00000233656
ENSG00000111863
ENSG00000095951
ENSG00000218893
ENSG00000212802
ENSG00000289958
ENSG00000229931
ENSG00000112186
ENSG00000272269
ENSG00000220920
ENSG00000124795
ENSG00000201523
ENSG00000200957
ENSG00000227803

ENSG00000233848
ENSG00000280443
ENSG00000227766
ENSG00000263608
ENSG00000271581
ENSG00000197251
ENSG00000216636
ENSG00000265123
ENSG00000187762
ENSG00000023892
ENSG00000227154
ENSG00000112081
ENSG00000146192
ENSG00000216412
ENSG00000137193
ENSG00000065491
ENSG00000112139
ENSG00000183826
ENSG00000219273
ENSG00000164626
ENSG00000124602
ENSG00000112559
ENSG00000188112
ENSG00000048544
ENSG00000112624
ENSG00000172432

ENSG00000236961
ENSG00000272114
ENSG00000283573
ENSG00000289313
ENSG00000231881
ENSG00000237530
ENSG00000261080
ENSG00000112782
ENSG00000112818
ENSG00000153292
ENSG00000146072
ENSG00000287137
ENSG00000197261
ENSG00000270306
ENSG00000214641
ENSG00000289276
ENSG00000261745
ENSG00000235899
ENSG00000231683
ENSG00000137269
ENSG00000213335
ENSG00000168143
ENSG00000261116
ENSG00000227602
ENSG00000219653
ENSG00000238949

ENSG00000271111
ENSG00000223504
ENSG00000285844
ENSG00000220515
ENSG00000290002
ENSG00000272243
ENSG00000112701
ENSG00000285401
ENSG00000261970
ENSG00000198478
ENSG00000083123
ENSG00000279022
ENSG00000260645
ENSG00000181705
ENSG00000226089
ENSG00000260574
ENSG00000286875
ENSG00000083097
ENSG00000065833
ENSG00000065615
ENSG00000135324
ENSG00000218313
ENSG00000220960
ENSG00000146281
ENSG00000237027
ENSG00000260271

ENSG00000289988
ENSG00000135341
ENSG00000289178
ENSG00000233797
ENSG00000216378
ENSG00000218173
ENSG00000286084
ENSG00000269919
ENSG00000282408
ENSG00000225174
ENSG00000228976
ENSG00000203801
ENSG00000203799
ENSG00000135587
ENSG00000185250
ENSG00000155085
ENSG00000112290
ENSG00000155115
ENSG00000255389
ENSG00000265725
ENSG00000111897
ENSG00000236548
ENSG00000111907
ENSG00000286299
ENSG00000222445
ENSG00000111911

ENSG00000287731
ENSG00000220744
ENSG00000172673
ENSG00000224733
ENSG00000152894
ENSG00000233351
ENSG00000218689
ENSG00000112319
ENSG00000272428
ENSG00000213118
ENSG00000213117
ENSG00000240056
ENSG00000135541
ENSG00000029363
ENSG00000027697
ENSG00000234956
ENSG00000225177
ENSG00000225415
ENSG00000135597
ENSG00000112406
ENSG00000286452
ENSG00000259828
ENSG00000222764
ENSG00000112414
ENSG00000233138
ENSG00000237851

ENSG00000289850
ENSG00000118508
ENSG00000118492
ENSG00000224658
ENSG00000111962
ENSG00000283608
ENSG00000131013
ENSG00000201628
ENSG00000220598
ENSG00000232290
ENSG00000233823
ENSG00000146469
ENSG00000220181
ENSG00000235381
ENSG00000238789
ENSG00000271551
ENSG00000271899
ENSG00000233044
ENSG00000266617
ENSG00000233496
ENSG00000219249
ENSG00000233893
ENSG00000092820
ENSG00000285159
ENSG00000203711
ENSG00000288845

ENSG00000197081
ENSG00000230234
ENSG00000112499
ENSG00000231863
ENSG00000227913
ENSG00000185345
ENSG00000225683
ENSG00000281692
ENSG00000217514
ENSG00000270419
ENSG00000112531
ENSG00000206681
ENSG00000276643
ENSG00000286760
ENSG00000130396
ENSG00000235815
ENSG00000225879
ENSG00000289623
ENSG00000273100
ENSG00000261003
ENSG00000223855
ENSG00000164828
ENSG00000198517
ENSG00000106266
ENSG00000008256
ENSG00000136238

ENSG00000244167
ENSG00000106460
ENSG00000225606
ENSG00000230879
ENSG00000229688
ENSG00000272537
ENSG00000228675
ENSG00000221393
ENSG00000225000
ENSG00000136244
ENSG00000122591
ENSG00000233760
ENSG00000106049
ENSG00000146592
ENSG00000223437
ENSG00000240583
ENSG00000236494
ENSG00000286300
ENSG00000011426
ENSG00000006715
ENSG00000252480
ENSG00000238284
ENSG00000260997
ENSG00000146678
ENSG00000229628
ENSG00000237760

ENSG00000230680
ENSG00000224519
ENSG00000223829
ENSG00000228173
ENSG00000226122
ENSG00000106078
ENSG00000228627
ENSG00000218586
ENSG00000146648
ENSG00000224057
ENSG00000291136
ENSG00000154710
ENSG00000236978
ENSG00000106077
ENSG00000189143
ENSG00000232415
ENSG00000238391
ENSG00000251798
ENSG00000290729
ENSG00000146700
ENSG00000243566
ENSG00000186088
ENSG00000234456
ENSG00000234223
ENSG00000293394
ENSG00000230244

ENSG00000075213
ENSG00000227982
ENSG00000235139
ENSG00000235243
ENSG00000127954
ENSG00000105792
ENSG00000235450
ENSG00000001631
ENSG00000206763
ENSG00000205413
ENSG00000236938
ENSG00000127995
ENSG00000219807
ENSG00000004799
ENSG00000279067
ENSG00000127922
ENSG00000164715
ENSG00000180535
ENSG00000272950
ENSG00000214389
ENSG00000006453
ENSG00000002079
ENSG00000293408
ENSG00000106261
ENSG00000227053
ENSG00000106366

ENSG00000106436
ENSG00000257923
ENSG00000259294
ENSG00000272219
ENSG00000261535
ENSG00000105821
ENSG00000222386
ENSG00000005483
ENSG00000286076
ENSG00000267052
ENSG00000228742
ENSG00000164597
ENSG00000091128
ENSG00000226965
ENSG00000173114
ENSG00000184903
ENSG00000233607
ENSG00000202377
ENSG00000105967
ENSG00000279086
ENSG00000105974
ENSG00000231210
ENSG00000105976
ENSG00000235945
ENSG00000227199
ENSG00000106299

ENSG00000106302
ENSG00000292309
ENSG00000135253
ENSG00000128578
ENSG00000106477
ENSG00000273319
ENSG00000128585
ENSG00000231721
ENSG00000233287
ENSG00000236753
ENSG00000224545
ENSG00000189320
ENSG00000122778
ENSG00000105939
ENSG00000064393
ENSG00000006459
ENSG00000211714
ENSG00000211728
ENSG00000253534
ENSG00000253291
ENSG00000276405
ENSG00000188037
ENSG00000273314
ENSG00000286180
ENSG00000239719
ENSG00000127399

ENSG00000271582
ENSG00000243836
ENSG00000290095
ENSG00000228521
ENSG00000278998
ENSG00000204876
ENSG00000229660
ENSG00000285821
ENSG00000289418
ENSG00000207637
ENSG00000180190
ENSG00000285957
ENSG00000276580
ENSG00000253505
ENSG00000250305
ENSG00000253257
ENSG00000104723
ENSG00000254242
ENSG00000250316
ENSG00000253775
ENSG00000287812
ENSG00000120889
ENSG00000147454
ENSG00000199620
ENSG00000253397
ENSG00000254129

ENSG00000232024
ENSG00000253452
ENSG00000165046
ENSG00000253665
ENSG00000176907
ENSG00000253611
ENSG00000253782
ENSG00000290028
ENSG00000254065
ENSG00000169139
ENSG00000253654
ENSG00000253702
ENSG00000253484
ENSG00000289095
ENSG00000253849
ENSG00000168300
ENSG00000288818
ENSG00000023287
ENSG00000260484
ENSG00000272457
ENSG00000187735
ENSG00000270980
ENSG00000253114
ENSG00000199405
ENSG00000254055
ENSG00000137575

ENSG00000242970
ENSG00000035681
ENSG00000201231
ENSG00000253260
ENSG00000254777
ENSG00000251127
ENSG00000254119
ENSG00000261542
ENSG00000280725
ENSG00000206949
ENSG00000241961
ENSG00000255130
ENSG00000253658
ENSG00000253173
ENSG00000213003
ENSG00000104369
ENSG00000164749
ENSG00000253952
ENSG00000254001
ENSG00000261618
ENSG00000238884
ENSG00000276418
ENSG00000254205
ENSG00000205189
ENSG00000243951
ENSG00000286763

ENSG00000253859
ENSG00000164695
ENSG00000104231
ENSG00000258256
ENSG00000147614
ENSG00000227753
ENSG00000164893
ENSG00000085719
ENSG00000253171
ENSG00000248231
ENSG00000104312
ENSG00000180694
ENSG00000147606
ENSG00000253901
ENSG00000183808
ENSG00000244217
ENSG00000283522
ENSG00000272249
ENSG00000180543
ENSG00000147649
ENSG00000253415
ENSG00000164920
ENSG00000104450
ENSG00000248599
ENSG00000253395
ENSG00000254024

ENSG00000253669
ENSG00000244052
ENSG00000253851
ENSG00000164930
ENSG00000247081
ENSG00000288945
ENSG00000283157
ENSG00000254141
ENSG00000251003
ENSG00000253582
ENSG00000253796
ENSG00000248050
ENSG00000253756
ENSG00000239917
ENSG00000182197
ENSG00000225885
ENSG00000177570
ENSG00000242206
ENSG00000147676
ENSG00000253972
ENSG00000239872
ENSG00000248690
ENSG00000254209
ENSG00000253814
ENSG00000238901
ENSG00000263443

ENSG00000272384
ENSG00000178764
ENSG00000136986
ENSG00000253286
ENSG00000175946
ENSG00000104537
ENSG00000206724
ENSG00000253227
ENSG00000164961
ENSG00000242170
ENSG00000253111
ENSG00000286122
ENSG00000253427
ENSG00000207138
ENSG00000253530
ENSG00000253438
ENSG00000253573
ENSG00000246228
ENSG00000286266
ENSG00000283710
ENSG00000278324
ENSG00000280055
ENSG00000207110
ENSG00000221176
ENSG00000221261
ENSG00000286034

ENSG00000254275
ENSG00000285108
ENSG00000287195
ENSG00000253043
ENSG00000153310
ENSG00000212342
ENSG00000287201
ENSG00000253656
ENSG00000132294
ENSG00000129295
ENSG00000042832
ENSG00000104415
ENSG00000253638
ENSG00000271048
ENSG00000279518
ENSG00000279786
ENSG00000253266
ENSG00000253210
ENSG00000280035
ENSG00000254197
ENSG00000160886
ENSG00000277494
ENSG00000287465
ENSG00000147852
ENSG00000080298
ENSG00000232104

ENSG00000228322
ENSG00000153714
ENSG00000235448
ENSG00000213954
ENSG00000205549
ENSG00000178031
ENSG00000227167
ENSG00000147872
ENSG00000205442
ENSG00000252580
ENSG00000232203
ENSG00000086061
ENSG00000223394
ENSG00000107341
ENSG00000274157
ENSG00000184906
ENSG00000289843
ENSG00000286506
ENSG00000198312
ENSG00000182021
ENSG00000290559
ENSG00000290549
ENSG00000176134
ENSG00000225883
ENSG00000287988
ENSG00000224599

ENSG00000235893
ENSG00000279955
ENSG00000290938
ENSG00000107242
ENSG00000270236
ENSG00000241174
ENSG00000165072
ENSG00000272232
ENSG00000119125
ENSG00000107372
ENSG00000289993
ENSG00000236849
ENSG00000135046
ENSG00000202217
ENSG00000134996
ENSG00000099139
ENSG00000231137
ENSG00000225937
ENSG00000228248
ENSG00000156052
ENSG00000135069
ENSG00000237257
ENSG00000106829
ENSG00000228375
ENSG00000207206
ENSG00000252167

ENSG00000233309
ENSG00000165105
ENSG00000286451
ENSG00000172159
ENSG00000148057
ENSG00000165119
ENSG00000270166
ENSG00000233262
ENSG00000285987
ENSG00000148053
ENSG00000226590
ENSG00000287930
ENSG00000287333
ENSG00000236709
ENSG00000135047
ENSG00000290883
ENSG00000213694
ENSG00000123975
ENSG00000263967
ENSG00000231107
ENSG00000287212
ENSG00000260454
ENSG00000237422
ENSG00000230147
ENSG00000165030
ENSG00000233081

ENSG00000228595
ENSG00000283351
ENSG00000169071
ENSG00000090054
ENSG00000264158
ENSG00000213669
ENSG00000127084
ENSG00000283146
ENSG00000165140
ENSG00000252153
ENSG00000224764
ENSG00000285807
ENSG00000274115
ENSG00000236130
ENSG00000106785
ENSG00000119514
ENSG00000267026
ENSG00000270412
ENSG00000252942
ENSG00000271086
ENSG00000136874
ENSG00000136891
ENSG00000066697
ENSG00000286243
ENSG00000136783
ENSG00000226334

ENSG00000230013
ENSG00000201583
ENSG00000286536
ENSG00000070214
ENSG00000106701
ENSG00000229297
ENSG00000148143
ENSG00000119318
ENSG00000287233
ENSG00000289987
ENSG00000136826
ENSG00000227350
ENSG00000222558
ENSG00000244104
ENSG00000213557
ENSG00000237360
ENSG00000230030
ENSG00000169253
ENSG00000070159
ENSG00000263861
ENSG00000228053
ENSG00000136810
ENSG00000198121
ENSG00000136813
ENSG00000165181
ENSG00000266315

ENSG00000259953
ENSG00000106868
ENSG00000250068
ENSG00000237073
ENSG00000233817
ENSG00000229314
ENSG00000228278
ENSG00000106948
ENSG00000271214
ENSG00000095397
ENSG00000235797
ENSG00000136888
ENSG00000157693
ENSG00000230284
ENSG00000173077
ENSG00000106952
ENSG00000041982
ENSG00000234692
ENSG00000256040
ENSG00000119401
ENSG00000285820
ENSG00000225050
ENSG00000106780
ENSG00000239593
ENSG00000227355
ENSG00000119446

ENSG00000095303
ENSG00000233616
ENSG00000119408
ENSG00000227200
ENSG00000136930
ENSG00000185585
ENSG00000239705
ENSG00000243845
ENSG00000253079
ENSG00000169155
ENSG00000167106
ENSG00000230536
ENSG00000148346
ENSG00000226355
ENSG00000261334
ENSG00000227619
ENSG00000167157
ENSG00000236024
ENSG00000288924
ENSG00000130707
ENSG00000130720
ENSG00000288701
ENSG00000240853
ENSG00000188523
ENSG00000224992
ENSG00000165698

ENSG00000160271
ENSG00000263897
ENSG00000223795
ENSG00000130635
ENSG00000279141
ENSG00000198435
ENSG00000188747
ENSG00000169059
ENSG00000279682
ENSG00000219870
ENSG00000291314
ENSG00000207202
ENSG00000004961
ENSG00000288817
ENSG00000197582
ENSG00000123595
ENSG00000101958
ENSG00000102010
ENSG00000087842
ENSG00000147010
ENSG00000283631
ENSG00000233067
ENSG00000224396
ENSG00000201095
ENSG00000234230
ENSG00000227393

ENSG00000264090
ENSG00000276940
ENSG00000289127
ENSG00000251807
ENSG00000183690
ENSG00000068438
ENSG00000272080
ENSG00000238190
ENSG00000224735
ENSG00000188021
ENSG00000147065
ENSG00000289038
ENSG00000181191
ENSG00000131263
ENSG00000227521
ENSG00000102359
ENSG00000123576
ENSG00000213569
ENSG00000289923
ENSG00000189372
ENSG00000224142
ENSG00000243978
ENSG00000271421
ENSG00000123496
ENSG00000180772
ENSG00000238066

ENSG00000264933
ENSG00000101972
ENSG00000232412
ENSG00000183631
ENSG00000171388
ENSG00000232160
ENSG00000206765
ENSG00000226107
ENSG00000129682
ENSG00000102081
ENSG00000147381
ENSG00000067646
ENSG00000233774
ENSG00000183878
ENSG00000227166
ENSG00000252323
ENSG00000176728

Table S6. Up-regulated genes of BLCA cell lines.

	Gene_name	Gene_id
1	5_8S_rRNA	ENSG00000283568
2	AARS2	ENSG00000124608
3	AASS	ENSG00000008311
4	ABHD16A	ENSG00000204427

5	AC004803.1	ENSG00000250132
6	AC006435.1	ENSG00000262456
7	AC008738.7	ENSG00000273420
8	AC008875.3	ENSG00000287263
9	AC009005.1	ENSG00000267751
10	AC009237.14	ENSG00000272913
11	AC009779.2	ENSG00000258056
12	AC010198.2	ENSG00000285517
13	AC010327.4	ENSG00000267577
14	AC011043.1	ENSG00000276256
15	AC011446.1	ENSG00000267512
16	AC021016.2	ENSG00000273361
17	AC022384.1	ENSG00000272410
18	AC022415.2	ENSG00000286132
19	AC024592.3	ENSG00000267740
20	AC037459.1	ENSG00000248235
21	AC083855.2	ENSG00000285906
22	AC091167.1	ENSG00000228998
23	AC099066.2	ENSG00000227496
24	AC125611.3	ENSG00000258232
25	AC145212.1	ENSG00000277400
26	AC233992.3	ENSG00000272115
27	AC245140.2	ENSG00000280195
28	ACO1	ENSG00000122729
29	ACOT1	ENSG00000184227
30	ACOT2	ENSG00000119673

31	ACVR1	ENSG00000115170
32	ADAM23	ENSG00000114948
33	ADAT3	ENSG00000213638
34	ADGRD1	ENSG00000111452
35	ADGRL2	ENSG00000117114
36	AGPAT1	ENSG00000204310
37	AHNAK2	ENSG00000185567
38	AKR1C1	ENSG00000187134
39	AKR1C2	ENSG00000151632
40	AKR1C3	ENSG00000196139
41	AL133410.3	ENSG00000285645
42	AL590666.2	ENSG00000229953
43	ALKBH7	ENSG00000125652
44	ANK2	ENSG00000145362
45	ANTXR2	ENSG00000163297
46	ANXA5	ENSG00000164111
47	ANXA6	ENSG00000197043
48	APLN	ENSG00000171388
49	APOBEC3D	ENSG00000243811
50	APOC1	ENSG00000130208
51	ARF3	ENSG00000134287
52	ARHGAP24	ENSG00000138639
53	ARHGEF25	ENSG00000240771
54	ARHGEF9	ENSG00000131089
55	ARL2	ENSG00000213465
56	ARMCX1	ENSG00000126947

57	ARPC4-TTLL3	ENSG00000250151
58	ARSJ	ENSG00000180801
59	ATF5	ENSG00000169136
60	ATF6B	ENSG00000213676
61	ATG7	ENSG00000197548
62	ATN1	ENSG00000111676
63	ATP11C	ENSG00000101974
64	ATP2B1	ENSG00000070961
65	ATP5MC2	ENSG00000135390
66	ATP6V0B	ENSG00000117410
67	B4GALNT1	ENSG00000135454
68	B4GALT6	ENSG00000118276
69	BAG1	ENSG00000107262
70	BAG2	ENSG00000112208
71	BAG6	ENSG00000204463
72	BASP1	ENSG00000176788
73	BBOX1-AS1	ENSG00000254560
74	BCAT1	ENSG00000060982
75	BCL2	ENSG00000171791
76	BCL2A1	ENSG00000140379
77	BCL6	ENSG00000113916
78	BCYRN1	ENSG00000236824
79	BEX3	ENSG00000166681
80	BLOC1S1	ENSG00000135441
81	BMI1	ENSG00000168283
82	BNC2	ENSG00000173068

83	BRD2	ENSG00000204256
84	BRPF3	ENSG00000096070
85	BSG	ENSG00000172270
86	BTN2A1	ENSG00000112763
87	BTN3A2	ENSG00000186470
88	BX640514.2	ENSG00000273812
89	C12orf57	ENSG00000111678
90	C6orf89	ENSG00000198663
91	CA11	ENSG00000063180
92	CAB39L	ENSG00000102547
93	CACNA2D1	ENSG00000153956
94	CADPS2	ENSG00000081803
95	CAMK2D	ENSG00000145349
96	CAMK2N2	ENSG00000163888
97	CASC9	ENSG00000249395
98	CASP1	ENSG00000137752
99	CAVIN3	ENSG00000170955
100	CC2D2A	ENSG00000048342
101	CCDC107	ENSG00000159884
102	CCDC167	ENSG00000198937
103	CCDC169	ENSG00000242715
104	CD163L1	ENSG00000177675
105	CD320	ENSG00000167775
106	CD63	ENSG00000135404
107	CDK4	ENSG00000135446
108	CEACAM6	ENSG00000086548

109	CEBPB	ENSG00000172216
110	CELSR3	ENSG00000008300
111	CFD	ENSG00000197766
112	CGAS	ENSG00000164430
113	CHAC1	ENSG00000128965
114	CHPF2	ENSG00000033100
115	CHST2	ENSG00000175040
116	CKAP4	ENSG00000136026
117	CLEC2B	ENSG00000110852
118	CLGN	ENSG00000153132
119	CLIC1	ENSG00000213719
120	CLMP	ENSG00000166250
121	CLTA	ENSG00000122705
122	CMBL	ENSG00000164237
123	CNIH3-AS2	ENSG00000233384
124	CNKSR3	ENSG00000153721
125	CNN3-DT	ENSG00000235501
126	COPS7A	ENSG00000111652
127	CPT1C	ENSG00000169169
128	CRACD	ENSG00000109265
129	CREB3	ENSG00000107175
130	CSF1	ENSG00000184371
131	CTDSP2	ENSG00000175215
132	CTSC	ENSG00000109861
133	CTSO	ENSG00000256043
134	CXCL2	ENSG00000081041

135	CXCL3	ENSG00000163734
136	CYB561A3	ENSG00000162144
137	CYP4V2	ENSG00000145476
138	DCLK2	ENSG00000170390
139	DCP1B	ENSG00000151065
140	DDIT3	ENSG00000175197
141	DECR1	ENSG00000104325
142	DENND2A	ENSG00000146966
143	DENND5B	ENSG00000170456
144	DGLUCY	ENSG00000133943
145	DNAJB5	ENSG00000137094
146	DNER	ENSG00000187957
147	DOCK10	ENSG00000135905
148	DOCK11	ENSG00000147251
149	DPP9	ENSG00000142002
150	DSTNP2	ENSG00000248593
151	DTX3	ENSG00000178498
152	DUS3L	ENSG00000141994
153	DUSP5	ENSG00000138166
154	DXO	ENSG00000204348
155	EEF2	ENSG00000167658
156	EHMT2	ENSG00000204371
157	EIF5AP4	ENSG00000234743
158	ELAPOR2	ENSG00000164659
159	ELMOD1	ENSG00000110675
160	ELOVL6	ENSG00000170522

161	EMILIN2	ENSG00000132205
162	EMP3	ENSG00000142227
163	EN1	ENSG00000163064
164	ENO2	ENSG00000111674
165	ESYT1	ENSG00000139641
166	ETHE1	ENSG00000105755
167	ETNK1	ENSG00000139163
168	ETV1	ENSG00000006468
169	EVI2A	ENSG00000126860
170	EXOSC4	ENSG00000178896
171	EXTL2	ENSG00000162694
172	FABP5	ENSG00000164687
173	FABP5P7	ENSG00000234964
174	FAM155A	ENSG00000204442
175	FAM174C	ENSG00000228300
176	FAM189B	ENSG00000160767
177	FAM225A	ENSG00000231528
178	FAM234B	ENSG00000084444
179	FAM43A	ENSG00000185112
180	FAM89B	ENSG00000176973
181	FAR2	ENSG00000064763
182	FASN	ENSG00000169710
183	FAT1	ENSG00000083857
184	FBXO9	ENSG00000112146
185	FGF13	ENSG00000129682
186	FGF2	ENSG00000138685

187	FGFR1	ENSG00000077782
188	FLRT2	ENSG00000185070
189	FMNL3	ENSG00000161791
190	FOCAD	ENSG00000188352
191	FOSL1	ENSG00000175592
192	FOXD1-AS1	ENSG00000247993
193	FOXF1	ENSG00000103241
194	FP236383.3	ENSG00000280441
195	FP236383.4	ENSG00000280614
196	FP236383.5	ENSG00000281181
197	FP671120.6	ENSG00000280800
198	FP671120.7	ENSG00000281383
199	FRG1BP	ENSG00000149531
200	G6PC3	ENSG00000141349
201	G6PD	ENSG00000160211
202	GABRA3	ENSG00000011677
203	GALM	ENSG00000143891
204	GAPDH	ENSG00000111640
205	GBA2	ENSG00000070610
206	GBP2	ENSG00000162645
207	GCAT	ENSG00000100116
208	GCHFR	ENSG00000137880
209	GDF11	ENSG00000135414
210	GEM	ENSG00000164949
211	GJA1	ENSG00000152661
212	GLIPR1	ENSG00000139278

213	GLIS3	ENSG00000107249
214	GLMP	ENSG00000198715
215	GLYCTK	ENSG00000168237
216	GNG11	ENSG00000127920
217	GNS	ENSG00000135677
218	GPAA1	ENSG00000197858
219	GPANK1	ENSG00000204438
220	GPR176	ENSG00000166073
221	GPX4	ENSG00000167468
222	GRINA	ENSG00000178719
223	GSTA4	ENSG00000170899
224	GTPBP2	ENSG00000172432
225	GXYLT2	ENSG00000172986
226	H1-4	ENSG00000168298
227	H3C2	ENSG00000286522
228	H3C7	ENSG00000277775
229	H4C1	ENSG00000278637
230	H4C3	ENSG00000197061
231	HACD4	ENSG00000188921
232	HAGLROS	ENSG00000226363
233	HEBP1	ENSG00000013583
234	HECW1	ENSG00000002746
235	HEY1	ENSG00000164683
236	HFM1	ENSG00000162669
237	HIPK2	ENSG00000064393
238	HMGA1	ENSG00000137309

239	HMGA2	ENSG00000149948
240	HMGN5	ENSG00000198157
241	HNMT	ENSG00000150540
242	HNRNPA1P7	ENSG00000215492
243	HOXB4	ENSG00000182742
244	HOXB5	ENSG00000120075
245	HOXB6	ENSG00000108511
246	HOXD-AS2	ENSG00000237380
247	HOXD11	ENSG00000128713
248	HOXD3	ENSG00000128652
249	HOXD4	ENSG00000170166
250	HOXD8	ENSG00000175879
251	HOXD9	ENSG00000128709
252	HRH1	ENSG00000196639
253	HSD11B1L	ENSG00000167733
254	HSD17B11	ENSG00000198189
255	HSD17B14	ENSG00000087076
256	ICAM2	ENSG00000108622
257	IFI16	ENSG00000163565
258	IGF2R	ENSG00000197081
259	IL18	ENSG00000150782
260	IL6R	ENSG00000160712
261	INSIG1	ENSG00000186480
262	INSR	ENSG00000171105
263	INTS6L	ENSG00000165359
264	IRS1	ENSG00000169047

265	ITFG2	ENSG00000111203
266	ITGA3	ENSG00000005884
267	ITGB3	ENSG00000259207
268	ITPRIP	ENSG00000148841
269	KCNMB2-AS1	ENSG00000237978
270	KDM4B	ENSG00000127663
271	KHSRP	ENSG00000088247
272	KLHL13	ENSG00000003096
273	KLHL21	ENSG00000162413
274	KMT2D	ENSG00000167548
275	KYNU	ENSG00000115919
276	LAMA1	ENSG00000101680
277	LDHB	ENSG00000111716
278	LDLR	ENSG00000130164
279	LEF1	ENSG00000138795
280	LERFS	ENSG00000234665
281	LHFPL3	ENSG00000187416
282	LINC00165	ENSG00000261706
283	LINC00649	ENSG00000237945
284	LINC00960	ENSG00000242516
285	LINC01116	ENSG00000163364
286	LINC01605	ENSG00000253161
287	LINC01842	ENSG00000267147
288	LINC02454	ENSG00000256268
289	LINC02577	ENSG00000228742
290	LINC02709	ENSG00000245522

291	LIPE-AS1	ENSG00000213904
292	LONP1	ENSG00000196365
293	LOX	ENSG00000113083
294	LPCAT3	ENSG00000111684
295	LPIN1	ENSG00000134324
296	LRMDA	ENSG00000148655
297	LRP1	ENSG00000123384
298	LRP12	ENSG00000147650
299	LRP3	ENSG00000130881
300	LRRC8C	ENSG00000171488
301	LSM2	ENSG00000204392
302	LTBP1	ENSG00000049323
303	LTBR	ENSG00000111321
304	LUCAT1	ENSG00000248323
305	LYL1	ENSG00000104903
306	M6PR	ENSG00000003056
307	MAFIP	ENSG00000274847
308	MAGEA3	ENSG00000221867
309	MAGEA6	ENSG00000197172
310	MAMLD1	ENSG00000013619
311	MAN1A1	ENSG00000111885
312	MANBA	ENSG00000109323
313	MAP1B	ENSG00000131711
314	MAP2K2	ENSG00000126934
315	MAP3K7CL	ENSG00000156265
316	MAPKBP1	ENSG00000137802

317	MARCHF3	ENSG00000173926
318	MBD6	ENSG00000166987
319	MCOLN1	ENSG00000090674
320	MCRIP1	ENSG00000225663
321	MCTP1	ENSG00000175471
322	ME3	ENSG00000151376
323	MECOM	ENSG00000085276
324	MED16	ENSG00000175221
325	METTL1	ENSG00000037897
326	METTL27	ENSG00000165171
327	MEX3B	ENSG00000183496
328	MFSD12	ENSG00000161091
329	MICA	ENSG00000204520
330	MIDN	ENSG00000167470
331	MLLT3	ENSG00000171843
332	MME	ENSG00000196549
333	MMP3	ENSG00000149968
334	MRC2	ENSG00000011028
335	MROH5	ENSG00000226807
336	MRPL54	ENSG00000183617
337	MSRB3	ENSG00000174099
338	MTHFR	ENSG00000177000
339	MXRA7P1	ENSG00000232320
340	MYADM	ENSG00000179820
341	MYDGF	ENSG00000074842
342	NAPA	ENSG00000105402

343	NAV3	ENSG00000067798
344	NCLN	ENSG00000125912
345	NCR3LG1	ENSG00000188211
346	NDUFS7	ENSG00000115286
347	NECTIN3	ENSG00000177707
348	NELFE	ENSG00000204356
349	NELL2	ENSG00000184613
350	NEU1	ENSG00000204386
351	NFIB	ENSG00000147862
352	NFIC	ENSG00000141905
353	NFKBIL1	ENSG00000204498
354	NKX2-5	ENSG00000183072
355	NME1-NME2	ENSG00000011052
356	NPIPA5	ENSG00000183793
357	NR1D1	ENSG00000126368
358	NRP1	ENSG00000099250
359	NRP2	ENSG00000118257
360	NRROS	ENSG00000174004
361	NT5E	ENSG00000135318
362	OAF	ENSG00000184232
363	OAZ1	ENSG00000104904
364	OMA1	ENSG00000162600
365	ORAI3	ENSG00000175938
366	OS9	ENSG00000135506
367	P3H1	ENSG00000117385
368	PAK1	ENSG00000149269

369	PALLD	ENSG00000129116
370	PAX6	ENSG00000007372
371	PCDHB9	ENSG00000177839
372	PDE4A	ENSG00000065989
373	PDIA3	ENSG00000167004
374	PFKM	ENSG00000152556
375	PHB2	ENSG00000215021
376	PHF1	ENSG00000112511
377	PHF19	ENSG00000119403
378	PHLDA1	ENSG00000139289
379	PHLDA2	ENSG00000181649
380	PI3	ENSG00000124102
381	PICSAR	ENSG00000275874
382	PIGO	ENSG00000165282
383	PIP4P2	ENSG00000155099
384	PIR	ENSG00000087842
385	PKIA	ENSG00000171033
386	PLCB1	ENSG00000182621
387	PLEKHO1	ENSG00000023902
388	PLIN3	ENSG00000105355
389	PLTP	ENSG00000100979
390	POLR2E	ENSG00000099817
391	POPDC3	ENSG00000132429
392	POR	ENSG00000127948
393	POU6F1	ENSG00000184271
394	PPAN-P2RY11	ENSG00000243207

395	PPP1R10	ENSG00000204569
396	PPP1R14B	ENSG00000173457
397	PPP1R14B-AS1	ENSG00000256940
398	PPP1R14BP3	ENSG00000179967
399	PPP1R15A	ENSG00000087074
400	PPP1R18	ENSG00000146112
401	PRDM8	ENSG00000152784
402	PREX1	ENSG00000124126
403	PRKD1	ENSG00000184304
404	PRNP	ENSG00000171867
405	PRR13	ENSG00000205352
406	PRR16	ENSG00000184838
407	PRR5L	ENSG00000135362
408	PRRC2A	ENSG00000204469
409	PRSS23	ENSG00000150687
410	PSAP	ENSG00000197746
411	PSKH1	ENSG00000159792
412	PSMC1P5	ENSG00000250273
413	PTN	ENSG00000105894
414	PTPRG	ENSG00000144724
415	PTPRH	ENSG00000080031
416	PVR	ENSG00000073008
417	RAI14	ENSG00000039560
418	RASA3	ENSG00000185989
419	RBPMS	ENSG00000157110
420	RBPMS2	ENSG00000166831

421	RIPPLY2	ENSG00000203877
422	RN7SK	ENSG00000283293
423	RN7SL1	ENSG00000276168
424	RN7SL2	ENSG00000274012
425	RNA5-8SN1	ENSG00000278189
426	RNA5-8SN2	ENSG00000278233
427	RNA5-8SN3	ENSG00000275215
428	RNA5-8SN4	ENSG00000276700
429	RNA5-8SN5	ENSG00000274917
430	RNF122	ENSG00000133874
431	RNF41	ENSG00000181852
432	RNU2-2P	ENSG00000222328
433	RNU4-1	ENSG00000200795
434	RNU4-2	ENSG00000202538
435	ROBO1	ENSG00000169855
436	RPL13P5	ENSG00000240370
437	RPL36	ENSG00000130255
438	RPPH1	ENSG00000277209
439	RPS28	ENSG00000233927
440	RPS28P7	ENSG00000227097
441	RPSAP58	ENSG00000225178
442	RRAGD	ENSG00000025039
443	RTL8B	ENSG00000212747
444	RUNX2	ENSG00000124813
445	RUSC2	ENSG00000198853
446	SATB1	ENSG00000182568

447	SATB2	ENSG00000119042
448	SCARNA12	ENSG00000238795
449	SCD	ENSG00000099194
450	SCPEP1	ENSG00000121064
451	SEMA3C	ENSG00000075223
452	SERF2	ENSG00000140264
453	SFXN2	ENSG00000156398
454	SH3BGRL	ENSG00000131171
455	SH3PXD2B	ENSG00000174705
456	SIRT6	ENSG00000077463
457	SLAIN1	ENSG00000139737
458	SLC11A2	ENSG00000110911
459	SLC16A2	ENSG00000147100
460	SLC16A9	ENSG00000165449
461	SLC24A1	ENSG00000074621
462	SLC26A10	ENSG00000135502
463	SLC35B2	ENSG00000157593
464	SLC35E3	ENSG00000175782
465	SLC38A6	ENSG00000139974
466	SLC43A1	ENSG00000149150
467	SLC7A11	ENSG00000151012
468	SLC9B2	ENSG00000164038
469	SMIM10L1	ENSG00000256537
470	SMIM29	ENSG00000186577
471	SMPD1	ENSG00000166311
472	SMPDL3A	ENSG00000172594

473	SNHG14	ENSG00000224078
474	SNORA53	ENSG00000212443
475	SNORD3A	ENSG00000263934
476	SNRPN	ENSG00000128739
477	SOAT1	ENSG00000057252
478	SOCS3	ENSG00000184557
479	SP5	ENSG00000204335
480	SPATS2	ENSG00000123352
481	SPHK2	ENSG00000063176
482	SPRED2	ENSG00000198369
483	SPRY2	ENSG00000136158
484	SPRYD3	ENSG00000167778
485	SQLE	ENSG00000104549
486	SQSTM1	ENSG00000161011
487	SREBF1	ENSG00000072310
488	SRGAP1	ENSG00000196935
489	SSBP2	ENSG00000145687
490	SSBP3	ENSG00000157216
491	STAT6	ENSG00000166888
492	STEAP1	ENSG00000164647
493	SUSD5	ENSG00000173705
494	SV2A	ENSG00000159164
495	TANGO6	ENSG00000103047
496	TARBP2	ENSG00000139546
497	TBC1D9	ENSG00000109436
498	TBL1X	ENSG00000101849

499	TBXA2R	ENSG00000006638
500	TCEAL8	ENSG00000180964
501	THSD1P1	ENSG00000285160
502	TICAM2	ENSG00000243414
503	TLN1	ENSG00000137076
504	TM7SF3	ENSG00000064115
505	TMBIM6	ENSG00000139644
506	TMEM132A	ENSG00000006118
507	TMEM135	ENSG00000166575
508	TMEM150A	ENSG00000168890
509	TMEM156	ENSG00000121895
510	TMEM158	ENSG00000249992
511	TMEM192	ENSG00000170088
512	TMSB15A	ENSG00000158164
513	TMSB4X	ENSG00000205542
514	TNFRSF1A	ENSG00000067182
515	TNFSF13	ENSG00000161955
516	TNRC6C	ENSG00000078687
517	TNS2	ENSG00000111077
518	TOB2P1	ENSG00000176933
519	TP53INP2	ENSG00000078804
520	TPGS1	ENSG00000141933
521	TPI1	ENSG00000111669
522	TPP1	ENSG00000166340
523	TRABD2A	ENSG00000186854
524	TRAF1	ENSG00000056558

525	TRAPPC5	ENSG00000181029
526	TRERF1	ENSG00000124496
527	TRIB1	ENSG00000173334
528	TRPS1	ENSG00000104447
529	TSPAN31	ENSG00000135452
530	TSPAN4	ENSG00000214063
531	TSPAN9	ENSG00000011105
532	TTC28	ENSG00000100154
533	TUBA1A	ENSG00000167552
534	TUBA1C	ENSG00000167553
535	TYMP	ENSG00000025708
536	Telomerase-vert	ENSG00000277925
537	UBXN6	ENSG00000167671
538	UGT8	ENSG00000174607
539	UPP1	ENSG00000183696
540	USP13	ENSG00000058056
541	UST	ENSG00000111962
542	VARS1	ENSG00000204394
543	VEGFA	ENSG00000112715
544	VEGFB	ENSG00000173511
545	VPS18	ENSG00000104142
546	VPS28	ENSG00000160948
547	WDR45	ENSG00000196998
548	WLS	ENSG00000116729
549	WNK1	ENSG00000060237
550	WNT5B	ENSG00000111186

551	ZBED1	ENSG00000214717
552	ZBTB10	ENSG00000205189
553	ZC4H2	ENSG00000126970
554	ZEB1	ENSG00000148516
555	ZEB2	ENSG00000169554
556	ZFPM2-AS1	ENSG00000251003
557	ZIC2	ENSG00000043355
558	ZIC5	ENSG00000139800
559	ZNF114	ENSG00000178150
560	ZNF358	ENSG00000198816
561	ZNF486	ENSG00000256229
562	ZNF711	ENSG00000147180
563	ZNF749	ENSG00000186230

Table S7. Potentially malignant genes in BLCA progression.

Gene_name	Gene_id
AC008875.3	ENSG00000287263
ACVR1	ENSG00000115170
AHNAK2	ENSG00000185567
APLN	ENSG00000171388
ARL2	ENSG00000213465
ATP2B1	ENSG00000070961
BBOX1-AS1	ENSG00000254560
CEBPB	ENSG00000172216
CXCL3	ENSG00000163734

DGLUCY	ENSG00000133943
ETNK1	ENSG00000139163
FAR2	ENSG00000064763
FGF13	ENSG00000129682
GTPBP2	ENSG00000172432
HIPK2	ENSG00000064393
IGF2R	ENSG00000197081
IL6R	ENSG00000160712
IRS1	ENSG00000169047
KYNU	ENSG00000115919
LINC02577	ENSG00000228742
LOX	ENSG00000113083
LUCAT1	ENSG00000248323
ME3	ENSG00000151376
MMP3	ENSG00000149968
MYADM	ENSG00000179820
NRP1	ENSG00000099250
PAK1	ENSG00000149269
PIR	ENSG00000087842
PPP1R15A	ENSG00000087074
PRSS23	ENSG00000150687
PSAP	ENSG00000197746
PTPRH	ENSG00000080031
PVR	ENSG00000073008
SLC7A11	ENSG00000151012
SREBF1	ENSG00000072310

TMEM156	ENSG00000121895
UST	ENSG00000111962
ZBTB10	ENSG00000205189
ZFPM2-AS1	ENSG00000251003
